# Proceedings of the 4th IPLeiria’s International Health Congress: Leiria, Portugal. 11-12 May 2018

**DOI:** 10.1186/s12913-018-3444-8

**Published:** 2018-09-13

## Keynote lectures

### S1 The role of practice-based research in stimulating educational innovation in healthcare

#### Sandra Hasanefendic (s.hasanefendic@vu.nl)

##### Vrije Universiteit Amsterdam, De Boelelaan 1105, 1081 HV Amsterdam, the Netherlands

**Background**

Practice-based research is not uncommon in healthcare. In fact, the way nurses and doctors train is through extensive and intensive practice [1]. In other words, practice-based research has been used to gain new knowledge partly by means of practice and the outcomes of that practice [2]. Practice based research networks have also been gaining on importance in healthcare as ways of addressing research questions informed by practicing clinicians. They aim to gather data and improve existing practices of primary care [3], practice-based research is not only about gaining new knowledge via practice and improving existing practices.

**Objective**

In this presentation/paper I explain and highlight the role of practice-based research as an instrument for educational innovation in healthcare sciences.

**Methods**

I used interview excerpts and examples of projects related to healthcare at different universities of applied sciences in the Netherlands and Germany (also known as polytechnics in Portugal) to advance the role of practice-based research in educational innovation. This type of research is an integral part of teaching and curricular assignments in the healthcare settings in the Netherlands and Germany, and particularly at universities of applied sciences. I emphasized how practice-based research can improve and enrich the curricula, while at the same time, building necessary skills of future healthcare professionals and improving practices in already existing healthcare institutions.

**Results**

I show that practice-based research is in fact short term problem-oriented research which serves educational purposes by upgrading students’ and teachers’ skills and knowledge of the profession and dynamics in the work environment; which also has the potential to improve company products or design solutions and at the same time contribute to local and regional innovation in professions and profession related institutions [4-5]. Its role is multidimensional and dialectic insofar it serves multitude goals and is accomplished in dialogue among relevant stakeholders [6]. Practical suggestions for healthcare educators and practitioners in designing their curricula to incorporate the basic elements of this practice-based research are also offered in this presentation/paper.

**Conclusions**

Practice-based research is more than knowledge acquisition via practice. Its role and goals expand to enriching educational curricula with a more comprehensive engagement of external and professional stakeholders, at the same time contributing to student soft and professional skill development and solving stakeholder problems or optimizing services and products at local or regional levels.

**References**

1. Westfall JM, Mold J, Fagnan L. Practice-based research—“Blue Highways” on the NIH roadmap. Jama, 2007; 297(4): 403-406.

2. Andrews JE, Pearce KA, Ireson C, Love MM. Information-seeking behaviors of practitioners in a primary care practice-based research network (PBRN). Journal of the Medical Library Association, 2005;93(2):206.

3. Hartung DM, Guise JM, Fagnan LJ, Davis MM, Stange KC. Role of practice-based research networks in comparative effectiveness research. Journal of comparative effectiveness research. 2012;1(1):45-55.

4. Frederik H, Hasanefendic S, Van der Sijde P. Professional field in the accreditation process: examining information technology programmes at Dutch Universities of Applied Sciences. Assessment & Evaluation in Higher Education. 2017, 42(2): 208-225.

5. Hasanefendic S. Responding to new policy demands: A comparative study of Portuguese and Dutch non-university higher education organizations. [Doctoral Thesis]. Vrije Universiteit Amsterdam, the Netherlands. 2018.

6. Hasanefendic S, Heitor M, Horta H. Training students for new jobs: The role of technical and vocational higher education and implications for science policy in Portugal. Technological Forecasting and Social Change. 2016; 113: 328-340.

**Keywords**

Practice-based research, Short term, Problem oriented, Healthcare, Universities of applied sciences.

### S2 Is sexuality a right for all? Sexual revolution in the old age

#### Francisco J Hernández-Martínez (francisco.hernandezmartinez@ulpgc.es)

##### Universidad de Las Palmas de Gran Canaria, 35001 Las Palmas de Gran Canaria, España

**Background**

*“Do not you think your grandmother has sex? What happens with old gays? Why does a kiss between two elders tenderizes us and we do not think it is erotic”* (interview, Ricardo Iacub, 2018). It still impacts us, and what do we do with it? Do we let it pass? Do we encourage them?

Throughout the centuries, sex has been postulated as the impulse that gives life to people. This word, of Latin origin, has always aroused much interest in society and in all stages of life; but it must be differentiated from “sexuality”, because it contemplates various aspects among which it is found; sex, identities and gender roles, eroticism, pleasure, intimacy, reproduction and sexual orientation [1-6]. Sexuality is a vital dimension that is present in all stages of life, at least since adolescence. It contributes significantly to health and quality of life and is, moreover, a right recognized by international organizations such as the World Health Organization (WHO) [4, 7-9]. Despite this, old age has traditionally been considered as a stage in which sexual needs would be absent, in which people are no longer interested or have the capacity to lead an active sexual life [3-8, 11]. Master and Johnson, two famous American sexologists, argued that older people should fight against a false belief, which considers that “*sexual incompetence is a natural component of the aging process*”. This belief limits access to sexuality due to fear of failure, to consider that it is no longer correct, that it can be sick or perverse. The same authors pointed out that many of their patients had gone to priests, rabbis, doctors or psychologists and that they had received the answer “*it is logical at their age*” [3, 7, 10]

The studies carried out, in our country and internationally, show that the majority of the elderly, and especially those who have a partner, are still sexually active until very old ages [6-9]. The keys to continue carrying and enjoying a quality sexual life in old age should be recognized and admitted at a social level, and among others, we should start; to be free of prejudices and stereotypes that condemn the elderly to lack of desire, or that associate sexuality in old age to something dirty or morally condemnable. Stop associating youth and sexuality. Do not assume the possible problems or difficulties that may appear as irreversible barriers. Age influences the decrease in sexual activity and interest, but not in satisfaction. It is demonstrated that sex and sexuality play an important role in healthy and full aging [1-3, 6-9, 11]. Taking into account these premises, throughout the presentation will present the results of a study conducted in the Canary Islands among people over 65 years, users of senior centers whose main objective was; obtain data on sexual activity, sexuality and whether age-related pathologies have affected their sexual relations. Against these prejudices, older adults need, want and seek some kind of loving exchange; “*Old people want and need to talk about sex*”, and also young people need to think that we have a lifetime to continue enjoying and experimenting with our sexuality.

**References**

1. Baudracco CP, Romero M. Derecho e Igualdad para la comunidad trans se llama Ley de Identidad de Género. En Derecho a la Identidad. Ley de Identidad de Género y Ley de Atención Integral de la Salud para Personas Trans. Buenos Aires: Federación Argentina LGBTTI; 2011.

2. Uchôa YS, Costa DA, Silva Jr. IAD, Saldanha ST, Silva ES, Freitas WMTM, Soares SS (2016). A sexualidade sob o olhar da pessoa idosa. Rev. Bras. Geriatr. Gerontol. 2016;19(6):939-949.

3. Iacub R. Erótica y Vejez. Perspectivas de Occidente. Ed. Paidós S.A.C.F. Buenos Aires. 2006.

4. Foucault M. Historia de la sexualidad, Buenos Aires: Ed. Siglo Veintiuno; 2010.

5. Grosman CP. et al. Los adultos mayores y la efectividad de sus derechos. Nuevas realidades en el derecho de familia. Rubinzal-Culzoni Editores. Buenos Aires; 2015.

6. Guadarrama R, Ortiz Zaragoza MC, Moreno Castillo YC, González Pedraza AA. Características de la actividad sexual de los adultos mayores y su relación con su calidad de vida. Revista de Especialidades Médico-Quirúrgicas. 2010;15(2):72-79.

7. Hernandez-Martínez FJ, Jiménez-Díaz JF, Rodriguéz-de-Vera BC, Quintana-Montesdeoca MP, García-Caballero A, Rodrigues A. Tapersex en ancianos: ¿Existe el placer después de?. En libro de Actos del XX Congreso de la Sociedad Española de Geriatría y Gerontología. Valladolid. 2013.

8. López Sánchez F. Sexualidad y afectos en la vejez. Madrid: Ediciones Pirámide; 2012.

9. Martín Hernández M, Renteria Díaz P, Sardiñas Llerenas E. Estados clínicos y autopercepción de la sexualidad en ancianos con enfoque de género. Revista Cubana de Enfermería, 2009;25(1-2).

10. Masters W, Johnson V. Incompatibilidad sexual humana, Inter Médica, Buenos Aires; 1976

11. Pérez Martínez VT. Sexualidad humana: una mirada desde el adulto mayor. Revista Cubana de Medicina General Integral. 2008:24(1).

**Keywords**

Active Aging, Sexuality, Elderly, Sexual activity and benefits.

### S3 Promoting independent living in frail older adults by improving cognition and gait ability and using assistive products – MIND&GAIT Project

#### João Apóstolo (apostolo@esenfc.pt)

##### The Health Sciences Research Unit: Nursing, Portugal Centre for Evidence Based Practice: A Joanna Briggs Institute Centre of Excellence, Nursing School of Coimbra, 3000-232 Coimbra, Portugal

**Background**

Frail older adults are more susceptible to falls, fractures, disability, dependency, hospitalization and institutionalization [1]. Physical and cognitive decline, associated with frailty, potentiate the development of geriatric syndromes and lead to a decrease in self-care, depressive vulnerability and a decrease in quality of life [2]. Adapted physical exercise and cognitive stimulation allow the maintenance of physical and cognitive capacities, which is reflected in an improvement on the functional status of the elderly and in a reduction of associated comorbidities [3].

**Objective**

To promote independent living in frail older adults by improving cognition and gait ability and using assistive products.

**Methods**

It is planned to develop a combined intervention that will be composed by a digital cognitive stimulation program and an adapted physical exercise program. It is also being developed an auto-blocking kit mechanism for rolling walkers as an assistive product that could be used during the physical exercise program. A randomized-controlled trial will be developed to test the efficacy of the combined intervention in frail older adults. At the same time, a web platform will be developed and it will be used as a repository, providing digital intervention’ materials and results.

**Results**

Through the implementation of a multidisciplinary strategy, significant benefits are expected in the prevention and maintenance of physical and cognitive decline of the frail older adults. It is hoped that for frail older adults the combined intervention and its digital components would be synonymous of autonomy and improvement of their quality of life, contributing to active aging. The project, being based and tested in clinical practice, will guide health professionals, caregivers and general public to promote the independence of this population.

**Conclusions**

Cognitive interventions and physical exercise have impact on cognitive decline, a condition that assumes more importance once it is related with frailty in older adults. This multidisciplinary strategy gives the opportunity to older adults to act actively in their health through the spontaneous performance of cognitive and physical exercises available on the web platform. The components of the combined intervention will allow better reintegration of this population into society of today’s world. By promoting research policies among educational institutions and health service delivery institutions, the MIND & GAIT project will make health care available to the frail elderly population more accessible to professionals, caregivers and general public.

**Trial Registration**

NCT03390478

**Acknowledgements**

The current abstract is being presented on behalf of a research group. It is also part of the MIND&GAIT project Promoting independent living in frail older adults by improving cognition and gait ability and using assistive products, which is a Portuguese project with the support of COMPETE 2020 under the Scientific and Technological Research Support System, in the co-promotion phase. We acknowledge The Health Sciences Research Unit: Nursing (UICISA: E) of the Nursing School of Coimbra, the Polytechnic Institute of Leiria, the Polytechnic of Santarém, Polythecnic of Coimbra and also to other members, institutions and students involved in the project.

**References**

1. Apóstolo J, Holland C, O'Connell MD, Feeney J, Tabares-Seisdedos R, Tadros G et al. Mild cognitive decline. A position statement of the Cognitive Decline Group of the European Innovation Partnership for Active and Healthy Ageing (EIPAHA). Maturitas. 2016;83:83-93.

2. Apóstolo J, Cooke R, Bobrowicz-Campos E, Santana S, Marcucci M, Cano A et al. Effectiveness of interventions to prevent pre-frailty and frailty progression in older adults: a systematic review. JBI Database of Systematic Reviews and Implementation Reports. 2018;16(1):140–232.

3. Mewborn CM, Lindbergh CA, Miller LS. Cognitive interventions for cognitively healthy, mildly impaired and mixed samples of older adults: a systematic review and meta-analysis of randomized-controlled trials. Neuropsychology Rev. 2017;27(4):403-439.

**Keywords**

Aged, Cognitive decline, Cognitive stimulation, Frailty, Physical exercise.

### S4 Electronic health records in Portugal

#### Cristiana Maia (academia@spms.min-saude.pt)

##### Serviços Partilhados do Ministério da Saúde, 1050-189 Lisboa, Portugal

In the digital transformation Era, there is an increasing need to provide systems capable of offering functionalities that allow the user a quicker and easier access to healthcare related information’s. These digital services aim to provide access to more information, allowing the users to make better informed decisions.

In Portugal’s National Health Service Portal (SNS Portal www.sns.gov.pt), there are already several digital services available, the Citizen’s Area aggregates these services for the user.

Citizen’s Area main objectives is to facilitate communication and interaction between Citizens, Professionals and Health Institutions, allowing access to information in an integrated way, providing a better healthcare. Simple and accessible to all users, this area allows personal health information access in one place at any time, thus avoiding unnecessary commuting. This area has access monitoring and permission policy configurations, allowing the Citizen to view access history and configure access permissions to their health information, thus increasing control and management of their own personal health information.

Health Literacy is actively promoted though multiple initiatives, in dedicated areas accessible in SNS Portal and Citizen’s Area.

**Keywords**

Citizen’s Area, SNS Portal, Healthcare, Digital Services, Literacy.

### S5 Economic crisis and inequalities in the Southern European health systems

#### Mauro Serapioni (mauroserapioni@ces.uc.pt)

##### Centro de Estudos Sociais, Universidade de Coimbra, 3000-104 Coimbra, Portugal

**Background**

Despite the overall increase in living standards and the introduction of universal health systems, many studies have identified persistent inequalities in all industrialized countries. The Southern European countries, namely Greece, Italy, Portugal and Spain, although the reforms of the 1970s and 1980s introduced universal national health services, social inequalities in health only became a critical issue in the late 1990s. However, the issue of health inequalities became a priority from 2010-2011, when (although with different degrees of severity) the four countries began to feel the first effects of the financial crisis. Various studies have identified the impact of the economic crisis on the most vulnerable population groups, with increasing rates of mental health disorders and a rise in suicides.

**Objective**

After a brief contextualization of the welfare state in southern European countries and a characterization of health systems in Greece, Spain, Italy and Portugal, the main health inequalities are described, identifying the potential inequity induced by the reform processes undertaken and the current austerity policies implemented.

**Methods**

The study resulted from a non-systematic literature review, based on the Scoping review proposal. A total of 74 publications were analysed.

**Results and Discussion**

The analysis has highlighted common characteristics and trends in the Southern European health systems, as well as some significant differences between them. In the four countries, the social gradient (particularly in education, income, and work status) is the principal determinant factor in health inequalities. Another key aspect is the steady increase in out-of-pocket spending in health as a percentage of total health spending in all four countries, markedly in Greece and Portugal. The analysis has identified potential inequalities induced by the reform processes, as result of new relations between the public and private sectors in services provision. Another example of how health systems produce inequalities is the rising proportion of users’ health expenses covered by co-payments and user fees. Geographic inequality in health is another critical issue, observed in all four Southern European countries. Finally, the recent debate in the international literature on the relationship between different welfare state regimes and health inequalities will be discussed.

**Conclusions**

The crisis and austerity policies have greatly increased the level of dissatisfaction with healthcare provision in these countries.

**Keywords**

Health Inequalities, Health Systems, Economic Recession, Southern European Countries.

### S6 CBmeter- a new medical device for early screening of metabolic diseases

#### Maria P Guarino^1,2,3^, Gabriel Brito^1,4^, Marlene Lages^1^, Rui Fonseca-Pinto^1,4^, Nuno Lopes^1,4^

##### ^1^Center for Innovative Care and Health Technology, Polytechnic Institute of Leiria, 2411-901 Leiria, Portugal; ^2^School of Health Sciences, Polytechnic Institute of Leiria, 2411-901 Leiria, Portugal; ^3^Chronic Diseases Research Center, NOVA Medical School, 1150-082 Lisbon, Portugal; ^4^School of Technology and Management, Polytechnic Institute of Leiria; 2411-901 Leiria, Portugal

###### **Correspondence:** Maria P Guarino (maria.guarino@ipleiria.pt)

**Background**

Type 2 diabetes mellitus (T2DM) is a highly prevalent disease worldwide which is asymptomatic in about 44% of patients being critical to search for new ways of early diagnosis. Recent studies have demonstrated that the etiology of this disease may be associated with alterations in the function of the carotid body (CB), a chemosensor organ located within the bifurcation of the carotid artery. In animal models of metabolic syndrome, it was observed that the CBs are overactivated, underlying diseases such as obesity, hypertension and T2DM. This discovery provided a new paradigm in the neuroendocrinology field, suggesting that diagnostic function of the CBs has predictive value for the development of metabolic diseases. Despite this fact, it is not common in clinical practice to look at the CBs as organs associated with endocrine dysfunction and we believe this is probably due to the nonexistence of a user-friendly, portable medical device that diagnosis the function of the CBs.

**Objective**

The general aim of this work is to develop a novel device that evaluates the function of the carotid bodies - a CBmeter. We are also developing a standard test meal to be used as a physiological dynamic test during CBmeter utilization.

**Methods**

This medical device will synchronously assess several physiological variables: heart rate, respiratory rate, blood pressure variation, arterial pulse oximetry and circulating glucose, as well as the physiological responses to hyperoxia and meal ingestion. The results obtained will be analyzed using MatLab, in order to develop an algorithm with predictive value for early diagnosis of metabolic diseases. We are also developing a standard test mixed- meal to assess post-prandial glucose excursions with the CBmeter. The work is currently in the prototype development phase.

**Results**

A preliminary pilot-test performed with the prototype revealed that all the proposed variables are assessable with the CBmeter. The standardized test meal used in the pilot-test caused a glucose excursion curve that stabilized 30 minutes after ingestion, being suitable for metabolic evaluation with the CBmeter. Interstitial glucose variation was 16.6mg/dl glucose with a latency time of 21min. Heart rate did not vary significantly after the meal ingestion.

**Conclusions**

The CBmeter prototype is currently optimized to be used in a medical device clinical-trial with healthy volunteers. The mixed meal developed has proven to be suited in healthy volunteers to determine variations in CB-related cardiorespiratory parameters.

**Acknowledgements**

Project funded by FCT/SAICT-POL/23278/2016

**Keywords**

Carotid body, Diabetes, Early diagnosis, Medical device.

### S7 Help to care for users and caregivers: Help2care

#### Maria dos Anjos Coelho Rodrigues Dixe^1,2^ (maria.dixe@ipleiria.pt)

##### ^1^Center for Innovative Care and Health Technology, Polytechnic Institute of Leiria, 2411-901 Leiria, Portugal; ^2^School of Health Sciences, Polytechnic Institute of Leiria, 2411-901 Leiria, Portugal

There are several studies showing that the family members providing care to their relatives need to acquire abilities that enable them to be competent in their performance, having the health care professionals an indispensable role in their training [1]. Empowering caregivers can help in reducing health care costs, improve the quality of life of both user and caregiver [2], their mental health [3] and greater satisfaction with their care [4]. The continued support to caregivers can help them in decision making in less serious heath situations and to use fewer health services [5].

The main aims are: to construct assessment instruments to evaluate the patient and caregivers needs and abilities concerning self-care; to develop a support manual accessible to all caregivers; to make videos that demonstrate technics and task procedures to support the caregiver in the caring process; to develop a digital platform where all the developed resources will be available (website and app) to support the care transition from the hospital to the residence integrating professionals from the hospital and from the primary healthcare services; to empower health professionals to use the caregivers’ and users’ self-care empowerment model.

This project will include participation of students, teachers, researchers and stakeholders throughout the project using an action research, where, as the materials are developed, the population target acceptance will be tested, justifying the corrections needed before moving to the next step, using a consistent methodology with an action and learning research process. Population: The population will be: dependent patients diagnosed with a chronic illness, total or partial dependency admitted to the Hospital and require caregiver after hospital discharge; Informal Caregivers whose dependent members of the family present the criteria laid up and Heath professional. To evaluate the patient and caregivers’ needs and capacity concerning self-care we will construct them (activity 1). During the pilot test period we will have, two kinds of metrics: Qualitative metrics available on (http://garyperlman.com/quest/). And quantitative monitoring metrics for the use of the mobile app, including retention rate, churn rate, daily active users (DAU), daily sessions per DAU and stickiness, and also access statistics per module/feature on the app.

The mains output will be: A training model of caregivers and users for self-care composed with: a caregiver’s. support manual; a digital platform and a manual with the empowerment model to be used by health professionals

**Acknowledgements**

The current abstract is being presented on behalf of a research group. It is also part of the Help2care - project: Help to care for users and caregivers, which is a Portuguese project with the support of COMPETE 2020 under the Scientific and Technological Research Support System, in the co-promotion phase. We acknowledge the Polytechnic of Leiria, the Polytechnic of Santarém, Polythecnic of Castelo Branco, Centro Hospitalar de Leiria and also to other members, institutions and students involved in the project.

**References**

1. Clarke DJ, Hawkins R, Sadler E, Harding G., McKevitt C, Godfrey M, Dickerson J, Farrin, A.J, Kalra L, Smithard D, Forster. A .Introducing structured caregiver training in stroke care: findings from the TRACS process evaluation study. BMJ Open. 2014;4:1-10.

2. Cheng HY., Chair SY, Chau JP. The effectiveness of psychosocial interventions for stroke family caregivers and stroke survivors: A systematic review and meta-analysis. Patient Education and Counseling. 2014;95:30-44.

3. Legg, LA, Quinn TJ, Mahmood F, Weir CJ, Tierney J, Stott DJ, Smith L.N, Langhorne P. Non-pharmacological interventions for caregivers of stroke survivors. The Cochrane Database of Systematic Reviews. 2014. 10. CD008179. Doi: 10.1002/14651858

4. Bakas T, Farran CJ, Austin JK, Given BA, Johnson EA, Williams LS. Content Validity and Satisfaction With a Stroke Caregiver Intervention Program. Journal of nursing Scholarship. 2009;41(4):368-375.

5. Pierce L, Steiner VL, Khuder SA, Govoni, AL, Horn LJ. The effect of a Web-based stroke intervention on carers' well-being and survivors' use of healthcare services. Disability and Rehabilitation. 2009;31(20):1676-1684.

**Keywords**

Transitions of care, Caregivers, Self-care, Users.

### S8 TeenPower: e-Empowering teenagers to prevent obesity

#### Pedro Sousa^1,2^ (pedro.sousa@ipleiria.pt)

##### ^1^Center for Innovative Care and Health Technology, Polytechnic Institute of Leiria, 2411-901 Leiria, Portugal; ^2^School of Health Sciences, Polytechnic Institute of Leiria, 2411-901 Leiria, Portugal

**Background**

Adolescent obesity has reached epidemic proportions, being urgent to find effective prevention strategies. The core components of classic prevention programs have been unable to obtain the desired adherence. The solution may involve a more extensive and frequent contact with the healthcare team and the use of alternative communication channels and interacting/dynamic technologies with adolescents. TeenPower is a transdisciplinary practice-based action research project that aims to develop innovative interventions to promote healthy behaviors. This project is promoted by the polytechnics of Leiria, Santarém and Castelo Branco, Município de Leiria (City Council), as well as local schools and primary healthcare stakeholders, key partners in the development phase and in the implementation of the intervention program.

**Objective**

The main goal is the development, implementation and evaluation of a program for the promotion of healthy behaviors and prevention of obesity in adolescence, based on e-therapy and sustained by the case management methodology. The project is directed to the cognitive-behavioral empowerment of adolescents, through increased and interactive contact between adolescents and a multidisciplinary healthcare team. The use of Information and Communication Technologies (ICT) in the intervention can optimize resources and maximize impact, as a complement to conventional approaches.

**Methods**

The project includes the development of three complementary studies: (S1) evaluation of adolescents’ health status and cognitive-behavioral indicators, (S2) usability evaluation of the TeenPower platform and mobile app (S3) implementation and adherence evaluation to the TeenPower intervention program. Participants will be recruited from the school groups of Leiria, Santarém and Castelo Branco, aged between 12 and 16, with easy access to internet and smartphone/tablet (inclusion criteria). Intervention include behavioral, nutritional and physical activity counselling (online and face-to-face psycho-educative sessions). The e-therapeutic platform and mobile app (TeenPower) includes educational resources, self-monitoring, social support, interactive training modules and motivational tools. In addition to the case manager, the program will also have the direct support of an interdisciplinary team (nurse, nutritionist, exercise physiologist, among others).

**Results**

Expected results includes the delivery of the TeenPower intervention program, including an interactive application (web and mobile); scientific papers, communications and reports; workshops and conferences. We will evaluate adolescents’ health status and cognitive-behavioral indicators, evaluate Teenpower usability and program adherence.

**Conclusions**

The positive evaluation of the intervention program will stimulate the inclusion of ICT in the promotion of salutogenic behaviors and overweight prevention, creating technological interfaces that will allow customizing the intervention parameters and facilitating the monitoring and tracking.

**Acknowledgements**

The current abstract is presented on the behalf of a research group, the TeenPower research team. It is also part of the project TeenPower: e-Empowering teenagers to prevent obesity, co-funded by FEDER (European Regional Development Fund), under the Portugal 2020 Program, through COMPETE 2020 (Competitiveness and Internationalization Operational Program). We acknowledge the Polytechnic Institutes of Leiria, Santarém and Castelo Branco, the Municipality of Leiria (city council), and also other members, institutions and students involved in the project.

**Keywords**

Adolescents, Obesity, Health promotion, e-health, Mobile.

### S9 The Early Warning System for Basic School - SAPIE-EB in the promotion of school success, psychological health and career development

#### Pedro Cordeiro, Paula Paixão

##### Faculdade de Psicologia e de Ciências da Educação, Universidade de Coimbra, 3000-115 Coimbra, Portugal

###### **Correspondence:** Pedro Cordeiro (pedrcordeiro@gmail.com)

We present the “Sistema de Alerta Precoce do Insucesso Escolar no Ensino Básico (SAPIE-EB)”, a sophisticated early warning system that ealy flag the students’risk for school failure and ill-being, systematically monitor the students’ progress in the dimensions of and empirically assess the educational impact of the interventions in the dimensions of academic success, psychological health and career development. The SAPIE-EB is a user-friendly system that converts students’ raw data available at schools in knowledge, providing easy-delivered and intuitive reports on students’school failure, dropout and interventions, hereby allowing to deepen the knowledge about their causes and explanatory processes. The SAPIE-EB will be tested in 75 Portuguese basic schools. It is expected, with the implementation of the SAPIE-EB to reduce school retention in about 3% in a two-year interval period. Longitudinal research will attest the efficacy of the SAPIE-EB, from longitudinal quase-experimental research designs.

**Keywords**

Early Warning Systems, SAPIE-EB, School Success, Psychological Health, Career development.

## Oral Communications

### O1 Nursing professionals victims of verbal abuse by their coworkers from the same work unit

#### Maiara Bordignon, Inês Monteiro

##### School of Nursing, University of Campinas Campinas, 13083-887 Campinas, São Paulo, Brazil

###### **Correspondence:** Maiara Bordignon (bordignonmaiara@gmail.com)

**Background**

Violence among professionals of health teams has been exploited over the years with considerable attention to nursing [1-5]. The literature has also highlighted the impact of horizontal violence on the individual, unit and institution, such as, its negative influence on job satisfaction and possible harms to the safety culture, as well as to the wellbeing of professionals [3-5].

**Objective**

To present the frequency with which nursing professionals have suffered verbal abuse perpetrated by their coworkers, from the same work unit, during the last year and the professional categories involved in the abuse.

**Methods**

A cross-sectional study performed with a sample of 267 nursing professionals – registered nurse, nursing assistant/technician – working in Emergency units in Brazil. The experience of verbal abuse at work suffered by nursing professionals in the last 12 months was accessed using questions of a questionnaire about verbal abuse [6]. Data were described allowing to identify frequencies and professionals involved in the abuse. This study was authorized by institutions and approved by the Ethical Research Board of the university.

**Results**

Among the victims of verbal abuse, 23 (15%) nursing professionals reported that the last verbal abuse suffered was from coworkers of the same work unit, excluding abuses perpetrated by a boss or supervisor. At least twenty-one (91%) cases occurred in the emergency units that were part of the study and of these eighteen professionals indicated the perpetrator's profession, revealing that in nine (50%) cases the abuse was perpetrated by a coworker with the same profession, mostly registered nurse-to-registered nurse (5–56%). When the perpetrator's and the victim's profession were different (9–50%), the abuse occurred more frequently from nursing technician-to-registered nurse (3–33%). The presence of the doctor was identified in at least one situation of abuse occurred in the emergency units studied and was directed to a nursing technician. There was no report of verbal abuse perpetrated by other professionals of the nonmedical team and who did not participate in the nursing team.

**Conclusions**

Our study showed that in almost all instances verbal abuse occurred among the nursing staff professionals themselves. Organizational policies and strategies focusing on violence contributors factors in health care teams need to be structured to prevent violence among professionals, representing a challenge to health management.

**Acknowledgements**

Authors are grateful for the funding by grant#2016/06128-7, São Paulo Research Foundation (FAPESP), National Council for Scientific and Technological Development (CNPq) and Coordination for the Improvement of Higher Education Personnel (CAPES), Brazil.

**References**

1. Duffy E. Horizontal violence: a conundrum for nursing. Collegian. 1995;2(2):5-17.

2. McKenna BG, Smith NA, Poole SJ, Coverdale JH. Horizontal violence: experiences of Registered Nurses in their first year of practice. J Adv Nurs. 2003;42(1):90-96.

3. Longo J, Cassidy L, Sherman R. Charge nurses’ experiences with horizontal violence: implications for leadership development. J Contin Educ Nurs. 2016;47(11):493-499.

4. Purpora C, Blegen MA. Job satisfaction and horizontal violence in hospital staff registered nurses: the mediating role of peer relationships. J Clin Nurs. 2015;24(15-16):2286-94.

5. Armmer F, Ball C. Perceptions of horizontal violence in staff nurses and intent to leave. Work. 2015;51(1):91-7.

6. Bordignon M, Monteiro MI. Apparent validity of a questionnaire to assess workplace violence. Acta Paul Enferm. 2015;28(6):601-8.

**Keywords**

Workplace violence, Nurses, Nursing, Emergency Nursing.

### O2 Social representations of violence on the elderly: an injustice and a badness

#### Felismina RP Mendes^1,2^, Otília Zangão^1^, Tatiana Mestre^1^

##### ^1^Escola Superior de Enfermagem S. João de Deus, Universidade de Évora, 7004-516 Évora, Portugal; ^2^Centro de Investigação em Desporto, Saúde e Desenvolvimento Humano, Universidade de Évora, 7004-516 Évora, Portugal

###### **Correspondence:** Otília Zangão (otiliaz@uevora.pt)

**Background**

In contemporary society, ageing is a phenomenon that marks all developed societies. Portugal is one of the most aged countries in Europe. Currently, Portugal shows a life expectancy at birth of 81.3 years, an average value in terms of EU [1]. Social representations allow access to lay forms of thought, fundamental for understanding social phenomena and their consequences, and for the construction of scientific knowledge itself [2]. The Social Representations conduct “*the behaviors and the practices and, in this way, justify the positions taken and the behaviors*” [3]. Analyzing the social representations of violence on the elderly, from the current and past conceptions and daily practices of the elderly, allows us to have access to the dominant constructions in society about the social phenomenon that is violence and the way it is socially and individually expressed by its main actors.

**Objective**

To analyze the social representations of a group of elderly people about violence on the elderly and the reasons why this violence occurs.

**Methods**

Exploratory and descriptive research with qualitative approach, supported by Theory of Social Representations. It was attended by 237 elderly people aged 65-96 years, from the project “Ageing Safely in Alentejo” from University of Évora. The Free Speech Association technique was used and data were processed through qualitative data analysis software. All ethical procedures of human research were followed. Thus, all necessary authorizations for the study were requested, such as informed consent to the elderly. All conditions of anonymity and confidentiality of the responses obtained were also guaranteed.

**Results**

In social representations of violence on the elderly, the words most evoked by the elderly were injustice, to which were added: mistreatment, badness, bad, lack of respect, sadness, horrible and abandonment. In social representations about the reasons that lead to violence on the elderly, the words such as: lack of respect, lack of education and badness were predominant. These terms refer to the social devaluation of the elderly and their role in today's society, as in the representations about violence.

**Conclusions**

The social representations of these elderly people about violence and their reasons points to the stereotypes associated with the prevalent ageism in our society, where the social devaluation of the elderly dominates the daily life conceptions and practices.

**Acknowledgements**

This study was carried out under the ESACA - Ageing Safely in Alentejo - Ref: ALT20-03-0145-FEDER-000007, financed by Alentejo 2020, Portugal 2020 and EU.

**References**

1. PORDATA. Esperança de vida à nascença: total e por sexo - 2015 [Internet]. 2003 [cited 2017 Out 23]. p. 1–4. Available from: https://www.pordata.pt/Europa/Esperança+de+vida+à+nascença+total+e+por+sexo-1260.

2. Dantas, M., Abrão, F., Freitas, C. & Oliveira, D. Representações sociais do HIV/AIDS por profissionais de saúde em serviços de referência. Revista Gaúcha Enfermagem. [periódico na Internet]. 2014 [cited 2017 Set 05]; 35 (4): 94-100. Available from http://seer.ufrgs.br/index.php/RevistaGauchadeEnfermagem/article/view/45860/3 2387.

3. Mendes, F., Zangão, M., Gemido, M., & Serra, I. Representações sociais dos estudantes de enfermagem sobre assistência hospitalar e atenção primária. Revista Brasileira de Enfermagem. [periódico na Internet]. 2015 [cited 2017 Set 18]; 69 (2): 343-350. Available from http://www.redalyc.org/pdf/2670/267045808018.pdf.

**Keywords**

Social representations, Violence, Elderly, Elderly health, Discrimination.

### O3 Relationship between the use of new technologies and musculoskeletal symptoms in children and adolescents

#### Paula C Santos^1,2^, Sofia Lopes^1,3,4^, Rosa Oliveira^1^, Helena Santos, Jorge Mota^2^, Cristina Mesquita^1,4^

##### ^1^Department of Phisioterapy, School of Allied Health Technologies, Polytechnic Institute of Porto, 4050-313 Porto, Portugal; ^2^Research Centre in Physical Activity, Health and Leisure, Faculty of Sport, University of Porto, 4050-313 Porto, Portugal; ^3^Escola Superior de Saúde de Vale de Sousa, 4585-116 Gandra, Portugal; ^4^Centro de Estudos do Movimento e Atividade Humana, Escola Superior de Saúde, Instituto Politécnico do Porto, 4200-072 Porto, Portugal

###### **Correspondence:** Paula C Santos (paulaclara@ess.ipp.pt)

**Background**

Childhood and adolescence are determinants of musculoskeletal development, and the attitudes and habits adopted during these periods can have repercussions in adult life. The increasing use of technologies is becoming more worrying due to the sustained and prolonged postures due to the use of these devices and the consequent impact on musculoskeletal health.

**Objective**

This study analyzes the relationship between the use of new technologies with musculoskeletal symptoms (MSS) in children and adolescents.

**Methods**

Cross-sectional study with a sample of 460 students aged between 10 and 18 years. Data were collected through a questionnaire that included the Nordic Musculoskeletal Questionnaire.

**Results**

98.5% of students reported the used a mobile phone, 84.3% laptop and 52.4% tablet. Only 50.0% of the individuals who used mobile phones, 48.5% of the laptop and 31.1% of the tablet considered having a correct posture during the use of these technologies. We verified that the individuals with MSS showed more times of use of new technologies than individuals without MSS. There were differences in the time (min/day) of mobile phone and laptop use among children and adolescents, respectively 102.6 ± 121.47 *vs* 205.8 ± 175.89 (p < 0.001) and 74.0 ± 78.08 *vs* 117.9 ± 127.26 (p < 0.001).

**Conclusions**

Most students use new technologies in their daily lives, with less than half of them considering using these technologies in the right posture. It was also verified that individuals with MSS used more times new technologies than individuals without MSS. The time of use of new technologies increases with the age.

**Keywords**

New technologies, Musculoskeletal symptoms, Children and adolescents.

### O4 An ecological approach to fall risk factors for preventive interventions design: a pilot study

#### Jorge Bravo^1^, Hugo Rosado^1^, Felismina Mendes^3^, Catarina Pereira^2^

##### ^1^Nursing Department, São João de Deus Superior Nursing School, University of Évora, 7000-811 Évora, Portugal; ^2^Health Sciences and Human Development Center, Health and Sports Department, Science and Technology School, University of Évora, 7000-671 Évora, Portugal; ^3^Health Sciences and Human Development Center, São João de Deus Superior Nursing School, University of Évora, 7000-811 Évora, Portugal

###### **Correspondence:** Jorge Bravo (jorgebravo@uevora.pt)

**Background**

Recent literature reinforces that interventions for fall prevention should include multimodal training [1]. However, even multimodal training tends to focus on exercises separately in single physical, cognitive or environmental hazards variables. An ecological approach to explain phenomena’s such as fall occurrence, underlines not only the accumulative effect of isolated variables but also interactions between different variables.

**Objective**

To reduce a set of correlated variables to a smaller number that may explain fall occurrence.

**Methods**

187 older adults aged 65 to 96 years were assessed for falling risk factors. Principal component analysis (PCA) was performed including data from the 6-minute walk test (6MWT) [2], Gait Scale [3], Fullerton Advanced Balance Scale (FAB) [4], body composition - fat body mass percentage (FBM %), Mini-Mental State Examination (MMSE) [5], Environmental Hazards Scale (EH) [6], health conditions (HC), time up and go test (TUG) [2] and the Epworth Sleepiness Scale (ESS) [7]. Factors with eigenvalues of at least 1.0 were retained and a varimax rotation was used to produce interpretable factors. A binary regression analysis was performed using the forward stepwise (conditional) technique to identify the most significant components explaining fall occurrence. Receiver operating characteristics (ROC) curves were used to assess the discriminative ability of the logistic model.

**Results**

Three principal components were identified. In component 1, the dominant variables concerned physical and cognitive fit (6MWT, Gait Scale, FAB, MMSE, TUG), in component 2 dominant variables concerned health and environmental conditions (FBM %, EH, HC), whereas in component 3, the dominant variable concerned alertness (ESS). These components explained cumulatively 37%, 56% and 70% of the variance in fall occurrence. Logistic regression selected components 1 (OR: 0.527; 95% CI: 0.328–0.845) and 2 (OR: 1.614; 95% CI: 1.050–2.482) as predictive of falls. The cut-off level yielding the maximal sensitivity and specificity for predicting fall occurrence was set as 0.206 (specificity = 72.7%, sensitivity = 47.7%, and the area of the ROC curve was computed as 0.660 (95% CI: 0.564-0.756).

**Conclusions**

This pilot study showed that multiple correlated variables for fall risk assessment can be reduced to three uncorrelated components characterized by: physical and cognitive fit; health and environmental conditions; and alertness. The first two were the main determinants of falls. Recommendations: Interventions for fall prevention should privilege multimodal training including tasks that work simultaneously physical fitness, cognitive fitness and alertness, considering participant’s specific health and environmental conditions.

**Trial Registration**

NCT03446352

**References**

1. Hafström A, Malmström EM, Terdèn J, Fransson PA, Magnusson M. Improved balance confidence and stability for elderly after 6 weeks of a multimodal self administered balance-enhancing exercise program: a randomized single arm crossover study. Gerontology and geriatric medicine 2016;2:2333721416644149.

2. Rikli RE, Jones CJ. Development and validation of a functional fitness test for community-residing older adults. Journal of aging and physical activity 1999; 7(2): 12961.

3. Tinetti ME. Performance-Oriented Assessment of Mobility Problems in Elderly Patients. Journal of the American Geriatrics Society 1986; 34(2): 119-26.

4. Rose DJ, Lucchese N, Wiersma LD. Development of a multidimensional balance scale for use with functionally independent older adults. Archives of physical medicine and rehabilitation 2006; 87(11): 1478-85.

5. Guerreiro M, Silva AP, Botelho MA, Leitão O, Castro-Caldas A, Garcia C. Adaptação à população portuguesa da tradução do Mini Mental State Examination (MMSE). Revista Portuguesa de Neurologia 1994; 1(9): 9-10.

6. Tinetti ME, Speechley M. Prevention of Falls among the Elderly. New England Journal of Medicine 1989; 320(16): 1055-9.

7. Johns MW. A new method for measuring daytime sleepiness: the Epworth sleepiness scale. sleep 1991; 14(6): 540-5.

**Keywords**

Principal component analysis, Falling risk, Physical fitness, Cognitive fitness, Environmental hazards.

### O5 Relationship between smartphone use and musculoskeletal symptoms in adolescents

#### Paula C Santos^1,2^, Cristina Mesquita^1^, Rosa Oliveira, Raquel Azevedo, Sofia Lopes^1,3^

##### ^1^Department of Physiotherapy, School of Allied Health Technologies, Polytechnic Institute of Porto, 4050-313 Porto, Portugal; ^2^Research Centre in Physical Activity, Health and Leisure, Faculty of Sport, University of Porto, 4050-313 Porto, Portugal; ^3^North Polytechnic Institute of Health, 4585-116 Gandra, Portugal

###### **Correspondence:** Paula C Santos (paulaclara@ess.ipp.pt)

**Background**

We are currently facing a society of adolescents who are increasingly dependent on technology, in particular the smartphone, and this phenomenon can even lead to limiting situations in which the person’s physical well-being is called into question. Intensive use of the smartphone may contribute to a decrease in physical activity and generate musculoskeletal symptoms (MMS).

**Objectives**

To verify the existence of a relationship between the use of the smartphone and: 1) MMS; 2) vigorous, moderate and sedentary physical activity

**Methods**

An observational, analytical, cross-sectional study was conducted on a sample of 834 adolescents from five schools in the regions of Viseu, Vila Real and Porto. Data collection was performed through online questionnaires through the Qualtrics program, in order to perform the sociodemographic characterization of the sample and to determine behavioral habits related to health, as well as to the use of new technologies. Musculoskeletal symptoms were evaluated through the Portuguese version of the Nordic musculoskeletal questionnaire. (NMQ) and physical activity through the International Questionnaire of Physical Activity (IPAQ).

**Results**

The adolescents who used the smartphone for the most time referred MMS in the cervical (p < 0.001), thoracic (p = 0.017), lumbar (p < 0.001), shoulders (p < 0.001), wrists/hands (p = 0.003) and knees (p = 0.013). Adolescents who practice more vigorous physical activity (p = 0.023) use less smartphone, and those who have more time in sedentary physical activity (p = 0.008) use it more.

**Conclusions**

Adolescents who spend more time on smartphones refer more MMS. The use of the smartphone is associated with a more sedentary lifestyle, unlike the adolescents who practice vigorous physical activity that give less use to it.

**Keywords**

Smartphone, Physical Activity, Musculoskeletal Symptoms.

### O6 Functional fitness and cognitive performance in independent older adults – fallers and non-fallers: an exploratory study

#### Jorge Bravo^1^, Hugo Rosado^1^, Felismina Mendes^2^, Catarina Pereira^3^

##### ^1^São João de Deus Superior Nursing School, University of Évora, 7000-811 Évora, Portugal; ^2^Health Sciences and Human Development Center, São João de Deus Superior Nursing School, University of Évora, 7000-811 Évora, Portugal; ^3^Health Sciences and Human Development Center, Health and Sports Department, Science and Technology School, University of Évora, 7000-671 Évora, Portugal

###### **Correspondence:** Jorge Bravo (jorgebravo@uevora.pt)

**Background**

Actual research reinforces the importance of multimodal exercise programs for fall prevention; however remains unclear which components should be included in exercise programs, considering physical and cognitive components.

**Objectives**

This exploratory study aims to identify the associations between functional fitness (FF) and cognitive performance (CP) in independent older adults, regarding fallers and non-fallers.

**Methods**

63 males and 124 females (65-96 years) were selected based on the criteria of moderate or high functional independency (≥18 points) determined by responses to the 12-item of Composite Physical Functioning Scale [1]. FF was assessed by the Senior Fitness Test Battery [2]. A composite Z-score was created based on the individual scores for each fitness item. CP was assessed by the Mini-mental State Examination adapted for the Portuguese population [3]. Descriptive statistics were calculated for all outcome measurements and comparisons were performed using independent sample t-Tests. Multiple regression analyses were performed to test associations between FF and CP.

**Results**

T-test comparisons showed that females were more flexible than males (p < 0.05). Males were taller and heavier than females (p < 0.05). No differences were observed between these independent fallers and non-fallers sample. Multiple regression analyses were performed to understand the association of FF with CP in fallers and non-fallers. Agility was negatively associated with the MMSE score in fallers and non-fallers; however, after adjusting for gender, age and education, this association was not significant for non-fallers (p < 0.05). Lower body strength showed positive associations (p < 0.05) with the MMSE score exclusively in non-fallers, regardless the adjustments. Likewise, the upper body strength was positively associated with the MMSE score (p < 0.05) in non-fallers after adjusting for age, gender and education (p < 0.05). On the other hand, the upper body flexibility showed negative associations with the MMSE score (p < 0.05) however this association did not remain significant after adjusting for gender, age and education.

**Conclusions**

Independent older adults with higher agility scores were more likely to have an improved CP, whether they are fallers or non-fallers. Body strength, particularly improved lower body strength, is associated with higher CP in non-faller older adults, independently of age, gender and education. This exploratory study increases the spectrum of research in multimodal programs by suggesting that agility and strength training should be included in exercise prescription for fall prevention, in order to foment CP.

**Acknowledgements**

Study was funded by Horizon 2020, Portugal 2020 (ALT20-030145-FEDER-000007).

**References**

1. Rikli RE, Jones CJ. The reliability and validity of a 6-minute walk test as a measure of physical endurance in older adults. Journal of aging and physical activity 1998; 6(4): 363-75.

2. Rikli RE, Jones CJ. Development and validation of a functional fitness test for community-residing older adults. Journal of aging and physical activity 1999; 7(2): 129-61.

3. Guerreiro M, Silva AP, Botelho MA, Leitão O, Castro-Caldas A, Garcia C. Adaptação à população portuguesa da tradução do Mini Mental State Examination (MMSE). Revista Portuguesa de Neurologia 1994; 1(9): 9-10.

**Keywords**

Aging, Physical fitness, Accidental falls, Cognitive aging.

### O7 Association between endurance of the trunk extensor muscles and the risk of falling in community-dwelling older adults

#### Sofia Flora, Ana Tavares, Joana Ferreira, Nuno Morais

##### School of Health Sciences, Polytechnic Institute of Leiria, 2411-901 Leiria, Portugal

###### **Correspondence:** Sofia Flora (sofiiaflora@gmail.com)

**Background**

Falls in the elderly are a serious health problem and the result of the complex interaction between individual and environmental risk factors. Balance is considered a key factor for higher falling risk in this population [1, 2]; thus, assessment and preventive/rehabilitation programs targeting the balance control system are currently a clinical guideline [1]. Programs commonly include strength/power training of the lower limbs and trunk muscles and postural control exercises [1, 3]. Recently it has been shown that the elderly reach premature muscle fatigue during upright stance tasks [2], and that fatigue leads to poor balance control [4]. Muscular endurance hence appears to play an important role in the efficiency of the balance control system, particularly during performance of long lasting functional tasks. However, the association between muscle endurance and balance control measures has been overlooked, especially in the trunk muscles, despite its potential to assist clinicians and researchers to comprehensively screen falling risk factors and tailoring interventions accordingly.

**Objective**

The main purpose of this cross-sectional study was to determine the association between endurance of the trunk extensor muscles and the risk of falls in the elderly, considering possible co-factors such as age and BMI.

**Methods**

Community-dwelling adults ≥ 65 years were recruited from senior universities in the Centre region of Portugal. Exclusion criteria included severe physical/cognitive limitations that would prevent subjects from performing the testing protocol. Falling risk/balance was assessed using the Berg Balance Scale (BBS, score 0–56). Muscle performance was measured through the trunk extensor endurance test (in seconds). Simple and multiple linear regression analyses, using SPSS (v20), were conducted to predict the effects of muscle endurance, BMI and age on balance control. Statistical significance was set at 0.05.

**Results**

Fifty-nine volunteers (44 females, age = 71 ± 5 years, height = 1.60 ± 0.09 m, mass = 71.67 ± 14.35 kg, BMI = 28.02 ± 4.62 kg/m^2^) were included in the study. The largest correlation was found between the BBS score, and muscle endurance (*ρ* = 0.379), and BMI (*ρ* = -0.335). Muscle endurance predicted 7% of the BBS score (*r*^*2*^_*a*_ = 0.070, p = 0.024). When combined with BMI, muscle endurance accounted for ~16% (*r*^*2*^_*a*_=0.162, p = 0.003) of the total variance of the BBS score. Unsurprisingly, when age was added to the previous model the predictive capacity increased, reaching ~21% (*r*^*2*^_*a*_ = 0.214, p = 0.01).

**Conclusions**

Endurance of the trunk extensor muscles and BMI predicted approximately 16% of the BBS score. Since these are modifiable factors, it is recommended that they should be routinely included in the screening of falling risk factors in the elderly and addressed accordingly in preventive programs.

**References**

1. Phelan EA, Mahoney JE, Voit JC, Stevens JA. Assessment and management of fall risk in primary care settings. Med Clin North Am. 2015 Mar;99(2):281–93.

2. Pizzigalli L, Micheletti Cremasco M, Mulasso A, Rainoldi A. The contribution of postural balance analysis in older adult fallers: A narrative review. Journal of Bodywork and Movement Therapies. 2016 Apr;20(2):409–17.

3. Granacher U, Gollhofer A, Hortobágyi T, Kressig RW, Muehlbauer T. The Importance of Trunk Muscle Strength for Balance, Functional Performance, and Fall Prevention in Seniors: A Systematic Review. Sports Med. 2013 Apr 9;43(7):627–41.

4. Papa EV, Garg H, Dibble LE. Acute Effects of Muscle Fatigue on Anticipatory and Reactive Postural Control in Older Individuals. Journal of Geriatric Physical Therapy. 2015;38(1):40–8.

**Keywords**

Muscle Endurance, Balance, Elderly, Risk of falls, Trunk Extensor Muscles.

### O8 ICF Core Set for Obstructive Pulmonary Diseases: validation of the environmental factors component through the perspective of patients with asthma

#### Cristina Jácome^1,2^, Susan M Lage^3^, Ana Oliveira^2^, Augusto G Araújo^4^, Danielle AG Pereira^3^, Verônica F Parreira^3^

##### ^1^Centro de Investigação em Tecnologias e Serviços de Saúde, Faculdade de Medicina, Universidade do Porto, 4200-319 Porto, Portugal; ^2^Laboratório de Investigação e Reabilitação Respiratória, Escola Superior de Saúde, Universidade de Aveiro, 3810-193 Aveiro, Portugal; ^3^Universidade Federal de Minas Gerais, 31270-901 Belo Horizonte, Minas Gerais, Brasil; ^4^Hospital Carlos Chagas, 35900-595, Itabira, Minas Gerais, Brasil

###### **Correspondence:** Cristina Jácome (cristinajacome@ua.pt)

**Background**

To optimize a patient-oriented approach in asthma management, health professionals need to consider all aspects of the patient’s actual context (physical, social and attitudinal). This context can be assessed using the Environmental Factors component of the International Classification of Functioning, Disability and Health (ICF) Core Set for Obstructive Pulmonary Diseases (OPD). The categories included in the Environmental factors component have been selected by respiratory experts, and have been validated from the perspective of physicians, physiotherapists and patients with chronic obstructive pulmonary disease. However, the validation from the perspective of patients with asthma will be essential to allow a more widespread application of the ICF in this population.

**Objective**

This study aimed to validate the Environmental factors component of the Comprehensive and Brief versions of ICF Core Set for OPD from the perspective of patients with asthma.

**Methods**

A cross-sectional qualitative study was conducted with outpatients with asthma using semi-structured individual interviews. Qualitative data were analysed through the meaning condensation procedure by two researchers with expertise in the ICF.

**Results**

Thirty-five participants (26 females; 41±13 years) were included. Eight (35%) categories contained in the Environmental factors component of the Comprehensive version of the ICF Core Set for OPD and 4 (100%) of those contained in the Brief version were confirmed by the participants. Additionally, 5 second level categories (Products and technology for employment; Flora and fauna; Natural environment and human-made changes to environment, unspecified; Domesticated animals; Support and relationships, unspecified) and 13 third level categories (Food; Drugs; General products and technology for personal use in daily living; General products and technology for employment; Assistive products and technology for employment; Design, construction and building products and technology for gaining access to facilities in buildings for private use; Plants; Animals; Temperature; Humidity; Indoor air quality; Outdoor air quality; Health services) not included in the Core Set were identified.

**Conclusions**

The Environmental factors component of the Brief ICF Core Set for OPD was fully supported by the perspective of patients with asthma, contrasting with only one third of the categories of the Comprehensive version. The categories included in the ICF Core Set that were not confirmed by the participants and the additional categories that were raised need to be further investigated in order to develop an instrument tailored to patients’ needs. This will promote more patient-centred assessments and rehabilitation interventions.

**Keywords**

Asthma, International Classification of Functioning, Disability and Health, Environmental factors, Patient’s perspective.

### O9 Attachment, self-compassion and mental health in the use of the internet to establish intimate relationships

#### Sónia C Simões^1,2^, Vanessa Vieira^1^, Mariana Marques^1^, Laura Lemos^1^

##### ^1^Instituto Superior Miguel Torga, 3000-132 Coimbra, Portugal; ^2^Centro de Estudos da População, Economia e Sociedade, 4150-171 Porto, Portugal

###### **Correspondence:** Sónia C Simões (soniasimoes@ismt.pt)

**Background**

Currently, to our knowledge, there is no research with Portuguese samples comparing the levels of attachment, self-compassion and psychopathological symptoms in subjects who use and do not use the Internet to establish intimate relationships.

**Objective**

The present study aimed to investigate how individuals, who use the Internet to establish intimate relationships, differ psychologically from individuals who do not use the Internet for this purpose.

**Methods**

We used the following scales: Experiences in Close Relationships (ERP), Self-Compassion Scale (SELFCS), Brief Symptom Inventory (BSI) and a short sociodemographic questionnaire. The sample consisted of 350 individuals of whom 284 used social networks to establish intimate relationships (I) and 66 did not use the Internet for this purpose (NI), with a mean age of 29.90 (SD = 7.41) for the I group and 30.72 (SD = 8.26) for the NI group. The majority of the sample was single in both groups (I: 83.5% vs. NI: 68.2%), heterosexual (I: 79.6% vs. NU: 93.9%), was attending or attended Higher Education (I: 66.5% vs. NI: 60.6%).

**Results**

We found that man used more, compared to woman, the Internet to establish intimate relationships, and that the individuals that used most the Internet for this purpose had a higher number of short-term intimate relationships, compared to the group of subjects that did not use the Internet for this purpose. We also found that individuals without a romantic relationship (single, separated/divorced or widowed) were who resorted to this type of online service. Differences between groups regarding attachment, self-compassion and psychopathology were not found. However, it is important to highlight the stronger associations between psychopathological symptoms (global severity index and BSI dimensions) with attachment and self-compassion in the group that did not use the Internet to establish intimate relationships, comparing to the group that reported using the Internet with this intention.

**Conclusions**

The results show the importance of deepening the research about the use of Internet to establish intimate relationships, since there are no studies concerning this area in Portugal.

**Keywords**

Self-compassion, Attachment, Psychopathology, Intimate relationships, Online dating.

### O10 The obsessive-compulsive symptomatology: its relation with alexithymia, traumatic experiences and psychopathological symptoms

#### Sónia C Simões^1,2^, Timóteo Areosa^1^, Helena Espírito-Santo^1^, Laura Lemos^1^

##### ^1^Instituto Superior Miguel Torga, 3000-132 Coimbra, Portugal; ^2^Centro de Estudos da População, Economia e Sociedade, 4150-171 Porto, Portugal

###### **Correspondence:** Sónia C Simões (soniasimoes@ismt.pt)

**Background**

Alexithymia has been reported more significantly in subjects with obsessive-compulsive disorder (OCD), since they have a hard time recognizing and describing their own emotions. It should also be noted that many individuals with OCD often report experiencing traumatic situations. However, to our knowledge there are no Portuguese studies on the relationship between OCD (or obsessive-compulsive symptomatology), traumatic experiences, and alexithymia, justifying the relevance of this study.

**Objective**

The following goals were outlined: 1) To study the relationship between psychopathological symptoms, alexithymia and traumatic experiences in clinical and non-clinical samples; 2) To study and compare the traumatic experiences, the levels of alexithymia, and the psychopathological symptoms in clinical and non-clinical samples; 3) Check the relations of variables such as age, gender, marital status, and literacy with the presence or absence of obsessive-compulsive symptomatology in order to identify potential confounding factors.

**Methods**

The total sample comprised 115 individuals aged between 18 and 64 years (M = 31.50; SD = 10.61). For the creation of the 2 groups in comparison, the Maudsley Obsessive Compulsive Inventory (MOCI) cut-off point was used, in which results above 10 indicated the presence of obsessive-compulsive symptomatology. The clinical group had 40 subjects, aged between 18 and 49 years (M = 27.03; SD = 7.68) and the nonclinical group had 75 subjects, aged between 18 and 60 years (M = 33.89; SD = 11.21). The research protocol included: MOCI, Traumatic Experiences Checklist (TEC), Toronto Alexithymia Scale and the Brief Symptom Inventory.

**Results**

The clinical sample presented more psychopathological symptomatology and higher values of alexithymia compared to the non-clinical sample. No differences were found between groups in the presence of traumatic experiences, but the clinical sample presented higher scores of sexual abuse and trauma in the family of origin. Finally, there was a greater number of statistically significant associations and of a stronger magnitude in the non-clinical sample among the studied variables, compared to the clinical sample, especially according to the TEC.

**Conclusions**

It was verified that traumatic experiences and alexithymia, in particular, might be factors associated with the onset of obsessive-compulsive symptomatology, being a future study area to understand if they will also be risk factors for developing OCD.

**Keywords**

Obsessive-compulsive symptomatology, Traumatic experiences, Alexithymia.

### O11 Loss of group immunity against measles

#### João MG Frade^1,2^, Carla Nunes^3^, João R Mesquita^4^, Maria SJ Nascimento^5^, Guilherme Gonçalves^1^

##### ^1^Multidisciplinary Unit for Biomedical Research, Institute of Biomedical Sciences Abel Salazar, University of Porto, 4050-313 Porto, Portugal; ^2^Center for Innovative Care and Health Technology, Polytechnic Institute of Leiria, 2411-901 Leiria, Portugal; ^3^Public Health Research Centre, National School of Public Health, Nova University, 1600-560 Lisboa, Portugal; ^4^Agrarian Superior School, Polytechnic Institute of Viseu, 3500-606 Viseu, Portugal; ^5^Laboratory of Microbiology, Department of Biological Sciences, Faculty of Pharmacy, University of Porto, 4050-313 Porto, Portugal

###### **Correspondence:** João MG Frade (joao.frade@ipleiria.pt)

**Background**

Vaccination coverage rates higher than 95% contribute to the so-called group immunity effect with regard to measles vaccination [1]. However, to guarantee such immunity, it is important that those vaccine coverage rates also correspond to levels of seropositivity of measles antibodies (specific IgG antibodies levels > 150 mIU/ml) higher than 95% [2].

**Objective**

This study intends to evaluate from which moment, after having been vaccinated with MMR II (triple viral vaccine against mumps, measles and rubella), 95% of the individuals have specific IgG antibodies (Anti-Measles-IgG) <150 mIU/ml.

**Methods**

A cross-sectional study was conducted on 190 individuals, born in Portugal after 1990, with records of vaccine history documented in the Individual Record of Vaccination (IRV) and in the Individual Health Bulletin (IHB). Specific IgG antibodies to measles virus (Anti-Measles-IgG) were measured using the commercial immunoassay Siemens Enzygnost®Anti-Measles Virus/IgG.

**Results**

Data were grouped into three birth cohorts: born between 1990 and 1993, born between 1994 and 1995 and born between 2001 and 2004. It was found that those born between 2001 and 2004 were those that presented the highest levels of protection against measles, less than 2% of these individuals had levels of protection below the 150 mIU /ml. The cohort born between 1994 and 1995 was the one that presented the lowest protection against the disease, in which more than 50% of the individuals were below the protection threshold (150 mIU/ml). The cohort born between 1990 and 1993 is an intermediate cohort, where more than 50% of individuals are above protection threshold, but with a significant percentage of seronegative individuals. ANOVA analysis and Tukey's multiple comparison analysis showed a statistically significant difference among the 3 birth cohorts (p < 0.001). The use of mathematical modelling showed that at the end of 9 years, after individuals have received MMR II, more than 95% individuals no longer presented specific IgG antibodies against measles virus (Anti-Measles-IgG) above 150 mUI/ml (p < 0.0001).

**Conclusions**

The time elapsed since the last MMR II vaccination seems to be associated with protection against measles. Nine years after MMR II, more than 95% of individuals are seronegative for the Specific IgG antibodies to measles virus.

**References**

1. Gonçalves G, Frade J Nunes C, Mesquita J R, Nascimento MSJ, Persistence of measles antibodies, following changes in the recommended age for the second dose of MMRvaccine in Portugal. Vaccine 33, 2015: 5057–63.

2. Gonçalves G, Nunes C, Mesquita JR, Nascimento MSJ, Frade J. Measles antibodies in cord blood in Portugal. Possible consequences for the recommended age of vaccination. Vaccine 34, 2016: 2750–57.

**Keywords**

Immunity, Measles, Vaccination.

### O12 Code Stroke in an emergency department - evaluation of results after 7 years of protocol implementation

#### Ilda Barreira^1^, Matilde Martins^2^, Leonel Preto^2^, Norberto Silva^1^, Pedro Preto^3^

##### ^1^Serviço de Urgência, Unidade Local de Saúde do Nordeste, 5301-852 Bragança, Portugal; ^2^Departamento de Enfermagem, Escola Superior de Saúde, Instituto Politécnico de Bragança, 5300-146 Bragança, Portugal; ^3^Serviço de Ortotraumatologia, Unidade Local de Saúde do Nordeste, 5301-852 Bragança, Portugal

###### **Correspondence:** Leonel Preto (leonelpreto@ipb.pt)

**Background**

Fibrinolysis reduces mortality and disability after an ischemic stroke, and its benefits are documented with level of evidence I [1]. The major goal of the Code Stroke (CS) is to treat the eligible cases by fibrinolysis, within the therapeutic window of 4.5 hours after symptom onset [2]. Thus, an emergency department must operate efficient mechanisms to receive, diagnose, treat or transfer patients with stroke [3].

**Objective**

The main objective was to evaluate the results of the CS protocol implementation in an Emergency Department (ED) of a hospital in the North of Portugal. As secondary objectives we aimed to: (I) Characterize the patients in sociodemographic and clinical variables; (II) Calculate the activation rate of CS protocol and the rate of fibrinolysis.

**Methods**

Retrospective descriptive analysis, using data from the Manchester triage system and other secondary source of information, of all patients with ischemic stroke, haemorrhagic stroke, and transient ischemic attack (TIA) admitted to the Emergency Department between January 1, 2010 and December 31, 2016. Socio-demographic data, care times, cardiovascular risk factors and other clinical variables were collected. The statistical analysis was performed by ANOVA, at 0.05 significance level.

**Results**

In the 7 years analysed, 1200 patients with cerebrovascular disease were admitted in the ED. Among these patients, 63.0% presented ischemic stroke, 17.3% haemorrhagic stroke and 19.8% TIA. The population was predominantly male (54.8%) and had a mean age of 77.4 (± 11.2) years. Stroke code was activated 431 times, covering 37.2% (n = 282) of ischemic stroke, and have received thrombolytic therapy 18.4% (n = 52) of these patients. Door-to-needle time was, in average, 69.5 minutes. Mean (± SD) NIHSS (National Institutes of Health Stroke Scale) score was 14.8 (± 5.2) before treatment, decreasing to 11.8 (± 6.0) at two hours post- fibrinolysis (p < 0.05). For all patients (N = 1,200), we obtained the following prevalence of risk factors: Hypertension (64.7%), dyslipidaemia (30.3%), diabetes (26.5%), atrial fibrillation 23.3%), obesity (12.9%), smoking (6.3%) and ischemic heart disease (5.9%). The 24-hour mortality rate was 0.9% for ischemic stroke, 10.6% for haemorrhagic stroke, and 0% for TIA.

**Conclusions**

High rates of activation protocol were obtained for acute ischemic stroke, but only 52 patients met the criteria for fibrinolysis. The high age and comorbidity of patients with ischemic disease and its origin, predominantly rural, may have influenced the therapeutic window and the eligibility criteria for fibrinolysis.

**References**

1. Jauch EC, Saver JL, Adams HP, Bruno A, Connors JJ, Demaerschalk BM, et al.Guidelines for the early management of patients with acute ischemic stroke: a guideline for healthcare professionals from the American Heart Association/American Stroke Association. Stroke. 2013;44(3):870-947.

2. Baldereschi M, Piccardi B, Di Carlo A, Lucente G, Guidetti D, Consoli D, et al. Relevance of prehospital stroke code activation for acute treatment measures in stroke care: a review. Cerebrovasc Dis. 2012;34(3):182-90.

3. Alonso de Leciñana M, Egido JA, Casado I, Ribó M, Dávalos A, Masjuan J, et al. Guidelines for the treatment of acute ischaemic stroke. Neurologia. 2014;29(2):102-22.

**Keywords**

Stroke, Emergency Service Hospital, Fibrinolysis, Outcome and Process Assessment.

### O13 SEMantic and PRAgmatic assessment platform for school-age children

#### Dulce Tavares, Eileen S Kay

##### Escola Superior de Saúde de Alcoitão, 2649-506 Alcabideche, Portugal

###### **Correspondence:** Dulce Tavares (mdtavares@essa.pt)

**Background**

Semantic and pragmatic skills are developed throughout life and are essential in the development of school and social learning. Upon entering school, learning to read and write is developed in two large areas of knowledge. The first implies capacities of recognition and decoding of written symbols of the word and vocabulary development and the second allows the understanding of what is read through inferential capacities and non-literal interpretation. Often, students with reading comprehension difficulties go unnoticed. It is easier to detect a child who reads slowly, syllable by syllable, or with mistakes than those who read fluently but without understanding the content. These difficulties only become evident when questions are asked about the text and when it is necessary to understand the questions of subjects such as mathematics or science. Thus, success to reach the National Curricular Plan can be compromised.

**Methods**

Material was developed to evaluate semantic and pragmatic skills in school-aged children. In semantics, aspects of syntagmatic and paradigmatic relations (lexical field, synonymy and antonyms) and paronymy are evaluated. In pragmatics, competences are evaluated such as inferences, comprehension of idioms and proverbs. This material will be placed on a platform that can be consulted and used by different professionals working with children. The items that constitute this material took into account the stages of language development and school level. The lexicon used is in the domain of European Portuguese.

**Results**

The 756 children who were assessed attended public and private schools in Portugal. The results show an increasing evolution of the lexical competences of the children, with significant differences between the different age groups in all tests. There were no significant differences between female and male except in the paronym test. Regarding the socio-professional level of the child's origin, it is verified that it is a differentiating factor of lexical competence because significant differences in all tests were observed regardless of the age of the child.

**Conclusions**

The authors concluded that it is of great importance to analyse lexical competence regarding the aspects of its organization, as it enables students to deal with academic tasks successfully, improving literacy as well as to be able to act in a systematic and productive way in the intervention with children with language disorders. The complexity and innovation of the pragmatic skills assessment (in European Portuguese) leads to this work to continue in development.

**Keywords**

Semantic, Pragmatics, Assessment, School age.

### O14 Quality of Life in Portugal – what factors can determine the QoL in people with Intellectual Disabilities and a great need of supports?

#### António Rodrigo, Sofia Santos, Fernando Gomes

##### Faculty of Human Kinetics, University of Lisbon, 1495-687 Cruz Quebrada, Portugal

###### **Correspondence:** António Rodrigo (antoniorodrigo92@gmail.com)

**Background**

In Portugal, the Quality of Life (QoL) concept has become increasingly relevant, leaving aside a vision that only focus on the person’s limitations to one that emphasizes the quality of interactions between personal characteristics and environmental demands, within a socioecological model. This new paradigm changes the approach to evaluation and planning individualized supports, regarding adults with Intellectual and Developmental Disability (IDD). Research shows an emerging interest in analysing what personal and environmental factors have impact in QoL of persons with IDD. Therefore, our main goal is to analyse how individual characteristics influence QoL of people with intellectual disability with greater need of supports.

**Methods**

The Portuguese version of the Escala San Martín, that focus on 8 QoL domains: Self-determination, Emotional Well-Being, Physical Well-Being, Material Well-Being, Rights, Personal Development, Social Inclusion and Interpersonal Relations was applied to 293 individuals with intellectual disabilities, over 18 years-old (32.31 ± 8.29), 128 females and 165 males. All participants were institutionalized. The dependent variables were the domains/QoL total scores and the independent variables were gender, diagnosis, age, comorbidities and tacking medication. A comparative study was carried out using either independent samples t-tests or the one-way analyses of variance (ANOVA).

**Results**

When comparing gender, age and medication consumption, no significant differences were found, with all groups presenting similar mean QoL scores. Regarding comorbidities, significant differences were found when comparing physical well-being (p < 0.001), rights (p < 0.001) and social inclusion (p = 0.001) domains, with higher mean QoL scores to those without comorbidities. Significant differences were also found regarding diagnosis, in all domains except for the material well-being. Higher mean scores were found in individuals with a mild intellectual disability diagnosis, when compared to those with severe or profound ID diagnosis.

**Conclusions**

The information about personal factors with impact in QoL will help to meet challenges and will allow a more adjusted person-centred planning. Discussion and implications for practice will be presented.

**Keywords**

Individualized supports, Intellectual and Developmental Disability, Quality of Life, Escala San Martín.

### O15 Relationship between the levels of functional capacity and family functionality and depression in the elderly

#### Andréia WB Silva, Akemi Izumi, Giovanna G Vietta, Márcia R Kretzer

##### Universidade do Sul de Santa Catarina, 88137-270 Palhoça, Santa Catarina, Brasil

###### **Correspondence:** Andréia WB Silva (andreiawb@hotmail.com)

**Background**

Depression is one of the most prevalent mental health problems among the elderly [1]. Functional limitations and changes in family dynamics characterize important risk factors for the onset of depression [1,2].

**Objective**

To analyse the relation between levels of functional capacity and family functionality and depression in the elderly.

**Methods**

A cross-sectional study was conducted including one hundred and thirty-eight (138) elderly individuals. The presence of depression, the levels of capacity to perform Basic Activities of Daily Living (BADL) and Instrumental Activities of Daily Living (IADL) and family functionality were assessed, respectively, by the Geriatric Depression Scale (GDS), Katz Index of Independence in Activities of Daily Living, Lawton Scale and family APGAR. The Statistical Package for Social Sciences (SPSS) version 18.0 was used to enter and analyse data (p < 0.05). The present study was approved by the Research Ethics Committee of UNISUL.

**Results**

The most prevalent characteristics were the age between 60 and 69 years (62.3%), the female gender (52.9%), the white ethnicity (87.0%), having accomplished up to 8 years of schooling (75.8%), and being retired (80.4%). 67.4% of the elderly did not have a spouse, and 14.5% lived alone. Depression presented a prevalence in 43.5% of the participants, of whom 88.3% were mildly depressive and 11.7% were severely depressive. There was a high frequency of hypertension (64.5%), Diabetes Mellitus (37.7%), osteoarthritis (39.1%), heart failure (28.3%), chronic obstructive pulmonary disease (15.9%) and asthma (9.4%). When evaluating functional capacity, 1.4% and 12.3% of the participants were classified as dependent to perform BADL and IADL, respectively. Family dysfunction was observed in 12.3% of the elderly (5.1% moderate dysfunction and 7.2% high dysfunction). When testing associations between depression and sociodemographic characteristics, the results showed statistical differences when comparing gender and marital status. Women were 1,538 times more likely to have depression than men (p = 0.031), and individuals who had a spouse were 1,580 times more likely to suffer from the disease (p = 0.018). When associating depression with other comorbidities, arterial hypertension was 1.652 times more prevalent (p = 0.024). Statistical differences were also identified with heart failure (PR = 1.941, p = 0.001) and asthma (PR = 1.923, p = 0.012). The functional capacity for BADL and IADL did not differ statistically. Family dysfunction was significantly associated with depression, which was 1,969 times more frequent in dysfunctional families (p = 0.003).

**Conclusions**

Depression in the elderly is associated with the female gender, having a spouse, cardiovascular and respiratory morbidities and family dysfunction.

**References**

1. Lima AMP, Ramos JLS, Bezerra IMP, Rocha R, Batista HHMT, Pinheiro WR. Depressão em idosos: uma revisão sistemática da literatura. Rev Epidemiol Controle Infecç. 2016;6(2):97-103.

2. Bretanha AF, Fachinni LA, Nunes BP, Munhoz TN, Tomazi E, Thumé E. Sintomas depressivos em idosos residentes em áreas de abrangência das Unidades Básucas de Saúde da zona urbana de Bagé, RS. Rev Bras Epidemiol. 2015;18(1):1-12.

**Keywords**

Depression in the elderly, Functional capacity, Family functionality.

### O16 Microbiological evaluation of hotel units swimming pools

#### Diana Assembleia, Teresa Moreira, António Araújo, Cecília Rodrigues, Marlene Mota, Manuela Amorim

##### Escola Superior de Saúde, Instituto Politécnico do Porto, 4200-072, Porto, Portugal

###### **Correspondence:** Manuela Amorim (mas@ess.ipp.pt)

**Background**

Swimming pools are currently operated by public and private entities for the development of sports, recreational and therapeutic activities [1]. For this reason, it is essential to guarantee the chemical and microbiological quality of the pool water, since they may be the origin of several pathologies [2].

**Objective**

The present research aimed to analyse data from the microbiological evaluation of indoor and outdoor swimming pool waters of Hotel Units of Mainland Portugal and Madeira in the year 2016, in order to verify the water quality.

**Methods**

A cross-sectional descriptive study was performed using database records from a northern laboratory. The microbiological parameters studied to characterize the indoor and outdoor swimming pool waters included CFU/mL of viable microorganisms at 37ºC/24h, CFU/100mL of total coliforms, CFU/100mL of *Escherichia coli*, CFU/100mL of *Enterococcus spp*., CFU/100mL of *Pseudomonas aeruginosa*, CFU/100mL of total *Staphylococcus* and CFU/100mL of coagulase producers *Staphylococcus*. The samples were characterized as conforming and non-conforming according to the reference intervals indicated in Regulatory Circular nº 14/DA of 21/08/2009 of Direção Geral de Saúde [1].

**Results**

Of the total of indoor pools (n = 610) analysed, 25.09% (n = 153) were classified as non-conforming, being the microorganisms viable at 37 ºC the most frequent cause of nonconformities (n = 105), followed by total coliforms and total *Staphylococcus* (n = 42 each). For the outdoor pools (n = 1982), 29.92% (n = 593) were also classified as non-conforming, once more being microorganisms viable at 37 ºC the most frequent cause of nonconformities (n = 420), followed by total coliforms (n = 154).

**Conclusions**

Indoor swimming pools have a lower frequency of nonconformities compared to outdoor swimming pools. The ambient temperature and the presence of soils around the pool influence the microbiological quality of the water [2]. These results also suggest that water treatment is not effective, indicating water pollution, being hygienic care other factor that influence the microbiological quality of the water [3]. The determination of these parameters is useful when microbiological monitoring is carried out constantly.

**References**

1. Direcção-Geral da Saúde. Normativa Circular Nº 14/DA de 21/08/2009. Programa de Vigilância Sanitária de Piscinas; 2009.

2. Rebelo H, Rodrigues R, Grossinho J, Almeida C, Silva M, Silva C, et al. Avaliação da qualidade da água de piscinas: estudo de alguns parâmetros bacteriológicos e físico-químicos. Boletim Epidemiológico Observações. 2014, 3(4):3-5.

3. World Health Organization. Guidelines for safe recreational water environments. Swimming pools and similar environments. Geneva: World Health Organization; 2006.

**Keywords**

Microbiological evaluation, Microbiological quality, Swimming pool waters, Fecal contamination indicators.

### O17 Normative values of functionality and quality of life of the portuguese healthy older people

#### Cátia Paixão^1^, Sara Almeida^1,2^, Alda Marques^1,2^

##### ^1^Respiratory Research and Rehabilitation Laboratory, School of Health Sciences, University of Aveiro, 3810-193 Aveiro, Portugal; ^2^Institute for Research in Biomedicine, University of Aveiro, 3810-193 Aveiro, Portugal

###### **Correspondence:** Alda Marques (amarques@ua.pt)

**Background**

The older population is increasing worldwide [1]. Since the average life expectancy is currently 71.4 years at birth and the healthy life expectancy is only 63.1 years, there is a need to enhance the focus on public health to promote health and healthy ageing [2, 3]. Measures of functionality and health-related quality of life (HRQoL) have been identified as predictors of healthy aging [4-6]. However, to interpret results from those measures, and compare them within a population or across populations, normative data are necessary [7-9].

**Objective**

To establish age and gender-related normative values for the Five Times Sit to Stand Test (5 STS), 10 Meter Walk Test (10MWT), and World Health Organization Quality of Life-Bref (WHOQoL-Bref) for Portuguese healthy older people.

**Methods**

An exploratory cross-sectional study was conducted. Participants were recruited from the community. Functionality was assessed with the 5STS [4, 10] and 10MWT [5, 11] and Health-related Quality of Life (HRQoL) with the WHOQoL-Bref (scores: 0-20 and 0-100) [6]. Descriptive statistics was used to determine normative scores by age decades (60-69; 70-79; 80-89 years) and gender. Differences between age and gender were explored with multiple comparison tests using the Bonferroni correction.

**Results**

118 older people (76.2 ± 8.9yrs; n = 79, 66.9% female) participated in this study. Mean scores of 5STS (9.4 ± 3.5s; 13.0 ± 4.9s; 16.7 ± 6.7s), 10MWT (5.4 ± 2.1s; 6.5 ± 3.1s; 12.4 ± 5.9s) increased with age. Mean scores of the different domains of the WHOQoL-Bref 0-20 scale: physical health (15.9 ± 2.6; 15.1 ± 2.2; 13.6 ± 2.3), psychological (15.6 ± 2.6; 15.0 ± 2.3; 13.9 ± 1.9), social relationships (15.8 ± 2.8; 14.6 ± 2.4; 13.5 ± 2.4), environment (16.4 ± 2.3; 16.0 ± 2.3; 15.1 ± 1.6) decreased with age. Similar findings were observed in the WHOQoL-Bref 0-100 scale: physical health (74.6 ± 16.4; 69.3 ± 13.5; 60.4 ± 14.2), psychological (72.6 ± 16.4; 68.4 ± 14.5; 61.8 ± 12.1), social relationships (73.6 ± 17.6; 66.4 ± 15.2; 59.6 ± 15.2) and environment (77.6 ± 2.3; 74.9 ± 14.6; 69.4 ± 10.2). Females showed worst results in all measures. Mean scores of most measures were significantly different among age decades and gender (p < 0.05).

**Conclusions**

This study provided normative values of 5STS, 10MWT and WHOQoL-Bref for the Portuguese healthy older people. These data may improve the utility of these measures for health professionals to screen people and develop tailored interventions to improve functionality and HRQoL in this population. Normative values of WHOQoL-Bref will also allow identifying vulnerable groups and describing the profile of HRQoL in Portuguese healthy older people living in the community.

**Acknowledgements**

This work was partial supported by Programa Operacional de Competitividade e Internacionalização (COMPETE), through Fundo Europeu de Desenvolvimento Regional (FEDER) and Fundação para a Ciência e Tecnologia (FCT) under the project UID/BIM/04501/2013.

**References**

1. WHO. Global health and aging. Geneva: World Health Organization. 2011.

2. Ageing H. Keystone for a Sustainable Europe–EU Health Policy in the Context of Demogrpahic Change. European Comission. 2007.

3. Fuchs J, Scheidt-Nave C, Hinrichs T, Mergenthaler A, Stein J, Riedel-Heller SG, et al. Indicators for healthy ageing—a debate. International journal of environmental research and public health. 2013;10(12):6630-44.

4. Marques A, Almeida S, Carvalho J, Cruz J, Oliveira A, Jácome C. Reliability, Validity, and Ability to Identify Fall Status of the Balance Evaluation Systems Test, Mini-Balance Evaluation Systems Test, and Brief-Balance Evaluation Systems Test in Older People Living in the Community. Arch Phys Med Rehabil. 2016;97(12):2166-73.

5. Marques A, Cruz J, Quina S, Regêncio M, Jácome C. Reliability, Agreement and Minimal Detectable Change of the Timed Up & Go and the 10-Meter Walk Tests in Older Patients with COPD. COPD: Journal of Chronic Obstructive Pulmonary Disease. 2016;13(3):279-87.

6. Huang T, Wang W. Comparison of three established measures of fear of falling in community-dwelling older adults: psychometric testing. Int J Nurs Stud. 2009;46(10):1313-9.

7. Rothstein JM, Echternach JL. Primer on measurement: an introductory guide to measurement issues, featuring the American Physical Therapy Association's standards for tests and measurements in physical therapy practice: Amer Physical Therapy Assn; 1993.

8. Bohannon RW, Andrews AW. Normal walking speed: a descriptive meta-analysis. Physiotherapy. 2011;97(3):182-9.

9. Mitrushina M, Boone KB, Razani J, D'Elia LF. Handbook of normative data for neuropsychological assessment: Oxford University Press; 2005.

10. Bohannon R. Reference values for the five-repetition sit-to-stand test: a descriptive metaanalysis of data from elders. Percept Mot Skills. 2006;103(1):215-22.

11. Bohannon R. Comfortable and maximum walking speed of adults aged 20-79 years: reference values and determinants. Age Ageing. 1997;26(1):15-9.

**Keywords**

Normative values, Functionality, Quality of Life, Portuguese healthy older people.

### O18 Relationship between balance and functionality, gait speed, physical activity and quality of life in community-dwelling older people

#### Sara Almeida^1,2^, Cátia Paixão^1^, Alda Marques^1,2^

##### ^1^Respiratory Research and Rehabilitation Laboratory, School of Health Sciences, University of Aveiro, 3810-193 Aveiro, Portugal; ^2^Institute for Research in Biomedicine, University of Aveiro, 3810-193 Aveiro, Portugal

###### **Correspondence:** Alda Marques (amarques@ua.pt)

**Background**

Balance is a modifiable risk factor for falls which represent a major public health problem for healthy ageing [1]. Predictors of healthy ageing in older people (i.e., functionality, gait speed, physical activity (PA) and health-related quality of life (HRQoL)) have been correlated with balance measures [2-5]. However, most balance measures do not assess the different components of balance hindering the design of interventions. To overcome this difficulty the Balance Evaluation System Test (BESTest) [6] and its short versions [7, 8] (new comprehensive measures of balance) were developed. Nevertheless, the relationship between the BESTest [6] and its short versions [7, 8] with functionality, gait speed, physical activity and health-related quality of life older people living in the community is still unknown.

**Objective**

To explore the relationship between the BESTest, Mini-BESTest and Brief-BESTest with functionality, gait speed, PA and HRQoL in community-dwelling older people.

**Methods**

An exploratory cross-sectional study was conducted. Community-dwelling older people (> 60 yrs) were recruited. Balance was assessed with the BESTest, Mini-BESTest and Brief-BESTest, functionality with the 5STS [9], gait speed with the 10MWT [10], PA with the Brief-PA questionnaire [11] and HRQoL with the WHOQoL-Bref [12]. Descriptive statistics was used to characterize the sample. Correlations were explored with the Spearman correlation coefficient. By convention, the interpreting size of a correlation coefficient was negligible (0.00-0.30), low (0.30-0.50), moderate (0.50-0.70), high (0.70-0.90) and very high (0.90-1.00) correlation [13].

**Results**

One hundred and eighteen older people living in the community (76.2 ± 8.9 years; n = 79, 66.9% female) participated in this study. On average participants were overweight, with high body mass index (male: 26.9 ± 4.2 kg/m^2^; female: 26.8 ± 4.3 kg/m^2^) and fat-free mass (male: 29.5 ± 6.3 %; female: 37.6 ± 6.2%). BESTest, Brief-BESTest and Mini-BESTest were I) low and negatively correlated with intense (-0.34; -0.37; -0.32, respectively) and moderate (-0.37; -0.37; -0.35, respectively) PA; II) moderate and negatively correlated with the 5STS (-0.51; -0.61; -0.59, respectively); III) moderate to high and negatively correlated with the 10MWT (-0.69; -0.77; -0.78) and IV) negligible to moderate and positively correlated with the WHOQoL-Bref domains (I-Physical health 0.46; 0.57; 0.53; II-Psychological 0.47; 0.52; 0.53; III-Social relationships 0.32; 0.36; 0.28; IV-Environment 0.46; 0.51; 0.46).

**Conclusions**

This study shows that there is a relationship between the BESTest and its short versions with functionality, gait speed and HRQoL in community-dwelling older people. Higher correlations were found in the short versions, especially with functionality measures. This is useful for clinical practice since these versions are simpler, require less material and are quicker to apply.

**Acknowledgements**

This work was partial supported by Programa Operacional de Competitividade e Internacionalização (COMPETE), through Funfo Europeu de Desenvolvimento Regional (FEDER) and Fundação para a Ciência e a Tecnologia (FCT) under the project UID/BIM/04501/2013.

**References**

1. WHO. Falls: fact sheet. Geneva; 2016.

2. Iwakura M OK, Shibata K, Kawagoshi A, Sugawara K, Takahashi H, et al. . Relationship between balance and physical activity measured by an activity monitor in elderly COPD patients. Int J Chron Obstruct Pulmon Dis. 2016;11:1505-14.

3. Ozcan A DH, Gelecek N, Ozdirenc M, Karadibak D. . The relationship between risk factors for falling and the quality of life in older adults. BMC Public Health. 2005;5:90.

4. Spagnuolo D JS, Iwama Â, Dourado V. Walking for the Assessment of Balance in Healthy Subjects Older than 40 Years. Gerontology. 2010;56(5):467-73.

5. Nilsagård Y AM, Carling A, Vesterlin H. Examining the validity and sensitivity to change of the 5 and 10 sit-to-stand tests in people with multiple sclerosis. Physiotherapy Research International. 2017;22(4):e1681.

6. Horak F, Wrisley D, Frank J. The Balance Evaluation Systems Test (BESTest) to Differentiate Balance Deficits. Phys Ther. 2009;89(5):484-98.

7. Franchignoni F, Horak F, Godi M, Nardone A, Giordano A. Using psychometric techniques to improve the Balance Evaluation Systems Test: the mini-BESTest. Journal of rehabilitation medicine. 2010;42(4):323-31.

8. Padgett P, Jacobs J, Kasser S. Is the BESTest at its best? A suggested brief version based on interrater reliability, validity, internal consistency, and theoretical construct. Phys Ther. 2012;92(9):1197-207.

9. Goldberg A, Chavis M, Watkins J, Wilson T. The five-times-sit-to-stand test: validity, reliability and detectable change in older females. Aging Clin Exp Res. 2012;24(4):339-44.

10. Peters D, Fritz S, Krotish D. Assessing the Reliability and Validity of a Shorter Walk Test Compared With the 10-Meter Walk Test for Measurements of Gait Speed in Healthy, Older Adults. J Geriatr Phys Ther. 2013;36(1):24-30.

11. Marshall A, Smith B, Bauman A, Kaur S. Reliability and validity of a brief physical activity assessment for use by family doctors. Br J Sports Med. 2005;39(5):294-7.

12. Kluthcovsky A, Kluthcovsky F. O WHOQOL-bref, um instrumento para avaliar qualidade de vida: uma revisão sistemática. Revista de Psiquiatria do Rio Grande do Sul. 2009;31(3).

13. Mukaka M. A guide to appropriate use of Correlation coefficient in medical research. Malawi Med J. 2012;24(3):69-71.

**Keywords**

Correlations, Balance, Healthy ageing predictors, Older people.

### O19 Trends of hospitalization for chronic obstructive pulmonary disease in Brazil from 1998 to 2016

#### Bárbara O Gama, Andréia WB Silva, Fabiana O Gama, Giovanna G Vietta, Márcia R Kretzer

##### University of Southern Santa Catarina, Campus Pedra Branca, 88137-270 Palhoça, Santa Catarina, Brazil

###### **Correspondence:** Bárbara O Gama (barbara.oenning@hotmail.com)

**Background**

Chronic Obstructive Pulmonary Disease (COPD) is a major public health problem. In Brazil, it is the fifth largest cause of hospitalization in the public health system when analysing patients over 40 years of age.

**Objective**

To analyse the trend of hospitalization for COPD in Brazil from 1998 to 2016.

**Methods**

Trend analysis of hospitalization for COPD were based on data from the “*Sistema de Informação Hospitalar do Departamento de Informática do Sistema Único de Saúde*” (DATASUS). Simple linear regression analysis, p < 0.05. Approved by the Ethics Committee of the Universidade do Sul de Santa Catarina (UNISUL).

**Results**

In the period, 3,403,536 hospitalizations for COPD were analysed in Brazil, with a strong tendency to reduce rates (β= -6,257, p < 0.001), from 166.2/100,000 inhabitants in 1998 to 56.7/100,000 inhabitants in 2016. Among the Brazilian regions, there were higher hospitalization rates in the southern region, 461.8/100,000 inhabitants in 1998 and 133.1/100,000 inhabitants in 2016, followed by the central-west region, 222.2/100,000 inhabitants for 61,6/100,000 inhabitants. The region with the lowest rates of hospitalization was the northeast, with 69.6/100,000 inhabitants at 36.7/100,000 inhabitants. There largest decreases in the southern region (β= -19.4). The trend is decreasing in both sexes, with the largest reduction in male (β = -6,976), which has the highest admission rates at the beginning and end of the period (180.7 and 60.6/100.000 inhabitants, respectively). All age groups analysed showed a significant reduction tendency, with the largest decreases in the age groups above 60 years. In the age group of 80 years or more, there was reduction from 3370.1/100,000 in 1998 to 1535.3/100,000 inhabitants in 2016 (β = -101,198). In females, the reduction was from 2089.1/100,000 inhabitants to 548.4/100,000 inhabitants (β = -84,372).

**Conclusions**

The trend of hospitalization for COPD in Brazil is decreasing. The southern region has the highest rates, as does the male sex. The age groups of 60 and older in both sexes present the highest rates of hospitalization, with increase proportional to the increase in age. The results indicate a change in the profile of this disease, which can be attributed to a greater coverage of the family health strategy, better monitoring of diagnosed cases, and free access to medicines dispensed by the SUS (*Sistema Único de Saúde*), which reduce exacerbations of the cases.

**Keywords**

Chronic Obstructive Pulmonary Disease, Hospitalization, Trends.

### O20 Temporal trend of hospitalization for acute myocardial infarction in the southern Brazilian states from 2008 to 2016

#### Jessica M Okabe, Bárbara O Gama, Pedro F Simão, Márcia Kretzer, Giovanna G Vietta, Fabiana O Gama

##### University of Southern Santa Catarina, Campus Pedra Branca, 88137-270 Palhoça, Santa Catarina, Brazil

###### **Correspondence:** Jessica M Okabe (jessica.okabe@gmail.com)

**Background**

Acute Myocardial Infarction (AMI) is responsible for high hospitalization rates in Brazil Southern regions and represents one of the major causes of morbidity and mortality.

**Objective**

To analyse the temporal trend of hospitalization for AMI in the southern Brazilian states from 2008 to 2016.

**Methods**

Ecological study of time series of hospitalization for AMI, with data from the Hospital Information System provided by the Department of Informatics of the Single System (CID 10 - code I 21.9) in the resident population of the States of Paraná (PR), Santa Catarina (SC) and Rio Grande do Sul (RS), according to sex and age group. Simple linear regression was performed, with p < 0.05. Study approved by the Research Ethics Committee of the Southern University of Santa Catarina.

**Results**

In the analysed period, there were 154,828 hospitalizations in the South region. There was an upward trend in rates, with an average annual increase of 4,261 hospitalizations per AMI/100,000 inhabitants. At the beginning of the period a rate of 82.65/100,000 inhabitants was registered and at the end a rate of 118.67/100,000 inhabitants. The same trend was observed in the three southern states. Paraná presented a rate of 15.57/100,000 in 2008, to 24.89/100,000 in 2016 (β = 1.064; p = 0.002). In Santa Catarina the rate ranged from 17.11/100,000 in 2008 to 28.23/100,000 in 2016 (β = 1.159; p < 0.001). Rio Grande do Sul presented the highest rates among states, from 21.98/100,000 in 2008 to 30.57/100,000 in 2016 (β = 1.156; p < 0.001). Both sexes had an upward trend (male β = 15.732; p < 0.001; female β = 8.553; p < 0.001), with a variation from 279.32 (2008) to 418.46/100,000 inhabitants (2016) among men; and from 165.76 to 238.05/100,000 inhabitants among women. It was observed that the age groups between 40-49 years and 70-79 years, in both sexes, presented an upward tendency of hospitalization rates. In the male age group between 40 and 49 years, the increase in hospitalization rate was 368.56/100,000 in 2008 to 475.21/100,000 in 2016 (β = 10.553; p = 0.01). Between 70-79 years there was an increase of 10791.40/100,000 to 12458.46/100,000 in this same period (β = 160.084; p = 0.04). In the female age group between 40-49 years the increase in hospitalization rate was from 168.11/100,000 in 2008 to 210.63/100,000 in 2016 (β = 5.184; p = 0.02). And between 70-79 years there was an increase from 4452.09/100,000 to 5130.12/100,000 in this same period (β = 78.868; p = 0.02).

**Conclusions**

The study showed an upward trend in hospitalization rates for AMI in the Southern Region, by sex and age groups above 30 years for both sexes. Males present the highest rates.

**Keywords**

Acute myocardial infarction, Trend, Hospitalization.

### O21 Temporal trend of the incidence of tuberculosis in the state of Santa Catarina from 2001 to 2015

#### Rafaela F Abreu, Bárbara O Gama, Pedro F Simão, Giovanna G Vietta, Márcia Kretzer, Fabiana O Gama

##### University of Southern Santa Catarina, Campus Pedra Branca, 88137-270 Palhoça, Santa Catarina, Brazil

###### **Correspondence:** Rafaela F Abreu (rafaelafujii@hotmail.com)

**Background**

World Health Organization (WHO) declared Tuberculosis (TB) as a global public health emergency. TB control is a priority in Brazil.

**Objective**

To analyse the temporal trend of incidence of Tuberculosis in the State of Santa Catarina from 2001 to 2015.

**Methods**

Ecological study of time series of TB incidence trends selected from the SINAN (Information System for Notifiable Diseases) of the Ministry of Health in the population residing in the State, by sex, age group and macro-regions. Simple linear regression was performed, p < 0.05. Study approved by the Research Ethics Committee of the Southern University of Santa Catarina.

**Results**

From 2001 to 2015, 30,213 TB cases were confirmed in Santa Catarina, with a steady trend in incidence rates, with 31.2/100,000 inhabitants in 2001 and 32.0/100,000 inhabitants in 2015 (p = 0.27). The male gender presented the highest rates, showing a strong tendency to increase, with an increase of 0.456 per year, ranging from 42.24/100,000 inhabitants in 2001 to 47.55/100,000 inhabitants, in the year 2015 (p < 0.001). In the male groups, aged from 0 to 19 years and from 20 to 29 years, a significant trend occurred in the increase of incidence rates, with an increase of 0.159 and 0.606, respectively, of the rate (p = 0.02) per year. The age group from 40 to 49 years, in turn, showed a decreasing trend, with a reduction of 1.292 in the incidence rate per year (p = 0.001). In females, there was a reduction of 0.802 in the rate per year in the age group of 20 to 29 years (p = 0.003). The macro-regions of the Midwest, Foz do Rio Itajaí and Plateau Norte presented a reduction in TB incidence rates. In the macro-regions of Greater Florianópolis and South, the trend was increasing (p < 0.05).

**Conclusions**

TB incidence rates in Santa Catarina are stationary. Growing trend in males. Growing trend in the male age groups up to 29 years and decreasing between 40 and 49 years. Decreasing trend in the female age group from 20 to 29 years. Macro-regions located in the coastal range have an increasing tendency and the macro-regions located in the Centre West of the State, a decreasing trend.

**Keywords**

Tuberculosis, Trend, Incidence.

### O22 Trauma, impulsivity, suicidality and binge eating

#### Ana C Ribeiro, Mariana Marques, Sandra Soares, Pedro Correia, Cidália Alves, Paula Silva, Laura Lemos, Sónia Simões

##### Instituto Superior Miguel Torga, 3000-132 Coimbra, Portugal

###### **Correspondence:** Ana C Ribeiro (anaclaudia_aac@hotmail.com)

**Background**

Binge eating is a public health problem with physical and psychological effects, throughout life. Several studies explored the association between some variables (e.g. shame) and binge eating symptoms, but it is important to continue exploring the contribution of other correlates.

**Objective**

Explore the association and the predictive role of traumatic experiences, impulsivity and suicidality with/to binge eating symptoms.

**Methods**

421 subjects from the general population and college students (women, n = 300, 71.3%) completed the Traumatic Events Checklist, the Binge Eating Scale, the Barratt Impulsiveness Scale and the Suicidality Scale.

**Results**

The values of punctual prevalence of binge eating symptoms were similar to those from recent national studies, having found a severe severity of 2.6% in the total sample (3.3% in women). In both genders, suicidality total score and the body mass index (BMI) associated with binge eating total score. Only in women this score correlated with sexual and family trauma total scores and with the total score of traumatic events. If in men suicidality total score associated with family trauma total score and with the total score of traumatic events; in women that score also correlated with sexual trauma total score. In men, binge eating total score associated to attentional impulsivity (one of the first order impulsivity factors) and, in women, to all the first order impulsivity factors (attentional impulsivity, motor and non-planning), and with all the second order impulsivity factors (psychological attention, cognitive instability, motor, self-control and cognitive complexity), with the exception of perseverance. In women, attentional impulsivity particularly associated with sexual and family trauma total scores and with the total score of traumatic experiences. In women, the BMI, suicidality and attentional impulsivity total scores were the binge eating total score predictors.

**Conclusions**

In a sample from the general population and college students, we found that it is salient and of importance for future interventions, mainly in women, the predictive role of BMI, suicidality and attentional impulsivity scores to binge eating symptoms, with traumatic events (a more distal correlate) revealing significant associations, but not predicting these symptoms.

**Keywords**

Traumatic events, Impulsiveness, Suicidality, Binge eating.

### O23 Computerised respiratory sounds during acute exacerbations of chronic obstructive pulmonary disease

#### Ana Oliveira^1,2,3^, Patrícia Rebelo^2,3^, Lília Andrade^4^, Carla Valente^4^, Alda Marques^2,3^

##### ^1^Faculty of Sports, University of Porto, Porto,4200-450 Portugal; ^2^Respiratory Research and Rehabilitation Laboratory, School of Health Sciences, University of Aveiro, 3810-193 Aveiro, Portugal; ^3^Institute for Research in Biomedicine, University of Aveiro, 3810-193 Aveiro, Portugal; ^4^Pulmonology Department, Baixo Vouga Hospital Center, 3810-501 Aveiro, Portugal

###### **Correspondence:** Ana Oliveira (alao@ua.pt)

**Background**

Timely treatment and adequate monitoring of acute exacerbations of chronic obstructive pulmonary disease (AECOPD) have shown to reduce hospital admissions and recovery time, while improving patients’ quality of life [1]. Nevertheless, this is challenging as AECOPD diagnosis/monitoring relies exclusively on patients’ reports of symptoms worsening [2]. AECOPD are characterised by an increased airway inflammation and obstruction, abnormal bronchial mucus and air trapping, which results in changes in lung acoustics [2,3]. Thus, changes in respiratory mechanics related with AECOPD may be successfully monitored by respiratory sounds, namely adventitious respiratory sounds (ARS, crackles and wheezes) [3]. Nevertheless, little is known on ARS changes during the time course of AECOPD.

**Objective**

To evaluate ARS changes during the time course of AECOPD.

**Methods**

25 non-hospitalised patients with AECOPD (16 males, 70.0 ± 9.8yrs, FEV1 54.2 ± 20.6% predicted) were enrolled. Patients were treated with pharmacological therapy. ARS at anterior and posterior right/left chest were simultaneously recorded at hospital presentation (T1) and at weeks 3 (T3) and 8 (T8). ARS (no. of crackles and wheeze occupation rate–%Wh) were processed, per respiratory phase, using validated algorithms [4,5]. Differences were examined with Friedman and Cochran tests and both tests were corrected with Bonferroni corrections.

**Results**

Significant differences were found in no. of inspiratory crackles (0.6 [0.1-2.2] vs. 0.5 [0.1-2.5] vs. 0.3 [0.0-0.9]; p = 0.008) in T1, T3 and T8 at posterior chest, namely participants presented more inspiratory crackles (p = 0.013) at T1 than at T8. Similar results were found for inspiratory %Wh (0.0 [0.0-12.3] vs. 0.0 [0.0-0.0] vs. 0.0 [0.0-0.0]; p = 0.019), namely, participants presented significantly more inspiratory %Wh at T1 than at T3 (p = 0.006). A significant higher number of participants presenting inspiratory wheezes was found at T1 than at T3 at the anterior chest (%Wh: 10 vs. 2 vs. 5; p=0.017) and a trend to significance was found at posterior chest (%Wh: 10 vs. 3 vs. 4; p = 0.052). No differences were found for the remaining variables.

**Conclusions**

Crackles and wheezes seem to be sensitive to monitor the course of AECOPD. Inspiratory crackles seem to persist until 15 days after the exacerbations (*i.e.*, approximate time needed to resolve AECOPD [6]) whilst inspiratory %Wh significantly decreased after this period. This information may allow further advances in the monitoring of patients with COPD across all clinical and non-clinical settings, as respiratory sounds are simple, non-invasive population-specific and available by nearly universally means. Further studies with larger samples and including data collected before the AECOPD are needed to confirm these findings.

**References**

1. Wilkinson TM, Donaldson GC, Hurst JR, Seemungal TA, and Wedzicha JA. Early therapy improves outcomes of exacerbations of chronic obstructive pulmonary disease. Am J Respir Crit Care Med 169: 1298-1303, 2004.

2. The Global Initiative for Chronic Obstructive Lung Disease. Global Strategy for Diagnosis, Management, and Prevention of 543 Chronic Obstructive Pulmonary Disease—2017 Report. The 544 Global Initiative for Chronic Obstructive Lung Disease, Inc.; 2017.

3. Gavriely N, Nissan M, Cugell DW, Rubin AH. Respiratory health screening using pulmonary function tests and lung sound analysis. Eur Respir Rev. 1994;7(1):35–42.

4. Pinho C, Oliveira A, Jácome C, Rodrigues JM, Marques A. Integrated approach for automatic crackle detection based on fractal dimension and box filtering. IJRQEH. 2016;5(4):34-50.

5. Taplidou SA, Hadjileontiadis LJ. Wheeze detection based on time-frequency analysis of breath sounds. Comput Bio Med. 2007;37(8):1073-83.

6. Seemungal TA, Donaldson GC, Bhowmik A, Jeffries DJ, Wedzicha, JA. Time course and recovery of exacerbations in patients with chronic obstructive pulmonary disease. Am J Respir Crit Care Med. 2000; 161(5): 1608-1613.

**Keywords**

Chronic Obstructive Pulmonary Disease, Acute exacerbations, Computerised respiratory sounds, Crackles, Wheezes.

### O24 Trauma, self-disgust and binge eating

#### Sandra Soares, Mariana Marques, Ana C Ribeiro, Pedro Correia, Cidália Alves, Paula Silva, Helena E Santo, Laura Lemos

##### Instituto Superior Miguel Torga, 3000-132 Coimbra, Portugal

###### **Correspondence:** Sandra Soares (sandra.soares.93@hotmail.com)

**Background**

Binge eating disorder is finally recognized in the current Diagnostic and Statistical Manual of Mental Disorders-5. Additionally, international and national studies explored correlate binge eating symptoms, but it is important to evaluate the role of other variables for these symptoms, in the general population.

**Objective**

Explore the association and predictive role of traumatic experiences and of self-disgust with/in binge eating symptoms, exploring, also, the possible mediation role of self-disgust in the relation between traumatic experiences and those symptoms.

**Methods**

421 subjects from the general population and college students (women, n = 300, 71.3%) completed the Traumatic Events Checklist, the Binge Eating Scale and the Multidimensional Self-disgust scale.

**Results**

We found binge eating (BE) values similar to those from other national studies: mild to moderate BE (women: 6.3%; men: 5.0%) and severe BE (women: 3.3%; men: 0.8%). In men, BE total score positively correlated with defensive activation, cognitive-emotional and avoidance dimensions (self-disgust). Body mass index (BMI) positively correlated with BE total score and defensive activation (self-disgust) and negatively with family trauma. In women, BE total score positively associated with all self-disgust dimensions. Sexual trauma, family trauma, total of traumatic events and BMI positively associated with BE total score and all the self-disgust dimensions. In a hierarchical multiple regression analysis, BMI, total of traumatic events and the cognitive-emotional of self-disgust predicted BE total score. The cognitive-emotional (self-disgust) dimension mediated totally the relation between traumatic events and the BE total score.

**Conclusions**

In a sample from the general population and college students, BE values were similar to those from national studies. In women, sexual trauma, family trauma and total traumatic experiences (and all self-disgust dimensions) associated with BE. A higher BMI was associated with higher BE levels. In future interventions focusing on BE, in women, it seems important to consider the role of cognitive-emotional self-disgust in the relation between BE occurrence and distal traumatic events.

**Keywords**

Traumatic events, Self-disgust, Binge eating.

### O25 New paediatric screening procedures: health promotion in primary care

#### Marisa Lousada^1,2^, Ana P Mendes^3,4^, Helena Loureiro^1,5^, Graça Clemêncio^6^, Elsa Melo^1,5^, Ana RS Valente^1^

##### ^1^School of Health Sciences, University of Aveiro, 3810-193 Aveiro, Portugal; ^2^Center for Health Technology and Services Research, University of Aveiro, 3810-193 Aveiro, Portugal; ^3^Health Sciences School, Polytechnic Institute of Setúbal, 2914-503 Setúbal, Portugal; ^4^Centro Interdisciplinar de Investigação Aplicada em Saúde, Polytechnic Institute of Setúbal, 2914-503 Setúbal, Portugal; ^5^Health Sciences Research Unit: Nursing, Nursing School of Coimbra, 3046-851 Coimbra, Portugal; ^6^ACES Baixo Vouga, 3804-502 Aveiro, Portugal

###### **Correspondence:** Marisa Lousada (marisalousada@ua.pt)

**Background**

Screening procedures do not identify the specific disorder but allow a quick identification of children who may need a detailed assessment in speech therapy. Screening instruments are usually performed by different health professionals (e.g. pediatricians, nurses). The Child Health Program for primary care in Portugal determined that all 5-year-old children should be screened by nurses and general practitioners to conclude if they present a typical development suitable to school requirements. This screening is usually implemented through the Mary Sheridan test and there is no speech-language screening test used in primary care. Recently a Speech and Language Screening was validated for Portuguese children in kindergartens with excellent levels of specificity, sensitivity and reliability. RALF aims to quickly identify (5 minutes) children who may be at risk of speech-language impairment and need to be referred to a in depth assessment by a Speech-Language Therapist.

**Objectives**

This study aims to implement a new screening procedure in primary health care contributing to best practices. Specifically, the study aims to identify children with speech-language disorder that are undiagnosed due to the absence of a known condition such as neurological, hearing or cognitive impairment.

**Methods**

Ethical approval was granted by the Ethics Committee (UICISA) (ref.14/2016). A sociocultural questionnaire characterizing child and family background was fulfilled by caregivers to collect information about the child’s background (e.g., mother language; neurological, hearing, cognitive disorder) and child’s family background. Subject selection criteria included: Portuguese as native language and absence of a language disorder secondary to a known condition. The sample comprised 37 children whose parents returned informed consents. The screening was applied by 10 nurses in the Global Health Examination of 5 years old children in 2 health care centres.

**Results**

Twenty-one percent of children failed the screening. This illustrates the high level of speech-language difficulties (without any other associated condition) and is consistent with previous research studies. The children that failed the screening were already been referred to speech-language services for a detailed assessment.

**Conclusions**

This study highlights the importance of the implementation of a screening procedure in primary health care contributing to best practices.

**Acknowledgements**

Study supported by FEDER through POCI-01-0145-FEDER-007746 and FCT via CINTESIS, R&D Unit (ref. UID/IC/4255/2013).

**Keywords**

Screening, Speech and language, Health promotion.

### O26 Practices on using wearables during aquatic activities

#### Henrique P Neiva^1,2^, Luís Faíl^1^, Mário C Marques^1,2^, Maria H Gil^1,2^, Daniel A Marinho^1,2^

##### ^1^Department of Sport Sciences, University of Beira Interior, 6201-001 Covilhã, Portugal; ^2^Research Center in Sports Sciences, Health Sciences and Human Development, 6201-001 Covilhã, Portugal

###### **Correspondence:** Henrique P Neiva (henriquepn@gmail.com)

**Background**

Several studies described the use of different sensors to detect the daily activity, movement and sleep patterns and physical activities [1]. These are easily available for all those who are interested in tracking physical activity and progresses to improve physical fitness and health-related parameters [1]. However, little is known about the people’s knowledge about this equipment and specially in some specific activities that have some restrictions, for instance those performed in-water.

**Objective**

The purpose of this study was to characterize Portuguese practices on the use of wearable technology during aquatic activities.

**Methods**

Swimming pools from the interior region of Portugal were selected randomly and their users completed a questionnaire consisting of 33 questions. The first part focused on the characterization of their motivations and usual in-water activities, and the second focused on their views on the value of the wearable technology, its use and suggestions for future development of those devices according to aquatic activities.

**Results**

Ten swimming pools were accessed, and 418 questionnaires were filled by people ranging from 18 to 79 years-old. About 79% of these subjects have heard about wearables for sport, but 65% never used them during exercise. At the time of the inquiries, 24% still used and 11% gave up using it mainly because of lack of interest or because the devices did not work well underwater. Among the non-users, most reported that they did not have the opportunity (53%), considering that they are not useful (17%), or complaining about the financial cost (15%). However, most of them (74%) would be interested in trying this type of equipment during aquatic activities. Interestingly, 71% did not consider doing more exercise after they have the equipment. From those subjects using wearables, only a few (n = 24) used during in-water exercise.

**Conclusions**

For future, the devices should be more comfortable, be more reliable, be water resistant, with longer battery life. Besides the usual feedbacks provided, they also would like to see some technical corrections evidenced by that technology. People seemed to know about the existence of wearables to monitor physical activity but are still reluctant because of their underwater reliability, cost, and opportunity to try them. These results evidenced a need for improving these technological devices according to subjects needs and the activities performed. Some suggestions were made according to the future development of these devices to use during in-water exercitation.

**Acknowledgements**

NanoSTIMA: Macro-to-Nano Human Sensing Towards Integrated Multimodal Health Monitoring and Analytics, NORTE-01-0145-FEDER-000016, co-financed by FEDER-NORTE2020.

**References**

1. Chambers R, Gabbett TJ, Cole MH, Beard A. The use of wearable microsensors to quantify sport-specific movements. Sports Med. 2015, 45(7): 1065-1081.

**Keywords**

Technology, In-water activities, Sensors.

### O27 Does the recall of caregiver eating messages exacerbate the pathogenic impact of shame on eating and weight-related difficulties?

#### Sara Oliveira, Cláudia Ferreira

##### Cognitive and Behavioural Centre for Research and Intervention, University of Coimbra, 3000-115 Coimbra, Portugal

###### **Correspondence:** Sara Oliveira (sara.oliveira.uc@gmail.com)

**Background**

It has been recognized the central role of caregiver eating messages - restriction of food intake and pressures to eat - on later individual's eating behaviour, body image and weight status [1-3]. Additionally, shame is a painful emotion [4] also associated with the development and maintenance of body image and eating-related difficulties [5, 6], namely inflexible eating and concerns and maladaptive attitudes regarding body weight and shape [7].

**Objective**

The main aim of the present study was to test whether recalling caregiver eating messages [3] moderates the association of external shame [8] with inflexible eating rules [7] and with concerns and maladaptive attitudes regarding body weight and shape [9,10].

**Methods**

The sample comprised 479 Portuguese women, aged between 18 and 60 (M = 25.66; SD = 8.50), who completed validated self-report measures. The relationship between the study variables was accessed by Pearson product-moment correlation and the moderator effect was tested through path analysis.

**Results**

Results revealed that caregiver restrictive/critical messages played a significant moderator effect on the relationships of external shame with inflexible eating rules, and with concerns and maladaptive attitudes regarding body weight and shape. These findings suggested that caregiver restrictive/critical eating messages exacerbated the impact of shame on these psychopathological outcomes, with the tested model accounting for 17% and 29% of the variance of inflexible eating rules and body weight and shape concerns, respectively. In addition, pressure to eat caregiver messages was not correlated with all variables examined. A graphical representation of the moderation analyses allowed to understand that, for the same levels of external shame, women who recall more caregiver restrictive/critical eating messages tend to adopt more inflexible eating rules and present greater concerns and maladaptive attitudes regarding body weight and shape.

**Conclusions**

These findings appear to offer important clinical and investigational implications, highlighting the importance of the development of efficient parental intervention approaches as a refuge against maladaptive eating regulation strategies.

**References**

1. Abramovitz BA, Birch LL. Five-year-old girls’ ideas about dieting are predicted by their mothers’ dieting. J Am Diet Assoc. 2000; 100: 1157-1163. doi: 10.1016/S0002-8223(00)00339-4.

2. Birch LL, Fisher JO. Mother’s child-feeding practices influence daughters eating and weight. Am J Clin Nutr. 2000; 71: 1054-1061.

3. Kroon Van Diest A, Tylka T. The Caregiver Eating Messages Scale: Development and psychometric investigation. Body Image. 2010; 7:317-326. doi: 10.1016/j.bodyim.201006.002.

4. Gilbert P. What is shame? Some core issues and controversies. In: Gilbert P, Andrews B, editors. Shame: Interpersonal behavior, psychopathology and culture. New York: Oxford University Press; 1998. pp. 3- 38.

5. Goss K, Gilbert P. Eating disorders, shame and pride: A cognitive behavioural functional analysis. In: Gilbert P, Miles J, editors. Body shame: Conceptualization, research & treatment. Hove, UK: Brunner Routledge; 2002. pp. 219–255.

6. Hayaki J, Friedman M, Brownell K. Shame and severity of bulimic symptoms. Eat Behav. 2002; 3:73-83. doi:10.1016/S1471-0153(01)00046-0.

7. Duarte C, Ferreira C, Pinto-Gouveia J, Trindade I, Martinho A. What makes dietary restraint problematic? Development and validation of the Inflexible Eating Questionnaire. Appetite. 2017; 114:146-154. doi: 10.1016/j.appet.2017.03.034.

8. Matos M, Pinto-Gouveia J, Gilbert P, Duarte C, Figueiredo C. The Other As Shamer Scale – 2: Development and validation of a short version of a measure of external shame. Personal Individ Differ. 2015; 74:6-11. doi: 10.1016/j.paid.2014.09.037.

9. Fairburn CG, Beglin SJ. Assessment of eating disorders: interview of self report questionnaire? Int J Eat Disord. 1994; 16(4):363–370. doi:10.1002/1098-108X(199412).

10. Machado PP, Martins C, Vaz AR, Conceição E, Bastos AP, Gonçalves S. Eating Disorder Examination Questionnaire: psychometric properties and norms for the Portuguese population. Eur Eat Disord Rev. 2014; 22(6):448–453. doi:10.1002/erv.2318.

**Keywords**

Caregiver eating messages, External shame, Inflexible eating rules, Eating disordered, Women.

### O28 How does shame mediate the link between a secure attachment and negative body attitudes in men?

#### Sara Oliveira, Cláudia Ferreira

##### Cognitive and Behavioural Centre for Research and Intervention, University of Coimbra, 3000-115 Coimbra, Portugal

###### **Correspondence:** Sara Oliveira (sara.oliveira.uc@gmail.com)

**Background**

Shame is a painful self-conscious and universal emotion [1] regarded as a central feature of the development and maintenance of body image difficulties [2]. Additionally, it’s known the association between attachment style and body concerns, among women [3]. Particularly, a secure attachment may promote a more favourable body image [4]. However, few studies have focused on mechanisms that may explain body image difficulties in men.

**Objective**

The present study tested a model which hypothesized that the impact of a secure attachment on the engagement in negative male body attitudes, namely attitudes towards their muscularity and body fat [5, 6], is carried by general feelings of shame [7], while controlling the effect of body mass index.

**Methods**

The sample comprised 133 men, aged between 18 and 60 years old (M = 28.83; SD = 10.24), who completed validated self-report measures. The relationship between the study variables was accessed by Pearson product-moment correlation and the mediator effect was conducted through path analysis.

**Results**

The tested path model explained 22% and 49% of negative male attitudes towards their muscularity’s and low body fat’s variance, respectively. Results demonstrated that a secure attachment presented a significant direct effect on attitudes towards body fat, and an indirect effect through external shame on attitudes towards muscularity. In fact, these findings seem to suggest that men who were secure in attachment tend to experience less general feelings of shame and, consequently, presented low negative body attitudes, namely in regards to their muscularity and body fat.

**Conclusions**

These data support the relevance of addressing shame experiences when working with men with body image related-difficulties, especially in a context of early adverse experiences in their attachment.

**References**

1. Gilbert P. What is shame? Some core issues and controversies. In: Gilbert P, Andrews B, editors. Shame: Interpersonal problems, psychopathology and culture. New York: Oxford University Press; 1998. pp.3–3.

2. Goss K, Gilbert P. Eating disorders, shame and pride: A cognitive behavioural functional analysis. In Gilbert P, Miles J, editors. Body shame: Conceptualization, research & treatment. Hove, UK: Brunner Routledge; 2002. pp. 219–255.

3. Sharpe TM, Killen JD, Bryson SW, Shisslak CM, Estes LS, Gray N, et al. Attachment style and weight concerns in preadolescent and adolescent girls. Int J Eat Disord. 1998; 23(1):39-44.

4. Cash T. Cognitive-behavioral perspectives on body image. In: Cash T, Pruzinsky T, editors. Body image: A handbook of theory, research, and clinical practice. New York: The Guilford Press; 2002. pp.38-36.

5. Tylka TL, Bergeron D, Schwartz JP. Development and psychometric evaluation of the Male Body Attitudes Scale (MBAS). Body Image. 2005; 2(2):161-175. doi: 10.1016/j.bodyim.2005.03.001.

6. Ferreira C, Oliveira S, Marta-Simões J. Validation and psychometric properties of Portuguese Version of Male Body Attitudes Scale-Revised (MBAS-R). Manuscript in preparation, 2017.

7. Matos M, Pinto-Gouveia J, Gilbert P, Duarte C, Figueiredo C. The Other As Shamer Scale – 2: Development and validation of a short version of a measure of external shame. Personal Individ Differ. 2015; 74:6–11. doi:10.1016/j.paid.2014.09.03.

**Keywords**

Secure attachment, External shame, Negative body attitudes, Men.

### O29 Potential contamination of tourniquets used in peripheral venipuncture: preliminary results of a scoping review

#### Anabela S Oliveira^1^, Pedro Parreira^1^, Nádia Osório^2^, Paulo Costa^1^, Vânia Oliveira^1^, Fernando Gama^3^, João Graveto^1^

##### ^1^Health Sciences Research Unit: Nursing, Nursing School of Coimbra, Coimbra, 3046-851, Portugal; ^2^Coimbra Health School, Polytechnic Institute of Coimbra, Coimbra, 3046-854, Portugal; ^3^Coimbra Hospital and Universitary Centre, 3000-075 Coimbra, 3000-075, Portugal

###### **Correspondence:** João Graveto (jgraveto@esenfc.pt)

**Background**

Peripheral venipuncture constitutes one of the most frequent and invasive clinical procedures performed in healthcare settings [1-2]. In order to stop blood flow and promote vascular distension, the use of a tourniquet five to ten centimetres above the desired puncture site is recommended [3]. The irregular management of these specific medical devices, without complying with guidelines, constitutes a risk of microorganism dissemination [4-5].

**Objective**

To map the available evidence on the microbiological contamination of tourniquets used in peripheral venipuncture, identifying recurrent practices in their manipulation.

**Methods**

Scoping review based on the principles advocated by Joanna Briggs Institute [6]. The analysis of relevance of the articles, the extraction and synthesis of data was performed by two independent reviewers. The search strategy included all articles published until November 2017, written in Portuguese, Spanish, French and English.

**Results**

An initial total of 2,052 articles derived from the search conducted. Through Endnote software, 998 duplicates were removed. The remaining 1,054 articles were screened by title and abstract. Of these, 33 articles were included for full-text analysis by two independent reviewers. During this process, the reference lists of all included articles were screened, which resulted in the inclusion of 3 new articles. Ten studies were excluded due to absence of microbiological data inclusion and 6 were excluded due to lack of full-text access and author's reply. Overall, a total of 1,337 tourniquets belonging to nurses, nursing assistants, doctors, phlebotomists and lab workers were analysed for microorganism contamination. A small number of studies verified that the same tourniquets were used continuously by professionals between 3 days to 104 weeks. Preliminary results evidenced contamination rates varying between 9% and 100%, composed by diverse microorganisms such as *Staphylococcus aureus*, *Escherichia coli*, *Pseudomonas aeruginosa*, *Enterococcus* and *Acinteobacter baumannii*. Several of the included studies described conflicting practices during tourniquet manipulation by health professionals, especially when focused on domains such as hand hygiene before and after tourniquet use, glove usage during venipuncture, tourniquet cleaning and disinfecting, sharing tourniquets with other professionals and storage conditions. The most cited reason for tourniquet replacement in clinical settings was due to their loss by health professionals.

**Conclusions**

As a contribution to clinical practice, it is expected that the mapping of the available scientific evidence regarding the potential contamination of these devices will appear as an informative contribution that supports the analysis of current practices in this field, promoting the implementation of quality assurance systems in health institutions.

**Acknowledgements**

This protocol is part of the project “Transfer of technological innovations to nursing practice: a contribution to the prevention of infections”, funded from the European Regional Development Fund, by the Operational Program Competitiveness and Internationalization of PORTUGAL 2020.

**References**

1. Marsh N, Webster J, Mihala G, Rickard C. Devices and dressings to secure peripheral venous catheters: A Cochrane systematic review and meta-analysis. International Journal of Nursing Studies. 2017;67:12-19.

2. Oliveira AS. Intervenção nas práticas dos enfermeiros na prevenção de flebites em pessoas portadoras de cateteres venosos periféricos: um estudo de investigação-ação [PhD thesis]. Universidade de Lisboa; 2014.

3. Veigar B, Henriques E, Barata F, Santos F, Santos I, Martins M et al. Manual de Normas de Enfermagem: Procedimentos Técnicos. 2nd ed. Administração Central do Sistema de Saúde, IP; 2011.

4. World Health Organization. Decontamination and reprocessing of medical devices for healthcare facilities. Geneva, Switzerland: WHO Document Production Services; 2016.

5. Costa P. Gestão de material clínico de bolso por enfermeiros: fatores determinantes e avaliação microbiológica [Masters dissertation]. Nursing School of Coimbra; 2017.

6. Peters M, Godfrey C, McInerney P, Baldini Soares C, Khalil H, Parker D. Chapter 11: Scoping Reviews. In: Aromataris E, Munn Z, ed. by. Joanna Briggs Institute Reviewer's Manual [Internet]. The Joanna Briggs Institute; 2017 [cited 14 December 2017]. Available from: https://reviewersmanual.joannabriggs.org/.

**Keywords**

Tourniquets, Contamination, Peripheral venipuncture.

### O30 Effects of a community-based food education program on nutrition-related knowledge in middle-aged and older patients with type 2 diabetes: a RCT

#### Carlos Vasconcelos^1,2^, António Almeida^1^, Maria Cabral^3^, Elisabete Ramos^3,4^, Romeu Mendes^1,3,5^

##### ^1^University of Trás-os-Montes e Alto Douro, 5000-801 Vila Real, Portugal; ^2^Polytechnic Institute of Viseu, 3504-510 Viseu, Portugal; ^3^Instituto de Saúde Pública, Universidade do Porto, 4050-600 Porto, Portugal; ^4^Faculty of Medicine, University of Porto, 4200-319 Porto, Portugal; ^5^Public Health Unit, ACES Douro I – Marão e Douro Norte, 5000-524 Vila Real, Portugal

###### **Correspondence:** Romeu Mendes (romeuduartemendes@gmail.com)

**Background**

Peripheral Diabetes imposes an unacceptably high human, social and economic cost, especially on aging populations. Nutrition-related knowledge is of crucial importance to make healthier food choices, contributing for type 2 diabetes (T2D) control and related comorbidities prevention.

**Objective**

To analyse the effects of a food education program (FEP) on the nutrition-related knowledge (NRK) in middle-aged and older patients with T2D.

**Methods**

Forty-two individuals between 50 and 80 years old with T2D were recruited in primary health care institutions, to participate in *Diabetes em Movimento*®, a community-based exercise program (3 exercise sessions per week; 75 minutes each; during 9 months), developed in Vila Real, Portugal. Participants were randomized into two groups: a control group (CG; N = 19; exercise program only) and an experimental group (EG; N = 23; exercise program plus a FEP). The FEP was 16 weeks long and, on each week, a different nutrition-related theme was addressed. Each theme was driven through a theoretical session (15 minutes) and dual-task exercise strategies integrated in *Diabetes em Movimento*®'s sessions. The NRK was evaluated, before and after the 9-month intervention, using the Portuguese reduced version of Nutritional Knowledge Questionnaire (from 0 to 56 points; higher score, better knowledge).

**Results**

Thirty-six participants completed the study (CG, N = 16; EG, N = 20). The baseline score was 30.19 ± 6.10 (CG) vs. 29.40 ± 6.16 points (EG). After the intervention, the score was 31.31 ± 7.40 (CG) vs. 35.20 ± 5.68 points (EG). A significant time*group interaction effect was identified (p = 0.001; η2p = 0.290). Considering the FEP's sessions adherence level (< 50% vs. ≥ 50%), a significant time*group interaction effect was also identified (baseline, 29.78 ± 7.84 [< 50%] vs. 29.09 ± 4.76 [≥ 50 %]; after intervention, 32.78 ± 5.93 [50 %] vs. 37.18 ± 4.85 [≥ 50%]; p = 0.004, η2p = 0.370).

**Conclusions**

A community-based easy-to-implement food education program was effective in increasing NRK of middle-aged and older patients with type 2 diabetes and may contribute to better food choices. Program's adherence levels play a major role on knowledge acquisition.

**Trial Registration**

NCT02631902

**Keywords**

Type 2 diabetes; Food education program; Nutrition-related knowledge; Community-based intervention.

### O31 Perception of oral antidiabetic agents adverse events and their impact on Health Related Quality of Life in type 2 diabetic patients

#### Rui S Cruz^1^, Luiz M Santiago^2^, Carlos F Ribeiro^3^

##### ^1^Coimbra Health School, Polytechnic Institute of Coimbra, 3046-854 Coimbra, Portugal; ^2^Faculty of Medicine, University of Coimbra, 3004-504 Coimbra, Portugal; ^3^Department of Pharmacology and Experimental Therapeutics, Faculty of Medicine, University of Coimbra, 3004-504 Coimbra, Portugal

###### **Correspondence:** Rui S Cruz (ruic@estescoimbra.pt)

**Background**

Currently, drug therapy with oral antidiabetic agents, is capable of inducing normoglycemia levels able to decrease the risk of complications associated with diabetes mellitus. However, it is also known that the various existing oral antidiabetic agents may trigger a large number of adverse events, either alone or in combination. Some of these tolerability and security issues related to the oral antidiabetic are reported by patients and can influence negatively or satisfaction with treatment or glycaemic control, or the therapeutic adherence and maintenance. It is therefore very important the role of patients in monitoring adverse events related to the use of the oral antidiabetic drugs in order to optimize treatment and improve the quality of life of patients with type 2 diabetes (DM2).

**Objective**

The aim of this study was to determine the prevalence of adverse events associated with use of oral antidiabetics and assessing their impact on Health-related Quality of Life (HRQoL) of diabetic patients tracked in primary health care.

**Methods**

A total of 357 DM2 patients were enrolled in observational and cross-sectional study, recruited in six Health Care Centres/Family Health Units (FHU) of the central region of Portugal. Data collection comprised three questionnaires to measure the prevalence of adverse events, the diabetes health profile (DHP-18) and EQ-5D-3L.

**Results**

Results showed that the highest prevalence of adverse events is in the DipeptidylPeptidase-4 Inhibitors followed by Metformin+Sitagliptin (fixed dose) and Metformin+Vildagliptin (fixed dose) therapeutic classes. We also found that all correlations between different variables were statistically significant (p < 0.001).

**Conclusions**

Thus, we conclude that patients who show a greater number of adverse events tend to have poorer health profiles, worse general health and also lower health-related quality of life.

**Keywords**

Diabetes Medication, Therapy, Quality of Life.

### O32 Third stage of waterbirth: observational study

#### Joyce CS Camargo^1,2^, Vitor Varela^3^, Elisabete Santos^3^, Natalucia M Araújo^2^, Kelly CMP Venâncio^4^, Manuela Néné^5^, Maria CLR Grande^6^

##### ^1^Abel Salazar Institute of Biomedical Sciences of the University of Porto, 4200-135 Porto, Portugal; ^2^School of Arts, Sciences and Humanities, University of São Paulo, 03828-000 São Paulo, Brazil; ^3^São Bernardo Hospital, 2910-445 Setúbal, Portugal; ^4^College of Nursing, University of São Paulo, 05403-000 São Paulo, Brazil; ^5^Escola Superior de Saúde da Cruz Vermelha Portuguesa, 1350-125 Lisboa, Portugal; ^6^Faculty of Psychology and Educational Sciences, University of Porto, 4200-135 Porto, Portugal

###### **Correspondence:** Joyce CS Camargo (joyce@usp.br)

**Background**

The placental-delivery, in waterbirth (WB), usually occurs while maternal wellbeing is monitored through clinical aspects, heart-rate and blood-pressure, as well as water-coloration. Adequate care should be taken in this period, with prevention of postpartum-haemorrhage(HPP), which is the main cause of maternal death in developing countries, and approximately ¼ of all maternal deaths worldwide [1].

**Objective**

To verify the outcome of the labour’s third stage at the Waterbirth-Project (PWB), in Setúbal, at São Bernardo’s Hospital located in Portugal. Study's question: *What’s the maternal outcome of childbirth’s 3rd stage in PWB?*

**Methods**

Observational-study, cross-sectional, descriptive based on ethical guidelines (CNPD-9885/2015) approved by the hospital, where delivery room’s infrastructure, protocol’s definition and technical and scientific training of the obstetric-team began in 2006. The PWB occurred between 2011-2014. 153 women, with a single pregnancy, gestational age ≥ 37 weeks with low-risk prenatal care participated in the PWB, signed an informed consent form about study’s benefits and risks, resulting in 90 waterbirths. Data were collected from the specific PWB forms in April 2016. Data management was developed in Excel® and SPSS® version 16.0 programs.

**Results**

In the PWB, 51.1% of women had placental waterbirth *vs* 48.9% out-of-water. The active management occurred in 7.7% vs 86.7% of physiological management, 92.3% of the women had physiological blood loss, and 7.7% had increased bleeding, controlled with uterotonics. These results corroborate with evidence: Swiss study [2], with 89 WB *vs* 279 out-of-water births, 57% had physiological defect in the water and the 3rd-Stage was significant (p < 0.01) in PA; English study [3] with 5,192 WB, with third physiological stage, 86.1% of which 55.8% were in water. For 3rd-Stage’s management [4]: 1. Active: administration of uterotonic (oxytocin [1], 1st choice) after birth, timely clamping of the umbilical cord and controlled cord traction. 2. Physiological: Spontaneous relief assisted by gravity and/or maternal effort. In order to evaluate PPH in WB, in addition to the clinical state of the puerperium, the coloration of the comparison to wine is analysed: 50-100 ml blood loss to the pink-Chablis; from 150-250 ml to bleed and 500-750 ml to Merlot-wine [5] or, study-site midwife’s puts-hand below the surface of the water horizontally. If visible hand, haemorrhage ≤ 500mL, if hand not-visible, haemorrhage > 500mL.

**Conclusions**

The 3rd-Stage’s management in WB is safe and according to the experience of the PWB, no adverse events were related to it. More studies are needed to support good clinical practices based on scientific evidence.

**References**

1. OMS. Recomendações da OMS para a prevenção e tratamento da hemorragia pós-parto. In: Saúde OMd, editor. 2014. p. 48.

2. Zanetti-Dallenbach RA, Lapaire O, Maertens A, Holzgreve W, Hosli I. Water birth, more than a trendy alternative: a prospective, observational study. Arch Gynecol Obstet. 2006;274(6):355-65.

3. Burns EE, Boulton MG, Cluett E, Cornelius VR, Smith LA. Characteristics, interventions, and outcomes of women who used a birthing pool: A prospective observational study. Birth. 2012;39(3):192-202.

4. ICM, FIGO. Prevention and Treatment of Post-partum Haemorrhage: New Advances for Low Resource Settings International Confederation of Midwives (ICM). International Federation of Gynaecology and Obstetrics (FIGO); 2006.

5. Harper B. Gentle Birth Choices. Revised Edition ©2005 Barbara Harper ed: Inner Traditions Bear and Company; 2005 August 09, 2005.

**Keywords**

Childbirth’s 3rd stage, Placental-delivery, Postpartum-haemorrhage, Waterbirth, Midwifery.

### O33 Aqua apgar in waterbirth: cross-sectional study

#### Joyce CS Camargo^1,2^, Vitor Varela^3^, Elisabete Santos^3^, Maria AJ Belli^2^, Maryam MJ Trintinália^2^, Manuela Néné^4^, Maria CLR Grande^5^

##### ^1^Abel Salazar Institute of Biomedical Sciences of the University of Porto, Portugal; ^2^School of Arts, Sciences and Humanities, University of São Paulo, 03828-000 São Paulo, Brazil; ^3^São Bernardo Hospital, 2910-445 Setúbal, Portugal; ^4^Escola Superior de Saúde da Cruz Vermelha Portuguesa, 1350-125 Lisboa, Portugal; ^5^Faculty of Psychology and Educational Sciences, University of Porto, 4200-135 Porto, Portugal

###### **Correspondence:** Joyce CS Camargo (joyce@usp.br)

**Background**

Waterbirth (WB) is the complete underwater fetal expulsion [1,2] with much discussion [3] about it. Aqua Apgar [4] is an index that evaluates the newborn’s vitality (NB) while still submerged in water until life’s first minute, developed by Cornelia Enning.

**Objective**

To know the neonatal outcome of the Waterbirth Project (PWB) at a Setubal’s (Portugal) Hospital, São Bernardo. Study’s Question: *What is the neonatal outcome of new-borns born in PWB?*

**Methods**

Cross-sectional study, observational, descriptive based on ethical guidelines (CNPD-9885/2015) approved by the hospital, whose delivery-room’s infrastructure, protocol’s definition and obstetric-team’s technical and scientific training began in 2006. The PWB occurred between 2011-2014. 153 women with single pregnancy, gestational age ≥ 37 weeks with low-risk prenatal care participated in the PWB and signed an informed consent form about study’s benefits and risks, resulting in 90 waterbirth. Data were collected from the specific PWB form in April 2016. Data management was developed in Excel® and SPSS® version 16.0 programs.

**Results**

The 1st minute’s Aqua Apgar and the 5th minute’s Apgar were superior to 7 in all the cases, with an average of 9.4 at the 1st minute and 9.9 at the 5th minute. A cross-sectional-study in Sydney [5] observed minor Apgar and may be due to disregarding that water-born NB manifest their vitality by moving the legs and arms, opening and closing their eyes and mouth and swallowing [4]. A cohort study in the UK [6] corroborates our study that NB of aquatic birth were less likely to have a low Apgar score in the 5th minute. The use of Aqua Apgar in our study allowed a coherent outcome in the NBs who is kept submerged in water until life’s first minute, with a soft transition to extra uterine life and with no negative repercussions on heart rate and absence of complication or neonatal hospitalization.

**Conclusions**

This study provides evidences that may support clinical decisions regarding delivery in water. Further studies on Aqua Apgar should be conducted to support evidence-based practices.

**References**

1. Nutter, E., Meyer, S., Shaw-Batista, J. & Marowitz, A. (2014). Waterbirth: an integrative analysis of peer – reviewed literature. Journal of Midwifery & Women’s Health. 59, (3), 286-319.

2. Cluett ER, Burns E. Immersion in water in labour and birth. Cochrane Database Syst Rev 2009;(2):CD000111.

3. ACOG. American College of Obstetricians & Gynecologists. (2014). Immersion in water during labor and delivery (Committee Opinion No. 594). Retrieved from http://www.acog.org/ Resources_And_Publications/Committee_Opinions/ Committee_on_Obstetric_Practice/Immersion_in_ ater_During_Labor_and_Delivery 4.Garland D. Revisiting Waternirth: an attitude to care. 2011. Published by Palgrave Macmillan. ISBN 10: 0230273572 / ISBN 13: 978023273573

5. Bovbjerg M L; Cheyney M; Everson C. (2016). Maternal and Newborn Outcomes Following Waterbirth: The Midwives Alliance of North America Statistics Project, 2004 to 2009 Cohort. J Midwifery Womens Health. Jan-Feb;61(1):11-20. doi: 10.1111/jmwh.12394. Epub 2016 Jan 20.

6. Dahlen HG, Dowling H, Tracy M, Schmied V, Tracy S. (2013). Maternal and perinatal outcomes amongst low risk women giving birth in water compared to six birth positions on land. A descriptive cross sectional study in a birth centre over 12 years. Midwifery;29(7):759-64.

**Keywords**

Aqua Apgar, Waterbirth, Midwifery, Apgar, Childbirth.

### O34 Portuguese centenarians from Oporto and Beira Interior: distinctive health profiles?

#### Daniela Brandão^1,2^, Oscar Ribeiro^1,3^, Rosa M Afonso^1,4^, Constança Paúl^1,5^

##### ^1^Center for Health Technology and Services Research, 4200-450 Porto, Portugal^; 2^Faculty of Medicine, University of Porto, 4200-319 Porto, Portugal^; 3^University of Aveiro, 3810-193 Aveiro, Portugal^; 4^University of Beira Interior, 6201-001 Covilhã, Portugal^; 5^Institute of Biomedical Sciences Abel Salazar, University of Porto, 4050-313 Porto, Portugal

###### **Correspondence:** Daniela Brandão (danielafsbrandao@gmail.com)

**Background**

In Portugal, the number of centenarians almost tripled over the last decade from 589 centenarians in 2001 to 1526 in 2011 [1], and recent projections point to the existence of 3,393 centenarians in 2013 [2]. Reaching the age of 100, though an important landmark, does not necessarily indicates successful aging as it is often accompanied by severe health and functional constraints. Understanding health trajectories of these long-lived individuals and studying the prevalence of diseases that are the most common causes of death is important for conveniently addressing their current caregiving needs.

**Objective**

The aim of this study is to present an overview of the sociodemographic and health-related characteristics of two distinct samples of Portuguese centenarians (predominantly rural *vs*. predominantly urban) and acknowledge potential dissimilarities.

**Methods**

A sample of 241 centenarians was considered (140 from the PT100 Oporto Centenarian Study and 101 from the PT100 Beira Interior Centenarian Study). Sociodemographic information, nature and number of diseases, functionality and physical health variables were collected.

**Results**

In both samples, most centenarians were female (89.3% in Oporto, and 86.1% in Beira Interior), and widowed (76.4% in Oporto, 91.1% in Beira Interior), and lived in the community (57.9% in Oporto, 49.0% in Beira Interior). Higher levels of basic activities of daily living (BADL) and instrumental activities of daily living (IADL) dependency were found in the Oporto sample, as well as a higher percentage of bedridden centenarians (61.0% in Oporto *vs*. 38.1% in Beira Interior). Sensorial impairments and incontinence were the most frequent conditions reported in both samples; however, lower percentages of age-related illnesses were found in the Beira Interior sample. Considering the three most lethal diseases among the elderly population (heart disease, non-skin cancer and stroke), 60.0% of centenarians in Oporto escaped these conditions, whereas in Beira Interior this percentage increases to 85.4%.

**Conclusions**

This study provides a general overview about the health profile of Portuguese centenarians in two types of communities: one rural and with low population density, and another in an urban context. Our findings raise important differences between centenarians from the two samples, which reinforce the heterogeneity of this population, and the importance of environmental factors in how such an advanced age was achieved. Findings highlight the need for potentially distinctive health promotion initiatives in these two settings.

**Acknowledgements**

This work was supported by the Portuguese Foundation for Science and Technology (FCT) [PhD Grant for the first author - SFRH/BD/101595/2014]. The PT100 Oporto Centenarian Study was supported by the Portuguese Foundation for Science and Technology (FCT; Grant Pest – C/SAU/UI0688/2011 and C/SAU/UI0688/2014).

**References**

1. National Statistical Institute of Portugal, (INE). Censos - Resultados definitivos. Região Norte – 2011. Lisboa: Instituto Nacional de Estatística; 2012.

2. National Statistical Institute of Portugal (INE). Projeções de População Residente 2015–2080. Lisboa: Instituto Nacional de Estatística; 2017.

**Keywords**

Centenarians, Health, Functionality, Diseases, Portugal, Morbidity.

### O35 Implementation of an educational program to promote functionality in medical wards: quasi-experimental study

#### João Tavares^1,2^, Joana Grácio^3^, Lisa Nunes^3^

##### ^1^Nursing School of Coimbra, Coimbra, 3046-851, Portugal; ^2^Coimbra Education School, Polytechnic Institute of Coimbra, 3030-329, Portugal; ^3^Coimbra Hospital and Universitary Centre, 3000-075 Coimbra, 3000-075, Portugal

###### **Correspondence:** João Tavares (enf.joaotavares@hotmail.com)

**Background**

Functional decline, diminished performance in at least one activity of daily living, is often of 30 to 60% among hospitalized older adults (OA) [1]. Quality nursing care is essential to prevent functional decline. A “new” theoretically based philosophy of care has been proposed: the Function Focused Care (FFC), which is geared toward optimization of function and physical activity during all personal/care related activities that occur throughout the hospital stay [2]. The FFC has demonstrated better outcomes at discharge and post-acute periods.

**Objective**

To evaluate the effect of an educational program for nurses in promoting the FFC among hospitalized OA.

**Methods**

This is a prospective quasi-experimental study developed in four internal medical units. These units were randomly selected in two units for case (intervention) and two for control. Participants were 117 OA and 94 registered nurses (RN). Intervention consisted in the development and implementation of an educational program about FFC to RN, lasting 10 hours, and a maintenance program, during 5 months. Further details about the program can be found in Tavares et al [3]. Implementation of FFC activities by RN was assessed through the FFC Behaviour Checklist, which was completed by the researchers through non-participant observation [4]. The measures for patients were the functional decline (DF) assessed by the Katz Index: difference between baseline and discharge (t0), discharge and follow-up of 3 months (t1) and baseline and follow-up (t2). For comparison of the case and control groups, an independent t-test was calculated.

**Results**

The patient’s sociodemographic and clinical characteristics showed no statistical differences between groups. The provision of FFC mean was 0.46 ± 0.22, indicating that RN promoted only 46% of total possible FFC activities. Significant statistical differences were found between case and control group (*t*(91)= -2.85; p= 0.01), with means of 0.52 ± 0.24 and 0.39 ± 0.19, respectively. No statistical difference was found between the promotion of FFC and the functional decline at t0 (U = 30.5, p = 0.15), t1 (*t*(38.82) = 6.293; p< 0.15) or t2 (*t*(83) = 2.49, p = 0.44).

**Conclusions**

Promotion of functionality is very low, which could be explained by the lack of impact in FD prevention. However, in the case group, more FFC activities were developed. These results suggest a positive impact of the educational program in OA care. The FFC can be seen as a challenge and opportunity for change, innovation, and creativity, in order to improve the effectiveness, efficiency, and quality of care of hospitalized OA.

**References**

1. Hoogerduijn JG, Schuurmans MJ, Duijnstee MSH, De Rooij SE, Grypdonck MFH. A systematic review of predictors and screening instruments to identify older hospitalized patients at risk for functional decline. J Clin Nurs. 2007;16(1):46-57.

2. Burket TL, Hippensteel D, Penrod J, Resnick B. Pilot testing of the function focused care intervention on an acute care trauma unit. Geriatr Nurs. 2013;34(3):241-246.

3. Grácio J, Tavares JP de A, Nunes L, Silva R. Programa educacional para enfermeiros: eficácia de duas estratégias formativas. In: XI Congresso Internacional Galego-Português de Psicopedagogia, 2017; Braga: Universidade do Minho. Instituto de Educação. Centro de Investigação em Educação Universidade Minho, 2017; 540-541.

4. Tavares JP de A, Grácio J, Nunes L. Functional Focused care: content validity of Functional Focused Care Behavior Checklist. Eur Geriatr Med. 2016;7(supplement 1):S1-S282.

**Keywords**

Function focused care, Older adults, Hospitalization functionality, Educational program.

### O36 Self-reported data and its relation to the standard and validated measures to predict falls

#### Anabela C Martins, Catarina Silva, Juliana Moreira, Nuno Tavares

##### Physiotherapy Department, Coimbra Health School, Polytechnic Institute of Coimbra, 3026-854 Coimbra, Portugal

###### **Correspondence:** Anabela C Martins (anabelamartins@estescoimbra.pt)

**Background**

According to National Institute for Health and Care Excellence quality standards, the assessment of fall risk and preventing falls should be multifactorial and include self-reported questions like fall history, fear of falling (FoF), self-perception of functional ability, environment hazards, gait pattern, balance, mobility and muscle strength [1]. Concerning the self-reported data, some studies described subjectivity and difficulty in extracting reliable information when using such methods. History and number of previous falls are often used as golden standard in fall risk assessment studies [2]; however, these questions are source of misjudgement, in part, due to difficulty for an older person remember exactly how many times he/she had fallen in a past period of time.

**Objective**

The study aimed to compare self-reported questions and standard and validated measures for screening risk of fall to verify the confidence of the self-reported data.

**Methods**

506 community-dwelling adults aged 50+ years old (mean age 69.56 ± 10.29 years old; 71.7% female) were surveyed regarding demographics, history of fall, FoF, sedentary lifestyle, use of upper-extremities to stand up from a chair, by self-reported questionnaire; analysis of gait, balance and muscle strength, by standard and validated measures for screening risk of fall - 10 meters walking speed test [3], Timed Up & Go test [4] and 30 second sit to stand test [4], respectively. Independent samples t tests were performed to compare groups.

**Results**

33.2% of the sample reported at least one fall in the last year (fallers), 50% reported FoF, 46.4% sedentary lifestyle, 31.8% needed their upper extremities assistance to stand from a chair. Fallers demonstrated lower scores of gait velocity (p < 0.001), lower extremities strength (p < 0.001) and balance (p = 0.034) compared with non-fallers; who reported sedentary lifestyle also showed lower scores of gait velocity (p < 0.001), lower extremities strength (p = 0.001) and balance (p < 0.001) compared with non-sedentary. Simultaneously, who assumed FoF showed lower scores of gait velocity (p < 0.001), lower extremities strength (p < 0.001) and balance (p < 0.001) compared with who had no FoF. Finally, those who use the upper-extremities to stand up from a chair showed lower scores of gait velocity (p < 0.001), lower extremities strength (p < 0.001) and balance (p < 0.001) compared with those who do not.

**Conclusions**

The findings suggest that self-reported data like history of falls, sedentary lifestyle, FoF and use of upper extremities to stand up from a chair, obtained by simple questions, have emerged as reliable information on risk factors for falling and can be used to complete the fall risk screening.

**Acknowledgements**

Authors would like to thank all participants and centres, clinics and other entities hosting the screenings. Financial support from project FallSensing: Technological solution for fall risk screening and falls prevention (POCI-01-0247-FEDER-003464), co-funded by Portugal 2020, framed under the COMPETE 2020 (Operational Programme Competitiveness and Internationalization) and European Regional Development Fund (ERDF) from European Union (EU).

**References**

1. NICE, Nacional Institute for Health and Care Excellence. Falls in older people: assessing risk and prevention. Clinical Guideline, 2013 Available at: nice.org.uk/guidance/cg161 (faltam dados à referência)

2. Garcia AG, Dias JMDD, Silva SLA, Dias RC. Prospective monitoring and self-report of previous falls among older women at high risk of falls and fractures: a study of comparison and agreement. Braz J Phys Ther. 2015; 19(3).

3. Fritz S & Lusardi M. White paper: “walking speed: the sixth vital sign”. J Geriatr Phys Ther. 2009; 32(2): 2-5.

4. Stevens JA. The STEADI tool kit: a fall prevention resource for health care providers. IHS Prim Care Provid. 2016, 39: 162-6.

**Keywords**

Self-reported data, Fall Risk Assessment, Community dwelling adults.

### O37 Life after falling: which factors better explain participation in community dwelling adults?

#### Juliana Moreira, Catarina Silva, Anabela C Martins

##### Physiotherapy Department, Coimbra Health School, Polytechnic Institute of Coimbra, 3026-854 Coimbra, Portugal

###### **Correspondence:** Juliana Moreira (juliana.moreira@estescoimbra.pt)

**Background**

Participation is defined by World Health Organization (WHO), as the person’s involvement in a life situation [1]. There are few studies exploring the association between participation restriction and being older, exhibiting more depressive moods, poor mobility, and a lack of balance confidence [2,3].

**Objective**

The objective of this study was to identify which factors, namely, age, functional capacity and self-efficacy for exercise have the best association with participation.

**Methods**

A sample of 168 community-dwelling adults (age ≥50 years), mean age 70.45 ± 10.40 years old (78.6% female), with history of at least one fall in the previous year, participated in the study. Measures included demographic variables, functional capacity, assessed by six functional tests: Grip strength, Timed Up and Go (TUG), 30 seconds Sit-to-Stand, Step test, 4 Stage Balance “modified” and 10 meters Walking Speed and two questionnaires (Self-efficacy for exercise and Activities and Participation Profile related to Mobility - PAPM). Descriptive and correlational statistics were performed to analyse data.

**Results**

Fifty-nine percent of participants presented restrictions in participation (34.8% mild restrictions, 17.4% moderate restrictions and 6.8% severe restrictions). Participation showed a strong correlation with 10 meters walking speed (r = -0.572) and TUG (r = 0.620) for a significance level p < 0.001. A moderate correlation was found between participation and 30 seconds Sit-to-Stand (r = -0.478), Step test (r = -0.436), Grip strength (r = -0.397), 4 Stage Balance test “modified” (r = -0.334), as well as, Self-efficacy for exercise (r = -0.401) and age (r = 0.330), for a significance level p < 0.001.

**Conclusions**

This study suggests that participation of individuals with history of fall is associated with functional capacity, self-efficacy for exercise and age. Previous studies have showed comparable findings [4,5,6], however, admitting the strong association between participation and 10 meters Walking Speed and TUG, it is essential to include these instruments in a comprehensive evaluation of the individuals who have suffered a fall in the past year to predict participation restrictions. The performance assessed, in few minutes, by these tests, will gather information about balance and mobility impairments, that associated with a quick assess of Self-efficacy for exercise [7] will outline the quality of life of persons with history of falls.

**Acknowledgements**

Authors would like to thank all participants and centres, clinics and other entities hosting the screenings. Financial support from project FallSensing: Technological solution for fall risk screening and falls prevention (POCI-01-0247-FEDER-003464), co-funded by Portugal 2020, framed under the COMPETE 2020 (Operational Programme Competitiveness and Internationalization) and European Regional Development Fund (ERDF) from European Union (EU).

**References**

1. WHO, World Health Organization. International Classification of Functioning, Disability, and Health. Geneva: Classification, Assessment, Surveys and Terminology Team, 2001

2. Liu J. The severity and associated factors of participation restriction among community dwelling frail older people: an application of the International Classification of Functioning, Disability and Health (WHO-ICF). BMC Geriatrics, 2017, 17:43.

3. Desrosiers J, Robichaud L , Demers L, Ge’linas I, Noreau L, Durand D. Comparison and correlates of participation in older adults without disabilities. Archives of Gerontology and Geriatrics, 2009, 49: 397–403.

4. Anaby D, Miller WC, Eng JJ, Jarus T, Noreau L, Group PR. Can personal and environmental factors explain participation of older adults? Disability and Rehabilitation, 2009;31(15):1275–82.

5. Rubio E, Lázaro A, Sánchez-Sánchez A. Social participation and independence in activities of daily living: across sectional study. BMC Geriatrics, 2009, 9:26.

6. Tomioka K, Kurumatani N, Hosoi H. Social Participation and the Prevention of Decline in Effectance among Community-Dwelling Elderly: A Population-Based Cohort Study. PLoS ONE, 2015, 10(9).

7. Martins AC, Silva C, Moreira J, Rocha C, Gonçalves A. Escala de Autoeficácia para o Exercício: validação para a população portuguesa. Conversas de Psicologia e do Envelhecimento Ativo, 2017, 126-141

**Keywords**

Participation, Community-dwelling adults, Falls, Functional capacity, Self-efficacy for exercise.

### O38 History of fall and social participation profile among community dwelling older adults: is there any relation with frailty phenotype?

#### Mónica Calha, Anabela C Martins

##### Physiotherapy Department, Coimbra Health School, Polytechnic Institute of Coimbra, 3026-854 Coimbra, Portugal

###### **Correspondence:** Mónica Calha (monicacalha@gmail.com)

**Background**

Ageing population is a worldwide phenomenon. The number of older frail people increases rapidly, which leads to a substantial impact on the economic, social and health systems. Cardiovascular Health Study data [1] estimated that, in a population with 65 years or more, 6.3% of aged adults have the frailty phenotype. According to Fried *et al.*, frailty is a vulnerable condition characterized by the decline of biological reserves [2,3]. This happens due to deregulation of multiple physiological systems, which puts the individuals at risk by reducing the organism resistance to stressful factors, with a subsequent loss of functional homeostasis. One of the most significant aspects described in the literature is the fact that frailty is an important risk factor for falls. It is estimated that one in every three adults over 65 years fall each year. The frailty syndrome also compromises the social participation of aged adults.

**Objective**

To understand if adults with 65 years or over with frailty phenotype have history of falls in the period of the previous 12 months prior to the study and worst social participation, when compared to the ones who don't have this phenotype.

**Methods**

A sample of 122 community-dwelling adults (age ≥65 years), mean age 72.22 ± 6.44 years old (63.9% female), with history of at least one fall in the previous year participated in this cross-sectional study. Data were collected by a demographic, clinical and history of falls questionnaire, functional tests and the Activities and Participation Profile related to Mobility (PAPM).

**Results**

We verified that there are statistically significant differences in the history of falls between no-frailty (n = 24; mean number of falls = 1.92) and frailty/pre-frailty (n = 31; mean number of falls = 3.06) individuals (p = 0.036), as well as in the social participation score of both groups, with worse profile among the frailty/pre-frailty (0.821), when compared to no-frailty (0.276) (p = 0.000).

**Conclusions**

Adults with 65 years or over who present frailty or pre-frailty phenotype, when compared to no-frailty ones, have higher rate of falls in the previous 12 months and more restrictions in social participation. Physiotherapists benefit from this knowledge to understand needs of this population and to plan interventions focus on prevention of falls and strategies to promote participation as promising outcomes.

**References**

1 Etman, A., Burdorf, A., Van der Cammen, T.J.M., Mackenbach, J.P., Van Lenthe, F.J. (2012). Socio-demographic determinants of worsening in frailty among community-dwelling older people in 11 European countries. Journal of Epidemiology and Community Health; 66(12):1116-1121.

2 Eyigor, S., Kutsal, Y.G., Duran, E., et al. (2015). Frailty prevalence and related factors in the older adult - FrailTURK Project. American Aging Association; 37(3):1-13.

3 Tarazona-Santabalbina, F.J., Gómez-Cabrera, M.C., Pérez-Ros, P., et al. (2016). A Multicomponent Exercise Intervention that Reverses Frailty and Improves Cognition, Emotion, and Social Networking in the Community-Dwelling Frail Elderly: A Randomized Clinical Trial. Journal of the American Medical Directors Association; 17(5):426-433.

**Keywords**

Community dwelling adults, Frailty phenotype, Risk of falls, Social participation.

### O39 Sexual assistance through the eyes of sex workers: one path to improve sexual lives of people with disabilities

#### Ana R Pinho^1^, Fernando A Pocahy^2^, Conceição Nogueira^1^

##### ^1^Center for Psychology, Faculty of Psychology and Education Sciences, University of Porto, 4200-135 Porto, Portugal; ^2^Universidade do Estado do Rio de Janeiro, 20550900 Rio de Janeiro, Brazil

###### **Correspondence:** Ana R Pinho (mipsi20699@fpce.up.pt)

**Background**

Historically, people with disabilities have been seen as asexual and their sexual rights were often neglected. Nowadays, some progresses have been made but they still face multiple stereotypes and barriers that limit their social and sexual lives. Sexual assistance is a way of sexual expression in which trained individuals provide sexual services to clients with disabilities, improving their well-being in relation to sexuality. However, in Portugal the only way to access commercial sex is through sex workers who have no training to attend disabled clients.

**Objective**

To understand if sex workers see training as a useful aspect to be taken into account for improving psychological and sexual health of clients with disabilities and themselves.

**Methods**

An explorative study of qualitative approach, with 13 sex workers interviews analysed using the thematic analysis method proposed by Braun and Clarke (2006) [1].

**Results**

From the analysis of the interviews four themes have emerged. Sex workers theme focus on the life experiences and motivations to attend clients with disabilities. Clients theme characterizes who are the people with disabilities seeking commercial sex. Search for sex work theme deepens knowledge about how they get in touch with sex workers. Finally, the attendance theme explains the dynamics of the relationship established and the many obstacles they overcome in order to express their sexuality through commercial sex.

**Conclusions**

The main conclusions provide evidence of the use of commercial sex by people with disabilities who seek in this service sexual and emotional satisfaction. Certain relationship specificities tend to be experienced with feelings of embarrassment on the part of professionals. Based on the experiences and obstacles sex workers observed when working with people with disabilities, measures were pointed out to improve the psychological and sexual health of those involved in the situation, which highlights the need for training to serve this group of clients, as well as the need for legalization of sex work.

**References**

1. Braun V, Clarke V. Using thematic analysis in psychology. Qualitative Research in Psychology. 2006; 3: 77-101.

**Keywords**

Sexual health, Sexual Assistance, Sex Work, Clients with Disabilities.

### O40 Results of an intervention program for men who batter women: perceptions of accompanied men

#### Anne CLG Silva, Elza B Coelho

##### Department of Public Health, Federal University of Santa Catarina, 88040-900 Florianópolis, Santa Catarina, Brazil

###### **Correspondence:** Anne CLG Silva (anne_clg@hotmail.com)

**Background**

In intimate partner violence, man is the main perpetrator of violence, and it is essential to include him in interventions to decrease violence, because he can take responsibility for violence, seeking new forms of expression. However, intervention with men is criticized, such as: using resources that could be targeted to victims; the imposition of re-education measures rather than punitive measures; consider that men do not change their behaviour [1]. Nevertheless, it is within the framework of evaluation that we find one of the major shortcomings of batterer intervention programs, since the effects of participation of men in that, have been receiving little analysis [2].

**Objective**

This research aims to analyse the results of a batterer intervention program from the perspective of man accompanied by the program.

**Methods**

It is a case study conducted in a batterer intervention program with 86 men. It was used the Centres for Disease Control and Prevention Follow Up Questionnaire, adapted to be used in Brazil. Data were analysed according to content analysis techniques. This project was approved by the Human Research Ethics Committee of the Infantile Hospital Joana de Gusmão. Subjects were asked to agree through an informed consent.

**Results**

When asked about changes occurred after 3 months of follow-up in the program some men reported having not noticed any changes, which indicates that the program is not effective to all participants and the importance of longer follow-ups. However, most men cited changes in the way they act and perceive the division of tasks between men and women, thus participation in the program can be the starting point for rethinking and building new ways of expressing masculinity. And the changes cited go beyond the scope of the marital relationship, encompassing the relationship with the children, the abandonment of addictions and the desire to seek school education.

**Conclusions**

According to the data, attention to perpetrators of violence has a positive influence not only in the behaviour towards the partner, but also on the relationship with children and the abandonment of addictions. Although longer follow-ups - including the couple - are needed, the batterer intervention program may be a tool to decrease violence against women.

**References**

1. Antezana AP. Intervenção com Homens que Praticam violência contra seus cônjuges: reformulações teórico-conceituais para uma proposta de intervenção construtivista-narrativista com perspectiva de gênero. Nova Perspectiva sistêmica, 2012; 42:9-27.

2. Toneli MJF; Lago MCS; Beiras A; Climaco DA, organizadores. Atendimento a homens autores de violência contra as mulheres: experiências latino-americanas. Florianópolis: UFSC/CFH/NUPPE; 2010.

**Keywords**

Violence against women, Batterer intervention, Men, Program evaluation.

### O41 Effectiveness of a reminiscence program on cognitive frailty, quality of life and depressive symptomatology in the elderly attending day-care centres

#### Isabel Gil^1^, Paulo Costa^2^, Elzbieta Bobrowicz-Campos^2^, Rosa Silva^3^, Maria L Almeida^1^, João Apóstolo^4^

##### ^1^Nursing School of Coimbra, 3046-851 Coimbra, Portugal; ^2^Health Sciences Research Unit: Nursing, Nursing School of Coimbra, 3046- 851 Coimbra, Portugal; ^3^Universidade Católica Portuguesa, Institute of Health Sciences, 4200-374 Porto, Portugal; ^4^Health Sciences Research Unit: Nursing, Portugal Center for Evidence-Based Practice: A JBI Centre of Excellence, 3046-851 Coimbra, Portugal

###### **Correspondence:** Isabel Gil (igil@esenfc.pt)

**Background**

Reminiscence is a therapeutic intervention based on the account of personal experiences that allows access to significant life events. Evidence suggests that this intervention is particularly beneficial for the elderly with neurocognitive disorders [1], especially with regard to psychosocial variables. In Portugal, this intervention is underused, and there is a need to study its applicability and efficacy.

**Objective**

To evaluate the effect of a reminiscence-based program (RBP) [2] on cognitive frailty, quality of life and depressive symptomatology in elderly people attending day-care centres. Evaluate professional's satisfaction with the program and identify obstacles in its implementation.

**Methods**

A quasi-experimental study with one group was carried out in four day-care centres in the central region of Portugal. The framing sample included 69 older adults aged ≥ 65 years. Of those, 28 (average age of 79.33 ± 7.35 years and average education of 3.29 ± 1.86 years) participated in the 7-week RBP, twice a week. Outcomes of interest were cognitive frailty indicators measured through the Montreal Cognitive Assessment (MoCA); quality of life measured using the short version of World Health Organization Quality of Life Scale-module for older adults (WHOQOL-OLD-8); and depressive symptomatology measured by the 10-item Geriatric Depression Scale (GDS-10). In addition, the eight professionals conducting the study were asked to identify obstacles to the successful implementation of the program, and to evaluate its structure, themes, contents, and the involvement of the elderly in each session.

**Results**

RBP was shown to have positive effects on the MoCA and WHOQOL-OLD-8 score (p < 0.05). Improvement in the GDS-10 score was observed; however, it was statistically non-significant. The structure of the program sessions was considered as mostly clear and perceptible (94%), and themes and contents as mostly pleasant and appropriate (94%). Positive feedback was obtained regarding the program capacity to involve the elderly in the activities proposed (87.5%). However, according to the professionals’ opinion, there is a need for ampler capacitation of the teams implementing RBP, better articulation with the institutions regarding the used space and activities schedule, and better articulation with the elderly to guarantee their commitment to the program.

**Conclusions**

Reminiscence was shown to be effective in improving cognition and quality of life, as well as potentially effective in decreasing depressive symptomatology. Therefore, it presents a therapeutic potential, contributing to the improvement of the care provided. It is also worth mentioning the good acceptance of the program which, however, implies the qualification of the professional teams for its implementation.

**Acknowledgements**

This study was developed within the context of the project “664367/FOCUS” (funded under the European Union’s Health Programme (2014-2020)) and project ECOG (funded by the Nursing School of Coimbra).

**References**

1. Thorgrimsen L, Schweitzer P, Orrell M. Evaluating reminiscence for people with dementia: a pilot study. The Arts in Psychotherapy. 2002;29(2):93-97.

2. Gil I, Costa P, Bobrowicz-Campos E, Cardoso D, Almeida M, Apóstolo J. Reminiscence therapy: development of a program for institutionalized older people with cognitive impairment. Revista de Enfermagem Referência. 2017;4(15):121-132.

**Keywords**

Reminiscence, Elderly, Cognition, Quality of life, Depressive symptomatology.

### O42 Development and validation of a reminiscence group therapy program for older adults with cognitive decline in institutional settings

#### Isabel Gil^1^, Paulo Costa^2^, Elzbieta Bobrowicz-Campos^2^, Rosa Silva^3^, Daniela Cardoso^4^, Maria Almeida^1^, João Apóstolo^4^

##### ^1^Nursing School of Coimbra, 3046-851 Coimbra, Portugal; ^2^Health Sciences Research Unit: Nursing, Nursing School of Coimbra, 3046- 851 Coimbra, Portugal; ^3^Universidade Católica Portuguesa, Institute of Health Sciences, 4200-374 Porto, Portugal; ^4^Health Sciences Research Unit: Nursing, Portugal Center for Evidence-Based Practice: A JBI Centre of Excellence, 3046-851 Coimbra, Portugal

###### **Correspondence:** Isabel Gil (igil@esenfc.pt)

**Background**

Research has evidenced the positive impact of non-pharmacological therapies aimed at elderly people with cognitive decline in the institutional setting. Reminiscence Therapy (RT) emerges in this category as an enabling strategy, which favours moments of happiness, dignity and life purpose [1]. Nonetheless, studies centred in RT are limited in Portugal, with a clear absence of structured interventional programs, emerging the need to develop and validate well-defined and replicable RT programs [2].

**Objective**

We intend to construct and validate a RT program directed to elderly people with cognitive decline, to be implemented in institutional settings by healthcare professionals.

**Methods**

Guidelines for complex interventions development from the Medical Research Council were followed [3]. The program was conceptualized in four distinct phases: Phase I (Preliminary), the initial conceptualization of the program design and supportive materials; Phase II (Modelling), consisting in the conduction of interviews and focus groups with healthcare specialists; Phase III (Field Test), aiming at the evaluation of each program session; and Phase IV (Consensus Conference), to synthesize the contributions and analyse challenges that emerged in preceding phases.

**Results**

Based on the contributions of experts, healthcare professionals and the institutionalized elderly, a RT program divided into two strands was formed. The main strand includes 14 sessions, performed twice a week. The maintenance strand included seven weekly sessions. Each thematic session is related to the participants' life course, with a maximum duration of 60 minutes. The 4-phase conceptualization process resulted in the creation of a digital platform with audio-visual contents to aid professionals during each session; inclusion of an introductory section that contextualizes the therapeutic potential of RT; introduction of complementary activities that can be developed additionally in the institutional settings; reinforcement of multisensory stimulation throughout the program; introduction of a final moment of relaxation through abdominal breathing. The terminology used and visual presentation of the program were reformulated in order to improve user experience. The created program was considered by the elderly and healthcare professionals involved during the course of this process as pleasant and interesting, praising its structure, thematic contents and proposed activities.

**Conclusions**

The involvement of experts and potential users enabled the program to mirror the needs of the elderly with cognitive decline in an institutional setting. The RT program, structured and validated in the course of this study, demonstrated characteristics adjusted to the target population and setting. However, the effectiveness of the program should be tested in a future pilot study.

**Acknowledgements**

This study was developed within the context of the project ECOG, funded by the Nursing School of Coimbra.

**References**

1. Subramaniam P, Woods B. The impact of individual reminiscence therapy for people with dementia: systematic review. Expert Review of Neurotherapeutics. 2012;12(5):545-555.

2. Berg A, Sadowski K, Beyrodt M, Hanns S, Zimmermann M, Langer G et al. Snoezelen, structured reminiscence therapy and 10-minutes activation in long term care residents with dementia (WISDE): study protocol of a cluster randomized controlled trial. BMC Geriatrics. 2010;10(1).

3. Craig P, Dieppe P, Macintyre S, Michie S, Nazareth I, Petticrew M. Developing and evaluating complex interventions: the new Medical Research Council guidance. BMJ. 2008.

**Keywords**

Cognitive dysfunction, Aged, Program development, Reminiscence therapy.

### O43 Short-term efficacy of a nursing psychotherapeutic intervention for anxiety on adult psychiatric outpatients: a randomised controlled trial

#### Francisco Sampaio^1,2,3^, Odete Araújo^3,4^, Carlos Sequeira^2,4^, Teresa L Canut^5^, Teresa Martins^2,4^

##### ^1^Psychiatry Department, Hospital of Braga, 4710-243 Braga, Portugal; ^2^Nursing School of Porto, 4200-072 Porto, Portugal; ^3^Center for Health Technology and Services Research, 4200-450 Porto, Portugal; ^4^School of Nursing, University of Minho, 4710-057 Braga, Portugal; ^5^Department of Public Health, Mental Health and Perinatal Nursing, School of Nursing, Barcelona University, 08907 Barcelona, Spain

###### **Correspondence:** Francisco Sampaio (fmcsampaio@gmail.com)

**Background**

Several efficacious treatments for anxiety are available, among which different forms of psychotherapy and pharmacotherapy [1]. However, literature favour more findings stemming from studies about the efficacy of psychotherapies/therapies provided by nurses [2,3] than those arising from studies about the efficacy of nursing psychotherapeutic interventions (interventions classified, for instance, on Nursing Interventions Classification) [4]. Moreover, no studies were found in literature about the efficacy of psychotherapeutic interventions on anxiety as a symptom.

**Objectives**

Evaluating the short-term efficacy of a psychotherapeutic intervention in nursing on Portuguese adult psychiatric outpatients with the nursing diagnosis “anxiety”.

**Methods**

A single-blind randomised controlled trial was conducted at a Psychiatry Ward Outpatient Service of a Hospital in the north of Portugal. Participants were psychiatric outpatients, aged 18-64, with nursing diagnosis “anxiety”, who were randomly allocated to an intervention group (n = 29) or a treatment-as-usual control group (n = 31). The interventions consisted in psychotherapeutic interventions for the nursing diagnosis “anxiety”, integrated in the Nursing Interventions Classification. One mental health nurse provided the individual-based intervention over a 5-week period (one 45-60 minutes weekly session). A treatment-as-usual control group received only pharmacotherapy (if applicable). The primary outcomes, anxiety level and anxiety self-control, were assessed with the outcomes “Anxiety level” and “Anxiety self-control”, integrated in the Nursing Outcomes Classification (Portuguese version) [5] respectively. Time frames for assessment were at baseline and post-test (6 weeks after).

**Results**

Patients from both groups presented improvements in anxiety levels, between the pre-test and the post-test assessment; however, analysis of means showed that patients of the intervention group presented significantly better results than those of the control group. Furthermore, only patients in the intervention group presented significant improvements in anxiety self- control. The psychotherapeutic intervention presented a very large effect size on the anxiety level and a huge effect size on the anxiety self-control. 22.8% and 40% of the outcomes related to the anxiety level and anxiety self-control, respectively, are predicted in the event of integrating the intervention group.

**Conclusions**

This study demonstrated the psychotherapeutic intervention model in nursing was efficacious in the decrease of anxiety level and improvement of anxiety self-control in a group of Portuguese adult psychiatric outpatients with pathological anxiety, immediately after the intervention. The results of the multiple linear regression and the very large effect size identified suggest that a significant part of the improvements could be directly attributed to the intervention.

**Trial Registration Number**

NCT02930473

**References**

1. Cuijpers P, Sijbrandij M, Koole SL, Andersson G, Beekman AT, Reynolds CF. The efficacy of psychotherapy and pharmacotherapy in treating depressive and anxiety disorders: a meta-analysis of direct comparisons. World Psychiatry. 2013, 12: 137-148.

2. Asl NH, Barahmand U. Effectiveness of mindfulness-based cognitive therapy for comorbid depression in drug-dependent males. Arch Psychiatr Nurs. 2014, 28: 314- 318.

3. Hyun M, Chung HC, De Gagne JC, Kang HS. The effects of cognitive-behavioral therapy on depression, anger, and self-control for Korean soldiers. J Psychosoc Nurs Ment Health Serv. 2014, 52: 22-28.

4. Bulechek GM, Butcher HK, Dochterman JM, Wagner C. Nursing Interventions Classification (NIC). 6th ed. St. Louis: Elsevier; 2012.

5. Moorhead S, Johnson M, Maas ML, Swanson E. Nursing Outcomes Classification (NOC). 5th ed. St. Louis: Elsevier; 2013.

**Keywords**

Anxiety, Clinical nursing research, Nursing, Psychiatric nursing, Psychotherapy, Brief.

### O44 “Art therapy” in acute psychiatry: a Portuguese case study

#### Clara Campos^1^, Aida Bessa^1^, Goreti Neves^1^, Isabel Marques^2^, Carlos Laranjeira^3^

##### ^1^Centro Hospitalar e Universitário de Coimbra, 3000-075 Coimbra, Portugal; ^2^Escola Superior de Enfermagem de Coimbra, 3046-851 Coimbra, Portugal; ^3^Hospital Distrital da Figueira da Foz, 3094-001 Figueira da Foz, Portugal

###### **Correspondence:** Clara Campos (ccalmeidacampos@gmail.com)

**Background**

In the last few years, some researchers have focused on the valorisation of interventions that stimulate the use of art therapy in individuals with mental illness. This assessment is based on the assumption that biological programs (including psycho pharmaceuticals) should be increasingly inclusive, and therefore should include psychosocial approaches based on the recovery model. However, there is as yet no effective consensus on techniques and interventions that reveal greater effectiveness as well as systematization.

**Objectives**

a) Evaluate the effectiveness of a program of 3 sessions of “art therapy” in individuals with mental illness, in the change of emotional indicators, namely depression, anxiety, stress, and psychological well-being; b) analyse the meanings attributed by the person to his creative self-expression.

**Methods**

We chose a pre-experimental study, of mixed approach (quantitative and qualitative), with pre- and post-test design and without control group. Twelve male subjects mostly diagnosed with Schizophrenia and Mood Disorders, who were admitted to an acute psychiatry unit, participated in the study. The instruments used to collect information were: Depression, Anxiety and Stress Scale [DASS-21]; Subjective Well-Being Scale (EBEP-18 items) and a semi-structured interview.

**Results**

The main results suggest, after the evaluation between the pre- and post-test that there was an improvement in the dimensions anxiety, stress, self-acceptance, life goals and overall psychological well-being. The categories that resulted from the thematic analysis of the interviews (hope for the future, learning to manage difficulties and dealing with difficult emotions) revealed the usefulness of the program in the participant’s recovery process.

**Conclusions**

The inclusion of this type of psychosocial intervention in specialized clinical practice in Mental Health Nursing allows minimizing the impact of the disease in an organizational culture that should increasingly be oriented towards recovery.

**Trial registration**

NCT03575442

**Keywords**

Recovery, Art therapy, Mental Health Nursing.

### O45 Nursing care at the postpartum home visit: the couple perspective

#### Bárbara Pinto^1^, Marília Rua^2^, Elsa Melo^2^

##### ^1^Unidade de Cuidados de Saúde Primários Estarreja I, Agrupamento de Centros de Saúde Baixo Vouga, 3860-335 Beduído, Portugal; ^2^Escola Superior de Saúde, Universidade de Aveiro, 3810-193 Aveiro, Portugal

###### **Correspondence:** Bárbara Pinto (barbarapinto_41@hotmail.com)

**Background**

The birth of a child corresponds to a new stage in the family life cycle and implies a process of restructuring, adaptation to physical, psychological, family and social readjustments [1]. This transition predicts a change of roles of all the members of the family and the construction of a new personal, conjugal and familiar identity [2]. From institutions and health professionals, interventions are expected to successfully overcome these challenges. At this stage, the home visit imposes itself as an important intervention in Nursing care. Its accomplishment, by the family nurse, promotes individual and family empowerment and autonomy in healthy parenting.

**Objectives**

Understanding the couple's perception about nursing practices in the context of home visit postpartum, as a contribution to the transition to parenting.

**Methods**

The research was based on the phenomenological domain, in a qualitative approach and includes eleven couples experiencing parenthood for the first time, between October 2016 and January 2017, enrolled in the Family Health Unit of Barrinha. Data collection included semi-structured interviews were conducted in order to guarantee the narratives of the experiences and their deeper understanding. The information was analysed according to the technique of content analysis, using WEBQDA software.

**Results**

This study revealed that the birth of the first child is an event of individual and family development and growth, which implies adaptation to a set of changes and redefinition of roles, built on a day-to-day basis, and in close cooperation between the family and the nursing team. The approach in the home nursing visit was directed to the well-being of the new born and its mother, appearing to the family as a resource, not having the concern to explore the interaction and the reciprocity within the family. We highlight three dimensions: Postpartum home visit, that describes the experiences of the participants about the care operationalized in this visit; Family Nursing, which traces the way they understand the work of the family nurse in this transition and, lastly, the Postpartum parenting, which reports the mother perception about this stage of the life cycle.

**Conclusions**

The home visit and work philosophy by the family nurse contributed to the positive adaptation to parenthood and to approach the family and added value for improvement of the quality of health care, yet it was not assumed as a reality in the context of caring.

**References**

1. Walsh F. Processos normativos da família: diversidade e complexidade. 4 ed. Porto Alegre: Artmed; 2016.

2. Martins C, Abreu W, Figueiredo MC. Transição para a parentalidade: A Grounded Theory na construção de uma teoria explicativa de Enfermagem. Investigação qualitativa em saúde. 2017;2(2017):40-49.

**Keywords**

Family Nurse, Family, Puerperium, Home visit.

### O46 Skills of occupational therapy students required for an effective relationship

#### María Y González-Alonso^1^, Valeriana G Blanco^1^, Reninka De Koker^2^, Luc Vercruysee^2^

##### ^1^Department of Health Sciences, University of Burgos, 09001 Burgos, Spain; ^2^Department of Occupational Therapy, University Odisee, 1000 Brussel, Belgium

###### **Correspondence:** María Y González-Alonso (mygonzalez@ubu.es)

**Background**

The acquisition of skills throughout the career facilitates the professional practice and satisfaction of the occupational therapist. In order to give direct attention to a situation of disability or risk, the professional must apply the best evidence-based strategy; and to establish a productive relationship with the client, the professional needs to learn to use interpersonal skills.

**Objective**

The objective of the study was to analyse how the perception of occupational therapy students changes their personal traits and challenges throughout their careers.

**Methods**

This is a descriptive, cross-sectional study of an intentional sample consisting of 183 students of occupational therapy. The study is part of the 2016-2017 academic course. An ad hoc questionnaire was prepared based on the collection of personal data and the perception of 29 skills that [1] proposed: The students should value the traits and challenges.

**Results**

Of the 183 students, 122 were from the first and 61 from the final year, 47.5% from Belgium and 52.5% from Spain. The profile of the sample was 85.8% women; 60.1% live with their family and 85.8% had not done work placements outside their country. Regarding the skills that defined them, the respondents indicated friendly, respectful and loyal, with an average of 22.4 skills. Regarding abilities that they felt they must achieve, they identified patient, firm and assertive with an average of 9.9. The first-year students self-evaluated more positively than those in their final year in respect to the different variables. Significant differences related to the course were only observed in two traits: empathetic and collaborative.

**Conclusions**

Occupational therapy students, those in both their first and final years, consider that they have a large number of relational skills which enable them to give an appropriate response to the events that occur in therapy. Empathy is the only trait which indicates differences depending on the independent variables studied. for improvement of the quality of health care, yet it was not assumed as a reality in the context of caring.

**References**

1.Taylor, R.R. The International relationship: Occupational Therapy and the use of self; Philadelphia: F.A. Davis; 2008.

**Keywords**

Occupational Therapy, Traits, Challenges, Interpersonal Relationships, Attitudes.

### O47 Use of performance-enhancing substances in Portuguese gym/fitness users: an exploratory study

#### Ana S Tavares, Elisabete Carolino

##### Escola Superior de Tecnologia da Saúde de Lisboa, Instituto Politécnico de Lisboa, 1990-096 Lisboa, Portugal

###### **Correspondence:** Ana S Tavares (ana.tavares@estesl.ipl.pt)

**Background**

The use of performance-enhancing substances (PES) by competitive or recreational sports practitioners is a pertinent and current topic, particularly in the field of public health. People who use gyms come from diverse socio-demographic conditions, where the consumption of this type of substances is not only used for the purpose of improving physical performance, but also to obtain a more muscular physique, especially for men, and leaner, especially for women whose goal is faster weight loss [1]. In Portugal there are practically no studies on the use of PES outside competitive sport, highlighting a study developed in 2012 by the European Health & Fitness Association [2].

**Objective**

Investigate the prevalence and profile of PES users amongst a sample of Portuguese gym/fitness users.

**Methods**

Cross-sectional, quantitative and exploratory study, amongst a convenience sample of 453 Portuguese gym/fitness users, recruited, directly on social networks (Facebook) and by institutional email (via gyms). Data were collected via a structured on-line questionnaire. Statistical analysis was performed using SPSS 22.

**Results**

Among the 453 gym/fitness users (61.3% female; 38.7% male) who participated in the survey, 50 (11.1%) reported PES use (5.4% female; 19.5% male). The mean age of PES users was 34.96 years (Std. Dev. = 10.00). They were married, unemployed and with a low level of education (until 9 years = 41.7%). PES users showed more years of training (4 years) than no PES users. The main sports modalities of the respondents were cardio fitness (57.0%), bodybuilding (56.5%), stretching (27.8%) and localized (27.2%). PES use was suggested mostly by friends (51.9%), peers (30.8%) and by internet (30.8%). The most commonly consumed PES were diuretics (46.0%) and anabolic steroids (44.0%). Thirty percent of PES users reported side effects and the most commonly reported was acne (53.3%), agitation and tremors (40.0%). The main reason for using PES is the improvement of the physical condition (54.0%). Five-point three percent of non-PES users expressed an interest in using PES in the future.

**Conclusions**

This exploratory survey revealed the use of PES amongst Portuguese gym/fitness users and its increasing importance to investigate the psychosocial factors that may influence PES use in this specific population. Exploring these factors may improve the effectiveness of practical interventions and motivational strategies to reduce PES use among gym/fitness users.

**References**

1. European Health and Fitness Association. Fitness against doping: Relatório intercalar - principais resultados. Bruxelles; 2011.

2. European Health and Fitness Association. Executive summary of the final report for the Copenhagen Fitness Anti-Doping Conference. Bruxelles; 2012.

**Keywords**

Performance enhancing substances, Orevalence, Gym/fitness users.

### O48 Identification of frailty condition of elderly people in the community

#### Inês Machado^1^, Pedro Sá-Couto^2^, João Tavares^3,4^

##### ^1^Department of Medical Sciences, University of Aveiro, 3810-193 Aveiro, Portugal; ^2^Center for Research and Development in Mathematics and Applications, Department of Mathematics, University of Aveiro, 3810-193 Aveiro, Portugal; ^3^Nursing School of Coimbra, 3046-851 Coimbra, Portugal; ^4^Coimbra Education School, Polytechnic Institute of Coimbra, 3030-329 Coimbra, Portugal

###### **Correspondence:** Inês Machado (inessantiago@live.ua.pt)

**Background**

Frailty is a geriatric syndrome with multiple causes and contributors that is characterized by diminished strength, endurance, and reduced physiologic function that increases an individual’s vulnerability for developing increased dependency and/or death [1]. Current research is still under discussion regarding the nature, definition, characteristics and prevalence of frailty. Identify frail older adults (OA) has recently been recognized as an important priority, especially in community-dwelling OA.

**Objective**

Determine the prevalence of frail OA in a primary care (PC) settings and to assess the concurrent validation of the Portuguese version of Prisma7 (P7) with two other published and validated instruments: Frailty Phenotype (FF) and the Groningen Frailty Indicator (IFG).

**Methods**

This study was conducted in one PC unit in the north region of Portugal with a convenience sample of 136 OA (≥ 65 years). The questionnaire included: 1) sociodemographic, family and health variables; and 2) the frailty instruments P7, FF and IFG. OA were considered frail: ≥ 3 positive questions out of 7 for P7; ≥3 factors out of 5 for FF; and ≥ 4 dimensions out of 8 for IFG. Further details about these scales can be found in Machado. For the concurrent validity, methods based on correlation (Spearman Rank test) and agreement (Cohen's Kappa, sensitivity and specificity values) were used. For comparison of the two groups (frailty or non-frailty), an independent t-test was calculated. Finally, binary logistic regression model was considered to identify predictors of frailty.

**Results**

According to the characterization of P7, IFG and FF, the prevalence of frail OA was 7.4%, 19.9% and 26.5%, respectively. The agreement percentage between the instruments was moderate ranging from 68% to 77%, observing that the P7 is partially concordant with the other instruments. The P7 showed high specificity values, but low sensitivity values. Frail OA were characterized (p < 0.05) as being older, having worse health perception, lower physical capacity, slower walking velocity, higher IFG scores, and decreased hand grip strength. As predictors of frailty, in the multivariate model, older age (OR = 1.111) and better physical capacity (OR = 0.675) were significant (p < 0.01).

**Conclusions**

A sample of more robust people and a “synthetic” application of P7 (without explaining the questions) may have influenced the prevalence results presented. More studies are needed in order to further evaluate the psychometric properties of the various tools tested. The P7 should be used with caution in identifying frailty in PC, therefore we suggest the incorporation of another measure of frailty assessment.

**Acknowledgements**

This work was supported in part by the Portuguese Foundation for Science and Technology (FCT-Fundação para a Ciência e a Tecnologia), through CIDMA - Center for Research and Development in Mathematics and Applications, within project UID/MAT/04106/2013.

**References**

1. Morley JE, Vellas B, van Kan GA, et al. Frailty Consensus: A Call to Action. Journal of the American Medical Directors Association. 2013;14(6):392-397.

**Keywords**

Frailty, Elderly, Instrument, Prisma7.

### O49 Use of software in learning difficulties of reading: comparative analysis between digital environment and hybrid environment

#### Ana Sucena^1,2,3,4^, Ana F. Silva^1,2^

##### ^1^Instituto Politécnico do Porto, 4200-465 Porto, Portugal; ^2^Centro de Investigação e Intervenção na Leitura, Instituto Politécnico do Porto, 4200-465 Porto, Portugal; ^3^Centro de Investigação em Estudos da Criança, Instituto de Educação, Universidade do Minho, 4710-057 Braga, Portugal; ^4^Centro de Investigação em Reabilitação, Escola Superior de Saúde, Instituto Politécnico do Porto, 4200-465 Porto, Portugal

###### **Correspondence:** Ana Sucena (sucena.ana@gmail.com)

**Background**

The learning difficulties of the letter-sound relations are seen as a risk factor for future difficulties in learning to read [1]. Ideally, the identification of children at risk of failure to learn reading and writing should occur in the last year of pre-school or early in the first year, so that intentional programs can be implemented to promote basic reading skills [2,3,4]. The most promising reading learning support programs combine explicit phonological awareness training with highly structured reading instruction [5,6].

**Objective**

This study evaluated the impact of two early intervention programs on reading learning difficulties. A program exclusively in virtual environment and a hybrid program, comprising sessions in virtual environment and in real environment.

**Methods**

Participants were 57 children, attending the first year of schooling, native speakers of European Portuguese, identified as at risk of having learning reading difficulties. The children were divided into three groups: (a) virtual environment intervention - training with Graphogame software, (b) hybrid intervention - training using Graphogame software and real-time sessions of pre-reading and reading skills oriented by a technician from the CiiL team (Center for Research and Intervention in Reading) and (c) absence of intervention beyond that provided for in the regular system of education. The intervention programs were developed in a school context, with the virtual component (Graphogame) developed with daily periodicity, with duration between 10 to 15 minutes. The intervention in real environment was carried out once a week, with activities of 30 to 40 minutes, using materials of a playful character, created specifically for the present study. In both types of sessions, the groups consisted of two to five children. The participants were evaluated at the level of letter-sound relations, phonemic awareness, word reading and pseudo word reading.

**Results**

Both intervention environments produced significantly more positive effects than those obtained by the control group. Still, the software Portuguese Basis Graphogame is an effective tool, however, with a more positive effect when used in parallel with a face-to-face reading promotion session.

**Conclusions**

The early intervention in reading difficulties should promote the explicit training of phonemic awareness and letter-sound relations in order for the decoding process to be developed. Although the virtual environment – in this case the software Portuguese Basis Graphogame – is a highly effective tool, ideally, it should be combined with a real-environment intervention to ensure that the child effectively dominates letter-sound relationships and that trains intensively the decoding process.

**References**

1. Lyytinen H. State-of-Science Review: SR-D12 New Technologies and Interventions for Learning Difficulties: Dyslexia in Finnish as a Case Study. Foresight Mental Capital and Wellbeing Project: The Government Office for Science. London: UK. 2008.

2. Hatcher P, Hulme C, Snowling M. Explicit phoneme training combined with phonic reading instruction helps young children at risk of reading failure. Journal of Child Psychology and Psychiatry, University of York, UK. 2004, 45: R338-358

3. Wimmer H, Mayringer H. Dysfluent reading in the absence of spelling difficulties: A specific disability in regular orthographies. Journal of Educational Psychology. 2002, 94: R272-277

4. Saine N, Lerkkane M, Ahonen T, Tolvanen A, Lyytinen H. Computer-Assisted Remedial Reading Intervention for School Beginners at Risk for Reading Disability. Child Development. 2011, 82: R1013-1028

5. Hatcher P, Hulme C, Ellis A. Ameliorating early reading failure by integrating the teaching of reading and phonological skills: The phonological linkage hypothesis. Child Development. 1994, 65: R41-57

6. Hatcher P, Hulme C, Miles J, Carroll J, Hatcher J, Gibbs S, Smith G, Bowyer-crane C, Snowling M. Efficacy of small group reading intervention for beginning readers with reading-delay: a randomised controlled trial. Journal of Child Psychology and Psychiatry. 2006, 47: 820-827.

**Keywords**

Graphogame, Reading acquisition, Reading intervention.

### O50 Consumption patterns of non-steroidal anti-inflammatory drugs and attitudes towards the medicine residues in north and central regions of Portugal

#### Andreia Carreira^1^, Catarina Valente^1^, Joana Tomé^1^, Tânia Henriques^1^, Fátima Roque^1,2^, Márcio Rodrigues^1,2^, Maximiano P Ribeiro^1,2^, Paula Coutinho^1,2^, Sandra Ventura^1,2^, Sara Flores^1,2^, Cecília Fonseca^1,2^, André RTS Araujo^1,2,3^

##### ^1^School of Health Sciences, Polytechnic Institute of Guarda, 6300-749 Guarda, Portugal; ^2^Research Unit for Inland Development, Polytechnic Institute of Guarda, 6300-559 Guarda, Portugal; ^3^LAQV/REQUIMTE, Department of Chemical Sciences, Faculty of Pharmacy, University of Porto, 4050-313 Porto, Portugal

###### **Correspondence:** André RTS Araujo (andrearaujo@ipg.pt)

**Background**

Non-steroidal anti-inflammatory drugs (NSAIDs) are one of the most commonly used medications in the world because of their analgesic, antipyretic and anti-inflammatory properties [1–3]. However, their use is associated with the occurrence of serious adverse drug events, particularly gastrointestinal, cardiovascular and renal complications [3].

**Objective**

To assess the NSAIDs consumption pattern by the adult residents in the north and central regions of Portugal, as well as, to evaluate their individual's behaviour concerning the resulting residues after the use of the packages of medicines.

**Methods**

A questionnaire survey was administered to a sample of 400 pharmacy costumers in the districts of Aveiro, Leiria, Porto and Viseu between December 2015 and February 2016, with questions regarding the knowledge of NSAIDs consumption and their attitudes towards the medicine residues.

**Results**

In our study, the prevalence rate of NSAIDs use in the last 6 months was 74.3 % (95 % CI 70.0–78.6), showing a high level of consumption of this pharmacotherapeutic group. The most commonly used NSAID was ibuprofen (76.4 %), followed by diclofenac (36.0 %) and nimesulide (8.4 %). The most reported therapeutic indications were headaches (36.4 %), followed by back pain (33.3%), fever (24.6%) and flu (20.5%). Surprisingly, adverse drug events were reported by only 6.7 % of respondents. Even so, the most common adverse drug event was diarrhoea (4.0%). These results could be explained considering that NSAIDs use is episodic and limited to shorter periods and probably the respondents did not correlate the adverse effects of these medicines. Relatively to the destination of the packages of medicines that respondents no longer used, it was verified that 58% of the respondents claimed to deliver them in a pharmacy, 17.3% throw away in the common waste, 24.0% keep them at home, 0.5% put in sanitary sewers and 0.2% donate to charities.

**Conclusions**

According to these findings, it was evident the trivialization of NSAIDs consumption, being imperative to monitor their use and educate the users for its rational use. On another hand, it is important to maintain the incentive and to educate the population to adopt adequate attitudes regarding medicine residues recycling.

**References**

1. Cryer B, Barnett MA, Wagner J, Wilcox CM. Overuse and misperceptions of nonsteroidal anti-inflammatory drugs in the United States. Am J Med Sci. 2016, 352(5):472–80.

2. Green M, Norman KE. Knowledge and use of, and attitudes toward, non-steroidal antiinflammatory drugs (NSAIDs) in practice: A survey of ontario physiotherapists. Physiother Canada. 2016, 68(3):230–41.

3. Koffeman AR, Valkhoff VE, Celik S, W’t Jong G, Sturkenboom MCJM, Bindels PJE, et al. High-risk use of over-the-counter non-steroidal anti-inflammatory drugs: a populationbased cross-sectional study. Br J Gen Pract. 2014, 64(621):e191-8.

**Keywords**

Nonsteroidal anti-inflammatory drugs; Consumption patterns; Attitudes; Medicine residues.

### O51 Allergic rhinitis characterization in community pharmacy customers of Guarda city

#### Hélio Guedes^1^, Agostinho Cruz^2^, Cecília Fonseca^1,3^, André RTS Araujo^1,3,4^

##### ^1^School of Health Sciences, Polytechnic Institute of Guarda, 6300-749 Guarda, Portugal; ^2^School of Health Sciences, Polytechnic Institute of Porto, 4200-072, Porto, Portugal; ^3^Research Unit for Inland Development, Polytechnic Institute of Guarda, 6300-559 Guarda, Portugal; ^4^LAQV/REQUIMTE, Department of Chemical Sciences, Faculty of Pharmacy, University of Porto, 4050-313 Porto, Portugal

###### **Correspondence:** André RTS Araujo (andrearaujo@ipg.pt)

**Background**

Allergic rhinitis (AR) is a hypersensitivity reaction caused when inhaled particles contact the nasal mucosa and induce an immunoglobulin E -mediated inflammatory response resulting in sneezing, nasal itching, rhinorrhoea, nasal obstruction, or a combination of those symptoms [1]. The prevalence of AR is sometimes cited as 10% to 30% in adults [1]. It is increasingly recognized that the symptoms of AR often adversely impact the quality of life of the affected individuals and impose a significant health and socio-economic burden on the individual and society [2].

**Objective**

The aims of the present research were to estimate the prevalence of AR, determine the predominance of the symptoms, determine the impact on quality of life (QoL), as well as characterize the control strategies and treatment of AR in pharmacy customers of Guarda city.

**Methods**

An observational, cross-sectional and analytical study was conducted, and a questionnaire survey was developed and used as the data collection instrument. This included the Control of Allergic Rhinitis and Asthma (CARAT) test and the scale Quality of Life of the World Health Organization (WHOQOL-Bref). Data collection took place in community pharmacies in the city of Guarda between May and December of 2014.

**Results**

In the sample of 804 respondents, there was a predominance of females (66.3%) and the average age was 48.3 ± 16.5 years. The prevalence rate of AR was 13.1% (95 % CI 10.8–15.4). About 40% of the respondents with AR had no medical diagnosis. It was verified that there weren’t differences by gender in terms of quality of life (p = 0.929) or in the control of the AR symptoms (p = 0.168). On another hand, a high level of education (higher education) seemed to be a factor that contributed to a better quality of life (p = 0.001) and to a better control of symptoms (p = 0.019). It was also observed that a better control of the symptoms of AR was associated with a better quality of life (Pearson’s r = 0.292, p = 0.003).

**Conclusions**

The prevalence rate was estimated between 10.8% and 15.4%, which resulted from the medical diagnosis and the symptomatic diagnosis made through the data collection instrument. The results indicated that although the respondents do not have properly controlled the AR and suffer from associated comorbidities, they have a reasonable quality of life indexes.

**References**

1. Mims JW. Epidemiology of allergic rhinitis. Int Forum Allergy Rhinol. 2014,4(S2):S18–20.

2. Maspero J, Lee BW, Katelaris CH, Potter PC, Cingi C, Lopatin A, et al. Quality of life and control of allergic rhinitis in patients from regions beyond western Europe and the United States. Clin Exp Allergy 2012, 42(12):1684–96.

**Keywords**

Allergic rhinitis; Community pharmacy customers; Quality of life.

### O52 Weight transfer during walking and functional recovery post-stroke in the first 6 months of recovery – an exploratory study

#### Marlene Rosa^1,2^ (marlene.rosa@ipleiria.pt)

##### ^1^School of Health Science, Polytechnic Institute of Leiria, 2411-901 Leiria, Portugal; ^2^Center for Innovative Care and Health Technology, Polytechnic Institute of Leiria, 2411-901 Leiria, Portugal

**Background**

One of the most controversial abnormal patterns during walking in patients with stroke occurs during weight transfer (WT) of the paretic lower limb, however no perception of the knee patterns developed during stroke recovery exists.

**Objective**

To explore the importance of the knee kinematic pattern in the weight transfer (WT) walking period for functional recovery in the first 6 months post-stroke.

**Methods**

Inpatients with a first ischemic stroke (< 3 months), able to walk, were evaluated (T0) and revaluated 6 months post-stroke (T1). Patients were video-recorded in the sagittal plane while walking at their self-speed and the video was used to classify the knee pattern during WT. Walking speed, self-perceived balance, knee muscle strength and sensory-motor function of the hemiparetic lower limb were also assessed. Participants were stratified according to the knee pattern recovery. Comparisons between and within groups were conducted.

**Results**

Thirty-two patients (70.28 ± 10.19 years; 25.54 ± 3.26 Kg/m^2^) were included. Different groups were identified, according to the knee pattern: (1) normal at T0 and T1 (N = 10); (2) normal pattern only at T1 (N = 7); (3) acquisition/change in the knee pattern deviation (N = 7); (4) maintenance of the knee pattern deviation (N = 8). Modifications in the normal knee pattern might be developed to reach acceptable levels of functioning performance (p > 0.05, Groups 1/ 3). Speed and balance recovery was restricted when an abnormal knee pattern in WT was observed (Group 3 and 4), being worst when this pattern persisted (Group 4).

**Conclusions**

The knee pattern correction in WT might have benefits for stroke recovery. A further understanding of the causes for deviations in the knee pattern in WT will help establishing stroke treatment priorities.

**Trial Registration**

NCT02746835

**Keywords**

Weight transfer, Gait, Stroke, Knee patterns.

### O53 Reference values of cardiorespiratory fitness field tests for the healthy elderly Portuguese

#### Patrícia Rebelo^1,2^, Ana Oliveira^1,2,3^, Alda Marques^1,2^

##### ^1^Respiratory Research and Rehabilitation Laboratory, School of Health Sciences, University of Aveiro, 3810-193 Aveiro, Portugal; ^2^Institute for Research in Biomedicine, University of Aveiro, 3810-193 Aveiro, Portugal; ^3^Faculty of Sports, University of Porto, 4200-450 Porto, Portugal

###### **Correspondence:** Patrícia Rebelo (patriciarebelo@ua.pt)

**Background**

Cardiorespiratory fitness (CRF) is recognized as an independent predictor of all age morbidity and mortality and is closely related with people’s functional capacity [1]. Recently, CRF has been described as a clinical vital sign, which highlights its role in health promotion and disease prevention [2]. The 6-min-walk test (6MWT), incremental shuttle walk test (ISWT), unsupported upper limb exercise test (UULEX) and the 1-min sit-to-stand test (1’STS) are worldwide tests to assess CRF. Reference values of these tests are however, lacking for the Portuguese elderly population. This hinders the interpretability and limits the confidence of clinical decision-making in the field of CRF.

**Objective**

To contribute for establishing reference values for the 6MWT, ISWT, UULEX and 1’STS in the Portuguese healthy elderly population.

**Methods**

A cross-sectional study was conducted with healthy elderly volunteers [3] recruited from the Centre region of Portugal. Each participant conducted two repetitions of the 6MWT, ISWT, UULEX and 1-min STS. The best repetition was considered for analysis. Descriptive statistics were used to determine reference values by age decade (61-70; 71-80; 81- 90) and gender. Two-way ANOVA was used to investigate significant effects for age/gender and their interaction. Values were presented as mean ± standard deviation or median [95%, Confidence Intervals].

**Results**

262 healthy people were enrolled (61.5% female; 75.0±0.5yrs), 125 completed the 6MWT (66.4% female; 75.1±0.7yrs), 83 the ISWT (57.3% female; 76.1±1.0yrs), 210 the UULEX (63.3% female; 75.7±0.6yrs) and 50 the 1’STS (54% female; 72.1±1.0yrs). Values decreased significantly along the decades and were statistically different between male and female (p < 0.05) across all tests. The following values were found for the I) 6MWT (61-70y: males - 519.7[484.7-554.7]m *vs*. females - 488.3[458.8-517.7]m; 71-80y: 461.0[389.5-532.5]m *vs*. 377.1[316.4-437.8]m; 81-90y: 294.0[226.5-361.6]m *vs*. 254.1[211.6-296.6]m); II) ISWT (61-70y: males - 515.0[304.2-725.8]m *vs*. females - 353.3[212.5-494.2]m; 71-80y: 428.8[299.9-557.6]m *vs*. 234.6.2[151.6-317.6]m; 81-90y: 131.8[45.3-218.4]m *vs*. 161.0[113.2-208.8]m); III) UULEX (61-70y: males - 9.6[8.5-10.7]min. *vs*. females - 8.3[7.4-9.2]min.; 71-80y: 8.5[7.1-9.9]min. *vs*. 6.6[5.4-7.8]min.; 81-90y: 5.8[4.4-7.1]min. *vs*. 4.7[3.9-5.9]min.) and IV) 1’STS (61-70y: males - 40.0[33.2-46.8]rep/min *vs*. females - 37.13[33.65-40.61]rep/min; 71-80y: 30.7[26.4-34.9]rep/min *vs*. 33.3[26.1-40.4]rep/min; 81-90y: 29.0[9.1-67.1]rep/min *vs*. 22.3[16.2-28.3]rep/min). Significant interactions between age and gender were only observed in the ISWT.

**Conclusions**

The population studied presented worse results in the 6MWT, similar results in the ISWT and better results in the 1’STS test comparing with international studies [4-6]. No studies were found for the UULEX test. These differences highlight the importance of using population specific reference values in CRF assessment. Further studies with larger and representative sample sizes are needed to confirm results.

**References**

1. Harber MP, Kaminsky LA, Arena R, Blair SN, Franklin BA, Myers J, et al. Impact of cardiorespiratory fitness on all-cause and disease-specific mortality: Advances since 2009. Prog Cardiovasc Dis. 2017; 60(1):11-20.

2. Ross R, Blair SN, Arena R, Church TS, Després J-P, Franklin BA, et al. Importance of assessing cardiorespiratory fitness in clinical practice: a case for fitness as a clinical vital sign: a scientific statement from the American Heart Association. Circulation. 2016;134(24)

3. Organization WH. World report on ageing and health: World Health Organization; 2015.

4. Casanova C, Celli B, Barria P, Casas A, Cote C, De Torres J, et al. The 6-min walk distance in healthy subjects: reference standards from seven countries. Eur Respir J. 2011;37(1):150-6.

5. Dourado VZ, Vidotto MC, Guerra RLF. Reference equations for the performance of healthy adults on field walking tests. J Bras Pneumol. 2011;37(5):607-14.

6. Strassmann A, Steurer-Stey C, Dalla Lana K, Zoller M, Turk AJ, Suter P, et al. Population-based reference values for the 1-min sit-to-stand test. Int J Public Health. 2013;58(6):949-53.

**Keywords**

Cardiorespiratory Fitness, Cardiorespiratory field tests, Reference values, Elderly population.

### O54 Prevalence and factors associated with frailty in the elderly attended in ambulatory care

#### Clóris RB Grden^1^, Luciane PA Cabral^1^, Carla RB Rodrigues^2^, Péricles M Reche^1^, Pollyanna KO Borges^1^, Everson A Krum^2^

##### ^1^Departamento de Enfermagem e Saúde Pública, Universidade Estadual de Ponta Grossa, 4748 Ponta Grossa, Paraná; ^2^Hospital Universitário Regional dos Campos Gerais, Universidade Estadual de Ponta Grossa, 84031-510 Ponta Grossa, Paraná

###### **Correspondence:** Clóris RB Grden (reginablanski@hotmail.com)

**Background**

The aging process, understood as dynamic and progressive, contributes to the reduction of physical reserves and a higher prevalence of pathological processes, predisposing the elderly to frailty [1]. Canadian researchers define frailty as a multifactor syndrome involving biological, physical, cognitive, and social factors [2], which contribute significantly to disability and hospitalization [3]. Considered a modern geriatric syndrome, it is related to physiological changes, diseases, polypharmacy, malnutrition, social isolation and unfavourable economic situation [4, 5].

**Objective**

The objective of this study was to identify the prevalence and factors associated with frailty in the elderly attended in outpatient care. A cross-sectional study was carried out with 374 elderly individuals in outpatient care between October 2015 and March 2016. Data collection was applied to the Edmonton Fragility Scale [2]. Data were analysed by Stata software version 12 and described by measures of frequency, mean and standard deviation (SD). Prevalence ratios (PR) were calculated to investigate associations between independent variables and frailty. The adjusted prevalence ratios were obtained by multiple Poisson regression analysis. It was started with a saturated model and the variables that were not statistically relevant were removed, since their exclusion did not modify the results of the independent variables that remained in the model. The statistical significance was p < 0.05. The study complied with national and international standards of research ethics involving human subjects and was approved by the Research Ethics Committee in Human Beings of the institution under registration CAAE: 34905214.0.0000.0105.

**Results**

The results showed a predominance of female (67.4%), married (54.4%), with low educational level (55.1%), who lived with relatives (46.3%). The mean age of participants was 67.9 years. Regarding the clinical variables, 97% of the elderly reported having some type of disease, 92.3% used medication, 56.9% had no urine loss, 4.5% used walking sticks, 65.8% denied falls and 69.8% hospitalization. Regarding the fragility syndrome, the mean score was 5.9 points, with 40.1% elderly classified as fragile and 59.9% non-fragile. After multiple regression analysis, the variables that remained associated with the fragility were gender (p = 0.002), low education (p = 0.01), falls (p = 0.005), urinary incontinence (p = 0.000) (p = 0.001), medications (p = 0.02) and hospitalization (p = 0.001).

**Conclusions**

The study identified important factors associated with frailty in the elderly attending the outpatient clinic. Such results may support the development of gerontological care plans aimed at preventing functional decline and negative outcomes of the syndrome.

**References**

1. Maciel GMC, Santos RS, Santos TM, Menezes RMP, Vitor AF, Lira ALBC. Avaliação da fragilidade no idoso pelo enfermeiro: revisão integrativa. R. Enferm. Cent. O. Min. 2016, 6(3):2430-2438.

2. Rolfson D, Majumdar S, Tsuyuki R, Tahir A , Rockwood K. Validity and reliability of the Edmonton Frail Scale. Age Ageing. 2006, 35(5):526-9.

3. Vermeiren S, Vella-Azzopardi R, Beckwée D, Habbig AK, Scafoglieri A, Jansen B, et al. Frailty and the prediction of negative health outcomes: a meta-analysis. J Am Med Dir Assoc. 2016; 17(12): 1163.e1–1163.e17.

4. Morley JE, Vellas B, Kan GAV, Anker SD, Bauer JM, Bernabei R, et al. Frailty consensus: a call to action. JAMDA. 2013, 14(6):392-7.

5. Dent E, Kowal P, Hoogendijk EO. Frailty measurement in research and clinical practice: a review. Eur J Intern Med. 2016, 31:3-10.

**Keywords**

Aged, Frail Elderly, Prevalence, Geriatric Nursing.

### O55 Trend of mortality for acute myocardial infarction in state Santa Catarina, Brazil, for the period from 1996 to 2014

#### Pedro CM Morais^1^, Aline Pinho^2^, Daniel M Medeiros^3^, Giovanna G Vietta^3^, Pedro F Simão^3^, Bárbara O Gama^3^, Fabiana O Gama^3^, Paulo F Freitas^3^, Márcia R Kretzer^3^

##### ^1^Secretaria Municipal de Saúde de Palhoça, 88132-149 Palhoça, Santa Catarina, Brasil; ^2^Hospital Universitário Polydoro Ernani de São Thiago, Universidade Federal de Santa Catarina, 88036-800 Florianópolis, Santa Catarina, Brasil; ^3^Universidade do Sul de Santa Catarina, Campus Pedra Branca, 88137-270 Palhoça, Santa Catarina, Brasil

###### **Correspondence:** Pedro CM Morais (pedromorais182@hotmail.com)

**Background**

Acute Myocardial Infarction (AMI) is a public health problem in the world and in Brazil, due to the high morbimortality rates observed. Despite major advances in treatment, AMI accounts for 30% of deaths in Brazil.

**Objective**

To analyse the mortality trend due to acute myocardial infarction in the State of Santa Catarina, from 1996 to 2014.

**Methods**

Ecological study of time series, based on the Database of the Mortality Information System, made available by the Department of Informatics of SUS (DATASUS). Selected deaths by AMI, ICD-10, code I21, of the resident population in the state, according to gender and age group. Performed simple linear regression. The Research Ethics Committee of the Southern University of Santa Catarina approved this study.

**Results**

There were 40,204 deaths from AMI between 1996 and 2014 in Santa Catarina, with small oscillations in mortality rates in the period, 40.33/100,000 inhabitants in 1996 and 36.58/100,000 in 2014 (β = -0.062, p = 0.546). There were higher rates in males, but stationary, with 49.41/100,000 inhabitants in 1996 and 45.62/100,00 in 2014 (β = -0.008; p = 0.949). The female sex presented a steady trend, with a rate of 31.19/100,000 inhabitants in 1996 and 27.49 / 100,000 in 2014 (β = -0,113; p = 0.224). The male and female age groups showed a decreasing and significant trend after 30 years. Male age group 60 and older presented high mortality rates, however, declining. It stands out that the male age group from 70 to 79 years of age presented a decrease in rates of -13,936 per year, with a variation from 626.51/100,000 inhabitants in 1996 to 379.21/100,000 inhabitants in 2014 (p > 0.001). In the male age group of 80 years or more the rate was 935.88/100,000 inhabitants in 1996 and 669.99/100,000 in 2014 (β = -12,267; p = 0.004). Female age group 70 and older presented high mortality rates. In the female age group of 70 to 79 years, there was a decrease from 399.60/100,000 inhabitants in 1996 to 193.78/100,000 in 2014 (β = -12,115; p < 0.001). The female age group aged 80 years and over, from 811.78/100,000 inhabitants in 1996, decreased to 497.81/100,000 in 2014 (β = -16,081, p < 0.001).

**Conclusions**

The trend of AMI mortality in Santa Catarina is stationary for both genres but decreasing significantly in the age groups over 30 years, with the greatest reductions over 70 years.

**Keywords**

Acute Myocardial Infarction, Mortality rate, Ecological study.

### O56 Family nurse intervention in the mental adjustment of patients with arterial hypertension

#### Ana Alves^1^, João Simões^1^, Alexandre Rodrigues^1^, Pedro Couto^2^

##### ^1^Escola Superior de Saúde da Universidade de Aveiro, 3810-193 Aveiro, Portugal; ^2^Departamento de Matemática, Universidade de Aveiro, 3810-193 Aveiro, Portugal

###### **Correspondence:** Ana Alves (anamargaridamartins@ua.pt)

**Background**

The increase on life expectancy, and the raise of chronic condition, represents new challenges to the family nurse practitioner. Cardiovascular diseases are the main cause of death among the population, however the numbers have been declining in past years [1]. There is an important economic impact that is a result of the incapacity cause by such diseases, as well as the treatment-related costs. Arterial hypertension has been gaining some relevance, due to its prevalence, and because its epidemiological base studies reveal a lack of control sample. Considering this problematic, a study was conducted, that analyses the mental adjustment of patients with arterial hypertension and the impact of the family nurse practitioner during appointments for hypertension monitoring.

**Objective**

The objective was to evaluate the impact of the family nurse practitioner on the mental adjustment of patients that suffer from arterial hypertension, registered at the “HTA” program of the Personalised Healthcare Unit of Healthcare Centre of Sever do Vouga.

**Methods**

A quantitative study was conducted, meeting one of the cycles of the research-action method, since an initial analysis was conducted, followed by the implementation of the intervention, and then carried by a new evaluation. Thus, using the Mental Adjustment to Disease Scale, as an evaluation instrument, regarding mental adjustment, along with a sociodemographic and clinical characterization questionnaire, addressed to the participants. The ethical principles were followed during the entire course of the investigation.

**Results**

The participants in this study had and average age of 70.8 years, and being mostly females, diagnosed with for 8.4 years. To evaluate the internal consistency of MADS, it was calculated the Cronbach Alpha in moments 1 and 2, obtaining acceptable results, except for the subscale regarding fighting spirit. At moment 1 of the data collection, in the subscale regarding fighting spirit, all participants were classified as “fitted”, however for the remaining subscales, the participants were classified as “fitted” and “Not fitted”. The results obtained at moment 2, have revealed the impact from the conducted intervention, since the participants initially classified as “not fitted”, shifted to “fitted” at the 2nd moment.

**Conclusions**

Performing a balance of the internship, it can be claimed that the expected competences and objectives were achieved. Ultimately, we can withdraw the conclusion that the intervention developed for the mental adjustment obtained the expected results, since the participants classified as “not fitted” on the 1st moment, were classified as “fitted” during the 2nd assessment moments.

**References**

1. Trindade, I. D. (2016). Análise Pragmética da Comorbilidade Associada a Doentes com Hipertensão Arterial em Cuidados de Saúde Primários. Revista Portuguesa de Hipertensão E Risco Cardiocascular, 51, 40.

**Keywords**

Mental adjustment, Nursing Family, Arterial Hypertension.

### O57 Family conferences – the two year experience of a palliative care support team (PCST) in a tertiary hospital

#### Júlia Alves, Joana Mirra, Rita F Soares, Margarida Santos, Isabel Barbedo, Sara Silva, Elga Freire

##### Equipa Intra-hospitalar de Suporte em Cuidados Paliativos, Departamento de Medicina Interna, Centro Hospitalar do Porto, 4099-001 Porto, Portugal

###### **Correspondence:** Júlia Alves (juliasousaalves@gmail.com)

**Background**

Family conference (FC) is an important work that represents an opportunity to evaluate family dynamics, provide anticipatory care and support feelings related to the loss of a loved one [1]. FC facilitates the communication between healthcare providers, patient and family and allows the discussion of different options and summarize consensus with the ultimate goal of problem solving, decision making and instituting a plan [2].

**Objective**

Describe the experience of FCs made by a PCST from 1 of January 2015 to 31 of December 2016.

**Methods**

Raw data from FCs registers was retrieved and a descriptive analysis was performed.

**Results**

We consulted 809 patients and 431 FCs were held (81% scheduled). In 56 FC the patient was present; when the patient was absent, most cases were due to clinical condition and in a minority the patient chose not to be present. Family members attending FCs were offspring in 67%, spouses in 23%, other relatives in 38% and the parents in 4% of cases. All meetings occurred in the presence of one physician and one nurse from the PCST. FCs were held because of patient discharge (88%), worsening of clinical condition (24%), family needs (59%), discussion of therapeutic goals (38%) and conspiracy of silence (1%). As a consultant team, the PCST is concerned with post-discharge and evaluates the needs of patients at home with the help of an ambulatory healthcare team or primary care team. Therefore, FCs have the objective of preparing families for patient discharge. During FC the main subjects of discussion were post-discharge healthcare referral (94%), objectives of healthcare (84%), clinical information about diagnosis and prognosis (42%), symptom control (47%), management of expectations concerning the illness (73%), nutrition (12%) and family needs (psychological support in 17% and nursing instructions 10%).

**Conclusions**

There is an increasing number of FC and more are being requested by the referral healthcare team. To facilitate the registry of FC a document was elaborated and soon it will be made a software. This registry will allow an easier analysis and strategy planning to improve interventions and healthcare quality provided.

**References**

1. Neto I. A conferência familiar como instrumento de apoio à família em cuidados paliativos. Revista Portuguesa de Clínica Geral. Vol.19 (2003), p.68-74.

2. Barbosa A, et al. Manual de Cuidados Paliativos. 2ªed. Lisboa (2010): Núcleo de Cuidados Paliativos, Centro de Bioética, Faculdade de Medicina da Universidade de Lisboa.

**Keywords**

Palliative care, Family, Health.

### O58 Diabetes mellitus and polypharmacy in elderly population: what is the reality?

#### Claudia Oliveira^1^, Helena José^2,3^, Alexandre C Caldas^1^

##### ^1^Institute of Health Sciences, Universidade Católica Portuguesa, 1649-023 Lisbon, Portugal; ^2^Health Sciences Research Unit: Nursing, Nursing School of Coimbra, 3046- 851 Coimbra, Portugal; ^3^Centro de Formação de Saúde Multiperfil, Luanda, Angola

###### **Correspondence:** Claudia Oliveira (claudiajs.oliveira@gmail.com)

**Background**

Diabetes mellitus is a prevalent disease among the elderly and is listed as one of the leading causes of admissions and readmission [1]. Older people with diabetes represent a challenge, in terms of effective coordination and management in multiple areas. In this sense, older population needs to adhere to a medication regimen, sometimes complex. Polypharmacy is a reality and leads to unnecessary disease progression and complications, reduces functional abilities, increases hospitalizations, reduces the quality of life, increases health costs and even deaths [2]. Management of such phenomenon is extremely hard and requires awareness.

**Objective**

To identify the clinical profile of the older people with Diabetes mellitus in two Family Health Units in Faro (FHUs Farol and Al-Gharb).

**Methods**

Observational and descriptive study was performed, with people aged 65 years or above, living in the community and registered at the Health Centre of Faro (FHUs Farol and Al-Gharb). Three hundred and ninety-five patients were interview in terms of their medication regimen. For data collection, a sociodemographic questionnaire, Medication Regimen Complexity Index (MRCI) and chemical parameters (glycated haemoglobin (HbA1C) and capillary glycaemia) were used.

**Results**

The sample was composed of people aged 65 years and over [75.59 (±6.75)], with a maximum of 93 years (52.9% were women and 47.1% were men). Regarding the MRCI, an average of 15.63 (± 6.84), with a minimum of 5 and a maximum of 32 was found. We verified the existence of a high and statistically significant positive correlation (r = .897; p-value <.001) between the MRCI and the number of drugs prescribed. The study also showed that the increase of the number of drugs prescribed is related to advanced age. For HbA1C, an average of 7.09 (± 1.14), a minimum of 5.3 and a maximum of 12.4 was obtained. It was found that 57.47% of the patients had HbA1C value lower than 7%, 22.78% had values between 7%-7.9% and 19.75% had values higher than 7.9%. In relation to capillary glycaemia, we obtained a mean of 181.13 (± 66.54) with a minimum of 83 and a maximum of 500.

**Conclusions**

Medication non-adherence and polypharmacy are real problems with negative impact, and potentially fatal. High numbers of medications prescribed are nowadays more common. Unfortunately, the elevated rates of HbA1C and capillary glycaemia values demonstrate that disease management is not effective, so it is urgent to implement programs to help older people self-manage chronic condition.

**References**

1. Kirkman MS, Briscoe VJ, Clark N et al. Diabetes in Older Adults. Diabetes Care. 2012; 35(12): 2650-64.

2. Masnoon N, Shakib S, Kalisch-Ellet L, Caughey G. What is polypharmacy? A systematic review of definitions BMC Geriatrics. 2017; 17: 230.

**Keywords**

Medications Adherence, Patients, Aged, Polypharmacy, Diabetes Mellitus.

### O59 Nurses' perception of Computerized Information Systems impact on the global nurses’ workload

#### Paulino Sousa^1^, Marisa Bailas^2^

##### ^1^Centro de Investigação em Tecnologias e Serviços de Saúde, 4200-450 Porto, Portugal^; 2^Centro Hospitalar de São João, 4200-319 Porto, Portugal

###### **Correspondence:** Paulino Sousa (paulino@esenf.pt)

**Background**

Portugal has a story of almost two decades of computerized information systems use on health area, particularly in the nursing area, with the large-scale implementation of Computerized Information Systems to support nursing practice. We know that the use of electronic health information to support patient care will undoubtedly be responsible for a substantial time-spent on the overall workload of nurses. Too often, we are confronted with nurses’ opinions that the use of computerized information systems (CIS) has a great impact on the overall nurses’ workload (35 to 50% of the global nurses’ workload).

**Objective**

To identify the perception of nurses on the time spent on CIS in use in a hospital and his impact on the global nurses’ workload.

**Methods**

A cross-sectional survey was applied to collect data from 148 nurses that use CIS in a hospital (medical and surgical services). This allowed knowing the average percentage of nurses’ perception time spent on the use of SClinico® and other information supports, as well as their distribution by a set of nursing activities in use of the system.

**Results**

The results showed that nurses consider that time spent on information supports has an average of 42.4% on their total working time: 33.5% on the use of SClínico® (mode and median of 30%; SD ±16.25) and 8.9 % (mode and median of 5 %; SD ±6.67) on nursing records in other non-computerized structures (particularly on paper). These values are overlapping to those presented in some national and international studies. However, results are higher than the real-time shown in studies of Silva (2001) and Sousa and colleagues (2015). Nurses who underwent training processes on Nursing Information System in use (SClínico®) and on ICNP® have differences in time-spent perception on the use of CIS.

**Conclusions**

A permanent issue in the debate on the use of CIS is the time spent in its use, in particular in the processes of data access, care planning, and record keeping. Nurses have the perception that the time spent on CIS has, in fact, an essential part of nursing practice, but with a high impact on the workload of nurses. However, there are several national and international studies that point out in certain contexts for a lower “real-time” in the global nurses’ workload on the use of computerized information systems.

**Keywords**

Computerized Information Systems, Electronic health information, Nursing workload, Time spent.

### O60 Pain in people 75 and older: association with activity patterns

#### Maria C Rocha, José G Sousa, Madalena G Silva

##### Physiotherapy Department, School of Health, Polytechnic Institute of Setúbal, 2910-761 Setúbal, Portugal

###### **Correspondence:** Madalena G Silva (madalena.gomesdasilva@gmail.com)

**Background**

The prevalence of pain amongst the elderly population (19.5% [1] to 52.8%[2]) may not be disregarded and varies depending on the age range and context. Regardless, pain in this population group has been associated with reduced functional capacity, changes in gait and sleep patterns, depression and reduced social participation [3]. Exercise has often been recommended as an intervention to manage pain in the elderly [4], however long-term adherence to exercise programs is limited [5]. Characterization of pain and exploring its associations with light intensity physical activity may provide a base for discussing alternative clinical interventions for the management of pain in this population group.

**Objective**

To characterize the presence, location and duration of pain in very old adults and investigate its association with light intensity physical activity.

**Methods**

A cross-sectional study was implemented with 65 participants aged above 75 years, without cognitive impairment, average age of 79.48 ± 4.98. Presence, location and duration of pain were assessed with the socio-demographic and clinical characterization questionnaire. Light intensity activity was characterized with an Activity Diary. Given de non-normal distribution, dichotomic nominal variables were analysed with the biserial point correlation, and Spearmen’s rho was used for the remaining.

**Results**

Two thirds (61.5%) of our sample was female, with a low educational level (64.6%). Eighty three percent (n = 54) reported experiencing pain, and from these, 45 (83.3%) had pain for more than one year. Pain was mainly localized in the knees (n = 30) and in the lower back (n = 27). Our sample spent an average of 5h46min per day in sedentary behaviour (< 1.5 METs) and 4h47min in physical activity of light intensity (> 1.5 and < 3 METs, Metabolic Equivalents). Physical activity of low intensity showed a non-significant association with the presence of pain (p = 0.622) nor with the duration of pain (p = 0.525).

**Conclusions**

We conclude that our sample had a very high prevalence of pain for more than one year, and that this is not associated with the time spent in light intensity physical activity. Further studies are required to provide a better understanding of the association of specific types and location of pain and light intensity physical activity, before it can be promoted as a clinical intervention strategy.

**References**

1. Satghare P, Chong SA, Vaingankar J, Picco L, Abdin E, Chua BY, et al. Prevalence and correlates of pain in people aged 60 years and above in Singapore: Results from the wise study. Pain Res Manag. 2016;2016.

2. Pereira LV, Vasconcelos PP de, Souza LAF, Pereira G de A, Nakatani AYK, Bachion MM. Prevalence and intensity of chronic pain and self-perceived health among elderly people: a population-based study. Rev Lat Am Enfermagem. 2014;22(4):662–9.

3. Herr K. Pain assessment strategies in older patients. J Pain. 2011;12(3 SUPPL.):S3–13.

4. Tse MM, Vong SK, Tang SK. Motivational interviewing and exercise programme for community-dwelling older persons with chronic pain: A randomised controlled study. J Clin Nurs. 2013;22(13–14):1843–56.

5. Shubert TE, Goto LS, Smith ML, Jiang L, Rudman H, Ory MG. The Otago Exercise Program: Innovative Delivery Models to Maximize Sustained Outcomes for High Risk, Homebound Older Adults. Front Public Heal. 2017;5.

**Keywords**

Pain, Activity, Older adults.

### O61 Impact of the people with intellectual disability and proxies’ characteristics on quality of life assessment

#### Cristina Simões^1,2^, Sofia Santos^2,3^

##### ^1^Economics and Social Sciences Department, Portuguese Catholic University, 3504-505 Viseu, Portugal; ^2^Study Center for Special Education, Faculdade de Motricidade Humana, University of Lisbon, 1499-002 Cruz Quebrada, Portugal; ^3^Unidade de Investigação e Desenvolvimento em Educação e Formação, Instituto de Educação, University of Lisbon, 1649-013 Lisbon, Portugal

###### **Correspondence:** Cristina Simões (cristina-ferreira@iol.pt)

**Background**

The quality of life (QoL) assessment should include self-report measures in the field of intellectual disability (ID), which provide useful information for personalized support plans and give those with ID the opportunity to express their own perspectives regarding themselves and their individual contexts of life. Nevertheless, the communication and understanding limitations of people with ID can be a barrier to obtaining self-report perceptions. The inclusion of a proxy who knows the individual with ID well has been used to overcome the difficulties of the subjective assessments.

**Objective**

This proposal aims to explore the factors that could potentially explain the disagreements in QoL assessment of people with ID and their proxies.

**Methods**

Data were collected from 207 participants: 69 people with ID, 69 practitioners and 69 family members. QoL was assessed by the Portuguese version of the Personal Outcomes Scale. Paired-sample t tests were performed to examine the differences between the mean scores. Multiple regressions were calculated to analyse the determinants that could explain the directional mean difference between people with ID, support staff, and family members.

**Results**

The personal and environmental characteristics of people with ID (gender, diagnosis, living circumstances, and type of transportation) and the characteristics of practitioners (age, education level, relationship, health status of the person with the ID) had scores with a medium explanation of the disagreements between those participants. The education level of support staff and the health status of the person with the ID had largely explained the discrepancies between people with ID and key workers. Furthermore, the results revealed that four characteristics were major predictors of disagreement between people with ID and family members: the age of the person with ID, the type of transportation, the self-reported health status of the person with ID, and the health status of the person with the ID, as assessed by the family members. Finally, robust factors seemed to explain the discrepancies between practitioners and family members: living circumstances, self-reported health status of the person with the ID, education level of the key worker and education level of family members.

**Conclusions**

Among other factors, the health status was a major predictor of the different perceptions on QoL assessment. Findings showed that it was possible to predict differences among the three groups of respondents. Strictly speaking, the personal and environmental characteristics of people with ID and proxies predicted the disagreement among the participants.

**Keywords**

Quality of life, Intellectual disability, Self-report, Proxies, Predictors.

### O62 Factors that influence the decision on how and when to use a “health kiosk”

#### João Rodrigues^1,4^, Paulino Sousa^2^, Pedro Brandão^3,4^

##### ^1^Administração Regional de Saúde do Norte, 4000-099 Porto, Portugal; ^2^Centro de Investigação em Tecnologias e Serviços de Saúde, 4200-450 Porto, Portugal; ^3^Faculdade de Ciências, Universidade do Porto, 4169-007 Porto, Portugal; ^4^Instituto de Telecomunicações, 1049-001 Lisboa, Portugal

###### **Correspondence:** João Rodrigues (joaorodrigues006@gmail.com)

**Background**

Health kiosks have been recognized as an effective way to develop knowledge and capabilities of citizens, which can improve the promotion of a healthy behaviour. But several have been the problems that have hindered the large-scale implementation and use of health kiosks, one of the most prominent being the limited acceptance of technology by citizens.

**Objective**

To identify factors that influence the decision on how and when to use a health kiosk

**Methods**

This kiosk appears as an innovative project, allowing to monitor anthropometric data (weight) and vital signs (heart rate, oximetry, and blood pressure) on a routine basis or prior to a medical appointment. This was an exploratory study, descriptive and correlational, of a cross-sectional study, with a mixed approach (quantitative and qualitative). In the elaboration of the instruments of data collection, we based on the Technological Acceptance Model (TAM). The analysis of the factors influencing the decision on how and when to use the “health check” was supported by the constructs: perceived utility, perceived ease of use, perceived credibility, and perceived knowledge.

**Results**

92 citizens accepted to participate in the study. But, 34 refused to use the kiosk (justifying their refusal): they considered “*not having enough time to use the kiosk*”, most of which verbalized that “*they were afraid of losing their medical appointment if they did not hear the call*”, “*not feeling able to use it*”, “*not being able to use computers*” or “*not associating any utility in its use*”. The kiosk was used by 58 people who had come to the Health Centre with different objectives: nursing appointment (41.4%), medical consultation (36.2%), administrative contact (13.8%) and the remaining were companions of other health care users (5.6%). Participants were mostly female (70.7%), with an average of 51.3 years (median 51.4, SD±17.6). They reported using technological devices: 94.8% used mobile phones (62.1% has “smartphones”) and 60.3% use computers. Only one participant had experienced prior “health kiosk” use. Users appreciated the utility (94.1%) and easy use (85.7%), as well as the credibility (94.6%) of the kiosk. The perceived knowledge was considered by 80.4% of participants as very good (5.4%) or good (75.0%).

**Conclusions**

TAM was crucial to understand the strength that some of its dimensions may have as factors that influence the decision on how and when to use the “health kiosk”. Among the citizens who used the health kiosk, mostly found it useful, easy to use, credible and secure.

**Acknowledgements**

This article is a result of the project NanoSTIMA Macro-to-Nano Human Sensing: Towards Integrated Multimodal Health Monitoring and Analytics, Norte-01-0145-FEDER-000016, supported by Norte Portugal Regional Operational Programme (NORTE 2020), through Portugal 2020 and the European Development Fund.

**Keywords**

Health kiosk, Technological Acceptance Mode, Monitoring.

### O63 Delirium care: a survey into nursing perceptions and knowledge

#### Marta Bento^1^, Rita Marques^2^

##### ^1^Universidade Católica Portuguesa, 1649-023 Lisbon, Portugal^; 2^Hospital de Santa Maria, Centro Hospitalar Lisboa Norte, 1649-035 Lisbon, Portugal

###### **Correspondence:** Marta Bento (marsofia81@hotmail.com)

**Background**

Delirium is a reversible cognitive manifestation of sudden onset, developing in a matter of hours or days; characterized by a fluctuating course of disturbed attention, memory and perception [1]. Although common, this syndrome is often under-diagnosed and nursing staff are in the best position to recognize, prevent and monitoring delirium symptoms. The current approach to delirium care seems to be insufficient and nurses need to receive more support and guidance providing high quality care [2]. The education of nurses in all care settings can provide the foundation to address this massive international challenge.

**Objective**

The aim of the study is to assess nursing knowledge, in order to understand and perceive delirious adult/elderly patients.

**Methods**

In this exploratory study, we applied a questionnaire with closed questions and the sample consisted of 49 nurses working at an ER of a Central Hospital at Lisbon, during the month of December 2017. In order to safeguard ethical issues, we requested approval and informed consent to all participants in the study, with the anonymity and confidentiality of the data being ensured.

**Results**

The data yielded revealed that there was a high level of knowledge on the definition of delirium (93.8%) and also on the application of the Confusion Assessment Method (86.4%), although in this unit this instrument is not applied routinely. The analysis also reveals that there is a very high level of knowledge about the characteristics of a delirious patients and 100% of the nursing Staff recognize these patients, has not always aggressive. Furthermore, the dehydration and the poor nutrition were identified has risk factors for delirium (95.8% and 91.8%, respectively). On the contrary 63.3% (n = 31) of the respondents assumed that a patient with impaired vision isn’t at increased risk of delirium or neither 22.4% (n = 11) the risk for delirium increases with age 22.4% (n = 11). Equally important, 28.6% (n = 14) of the respondents did not know that patients with delirium present higher mortality rates.

**Conclusions**

Despite the literature assumes in same hospital settings nurses have insufficient knowledge of delirium-related information, the results of this study evidence an overall positively answered mean score, showing a high level of knowledge of delirium and its risk factors. Nurses have a key role to accurately recognizing and caring for delirious patients given the poor outcomes of untreated delirium.

**References**

1. American psychiatric association. Diagnostic and Statistical Manual of Mental Disorders, DSM-5. Fifth Edition. Artmed; 2014. 976 p.

2. Zamoscik K, Godbold R, Freeman P. Intensive care nurses’ experiences and perceptions of delirium and delirium care. Intensive Crit Care Nurs. Junho de 2017;40:94–100.

**Keywords**

Delirium, Nursing, Knowledge, Risk factors.

### O64 Nurses' satisfaction with the use of Health Information System in Funchal hospitals

#### Plácida Silva^1^, Paulino Sousa^2^, Élvio Jesus^1^

##### ^1^Hospital Dr. Nélio Mendonça, 9004-514 Funchal, Portugal; ^2^Centro de Investigação em Tecnologias e Serviços de Saúde, 4200-450 Porto, Portugal

###### **Correspondence:** Plácida Silva (placidasilva@hotmail.com)

**Background**

The evaluation of the Health Information System (HIS) is a fundamental activity to determine the success of the system and guarantee the continuity of its use. That is why it is important to know the true impact on the use and satisfaction of its users. In recent years, we have seen in Portugal different studies on nurses’ satisfaction with HIS use. However, none of the studies refers user satisfaction with the HIS structure that supports the practice of nurses in the Autonomous Region of Madeira (ARM).

**Objective**

To identify dimensions and level of nurses' satisfaction with the HIS in use.

**Methods**

A cross-sectional, exploratory and descriptive study was carried out in the ARM, in inpatient units of Funchal Hospitals. Data collection was supported by the application of the “User Satisfaction Questionnaire for Nursing Information Systems” based on DeLone & McLean Model of Information System Success (2003). This instrument uses a 5-point Likert scale structure with a semantic differential operationalized “*1-unsatisfied*” and “*5-very satisfied*”, in an increasing logic of level of satisfaction, in which there is no neutral intermediate point.

**Results**

The adherence rate of the study population was 50.5%, corresponding to a sample of 283 nurses. The exploratory factor analysis process was reduced to 5 factors, similar to previous studies which resulted in the following dimensions: 1) information sharing; 2) structure and content of information needed for decision-making; 3) support structures and HIS contributions; 4) Security, data protection and technical support; and 5) graphical data presentation. The dimension “*satisfaction with access to necessary information for decision making*” with an average value of 3.13 (Median 3, SD ±0.70), reports the area where the higher level of satisfaction is observed. The dimension “*support structures and HIS contributions*” has the lower average value of 2.81 (Median 2.8, SD ±0.64), The overall “nurses’ satisfaction with NIS in use” was 2.96 (±0.57), with a median of 3 on a Likert scale. This overall result reports a good level of nurses’ satisfaction with the HIS that they use.

**Conclusions**

This study allowed us to identify dimensions that incorporate the DeLone & McLean Model of Information System Success. At the same time, it has allowed us to identify factors that determine the level of satisfaction of the nurses with the HIS that they use and being able to determine the “use” and “intention to use them”.

**Keywords**

Health Information Systems, Nurses, Satisfaction, Evaluation.

### O65 How prevalent are psycoactive substances among health students?

#### Sandra Ventura^1^, André RTS Araujo^1^, João Leitão1, Odília D Cavaco^1^, Rui Correia^2^, Maria J. Nunes^2^

##### ^1^Escola Superior de Saúde, Instituto Politécnico da Guarda, 6300-749 Guarda, Portugal; ^2^Centro de Respostas Integradas da Guarda, Administração Regional de Saúde do Centro, 6300-725 Guarda, Portugal

###### **Correspondence:** Sandra Ventura (scventura@ipg.pt)

**Background**

Consumption of psychoactive substances is widespread among young adolescents and young adults and constitutes a public health problem with significant consequences for individuals and societies throughout the world. The main consequences depend on the consumption pattern of the substance used and may result from the immediate or cumulative toxic effect of the substance consumed, from intoxication or psychoactive effects, or from addiction or addiction syndrome. Particularly alcohol and tobacco consumptions are the second and third risk factors of morbidity and mortality in Europe. Harmful use of alcohol, under acute and chronic conditions, can have serious developmental and social consequences, including violence, neglect and accidents, as well as health problems. Tobacco use also has negative consequences on health and social life.

**Objective**

In this context, the objective of this study was to characterize the consumption of psychoactive substances by students of the Superior Health School of the Polytechnic Institute of Guarda and to reflect on prevention strategies to be implemented to dissuade and reduce consumption among students.

**Methods**

A questionnaire was applied to the students and we collected 261 answers, from a total of 175 students of the Nursing Course and 88 of the Pharmacy Course.

**Results**

The results obtained indicate that the most consumed substances were, in descending order: alcohol, tobacco, psychoactive drugs and illicit substances. Regarding alcohol experimentation, it was found that 76.6% of nursing students and 85.2% of pharmacy students had already consumed alcohol. These data were higher than the national prevalence throughout life in 2015 (71%). The consumption of alcohol by nursing students (74.3%) and by pharmacy students (89.8%) was also higher than the consumption of Portuguese young people between 13 and 18 years old (62%). Tobacco consumption by nursing and pharmacy students were of 51.6% and 56.8%, respectively, both higher than the national data of 40%. The consumption of tobacco was of 39.3% by nursing students and 45.5% by pharmacy students, and it was also higher than the 30% reported for national consumption. Illicit substances were the least consumed, with a prevalence throughout life of 13.3% and 9.0%, respectively, by nursing and pharmacy students, inferior than the national data (19%). Cannabis was the illicit substance more consumed by either nursing and pharmacy students.

**Conclusions**

These results indicate that there is work to be done in the prevention and dissuasion of consumption of psychoactive substances by our student community.

**Keywords**

Psychoactive substances, Consumption, Prevalence, Prevention, Dissuassion.

### O66 Diabetes Mellitus as a key indirect causal factor for pressure ulcer development

#### Pedro Sardo^1,2^, Jenifer Guedes^2^, José Alvarelhão^1^, Elsa Melo^1^

##### ^1^School of Health Sciences, University of Aveiro, 3810-193 Aveiro, Portugal; ^2^Centro Hospitalar do Baixo Vouga, 3810-501 Aveiro, Portugal

###### **Correspondence:** Pedro Sardo (pedro.sardo@ua.pt)

**Background**

Ensuring patient safety in healthcare is a challenge [1-6]. With the growing *Diabetes Mellitus* incidence, healthcare professionals and planners are encouraged to pay further attention to the major complications of this disorder [3]. According to EPUAP and EWMA [3], poor circulation and infection are among the most common complications that effect diabetic patients. A recent pressure ulcer conceptual framework [7, 8] identified *Diabetes Mellitus* as a key indirect causal factor (and poor perfusion as a direct causal factor) for pressure ulcer development and encourages the development of clinical studies that explore the correlation(s) between these specific risk factors and pressure ulcer development.

**Objective**

To identify the influence of *Diabetes Mellitus* on pressure ulcer development in adult patients admitted to medical and surgical wards in 3 Portuguese hospitals.

**Methods**

Cross sectional design survey developed on June 16th, 2015 with 236 adult patients admitted to medical and surgical wards in 3 Portuguese hospitals. The study was performed after Hospital Council Board and Ethics Committee approval (Reference Number 049688). Data were analysed using SPSS v25.0. Descriptive statistics were calculated for the sample characterization. Pressure ulcer risk, prevalence and incidence were calculated according to EPUAP statement [9]. Odds ratio (OR) was calculated by univariate logistic regression.

**Results**

This study included a sample of 236 participants with the median age of 76 years (Q1 = 62 years; Q3 = 83 years). The majority of the participants was male (56.8%), admitted trough the emergency service (80.9%) and stayed in medical units (60.2%). On the day of the survey, 121 (51.3%) participants were classified as “*high risk of pressure ulcer development*” (Braden Scale score ≤ 16); 45 (19.1%) participants had at least one pressure ulcer documented; 7 (3.0%) participants developed a new pressure ulcer since the admission in inpatient setting; and 67 (28.4%) participants had *Diabetes Mellitus*. Using a univariate logistic regression model, the odds of developing a pressure ulcer during the length of inpatient stay were significantly higher for the participants with *Diabetes Mellitus* with OR = 6.73 (95% CI:1.27-35.61, Nagelkerke R^2^ = 0.103) compared to the other participants.

**Conclusions**

This study supports the pressure ulcer conceptual framework proposed by Coleman, Nelson [7] and Coleman, Nixon [8], showing that *Diabetes Mellitus* is a key (indirect) causal factor for pressure ulcer development in inpatient settings. However, further studies are needed in order to understand the influence of *Diabetes Mellitus* on skin and tissue (poor) perfusion and consequently on pressure ulcer development.

**Acknowledgements**

Thanks are due to “Centro Hospitalar Baixo Vouga, EPE” (Portugal), particularly to the Nursing Council Board, head nurses and to the nurses that recorded the data in the medical and surgical services of Águeda Hospital, Aveiro Hospital and Estarreja Hospital.

**References**

1. EPUAP, EWMA. Patient safety across Europe: the perspective of pressure ulcers.2017.

2. EPUAP, EWMA. The time to invest in patient safety and pressure ulcer prevention is now! 2017.

3. EPUAP, EWMA. Diabetic Control & Pressure Ulcers: fighting fatal complications and improving quality of life. 2017.

4. Sardo P, Simões C, Alvarelhão J, Costa C, Simões CJ, Figueira J, et al. Pressure ulcer risk assessment: retrospective analysis of Braden Scale scores in Portuguese hospitalised adult patients. Journal of Clinical Nursing. 2015;24(21-22):3165-76.

5. Sardo P, Simões C, Alvarelhão J, Costa C, Simões CJ, Figueira J, et al. Analyses of pressure ulcer point prevalence at the first skin assessment in a Portuguese hospital. Journal of Tissue Viability. 2016;25(2):75-82.

6. Sardo P, Simões C, Alvarelhão J, Simões JL, Machado P, Amado F, et al. Analyses of pressure ulcer incidence in inpatient setting in a Portuguese hospital. Journal of Tissue Viability. 2016;25(4):209-15.

7. Coleman S, Nelson EA, Keen J, Wilson L, McGinnis E, Dealey C, et al. Developing a pressure ulcer risk factor minimum data set and risk assessment framework. J Adv Nurs. 2014;70(10):2339-52.

8. Coleman S, Nixon J, Keen J, Wilson L, McGinnis E, Dealey C, et al. A new pressure ulcer conceptual framework. J Adv Nurs. 2014;70(10):2222-34.

**Keywords**

Diabetes Mellitus, Nursing Assessment, Portugal, Pressure Ulcer, Risk Assessment

### O67 Frailty syndrome in the elderly hospitalized in a teaching hospital

#### Luciane Cabral, Clóris Regina, Bruno A Condas, Péricles Reche, Danielle Bordim, Jacy Sousa

##### Departamento de Enfermagem e Saúde Pública, Universidade Estadual de Ponta Grossa, 4748 Ponta Grossa, Paraná, Brasil

###### **Correspondence:** Luciane Cabral (luciane.pacabral@gmail.com)

**Background**

In Brazil and in the world, the growth of the elderly population is an indisputable reality, so it is necessary to identify the factors that favour the sickness of this age group, with emphasis on the fragility, which can be defined as a syndrome which presents innumerable causes and is characterized by a set of clinical manifestations, such as decreased in strength, endurance and physiological function, collaborating to make the individual more vulnerable to addiction and/or death [1].

**Objective**

In view of the above, the present study aimed to evaluate the fragility syndrome of the elderly hospitalized in a teaching hospital.

**Methods**

A cross-sectional study, carried out with a convenience sample of 107 elderly patients admitted to the emergency room, at the medical, surgical and neurology clinic of the medical, surgical and neurology of a teaching hospital in the Campos Gerais region, from October 2016 to April 2017. Data collection included the application of the Mini Mental State Examination [2] for cognitive screening and Edmonton Fragility Scale [3], culturally adapted to the Portuguese language in Brazil [4]. Data were analysed using Stata®12 software. The association was verified through simple linear regression (Fisher's F and Student's t tests), significance level of p = 0.05. The project was approved by the Ethics Committee of the State University of Ponta Grossa (CAAE nº 34905214.0.0000.0105).

**Results**

The results showed a predominance of females (58.9%), married (61.0%), low schooling (71.0%), living with spouse (n = 42, 39.3%), considered their income satisfactory (50.5%). The mean age of participants was 70.3 years. Regarding clinical variables, 99.1% had a disease, 36.5% used medication and 50.5% reported hospitalization in the last 12 months. The fragility evaluation identified that 19.6% of the elderly were nonfragile, 24.3% apparently vulnerable, 26.2% had mild fragility, 15.9% moderate and 14.0% severe. It was found that any level of schooling used medication (p = 0.001), solitude (p = 0.001), loss of urine (p = 0.001) and hospitalization in the last 12 months (p = 0.001) and was associated with the fragility syndrome.

**Conclusions**

The importance of early detection of the syndrome is emphasized through the use of an instrument that is valid, reliable and easy to apply by the health team, such as the Edmonton Fragility Scale. The results presented can support the planning of health care, considering the characteristics and demands of the elderly who are hospitalized, thus contributing to improve the quality of care provided.

**References**

1. Morley JE, Vellas B, Kan GAV, Anker SD, Bauer JM, Bernabei R, et al. Frailty consensus: a call to action. JAMDA. 2013, 14(6):392-7.

2. Folstein MF, Folstein SE, McHugh PR. “Mini-mental state”: a practical method for granding the cognitive state of patients for the clinican. J PsychiatrRes. 1975, 12(3):189-98.

3. Rolfson D, Majumdar S, Tsuyuki R, Tahir A , Rockwood K. Validity and reliability of the Edmonton Frail Scale. Age Ageing. 2006, 35(5):526-9.

4. Fabrício-Wehbe SCC, Cruz IR, Haas VJ, Diniz MA, Dantas RAS, Rodrigues RAP. Reproducibility of the Brazilian version of the Edmonton Frail Scale for elderly living in the community. Rev Latino-Am Enfermagem. 2013; 21(6):1330-6.

**Keywords**

Aged, Frail Elderly, Geriatric Nursing.

### O68 Braden Scale accuracy tests

#### Pedro Sardo^1,2^, Jenifer Guedes^2^, José Alvarelhão^1^, Elsa Melo^1^

##### ^1^School of Health Sciences, University of Aveiro, 3810-193 Aveiro, Portugal; ^2^Centro Hospitalar do Baixo Vouga, 3810-501 Aveiro, Portugal

###### **Correspondence:** Pedro Sardo (pedro.sardo@ua.pt)

**Background**

Pressure ulcers management is a challenge [1-6]. Portuguese guidelines [7] encourage the implementation of regular pressure ulcer risk assessments through the application of the Braden Scale and the patients’ categorisation into two levels of risk (defined by cut-off point of 16). However, the development of pressure ulcer(s) is complex and multifactorial [8] and whenever the Braden Scale score falls below 18, each patient functional deficit and/or risk factor should be individually addressed [9].

**Objective**

To analyse the Braden Scale accuracy tests in adult patients admitted to general wards in a Portuguese hospital during one year, using different cut-off points.

**Methods**

The study was designed as a retrospective cohort analysis of electronic health record database from 6,552 adult patients admitted without any pressure ulcer in medical and surgical wards in a Portuguese hospital during 2012. All data were extracted after Hospital Council Board and Ethics Committee approval (Reference Number 049688). Braden Scale Accuracy Tests (BSAT) such as sensitivity, specificity, positive predictive value (PPV), negative predictive value (NPV), and the area under the curve (AUC) were assessed [10].

**Results**

The study included 6,552 participants with a mean age of 64 years and 6 months. The majority of participants was male (52.6%); admitted trough emergency service (69.1%); in surgical (64.5%) units. During the length of stay 153 (2.3%) participants developed (at least) one pressure ulcer. Considering the cut-off point of 15, the BSAT showed: sensitivity of 50%(95%CI:42%-58%); specificity of 82%(95%CI:81%-83%); PPV of 6%(95%CI:5%-8%); NPV 99%(95%CI:98%-99%); and AUC of 66%(95%CI:61%-71%). Considering the cut-off point of 16, the BSAT showed: sensitivity of 63%(95%CI:55%-71%); specificity of 74%(95%CI:73%-75%); PPV of 5%(95%CI:4%-7%); NPV 99%(95%CI:98%-99%); and AUC of 69%(95%CI:64%-73%). Considering the cut-off point of 17 the BSAT showed: sensitivity of 78%(95%CI:71%-84%); specificity of 63%(95%CI:61%-64%); PPV of 5%(95%CI:4%-6%); NPV 99%(95%CI:99%-99%); and AUC of 71%(95%CI:67%-74%).

**Conclusions**

Although our BSATs follow the trend of the results found in a recent systematic review [11], they showed some of the limitations of the patients’ categorisation into two levels of risk according to a specific cut-off value. Like Braden [9], we believe that this assessment tool should be supplied with clinical judgment in order to identify patients’ specific risk factors that should be individually addressed with accurate preventive interventions. Furthermore, in order to develop evidence-based practice, we should create a minimum data set for pressure ulcer prevention, assessment and documentation [12-14] based on patients’ characteristics, international guidelines [15] and conceptual frameworks [12-14].

**References**

1. EPUAP, EWMA. Patient safety across Europe: the perspective of pressure ulcers. 2017.

2. EPUAP, EWMA. The time to invest in patient safety and pressure ulcer prevention is now! 2017.

3. EPUAP, EWMA. Diabetic Control & Pressure Ulcers: fighting fatal complications and improving quality of life. 2017.

4. Sardo P, Simões C, Alvarelhão J, Costa C, Simões CJ, Figueira J, et al. Pressure ulcer risk assessment: retrospective analysis of Braden Scale scores in Portuguese hospitalised adult patients. Journal of Clinical Nursing. 2015;24(21-22):3165-76.

5. Sardo P, Simões C, Alvarelhão J, Costa C, Simões CJ, Figueira J, et al. Analyses of pressure ulcer point prevalence at the first skin assessment in a Portuguese hospital. Journal of Tissue Viability. 2016;25(2):75-82.

6. Sardo P, Simões C, Alvarelhão J, Simões JL, Machado P, Amado F, et al. Analyses of pressure ulcer incidence in inpatient setting in a Portuguese hospital. Journal of Tissue Viability. 2016;25(4):209-15.

7. DGS. Escala de Braden: Versão Adulto e Pediátrica (Braden Q). Lisboa: Direção-Geral da Saúde; 2011.

8. Cox J. Predictors of pressure ulcers in adult critical care patients. Am J Crit Care. 2011;20(5):364-75.

9. Braden BJ. The Braden Scale for Predicting Pressure Sore Risk: reflections after 25 years. Adv Skin Wound Care. 2012;25(2):61.

10. Lalkhen AG, McCluskey A. Clinical tests: sensitivity and specificity. Continuing Education in Anaesthesia, Critical Care & Pain. 2008;8(6):221-3.

11. Park SH, Choi YK, Kang CB. Predictive validity of the Braden Scale for pressure ulcer risk in hospitalized patients. J Tissue Viability. 2015;24(3):102-13.

12. Coleman S, Nelson EA, Keen J, Wilson L, McGinnis E, Dealey C, et al. Developing a pressure ulcer risk factor minimum data set and risk assessment framework. J Adv Nurs. 2014;70(10):2339-52.

13. Coleman S, Nixon J, Keen J, Wilson L, McGinnis E, Dealey C, et al. A new pressure ulcer conceptual framework. J Adv Nurs. 2014;70(10):2222-34.

14. Coleman S, Nelson EA, Vowden P, Vowden K, Adderley U, Sunderland L, et al. Development of a generic wound care assessment minimum data set. J Tissue Viability. 2017;26(4):226-40.

15. NPUAP, EPUAP, PPPIA. Prevention and Treatment of Pressure Ulcers: Quick Reference Guide. Perth, Australia: Cambridge Media; 2014.

**Keywords**

Nursing Assessment, Portugal, Pressure Ulcer, Risk Assessment, Sensitivity and Specificity.

### O69 Health literacy: the importance of experimental activities in the 1st cycle of basic education: report of an educational intervention on hand hygiene

#### Maria C Lamas^1,2,3^, Carla Lago^4^

##### ^1^Escola Superior Saúde, Instituto Politécnico do Porto, 4200-072 Porto, Portugal; ^2^Centro de Investigação em Saúde e Ambiente, Instituto Politécnico do Porto, 4200-072 Porto, Portugal; ^3^Centro de Investigação em Tecnologias e Serviços de Saúde, 4200-450 Porto, Portugal; ^4^Escola EB1/JI Pícua, Agrupamento de Escolas de Águas Santas, 4425-143 Águas Santas, Maia, Portugal

###### **Correspondence:** Maria C Lamas (mariaceulamas@gmail.com)

**Background**

Primary school students usually have very little previous knowledge about a number of educational issues. So, it is important to create moments where the students can tell whatever they know about a subject, in order to make an additional scientific explanation. The program of the 1^st^ cycle of basic education aims to develop an attitude of permanent research and experimentation, and the part “*The health of your body*”, to produce knowledge and the application of norms of body hygiene [1]. However, the contents expressed in the textbooks for these levels of education do not justify the need for children to adopt these hygiene habits, which must be acquired as early as possible, to be a systematic routine throughout life. On the other hand, it allows to eradicate some of the alternative conceptions that some 1^st^ cycle students present on some issues [2], as the notion about the morphological view of microorganisms away from reality, idealizing them similar to animals [3,4,5]. There is evidence that children are able to learn about microorganisms at this age [3,4,5] and it is desirable that it occurs as early as possible, avoiding late conceptual changes that are difficult to reconstruct in their entirety [4]. For some authors [6,7], children should realize that the knowledge learned in the classroom can be applied in their daily lives.

**Objective**

In this context and with the purpose of promoting scientific and critical literacy, we developed an activity about hand hygiene because handwashing should be learned and be a properly reasoned behaviour.

**Methods**

The activities were developed by all 26 students in the class A, 2^nd^ grade of the School EB1/JI Picua. The students' age ranged from 7 to 8 years, with 54% (14) boys and 46% (12) girls. It started with the question “*Handwashing: Why, When, How*?”. According to the conceptions expressed by the students the appropriate theoretical contents were presented in a gradual and interactive way. This was followed by the experimental procedure with permanent monitoring and support based on the succeeding steps: role-playing stages for proper handwashing; applied activity; listing expected results; observation of cultures and microscopic observation of microorganisms, recording and reflection about the results achieved.

**Results**

All groups showed the expected results, *i.e.*, higher microbial growth in the quadrants corresponding to unwashed hands.

**Conclusions**

Giving the results and the theoretical framework, the students learned proper concepts on the subject, which allowed them a better understanding of the world around them.

**References**

1. DGE - Direção Geral de Educação (2004). Organização Curricular e Programas. 1º Ciclo do Ensino Básico. Lisboa, Ministério da Educação e Ciência, 4ª edição.

2. Mafra, P., Lima, N., Carvalho, G. (2015). Microbiologia no 1º Ciclo do Ensino Básico: Uma proposta de atividade experimental sobre a higiene das mãos. Livro de atas do XI Seminário Internacional de Educação Física, Lazer e Saúde.

3. Byrne J, Sharp J. Children’s ideas about micro-organisms. School science review. 2006;88(322):71-79.

4. Byrne J. Models of Micro-Organisms: Children’s knowledge and understanding of micro-organisms from 7 to 14 years old. International Journal of Science Education. 2011;33(14):1927-1961.

5. Mafra, P. (2012). Os Microrganismos no 1.º e 2.º Ciclos do Ensino Básico: Abordagem Curricular, Conceções Alternativas e Propostas de Atividades Experimentais. Tese de Doutoramento. Braga: Universidade do Minho, Portugal.

6. Pro, A. (2012). Los cuidadanos necessitan connocimientos de ciências para dar respuestas a los problemas de su contexto. In Pedrinaci, E. (coord.), Caamaño, A. Cañal, P.; Pro, A. 11 ideas clave. El desarollo de la competência científica. Barcelona: Editorial Graó.

7. Lupión, T. e Prieto, T. (2014). La contaminación atmosférica: un contexto para ell desarollo de competências en el aula de secundária. Enseñanza de las Ciencias, 32 (1), 1-18.

**Keywords**

Hygiene, Handwashing, 1^st^ cycle, Monitored support, Microrganisms.

### O70 Stand by me! Assessing the risk of falls in community –dwelling older adults

#### Luís PT Lemos^1^, João Pinheiro^2^, Edite Teixeira-Lemos^3,4^, Jorge Oliveira^3,4^, Ana P Melo^5^, Anabela C Martins^6^

##### ^1^Centro Hospitalar Tondela-Viseu, 3509-504 Viseu, Portugal; ^2^Faculty of Medicine, University of Coimbra, 3004-504 Coimbra, Portugal; ^3^Escola Superior Agrária de Viseu, Polytechnic Institute of Viseu, 3500-606 Viseu, Portugal; ^4^Centre for the Study of Education, Technologies and Health, Polytechnic Institute of Viseu, 3504-510 Viseu Portugal; ^5^Laboratory Medicine Unit and Department of Quality and Risk Management, Hospital Distrital da Figueira da Foz, 3094-001 Figueira da Foz, Portugal; ^6^Physiotherapy Department, Coimbra Health School, Polytechnic Institute of Coimbra, 3046-854 Coimbra Portugal

###### **Correspondence:** Edite Teixeira-Lemos (etlemos2@gmail.com)

**Background**

About a third of community-dwelling adults age 65 and older fall each year. Accidental falls are a cause of fractures, traumatic brain injury, and even death. They can also lead to restrictions in participation, eventually resulting in loss of independence in normal activities of self-care. Falls in older adults are multifactorial and can be caused by medical conditions, cognitive impairment, medications, and home hazards. Therefore, a single identifiable factor may account for only a small portion of the fall risk in the community-dwelling elderly population, stressing the need for a multifactorial evaluation in this population.

**Objective**

Identify the risk of accidental falls in an independent elderly population by using functional tests that can be routinely applied in clinical practice and evaluate the influence of medication in the risk of falling.

**Methods**

The sample consisted of 108 individuals who attended a health care facility between October 2016-January 2017. Inclusion criteria: age 65-85, Functional Independence Measure (FIM) ≥ 120 and Timed Up and Go (TUG) ≤ 12s. Individuals with serious cognitive or motor impairment were excluded. A form was filled with sociodemographic data, daily medication and history of falls. Handgrip strength was measured. Fear of falling was assessed using the Activities-specific Balance Confidence (ABC) scale. Participation was evaluated using the Activities and Participation Profile related to Mobility (APPM). Informed written consent was obtained for all participants.

**Results**

The average age was 72.28±6.02. The majority of subjects were female (54.6%). Fallers were older, had lower ABC and handgrip strength. ABC showed strong negative associations with APPM. All of the functional parameters were affected by age, with older individuals performing worse than younger participants. Polypharmacy was identified in 41.7% and increased the risk of falls (OR = 3.597; CI 95% 1.174-11.024; p = 0.025). Individuals taking antidepressants showed an increased risk of falls (OR = 9.467; CI 95% 2.337-38.495; p = 0.002). Anti-arrhythmic drugs (p = 0.002), benzodiazepines (p = 0.015) and other CNS-acting medication (p = 0.039) negatively influenced ABC scores. APPM scores were higher in subjects who reported taking CNS-acting medication (p = 0.012) and anti-arrhythmic medication (p = 0.035).

**Conclusions**

Individuals with low balance confidence showed higher restrictions in participation related to mobility. All of the functional parameters evaluated in this study were affected by age. These results stress that a comprehensive and multifactorial evaluation of risk factors for falls in older people and the adoption of interventions tailored to this age group, which could include a reassessment of their usual medication, are necessary in order to reduce fall risk and fall-related injury.

**Keywords**

Accidental falls, Risk factors, Elderly, Community-dwelling, Polypharmacy.

### O71 Trust requirements for the uptake of ambient assisted living digital advisory services

#### Soraia Teles^1,2^, Ana Ferreira^2^, Pedro Vieira-Marques^2^, Diotima Bertel^3^, Constança Paúl^1,2^, Andrea C Kofler^4^

##### ^1^Institute of Biomedical Sciences Abel Salazar, Department of Behavioral Sciences, University of Porto, 4050-313 Porto, Portugal; ^2^Center for Health Technology and Services Research, 4200- 450, Porto, Portugal; ^3^SYNYO GmbH, 1060 Vienna, Austria; ^4^Zurich University of Applied Sciences, Reidbach 8820 Wädenswil, Zurich, Switzerland

###### **Correspondence:** Soraia Teles (teles.s.soraia@gmail.com)

**Background**

For the last 10 years, Ambient Assisted Living (AAL) solutions have been conquering an important place in policies addressing economic and social challenges resulting from population ageing [1]. The AAL concept corresponds to a new paradigm building on ubiquitous computing devices and new interaction forms to improve older adults’ health, autonomy and security [2]. In spite of promising contributions of AAL solutions for ageing in place, low adoption by end users was reported [3-5]. This is thought to result from the intersection of technology features, user characteristics and attitudes [6]. Research has suggested that among attitudinal factors preventing adoption of these solutions there is a lack of trust, substantiated, among other factors, by user’s concerns about data security and privacy [5,7-10]. Digital advisory services for AAL solutions have to foster not only user’s trust on the advisory service *per se*, but also on AAL products and services and the web communication within a community.

**Objective**

To analyse stakeholders’ attitudes and requirements towards AAL digital advisory services, applying the findings to develop a pan-European advisory and decision-support platform for AAL solutions (ActiveAdvice).

**Methods**

A qualitative approach was used. Thirty-eight semi-structured interviews with AAL stakeholders– older adults and informal caregivers, businesses and government representatives– were conducted in six European countries (Austria, Switzerland, Belgium, Netherlands, Portugal and UK). The data was analysed using the matrix method [11].

**Results**

For the uptake of AAL digital advisory services, the level of user’s trust in the system seems to be critical. Features emerging as crucial to foment trust in digital advisory services were threefold: presence of security and privacy cues; personalization-related cues; and community features, including availability of client-to-client interactions and feedback given by reliable peers or experts. Older adults expressed their interest in becoming active in a digital community if provided an environment perceived as secure and, simultaneously, easy to use.

**Conclusions**

Building trust in AAL digital advisory services depends on multiple and complex user requirements. Security issues have shown to be of utmost relevance due to the nature of information exchanged, *i.e.* personal, health-related and sensitive data, and generational preferences, with privacy and security cues having primacy for ‘Baby Boomers’, as supported by previous research [12]. These findings stress the need for a paradigm shift towards user-centred and user empowering models and mechanisms for securing the interaction with systems (e.g. authentication mechanisms, access control models and visualization techniques; e.g. SoTRAACE model) [13].

**Acknowledgements**

The authors would like to acknowledge the co-financing by the European Commission AAL Joint

Programme and the related national agencies in Austria, Belgium, the Netherlands, Portugal,

Switzerland and the United Kingdom.

**References**

1. AAL Programme. Stategy 2014-2020 for the Active and Assisted Living Programme. 2014; Retrieved from: http://www.aal-europe.eu/wp-content/uploads/2015/11/20151001-AAL Strategy_Final.pdf.

2. Betchold U, Sotoudeh M. Assistive technologies: Their development from a technology assessment perspective. Gerontechnology. 2013;11(4):521-533.

3. Doyle J, Bailey C, Scanaill CN, van den Berg F. Lessons learned in deploying independent living technologies to older adults’ homes. Univ Access Inf Soc. 2013;13:191.

4. Michel JP, Franco A. Geriatricians and Technology. J Am Med Dir Assoc. 2014;15(12):860-2.

5. Peek ST, Wouters EJ, van Hoof J, Luijkx KG, Boeije HR, Vrijhoef HJ. Factors influencing acceptance of technology for aging in place: A systematic review. Int J Med Inform. 2014;83(4) :235-248.

6. Nedopil C, Schauber C, Glende I. AAL stakeholders and their requirement. 2013; Report by the Ambient and Assisted Living Association.

7. Damodaran L, Olphert W. User Responses to Assisted Living Technologies (ALTs) — A Review of the

Literature. Journal of Integrated Care. 2010;18(2):25-32.

8. Nordgren A. Personal health monitoring: ethical considerations for stakeholders. Journal of Information, Communication and Ethics in Society. 2013;11(3):156-173.

9. Olphert W, Damodaran L, Balatsoukas P, Parkinson C. Process requirements for building sustainable digital assistive technology for older people. Journal of Assistive Technologies. 2009;3(3):4-13.

10. Wright D. Structuring stakeholder e-inclusion needs. Journal of Information, Communication and Ethics in Society. 2010;8(2):178-205.

11. Nadin S, Cassell C. Using Data Matrices. In: Cassel C, Symon G, editors. Essential Guide to Qualitative Methods in Organizational Research. London: SAGE Publications Ltd ; 2011. p. 271–287.

12. Obal M, Kunz W. Trust development in e-services: a cohort analysis of Millennials and Baby Boomers. J. Serv. Manag. 2013;24(1):45–63.

13. Moura P, Fazendeiro P, Marques P, Ferreira A. SoTRAACE — Socio-technical risk-adaptable access control model. 2017 International Carnahan Conference on Security Technology (ICCST) [Internet]. IEEE; 2017 Oct; Available from: 10.1109/ccst.2017.8167835

**Keywords**

Ambient Assisted Living (AAL), Digital Advisory Services, Trust in Online Services, Data Security.

### O72 Self-confidence for emergency intervention and nurses’ perceptions of importance of the Intra-Hospital Emergency Team

#### Marisa J Cardo^1^, Pedro Sousa^2,3^

##### ^1^Centro Hospitalar de Leiria, 2410-197 Leiria, Portugal; ^2^School of Health Sciences, Polytechnic Institute of Leiria, 2411-901 Leiria, Portugal; ^3^Center for Innovative Care and Health Tecnhology, Polytechnic Institute of Leiria, 2411-901 Leiria, Portugal

###### **Correspondence:** Marisa J Cardo (marisacardo@gmail.com)

**Background**

The Intra-Hospital Emergency Team (IHET) has emerged to respond to situations of clinical deterioration of hospitalized patients, being nurses the fundamental links in the activation. During a situation of clinical deterioration with the need for IHET intervention, the actions of nurses in inpatient services depend on many factors. To facilitate the activation and effectiveness of this team, nurses must have self-confidence to carry out the activation of IHET. Nurses believe that IHET’s intervention is important in the pursuit for safe care and that the most important ingredient for the effective use of this team is the nurse.

**Objective**

This study aims to evaluate the level of self-confidence for emergency intervention of nurses and to identify the nurses’ perceptions of the importance of the IHET.

**Methods**

This correlational study included 129 nurses from the Centro Hospitalar de Leiria who answered a questionnaire about the perception of IHET importance and the self-confidence scale for emergency situations validated for the Portuguese population by Martins et al. (2014), which consists of 12 items with Likert type responses. Pearson correlation and t-student were used for data analysis.

**Results**

In this study, 84% were female nurses with a mean age of 39.70 ± 9.02 years, with an average professional experience of 16.97 ± 8.95 years, and the majority with training in the emergency area (94%). The mean self-confidence level of the nurses was 3.263 ± 0.571, for a maximum of 5 points. Regarding the nurses’ perception of importance of the IHET, a positive tendency was observed (results ranged from 3.426 ± 0.570 to 4.775 ± 0.419). A partial relation between the professional experience (r = 0.25; p = 0.004), the training (t = 6.143; p ≤ 0.0001) and the level of self-confidence (r = -0.205; p = 0.020) with the level of perception is highlighted.

**Conclusions**

In this study, regarding the self-confidence for emergency intervention, the nurses demonstrated confidence, albeit modestly. Likewise, they presented a tendency towards positive agreement regarding the importance of IHET. Of note, the higher the experience of the nurses, the greater the importance attributed to the IHET. Finally, it was verified that the higher the nurse's self-confidence index, the lower the feeling of insecurity in an emergency situation.

**Keywords**

Hospital Rapid Response Team, Nursing, Emergency situation, Perception of importance, Self-confidence in emergency

### O73 Relationship between cognitive impairment and nutritional assessment on functional status in institutionalized Portuguese older adults

#### Catarina Caçador^1^, Edite Teixeira-Lemos^2,3^, Jorge Oliveira^2,3^, Fernando Ramos^1^, Manuel T Veríssimo^4,5^, Maria C Castilho^6^

##### ^1^Faculty of Pharmacy, University of Coimbra, 3000-548 Coimbra, Portugal; ^2^Agrarian School, Polytechnic Institute of Viseu, 3500-606 Viseu, Portugal; ^3^Centre for the Study of Education, Technologies and Health, Polytechnic Institute of Viseu, 3504-510 Viseu, Portugal; ^4^Hospitais da Universidade de Coimbra, Centro Hospitalar e Universitário de Coimbra, 3000-075 Coimbra, Portugal; ^5^Faculty of Medicine, University of Coimbra, 3004-504 Coimbra, Portugal; ^6^Laboratory of Bromatology, Pharmacognosy and Analytical Science, Faculty of Pharmacy, University of Coimbra, 3000-548 Coimbra, Portugal

###### **Correspondence:** Catarina Caçador (cacasabel@hotmail.com)

**Background**

Elderly are particularly vulnerable to nutritional change deficits. Malnutrition in elderly patients is frequently underdiagnosed [1] and it has a large number of negative consequences on health and quality of life [2]. Even in industrialized countries undernutrition is becoming an alarming phenomenon, especially involving elderly institutionalized subjects. Few studies have focused on the relationship between patient’s nutritional assessment and a severity of cognitive impairments, comorbidity and functional status in institutionalized older adults.

**Objective**

In the present study we evaluated the relationship between functional disability, cognitive impairment and nutritional status.

**Methods**

This was an observational study with data collected from residents living in institutions in the district of Viseu (centre of Portugal). Inclusion criteria were: subjects aged 65 or older, living in institutions, that voluntarily accepted to participate in the study. All of the 216 subjects studied underwent multidimensional geriatric assessment. A form was filled with sociodemographic data and the nutritional state was assessed with the Mini Nutritional Assessment (MNA), whereas cognitive performance was evaluated by the Mini-Mental State Examination (MMSE). The functional state was assessed by Barthel Index (BI). Statistical evaluations (p < 0.05) were based on Qui-square tests between Barthel Index (BI), Mini Mental State Evaluation (MMSE), Body Mass Index (BMI) and Mini Nutritional Assessment (MNA) scores. Statistical evaluations (p < 0.05) were based on Qui-square tests between BI, MMSE, BMI and MNA scores.

**Results**

A cognitive impairment in MMSE performance was displayed in 39.4% patients. Slight disability occurred in 69.4% of the residents, 24.1% were independent in activities of daily living and only 6.5% of the seniors had moderate dependence. There was a proportional increase of the cognitive impairment of the elderly (p ≤ 0.001) with increasing dependence. According to MNA, 27.8% of the elderly were at risk of malnutrition and 71.3% showed no nutritional problems. Statistical analysis showed that dependence increased the risk of malnutrition.

**Conclusions**

A close relationship between malnutrition and functional dependence has been obtained. Both tests, MNA and BI, are positively associated. The scores of BI can help to determine who may be at risk of poor nutrition.

**References**

1. Gariballa SE. Nutritional support in elderly patients. J Nutr Health Aging. 2000; 4: 25-7.

2. Pérez-Llamas F. Risk of desnutrition in the Spanish population. Evaluation of the current situation and need for a nutritional intervention. Med Clin(Barc) 2012; 139:163-4

**Keywords**

Elderly, Cognitive impairment, Functional status, Nutritional assessment, Malnutrition.

### O74 The evaluation of nursing care provided by Integrated Continuing Care Teams

#### Carlos Vilela^1,2^, Paulino Sousa^2^, Filipe Pereira^2^

##### ^1^Institute of Health Sciences, Portuguese Catholic University, 1649-023 Lisboa, Portugal; ^2^Nursing School of Porto, 4200-072 Porto, Portugal

###### **Correspondence:** Carlos Vilela (carlosvilela@esenf.pt)

**Background**

The evaluation of nursing care is an imperative for the continuous development of quality improvement and for the cyclical redefinition of action plans that correspond to the real needs of the population. In the specific case of the Integrated Continuing Care Teams (ICCT), inserted in the National Network of Continued Integrated Care of Portugal, it is justified the use of a panel of indicators of health gains “Sensitive to Nursing Care”, given the nature of the services provided in these units, which helps to measure the clinical results obtained.

**Objective**

1) To identify the main nursing care needs of ICCT clients; and 2) Identify health gains sensitive to nursing care related to those needs.

**Methods**

Based on the definition model of indicators “sensitive to nursing care”, we developed a quantitative study-exploratory, descriptive and correlational, using the analysis of the nursing documentation available in Information Systems in use, in a convenience sample of 217 cases, attended in four ICCT of the northern region of Portugal, from October 2012 to May 2013.

**Results**

From the analysis of 9,258 documented nursing diagnoses, it was possible to generate eight “types” of health gains indicators. Five related to the dependent person and three referring to the family caregiver (FC). Here the great incidence of the care provided in these ICCT was revealed, where the Gains in autonomy/independence in the universal requirements of self-care and Gains in knowledge of the FC were the most representative areas of the care provided. Then, came the domains of Gains in the evolution of nursing diagnoses of the dependent person (“others”) and Gains in knowledge of the dependent person. Approximately 10% of the results computed were in areas that focused on Gains in FC performance (11.20%), Gains in performance of dependent persons (10.43%) and Gains in the prevention of Ulcers of Pressure (9.32%). With an order of magnitude in the order of 3.30% emerged the field of indicators related to Gains in the evolution of nursing diagnoses centred on the Role of Care Provider.

**Conclusions**

Reflecting on these results allows a more effective approach to the representation of nursing care in the evaluation of quality and, certainly, a better redefinition of the panel of indicators at the national level, with “sensitivity” to the nursing work developed by ICCT.

**Keywords**

Healthcare Quality Indicators, Quality of Health Care, Home Care Services, Nursing Care, Nursing Care Management.

### O75 Psychosocial impact of the powered wheelchair on the social participation of its users

#### Inês Domingues^1^, João Pinheiro^1^, Anabela Martins^2^, Patrícia Francisco^2^

##### ^1^Faculty of Medicine, University of Coimbra, 3004-504 Coimbra, Portugal; ^2^Physiotherapy Department, Coimbra Health School, Polytechnic Institute of Coimbra, 3046-854 Coimbra, Portugal

###### **Correspondence:** Inês Domingues (inesfsdomingues@gmail.com)

**Background**

There is a growing prevalence of disability worldwide, which indicates an increasing number of persons who might benefit from assistive technologies. Several studies showed positive effects of the use of assistive technologies on activity and participation of adults with mobility impairments [1, 2], as well as on psychosocial factors [3, 4].

**Objective**

The purpose of this study is to assess the psychosocial impact of the powered wheelchair, evaluating its repercussions on the social participation of its users.

**Methods**

Design - Observational, descriptive, cross-sectional study; Setting – All data was collected from May to October 2017; Participants - 30 powered wheelchair users with mean age of 40.63 years old (60% male) with diverse medical conditions (SCI, TBI, CP, among others); Main outcome measures - Interviews were conducted by an independent researcher using the Quebec User Evaluation of Satisfaction with Assistive Technology (QUEST), the Psychosocial Impact of Assistive Devices Scale (PIADS) and the Activities and Participation Profile Related to Mobility (PAPM), in addition to demographic, clinical and wheelchair-related questions.

**Results**

Participants were quite satisfied with both the assistive technologies and the related services, with the lowest QUEST scores belonging to those who had been using their wheelchairs for a longer period of time. PAPM scores revealed significant restrictions in participation (6.7% of participants with mild restrictions, 56.7% with moderate restrictions and 36.7% with severe restrictions), with a worst participation profile also among the users who had the wheelchairs for a longer period. The most satisfied users were the ones with better performance in terms of social participation. PIADS scores showed a positive impact of the powered wheelchairs in all subscales, with the following average scores: total 1.37, competence 1.39, adaptability 1.32 and self-esteem 1.38. The psychosocial impact, in terms of adaptability, was higher among users who transitioned from a manual wheelchair to a powered wheelchair compared to those who already had a powered wheelchair previously (1.85 *vs* 1.10; p = 0.02).

**Conclusions**

There was an overall positive psychosocial impact of powered wheelchairs, and, therefore, an increase in the quality of life of the users. Adaptability to the device seems to be the most contributing factor to social participation.

**References**

1. Salminen AL, Brandt A, Samuelsson K, Toytari O, Malmivaara A. Mobility devices to promote activity and participation: a systematic review. J Rehabil Med. 2009;41(9):697-706.

2. Lofqvist C, Pettersson C, Iwarsson S, Brandt A. Mobility and mobility-related participation outcomes of powered wheelchair and scooter interventions after 4-months and 1-year use. Disabil Rehabil Assist Technol. 2012;7(3):211-8.

3. Martins A, Pinheiro J, Farias B, Jutai J. Psychosocial Impact of Assistive Technologies for Mobility and Their Implications for Active Ageing. Technologies. 2016;4(3):28.

4. Buning ME, Angelo JA, Schmeler MR. Occupational performance and the transition to powered mobility: a pilot study. Am J Occup Ther. 2001;55(3):339-44.

**Keywords**

Assistive technologies, Powered wheelchair, Psychosocial impact, Social participation.

### O76 Work and breastfeeding: mom’s double duty

#### Rita MF Leal^1^, Amâncio AS Carvalho^2^, Marília S Rua^1^

##### ^1^School of Health, University of Aveiro, 3810-193 Aveiro, Portugal; ^2^School of Health, University of Trás-os-Montes e Alto Douro, 5000-801 Vila Real, Portugal

###### **Correspondence:** Rita MF Leal (ritamariaferreiraleal@gmail.com)

**Background**

Work is known to be an obstacle to breastfeeding (BF) continuation [1–5].

**Objective**

To analyse if there is a relationship between the employment status of the mother, the age of the child when she returns to work, the level of difficulty in reconciling work with BF and BF duration.

**Methods**

An observational, descriptive-correlational and cross-sectional study was conducted. The population comprised mothers who had a biological child in 2012 and 2013 in the Centre region of Portugal. A non-probabilistic sample (n = 427) was collected using an online questionnaire with snowball effect from November 2015 to September 2016. Data was analysed using SPSS software.

**Results**

Most women had difficulties in reconciling work with BF (72.5%). Of these, 26.6% said that conciliating work and BF was “very difficult” or “difficult”, while 73.4% said they had “some difficulty” or “very little difficulty”. Job status (employed *versus* unemployed) did not present a statistically significant relationship with BF duration. As to the age of the child when the mother returned to work, we verified a statistically significant relationship (p = 0.001) with BF duration. The level of difficulty the mother experienced in reconciling her job with BF also presented a statistically significant relationship (p = 0.002) with BF duration.

**Conclusions**

The majority of mothers reported difficulties in reconciling work with BF. Women who returned to work before their child was 6 months old had shorter BF duration. This reinforces the need to explore family’s timely expectations, and plan strategies to promote an effective management of work and BF. In this context, we believe there is a need to establish in our culture jurisdiction policies that favour BF and work. Health professionals should act as mediators in the definition of family-oriented health policies. In this case, the extension of a parental leave for mothers up to 6 months postpartum, the existence of a place at work where breastfeeding mothers can extract and conserve their breastmilk or the existence of day-care centres in the workplace, in accordance to the Global Strategy for Infant and Young Child Feeding [6,7], may prolong BF duration and ease mothers difficulty in managing BF when returning to work.

**References**

1. Lynch S. Breastfeeding and the workplace. Community Pract. 2016;89(6):29–31.

2. Smith JP, McIntyre E, Craig L, Javanparast S, Strazdins L, Mortensen K. Workplace support, breastfeeding and health. Fam Matters. 2013;93:58–73.

3. Sriraman NK, Kellams A. Breastfeeding: What are the Barriers? Why Women Struggle to Achieve Their Goals. J Women’s Heal. 2016;0:1–9.

4. UNICEF. From the First Hour of Life: Making the case for improved infant and young child feeding everywhere PartI: Focus on Breastfeeding [Internet]. New York; 2016. Available from: http://www.unicef.pt/docs/pdf_publicacoes/FromTheFirstHourOfLife-Part1.pdf

5. Rivera-Pasquel M, Escobar-Zaragoza L, González de Cosío T. Breastfeeding and Maternal Employment: Results from Three National Nutritional Surveys in Mexico. Matern Child Health J. 2015;19(5):1162–72.

6. World Health Organization, United Nations Children’s Fund. Global Strategy for Infant and Young Child Feeding. World Heal Organ [Internet]. 2003 [cited 2016 Dec 21];1–30. Available from: http://www.paho.org/english/ad/fch/ca/GSIYCF_infantfeeding_eng.pdf

7. IBFAN Portugal Rede Internacional Pró-Alimentação Infantil. Relatório de Portugal da Iniciativa Mundial Sobre Tendências do Aleitamento Materno (WBTi) Situação da Estratégia Global para a Alimentação de Lactentes e Criança [Internet]. 2015 [cited 2016 Aug 29]. Available from: http://www.worldbreastfeedingtrends.org/GenerateReports/report/WBTi-Portugal-2015.pdf

**Keywords**

Breastfeeding, Employment, Job, Parental leave.

### O77 Health information shared in blogs by breast cancer survivors living in Portugal

#### Francisca MMC Pinto^1^, Paulino AF Sousa^2,3^, Maria RSP Esteves^4,5^

##### ^1^Universidade Católica Portuguesa, 4169-005 Porto, Portugal; ^2^Escola Superior de Enfermagem do Porto, 4200-072 Porto, Portugal; ^3^Centro de Investigação em Tecnologias e Serviços de Saúde, 4200-450 Porto, Portugal; ^4^Cooperativa de Ensino Superior Politécnico e Universitário, 4560-462 Penafiel, Portugal; ^5^Instituto de Investigação e Formação Avançada em Ciências e Tecnologias Saúde, 4560-462 Penafiel, Portugal

###### **Correspondence:** Francisca MMC Pinto (franciscampinto@gmail.com)

**Background**

Breast cancer survivors (BCS), and other cancer patients, are digital social networks users; have their personal blogs where share their life/disease experiences after being diagnosed for cancer [1, 2]. Research on blogging activity among BCS is scarce and suggests that is a multifaceted activity with several purposes: self-management of emotions, problem-solving, and sharing information [3]. But the question remains: what health information is share by BCS in blogosphere?

**Objective**

The present work reports the results of a study that explored personal blogs of BCS who lives in Portugal, with focus on written health information that was posted and/or commented.

**Methods**

A qualitative study design and thematic content analysis. Blog selection by snowball strategy that included 3 phases: I) phase 1: first 20 search results on Google, with Portuguese keywords “*blogues cancro da mama*”; II) phase 2 and 3: links to other blogs that are present on each blog selected on phase 1 and 2, respectively. Blogs included for analysis met all criteria for inclusion: I) personal blog of women self-identified with a breast cancer diagnosis; II) blogger profile allows to confirm residence in Portugal; III) blog is public domain; IV) blog must present data related to post-primary treatment phase. Data collection was done between March–November, 2017. The scope analysis started on first post after finish primary treatment for breast cancer and ended in blog’s last post at time we finished to read it.

**Results**

38 blogs were included for analysis. Results refer to health information shared by BCS in posts and commentaries between 2007-2017. Most of the information shared were uncertainties regarding: I) nutrition & physical exercise recommendations; II) management of long-term side effects of cancer treatment and comorbidities; III) management recurrence risk and psychological wellbeing; IV) treatment plans & health surveillance during survivorship; V) management of body image changes; VI) non-conventional therapies benefits & risks; vii) news about research on cancer. Less shared information regards to: I) return to work & social protection; II) general healthcare recommendations; III) community support to cancer patients; IV) genetics & heredity; V) sexuality; VI) infertility/fertility after breast cancer.

**Conclusions**

BCS use personal blogs to share difficulties, uncertainties and to search for information support to manage their health condition. Having access to useful information and education may help BCS manage uncertainty in illness, improve health literacy and self-efficacy to manage health condition. This study alerts health professionals to pay attention to BCS information and emotional needs.

**References**

1. Kim S, Chung D. Characteristics of cancer blog users. J Med Libr Assoc. 2007;95(4):445-50.

2. Damásio C, Nunes LM, Sobral JM. A Análise de Redes Sociais no estudo do processo da construção da ajuda mútua da pessoa com doença oncológica com blogue. REDES- Revista hispana para el análisis de redes sociales. 2014;25(1):153-89.

3. Koskan A, Klasko L, Davis SN, Gwede CK, Wells KJ, Kumar A, et al. Use and Taxonomy of Social Media in Cancer-Related Research: A Systematic Review. American Journal of Public Health. 2014;104(7):e20-e37.

**Keywords**

Breast cancer survivors, Blogs, Health information needs, Content analysis.

### O78 Visual images spectrum android classification for diabetic foot ulcers

#### Ricardo Vardasca^1^, Rita Frade^2,3^, Rui Carvalho^4^, Joaquim Mendes^2,3^

##### ^1^Faculdade de Engenharia, Universidade do Porto, 4200-465 Porto, Portugal; ^2^Porto Biomechanics Laboratory, Faculdade de Engenharia, Universidade do Porto, 4200-465 Porto, Portugal; ^3^instituto de Ciência e Inovação em Engenharia Mecânica e Engenharia Industrial - Laboratório Associado de Energia, Transportes e Aeronáutica, 4200-465 Porto, Portugal; ^4^Clínica Multidisciplinar do Pé Diabético, Centro Hospitalar do Porto, 4099-001 Porto, Portugal

###### **Correspondence:** Ricardo Vardasca (ricardo.vardasca@fe.up.pt)

**Background**

According to the Portuguese Society of Diabetology, about 415 million (8.8%) of the worldwide population was diagnosed with Diabetes Mellitus in 2015. Being the Portuguese population incidence of 1 million people (13.3% of the total population), with a national annual total estimated cost of 1.7 billion euros with this condition. One in each four patients of DM develop Diabetic Foot Ulcer (DFU) in their lifetime, ending some in amputations and consequently in death [1]. Furthermore, the Directorate-General of Health estimated that in 2016, the prevalence of DFU was of 11.5% in the Portuguese population [2]. Throughout the years, DFU assessment tools have been created, such as: I) scales, which depend on visual examination being highly subjective; II) invasive methods that use manual procedures for depicting the shape, area, depth and volume of wounds, that are time consuming, susceptible to human errors and can lead to wound contamination [3]; or III) non-invasive methods such as optical based techniques which provide three-dimensional information about the lesion, which are expensive, time consuming and require user training [4]. Therefore, more objective measures are required.

**Objective**

This research study aims to create an objective and simple methodology based in a mobile application which incorporates an algorithm that characterises DFU ulcers providing information about its area and tissue colour composition.

**Methods**

An Android mobile application was developed, tested and evaluated in 200 diabetic foot ulcers, after signing the informed consent and the procedure being explained to patients. The study was approved by the ethical committee of Centro Hospitalar do Porto.

**Results & Conclusions**

The use of this new android mobile app showed a high correlation with the traditional clinical assessment (r^2^ = 0.97), reducing subjectivity, avoiding wound contamination probability and smaller costs when compared to conventional solutions.

**Acknowledgements**

The authors gratefully acknowledge the funding of project NORTE-01-0145-FEDER- 000022 - SciTech - Science and Technology for Competitive and Sustainable Industries, cofinanced by Programa Operacional Regional do Norte (NORTE2020), through Fundo Europeu de Desenvolvimento Regional (FEDER) and of project LAETA - UID/EMS/50022/2013.

**References**

1. Sociedade Portuguesa de Diabetologia. Relatório Anual do Observatório Nacional da Diabetes. Diabetes: Factos e Números; 2016.

2. Direcção Geral de Saúde. Relatório do Programa Nacional para a Diabetes; 2017.

3. Plassmann P. Measuring wounds. Journal of Wound Care 4(6); 1995. 269-272.

4. Wang L, Pedersen PC, Strong DM, Tulu B, Agu E, Ignotz R, He Q. An Automatic Assessment System of Diabetic Foot Ulcers Based on Wound Area Determination, Color Segmentation, and Healing Score Evaluation. Journal of diabetes science and technology 10(2); 2016. 421-428.

**Keywords**

Android, Classification, Diabetic foot ulcers, Imaging, Wound assessment.

### O79 Sleep disorders in elderly: is it a problem?

#### Vítor Moreira^1^, Ângela Mota^1^, Bárbara Santos^1^, Olívia R Pereira^1,2^, Xavier Costa^1,3^

##### ^1^Departamento de Tecnologias de Diagnóstico Terapêutica, Escola Superior de Saúde, Instituto Politécnico de Bragança, 5300-121 Bragança, Portugal; ^2^Centro de Investigação de Montanha, Instituto Politécnico de Bragança, 5300-253 Bragança, Portugal; ^3^Centro Hospitalar de Trás-os-Montes e Alto Douro, Unidade Hospitalar de Chaves, 5400-279 Chaves, Portugal

###### **Correspondence:** Olívia R Pereira (oliviapereira@ipb.pt)

**Background**

Sleep disorders are one of the most relevant clinical symptoms in adults, with increasing prevalence throughout life, reaching in large scale the elderly population.

**Objective**

The present study aimed to characterize sleep disorders in the elderly and its pharmacological therapy.

**Methods**

A cross-sectional study was performed through application of a questionnaire to 381 elderlies in pharmacies of Braga, Bragança and Porto cities. Descriptive statistics were used, as well as univariate and multivariate statistical analysis, with a significance level of 5%.

**Results**

Elders were most from female gender (60.1%), aged between 65 and 74 years (49.6%) and lived in rural areas (73.4%). Just 36.5% of the elderly practice physical exercise and an important amount of elderly drink coffee and tea (68.8% and 73.2%, respectively). Concerning sleep characteristics, the elders go to bed between 6 p.m. and 2 a.m. and about half of participants (52.8%) go between 10 p.m. and 12 a.m. Approximately one third had difficulty in falling asleep (38.1%), especially the elderly from the region of Bragança. During sleep, a large proportion of the elderly reported having sleep stops (78.2%) usually for 15-30 minutes and 26.5% reported waking up twice during the night. Taking into account that the time of delay to sleep is an important factor, in the present study, this was statistically related with the gender (p = 0.003) and with taking medication to sleep (p < 0.001). The same two factors are statistically related with “*wake up during the night*” (p = 0.046, p = 0.003, respectively). 40.7% of the surveyed elderly have been diagnosed with sleep disorders, mainly insomnia (19.7%) following by restless legs syndrome (3.4%), excessive drowsiness (2.9%) and sleep apnoea, sleep-walking and narcolepsy (about 1%). It is important to refer that among the elderly, that assume to suffer from sleep disorders, just 40.7% have been consulted by a physician. Of those who consulted a doctor, 21.3% of the elderly were advised to change their lifestyle habits, such as, to avoid heavy meals before bedtime, to establish a sleep routine, to lie down only when he/she is sleepy and to practice physical activity. Concerning the pharmacological therapy, 41.7% take medication for sleep disorders, 9% take medication without consulting a doctor, while 32.5% elderly people take medication after consulting a doctor. From these, the most used are benzodiazepines such as alprazolam (12.5%), diazepam (8.6%), lorazepam (4.5%) and brotizolam (3.7%).

**Conclusions**

Sleep disorders are frequent in the elderly population. It is necessary to raise awareness in this population group, which associates sleep problems to age.

**Keywords**

Sleep Disorders, Elderly, Pharmacological Therapy, Sleep and aging.

### O80 Physical and mental health in community-dwelling elderly: functional assessment and implications to multidisciplinary clinical practice

#### Rogério Rodrigues^1^, Zaida Azeredo^2^, Sandrina Crespo^1^, Cristiana Ribeiro^1^, Isabel Mendes^1^

##### ^1^Health Sciences Research Unit: Nursing, Nursing School of Coimbra, 3046-851 Coimbra, Portugal; ^2^Research in Education and Community Intervention, Piaget Institute, 1950-157 Lisbon, Portugal

###### **Correspondence:** Rogério Rodrigues (rogerio@esenfc.pt)

**Background**

Disability in old age may pose barriers to the achievement of goals and the ability to carry on roles that are important to a person. Knowledge about the functional disability in physical and mental areas in old people is crucial for the planning of interventions by the health technicians.

**Objective**

To evaluate functional physical and mental abilities of community-dwelling elderly for planning health care and the implementation of services.

**Methods**

Quantitative descriptive-correlational study of the project “*The oldest old: Coimbra*’*s ageing study*” PTDC/CS-SOC/114895/2009 [1]. Sample constituted by 202 elderlies from a population of 808 (three age groups: 65-74; 75-84 and ≥ 85 years old), obtained in a randomized and probabilistic trial, from the files of users of a Health Centre, after ethic commission approval. As instrument and method of data collection was used the QAFMI (Portuguese version of the Older Americans Resources and Services), to evaluate the functional status, in terms of physical and mental health. Data analysis: a) descriptive analyses on the most common pathologies, their limitation and medication consumption; b) functional evaluation using the score given by the computer software based on the model of QAFMI.

**Results**

As main results we point out, the pathologies with major interference in physical activities, which are chronic bronchitis, skin disease, arthritis or rheumatism, effects of stroke and circulation troubles. Related to the consumption of medication, it was observed that for the most cited pathologies (hypertension and cardiac problems) there is a great percentage of consumption. Others (arthritis or rheumatism) have a lower prescription. There was no statistically significant difference for physical health, in the comparative study between genders. There are differences between the age groups, with lower scores for the oldest. Related to mental functional abilities, there is a statistically significant difference for the diverse age groups, with an increase of impaired capacity for the oldest. For the whole of the sample gender differences exist, being worst scored the women.

**Conclusions**

Women and the oldest, in general, appear as the ones that present lower functional physical abilities. The classification by the QAFMI model, regarding the area of mental health, reports the approach of cognitive decline and perception of memory loss. Like in other studies, differences were found between genders, resulting in worse scores for women.

**References**

1. Rodrigues R. Os muito idosos: estudo do envelhecimento em Coimbra – Perfis funcionais e intervenção [eBook]. Coimbra: Unidade de Investigação em Ciências da Saúde: Enfermagem, Escola Superior de Enfermagem de Coimbra; 2014.

**Keywords**

Elderly, Community-dwelling, Functional assessment, Physical health, Mental health.

### O81 Adherence to therapy in elderly

#### Ana Rodrigues, Clara Rocha, Jorge Balteiro

##### Coimbra Health School, Polytechnic Institute of Coimbra, 3046-854 Coimbra, Portugal

###### **Correspondence:** Jorge Balteiro (balteiro@estescoimbra.pt)

**Background**

In recent decades, the elderly population has grown significantly, leading to an increase in the number of chronic diseases and, consequently, to an increased need for polymedication for disease control. Polymedication means the use of multiple medications, which can cause adverse reactions/drug interactions that increase depending on the number of medications administered. In elderly with high number of pathologies associated or not with age, complex therapies are instituted which may lead to non-adherence to therapy. This situation can impair the aim of treatment, worsen the disease, add errors to diagnosis and treatment itself, or even lead to therapy failure.

**Objective**

The objective of the present study is to assess the adherence to therapy in elderly institutionalized during the day and investigate the main factors that influence it.

**Methods**

The study was conducted with the collection and processing of questionnaires, consisting of 3 parts: demographic characterization (e.g., age, gender, marital status); therapeutic characterization (amount of daily medications and treatment regimen) and evaluation of adherence to therapy by adapting the scale of measurement of adherence to treatment (MAT). The study sample was made up of 51 elderlies institutionalized during the day.

**Results**

It was observed that 98% of seniors join the instituted therapy: 37.3% showed a level of 5 therapeutic membership, approximately 49% showed a level of accession of 6 and only 14.9% expressed below. Of the factors studied as susceptible of influencing therapeutic membership, it was only found that oblivion is the conditioning factor associated with the recommended therapy (p = 0.047), affecting the levels of membership.

**Conclusions**

The results obtained allowed to conclude that the high levels of membership can be associated to the fact that the elderly were institutionalized during the day, having support available. Another possible explanation is the fact that the same live with family, being also accompanied during the night.

**Keywords**

Adherence to therapy, Elderly, Polymedication, MAT.

### O82 Ionizing radiation effects in a bladder and an esophageal cancer cell lines

#### Neuza Oliveira^1^, Mafalda Vitorino^1^, Ricardo Santo^2^, Paulo Teixeira^1,3,4^, Salomé Pires^3,5^, Ana M Abrantes^3,5^, Ana C Gonçalves^5,6^, Clara Rocha^7,8^, Paulo C Simões^9^, Fernando Mendes^1,3,5^, Maria F Botelho^3,5^

##### ^1^Department Biomedical Laboratory Sciences, Coimbra Health School, Polytechnic Institute of Coimbra, 3046-854 Coimbra, Portugal; ^2^Faculty of Sciences and Technology, University of Coimbra, 3030-790 Coimbra, Portugal; ^3^Biophysics Institute-CNC.IBILI, Faculty of Medicine, University of Coimbra, 3000-548 Coimbra, Portugal; ^4^Serviço de Anatomia Patológica, Cento Hospitalar Universitário de Coimbra, 3000-075 Coimbra, Portugal; ^5^Center of Investigation in Environment, Genetics and Oncobiology, Faculty of Medicine, University of Coimbra, 3001-301 Coimbra, Portugal; ^6^Laboratory of Oncobiology and Hematology, Faculty of Medicine, University of Coimbra, 3001-301 Coimbra, Portugal; ^7^Department Complementary Sciences, Coimbra Health School, Polytechnic Institute of Coimbra, 3046-854 Coimbra, Portugal; ^8^Institute for Systems Engineering and Computers at Coimbra, 3030-290 Coimbra, Portugal; ^9^Radiation Oncology Department, Hospital and University Center of Coimbra, 3000-075 Coimbra, Portugal

###### **Correspondence:** Fernando Mendes (fjmendes@estescoimbra.pt)

**Background**

Oesophageal cancer (EC) and bladder cancer (BC) share the same embryonic origin (endoderm) and according to the latest numbers, EC is the eighth and BC is the ninth most frequently diagnosed cancers worldwide. Radiotherapy (RT) is currently used in the treatment of both types of cancer [1–4].

**Objective**

Assessment of the effects of ionizing radiation (IR) on cell lines of EC (OE19) and of BC (HT1376), namely viability and cell proliferation, characterisation of cellular death type and cell cycle, as well to establish survival factor and determination of the aggression model, after different radiation dose exposure to calculate half lethal dose (DL50).

**Methods**

Cell lines were cultured and exposed to single-shot doses of X-rays from 0.5 Gy to 12.0 Gy, except control cells (0.0 Gy). Cell viability and proliferation were assessed by trypan blue assay. The proliferation index determination was performed by immunocytochemistry trough Ki-67 expression. Flow cytometry was used to assess cell death type and cell cycle and the main morphological features of cell death were evaluated by May-Grünwald Giemsa stain. Clonogenic assays enabled assessment to differences in reproductive viability (capacity of cells to produce progeny) [5–11].

**Results**

Our results showed that IR induces cytotoxic and antiproliferative effects in OE19 and HT1376 cells in a dose-dependent manner and dose and time-dependent manner, respectively. Main types of cell death observed were apoptosis or necrosis. We also observed cell cycle arrest on G2/M phase and a decrease of the Ki-67 expression in both cell lines studied. The cell survival curves were established according to the quadratic linear model for both cell lines. For OE19 cell line the DL50 was 2.47 Gy and for HT1376 cell line was 3.10 Gy, accompanied by a decrease in the survival factor for both lines.

**Conclusions**

The direct effects of the DNA molecule that are unrepairable, activate multiple intracellular mechanisms on radio-sensitivity, such as cell death, namely by apoptosis and necrosis and cell cycle arresting G2/M phase. The increase radiation dose induces alterations in cell death, from apoptosis to necrosis. According to our results, OE19 is more radio-sensible than HT1376 cell line. This study demonstrates that molecular mechanisms underlying RT are important in oesophageal adenocarcinoma and bladder cancer therapeutic approaches.

**Acknowledgements**

The authors would like to thank to Institute for Biomedical Imaging and Life Sciences is a research Institution of the Faculty of Medicine, University of Coimbra and to Radiotherapy Service of the Centro Hospitalar e Universitário de Coimbra.

**References**

1. Torre LA, Bray F, Siegel RL, Ferlay J, Lortet-tieulent J, Jemal A. Global Cancer Statistics, 2012. CA a cancer J Clin. 2015;65(2):87–108.

2. Antoni S, Ferlay J, Soerjomataram I, Znaor A, Jemal A, Bray F. Bladder Cancer Incidence and Mortality: A Global Overview and Recent Trends. Eur Urol. 2016;1–13.

3. Nassim R, Mansure JJ, Chevalier S, Cury F, Kassouf W. Combining mTOR Inhibition with Radiation Improves Antitumor Activity in Bladder Cancer Cells In Vitro and In Vivo: A Novel Strategy for Treatment. PLoS One. 2013;8(6).

4. Rubenstein JH, Shaheen NJ. Epidemiology, diagnosis, and management of esophageal adenocarcinoma. Gastroenterology. 2015;149(2):302–317.e1.

5. Mendes F, Sales T, Domingues C, Schugk S, Abrantes AM argarida, Gon??alves AC ristina, et al. Effects of X-radiation on lung cancer cells: the interplay between oxidative stress and P53 levels. Med Oncol. 2015;32(12):266.

6. Mendes F, Domingues C, Schugk S, Abrantes AM, Gonçalves AC, Casalta-Lopes J, et al. Single Shot Irradiation and Molecular Effects on a Diffuse Large B Cell Lymphoma Cell Line. J Cancer Res Treat. 2016;4(1):9–16.

7. Santo RP. Resposta Celular à Radioterapia – Estudo in vitro em linhas celulares do carcinoma da próstata Resposta Celular à Radiação Ionizante. Faculdade de Medicina da Universidade de Coimbra; 2017.

8. Tran WT, Iradji S, Sofroni E, Giles A, Eddy D, Czarnota GJ. Microbubble and ultrasound radioenhancement of bladder cancer. Br J Cancer. 2012;107(3):469–76.

9. Barbosa T V., Rosas MP, Costa AC, Rapoport A. Valor prognóstico do Ki-67 no carcinoma indiferenciado de grandes células de glândula salivar maior: estudo de 11 casos. Rev Bras Otorrinolaringol [Internet]. 2003;69(5):629–34. Available from: http://www.scielo.br/scielo.php?script=sci_arttext&pid=S0034-72992003000500007&lng=en&nrm=iso&tlng=pt

10. Ding Z, Yang H-W, Xia T-S, Wang B, Ding Q. Integrative genomic analyses of the RNA-binding protein, RNPC1, and its potential role in cancer prediction. Int J Mol Med. 2015;36(2):473–84.

11. Franken NAP, Rodermond HM, Stap J, Haveman J, Bree C Van. Clonogenic assay of cells in vitro. 2006;(October 2016).

**Keywords**

Esophageal cancer, Urinary Bladder Neoplasms, Radiotherapy, Ki-67 antigen.

### O83 Sociodemographic characteristics and breastfeeding duration

#### Rita MF Leal^1^, Amâncio AS Carvalho^2^, Marília S Rua^1^

##### ^1^School of Health, University of Aveiro, 3810-193 Aveiro, Portugal; ^2^School of Health, University of Trás-os-Montes e Alto Douro, 5000-801 Vila Real, Portugal

###### **Correspondence:** Rita MF Leal (ritamariaferreiraleal@gmail.com)

**Background**

Sociodemographic characteristics have been related to breastfeeding (BF) duration. Research has shown interest in these factors which may even be predictive [1].

**Objective**

To analyse a correlation between sociodemographic characteristics (maternal age, level of education, marital status, number of older children, child gender, child’s year of birth) and breastfeeding (BF) duration.

**Methods**

An observational, descriptive-correlational and cross-sectional study was conducted. The population comprised mothers who had a biological child in 2012 and 2013 in the Centre region of Portugal. A non-probabilistic sample (n = 427) was collected using an online questionnaire with snowball effect. Data was analysed using SPSS software.

**Results**

Maternal age, marital status, child’s gender and child’s year of birth did not present a statistically significant relationship with BF duration. On the other hand, mothers educational level (p = 0.001) and the number of older children (p = 0.018) presented a statistically significant relationship with BF duration.

**Conclusions**

Our study did not establish a correlation between maternal age and BF duration which contradicts other findings [1–3] and can be explained by pregnancy and childbirth postponement nationwide [4]. In relation to mothers’ educational level, we verified a statistically significant relationship with BF duration, being that the mothers who detained a higher level of education, breastfed longer [1,5–7]. As for marital status, we did not verify a statistically significant relationship with BF duration, which is in line with a recent Portuguese study [5] but differs from other international studies [2,3] that refer that married mothers breastfeed longer. This difference can be explained by cultural differences and by the paradigmatic change of the women’s role, concept of marriage, and a diversity of forms of family life (great increase in unmarried couples). We also verified an increasing tendency in BF duration with the increase of the number of older children [6,7], but it was not lowest in primiparous women which contradicts other findings [6,7]. This may be clarified, once again, by the sociodemographic changes in the last decade in Portugal that include population ageing, an increasing tendency for families with an only child [4] and the great investment in parenthood and child well-being. Health care professionals should consider that sociodemographic characteristics are in constant change and so is its relation to health. These findings help health professionals to identify who may be most vulnerable to early weaning and allows them to explore expectations and develop a care plan accordingly.

**References**

1. Scott J a, Binns CW. Factors associated with the initiation and duration of breastfeeding: a review of the literature. Breastfeed Rev. 1999;7(1):5–16.

2. Callen J, Pinelli J. Incidence and duration of breastfeeding for term infants in Canada, United States, Europe, and Australia: a literature review. Birth. 2004;31(4):285–92.

3. Ogbuanu C, Glover S, Probst J, Hussey J, Liu J. Balancing Work and Family: Effect of Employment Characteristics on Breastfeeding. J Hum Lact. 2011;27(3):225–38.

4. Delgado A, Wall K. Famílias nos Censos 2011: Diversidade e Mudança [Internet]. 1a edição. Instituto Nacional de Estatística, editor. Lisboa: Imprensa de Ciências Sociais; 2014 [cited 2016 Dec 13]. 239 p. Available from: http://repositorio.ul.pt/bitstream/10451/23625/1/ICS_SAtalaia_VCunha_KWall_SMarinho_VRamos_Como_ASITEN.pdf

5. Dias A, Monteiro T, Oliveira D, Guedes A, Godinho C, Alexandrino AM. Aleitamento materno no primeiro ano de vida : prevalência, fatores protetores e de abandono. Acta Pediátrica Port. 2013;44(6):6–11.

6. Caldeira T, Moreira P, Pinto E. Aleitamento materno: estudo dos factores relacionados com o seu abandono. Rev Port Clínica Geral Clin Geral. 2007;23:685–99.

7. Lopes B, Marques P. Prevalência do aleitamento materno no distrito de Viana do Castelo nos primeiros seis meses de vida. Rev Port Clínica Geral. 2004;20:539–44.

**Keywords**

Age, Breastfeeding, Education, Population characteristics, Population dynamics.

### O84 Population analysis of cleft lip and/or palate patients treated in the Postgraduate Orthodontic Department of the Faculty of Medicine of the University of Coimbra

#### Ana Roseiro, Inês Francisco, Luisa Malo, Francisco Vale

##### Universidade de Coimbra, 3004-531 Coimbra, Portugal

###### **Correspondence:** Ana Roseiro (a-roseiro@hotmail.com)

**Background**

Cleft lip and palate is one of the most common dentofacial congenital anomalies, affecting on average 1:700 new-borns. Although the aetiology of this condition is not fully understood, it seems to be related with both genetic and environmental factors. This type of malformation may occur isolated or it could be associated with a syndrome. When compared with the general population, cleft lip and palate patients present in a larger number, a series of dental anomalies in number, size and tooth shape.

**Objective**

Analyse in a population of cleft lip and/or palate patients a number of anatomical and sociodemographic characteristics.

**Methods**

The study included 60 patients referred to the Postgraduate Orthodontic Department of the Faculty of Medicine of Coimbra by the Children’s Hospital during the year of 2015. All data related to patients was obtained through a meticulous and thorough orthodontic exam (medical history, cast models, intra and extra oral pictures and radiographic exams).

**Results**

Of the 60 patients included in the study, 65% were of male gender. Patients with 11 years of age were the most prevalent ones (5-22 years of range). The most common anomaly was unilateral lip and palate cleft (63%). Maxillary endognathy was present in 75% of the cases. 74% of the patients presented at least one dental agenesis being the upper lateral incisor the more common one.

**Conclusions**

Cleft lip and palate is more frequent in male individuals and seems to be associated with conditions like maxillary endognathy and dental agenesis, with orthodontic treatment being required in these patients.

**Keywords**

Cleft lip and palate, Dental agenesis, Maxillary endognathy.

### O85 Drug use in pregnant women of Mirandela, Macedo de Cavaleiros and Bragança

#### Ana Branco^1^, Ana Coutinho^1^, Ana Machado^1^, Bárbara Alves^1^, Miguel Nascimento^1,2^, Olívia R Pereira^1,3^

##### ^1^Departamento de Tecnologias de Diagnóstico Terapêutica, Escola Superior de Saúde, Instituto Politécnico de Bragança, 5300-121 Bragança, Portugal; ^2^Serviços Farmacêuticos, Unidade Local de Saúde do Nordeste, 5301-852 Bragança, Portugal; ^3^Centro de Investigação de Montanha, Instituto Politécnico de Bragança, 5300-253 Bragança, Portugal

###### **Correspondence:** Olívia R Pereira (oliviapereira@ipb.pt)

**Background**

The use of drugs during the gestational period is a subject of great concern, once the exposure of medicines may result in toxicities with possible irreversible lesions for the foetus. In fact, drugs in pregnancy have been restricted since the accident of thalidomide. In 1979 the U.S. Food and Drug Administration (FDA) adopted the classification of drugs as the risk associated with their use during pregnancy, these being classified into 5 classes (A, B, C, D and X).

**Objective**

The aim of the present study was to characterize the use of drug therapy in pregnant women of Mirandela, Macedo de Cavaleiros and Bragança regions.

**Methods**

A cross-sectional study was performed through application of a questionnaire to 134 pregnant women in the Northeast (Mirandela, Macedo de Cavaleiros and Bragança regions) during consultation in a health centre. Descriptive statistics were used, as well as univariate and multivariate statistical analysis, with a significance level of 5%.

**Results**

The sample comprised a total of 134 pregnant women from the Northeast area, mostly with ages between 21 and 30 years or between 31 and 40 years (56.7% and 35.8%, respectively), holding secondary or higher education (48.5% and 42.5%, respectively) and employment (67.2%). About half of the pregnant (47.8%) were in the 3rd quarter of pregnancy. 78.4% (105 women) of the pregnant had used drugs during the pregnancy, 64.4% after medical prescription, and 71.6% have acquired the medication at the pharmacy. In detail, the medication most used was folic acid (64.2%, 86 of the pregnant women) which belongs to class A; paracetamol from class B (35.1%, n = 47), iodine (17.2%, n = 23) and iron (14.9%, n=20), both belonging to the class A. Less reported drugs have included metoclopramide (6.0%) and Vitamin D_3_ (6.0%), from Class C and Class D, respectively. It is important to refer that 12.7% of the women had a chronic disease and 2.2% had an acute disease during pregnancy. Diseases more reported were asthma and diabetes.

**Conclusions**

In the present study, the use of drugs in pregnancy was independent of the education level, chronic or acute disease, locality, marital status, employment status, gestational period and health centre. The drugs most used by pregnant women belong to class A (18.5%), class B (25.9%) and class C (33.3%) and the less used belong to class D and X. Supplements such as folic acid, iodine and iron and the analgesic paracetamol were the most reported.

**Keywords**

Pregnancy, Drug therapy, Risk, Disease.

### O86 Knowledge representation about self-management of medication regime in Portuguese Nursing Information Systems

#### Inês Cruz, Fernanda Bastos, Filipe Pereira

##### Nursing School of Porto, 4200-072 Porto, Portugal

###### **Correspondence:** Inês Cruz (inescruz@esenf.pt)

**Background**

The use of a standardized language in nursing supports the nursing science and contributes to the management of the discipline's own knowledge [1, 2]. Nurses can control, practice, and teach only what they can name. The documentation of care in Nursing Information Systems in Portugal is based on the international classification for nursing practice (ICNP®) [3].

**Objective**

The purpose of this study is to describe and specify nursing diagnoses centred on the clients’ knowledge for self-managing the medication regime in chronic diseases.

**Methods**

Exploratory study. All nursing documentation, concerning all health centres and public hospitals, customized in the Portuguese nursing information System-SAPE® (2012) and in SClinico® (2016) was subject to content analysis. Content analysis of nursing documentation was based on ICNP® terminology. After conducting content analysis, the material was validated by a group of 14 nursing experts.

**Results**

A set of nursing diagnoses related to the person's knowledge on the medication regime management were specified. Knowledge refers to the development of the client's informational content about how to manage his medication regime. These diagnoses focus on the potential to improve knowledge about: self-management of the medication regime; medication regime; response to medication and side effects of medication; health services; complications and preventing complications of compromised self-management; and the use of devices to facilitate drug intake.

**Conclusions**

The specified diagnoses reflect nursing care needs that nurses document in the Portuguese nursing information systems, related to medication self-management. These results are contributes to the formalization of nursing science’s knowledge in the field of self-care of people living with chronic diseases.

**References**

1. Peace J, Brennan P. Formalizing Nursing Knowledge: from theories and models to ontologies. Connecting Health and Humans. 2009; 347-351.

2. Pereira F, Silva A. Information technologies and nursing practice: the Portuguese case. In Nursing and informatics for the 21st century: an international look at practice, education and HER trends. Weaver C, Delaney P, Weber, et al. AMIA. 2010; 435-441.

3. Cruz I, Bastos F, Pereira F, Silva A, Sousa P. Analysis of the nursing documentation in use in Portugal – building a clinical data model of nursing centered on the management of treatment regimen. Nursing Informatics. 2016. 225; 407-411.

**Keywords**

Self-management, Knowledge, Nursing Diagnoses, Nursing Information Systems.

### O87 Illness perceptions, beliefs about medicines and medication adherence in hypertension

#### Teresa Guimarães, André Coelho, Anabela Graça, Ana R Fonseca, Ana M Silva

##### Escola Superior de Tecnologia da Saúde de Lisboa, Instituto Politécnico de Lisboa, 1990-096 Lisboa, Portugal

###### **Correspondence:** Teresa Guimarães (tguimaraes@estesl.ipl.pt)

**Background**

Hypertension constitutes the most prevalent modifiable cardiovascular risk factor and a major risk factor for cognitive decline and dementia. Antihypertensive medication is essential to minimize the consequences of the disease, stressing the need for a high adherence to treatment to achieve hypertension control. Illness perceptions and beliefs about medication have been identified as important determinants of treatment adherence.

**Objective**

To identify patients’ perceptions on hypertension and beliefs about antihypertensive medication and assess associations between these beliefs and medication adherence.

**Methods**

63 hypertensive patients, 69.8% females, 54-95 years (M = 69.02; SD=10.07), 96.8% diagnosed for more than one year and with antihypertensive medication prescribed completed the Revised Illness Perception Questionnaire (IPQ-R), the Beliefs about Medicines Questionnaire (BMQ-Specific) and a medication adherence measure (Medida de Adesão aos Tratamentos – MAT).

**Results**

Most of the patients perceived hypertension as a chronic (100%), cyclical (96.8%) condition, which can be controlled by medication (96.8%) and behaviour (90.5%), presented strong beliefs in the necessity of medication (96.8%), but also strong concerns about the consequences of taking it (87.3%). Patients reported a high level of adherence to medication (M = 5.41; SD = 0.55 on MAT, 7 as highest possible score) and low frequency of non-adherent behaviours. Significant positive correlations were found between necessity scale (BMQ) and hypertension timeline (acute/chronic) (rs(63)= 0.34; p < 0.01), treatment control (rs(63)= 0.43; p < 0.01), emotional representations (rs(63)= 0.50; p < 0.01) and between concerns scale (BMQ) and hypertension consequences (rs(63)= 0.26; p < 0.05); timeline (cyclical) (rs(63)= 0.46; p < 0.01), emotional representations (rs(63) = 0.32; p < 0.05). Significant negative correlations were found between concerns scale and personal (rs(63)= - 0.35; p < 0.01) and treatment (rs(63)= - 0.28; p < 0,05) control. We also found significant negative correlations between adherence (MAT) and hypertension timeline (cyclical) (rs(63)= -0.27; p < 0.05), consequences (rs(63)= -0.50; p < 0.01) and emotional representations (rs(62)= -0.37; p < 0.01).

**Conclusions**

Our findings suggest that illness perceptions play a key role in the way patients cope with their illness, through the development of patient’s beliefs concerning the necessity of medication and concerns about taking it, and also by directly influencing adherence to treatment. In our study, non-adherence is essentially unintentional (patients forget or are careless about treatment), what explains the lack of association between adherence and beliefs about medication, although we have found that, for the majority of subjects, concerns about taking medicines are outweighed by a belief in the necessity of the prescribed medication.

**Keywords**

Hypertension, Illness perceptions, Beliefs about medicines, Medication adherence.

### O88 Self-determined motivation and life satisfaction of elderly for the supervised physical activity practice

#### Marco Batista^1^, João Martinho^1^, Jorge Santos^1^, Helena Mesquita^1^, Pedro Duarte-Mendes^1^, Rui Paulo^2^

##### ^1^Polytechnic Institute of Castelo Branco, 6000-084 Castelo Branco, Portugal; ^2^Sport, Health and Exercise Research Unit, Polytechnic Institute of Castelo Branco, 6000-084 Castelo Branco, Portugal

###### **Correspondence:** Marco Batista (marco.batista@ipcb.pt)

**Background**

The self-determination theory suggests that humans have several basic psychological needs that are innate, universal and essential to health and well-being, namely, autonomy, competence and relation perception. The wellness construct, measured by satisfaction with life is understood as a judgment process in which individuals generally estimate the quality of their lives based on their own criteria.

**Objective**

This study has as main objective to identify the motivations, basic psychological needs and satisfaction with the life of the Portuguese elderly for the practice of supervised Physical Activity; and to analyse the relations and comparisons between levels of practice, sex and institutional context.

**Methods**

A cross-sectional study was carried out with 62 elderly volunteers of both sexes (15 males and 47 females), institutionalized and non-institutionalized, belonging to the Municipality of Castelo Branco, with a mean age of 79.61 ± 9.34 years. The instruments used were the Behavioural Regulation in Exercise Questionnaire, the Basic Psychological Needs Scale Exercise and the Satisfaction with Life Scale.

**Results**

The results show that, apparently, the motivation that maintains the constant practice of physical activity supervised by the elderly focuses on the autonomous motivation. It can also be observed that, except for the amotivation, where women have higher levels of amotivation for the practice of supervised physical activity than men, that is, they may be more exposed to an absence of motivational orientation, there are no differences at the level of the remaining motivational variables, as well as the basic psychological needs and life satisfaction between the male and the female. The results showed that, in the supervised elderly, the satisfaction of the basic psychological needs leads to autonomously motivated behaviours, promoting high levels of satisfaction with life.

**Conclusions**

We can conclude that autonomous motivation and the satisfaction perception of basic psychological needs are externalized as factors of great importance, because they appear to be a catalyst for this population to remain active and, in a way, to “compromise” with This lifestyle.

**Keywords**

Self-determination theory, Satisfaction with life, Exercise, Well- being, Elderly.

### O89 Application of the transcontextual model of motivation in the prediction of healthy lifestyles of active adults

#### Marco Batista^1^, Marta Leytón^2^, Susana Lobato^3^, Maria Aspano^3^, Ruth Jimenez-Castuera^3^

##### ^1^Polytechnic Institute of Castelo Branco, 6000-084 Castelo Branco, Portugal; ^2^Universidad Pablo de Olavide, 41013 Sevilla, Spain; ^3^Universidad de Extremadura, 06071 Badajoz, Spain

###### **Correspondence:** Marco Batista (marco.batista@ipcb.pt)

**Background**

The Transcontextual Model suggests an original contribution to knowledge, and can illustrate human behaviour, interpreting the theory of self-determination, contrasting with the hierarchical model of motivation, as well as with the theory of planned behaviour, seeking to predict behaviours. The strength of this model lies in the integration of different motivational theories, such that an explanation is a predicted complement to the motivational processes that are inexplicable in theory by each component.

**Objective**

The present study was designed with the objective of testing an extension of the Transcontextual Model of motivation in predicting healthy lifestyles of active adults.

**Methods**

The study sample consisted in 560 Portuguese active adults of both genders, aged between 30 and 64 years (M = 44.86; DP = 7.14). The instruments used were the Behavioural Regulation in Exercise Questionnaire, the Basic Psychological Needs Scale Exercise, Questionnaire for Planned Behaviour and the Healthy Lifestyle Questionnaire. A structural equation model was elaborated with which the predictive relations between the analysed variables were examined. The indices obtained in the measurement model were: χ^2^ = 527.193, p < .001; χ^2^/gl = 3.46; CFI = .94; IFI = .94; TLI = .93; GFI = .93; RMSEA = .60; SRMR = .43. The model goodness test showed the following adjustment indices: χ^2^ = 728.052, p < .001, χ^2^/gl = 4.79, CFI = .91; IFI = .91; TLI = .90; GFI = .91; RMSEA = .075; SRMR = .070.

**Results**

The structural equations model showed that the perception of social relation positively and significantly predicts autonomous motivation. In turn, this positively and significantly predicts control perception, predicting this, positively and significantly, the intentions. Eating habits and rest habits were positively and significantly predicted by intentions, and tobacco consumption was predicted negatively and significantly by intentions.

**Conclusions**

From the conclusions reached in this study, it is important to emphasize the importance of fostering social relations, since this will favour autonomous motivation, promoting a greater behavioural control over the intentions of the practitioners, thus generating more healthy eating habits, rest habits and lower consumption of tobacco.

**Keywords**

Theory of planned behavior, Self-determination theory, Structural equations models, Exercise, Lifestyles.

### O90 Symptoms management and adherence to antiretroviral medication - a nursing intervention

#### Eunice Henriques, Maria FM Gaspar

##### Escola Superior de Enfermagem de Lisboa, 1600-190 Lisboa, Portugal

###### **Correspondence:** Eunice Henriques (eunice.henriques@esel.pt)

**Background**

The increase of new diagnoses of HIV infection in young males, especially those who have sex with men, and the high percentage of late diagnoses, particularly in middle-aged heterosexuals continues to be our major concerns. As AIDS is now considered a chronic disease, its effective and sustainable treatment relies naturally on self-management of symptoms as well as promoting adherence to therapy. This will reduce costs and promote well-being in the person's life.

**Objective**

To develop a nursing intervention program to enhance effectiveness in managing symptoms and consequently adherence to antiretroviral therapy in the person with HIV/AIDS. Specific Objectives: Validate and adapt the following instruments: “Revised Sign and Symptom checklist for Persons with HIV disease”; “Self-care symptom management for people living with HIV/AIDS; AACTG (Adult Aids Trial Group) Instruments; “HIV/AIDS Stigma Instrument - PLWA (HASI-P) ©”; I) to assess the frequency and intensity of the most common signs and symptoms associated with HIV infection among participants, as well as the strategies used; II) to evaluate the adherence to antiretroviral therapy (ART); and III) evaluate self-perception of stigma.

**Methods**

This is a quasi-experimental, study with pre and post intervention evaluation. Participants were selected at the HCC Day Hospital; HIV-infected, multi-stage, more than 18-year-old, to have ART for at least 6 months. We carried out the sociodemographic characterization and validation of the instruments. The intervention consisted in the application of a strategy manual for self-management of the most frequent symptoms.

**Results**

The 1st Study sample consisted of 374 individuals, 74.1% were males. The age varied from 20 years to 78. The average with 47.34 years. Of the 64 presented symptoms, the number of symptoms ranged from 0 to 53 by the same participant. The mean was 18.96 symptoms per person. The most frequent symptoms were: anxiety, fatigue, fear and worries and depression. Most do not use strategies, or they are not effective in managing these symptoms. Of the total number of respondents, 30% never stopped taking the medication and the same number failed to take the therapy in the last 3 months, the main reason is simple forgetting.

**Conclusions**

Most participants do not adequately manage the symptoms due to lack of knowledge of the appropriate strategies, indicating devaluation based on the belief of inevitability (pain). Most say they adhere to ART (70%), making more than 95% of the shots, which is not always consistent with viral load.

**Keywords**

Nursing intervention, Symptoms management, Adherence to antirretroviral medication.

### O91 Aortic valve prosthesis: cardiovascular complications due to surgical implantation

#### Virginia Fonseca^1^, Ana Rita Bento^1^, Joana Esteves^1^, Adelaide Almeida^1,2^, João Lobato^1^

##### ^1^Escola Superior de Tecnologia da Saúde de Lisboa, 1990-094 Lisboa, Portugal; ^2^Hospital da Luz, 1500-650 Lisboa, Portugal

###### **Correspondence:** Virginia Fonseca (virginia.fonseca@estesl.ipl.pt)

**Background**

Aortic stenosis represents the third most common cause of cardiovascular disease, with indication for surgical valve replacement with a biological or mechanical prosthesis in most symptomatic patients. Biological prostheses present a higher risk of reoperation due to valvular degeneration; however, do not require prolonged therapy. In spite of the long durability, mechanics prosthesis need chronic anticoagulant therapy. The surgical intervention for valvular replacement is not free of complications and can be grouped into three main categories: prosthesis complications, non-prosthesis related cardiac complications and non-cardiac complications.

**Objective**

To characterize the cardiovascular complications resulting from the implantation of biological or mechanical aortic valve prosthesis, by surgical procedure.

**Methods**

32 patients were evaluated in four follow-up moments. Cardiovascular complications resulting from the implantation of a valve prosthesis (biological or mechanical) were analysed, and also the value of the pre and post-surgical mean gradient, symptoms and cardiovascular risk factors. All the variables under study were characterized by descriptive statistics, except the mean gradient variable, for which the Friedman test was used.

**Results**

The most frequent complication detected in individuals who implanted biological aortic prosthesis in the first (18.75%), second (21.88%), third (21.88%) and fourth (9.38%) follow-up moments were arrhythmias and electrical conduction disturbances. There is a higher prevalence of Atrioventricular Block and Left Bundle Branch Block, in the first and second follow up moments that reverts in the last two. The complication with higher prevalence in the sample of individuals with mechanical prosthesis at the first (9.38%) and second (3.13%) follow-up moments were arrhythmias. At the third and fourth follow-up moments, the main complication was paravalvular leaks (9.38%). Statistically significant differences were detected in the mean gradient throughout the follow-up (χ_F_^2^(4) = 12.122, p = 0.016).

**Conclusions**

The most frequent complications in individuals with aortic prosthesis were arrhythmias and paravalvular leaks. The structural deterioration of the biological valve prosthesis is the most commonly described complication, which may result in insufficiency of the valvular prosthesis and paravalvular leaks. However, electrical conduction disturbances after aortic valve replacement may occur through tissue manipulation of the conduction system. These disorders may be transient; however, certain patients require pacemaker implantation in the post-surgical period.

**Keywords**

Prosthetic valves, Aortic prosthesis complications, Biological prosthesis, Mechanical prosthesis.

### O92 A Safe staff nursing model: relationship between structure, process and result variables

#### Maria J Freitas^1^, Pedro Parreira^2^

##### ^1^Escola Superior de Enfermagem São Francisco das Misericórdias, 1169-023 Lisboa, Portugal; ^2^Escola Superior de Enfermagem de Coimbra, 3046-851 Coimbra, Portugal

###### **Correspondence:** Maria J Freitas (mjbsfreitas@gmail.com)

**Background**

The need to adequate nursing resources to the real needs of patients, while maintaining a balance between the quantity and the skills, without neglecting the quality and safety, has been a concern for managers. The absence of a consensual methodology to support the operationalization of a safe staff nursing, was the starting point of this investigation.

**Objective**

Develop an explanation Model Safe Staff Nursing (SSN) and analyse the relationships between the Structure, Process and Results.

**Methods**

Cross-sectional and correlational study. Data collection was achieved through a three-sample questionnaire: nurses (629), chief-nurses (43) and patients (1,290), from 43 units of 8 Portuguese hospitals. A patient’s form was applied to assess the satisfaction with nursing care. The data collection instrument for nurses and chief- nurses consisted of three parts: the first characterizing the personal and professional variables, the second featuring the healthcare organization and the service on which the respondent work, and the third part was to obtain information about: overall satisfaction at work (Evaluation Scale of Overall Satisfaction at Work [1]), intended to abandon the Work (Intent Abandonment of Employment Scale [2]), quality nursing care and risk/occurrence of adverse events (Adverse Events Associated with Nursing Practices Scale [3]). The psychometric assessment study of the measuring instruments, performed by Factorial Exploratory Analysis and Confirmatory demonstrated adequate validity and reliability. For model validation, we used the technique Structural Equation Modelling.

**Results**

The relational structure of the model is statistically significant (χ^2^(421) = 2209.095; p = 0.000; χ^2^/gl = 5.247; GFI = 0.833; CFI =0.815; RMSEA = 0.082), being adequate to explain the impact of Structure variables over Results of SSN, and the Process variables over Results. The “Availability of nurses with the right mix of skills”, “Availability of nurses in adequate amount” and “Safe environment” (Structure-variables) explain 2% of the variable variance of “Provision of quality nursing care” (Process-variables), 15% of the variance “Patient satisfaction”, 94% of the variance “Risk and occurrence of adverse events on patients” (Results-Patients), 25% of the variance “Results-Nurses” and 100% of variance “Results-Organization”.

**Conclusions**

The Safe Staff Nursing Model clearly identifies the influence of SSN on the results obtained for patients, nurses and organizations. Warns are given for the need to give more attention to issues of SSN, in particular to the constitution of balanced teams, based on a mix that includes number and competencies of nurses, versus workload/patients nursing care needs, as a strategy for maximizing resources and promoting the sustainability of organizations.

**References**

1. Silva CF, Azevedo MH, Dias MR. Estudo Padronizado do Trabalho por Turnos: Versão Experimental. Bateria de escalas. Serviço de Psicologia Médica, Faculdade de Medicina da Universidade de Coimbra. Coimbra; 1994.

2. Meyer JP, Allen NJ, Smith CA. Commitment to organizations and occupations: Extension and test of a three-component conceptualization. J Appl Psychol 1993;78(4):538-51.

3. Castilho A, Parreira PM [InterSnet]. Design and assessment of the psychometric properties of an adverse event perception scale regarding nursing practices. Revista Investigação Em Enfermagem 2012;1(2):61–75.

**Keywords**

Health care allocation resources, Nursing human resources, Safe nursing supplies, Safe Staff Nursing.

### O93 Engineered healthy food with nutritional and therapeutic advantages

#### Geoffrey Mitchell^1^, Artur Mateus^1^, Maria Gil^2^, Susana Mendes^2^

##### ^1^Centre for Rapid and Sustainable Product Development, Polytechnic Institute of Leiria, 2430-028 Leiria, Portugal; ^2^Marine and Environmental Sciences Centre, Polytechnic Institute of Leiria, 2520-641 Peniche, Portugal

###### **Correspondence:** Geoffrey Mitchell (geoffrey.mitchell@ipleiria.pt)

Direct Digital Manufacturing is an emerging set of technologies, which are able to produce complex objects without the need for molds or specific tooling. The preparation of food for human consumption is a centuries old craft, which results in food with high sugar and salt levels leading to a poor health for many people and not contributing to the recovery of seriously ill-hospitalized patients. One of the key challenges is to tailor food to the individual to match their dietary requirement and metabolic characteristics. We believe that direct digital manufacturing is able to address these issues. To date the use of 3D printing for food has been restricted to the production of aesthetic shapes for chocolate and pasta products. We see the potential in a different way in which we can tailor the food to the individual incorporating where required drug-based therapies whilst retaining the requirements to be visually pleasing. In addition, it will be possible to guarantee all the organoleptic characteristics to which the consumer in general is familiar (such as smell, sound and texture). The mouth is the gateway for food and its acceptance requires specific taste triggers. We consider that by exploiting Direct Digital Manufacturing it will possible to optimize such taste triggers whilst retaining the nutritional balance and potential. Every consumer (in general) has challenges in chewing and swallowing and engineered food has the capability to achieve this, especially when coupled with a model of the processes of the mouth and the oesophagus, parameterized to the individual. Once the food reaches the digestive system, all of the foregoing topics are largely irrelevant, other than to consider where the nutritional or therapeutic agents are extracted. The challenge of the oral intake of insulin is the best-known situation, but the delivery of chemotherapeutics is another area of challenges. It is necessary to shield the toxic therapeutic from the taste sensors and the digestive system so that it reaches the critical area of the digestive system intact. We are developing a comprehensive food design and manufacturing system that will allow each of these challenges to be met. We expect the first use of such system will be to expedite the recovery of seriously ill patients in hospitals. As enhanced testing procedures become more widely available through technological developments a wider use in the home is expected.

**Keywords**

Engineered Food, Nutritional, Therapeutic.

### O94 The influence of an eight month multicomponent training program in edlerlies gait and bone mineral mass

#### António M Monteiro^1,2^, Filipe Rodrigues^2,3^, Pedro Forte^2,3^, Joana Carvalho^4^

##### ^1^Department of Sports Sciences, Polytechnic Institute of Bragança, 5300-253 Bragança, Portugal; ^2^Research Center in Sports Sciences, Health and Human Development, University of Trás-os-Montes and Alto Douro, 5001-801 Vila Real, Portugal; ^3^Department of Sports Sciences, University of Beira Interior, 6201-001 Covilhã, Portugal; ^4^Research Centre in Physical Activity Health and Leisure, Faculty of Sport, University of Porto, 4200-450 Porto, Portugal

###### **Correspondence:** Filipe Rodrigues (ptfiliperodrigues@gmail.com)

**Background**

Aging induces neuromuscular changes and mass, strength, muscular resistance and power, motor coordination, so, reaction and movement speed reduction may be compromised [1]. These changes result in slower movements and functional limitations in gait and weight transfer activities [1]. Even more, the functional fitness decreasing due to aging, increases the risk of falls and bone fractures [2], reducing the elderly’s quality of life.

**Objective**

Thus, the aim of this study was to assess the influence of an eight months multicomponent training program in elderly’s gait and bone mineral mass (BMM).

**Methods**

Forty-nine elderlies were recruited for this research with 64.39 (± 6.33) years old, 11 males with 67.45 (± 4.93) and 38 females with 63.50 (± 7.47) years old. The subjects were community living persons of Bragança. All procedures carried out in this research were in accordance to the Declaration of Helsinki. The multicomponent training program followed the Carvalho et al. [1], recommendations. Each session time volume was between 50 to 60 minutes. The sessions were divided in five parts: 1) general warm-up; 2) walking with aerobic exercises; 3) 1 to 3 sets of exercises of muscular resistance with 12 to 15 repetitions; 4) Static and dynamic balance training; 5) An active recovery period with stretching and breading exercises. The elderlies gait was evaluated with Berg Balance Scale (BBS) and BMM with bioimpedance (Tanita, BC-545). The Wilcoxon-Mann-Whitney test allowed to assess the differences between pre and post 8 months of the training program in BBS and BMM. The tests were performed with a significant level of 5%.

**Results**

The BBS values pre and post the multicomponent training program for BBS were 47.33 and 50.33 respectively. In BMM, the pre and post values were 2.36kg and 2.39 kg. Despite the differences in BMM means, they were not significant between the two moments (F = 1.253; p = 0.706). However, the same did not occur in terms of BMS values (F = 1.967; p< 0.001), where gait values increased significantly in the second moment.

**Conclusions**

Although the multicomponent training program did not increase the BMM in the elderly subjects, gait values increased significantly. Thus, it is possible to conclude that, the training program significantly improved the elderlies’ gait and quality of life.

**References**

1. Carvalho J, Marques E, Soares JM, Mota J. Isokinetic strength benefits after 24 weeks of multicomponent exercise training and a combined exercise training in older adults. Aging Clin Exp Res. 2010;22(1):63-69.

2. Miyamoto ST, Lombardi Júnior I, Berg KO, Ramos LR, Natour J. Brazilian version of the Berg balance scale. Brazilian Journal of Medical and Biological research. 2004;37(9):1411-1421.

**Keywords**

Elderly, Bone, Gait, Multicomponent, Training.

### O95 Perception of virginity among Portuguese and Cape Verdeans university students – crossborder study

#### Sónia Ramalho^1,2^, Carolina Henriques^1,2^, Caceiro Elisa^1^, Maria L Santos^1^

##### ^1^School of Health Sciences, Polytechnic Institute of Leiria, 2411-901 Leiria, Portugal; ^2^Center for Innovative Care and Health Technology, Polytechnic Institute of Leiria, 2411-901 Leiria, Portugal

###### **Correspondence:** Sónia Ramalho (sonia.ramalho@ipleiria.pt)

**Background**

Virginity can be defined as the attribute of a person who has never been subjected to any type of sexual intercourse. To be aware of the sexual behaviour and virginity of young people is fundamental that nurses construct health education intervention programs in this specific area.

**Objective**

To know the perception of Portuguese and Cape Verdean university students about virginity.

**Methods**

A descriptive, cross-sectional study using a questionnaire consisting of sociodemographic data and the perception scale on the loss of the virginity by Gouveia, Leal, Maroco and Cardoso (2010) [1]. A sample composed by 108 young people from the Republic of Cape Verde and 141 young Portuguese participated in the study. All formal and ethical procedures were taken into account.

**Results**

Young Portuguese university students presented a mean age of 20 years and 73% of the young people reported having started their sexual life at 17.00 years old, on average. The majority of the young people (66.7%) started their sexual activity with their boyfriends, using protection/contraception (70.9%). Young college students from Cape Verde had a mean age of 21.26 years, 69.4% reported having started their sexual life, on average, at 17.37 years. The majority (63.0%) started their sexual activity with their boyfriend, using protection/contraception (62.0%). Portuguese young people showed high levels of agreement with the ideal associated with the genital vision of loss of virginity (Md = 18.95, X_max_ = 25.00, X_min_ = 11.00), while Cape Verdean students had lower levels of agreement (Md = 12.34, X_max_ = 24.00, X_min_ = 5.00), showing in 41.7% of the cases, disagreement that 'a lesbian woman, who has never had sex with a man, is virgin and a 38.0% disagreement with the statement that “men who only practice oral sex, or anal sex or other forms of sex, do not lose their virginity”.

**Conclusions**

The study shows that there is still considerable lack of knowledge in young people about the conceptualization of virginity and a very genitalized view of it in the Portuguese young people, in lower agreement with the perception of young Cape Verdeans.

**Reference**

1. Gouveia P, Leal I, Maroco J, Cardoso J. Escala de percepção sobre a perda da virgindade. In: Leal I, Maroco J, editors. Avaliação em sexualidade e parentalidade. Porto: LivPsic; 2010. p. 73-82.

**Keywords**

Young, Sexuality, Virginity, Portugal, Cape Verde

### O96 Influence of a specific exercise program in the institutionalized elderly balance

#### Cátia Guimarães^1^, Margarida Ferreira^1^, Paula C Santos^3^, Mariana Saavedra^2^

##### ^1^Institute of Research and Advanced Training in Health, Sciences and Technologies, Cooperativa de Ensino Superior Politécnico e Universitário, 4585-116 Gandra, Portugal^; 2^Hospital da Senhora da Oliveira, 4835-044 Guimarães, Portugal^; 3^Department of Physical Therapy, School of Health, Polytechnic Institute of Porto, 4400-330 Vila Nova de Gaia, Portugal

###### **Correspondence:** Margarida Ferreira (margasufer@gmail.com)

**Objective**

To determinate the effectiveness of a specific exercise program on balance and functional capacity of the daily activities of institutionalized elderly.

**Methods**

A randomized controlled trial. A total of 21 elderly were selected from the Santa Casa da Misericórdia de Santo Tirso and randomly distributed into experimental (n = 11) and control groups (n=10). The experimental group performed a specific program of exercises (resistance training, balance, coordination and flexibility) during 4 weeks, while the control group wasn’t subjected to any intervention. The primary outcome was balance, as measured with a Performance Oriented Mobility Assessment scale (POMA), and the secondary outcome measure included functional capacity by the Timed Up & Go test. Evaluations were carried out at the beginning and end of the exercise program, for both groups. The data were analysed with Statistical Package for Social Sciences, version 22.0, for all test procedures, a probability of p< 0.05 was considered to be statistically significant. Statistical analyses of POMA and TUG were performed with use of independent and paired t-test. POMA and TUG score association were analysed via the Pearson correlation, after the intervention.

**Results**

In the pre-intervention, groups were homogeneous (p < 0.05). After intervention, there were no statistically significant differences between groups in terms of the total balance and dynamic balance subscale, except static balance subscale (p < 0.048). In the functional capacity test, the experimental group reduced significantly the functional activity time into intragroup (p < 0.001), however there were no significant differences between groups (p < 0.633). After intervention, the experimental group had a significantly strong negative association (p = 0.001).

**Conclusions**

The results of this study demonstrated that this specific exercise program was not effective in terms of the total balance and functional ability of institutionalized elderly.

**Trial Registration**

NCT03521752

**Keywords**

Balance, Institutionalized elderly people, Therapeutic exercise, Functional capacity.

### O97 Assessment of pain and effectiveness of analgesia in patient undergoing haemodialysis

#### Luís Sousa^1,2^, Cristina Marques-Vieira^3^, Sandy Severino^2,4,^ Cristiana Firmino^2^, Ana V Antunes^2^, Helena José^5^

##### ^1^Hospital Curry Cabral, Centro Hospitalar Lisboa Central, 1069-166 Lisboa, Portugal; ^2^Escola Superior de Saúde Atlântica, 2730-036 Barcarena, Portugal; ^3^Escola de Enfermagem de Lisboa, Instituto de Ciências da Saúde, Universidade Católica Portuguesa, 1649-023 Lisboa, Portugal; ^4^Agrupamento de Centros de Saúde Loures-Odivelas, Administração Regional de Saúde de Lisboa e Vale do Tejo, 2685-101 Sacavém, Portugal; ^5^Instituto Superior de Saúde Multiperfil, Clínica Multiperfil, Luanda, Angola

###### **Correspondence:** Luís Sousa (luismmsousa@gmail.com)

**Background**

Pain is the most common symptom in patient’s undergoing haemodialysis, due to comorbidity, although it is frequently underdiagnosed [1-2]. Pain in these patients is not valued in its entirety and does not consider the limitations resulting in their quality of life [3]. The Brief Pain Inventory short form (SF-BPI) is the most widely used instrument and has the most number of foreign language translations [4].

**Objective**

To evaluate the prevalence of chronic pain, and intradialytic pain in patient undergoing haemodialysis, as well as the effectiveness of analgesic therapy.

**Methods**

Cross-sectional, descriptive and observational study. A random sample consisting of 172 patients undergoing haemodialysis in two clinics in the region of Lisbon, Portugal. The Brief Pain Inventory, which analyses the influence of pain in a patient’s life, was only applied to evaluate chronic pain [5]. The Visual Analogue Scale was used to assess the intradialytic pain. Tests were administered during dialysis sessions from May to June 2015. Categorical variables were expressed as percentages and continuous variables were expressed as mean standard deviations or medians. This study was approved by the Ethics Committee of Diaverum (N 1/2015).

**Results**

The sample consisted mostly of men (61.6%) of Portuguese nationality (80.7%), the mean age was 60 years (± 14.4), and patients were under haemodialysis treatment for 72.6 months (± 54.4). Chronic pain occurs in 54.1% of patients and intradialytic pain in 75%. The causes of pain were musculoskeletal (69.3%), associated to vascular access (19.3%) and other causes (11.4%). Chronic pain was most commonly located in the legs (43.2%), followed by back (21.6%) and vascular access (19.3), head (8%), arms (4.5%), abdomen (2.3%) and, lastly, chest (1.1%). The percentage of patients that took analgesics for chronic pain was much higher (62.0%), of these 87.8% are non-opiates, 10.2% weak opiates and 2% strong opiates. The other therapeutic interventions referred were: rest (24.1%), massage and relaxation (6.3%), cryotherapy (1.3%), exercise (1.3%), while 5.1% reported doing nothing. The effectiveness of the treatment was successful for chronic pain, in 62.6% of the patients, there was a relief felt of over 50%.

**Conclusions**

Pain of musculoskeletal origin is a frequent symptom in our sample. The pharmacological management of chronic pain is the most applied intervention.

**References**

1. Pelayo Alonso R, Martínez Álvarez P, Cobo Sánchez JL, Gándara Revuelta M, Ibarguren Rodríguez E. Evaluación del dolor y adecuación de la analgesia en pacientes en tratamiento con hemodiálisis. Enferm Nefrol. 2015;18(4):253-259.

2. Calls J, Rodríguez CM, Hernández SD, Gutiérrez NM, Juan AF, Tura D, Torrijos J. An evaluation of pain in haemodialysis patients using different validated measurement scales. Nefrologia. 2009;29(3):236-243.

3. Ahís Tomás P, Peris Ambou I, Pérez Baylach CM, Castelló Benavent J. Evaluación del dolor en la punción de una fístula arteriovenosa para hemodiálisis comparando pomada anestésica frente a frío local. Enferm Nefrol. 2014;17(1):11-15.

4. Upadhyay C, Cameron K, Murphy L, Battistella M. Measuring pain in patients undergoing hemodialysis: a review of pain assessment tools. Clin Kidney J. 2014;7(4):367-372.

5. Sousa LM, Marques-Vieira CM, Severino SS, Pozo-Rosado JL, José HM. Validación del Brief Pain Inventory en personas con enfermedad renal crónica. Aquichan. 2016;17(1):42-52.

**Keywords**

Renal Insufficiency, Chronic, Renal Dialysis, Quality of life, Pain.

### O98 Prevalence of musculoskeletal symptoms in nursing students

#### Cristiana Firmino^1,2^, Luís Sousa^2,3^, Joana M Marques^2,5^, Fátima Frade^2^, Ana V Antunes^2^, Fátima M Marques^4^, Celeste Simões^6^

##### ^1^Hospital Cuf Infante Santo, 1350-070 Lisboa, Portugal; ^2^Escola Superior de Saúde Atlântica, 2730-036 Barcarena, Portugal; ^3^Hospital Curry Cabral, Centro Hospitalar Lisboa Central, 1069-166 Lisboa, Portugal; ^4^Escola de Enfermagem de Lisboa, 1600-190 Lisboa, Portugal; ^5^Centro de Medicina de Reabilitação de Alcoitão, 2645-109 Alcabideche, Portugal; ^6^Faculdade de Motricidade Humana / Instituto de Saúde Ambiental, 1495-687 Cruz Quebrada, Portugal

###### **Correspondence:** Cristiana Firmino (furtado.cristy@gmail.com)

**Background**

Musculoskeletal symptoms are the most common conditions in society, being indicated as one of the main factors of disability during the life cycle of an individual [1-2]. Students are exposed to the factors that can trigger these musculoskeletal symptoms [3], both during class periods and clinical teaching. Prevalence of musculoskeletal pain is higher in the cervical region among nursing students of 1st year and 2nd year, and lower back in nursing students of the 3rd and 4th years [4].

**Objective**

To determine the prevalence of musculoskeletal symptoms in nursing students.

**Methods**

Cross-sectional and descriptive study. One hundred and fifty-five (155) nursing students from two nursing schools in Lisbon participated in this study. The data collection instrument consisted on sociodemographic and health behaviour variables and the Nordic musculoskeletal questionnaire (NMQ). The NMQ consists of 27 binary choice questions (yes or no) [5]. The variables were expressed as percentages. This study was approved by the Ethics Committee of two nursing’s schools.

**Results**

83.23% of the sample are females, single (88.38%) and 32.26% are working students. 81.94% are non-smoking; 87.1% do not usually ingest alcoholic drinks; 65.81% use a backpack and 23.23% carry objects on their way to school. 49.03% spend between 2 and 4 hours on the computer and electronic devices and 42.58% spend more than 4 hours. 71% spend more than 4 hours seated during classes. 85.8% had no training prevention of musculoskeletal injuries. The prevalence of musculoskeletal symptoms by location of the aches, pain, discomfort and numbness were as following: 66.23% in the neck; 52.29% shoulders; 7.24% elbows; 39.47% wrists/hands; 20.53% upper back; 69.33% lower back; 15.33% hips/thighs, 32% knees and 22.82% ankles/feet.

**Conclusions**

The most frequent aches, pain, discomfort, numbness location are located on the neck, shoulders and lower back. The main causes related to musculoskeletal injuries are the transportation of weights, use of computer and electronic devices and to be seated for long periods of time. It is recommended the implementation of prevention strategies in order to reduce the occurrence of musculoskeletal injuries.

**References**

1. Abledu JK, Offei EB. Musculoskeletal disorders among first-year Ghanaian students in a nursing college. Afr Health Sci. 2015;15(2):444-449.

2. Alhariri S, Ahmed AS, Kalas A, Chaudhry H, Tukur KM, Sendhil V, Muttappallymyalil J. Self-reported musculoskeletal disorders and their associated factors among university students in Ajman, UAE. Southern Med J. 2016;5(S2):S61-70.

3. Martins AC, Felli VE. Sintomas músculo-esqueléticos em graduandos de enfermagem. Enferm Foco. 2013;4(1):58-62.

4. Nunes H, Cruz A, Queirós P. Dor músculo esquelética a nível da coluna vertebral em estudantes de enfermagem: Prevalência e fatores de risco. Rev Inv Enferm. 2016;II(14):28-37.

5. Mesquita CC, Ribeiro JC, Moreira P. Portuguese version of the standardized Nordic musculoskeletal questionnaire: cross cultural and reliability. J Public Health. 2010;18(5):461-466.

**Keywords**

Nursing Students, Musculoskeletal Pain, Prevalence, Cross-Sectional Studies.

### O99 Eating habits: determinants of Portuguese adolescents’ choices

#### Susana Cardoso^1^, Carla Nunes^1^, Osvaldo Santos^2^, Isabel Loureiro^1^

##### ^1^Escola Nacional de Saúde Pública, Universidade Nova de Lisboa, 1600-560 Lisboa, Portugal; ^2^Instituto de Saúde Ambiental, Faculdade de Medicina, Universidade de Lisboa, 1649-028 Lisboa, Portugal

###### **Correspondence:** Susana Cardoso (suscardoso@yahoo.com.br)

**Background**

Proper eating habits are crucial to a healthy life. It is important to understand the determinants of eating choices made at adolescence because this stage of life is paramount for the formation of lifelong enduring habits.

**Objective**

To identify determinants of eating choices based on adolescents' perception and characterizing them, in particular, to the level of relevance attributed by adolescents.

**Methods**

A cross-sectional study was carried out, based on a sample of 358 adolescents (14-18 years old) from two schools of Coimbra. First, a quantitative study was carried out using the scales: EHA (eating habits scale), TAA-25 (eating attitudes test) and GSQ (general self-efficacy scale). In a second step, a qualitative study was carried out with subgroups that were selected from the results of the first phase of the study. These subgroups presented opposite patterns of habits (group A: better eating habits - EHA≥160 and group B: worst habits - EHA≤125) and we moved into a grounded theory approach with semi-structured individual interviews.

**Results**

Gender emerges as a determinant of eating choices pattern, with girls assuming more adequate eating habits (t =3.84; p <. 0001; r^2^adjusted= .037, p< .0001). The perception of general self-efficacy assumes greater relevance for boys, functioning as a protective factor that reduces unhealthy options. Through multinomial regression models, we could see that gender and general self-efficacy have a big influence on eating habits. The ideals of beauty have influence on this effect. Resisting adversity has an important influence in the choices, being associated to self-regulation. The situations of risk to develop an eating behaviour disturbance appear mainly in the cases of adolescents presenting better habits (r_SP_= .203; p < .001) and are more frequent in girls (t = 3.54; p < .0001; OR = 4.04). Through content analysis it was possible to identify determinant factors that were perceived by adolescents in both groups. The ones that were more often mentioned (in a decreasing order) were family influence, taste preferences, knowledge of healthy eating rules and availability. This was followed by determinants such as self-control capacity, feeling well or bad, peer influence, feeling hungry or full, developing a task or not, impulsiveness, time available and humour/stress.

**Conclusions**

The differences found between sexes can justify differentiated interventions. Our results also suggest the relevance to work on self-image. Family must be considered as an integrating part of the interventions in health education. Political measures taken by schools and government agents can also have a very important role in making healthy choices easier.

**Keywords**

Adolescents, Eating habits, Determinants.

### O100 The influence of regular sports practice on motor skills and student’s physical fitness

#### Júlio Martins^1,2^, João Cardoso^1^, José Reis^1^, Samuel Honório^3^

##### ^1^University of Beira Interior, 6201-001 Covilhã, Portugal; ^2^Research Center in Sports Sciences, Health Sciences and Human Development, 6201-001 Covilhã, Portugal; ^3^School of Education, Polytechnic Institute of Castelo Branco, 6000-266 Castelo Branco, Portugal

###### **Correspondence:** Samuel Honório (samuelhonorio@hotmail.com)

**Background**

Sports practice develops several motor skills that help practitioners not only in game, but also brings benefits to their physical fitness. Practitioners develop an effective motor response and quick solutions to daily situations.

**Objective**

Determine if the regular practice of sports influences or not motor skills, physical fitness and body composition of students. We wish also to identify the correlation between the performance of students in specific motor skills tests of football, physical fitness and body composition.

**Methods**

The sample consisted in 160 (divided in two groups of 80) male students, 12-year-old, living in Madeira and that practice football regularly. One group of students had an extracurricular physical activity beyond curricular physical activity (Federated Sports), and the other group entails students who only practice physical activities as a curricular activity (School Sport). Body composition (BMI and %BF) were also evaluated by Fitnessgram. To evaluate motor abilities the software Predictive Analytics Software – PASW was applied.

**Results**

After analysing the results, we became aware that students with extracurricular physical activities had better results in motor skills than students with curricular physical activities. This result is perhaps the less surprising in the set of all assessments due to the longer physical commitment of these students in relation to the students that only exercise in the context of curricular activities. Regarding physical fitness, students with extracurricular physical activity can have more students in HFZ (healthy fitness zone) than students with curricular physical activity. Regarding the arms extension and flexion, 26% of the students with curricular physical activity are above HFZ, achieving better performance than the students that have extracurricular physical activity. According to the skills measured, the students with extracurricular physical activities achieve a relatively higher average than students with curricular physical activities. Regarding body composition and after determining the BMI and %BF of each group, it appears that students with extracurricular physical activities achieve better results than those achieved by the students of curricular physical activities.

**Conclusions**

The regular practice of sports with curricular and extracurricular physical activities, seems to contribute to an improvement of physical fitness, motor skills and body composition of students, keeping them in a healthy fitness zone, thus preventing premature cardiovascular diseases, among others.

**Keywords**

Motor Skills, Physical Fitness, Body Composition, Federated Sports, School Sports.

### O101 Promotion of language skills in children aged 5-6 years, without language disorders

#### Tiago Rodrigues, Catarina Mangas

##### School of Education and Social Sciences, Polytechnic Institute of Leiria, 2411-901 Leiria, Portugal

###### **Correspondence:** Tiago Rodrigues (tiagoac.rodrigues@gmail.com)

**Background**

Language is a human faculty that enables communication with various interlocutors. This appears early and develops, exponentially, in the early years of life. For such, it is important that the surrounding environment is stimulating and allows the exchange of experiences with other speakers. The pre-school age is a milestone in the development, not only for stimulation, but also to identify deviant behaviours. In order to avoid future complications, professionals who deal with these children should prevent any language disorders. The speech and language therapist, being a health professional trained to deal with the areas of communication and language, has a prominent role in this preventive action. In Portugal, there aren't many references concerning prevention in language disorders. As such, it is necessary to promote a definite change in the present scenario, namely with innovative and stimulating materials to develop language.

**Objective**

Therefore, this is an exploratory-descriptive study, with a qualitative-quantitative paradigm and the general objective of this study was “*to create and implement a Language Skills Promotion Program for 5-6 year-old children without any language disorders, in order to analyse their potential influence on their language skills*”.

**Methods**

To this end, a 10-session program was build, evaluated and validated by a panel of experts. Before the beginning of the program, all the sample children were evaluated with a language test for preschool age. A sample of 12 children, divided equally in two groups, was selected and the program was applied by a Speech and Language Therapist only to one of these groups. At the same time, the Childhood Educator’s opinion was collected in order to understand the influence of the respective program on the children's language skills.

**Results**

The final results of the study show that children who participated in the program improved their language skills, which was not the case of the children who did not take part in that same study. These results prove that the investment in prevention actions, through the promotion of language skills, enhances the oral and written language skills of children, especially in terms of literacy, something that is indispensable for their educational success.

**Conclusions**

Finally, the study also emphasizes the importance of primary prevention actions, such as the application and development of programs to stimulate or organize information actions, with a view to promoting the health and the well-being of society in general.

**Keywords**

Child language, Child-rearing, Early intervention, Prevention, Speech therapy.

### O102 The impact of a training program on the performance of nurses working at a chemotherapy ward

#### Joana M Silva, Isabel M Moreira, Anabela Salgueiro-Oliveira

##### Nursing School of Coimbra, 3046-851 Coimbra, Portugal

###### **Correspondence:** Joana M Silva (joanamota19@gmail.com)

**Background**

Oral mucositis (OM) is the major complication reported by patients undergoing chemotherapy and/or radiotherapy, with a strong impact on their quality of life by compromising physical and psychological functions [1]. OM affects 40-76% of patients undergoing chemotherapy and up to 90% of patients undergoing radiotherapy [3,4]. Inadequate oral hygiene is a patient-related risk factor in which health professionals can intervene [5,7], namely nurses [6]. Oral examination allows diagnosing the different stages of OM and establishing an individualized care plan.

**Objective**

To assess the impact of a training program on the performance of nurses working at a chemotherapy ward regarding OM risk assessment and prevention in cancer patients.

**Methods**

This action-research study aimed to identify nurses' interventions in patients with or at risk for OM. Data were collected from the nursing records of 110 patients between October and November 2016 in order to analyse the nursing documentation pattern based on an evidence-based grid. Data were analysed using descriptive statistics. The discussion with the team nurses about the results obtained in the document analysis was used to design a three-session training program. The next step was to reanalyse the nursing documentation pattern with the purpose of identifying positive changes in the aspects under study. The study was approved by the Ethics Committee.

**Results**

The analysis of the documentation pattern showed that 31.8% of the patients were not asked about oral hygiene practices, although 25.7% of the sampled patients were in the 1st cycle of chemotherapy. A total of 21.9% of patients were not observed during oral hygiene care. Only 14.5% of patients were given instructions about the treatment of side effects, and only 12.5% of them were given instructions about oral hygiene care. Only 2.7% of the patients had their oral cavity/mucous membranes examined, and all of them were diagnosed with OM. The implementation of the training program led to the introduction of standardized records for oral cavity surveillance. Nurses showed high adherence levels to this practice and considered it very relevant in clinical practice.

**Conclusions**

The research results show that nurses do not perform a systematic diagnostic evaluation of patients’ oral cavity and that few patients receive instruction on oral hygiene care, which does not contribute to patient empowerment in this area. The implementation of the training program showed that nurses recognize the need for and are committed to changing practices in this area.

**References**

1. Yarbro CH, WujciK D, Gobel B H. Cancer Nursing: Principles and Practice. 8^th^ edition. Destin, Florida: Jones & Bartlett Publishers; 2016.

2. Eilers J, Harris D, Henry K, Johnson L. Evidence-based interventions for cancer treatment – Related mucositis: Putting evidence into practice. Clin J Oncol Nurs. 2016;18:80-96.

3. Peterson D, Boers-Doets C, Bensadoun R, Herrstedt J. Management of oral and gastrointestinal mucosal injury: ESMO Clinical Practice Guidelines for diagnosis, treatment, and follow-up. Annals of Oncology. 2015;26(Suppl 5):139-151.

4. Araújo S, Luz M, Silva G, Andrade E, Nunes L, Moura R. O paciente oncológico com mucosite oral: desafios para o cuidado de enfermagem. Rev. Latino-Am. Enfermagem. 2015;23(2):267-274.

5. Gondin F, Gomes I, Firmino F. Prevenção e tratamento da mucosite oral. Rev. enferm. UERJ. 2010;18(1):67-74.

6. Eilers J, Million R. Clinical update: Prevention and management of oral mucositis in patientes with cancer. Semin Oncol Nurs. 2011;27(4):e1-16.

7. Teixeira S. Mucosite oral em cuidados paliativos (Dissertação de mestrado em oncologia – Especialização em enfermagem oncológica). Universidade do Porto, Instituto de Ciências Biomédicas Abel Salazar, Porto, Portugal. 2010.

8. Kuhne GW, Quigley BA. Understanding and Using Action Research in Practice Settings. In Quigley BA, Kuhne GW, editors. Creating Practical Knowledge Trough Action Research: Posing Problems, Solving Problems, and Improving Daily Practice. San Francisco: Jossey-Bass Publishers. 1997. p. 23-40.

**Keywords**

Oral mucositis, Nursing care, Oncology.

### O103 Styles of conflict management and patient safety

#### Anabela Almeida^1^, Sara Cabanas^2^, Miguel C Branco^1^

##### ^1^Universidade da Beira Interior, 6200-001 Covilhã, Portugal; ^2^Centro Hospitalar Cova da Beira, 6200-251 Covilhã, Portugal

###### **Correspondence:** Anabela Almeida (aalmeida@ubi.pt)

**Background**

Health is a demanding scenario of changes and successive adaptations that, very easily, allows the emergence of differences between the involved and conflicts between professionals. Ineffectively managed conflicts in health organizations reduce quality, compromise safety, and increase the costs of health care delivery, secondarily to the goals of being effective and efficient.

**Objective**

Thus, the general objective of the study is to investigate the relationship between the conflict management styles used and the level of patient safety climate among the clinical services professionals at Hospital Pêro da Covilhã of CHCB.

**Methods**

The research is of a quantitative nature, of a descriptive and correlational character and of a transverse nature. The sample is non-probabilistic, consisting of 137 health professionals who work at CHCB. The use of ROCI-II and SAQ allowed the evaluation of the styles of conflict management and the perceptions of attitudes of health professionals related to patient safety.

**Results**

The results show that professionals, in all relations with the opponent, opt preferentially for collaboration, with competition being the least common style of conflict management. There were no differences in the styles of conflict management used in relation to the opponent. The participants presented positive attitudes towards the patient's safety, and it was verified that the professionals perceived a lower security relative to the dimension of management's perception and greater in relation to the dimensions of job satisfaction and recognition of stress, which show the highest values. The relationship between conflict management styles and the security climate level was verified. There is an association between literacy and conflict management styles, and years of service and conflict management styles. Regarding the ordinal independent variables, all are associated with the perceptions of the security climate.

**Conclusions**

Gender, marital status, integration period, function and years of service, influence the conflict management styles used; and age, gender, choice of service, integration period, function, area of service and years of service, influence the perceptions of professionals' attitudes related to patient safety.

**Keywords**

Conflict, Conflict management, Patient safety, Safety climate.

### O104 Influence of a rehabilitation nursing care program on quality of life of the patients undergoing cardiac surgery

#### José Moreira, Jorge Bravo

##### Department of Sports and Health, School of Science and Technology, University of Évora, 7000 Évora, Portugal

###### **Correspondence:** José Moreira (jafonsomoreira@gmail.com)

**Background**

Cardiac rehabilitation (CR) is fundamental in the treatment of patients undergoing cardiac surgery (CS) regarding the educational, physical exercise and quality of life dimensions. Considering the competences of Specialist Nurses in Rehabilitation Nursing (SNRN) and the current prevalence of risk factors associated with cardiovascular disease, it is essential to implement programs in this area.

**Objective**

To assess the impact of SNRN interventions on a CR program during hospitalization (phase I) and 1 month after CS (phase II).

**Methods**

Participants (n = 11) submitted to CS, of both sexes, between 25 and 64 years of age (61.09 ± 7.09 years), that according to the American Heart Association and the American Association of Cardiovascular and Pulmonary Rehabilitation, met the criteria for low or moderate risk, class B for participation and exercise supervision, absence of signs/symptoms after CS, with a left ventricular ejection fraction greater than 40%. Supervised interventions were performed during hospitalization, pre- and post-cardiac surgery, and 1 month after hospital discharge. In phase II, a physical exercise program was fulfilled according to the norms of the American College of Sports Medicine, comprising 3 sessions of physical exercise per week lasting between 30 to 60 minutes, including heating, aerobic exercise and recovery/stretching. Hemodynamic data (blood pressure, heart rate, peripheral oxygen saturation, pain) and the Borg scale were recorded in the initial, intermediate and final periods of each session. The aerobic capacity was evaluated through the 6-minute Walk Test and the health-related quality of life using the Short Form Health Survey 36 (SF-36V2) questionnaire.

**Results**

Significant statistical improvements were observed in the time/walk relationship, such as the increase in the respective functional capacity (p = 0.05) and quality of life (in various domains). During the hospitalization, the subjective perception of the effort of session to session decreased in 81.82% of the participants. T-test for independent samples revealed that differences in resting heart rate (phase I) were not significant, however, the difference in distances was significant at a 95% confidence level.

**Conclusions**

Nursing rehabilitation care is essential to improve the quality of life of patients undergoing CS in a phase I and II rehabilitation program. The benefits of CR programs are evident when initiated early after CC, reinforcing the need to increase their implementation in the rehabilitation of cardiovascular disease. Although the reduced sample size, the results represent a basis for future studies with a larger number of participants and a longer intervention period after CC.

**Trial Registration**

NCT03517605

**Keywords**

Cardiac Rehabilitation, Quality of Life, Rehabilitation Nursing.

### O105 Study of knee arthroplasty in the elderly population with agricultural activity

#### Carla Costa^1^, Jorge Nunes^2,3^, Ana P Martins^4^

##### ^1^Faculdade de Ciências da Saúde, Universidade da Beira Interior, 6200-506 Covilhã, Portugal^; 2^Universidade da Beira Interior, 6200-001 Covilhã, Portugal^; 3^Centro Hospitalar Cova da Beira, 6200-251 Covilhã, Portugal^; 4^Centro de Matemática e Aplicações, Universidade da Beira Interior, 6200-001 Covilhã, Portugal

###### **Correspondence:** Carla Costa (csofia_29@hotmail.com)

**Background**

Arthrosis is a major cause of pain, disability and loss of quality of life [1]. It affects the knee of elderly, overweight people and women frequently, and is influenced by articular overload, that occurs in Agriculture [2-9]. The majority of the individuals submitted to Total Knee Arthroplasty (TKA) refer significant decrease of knee pain and increase of knee functionality [1]. In this context, there are no studies on the recovery of elderly if they return to Agriculture after TKA.

**Objective**

Realize if elderly people between 65 and 80 years old, patients of Pêro da Covilhã Hospital, with Agricultural activity before surgery and submitted to TKA, with medial approach and posterior cruciate ligament sacrifice for the first time, can return to Agriculture and how long does it take; otherwise, identify the reasons for the interruption. Secondarily, analyse if Body Mass Index (BMI), gender, job, among others, influence this return.

**Methods**

This is an observational retrospective study with 38 patients between 65 and 80 years old submitted to TKA. Data was collected through clinical processes and patients self-report Western Ontario and McMaster Universities Osteoarthritis Index (WOMAC) and analysed on SPSS and R software (statically significant p < 0.05).

**Results**

Of the 38 patients, 76.3% were female. Average age was 72.21 ± 4.50 and 75.13 ± 5.01 years old at the time of TKA and at the time of the questionnaire, respectively. On both moments the majority of the individuals had overweight or obesity. 84.2% returned to Agriculture (81.2% partially and 18.8 % fully), on average 6.34 ± 4.90 months after TKA. The median age at the surgery of the seniors who didn’t return to Agriculture is superior to the one of the seniors who returned (p = 0.025). The higher score in Stiffness and the lowest total score on WOMAC was seen in the individuals who returned four or more months after TKA (p = 0.0125 and p = 0.026, respectively).

**Conclusions**

The majority of the individuals between 65 and 80 years old, with Agricultural activity before surgery and submitted to TKA with medial approach and posterior cruciate ligament sacrifice, can return to Agriculture, in 6 months. Most of them don’t return fully. The most cited reason was surgery consequences. The median age at the time of TKA of the seniors who didn’t return is superior to the one of the seniors who returned. A worst score in Stiffness and a better Total score was seen in the seniors who took longer to return to Agriculture.

**References**

1. Surgeons TAA of O. Artroplastia total de joelho (Total Knee Replacement ). OrthoInfo. 2017. p. 1–10.

2. Saúde DDA. Programa Nacional Contra as Doenças Reumáticas. Lisboa; 2005.

3. Silverwood V, Blagojevic-Bucknall M, Jinks C, Jordan JL, Protheroe J, Jordan KP. Current evidence on risk factors for knee osteoarthritis in older adults: A systematic review and meta-analysis. Osteoarthr Cartil. 2015;23(4):507–515.

4. Alfieri FM, Silva NCOVE, Battistella LR. Study of the relation between body weight and functional limitations and pain in patients with knee osteoarthritis. Einstein (São Paulo. 2017;15(3):307–12.

5. Toivanen AT, Heliövaara M, Impivaara O, Arokoski JPA, Knekt P, Lauren H, et al. Obesity, physically demanding work and traumatic knee injury are major risk factors for knee osteoarthritis-a population-based study with a follow-up of 22 years. Rheumatology. 2010;49(2):308–14.

6. Srikanth VK, Fryer JL, Zhai G, Winzenberg TM, Hosmer D, Jones G. A meta-analysis of sex differences prevalence, incidence and severity of osteoarthritis. Osteoarthr Cartil. 2005;13(9):769–81.

7. Pua YH, Seah FJ, Seet FJ, Tan JW, Liaw JS, Chong HC. Sex Differences and Impact of Body Mass Index on the Time Course of Knee Range of Motion, Knee Strength, and Gait Speed After Total Knee Arthroplasty. Arthritis Care Res. 2015;67(10):1397–405.

8. Liljensøe A, Lauersen JO, Søballe K, Mechlenburg I. Overweight preoperatively impairs clinical outcome after knee arthroplasty: a cohort study of 197 patients 3–5 years after surgery. Acta Orthop. 2013;84(4):392–397.

9. Kennedy JW, Johnston L, Cochrane L, Boscainos PJ. Total knee arthroplasty in the elderly: Does age affect pain, function or complications? Clin Orthop Relat Res. 2013;471(6):1964–1969.

**Keywords**

Knee, Arthrosis, Elderly, Agriculture, Arthroplasty.

### O106 Teachers Acceptance and Action Questionnaire Portuguese version (TAAQ-PT): factor structure and psychometric characteristics

#### Ana Galhardo^1,2^, Bruna Carvalho^1^, Ilda Massano-Cardoso^1^, Marina Cunha^1,2^

##### ^1^Instituto Superior Miguel Torga, 3000-132 Coimbra, Portugal; ^2^Cognitive and Behavioural Center for Research and Intervention, University of Coimbra, 3001-802 Coimbra, Portugal; ^3^Faculty of Medicine, University of Coimbra, 3004-504 Coimbra, Portugal

###### **Correspondence:** Ana Galhardo (anagalhardo@ismt.pt)

**Background**

Teaching has the potential to provide high satisfaction levels but it is described as a demanding profession with multiple sources of stress. Teachers’ psychological well-being is essential not only for themselves abut also for students. Experiential avoidance of private events (e.g., thoughts, feelings, body sensations) has been pointed as a key construct linked to psychopathological symptoms. The Teachers Acceptance and Action Questionnaire (TAAQ) is a teacher-specific measure developed to target experiential avoidance related to the teaching activity.

**Objective**

The current study sought out to develop the Portuguese version of Teachers Acceptance and Action Questionnaire (TAAQ-PT), explore its factor structure and psychometric properties in a sample of Portuguese teachers teaching in the 1st, 2nd and 3rd basic cycles and secondary education.

**Methods**

A sample of 304 teachers, 256 women (84.2%) and 48 men (15.8%) was recruited through teachers’ professional associations. Participants completed online a sociodemographic and professional questionnaire and a set of self-report instruments: the TAAQ-PT, the Depression Anxiety and Stress Scale 21 (DASS – 21), the Utrecht Work Engagement Scale (UWES), and the Five Facet Mindfulness Questionnaire (FFMQ). TAAQ-PT confirmatory factor analysis was conducted and reliability and validity were estimated.

**Results**

The TAAQ-PT revealed a single factor structure. Correlated measurement errors were specified for items 5 and 7, 3 and 10, and 8 and 9 due to similar phrasing. The one factor model, which specified method effects between those items, fits the data well: χ^2^/gl = 1.55, CFI = .99, GFI = .97, RMSEA = .043, MECVI = .321. The TAAQ-PT presented a Cronbach alpha of .91. Additionally, composite reliability (CR) was calculated, and a value of .95 was found. The TAAQ-PT presented significant negative correlations with mindfulness facets (r = -.60; p <.01), and work engagement (r = -62, p <.01), and positive correlations with negative emotional symptoms of depression (r = .69; p <.01), anxiety (r = .63; p <.01) and stress (r = .70; p <.01).

**Conclusions**

Similar to the original version, confirmatory factor analysis revealed that the single-component model fits the data well. It showed good internal consistency, and correlations with other mental health measures suggested good convergent and discriminant validity. The TAAQ-PT was found to be a valid and reliable measure of experiential avoidance in teachers to be used in clinical and research contexts.

**Keywords**

Experimental avoidance, Teachers, Confirmatory factor analysis, Psychometric properties.

### O107 Youth sports injuries according to health-related quality of life and parental instruction

#### Lara C Silva^1,2^, Júlia Teles^2,3^, Isabel Fragoso^1,2^

##### ^1^Laboratory of Physiology and Biochemistry of Exercise, Faculty of Human Kinetics, University of Lisbon, 1499-002 Dafundo, Portugal; ^2^Interdisciplinary Center for the Study of Human Performance, Faculty of Human Kinetics, University of Lisbon, 1499-002 Dafundo, Portugal; ^3^Mathematics Unit, Faculty of Human Kinetics, University of Lisbon, 1499-002 Dafundo, Portugal

###### **Correspondence:** Lara C Silva (laras@uatlantica.pt)

**Background**

Participation in physical activity involves a risk of injury that has a considerable public health impact [1]. Sports injuries are the major cause of morbidity among children and adolescents in developed countries [2]. They account for half of all injuries in school age children. The relationship between sports injuries, health-related quality of life (HRQoL) and parental instruction is still not clear.

**Objective**

Determine sports injuries biosocial predictors in Portuguese youth.

**Methods**

Information about HRQoL, parental instruction and sports injuries was assessed via three questionnaires; KIDSCREEN-52 [3,4], RAPIL II [5,6] and LESADO [1,7,8] respectively. They were filled by 651 subjects aged 10 to 18 years, attending four Portuguese community schools. Univariate analyses were used to verify significant differences between groups. Logistic, linear and multinomial regression analyses were used to determine significant biosocial predictors of injury, injury rate, injury type and body area injury location.

**Results**

Injury rate was higher in boys with lower scores in the school environment dimension of KIDSCREEN-52 (p = .022) and in girls was higher in those with lower scores in the moods and emotions dimension (p .001) and higher scores in the self-perception dimension (p < .001). Also in girls, upper limbs injuries were associated with higher scores in the moods and emotions dimension, and the spine and torso with lower scores (p = .037). Lower limbs injuries were associated with lower parents’ education and upper limbs (p = .046) and spine and torso (p = .034) injuries with higher parents’ education.

**Conclusions**

Surprisingly given the large number of injuries resulting from participation in sports and the associated high costs of health care, very few investigations have been conducted into biosocial variables and their relation to sports injuries. Injuries in the Portuguese youth were linked to three dimensions of KIDSCREEN-52 (moods and emotions, self-perception and school environment) and parents education level. Sports injuries usually result from the combination of several risk factors interacting at a given time [9]. Understanding the role of social and environmental factors related to sports injuries is needed, as they can be a part of this complex equation.

**Acknowledgements**

We would like to express our immeasurable gratitude to Ana Lúcia Silva and João Albuquerque for helping in data collection, and Carlos Barrigas for evaluating all x rays. We also thank to Escola Básica 2,3 Professor Delfim Santos, Agrupamento de escolas de Portela e Moscavide and Escola Secundária Quinta do Marquês, for making both their infrastructures and students available for the study and to all participants for their time and effort. Lara Costa e Silva, Ana Lúcia Silva e João Albuquerque were supported by a scholarship from the Portuguese Foundation for Science and Technology (SFRH/BD/77408/2011), (SFRH/BD/91029/2012), and PTDC/DES/113156/2009, respectively) and by the Interdisciplinary Center for the Study of Human Performance (CIPER).

**References**

1. Costa e Silva L, Fragoso I, Teles J. Prevalence and injury profile in Portuguese children and adolescents according to their level of sports participation. J Sports Med Phys Fitness. 2018 Mar;58(3):271-279.

2. Williams JM, Currie CE, Wright P, Elton RA, Beattie TF. Socioeconomic status and adolescent injuries. Soc Sci Med . 1997;44(12):1881–1891.

3. The Kidscreen Group. Description of the KIDSCREEN instruments. KIDSCREEN-52, KIDSCREEN-27 & KIDSCREEN-10 index. Health Related Quality of Life Questionnaires for Children and Adolescents. 2004. Report No.: EC Grant Number: QLG-CT-2000-00751.

4. Janssens L, Gorter JW, Ketelaar M, Kramer WLM, Holtslag HR. Health-related quality-of-life measures for long-term follow-up in children after major trauma. Qual Life Res. 2008;17(5):701–13.

5. Varela-Silva M, Fragoso I, Vieira F. Growth and nutritional status of Portuguese children from Lisbon, and their parents. Notes on time trends between 1971 and 2001. Ann Hum Biol. 2010;37:702–716.

6. Fragoso I, Vieira F, Barrigas C, Baptista F, Teixeira P, Santa-Clara H, et al. Influence of Maturation on Morphology, Food Ingestion and Motor Performance Variability of Lisbon Children Aged Between 7 to 8 Years. In: Olds T, Marfell- Jones M, editors. Kinanthropometry X Proceedings of the 10th Conference of the International Society for the Advancement of Kinanthropometry (ISAK). London: Routledge; 2007. p. 9–24.

7. Costa e Silva L, Fragoso MI, Teles J. Physical Activity–Related Injury Profile in Children and Adolescents According to Their Age, Maturation, and Level of Sports Participation. Sports Health. 2017;9(2):118–125.

8. Pires D, Oliveira R. Lesões no sistema musculo-esquelético em tenistas portugueses. Rev Port Fisioter no Desporto. 2010;4(2):15–22.

9. Powell J, Barber-Foss K. Injury patterns in selected high school sports: A review of the 1995-97 seasons. J Athl Train. 1999;34(3):277–84.

**Keywords**

Sports Injuries, Children and Adolescents, Health Related Quality of Life, Parental Instruction.

### O108 The influence of moderate- to vigorous-intensity activity on the physical fitness of non-institutionalised elderly people

#### Fernanda Silva^1^, João Petrica^1,2^, João Serrano^1,2^, Rui Paulo^1,3^, André Ramalho^1,3^, José P Ferreira^4^, Pedro Duarte-Mendes^1,3^

##### ^1^Department of Sports and Well-being, Polytechnic Institute of Castelo Branco, 6000-266 Castelo Branco, Portugal; ^2^Centro de Estudos em Educação, Tecnologias e Saúde, Instituto Politécnico de Viseu, 3504-510 Viseu, Portugal; ^3^Research on Education and Community Intervention, 4411-801 Arcozelo – Vila Nova de Gaia, Portugal; ^4^Research Unit for Sport and Physical Activity, University of Coimbra, 3040-248 Coimbra, Portugal

###### **Correspondence:** Fernanda Silva (f.m.a.s_298@hotmail.com)

**Background**

As a result of the ageing process, there is evidence of a decline in physical aptitude (strength, endurance, agility and flexibility) associated with a lower performance in the activities of daily living [1]. Physical activity plays therefore a key role in maintaining the health and physical fitness of the elderly [2]. The recommendations on physical activity for health suggest that the elderly should perform at least 30 minutes of moderate- to vigorous-intensity activity per day [3, 4].

**Objective**

The aim of this paper is to accurately quantify physical activity time in the elderly and to verify the existence of differences regarding physical fitness levels between two groups of people: those who complied and those who did not comply with the Global Recommendations on Physical Activity for Health [4].

**Results**

This cross-sectional study sample includes 36 elderly individuals (72.28 ± 6.58 years old), both male and female, divided into two groups: the group which has fulfilled the recommendations (N = 16; 53.76 ± 24.39 minutes) and the group that has not fulfilled the recommendations (N = 20; 15.95 ± 7.79 minutes). Physical activity was assessed for 3 consecutive days and 600 minutes of daily recording, at least. The ActiGraph® GT1M Accelerometer was hence used. The “Functional Fitness Test” battery (Rikli and Jones) was used to assess the physical and functional autonomy of the elderly [5]. In order to analyse data, descriptive and inferential statistics were used. The Shapiro-Wilk test was applied to assess normality, whereas the Mann-Whitney test and the t-Test were used for independent samples.

**Results**

On average, participants spent more time in sedentary activities than in physical activity. The group which has fulfilled the recommendations on physical activity has achieved better results on almost all physical fitness tests: 30 s chair stand (repetitions), arm curl (repetitions), 6-minute walk test (m), 8-foot up-and-go (s). However, no significant difference was found between the groups.

**Conclusions**

The results therefore suggest that only 44.4% of the evaluated participants complied with the Global Recommendations on Physical Activity for Health. Evidence also suggests that the adherence to these guidelines might have a positive influence on the physical fitness of the elderly, particularly muscular strength, endurance and agility, but not flexibility.

**Acknowledgements**

This work was supported by the Portuguese Foundation for Science and Technology (FCT; Grant Pest – OE/CED/UI4016/2016).

**References**

1. Tuna HD, Edeer AO, Malkoc M, Aksakoglu G. Effect of age and physical activity level on functional fitness in older adults. Eur Rev Aging Phys Act. 2009;6:99–106.

2. Nawrocka A, Mynarski W, Cholew J. Adherence to physical activity guidelines and functional fitness of elderly women, using objective measurement. Ann Agr Env Med. 2017;24:632-635.

3. WHO. Global Recommendations on Physical Activity for Health. Switzerland: World Health Organization; 2011. Available from: http://apps.who.int/iris/bitstream/10665/44399/1/9789241599979_eng.pdf

4. Department of Health. Start Active, Stay Active: A report on physical activity for health from the four home countries’ Chief Medical Officers. London: Department of Health; 2011. Available from: https://www.sportengland.org/media/2928/dh_128210.pdf.

5. Rikli R, Jones C. Development and validation of a funcional fitness test for community- residing older adults. J Aging Phys Activ. 1999;7:129-161.

**Keywords**

Physical fitness, Elder, Physical activity, Recommendation.

### O109 Effects of strength and conditioning programs in strength and dynamic balance in older adults

#### Rogério Salvador^1,2^, Luís Coelho^1,2^, Rui Matos^1,2^, João Cruz^1,2^, Ricardo Gonçalves^1,2^, Nuno Amaro^1,2^

##### ^1^School of Education and Social Sciences, Polytechnic Institute of Leiria, 2411-901 Leiria, Portugal; ^2^Life Quality Research Centre, 2001-904 Santarém, Portugal

###### **Correspondence:** Rogério Salvador (rogerio.salvador@ipleiria.pt)

**Background**

To independently accomplish their daily routines with no need of assistance, older adults require an optimal physical fitness. In fact, this lack of physical fitness may reduce older individuals’ quality of life, leading to dependence on personal daily assistance or even to becoming significantly more prone to fatal falls [1]. Prevention through physical activity programs, are used to slow down and delay these aging effects, by improving individuals’ agility, flexibility and body improved functionality. Most of these programs take place in in-water environment due to age limiting factors such as high-risk osteoporosis, reduced mobility, higher risk of fracture from falls, arthrosis and spinal disorders among other.

**Objective**

To assess the effects of two strength and conditioning programs in strength and dynamic balance in older adults.

**Methods**

One hundred elderlies (36 males and 64 females) aged 67.3 ± 5.2 years old enrolled the 5-year long intervention program and were assessed for lower body strength (LBS) and dynamic balance (DB). Two intervention programs were set up and subjects were included in each group according to their own will. Program A (n = 52; 24 males and 28 females; age 67.2 ± 5.2 y-o) consisted of 1 in-water session and 2 in dry-land sessions per week. Program B (n = 48; 12 males and 36 females; age 67 ± 5.2 y-o) consisted of 2 in-water sessions and 1 in dry-land session per week. Wilcoxon test was used on inferential analysis for repeated measures (pre-post). Significance level was kept at 5%. The effect size for this test was calculated by dividing the z value by the square root of N [2].

**Results**

Combined data from both programs showed that LBS and DB improved significantly at the end of the intervention programs: LBS from 18.3±3.2 reps to 18.8±3.1 reps (p=0.003; r=-0.295), DB 4.2±0.7 secs to 4.0±0.7 secs (p=0.017; r=-0.245). Program A significantly improved LBS from 19.1±2.8 reps to 19.9±2.7 reps (p=0.001; r=-0.465) but not DB 4.1±0.7 secs to 4.0±0.7 secs (p=0.083; r=-0.240). No differences were found neither in Program B LBS – 17.5±3.4 reps to 17.6±3.1 reps (p=0.462; r=-0.106) – nor DB - 4.2±0.6 secs to 4.1±0.6 secs (p=0.083; r=-0.250).

**Conclusions**

Strength and conditioning programs over a 5-year time span seem to substantially delay the negative effects of aging on LBS/DB in the elderly. No visible decline in the assessed parameters was observed. Our results may suggest different effects of in-water and dry-land programs. However, participants generally responded positively to both intervention programs.

**References**

1. World Health Organization. Falls Fact Sheet. Updated August 2017. http://www.who.int/mediacentre/factsheets/fs344/en/

2. Rosenthal R. Parametric measures of effect size. In: Cooper H, Hedges LV, editors. The handbook of research synthesis. New York: Russell Sage Foundation; 1994. p. 231-244.

**Keywords**

Elderly, Physical activity, Quality of life, Strength, Balance.

### O110 Compassion attributes and actions in adolescents: are they related to affect and peer attachment quality?

#### Marina Cunha^1,2^, Cátia Figueiredo^1^, Margarida Couto^1^, Ana Galhardo^1,2^

##### ^1^Instituto Superior Miguel Torga, 3000-132 Coimbra, Portugal; ^2^Cognitive and Behavioural Center for Research and Intervention, Faculty of Psychology and Educational Sciences, University of Coimbra, 3001-802 Coimbra, Portugal

###### **Correspondence:** Marina Cunha (marina_cunha@ismt.pt)

**Background**

Research has been showing potential benefits of compassion practice in various populations, nonetheless it is relevant to extend the assessment of compassion attributes and actions for adolescents and explore its relationship with other psychosocial adjustment constructs.

**Objective**

To explore association patterns between the various directions of compassion (self- directed, directed to others and receiving compassionate from others) and variables related to affect, social comparison and peers’ attachment to quality.

**Methods**

A total of 338 adolescents, aged between 12 and 18 years old, completed a set of self-report instruments to assess their compassionate attitudes and actions towards themselves and others (EAAC), peers attachment to quality (AQ-C), positive and negative affect (PANAS), and peers social comparison (SCS-A).

**Results**

Significant correlations were found in the expected direction between self-compassion, compassion for others and received from others and the study variables (positive and negative affect, social comparison and attachment style). Specifically, positive affect, positive peer comparison, and secure attachment style were positively associated with compassionate attributes and actions. Negative affect, in turn, showed a negative correlation with compassionate actions in the three analysed directions, and with compassionate attributes when considering receiving compassion from others. The avoidant unsecure attachment style revealed a negative association with compassionate attributes and actions in the different directions. Finally, the ambivalent insecure attachment style revealed a significant negative correlation with self-directed compassionate actions and with receiving compassion from others, regarding actions and attributes.

**Conclusions**

These findings suggest the importance of stimulating a compassionate mind in adolescents. In fact, the positive association between compassion and psychological and emotional adjustment variables point to the relevance of developing compassion skills during this developmental stage.

**Keywords**

Compassion attributes, Compassion actions, Adolescents, Positive and negative affect, Peer attachmet

### O111 Association palmar grip strength with self-reported symptoms in the arm

#### Alice Carvalhais^1^, Tatiana Babo, Raquel Carvalho^1^, Paula Rocha, Gabriela Brochado^1^, Sofia Lopes^1,2^

##### ^1^Department of Technology Physiotherapy, Cooperativa de Ensino Superior Politécnico e Universitário, Polytechnic Institute of Health, 4585-116 Paredes, Portugal; ^2^Department of Physical Therapy, School of Health Technology, Polytechnic Institute of Porto, 4200-465 Porto, Portugal

###### **Correspondence:** Gabriela Brochado (gabriela.brochado@ipsn.cespu.pt)

**Background**

World Health Organization (WHO) defined work-related musculoskeletal injuries as multifactorial diseases. These injuries are the main concern of public health and individual health, and are becoming increasingly frequent, in both developed and developing countries. Workers during working hours are often exposed to repetitive movements, the lifting and carrying heavy loads, verifying an increase in demand in terms of muscle strength in the upper limbs. The palmar grip strength provides an objective index of the functional integrity for the evaluation of upper limbs.

**Objective**

Verify that the palmar grip strength is associated with self-reported symptoms in the arm in industry worker’s electrical components.

**Methods**

An observational, analytical study was performed on a sample of 167 workers. The Nordic Musculoskeletal Questionnaire was applied and the palmar grip strength was measured using the hydraulic dynamometer. Descriptive statistics were used to analyse the prevalence of self-reported symptoms and the U test of Mann-Whitney, Kruskal-Wallis H test, Chi-square test and Fisher's exact test was used to analyse relationships between variables, with a 95% confidence level.

**Results**

The palmar grip strength was related to self-reported symptomatology in the dominant upper limb, shoulder regions (p = 0.018) and wrist (p = 0.005) in females. It was also found that the risk factors are not associated with palmar grip strength in individuals of both genders.

**Conclusions**

Palmar grip strength is associated with self-reported symptomatology in the shoulder and wrist of the dominant upper limb in female workers.

**Keywords**

Dynamometer, Palmar grip strength, Upper limb, Symptomatology auto referred.

### O112 Social-skills as facilitators of a healthy lifestyle

#### Luisa Aires^1,2^, Sara Lima^3^, Susana Pedras^3^, Raquel Esteves^3^, Fátima Ribeiro^3^, Assunção Nogueira^3^, Gustavo Silva^1,4^, Teresa Herdeiro^3^, Clarisse Magalhães^3^

##### ^1^Instituto Universitário da Maia, 4475-690 Maia, Portugal; ^2^Centro de Investigação em Atividade Física Saúde e Lazer, Universidade do Porto, 4099-002 Porto, Portugal; ^3^Cooperativa de Ensino Superior Politécnico e Universitário, Polytechnic Institute of Health, 4585-116 Paredes, Portugal; ^4^Research Center in Sports Sciences, Health Sciences and Human Development, University of Beira Interior, 6201-001 Covilhã, Portugal

###### **Correspondence:** Luisa Aires (luisa.aires@gmail.com)

**Background**

The Knowledge of behaviours and social-skills of adolescents can contribute to the construction of an effective school-based intervention to promote healthy lifestyles.

**Objective**

Identify homogeneous groups (clusters) according to lifestyle and social skills.

**Methods**

This cross-sectional study included 1,008 students from 5 elementary schools of Tâmega and Sousa region, mean age of 13.43 (SD = 1.1) and 50% of girls. A sociodemographic questionnaire “My Lifestyle” was used with 28 items composing 4 subscales: Physical Exercise (PE), Nutrition, Self-Care, Monitored Safety, Use of Drugs and Similar (UDS) (0.41< α <0.85). A “*Social Skills Inventory for Teenagers*” questionnaire (Social-Skills) was applied, including subscales: Empathy, Civility, Assertiveness, Self-Control, Affective Approach and Social-Development (0.64< α <0.90). Both questionnaires had 5 categories of answers from “almost always” to “almost never” or “rarely”. In order to identify homogeneous groups of students, according to lifestyle and social skills, it was performed a k-means cluster analysis

**Results**

For Lifestyle, mean scores were: UDS = 4.09, Self-Care = 4.07, PE = 3.86, Monitored Safety = 3.63 and Nutrition = 3.40. For Social-Skills, 50.7% had a highly elaborate repertoire of Social Skills, 11% had elaborate repertoire, 20.1% had good repertoire and 2.7% had lower average of social skills repertoire. It was decided to follow a three-cluster solution. Cluster 1 included students with a poor elaborated repertoire of social skills, but with good lifestyle indicators in all subscales. In cluster 2, students had a good repertoire of social skills, with good lifestyle indicators in all subscales, except for subscales of nutrition with poor indicators (38.7) and Monitored Safety (46.95). Cluster 3 included students with highly developed repertoire of social skills and the best lifestyle indicators.

**Conclusions**

Results revealed healthy practices in general, however students had the lowest scores in Nutrition, especially in sugar intake and absence of dietary plan. Students included in cluster 2 presented also the lowest results in Monitored Safety, especially about driving with alcohol. These students at risk of develop unhealthy lifestyle need special attention. The high profile of social skills in particular Affective Approach and Assertiveness, should be taking into account as a mechanism for intervention programs. In addition, relevance given to PE, should also be used as a good strategy to reinforce the accomplishment of healthy eating habits in all students. In another point of view, good indicators of lifestyles (cluster 1) can act as matrix to reinforce improvements in social-skills.

**Keywords**

Adolescents, Lifestyle, Social Skills.

### O113 Palliative care: nursing student’s conceptions and motivations

#### Suzana Duarte, Vitor Parola, Adriana Coelho

##### Escola Superior de Enfermagem de Coimbra, 3046-851 Coimbra, Portugal

###### **Correspondence:** Suzana Duarte (susanafcduarte@gmail.com)

**Background**

Palliative care (PC) is an inevitability in view of the demographic and epidemiological transition curves of Western society. The inclusion of a PC Curricular Unit (CU) in the Nursing Undergraduate Program (NUP) translates into the acquisition of competencies that allow caring for people and families in need of those carefulness. Although considering professional, institutional and family barriers, there is evidence that students apply, in clinical practice, the principles inherent of PC [1]. During clinical education, students are confronted with persons in need of PC, however without benefiting from such care. These experiences can form the basis, from which, it is possible to build the teaching-learning process of future nurses, regarding this theme.

Objective

To identify the conceptions and motivations for the frequency of the CU option of PC, by nursing undergraduate students'.

**Methods**

In the first class, nurses’ students were asked to anonymously write what they understood as PC and the motivation for attending this CU. The 210 responses collected over 5 years were subjected to content analysis [2].

**Results**

The PC conceptions reported were grouped into the categories: “Care for people in the final stages of life”, “Care to alleviate suffering” and “Comfort care”. The reasons for choosing the PC option were grouped in “Difficult and not tackled area”, “Area that arouses more interest” and “Previous Experiences”. It is verified that the concept of PC remains as care for people in terminal phase of life and in suffering. Some students report experiencing situations that would lead to PC, conditions of therapeutic obstinacy and end of life in circumstances of intense suffering. Students also mention the nurses ‘attempts to provide those carefulness, which is not well favoured for, in the hospital wards. Students indicate interventions that are intrinsic to palliative care, such as, communication, psychological support, coping with death and mourning, without any reference to the need for knowledge in other areas, namely pathology, pharmacology, or maintenance and healthcare technologies. The orientation of care for quality of life, family integration and management of symptoms is not considered.

**Conclusions**

There is a need to include in each NUP a PC CU, preferably after a period of clinical education in hospital wards. In this way it is possible to consider the previous experiences of the students, capitalizing them to the understanding of the fundamental principles of Palliative Care.

**References**

1. Bassah N, Cox K, Seymour J. A qualitative evaluation of the impact of a palliative care course on preregistration nursing students’ practice in Cameroon. BMC Palliat Care. 2016;15(1):37.

2. Bardin L. Análise de Conteúdo. 6th edition. Edições 70; 2013.

**Keywords**

Palliative Care, Nursing student’s, Motivations and conceptions.

### O114 “As eat” effects of a physical exercise program and nutrition in obese and binge eating adults

#### Ana Barroco^1^, José A Parraça^1^, Nuno Pascoa^1^, Daniel Collado-Mateo^2^, Jose Adsuar^3^, Jorge Bravo^1^

##### ^1^Department of Sports and Health, School of Science and Technology, University of Évora, 7000 Évora, Portugal; ^2^Instituto de Actividad Fisica y Salud, Universid Autonoma de Chile, Providencia, Chile; ^3^Universidad de Extremadura, 06006 Badajoz, Spain

###### **Correspondence:** José A Parraça (jparraca@uevora.pt)

**Background**

Overweight and Obesity are defined as an abnormal or excessive fat accumulation and present a health risk. Binge eating is a food disorder characterized by episodes of abusive food intake in the absence of regular compensatory behaviours such as vomiting or abuse of laxatives. Those who suffer from this disorder often increase their weight and fat mass by excessive intake of calories, thus becoming overweight or obese.

**Objective**

To determine the relation between the effects of an exercise and nutrition program, in overweight or obese adults (30-60 years) and binge eating, regarding body composition and physical fitness. The program also aimed to promote learning and self-control in the practice of physical activity and in the food choices of this population.

**Methods**

41 patients from USF Planície de Évora. Groups were randomly assigned: the experimental group (N = 23) and the control group (N = 18). The study lasted eight months and consisted of 47 practical sessions of one-hour group exercise, twice a week, one weekly self-help session, and three sessions of nutritional monitoring throughout the program. Practical sessions were structured with specific exercises aimed at improving the different components evaluated; namely in physical fitness (strength, cardiovascular endurance and flexibility) and body composition (fat loss).There were significant improvements in body composition, namely in the percentage of fat mass (40.75 (±6.46) to 37.44 (±7.06) p = .000), fat free mass (59.98 (±6.44) to 62.26 (±7.56) p = .001), of fat mass in the trunk (35.95 (±4.90) to 32.06 (±4.93) p = .000), in the Visceral index (12.00 (±3.42) to 10.88 (±2.97) p = .000) and in metabolic age (59.88 (±9.35) to 55.94 (±7.92) p =. 024). There were improvements in physical fitness, mainly in trunk flexibility (-0.18 (±9.72) to 8.93 (±10.06) p = .002) and in leg strength (0.10 (±0.03) to 0.13 (±0.02) p = .034) and arms (25.48 (±8.91) to 30.81 (±7.68) p = .000). Regarding weight, there is a tendency to significance, since there was a significant improvement (92.25 (±12.73) to 88.93 (±13.77) p = .056).

**Conclusions**

We conclude that the physical exercise and nutrition program allows improvements in physical fitness and body composition in the obese population suffering from binge eating.

**Keywords**

Exercise, Nutrition, Obesity, Body composition, Food addiction.

### O115 Exploratory analysis of the association between motives for the practice of physical exercise and body composition

#### Roberta Frontini^1^, Maria Monteiro^2^, António Brandão^2^, Filipe M Clemente^2,3^

##### ^1^Center for Innovative Care and Health Technology, Polytechnic Institute of Leiria, 2411-901 Leiria, Portugal; ^2^School of Sports and Leisure, Polytechnic Institute of Viana do Castelo, 4900-347 Viana do Castelo, Portugal; ^3^Instituto de Telecomunicações, University of Beira Interior, 6201-001 Covilhã, Portugal

###### **Correspondence:** Roberta Frontini (roberta_frontini@hotmail.com)

**Background**

Understanding what reasons lead individuals to start and maintain physical activity is extremely important to help individuals to engage and adhere to physical exercise. It allows exercise professionals to define the most appropriate actions to implement more suitable strategies and remove possible barriers to exercise. The decrease in body fat mass may be indirectly related to the motivation that the individual has for the practice of the physical exercise. Higher levels of motivation to lose weight may be related with higher adherence to, for example, the training plan and, consequently, be related to higher levels of body mass fat reduction.

**Objective**

This study aimed to analyse the association between body fat and motives to practice physical exercise.

**Methods**

The sample comprised 85 adults (38 males and 47 females) attending the gym, who completed a sociodemographic form and the Exercise Motivations Inventory - 2 (EMI-2). A multiple regression analysis was used to predict the value of a %body fat (%BF) based on the value of survey categories. The significance was set at p < 0.05. The statistical procedures were made in SPSS software (version 23.0, IBM, USA).

**Results**

A multiple regression analysis was run to predict %BF of the participants from social recognition, positive health, weight management, stress management, revitalization, enjoyment, challenge, affiliation, competition, health pressures, health avoidance, appearance, strength and endurance and nimbleness categories. These variables statistically significantly predicted %BF, F(14.70) = 2.249, p < 0.014, R^2^ = 0.310. Only three variables (social recognition, positive health and weight management) added statistically significance to the prediction, p < 0.05. The unstandardized coefficient, B1, for social recognition is equal to 2.178, for positive health is equal to 4.860 and for weight management is equal to 2.490.

**Conclusions**

Social variables (specifically social recognition), positive health and weight management are important for body mass fat reduction, more than variables related, for example, with health concerns. It is important, in future studies, to understand what processes influence those relations. The results of our study reinforce the importance of these three variables for the reduction of body fat mass emphasizing that it may be important to take these issues into account not only to maintain the adherence of individuals, but also to promote the practice of physical exercise.

**Keywords**

Motivation, Physical exercise, Body fat, Social recognition, Positive health.

### O116 Simulation as a pedagogical strategy in nursing teaching

#### Cláudia Chambel^1^, Catarina Carreira^1^, Catarina Pinheiro^1^, Luís Ramos^1^, Catarina Lobão^1,2^

##### ^1^School of Health Sciences, Polytechnic Institute of Leiria, 2411-901 Leiria, Portugal; ^2^Center for Innovative Care and Health Technology, Polytechnic Institute of Leiria, 2411-901 Leiria, Portugal

###### **Correspondence:** Luís Ramos (luis_filipe-ramos@hotmail.com)

**Background**

Nowadays, the use of laboratories with specific equipment and classes with resource to simulated practice, are increasingly advocated, especially in graduation courses, whose practice is a crucial tool for students to apply in real life situations.

**Objective**

Therefore, we intended to know the perception of students and teachers of a nursing degree, on the use of simulated practice as a pedagogical strategy.

**Methods**

To achieve this, we developed a research study using a qualitative approach and a semi-structured interview applied to six students of the nursing graduation course and to seven teachers who teach classes, with the resource of simulated practice, at Escola Superior de Saúde de Leiria.

**Results**

From the results we verified that for teachers, simulation is a pedagogical strategy in the development of the students’ competences, in a way that will translate in a provision of care based on the scientific knowledge, safety and humanism that is expected from a health professional. However, from the students’ perspective, we verified that the results indicate that the simulation is undoubtedly an added value, since the interviewees were able to approach the concept of simulated practice at several levels, also enabling to highlight the partnership between the pertinence and the contributions of the simulation and, finally, to mention several constraints and respective solutions.

**Conclusions**

As Goostone et al., (2013) [1] states, simulation is a pedagogical strategy that allows the student to acquire skills necessary for clinical practice, in a risk-free real environment, that is, students are faced with a clinical situation like what they would find in a real clinical environment, receiving feedback on their performance. Thus, it’s fundamental that there are teachers with the necessary training to implement this type of pedagogical strategy, as well as the necessary resources, associated to the will and commitment of the students. This triad is essential for the development of the students’ competencies as future professionals. In summary, the groups interviewed highlighted the importance of simulation, being able to answer to our research questions, complementing each other, once they recognized the importance of simulation in the health field.

**References**

1. Goodstone L, Goodstone M, Cino K, Glaser C, Kupferman K, Dember-Neal T. Effect of Simulation on the Development of Critical Thinking in Associate Degree Nursing Students. Nurs Educ Perspect. 2013;34(3):159-62.

**Keywords**

Simulation, Nursing, Education.

### O117 Optimising protocol using dual in-situ hybridization to breast cancer in HER2 status

#### Paulo Teixeira^1,2,3^, Maria F Silva^1,2^, Paula C Borges^1^, José M Ruivo^2^, Diana Martins^4^, Fernando Mendes^1,3,5,6^

##### ^1^Department Biomedical Laboratory Sciences, Coimbra Health School, Polytechnic Institute of Coimbra, 3046-854 Coimbra, Portugal^; 2^Pathologic Anatomical Service, Centro Hospitalar e Universitário de Coimbra, 3000-075 Coimbra, Portugal^; 3^Biophysics Institute, CNC.IBILI, Faculty of Medicine, University of Coimbra, 3000-354 Coimbra, Portugal^; 4^Instituto de Investigação e Inovação em Saúde, University of Porto, 4200-135 Porto, Portugal^; 5^Center of Investigation in Environment, Genetics and Oncobiology, Faculty of Medicine, University of Coimbra, 3001-301 Coimbra, Portugal^; 6^Coimbra Institute for Clinical and Biomedical Research, University of Coimbra, 3004-504 Coimbra, Portugal

###### **Correspondence:** Paulo Teixeira (paulo.teixeira@estescoimbra.pt)

**Background**

Human epidermal growth factor receptor 2 (HER2) is overexpressed in 20 to 30 % of breast cancer, as well as in others human cancers [1,2]. The Dual in-situ hybridization (DISH) assay is widely used to study HER2 status, and gives predictive and therapeutic information in invasive breast cancer, although it is dependent on pre-analytical variables, as ischemic time and fixation, among others [1,3].

**Objective**

The aim is to implement a HER2 DISH assay, contributing to its optimization and decrease of variability, clarifying the pre-analytical and analytical variables, with impact in tissue staining and morphology.

**Methods**

Forty-four (44) cases of invasive breast cancer cases previously scored with HER2 2+, were included in this study. Thin 4 μm paraffin sections were submitted to DISH. Unsuccessful cases were submitted to subsequent DISH protocols to attempt a valid result. Slides were evaluated according to staining and morphology integrity, by three independent observers proficient in this methodology, in a blind way, with a light microscope.

**Results**

From the 44 cases, 30 (68.2%) were readily validated, since 14 (31.8%) showed nuclear vacuolization and morphologic disruption leading to further tests with optimized protocols. Unsuccessful cases showed severe morphology damage and were reprocessed with further optimized protocols.

**Conclusions**

According to the obtained results, we can conclude that the pre-analytical variables with major impact on the standardization of the results were time of cold ischemic; unsliced operatory specimens and length of fixation. Analytical variables as time and temperature of cellular permeabilization can be changed to improve inadequate tissue preservation.

**References**

1. Meric-Bernstam F, Hung M-C. Advances in Targeting Human Epidermal Growth Factor Receptor-2 Signaling for Cancer Therapy. Clin Cancer Res. 2006;12(21):6326–30.

2. Brenton JD, Carey LA, Ahmed AA, Caldas C. Molecular Classification and Molecular Forecasting of Breast Cancer: Ready for Clinical Application? J Clin Oncol. 2005;23(29):7350–60.

3. Khoury T, Sait S, Hwang H, Chandrasekhar R, Wilding G, Tan D, et al. Delay to formalin fixation effect on breast biomarkers. Mod Pathol. 2009;22(11):1457–67.

**Keywords**

Dual in situ hybridization, Pre-analytical variables, Breast cancer, Optimization protocols.

### O118 Psychometric properties update of AGITE – a medication self-management and adherence in the elderly questionnaire

#### Maria Almeida^1^, Suzana Duarte^1^, Hugo Neves^2,3^

##### ^1^Coimbra Nursing School, 3046-851 Coimbra, Portugal; ^2^Center for Innovative Care and Health Technology, Polytechnic Institute of Leiria, 2411-901 Leiria, Portugal; ^3^School of Health Sciences, Polytechnic Institute of Leiria, 2411-901 Leiria, Portugal

###### **Correspondence:** Maria Almeida (mlurdes@esenfc.pt)

**Background**

As the human body ages, its function also tends to decline resulting in a higher risk of development of diseases. This leads to the presence of multiple and complex medications in the life of many older adults (OA). As the self-management and adherence behaviours of these medications require the development of competences, it is important to develop a quick and easy instrument to provide systematized data for the health professional that will allow a better decision-making process and a higher probability of developing interventions with impact in the medication-taking ability of the OA. With this purpose, AGITE was developed following a process of systematic literature review, content analysis and psychometric testing, resulting in a total of nineteen questions with a Likert scale approach. Previous studies demonstrated the need for further testing of its psychometric properties.

**Methods**

After application of the AGITE to 146 elders in day centres in Central Portugal, exploratory factorial analysis (EFA) using the eigenvalue criteria, and internal consistency (IC) through Cronbach’s Alpha were performed.

**Results**

From the EFA, using varimax rotation and screen plot analysis, an acceptable KMO of 0.653 was obtained, with no items being eliminated through analysis of anti-image matrix. A total of five dimensions emerged explaining 53.6% of the variance: “Engagement”, “Neglect and External Influences”, “Perceived Benefits”, “Healthcare Professionals Support”, and “Value assigned to Written Information”. Through analysis of the items of each dimension, higher scores of “Engagement” indicate a responsible attitude towards self-management and adherence, while higher scores of “Neglect and External Influences” demonstrate a tendency to cease medication, according to individual and non-professional external beliefs. Regarding the dimension “Perceived Benefits”, higher scores evidence how the elder positively perceives the effects of the medication, while higher scores of “Healthcare Professionals Support” are related with perceived importance of the healthcare professionals in the medication-taking ability. Higher scores of “Value assigned to Written Information” demonstrate a tendency to attribute significance to written data regarding medication. Overall the questionnaire dimensions demonstrate questionable to acceptable IC (0.6 < α < 0.8).

**Conclusions**

New analysis of the psychometric properties evidences the emergence of new dimensions, allowing for a wider understanding of the profile of the medication-taking ability of the elder population in Portugal. These new dimensions will provide a better analysis of this skill to the healthcare professional, allowing a more personalized intervention, with higher chance of success.

**Keywords**

Polypharmacy, Medication management, Elders.

### O119 Emotional intelligence and fear of death in Spanish elders

#### Pedro Garcia-Ramiro^1^, Juan FJ Díaz^2^, Maria González-Melero^1^, Maria DCP Jiménez^1^, Antonio MP Jiménez^1^, Francisco JR Peregrina^1^

##### ^1^Universidad de Jaén, 23071 Jaén, Spain; ^2^Universidad Las Palmas de Gran Canaria, 35001 Las Palmas de Gran Canaria, Spain

###### **Correspondence:** Pedro Garcia-Ramiro (pgramiro@ujaen.es)

**Background**

Researchers, stakeholders and policy makers agree about the importance of the population ageing in modern societies. Emotional Intelligence (EI) has generated a broad interest in the scientific community in Spain [1]. Prestigious social scientists from different lines of research contribute to assess important theoretical and empirical topics on this construct [2]. Aging is a process during which important changes occur in different areas of development and emotional intelligence plays an essential role. Throughout the years, the subject of death has been conceived in different ways. People abstain from talking about it, and a conduct of avoidance can be observed manifesting itself in fear and anxiety [3].

**Objective**

The objective of this study was to examine the relationship between emotional intelligence and fear of death in an older population.

**Methods**

A Spanish sample of 384 older people aged 65 years and older (51.82% women; 71.23 ± 8.34 years of age), without cognitive impairment, were included in this descriptive and correlational study. Data on emotional intelligence and fear of death were obtained through the TMMS-24 and Collett-Lester scales, respectively.

**Results**

Structural equation modelling indicated that emotional intelligence exerted an influence on fear of death. The emotional perception component was positively correlated with the fear of death (r = 0.14; p < 0.05), while understanding and emotional regulation were negatively correlated with fear of death (r = -012; p < 0.001). The higher scores for fear of death were associated with the female gender, and singles. These aspects underscore the importance of the results of this study.

**Conclusions**

These findings show that high levels of emotional intelligence were associated with less fear of death. After controlling sociodemographic variables, the EI dimensions, emotional perception and emotional regulation, accounted for part of the variance in several fears of dead facets. These dimensions can have an important role in the fear of dead of older people.

**References**

1. Wilson CA, Saklofske DH. The relationship between trait emotional intelligence, resiliency, and mental health in older adults: the mediating role of savouring. Aging Ment Health. 2018;22(5):646-654.

2. Lloyd SJ, Malek-Ahmadi M, Barclay K, Fernandez MR, Chartrand MS. Emotional intelligence (EI) as a predictor of depression status in older adults. Arch Gerontol Geriatr. 2012;55(3):570-573.

3. Arca MG. Enfermería en el proceso de humanización de la muerte en los sistemas sanitarios. Enfermería Clínica. 2014;24(5):296-301.

**Keywords**

Emotional intelligence, Fear of death, Ageing, Older adults.

### O120 Vitamin D in food supplements: are we taking too much?

#### Isabel M Costa, Alexandra Figueiredo, Deolinda Auxtero

##### Instituto Universitário Egas Moniz, 2829-511 Caparica, Portugal

###### **Correspondence:** Alexandra Figueiredo (alexandra.f@netcabo.pt)

**Background**

Over the last years, an increase in vitamin D (VitD) supplements intake has been observed. Evidence has suggested multiple effects of VitD beyond bone homeostasis. Low VitD levels are associated with numerous disorders including diabetes, cancer, cardiovascular disease, Parkinson's disease, among others. Consumers have the general misperception that “vitamin” denotes something harmless and vital, disregarding its potentially harmful effects. Although vitD toxicity is uncommon, case reports attributed to vitD supplementation have raised. Being a fat-soluble vitamin, excessive supplementation may result in body accumulation and toxicity. It increases intestinal calcium absorption and plays a central role in its homeostasis. Thus, most symptoms of toxicity result from hypercalcemia. Adverse effects include gastrointestinal disorders (anorexia, diarrhoea, nausea, vomiting), muscle and joint pain, cardiac complaints, hypertension, central nervous system effects and renal disorders (polyuria, polydipsia).

**Objective**

The aim of this study was to evaluate whether VitD3 (cholecalciferol) daily dose indicated on food supplements (FS) labels coincided with the recommended daily allowance (RDA) for this vitamin defined by the European Union Directive.

**Methods**

Labels of 110 FS sold in Portuguese pharmacies, supermarkets or health shops were examined. Selection criteria included: oral solid pharmaceutical forms for adults, containing vitD in its composition, as stated in the label, regardless of the purpose of the FS.

**Results**

66.4% of FS presented vitD label doses above RDA and four of them indicated a daily dose ≥ the tolerable upper intake level defined by EFSA (UL=100 μg/day). In the majority of the FS evaluated, vitD label dose far exceeded RDA value and some exceeded UL defined by EFSA.

**Conclusions**

At present, the safety of FS and the authenticity of label information is exclusively ensured by the economic operators who place FS on the market. Since FS are usually taken without any medical supervision or counselling and attending the potential adverse effects of vitD excess, it is imperative that the daily doses of vitD present in FS are reviewed attending to RDA values. Authors also suggest that FS should be under the same quality control of pharmaceuticals, regarding FS consumers health.

**Keywords**

Vitamin D, Food Supplements, Recommended Daily Allowances, Tolerable Upper Intake Level.

### O121 Functional ability and risk of falling - a base for exercise prescription

#### Sílvia Vaz, Anabela Martins, Carla Guapo, Sara Martins

##### Physiotherapy Department, Coimbra Health School, Polytechnic Institute of Coimbra, 3046-854 Coimbra, Portugal

###### **Correspondence:** Sílvia Vaz (leontina_vaz@hotmail.com)

**Background**

Falls are currently considered one of the most common and serious public health problems [1, 2]. Faced with this problem, it becomes necessary to explore which factors can better predict the risk of falls in individuals living in the community [3], so that, preventive measures can be considered.

**Objective**

To identify fall risk indicators and to relate them to exercise prescription levels; to relate the history of falls, the functional capacity (measured through the Timed Up & Go, 10-meter walking speed test, Step test) and the fall risk factors and propose a guide based on those relations to address exercise prescription.

**Methods**

Descriptive and exploratory study. Two hundred community dwelling adults aged 55 or older were assessed, integrating two sub-samples, a Portuguese and a Polish. Study participants were assessed for socio-demographic data, history of falls, fear of falling, exercise, sedentary lifestyle, hearing problems and/or dizziness, visual problems, alcohol consumption, exercise self-efficacy and confidence in activities of the daily life (FES-Portuguese version). Functional capacity was assessed by three golden measures for the risk of fall: the Timed Up and Go (TUG), the 10-meter walking speed test and the Step Test (15s). The statistical design included descriptive analyses, inferential analyses (bivariate: t-test for independent samples, One-Way ANOVA and Pearson’s correlation coefficient).

**Results**

Fall incidence was 39.5% and 45.3% in the total and Portuguese samples, respectively. TUG, 10-meter walking speed test and step test can distinguish those with history of falls from those without, with statistically significant differences (p ≤ 0.05). Taking more than 4 different medications per day, fear of falling, hearing problems and/or dizziness and the need for help getting up from a chair were correlated to the history of falls, the TUG, the walking speed and the step test (p ≤ 0.05). The sedentary lifestyle and the use of assistive devices were associated with worst performance in the functional tests (p < 0.05) in the Portuguese sample. TUG, 10-meter walking speed test and step test were correlated with exercise self-efficacy.

**Conclusions**

The incidence of falls is higher than literature has reported and it is inversely associated with the functional capacity of community-dwelling adults aged over 55 years old. Data from this study is a valuable basis for exercise prescription, taking into account the levels of risk and the components of exercise prescription.

**References**

1. Gschwind Y, Kressig R, Lacroix A, Muehlbauer T, Pfenninger B, Granacher U. A best practice fall prevention exercise program to improve balance, strength / power, and psychosocial health in older adults: study protocol for a randomized controlled trial. BMC Geriatrics. 2013;13(1):105.

2.NICE. Falls in older people overview. NICE Pathways. 2016;1-13.

3. Avin K, Hanke T, Kirk-Sanchez N, McDonough C, Shubert T, Hardage J, Hartley G. Management of falls in community-dwelling older adults: clinical guidance statement from the Academy of Geriatric Physical Therapy of the American Physical Therapy Association.Physical Therapy. 2015;95(6):815-834.

**Keywords**

Risk of fall, Functional capacity, Prevention of falls, Exercise prescription, Self-efficacy.

### O122 The impact of the FIFA 11+ on physical performance of amateur futsal players: short and long term effects

#### Mário Lopes^1^, Daniela Simões^2^, João M Rodrigues^3,4^, Rui Costa^1,5^, José Oliveira^6^, Fernando Ribeiro^1,7^

##### ^1^School of Health Sciences, University of Aveiro, 3810-193 Aveiro, Portugal; ^2^Santa Maria Health School, 4049-024 Porto, Portugal; ^3^Institute of Electronics and Informatics Engineering of Aveiro, 3829-193 Aveiro, Portugal; ^4^Department of Electronics, Telecommunications and Informatics, University of Aveiro, 3810-193 Aveiro, Portugal; ^5^Center for Health Technology and Services Research, University of Aveiro, 3810-193 Aveiro, Portugal; ^6^Research Centre in Physical Activity, Health and Leisure, Faculty of Sport, University of Porto, 4200-450 Porto, Portugal; ^7^Institute of Biomedicine, University of Aveiro, 3810-193 Aveiro, Portugal

###### **Correspondence:** Mário Lopes (mariolopes77@ua.pt)

**Background**

The effects of the FIFA 11+ on physical performance parameters has demonstrated controversial results.

**Objective**

The aim of this study was to observe the short and long-term effects of the FIFA 11+ on performance in male amateur futsal players.

**Methods**

Seventy-one (71) male futsal players from six amateur clubs were randomized to an intervention (N = 37, age: 27.0 ± 5.1 years) or a control group (N = 34, age: 26.0 ± 5.1 years). The intervention group was submitted to 10 weeks of FIFA 11+ injury prevention program, 2 sessions/week, followed by a 10-week follow-up period, while the control group performed regular futsal warm-ups during the training sessions. During the follow-up period both groups performed only regular warm-ups during their training sessions. Physical performance was assessed by measuring agility (T-test), sprint (30-meter sprint), flexibility (sit and reach) and vertical jump performance (squat jump).

**Results**

Differences between groups were found at baseline for training exposure, body mass index, body weight, flexibility and sprint. The results of the effect of the FIFA 11+ on the sit and reach, speed, jump performance and agility did not show differences pre-post intervention after adjustment for the baseline differences, as well as for the 10-week follow-up.

**Conclusions**

The current study has shown no short and long-term performance enhancement in sprint, flexibility, agility and jump performance after the FIFA 11+ in male amateur futsal players.

**Keywords**

Prevention program, Warm-up, Injury, Neuromuscular training, Amateur male players.

### O123 Effects of aquatic fitness in older women conditioning: an 8-week program

#### Pedro Morouço^1^, Sandra Amado^1,2,3^, Susana Franco^4^, Fátima Ramalho^4^

##### ^1^Centre for Rapid and Sustainable Product Development, Polytechnic Institute of Leiria, 2430-028 Marinha Grande, Portugal; ^2^School of Health Sciences, Polytechnic Institute of Leiria, 2411-901 Leiria, Portugal; ^3^Center for Innovative Care and Health Technology, Polytechnic Institute of Leiria, 2411-901 Leiria, Portugal; ^4^Sport Sciences School of Rio Maior, Polytechnic Institute of Santarém, 2040-413 Rio Maior, Portugal

###### **Correspondence:** Pedro Morouço (pedro.morouco@ipleiria.pt)

**Background**

There are several evidences in the literature demonstrating a high positive association between increased levels of exercise and improved health, specifically in older adults [1]. As such, in recent years there has been a large number of studies examining the benefits imposed by different types of exercise (e.g. resistance training [2], and aquatic fitness [3]). However, in addition to the benefits imposed, it is crucial that the exercise is motivating and challenging.

**Objective**

It was aimed to examine the possible effects on conditioning induced by 8-weeks of aquatic fitness, in female older adults.

**Methods**

Fourteen women (64.3 ± 7.3 years old) enrolled in bi-weekly aquatic fitness of 45’ sessions for 8 weeks. Before and after the 8 weeks, participants performed the Senior Fitness Test [2], hand-grip strength and body measures. All participants were volunteer, informed consent was obtained and all procedures were in accordance to Helsinki Declaration. Sessions were instructed by a CSCS®.

**Results**

Significant and meaningful improvements were observed in lower body strength (p < 0.001; d = 1.22), lower body flexibility (p < 0.001; d = 3.54), aerobic endurance (p < 0.001; d = 1.35), dynamic balance (p < 0.001; d = 1.53) and hand grip strength (p < 0.001; d = 2.02). Significant, but moderate improvements were observed in body mass (p = 0.021; d = 0.72) and hip circumference (p = 0.048; d = 0.59).

**Conclusions**

Eight weeks of aquatic fitness induced extensive benefits in older women conditioning, suggesting that this activity is able to promote an increase in life quality. The present results corroborate previous studies, demonstrating that aquatic exercise is a reliable approach for improved health in the elderly.

**Acknowledgements**

This research was supported by the European Regional Development Fund (FEDER), through COMPETE2020 under the PT2020 program (POCI-01-0145-FEDER-023423), and by the Portuguese Foundation for Science and Technology (UID/Multi/04044/2013).

**References**

1. Taylor D. Physical activity is medicine for older adults. Postgrad Med J. 2014;90:26-32.

2. Martins WR, Safons MP, Bottaro M, Blasczyk JC, Diniz LR, Fonseca RMC, et al. Effects of short term elastic resistance training on muscle mass and strength in untrained older adults: a randomized clinical trial. BMC Geriatr. 2015;15(1):99.

3. Bartolomeu RF, Barbosa TM, Morais JE, Lopes VP, Bragada JA, Costa MJ. The aging influence on cardiorespiratory, metabolic, and energy expenditure adaptations in head-out aquatic exercises: Differences between young and elderly women. Women Health. 2017;57(3):377–391.

4. Rikli RE, Jones CJ. Senior fitness test manual. 2^nd^ edition. Human Kinetics; 2013.

**Keywords**

Exercise, Health, Aging, Physical Fitness.

### O124 Predicting social participation in the community-dwelling older adults

#### Carla Guapo, Anabela C Martins, Sara Martins, Sílvia Vaz

##### Physiotherapy Department, Coimbra Health School, Polytechnic Institute of Coimbra, 3046-854 Coimbra, Portugal

###### **Correspondence:** Sílvia Vaz (leontina_vaz@hotmail.com)

**Background**

Nowadays, active ageing is both a complex scientific term and a goal for most people, but also an undeniable political objective [1]. Participating socially helps to develop the feeling of belonging to a community and allows everyone to see each individual contribution in upholding the community [2-4].

**Objective**

Characterize the profile of community-dwelling adults aged 55 or older, regarding social participation, functional capacity (walking speed, grip strength, lower limb strength, static and dynamic balance) and personal factors (age, gender, BMI, confidence/fear of falling and perception of general health); verify the relationship between social participation and functional capacity as well as participation and personal factors; and find, among all the variables, which can be the best predictors of social participation.

**Methods**

Descriptive, exploratory and cross-sectional study. The sample is composed of 150 Portuguese community-dwelling older adults. The statistical design included descriptive analyses (measures of central tendency and dispersion); inferential analyses (bivariate: t-test for independent samples, One-Way ANOVA and Pearson’s correlation coefficient; multivariate: linear multiple regression, moderated linear multiple regression and hierarchical linear multiple regression). The level of significance was α =0.05, with a 95% confidence interval.

**Results**

The results have shown that this sample was composed mostly by women, mean age approximately 69 years old. Statistically significant associations between social participation and all study variables: Age (r = 0.301, p = 0.00), BMI (r = 0.169, p = 0.039), Grip strength (r = -0.318, p = 0.00), Pressure platform Hercules® (r = -0.337, p = 0.00), perception of general health (r = 0.468, p = 0.00), Timed Up & Go (r = 0.668, p = 0.00), T10M (r = -0.576, p = 0.00), Test Step (r = -0.456, p=0.00) and Fall Efficacy Scale (r = 0.768, p = 0.00). Regression analysis shows that the confidence in performing activities of the daily living without fear of falling, health perception and dynamic balance, measured by Timed Up & Go test, as a whole, are responsible for 65.5% of the variance of social participation (R^2^ = 0.655; p < 0.001). Using a second model we have seen that a sizeable part of the variance percentage related to the participants’ social participation, 55%, is due once again to dynamic balance and health perception, followed by age (R^2^ = 0.549; p < 0.001).

**Conclusions**

The Timed Up & Go test and the unique question on health perception: “*In general, would you say that your health is excellent, very good, good, satisfactory or poor?*” account for a significant percentage of the variance in social participation in elderly individuals. Incorporating these two factors into the physical therapist's clinical practice takes very little time and greatly benefits the decision-making process and planning of interventions.

**References**

1. Fernandez-Ballesteros R, Zamarron MD, Diez-Nicolas J, Lopez-Bravo MD, Molina MA, Schettini R, Productivity in Old Age. Research on Aging. 2011;33(2):205–226.

2. Holt-Lunstad J, Smith TB, Layton JB. Social relationships and mortality risk: A meta- analytic review. PLoS Medicine. 2010;7(7):e1000316.

3. Korpershoek C, van der Bijl J, Hafsteinsdottir TB. Self-efficacy and its influence on recovery of patients with stroke: A systematic review. Journal of Advanced Nursing. 2011;67(9):1876–1894.

4. Nayak N, Mahajan P. Walking Capacity and Falls-Efficacy Correlates with Participation Restriction in Individuals with Chronic Stroke: A Cross Sectional Study. International Journal of Physiotherapy. 2015;2(1):311.

**Keywords**

Active ageing, Elderly, Social participation, Functional capacity, Functioning.

### O125 Motor development in children from 11 to 44 months old: influence of the variable “presence of siblings”

#### Miguel Rebelo^1^, João Serrano^1^, Daniel Marinho^3,4^, Rui Paulo^1,2^, Vivian Corte^1^, Pedro Duarte-Mendes^1,2^

##### ^1^Department of Sports and Well-being, Polytechnic Institute of Castelo Branco, 6000-266 Castelo Branco, Portugal; ^2^Research on Education and Community Intervention, 4411-801 Vila Nova de Gaia, Portugal; ^3^Department of Sport Sciences, University of Beira Interior, 6201-001 Covilhã, Portugal; ^4^Research Centre in Sports, Health and Human Development, University of Beira Interior, 6201-001 Covilhã, Portugal

###### **Correspondence:** Miguel Rebelo (miguelrebelo7@hotmail.com)

**Background**

Motor development presupposes a set of life-long processes of change. These processes occur mostly during the first years of the child’s life, having each child different developmental rhythms [1]. Motor skills are fundamental to our day-to-day life, representing the key to the child's development [2]. As such, it is important to know the different factors that influence the development of motor skills during childhood. According to bibliography, the presence of siblings may be an important factor, because this relationship provides a basis for learning and socialization opportunities in various contexts [3].

**Objective**

The main goal of this study was to verify if there were differences in the development of motor skills (global and fine) through the scales of the PDMS-2, comparing children that have or do not have siblings.

**Methods**

In this study 91 children of both sexes participated (30.20 ± 10.56 months). Two groups were created: the sibling group, consisting of 48 children (31.06 ± 10.76 months) and the non-sibling group, consisting of 43 children (29.23 ± 10.37 months). Motor skills were assessed using the PDMS-2 test battery scales4. The evaluation was performed during 4 months, 3 times a week and individually (approximately 30 minutes for each child). For the data analysis, we used descriptive and inferential statistics. The Kolmogorov-Smirnov test was applied to test normality, and the Mann-Whitney test was applied to independent samples.

**Results**

The sibling group achieved, on average, better results in all motor skills (global and fine). However, there were only statistically significant differences in fine motor skills (p = 0.016), where the sibling group had the best results (average = 52.29) compared to the non-sibling group (average = 38.98).

**Conclusions**

These results show that the presence of siblings in the family context positively influences motor development, providing cooperative activities through play and challenges that improve cognitive, social, emotional and physical development.

**Acknowledgements**

This work was supported by the Portuguese Foundation for Science and Technology (FCT; Grant Pest – OE/CED/UI4016/2016).

**References**

1. Barreiros J, Neto C. O Desenvolvimento Motor e o Género. Lisboa: Faculdade de Motricidade Humana ; 2005.

2. Leonard HC, Hill EL. The impact of motor development on typical and atypical social cognition and language: a systematic review. Child and Adolescent Mental Health. 2014;19(3):163-170.

3. Brody GH. Siblings’ direct and indirect contributions to child development. Current Directions, Psychological Science. 2004;13(3):124–126.

4. Folio R, Fewell R. Peabody Developmental Motor Scales-2. Austin: TX: Pro-Ed; 2000.

**Keywords**

Motor Development, Family Context, PDMS-2.

### O126 Childhood obesity in the urban parishes of Coimbra municipality

#### Margarida Pereira, Cristina Padez, Helena Nogueira

##### Research Centre for Anthropology and Health, University of Coimbra, 3000-456 Coimbra, Portugal

###### **Correspondence:** Margarida Pereira (mmiguel06@gmail.com)

**Background**

Childhood obesity is a major public health concern worldwide and Portugal has one of the highest rates of childhood obesity among the European countries. It is known that childhood obesity is particularly high in urban settings. Thus, a deeper understanding of the impact of such areas in children weight is needed. Evidence suggests that parents’ perception of the neighbourhood safety might determine children weight once unsafety perceptions of the neighbourhood prevent children from playing outside.

**Objective**

This work main goal was to examine the impact of parents’ safety perception of the neighbourhood in children’s weight status regarding the localization of the neighbourhood (urban centre or urban periphery).

**Methods**

Weight (kg) and height (cm) of 1,493 children from Coimbra municipality were measured and BMI (weight/height^2^) was calculated to use IOTF cut-off points to classify children in “normal” or “obese”. Parents provided their parish of residence as well as their weight, height and number of schooling years. They also responded to a questionnaire regarding their neighbourhood perceptions and physical activity engagement of their children. The sample was analysed separately, *i.e.*, chi-square tests were computed to children living in parishes from the urban centre and posteriorly to children living in parishes from the urban periphery.

**Results**

This study results showed proportionality between overweight or obese children residing in the urban centre, mainly girls, with low socioeconomic status and obese parents that strongly agree that their neighbourhood is unsafe to walk in during the day. Except for mother weight status, none of the variables analysed differentiated normal from overweight or obese children living in the urban periphery, regarding the chi-square tests.

**Conclusions**

Overall, parents’ perceptions of the environment might impact children’s weight status. However, even within the same urban area, perceptions of neighbourhood safety change. The aspects that influence children weight status differ according to the parishes they live in - urban centre or peripheric parishes. For example, parents from a significant proportion of overweight or obese children living in the urban centre parishes perceives their neighbourhood environment as unsafe to walk during the day, however, no differences were found between normal and overweight or obese children from the peripheric parishes. This should be held in consideration when developing healthy urban planning strategies.

**Acknowledgements**

Work funded by the Foundation for the Science and Technology (PTDC/DTP-SAP/1520/2014 and grant SFRH/BD/133140/2017).

**Keywords**

Childhood Obesity, Urban Settings, Neighbourhood, Safety Perceptions.

### O127 Cardiovascular causes of disqualification from competitive sports: young vs. veteran athletes

#### Ana P Silva, Virgínia Fonseca, Carolina Diniz, Daniel Pereira, Rodrigo Sousa, João Lobato

##### Escola Superior de Tecnologia da Saúde de Lisboa, Instituto Politécnico de Lisboa, 1990-094 Lisboa, Portugal

###### **Correspondence:** Ana P Silva (ana.silva@estesl.ipl.pt)

**Background**

Cardiovascular disease is the most common cause of disqualification from competitive sports. The pre-participation screening is fundamental in order to detect these diseases and is based on clinical history and physical examination in addition to a 12-lead electrocardiogram. Additional tests are requested only for those with any abnormality in the initial evaluation [1-2]. According to previous studies, the most common cardiovascular diseases that disqualify young athletes are different from those associated to veteran athletes: congenital arrhythmias vs. subclinical coronary disease, respectively [3-5].

**Objective**

To analyse and compare, amongst young and veteran athletes, the cardiovascular causes of disqualification from competitive sports, consecutively screened at a sports medicine unit in a decade (2007-2017).

**Methods**

Descriptive-comparative retrospective study. The study population consisted of all case files from athletes disqualified from competitive sports due to cardiovascular disease during the 2007-2017 period. A sample of 58 case files was divided into group A (young athletes, < 35 years, n_A_= 36) and group B (veteran athletes, ≥ 35 years, n_B_=22). It was evaluated the clinical history, sport disciplines, symptoms and cardiovascular diseases. Descriptive statistics and statistical inference (Chi-squared distribution) were applied for the characterization and comparison of the study variables.

**Results**

Both sample groups consisted mainly in male athletes (group A 94.4%, group B 100%). The most referred symptom in group A was palpitations (16.7%), whereas in group B was chest pain (36.4%). There was a significant association between relevant cardiovascular history and veteran athletes. The most frequent cardiovascular diseases in group A were hypertrophic cardiomyopathy (19.4%), arterial hypertension (11.1%), left ventricle noncompaction (8.3%) and great vessel transposition (8.3%). Arterial hypertension (50%) and coronary disease (45.4% were the most frequent diseases that disqualified the practice of competition sports in veteran athletes. It’s important to emphasize that some veteran athletes presented simultaneously more than one cardiovascular cause of disqualification.

**Conclusions**

The most frequent cardiovascular diseases in groups A and B matched those found in literature [3-5]. The prevalence of hypertrophic cardiomyopathy and coronary disease in the respective groups may be associated with a higher awareness towards the dangers of these particular diseases in the practice of competition sports. The data in this study confirms the key role of pre-participation screening for the identification of cardiovascular diseases that can cause sudden cardiac death during sport.

**References**

1. Corrado D, Pelliccia A, Bjørnstad H, Vanhees L, Biffi A, Borjesson M, et al. Cardiovascular pre-participation screening of young competitive athletes for prevention of sudden death: proposal for a common European protocol. European Heart Journal. 2005;26:516-524.

2. Despacho no 25 357/2006. D.R. no 238 de 13.12.2006 - 2a Série, (2006).

3. Abbatemarco J, Bennett C, Bell A, Dunne L, Matsumura M. Application of Pre-participation Cardiovascular Screening Guidelines to Novice Older Runners and Endurance Athletes. SAGE Open Medicine. 2016;4:1–8.

4. Pescatore V, Basso C, Brugin E, Bigon L, Compagno S, Reimers B et al. Cardiovascular causes of disqualification from competitive sports in young athletes and long term follow-up. European Heart Journal. 2013;34(sup 1):1783.

5. Corrado D, Basso C, Schiavon M., Thiene G. Screening for Hypertrophic Cardiomyopathy in Young Athletes. The New England Journal Of Medicine. 1998;339:364-69.

**Keywords**

Cardiovascular diseases, Competitive sports, Pre-participation screening.

### O128 Bioethics, health promotion and sustainability: interfaces in higher education

#### Ivani N Carlotto, Maria AP Dinis

##### Energy, Environment and Health Research Unit, Energy, Environment and Environmental & Public Health Research Laboratories, Fernando Pessoa University, 4249-004 Porto, Portugal

###### **Correspondence:** Ivani N Carlotto (ivani.carlotto@gmail.com)

**Background**

Universities are essential institutions for health promotion (HP) [1]. As they have their own ethos and distinct cultures, they may act as potential enhancers of the conceptual frameworks of HP and interdisciplinary values such as equity, social justice and sustainable growth [2]. Bioethics, as a transversal discipline, seeks to ethically analyse and systematize such values, strengthening the synergy between health and sustainability [3]. Bioethics is a reflexive, mutually shared and interdisciplinary tool whose goal is to promote health and sustainability in an integrated and coherent way, adapting life actions in their equitable and inclusive characters.

**Objective**

1) Identify how bioethics takes place in daily life and how it is possible to establish links between scientific and ethical knowledge, in order to avoid negative impacts on people's lives; 2) Describe the appropriate bioethical tools (principles) for intervention in the context of higher education (HE), HP and sustainability.

**Methods**

Exploratory-descriptive methodology using a quanti-qualitative approach [4]. Sample: University teachers from Rio Grande do Sul/Brazil, random sample, probabilistic sampling by convenience, CI = 95%, n = 1400 persons. The research was approved by the Research Ethics Committee of the Hospital de Clínicas of Porto Alegre (HCPA)/Brazil, Ethics Committee of the Universidade Fernando Pessoa (UFP)/Porto-Portugal, receiving the approval number CAAE 55066616.8.0000.5327/Plataforma Brasil/Brazil. The interviews were carried out after receiving the informed consent from the participants, taking into account the assumptions of the National Health Council Brazil (NHC) 466-2012.

**Results**

Beyond the principalistic formulation - charity, non-maleficence, justice and respect for autonomy [5], certain subjacent referentials, such as, solidarity, shared commitment, and health environment/sustainability were evoked, causing a positive impact on HP, individual and collective well-being, quality of life, inclusion and social justice in the University environment.

**Conclusions**

HE upholds a fundamental role in HP for their faculty teachers. Universities act as places for investigation and learning in a way that it invigorates HP activities [6]. Bioethics, as a transdisciplinary activity, seeks to help building qualified actions in health, which uphold and promote well-being, cohesion, inclusion, sustainability and social justice, with the respective conceptual clarity that resides therein [2, 7].

**References**

1. Dooris M, Doherty S, Cawood J, Powell S. The Healthy Universities approach: Adding value to the higher education sector. In: Health promotion settings: Principles and practice. London: Sage; 2012. p. 153-169.

2. Dooris M, Doherty S, Orme J. The application of salutogenesis in universities. In: The Handbook of Salutogenesis. England: Springer; 2017.

3. Garrafa V. Da bioética de princípios a uma bioética interventiva. Bioética. 2005;13:125-134.

4. Prodanov CC. Metodologia do trabalho científico: métodos e técnicas da pesquisa e do trabalho acadêmico. Novo Hamburgo: Feevale; 2013.

5. Beauchamp TL, Childress JF. The principles of biomedical ethics. New York: Oxford;1979.

6. Organização PanAmericana de Saúde [http://www.paho.org/]. Regional program on bioethics. [Accessed in 02 may 2017]. Available in http://www.paho.org/ hq/index.php?option=com_content&view=article& id=5582%3A2011-regional-program-onbioethics&ca tid=3347%3Abioethics&Itemid=4124&lang=es

7. Carlotto IN, Dinis MAP. Bioética e promoção da saúde docente na educação superior: uma interface necessária. Revista Saber & Educar. 2017;23:168-179.

**Keywords**

Bioethics, Health Promotion, Higher Education, Sustainability.

### O129 Pilot program to develop clinical skills in counseling-based motivational interview (CBMI) to prevent obesity in Chile

#### Ricardo Cerda^1^, Daniela Nicoletti^1^, Macarena P Lillo^3^, Margarita Andrade, Patricia Galvez^1^, Lorena Iglesias^1^, Denisse Parra^2^, Magdalena C Coke, Natalia Gomez^1^

##### ^1^Department of Nutrition, Faculty of Medicine, University of Chile, Santiago, Chile; ^2^Department of Nursing, Faculty of Medicine, University of Chile, Santiago, Chile; ^3^School of Journalism, Faculty of Communication and Letters, University Diego Portales, Santiago, Chile

###### **Correspondence:** Ricardo Cerda (rcerdarioseco@gmail.com)

**Background**

To influence mediator variables of behavioural change in health and the adherence to treatment in an individual context, health professionals and users must develop a help-based relation mediated by effective communication. At the same time, health professionals must trigger processes in the users that allow them to recognize and develop intrinsic motivation towards change. In this sense, a pilot program is proposed for training CBMI for primary health care (PHC) nutritionists that develops knowledge and tools to foster behavioural change in users. The pilot project was developed as part of the Chilean health program “Vida Sana”.

**Objective**

Describe a training pilot program to develop clinical skills in CBMI, for PHC nutritionists, to prevent obesity in Chile.

**Methods**

A training program was built comprised by 34 face-to-face hours and 8 hours of accompaniment at the workplace for 13 Nutritionists. The program was based on a constructivist approach centred on the development of skills in the following sequence: critical analysis for regular practice, adherence comprehension and behavioural change, communication skills, motivational interviewing skills, skill integration in a simulated and real situation. The program employed psychometric scales for motivation, beliefs and self-efficacy for CBMI, video analysis, observations performed at the Centre for Clinical Skills at the Facultad de Medicina de la Universidad de Chile and accompaniment at PHC centres.

**Results**

Participant knowledge increased on average 5.25 to 20.85 (p = 0.008). The average of total points did not vary at the beginning or at the end (74 pts). Effective beliefs increased from 61.3 to 68.7 (p < 0.05) and self-efficacy from 1617 to 1851 (p < 0.05). Observation and video analysis showed that Nutritionists went from delivering information to open and strategic inquiring during the course. Accompaniment showed that skills were deepened and the level of satisfaction improved with practice.

**Conclusions**

This is an innovative program that incorporates CBMI and defines a methodology centred on reflection, practice and accompaniment in real and simulated situations. It is necessary to evaluate the effects in indicators that measure user behaviour and the effect and impact on adult obesity. This training program represents a tool to promote behavioural change and adherence in PHC to prevent obesity.

**Acknowledgements**

The project was financed by CONICYT: FONIS SA16I0122.

**Keywords**

Behavior Change, Motivational Interview, Professional Education, Obesity, Nutritionists Skills.

### O130 Childhood body fat and motor competence in elementary school (5 to 9 years old)

#### Francisco Campos^1^, Ricardo Santos^1^, Mariana Temudo^1^, Kátia Semedo^1^, Diogo Costa^1^, Ricardo Melo^1^, Fernando Martins^1,2^

##### ^1^Coimbra Education School, Polytechnic Institute of Coimbra, 3030-329 Coimbra, Portugal; ^2^Instituto de Telecomunicações, University of Beira Interior, 6201-001 Covilhã, Portugal

###### **Correspondence:** Francisco Campos (francicampos@gmail.com)

**Background**

Obesity rates have increased globally in the last decades, justifying the denomination “public health epidemic”. According some studies [1], in Portugal, childhood overweight and obesity affects about 31.5% of elementary school children, with higher values for girls, except between 7.5 and 9.0 years old. Overweight and obesity are strictly related with childhood motor competence [2]. To access overweight and obesity, among others, is recommended the body fat percentage (BFP), classified by age and gender by McCarthy centiles of BFP [3].

**Objective**

The main objectives of this investigation are: 1) to characterize childhood elementary school overweight/obesity and compare it by gender and age; 2) to correlate childhood elementary school overweight/obesity with motor competence.

**Methods**

Data was collected from 604 children’s between 5 to 9 years old (7.40 ± 1.16 years old; 295 female) of the 10 elementary schools from the “Agrupamento de Escolas de Montemor-o-Velho” (Coimbra-Portugal), using: a) the electrical bio-impedance (model BC-533®) method, to access BFP; and b) a battery of physical tests [shifting platforms and lateral jumps (stability); shuttle run and standing long jump (locomotion); throwing velocity and kicking velocity (manipulation); and handgrip strength] to access motor competence [3]. Data analysis was conducted using IBM SPSS software (version 24.0) for a statistical significance of 10%.

**Results**

In this study case, only 58.8% (n = 355) of the elementary school children had normal weight, and 41.2% showed overweight/obesity [overweight: 17.0% (n = 103); obesity: 24.2% (n = 146)]. There were no significant statistical differences (p = 0.519) between genders (Mann-Whitney). By age (Kruskal-Wallis), there were significant statistical differences (p = 0.001), specially between the 5 years old (Md=2) [p=0.016 (7 years old; Md=1); p=0.003 (8 years old; Md=1); p=0.021 (9 years old; Md=1)] and the 6 years old (Md=2) [p=0.005 (7 years old; Md=1); p=0.001 (8 years old; Md=1); p=0.013 (9 years old; Md=1)]. For Md interpretation, normal weight is classified by 1, overweight by 2 and obesity by 3. The Spearman test (r) allowed to verify significant statistical correlations, two positive [shuttle run (p=0.001; r=0.136); handgrip (p=0.002; r=0.123)] and three negative [lateral jump (p=0.001; r=-0.174); standing long jump (p=0.001; r=-0.249); throwing velocity (p=0.072; r=-0.073)].

**Conclusions**

It is important to take into account the current recommendations and concerns of the WHO [4] (healthy eating habits, physical activity regular practice), improving body composition and motor competence in childhood from an early age, resulting probably in healthier adults and minimizing possible social problems concerning public health.

**Acknowledgements**

This work is funded by FCT/MEC through national funds and when applicable cofounded by FEDER - PT2020 partnership agreement under the project UID/EEA/50008/2013 and by QREN, Mais Centro - Programa Operacional Regional do Centro, FEDER (CENTRO-07-CT62-FEDER-005012; ID: 64765).

**References**

1. Venâncio P, Aguilar S, Pinto G. Obesidade infantil… um problema cada vez mais atual. Revista Portuguesa de Medicina Geral e Familiar 2012;28:410-416.

2. Luz C, Cordovil R, Almeida G, Rodrigues L. Link between motor competence and Health related fitness in children and adolescents. Sports 2017;5(41):1-8.

3. McCarthy H, Cole T, Fry T, Jebb S, Prentice A. Body fat reference curves for children. International Journal of Obesity 2006;30:598-602.

4. Inchley J, Currie D, Jewell J, Breda J, Barnekow V. Adolescent obesity and related behaviours: trends and inequalities in the WHO. Copenhagen: WHO; 2017.

**Keywords**

Body Fat Percentage, Elementary School, Motor Competence, Obesity, Overweight.

### O131 Working pregnant woman affectivity assessment regarding psychological requirements of the work

#### Maria S Medina, Valeriana G Blanco

##### Universidad de Burgos, 09001 Burgos, Spain

###### **Correspondence:** Valeriana G Blanco (vguijo@ubu.es)

**Background**

The labour situation of the pregnant woman has special connotations, both physical and psychological and this may influence work performance and perceived welfare, therefore also interfering in the development of different emotions.

**Objective**

Aware of this, the present work has as an objective to evaluate the relationship between the psychological requirements of the work of pregnant women and affectivity.

**Methods**

It had a convenience sample of 165 pregnant working women living in Burgos (Spain). The study has a cross-cutting nature and the data collection was carried out with the PANAS questionnaire for affectivity rating, ISTAS for psychological demand and an *ad hoc* questionnaire to collect identification data. The criterion variables were: work psychological requirements (EP), positive affectivity (AP) and negative affectivity (AN).

**Results**

The results show that pregnant women have high unfavourable exposition levels for health regarding variables of psychological requirements.

**Conclusions**

The relation between variables showed significant relation between the EP and AN variables, concluding that pregnant women with a friendly exposition level of psychological requirements (EP) have a lot of positive affectivity (AP) and less negative. Pregnant women that had an unfavourable psychological exposition level (EP) had more negative affectivity (AN).

**Keywords**

Working pregnant woman, Affectivity assessment, Psychological requirements of the work.

### O132 The perception of social support and adherence to medication, on the person with COPD

#### Sílvia Vieira^1^, Celeste Bastos^1,2^, Lígia Lima^1,2^

##### ^1^Nursing School of Porto, 4200-072 Porto, Portugal; ^2^Center for Health Technology and Services Research, University of Porto, 4200-450 Porto, Portugal

###### **Correspondence:** Sílvia Vieira (silvia_vieira3@hotmail.com)

**Background**

COPD (Chronic Obstructive Pulmonary Disease) is a chronic and incapacitating disease, characterized by the presence of persistent respiratory symptoms and a gradual decrease in energy [1-3]. The person with COPD has to cope with a complex therapeutic regimen and with the progressive worsening of the clinical condition [3], which may compromise their capacity for self-care. Therefore, people with COPD need support to manage the disease and the therapeutic regimen [4-6].

**Objective**

The study aims were to study people with COPD perception about their social support, as well as their level of adherence to medications and to analyse the association between perceived social support and adherence to medication.

**Methods**

This is a quantitative, descriptive and cross-sectional study, with a sample of 45 adults diagnosed with COPD, admitted to medical service at a hospital in the northern part of Portugal, between February and May 2017. Participants mean age was 71 years (SD = 11.9), they were mostly male (86.7%), married (72.7%) and had a low level of education. The measures used were: a sociodemographic and clinical questionnaire, the Social Support Scale (SSS) and Reported Adherence to Medication Scale (RAMS).

**Results**

The results showed that the study participants perceived a positive social support (M = 3.5, SD = 0.8). The higher scores were found for the dimension of family and affective support (M = 3.9, SD = 1.0), and the lowest scores were found in the financial support dimension (M = 2.7, SD = 1.0). In relation to the treatment of COPD, most participants reported high adherence levels (M = 12.5, SD = 4.4). A positive association was found between perceived social support and medication adherence (r = 0.46, p = 0.001).

**Conclusions**

Our results support the importance of social support in adherence to medication, on the person with COPD. The study also suggests the existence of a group of patients more at risk, in terms of lack of social support and non-adherence to medication, pointing out the need to develop nursing interventions focused on the promotion of self-management of COPD.

**References**

1. Global Initiative for Chronic Obstructive Lung Diseases. Pocket Guide to COPD Diagnosis, Management, and Prevention. A Guide for Health Care Profissionals (2017 Report). Global Inititative for Chronic Lung Disease, Inc., 2017.

2. Criner G, Bourbeau J, Diekemper R, Ouellette D, Goodridge D, Stickland M, et al. Prevention of acute exacerbations of COPD: American College of Chest Physicians and Canadian Thoracic Society Guideline. Chest 2015;147(4):894-942.

3. Wedzicha J, Miravitlles M, Hurst J, Calverley P, Albert R, Krishnan J, et al. Management of COPD exacerbations: a European Respiratory Society/American Thoracic Society guideline. Eur Respir J. 2017 Mar 15;49(3). pii: 1600791.

4. Korpershoek Y, Bos-Touwen I, de Man-van Ginkel J, Lammers J, Schuurmans M, Trappenburg J. Determinants of activation for self-management in patients with COPD. Int J Chron Obstruct Pulmon Dis. 2016;11:1757-66.

5. Halding A, Grov E. Self-rated health aspects among persons living with chronic obstructive pulmonary disease. Int J Chron Obstruct Pulmon Dis. 2017 Apr 12;12:1163-1172.

6. Fotokian Z, Mohammadi Shahboulaghi F, Fallahi-Khoshknab M, Pourhabib A. The empowerment of elderly patients with chronic obstructive pulmonary disease: Managing life with the disease. Plos One 2017;12(4):e0174028.

**Keywords**

COPD, Chronic obstructive pulmonary disease, Medication adherence, Social support.

### O133 The organizational commitment of health professionals (doctors, nurses and auxiliaries) in two public hospitals in Cape Verde

#### Jacqueline Delgado^1^, António Nunes^1,2^, Amélia Nunes^1^

##### ^1^Universidade da Beira Interior, 6201-001 Covilhã, Portugal; ^2^Núcleo de Estudos em Ciências Empresariais, 6200-209 Covilhã, Portugal

###### **Correspondence:** António Nunes (anunes@ubi.pt)

**Background**

The organizational commitment (OC) has its origin in the “*Side bets*” theory, representing the result of the accumulation of bets, which can be lost in a situation where the interruption of an activity occurs [1] The terms are understood as the maintenance of the belonging to the organization, being something of value in which the individual invested [2]. That is, while the individual works, creates bonds, commits himself and goes investing in the organization. The three-dimensional model [3] identifies the three dimensions of OC: the affective commitment, which consists in the feeling or desire to participate in the organization; the continuance commitment, which consists in the obligation to remain in the organization; and finally, the normative commitment, which consists in the worker's need to remain in the organization.

**Objective**

The objective of this study is to measure the OC levels, in its several dimensions, on the health professionals (physicians, nurses and auxiliaries) in two public hospitals in Cape Verde, considering the importance of sociodemographic variables (age, gender, marital status and academic qualifications) and Working context (work income, seniority in the company, type of contract and Hierarchical position) for the CO levels revealed.

**Methods**

The study used a quantitative methodology to evaluate the impact of sociodemographic and professional context variables on OC levels. In order to measure OC, we used the scale of three components: affective, normative and calculative [3], adapted for the Portuguese language in 2008 [4]. The sample consisted of 224 health professionals.

**Results**

The scale presented good levels of internal consistency (Cronbach's alpha of 0.85), with median OC values correlating positively with age; simultaneously, low OC levels were identified in higher education levels and High values of OC were identified in lower education levels. Finally, OC levels were also significantly higher for the less qualified professionals, auxiliaries showed the highest levels while the doctors showed the lowest levels of OC.

**Conclusions**

It is emphasized the positive and statistically significant relationship between age and OC, implying higher OC levels in the higher age groups, as identified in previous studies [5-8]. The inverse relation between OC levels and levels of academic qualifications, as identified by other authors [2-3, 5,8-9], is also a subject of interest. As well as the fact that the lower levels of OC appear in the most qualified professions: doctors and nurses, a not treated aspect in the literature and that characterizes the health professionals of Cape Verde.

**References**

1. Becker HS. Notes on the concept of commitment. Am J Sociol 1960;66(1):32-40.

2. Meyer JP, Allen NJ. Testing “side-bet theory” of organizational commitment: some methodological considerations. J Appl Psychol 1984;69(3):372-378.

3. Meyer JP, Allen NJ. A three-component conceptualization of organizational commitment. Hum R manage R 1991;1(1):61-89.

4. Nascimento JL, Lopes A, Salgueiro MDF. Estudo sobre a validação do “Modelo de Comportamento Organizacional” de Meyer e Allen para o contexto português. Comp.Org Gestão; 2008.

5. Mathieu JE, Zajac DM. A review and meta-analysis of the antecedents, correlates, and consequences of organizational commitment. Psychol Bull 1990;108(2):171.

6. Addae HM, Praveen KP, Velinor N. Role stressors and organizational commitment: public sector employment in St Lucia. Int J Manpow 2008;29(6):567-582.

7. Allen NJ, Meyer JP. The measurement and antecedents of affective, continuance and normative commitment to the organization. J Occup Organ Psychol 1990;63(1):1-18.

8. Angle HL, Perry JL. An empirical assessment of organizational commitment and organizational effectiveness. Adm Sci 1981;Q:1-14.

9. Mowday RT, Steers RM, Porter LW. The measurement of organizational commitment. J Vocat Behav 1979; 14 (2):224-247.

**Keywords**

Organizational commitment, Health professionals, Physicians nurses and auxiliaries, Cape verde (Africa).

### O134 Sleep quality and food intake of high school students

#### Ana SC Carvalho^1^, Adília P Fernandes^2^, Josiana A Vaz^2^, Ana B Gallego^3^, Matilde S Veja^3^

##### ^1^Unidade Local de Saúde do Nordeste, 5301-852 Bragança, Portugal; ^2^Escola Superior de Saúde, Instituto Politécnico de Bragança, 5300-146 Bragança, Portugal; ^3^Universidad de León, 24071 León, Spain

###### **Correspondence:** Ana SC Carvalho (ana.s.coelho@hotmail.com)

**Background**

Poor sleep quality is associated with increased food intake and poor diet quality [1]. People with lack of sleep show a positive correlation between free time and food intake and also experience hormonal and brain changes that drive the intake of food with high calorific value [1-3]. In addition, scientific research has shown a healthy and balanced diet to positively influence the quality of sleep [1].

**Objective**

The present study was set out to assess the sleep quality of high school students in Bragança county, and its association with food intake.

**Methods**

The study used non-experimental, analytical and transversal methodology, of epidemiological character and with a quantitative approach. It was intended to carry out the study in a population of 862 high school students. However, due to consent being required from both legal guardians and students, a smaller sample of 345 students was obtained. The data was collected in May 2017 through a questionnaire that included the Pittsburgh Sleep Quality Index (PSQI), validated for the Portuguese population.

**Results**

Throughout the study and following PSQI analysis, it was concluded that 39.71% (n = 137) of participants showed poor quality of sleep (PSQI > 5 points). The correlation between sleep quality and food intake was assessed and a statistically significant association was found between the quality of sleep and the intake of snacks (X^2^ = 17.144; p = 0.000), sugary products (X^2^ = 18.603; p= 0 .000), fast-food (X^2^ = 12.353; p = 0.002) and ready meals (X^2^ = 14.852; p = 0.000). The risk of suffering from poor sleep quality is higher in young populations who frequently eat snacks ([OR]: 2.811; 99%), sugary products ([OR]: 1.901; 95%), fast-food ([OR]: 4.000; 99%) and ready meals ([OR]: 5.621; 95%) in comparison with young populations who rarely eat this sort of food. The sleep quality is also significantly related with the number of meals young people have in a day (X^2^ = 7.580; p = 0.023). The risk of having poor quality sleep is 2.240 times higher in young people who rarely eat 4-6 meals a day.

**Conclusions**

A correlation between sleep quality and food intake in the sampled students was seen. The risk of having poor quality of sleep is higher in students who frequently eat a high calorie diet and also in students who rarely have 4-6 meals a day. There are several connections between sleep quality and eating habits. Sleep promotion and its connection with standard diets should be included as an essential part of community empowerment for health-promoting lifestyles [1,4,5].

**References**

1. McNeil J, Doucet E, Chaput JP. Inadequate Sleep as a Contributor to Obesity and Type 2 Diabetes. Canadian Journal of Diabetes. 2013;37:103-108.

2. Dewald JF, Meijer AM, Oort J, Kerkhof GA, Bogels SM. The influence of sleep quality, sleep duration and sleepiness on school performance in children and adolescents: A meta-analytic review. Sleep Medicine Reviews, 2010;14:179–189.

3. Paiva T. Bom Sono, Boa Vida. Cruz Quebrada: Oficina do Livro; 2008.

4. Lakshman R, Elks CE, Ong KK. Childhood obesity. Circulation 2012;126(14):1770-1779.

5. Direção Geral da Saúde Programa Nacional de Saúde Escolar. Lisboa: Ministério da Saúde de Portugal; 2015.

**Keywords**

Sleep Quality, Food intake, Balanced diet.

### O135 Education matters!!! The link between childhood obesity and parents’ level of education

#### Ricardo Melo^1^, Ana Inácio^1^, Mariana Pereira^1^, Miguel Santos^1^, Simão Sousa^1^, Francisco Campos^1^, Fernando Martins^1,2^

##### ^1^Applied Sport Sciences Research Unit, Coimbra Education School, Polytechnic Institute of Coimbra, 3030-329 Coimbra, Portugal; ^2^Instituto de Telecomunicações, University of Beira Interior, 6201-001 Covilhã, Portugal

###### **Correspondence:** Ricardo Melo (ricardo.es.melo@gmail.com)

**Background**

Obesity is a public health problem in most developed countries [1,2]. In Portugal this scenario is very serious because it stands as one of the European countries with more obese children [3,4], which is associated to poor eating habits, low level of physical activity, and sedentary lifestyles [2].

**Objective**

The objectives of this investigation are: I) to determine the prevalence of overweight/obesity in elementary school children; II) to compare children’s levels of body mass by age and gender; III) to verify correlations between children’s levels of body mass and family socio-demographic characteristics.

**Methods**

The sample was composed by 294 children between 5-9 years old (M ± SD = 7.35 ± 1.18 years old; 147 female) of the 10 elementary schools from the “Agrupamento de Escolas de Montemor-o-Velho (AEMMV)” (Coimbra-Portugal). Data was collected from September to December 2017. Family socio-demographic characteristics data were collected using a survey questionnaire applied to parents of participating children. Weight was evaluated using a Tanita Body Composition Monitor (model BC-420 SMA). Height was calculated using a stadiometer. Body Mass Index (BMI) was calculated using the formula weight/height^2^. The definition of underweight (level 1), normal weight (level 2), overweight (level 3) and obesity (level 4) was based on the tables in use by the Portuguese Directorate-General for Health [5], which correlates BMI with percentile tables. Data analysis was conducted using IBM SPSS (version 24.0, Chicago, USA) and a statistical significance of 10.0% was defined.

**Results**

Results of this study show that 17.7% of the evaluated children are overweight and 16.3% are obese (34.0% are overweight/obese). No significant statistical differences were observed by gender (Mann-Whitney = 10416; p = 0.529) or by age (Kruskal-Wallis test = 4.01; p = 0.405). Results of Spearman correlation test (r) also evidence not existing significant statistical relations between levels of body mass and parents’ age (mother: r = -0.031; p = 0.608; father: r = 0.015; p = 0.797) or with household composition (r = -0.040; p = 0.499). However, a negative correlation exists between body mass levels and parents’ education (mother: r = -0.136, p = 0.019; father: r = -0.158, p = 0.006) evidencing that the higher the level of education of the parents the lower the prevalence of high levels of body mass (overweight/obesity).

**Conclusions**

Despite policies to tackle obesity are being implemented, results of this study show a high prevalence of overweight/obesity children’s in the AEMMV. Results also confirm that parents' education is a strong social health determinant [1]. This study suggests that public authorities need to implement more efficient programs (e.g. nutrition and physical activity) at schools and community to promote active and healthier lifestyles.

**Acknowledgements**

This work is funded by FCT/MEC through national funds and when applicable cofounded by FEDER - PT2020 partnership agreement under the project UID/EEA/50008/2013 and by QREN, Mais Centro - Programa Operacional Regional do Centro, FEDER (CENTRO-07-CT62-FEDER-005012; ID: 64765). The authors also would like to thank to: Agrupamento de Escolas de Montemor-o-Velho, Câmara Municipal de Montemor-o-Velho, and Unidade de Cuidados na Comunidade de Montemor-o-Velho.

**References**

1. OECD [internet]. Obesity Update 2017. Retrieved from https://www.oecd.org/els/healthsystems/Obesity-Update-2017.pdf

2. WHO [internet]. Adolescent obesity and related behaviours: trends and inequalities in the WHO European Region, 2002–2014 2017. Retrieved from http://www.euro.who.int/__data/assets/pdf_file/0019/339211/WHO_ObesityReport_ 2017_v3.pdf

3. Padez C, Fernandes T, Mourão I, Moreira P, Rosado V. Prevalence of overweight and obesity in 7-9-year-old Portuguese children: trends in body mass index from 19702002.Am J Human Biology. 2004;16 (6):670-678.

4. Venâncio P, Aguilar S, Pinto G. Obesidade infantil… um problema cada vez mais atual. Revista Portuguesa de Medicina Geral e Familiar 2012;28:410-416.

5. Divisão de Saúde Materna, Infantil e dos Adolescentes da Direcção Geral da Saúde. Actualização das Curvas de Crescimento. Circular Normativa Nº: 05/DSMIA; 2016.

**Keywords**

Body Mass Index, Education, Health, Obesity, Overweight.

### O136 Relationship between the -1562 C/T polymorphism in the MMP-9 gene and multiple sclerosis

#### Ana Valado^1^, Maria J Leitão^2^, Lívia Sousa^3^, Inês Baldeiras^4^

##### ^1^Departamento de Ciências Biomédicas Laboratoriais, Escola Superior de Tecnologia da Saúde de Coimbra, Instituto Politécnico de Coimbra, 3046-854 Coimbra, Portugal; ^2^Centro de Neurociências e Biologia Celular, 3004-504 Coimbra, Portugal; ^3^Serviço de Neurologia, Centro Hospitalar e Universitário de Coimbra, 3000-075 Coimbra, Portugal; ^4^Faculdade de Medicina, Universidade de Coimbra, 3004-504 Coimbra, Portugal

###### **Correspondence:** Ana Valado (valado@estescoimbra.pt)

**Background**

Matrix metalloproteinases (MMPs), particularly MMP-9, have showed an association with the influx of inflammatory cells into the CNS, disruption of the blood brain barrier and demyelination in Multiple Sclerosis (MS). The transcriptional activity of the MMP-9 gene is influenced by the -1562 C/T polymorphism in the promoter region of the gene, and the T alelle has been suggested as a genetic risk factor for MS.

**Objective**

To investigate the presence of the -1562 C/T polymorphism in the MMP-9 gene in healthy controls and MS patients and its association with clinical course of the disease.

**Methods**

Whole blood DNA was extracted from 169 patients (143 RRMS, 20 SPMS, 6 PPMS) and 186 controls, and the presence of the polymorphism was detected by PCR-RFLP. Quantification of MMP-9 was performed in 96 patients and 63 controls by ELISA. Data from patients was analysed for associations between the polymorphism distribution and clinical factors (gender, age at onset, disease duration, EDSS score and disease course).

**Results**

The -1562 T allele was present in 39 patients and 41 controls, with no significant difference between groups (p = 0.533). However, in MS patients, but not in controls, more women presented with the -1562 T allele than men (p = 0.014). In patients, the distribution of the polymorphism was not significantly associated with age at onset (p = 0.759), disease duration (p = 0.309), progression of the disease (p = 0.121) or disability status (p = 0.180). The levels of MMP-9 in serum were significantly higher in MS patients compared to controls (p = 0.001). There was also an increase in serum MMP-9 values in controls that carried the T allele (p = 0.003), but not in MS patients.

**Conclusions**

The -1562 C/T polymorphism, at least in our population, does not seem to be a susceptibility risk factor for MS. However, in patients, there seems to be an association between the T allele with the female gender.

**Keywords**

-1562C/T polymorphism, MMP-9, MS.

### O137 Falls prevention in older people living in nursing homes in Northern Portugal

#### Isabel Lage, Odete Araújo, Manuela Almendra, Fátima Braga, Rui Novais

##### School of Nursing, University of Minho, 4704-553 Braga, Portugal

###### **Correspondence:** Odete Araújo (odete.araujo@ese.uminho.pt)

**Background**

Falls in older people are the leading cause of injury-related mortality and morbidity. People aged 65 and older have the highest risk of falling, with 30% of people older than 65 [1]. A fall can have significant adverse outcomes including injury, hospitalization and admission to long term care, development of fear of falling, activity restrictions and social isolation [2, 3].

**Objective**

The aim of this study was to describe the risk of falling in older people living in nursing homes in northern Portugal.

**Methods**

A descriptive correlational study was conducted in this research. A total of 833 participants (mean age 83 years) were recruited from 14 nursing homes in Northern Portugal. The statistical analysis of the data was performed using Statistical Package for Social Sciences (SPSS®) version 22.0, with descriptive and inferential statistical analysis with a significance level of 0.05.

**Results**

The results showed that the older men have less probability of falling in comparison with older women (OR = 0.581). In addition, older people able to walk independently and talk also have less probability of falling (OR = 0.431, OR = 0.360). In opposition, older people with walking difficulties or using technical aids have high risk of falling (OR = 1.944 e OR = 1.518).

**Conclusions**

These findings support the idea that ongoing assessment could be more important than the admission assessment, in identifying risk factors for falls in older people after institutionalization, in order to prevent falls.

**References**

1. NICE. Falls: assessment and prevention of falls in older people. UK: NICE accredited; 2013.

2. Pellicer García B, Juárez Vela R, Delgado Sevilla D, Redondo Castan LC, Martínez Abadía B, Ramón Arbués E. [Prevalence and profile of the elderly home care valid suffering in a private residence falls]. Revista de enfermería. 2013;36(12):8-16.

3. Yingfeng Z. Falls in older people in long-term care. Lancet. 2013;381 (9873):1179.

**Keywords**

Falls, Older people, Nursing homes.

### O138 “+ COOLuna” – intervention program of physiotherapy in schools at ACeS Baixo Vouga

#### Vitor Ferreira^1,2^, Ana Oliveira^1^, Maritza Neto^1^, Marta Santo^1^

##### ^1^Agrupamento de Centros de Saúde do Baixo Vouga, 3800-159 Aveiro, Portugal; ^2^School of Health, University of Aveiro, 3810-193 Aveiro, Portugal

###### **Correspondence:** Vitor Ferreira (vitorfontesferreira@gmail.com)

**Background**

Musculoskeletal pain in children is one of the most common reasons to seek medical attention. The most common musculoskeletal pain conditions are nonspecific or idiopathic and include regional pain in the spine, with a high prevalence [1]. Multifactorial causes are indicated, like social, psychological, physiological and environmental factors [2, 3]. Within the environmental factors, the carriage of schoolbags is pointed out as a factor that contributes to the high prevalence of musculoskeletal pain [3-5]. However, some studies report that the weight of schoolbags has little influence on the perception of pain, mainly in the spine [6, 7]. Nevertheless, musculoskeletal pain in childhood can persist throughout adolescence and increases the risk of experiencing chronic pain in adulthood [8-10]. At this phase, adolescents undergo a period of accelerated muscle-skeletal growth and development, with spinal structures being sensitive to external aggressions [11].

**Objective**

The aim of this study was to evaluate musculoskeletal pain due to schoolbag carriage in terms of prevalence, intensity and predisposing risks factors in students of 5th grade in schools of the range of community health centres of Aveiro region, during the school year of 2016-2017.

**Methods**

A cross-sectional study was design. The presence, intensity and duration of pain was assessed using a body chart and numeric rating scale for pain. Predisposing risk factors was assessed by means of an *ad hoc* questionnaire.

**Results**

A total of 960 children (male 51.1%: female 48.6%) with a mean age of 10.4 years (± 7.6) were included. The majority had backpacks (96.6%) and 82.4% (n = 775) carried the backpack over 2 shoulders. The mean schoolbag weight (4.9 ± 1.3 kg) represented a mean % body weight (%BW) of 13.0% (± 4.8). Only 29.3% carried schoolbags that were ≤ 10 %BW. The majority (79.9%) carried schoolbags to school for ≤ 15 min. The prevalence of musculoskeletal pain was reasonable (37.8%), and in the region of spine was low (16.0%). Multiple linear regression model indicated that pain is only explained by the number of hours of physical activity (negative correlation: r = -0.367) in 12.4% (R^2^ = 0.124; p = 0.001, SEE = 0.143).

**Conclusions**

This study highlights the need to consider the multifactorial nature of musculoskeletal pain in children, and also the need to reinforce protective factor of physical exercise in future prevention programs dedicated to children.

**References**

1. Swain MS, Henschke N, Kamper SJ, Gobina I, Ottova-Jordan V, Maher CG. An international survey of pain in adolescents. BMC public health. 2014;14:447.

2. Paananen MV, Taimela SP, Auvinen JP, Tammelin TH, Kantomaa MT, Ebeling HE, et al. Risk factors for persistence of multiple musculoskeletal pains in adolescence: a 2-year follow-up study. European Journal of Pain. 2010;14(10):1026-32.

3. Stinson J, Connelly M, Kamper SJ, Herlin T, Toupin April K. Models of Care for addressing chronic musculoskeletal pain and health in children and adolescents. Best practice & research Clinical rheumatology. 2016;30(3):468-82.

4. Iyer SR. An ergonomic study of chronic musculoskeletal pain in schoolchildren. Indian journal of pediatrics. 2001;68(10):937-41.

5. Noll M, Candotti CT, da Rosa BN, Loss JF. Back pain prevalence and associated factors in children and adolescents: an epidemiological population study. Revista de Saúde Pública. 2016;50:31.

6. Aprile I, Di Stasio E, Vincenzi MT, Arezzo MF, De Santis F, Mosca R, et al. The relationship between back pain and schoolbag use: a cross-sectional study of 5,318 Italian students. The spine journal : official journal of the North American Spine Society. 2016;16(6):748-55.

7. Dianat I, Sorkhi N, Pourhossein A, Alipour A, Asghari-Jafarabadi M. Neck, shoulder and low back pain in secondary schoolchildren in relation to schoolbag carriage: should the recommended weight limits be gender-specific? Appl Ergon. 2014;45(3):437-42.

8. Hestbaek L, Leboeuf-Yde C, Kyvik KO, Manniche C. The course of low back pain from adolescence to adulthood: eight-year follow-up of 9600 twins. Spine (Phila Pa 1976). 2006;31(4):468-72.

9. Siivola SM, Levoska S, Latvala K, Hoskio E, Vanharanta H, Keinanen-Kiukaanniemi S. Predictive factors for neck and shoulder pain: a longitudinal study in young adults. Spine (Phila Pa 1976). 2004;29(15):1662-9.

10. Hakala P, Rimpela A, Salminen JJ, Virtanen SM, Rimpela M. Back, neck, and shoulder pain in Finnish adolescents: national cross sectional surveys. Bmj. 2002;325(7367):743.

11. Goodburn EA, Ross DA. A Picture of health? : a review and annotated bibliography of the health of young people in developing countries / undertaken World Health Organization; 1995.

**Keywords**

Physiotherapy, Schoolbags, Musculoskeletal pain, Children.

### O139 Looking over Portuguese school-aged children lifestyles: results from a pilot study

#### Goreti Marques, Ana R Pinheiro, Fátima Ferreira, Daniela Simões, Sara Pinto

##### Escola Superior de Saúde de Santa Maria, 4049-024 Porto, Portugal

###### **Correspondence:** Goreti Marques (goreti.marques@santamariasaude.pt)

**Background**

Childhood obesity is considered one of the new epidemics of the 21st century. This study is part of a largest project regarding the improvement of healthy lifestyles in school-aged children, through a transdisciplinary team.

**Objective**

To describe food consumption and sport activities of Portuguese school-aged children.

**Methods**

An exploratory/descriptive pilot-study was conducted with third grade school-aged students from two Portuguese primary schools. Data were collected during through a self-filling form focusing on socio-demographic variables, sport activities and anthropometric measures (sex, age, house hold composition, practice of at least 60 minutes/week of sport activities outside school, weight, height). A booklet was used during five consecutive days to register food consumption. The study was previously approved by an Ethics Committee, and by the National Data Protection Commission (NDPC no.1704/2015). The informed consent of the child’s legal representative was signed. Data were analysed using the SPSS®-version 24.0.

**Results**

Preliminary results included 109 school-aged children (mean age = 7.5 years old; mean weight = 28.50 kg; mean height = 131.60 cm). Regarding the Body Mass Index (BMI), 65.1% of the children were considered to have normal weight, 11.9% overweight, and 8.3% obesity; underweight emerged in 14.7% of children. The consumption of fruit/vegetables was significantly greater (p < 0.05) in underweight children when compared with normal weight and overweight/obese children. The average consumption of fat/oil, and sugary/salty products seemed smaller in underweight children and greater in overweight/obese children, while the consumption of dairies/meat/fish/eggs, cereals and their derivatives, tubers and water seemed similar; however, statistically significant differences were not found (p > 0.05). Most children (77.1%) performed at least 60 minutes/week of sport activities outside school (66.7% practice only one type, 25.0% practice two, and 8.3% practice three different sports per week). Food consumption was not significantly different between children that practiced at least 60 min/week of sports outside school comparing to the children who didn’t.

**Conclusions**

Though most children have normal weight, data show important abnormalities in BMI. The consumption of fruit/vegetables appears to be increased in underweight children and decreased in overweight/obese children, which highlights the need for more detailed research. Food consumption does not seem to differ depending on the practice of outside-school sports. Further stages must bring the development of a transdisciplinary healthcare program to improve healthy lifestyles among school-aged children.

**Acknowledgements**

This work was funded by project NORTE-01-0145-FEDER-024116.

**Keywords**

Childhood obesity, Food consumption, Sport activities, Health promotion.

### O140 Dating violence in university context: practices, beliefs and impacts on the health of victims

#### Sofia Neves^1,2^, Ana Sousa,^1^ Joana Topa^1,2^, Janete Borges^1^

##### ^1^Instituto Universitário da Maia, 4475-690 Maia, Portugal; ^2^Centro Interdisciplinar de Estudos de Género, Instituto Superior de Ciências Sociais e Políticas, Universidade de Lisboa, 1300-663 Lisboa, Portugal

###### **Correspondence:** Sofia Neves (asneves@ismai.pt)

**Background**

Dating violence is an obvious and worrying social and health problem with serious consequences for its victims. It is characterized as a pattern of coercive and abusive tactics employed by one partner in a relationship to gain power and control over the other partner. It can take many forms, including physical violence, coercion, threats, intimidation, isolation, and emotional, sexual or economic abuse and occurs in the context of intimate heterosexual or homosexual/lesbian relationships. This kind of violence seems to be supported on conservative and traditional gender norms and stereotypes.

**Objective**

The main objective of this study is to characterize university students' beliefs and practices regarding dating violence, identifying the impacts of this type of violence on the psychological, physical, sexual and social health of their victims.

**Methods**

Were used self-administered questionnaires and a socio-demographic survey for data collection: Gender Belief Inventory (Maia University Institute and Interdisciplinary Centre for Gender Studies, version for research, 2017) and the Inventory on Violent Youth Relations (University Institute of Maia and Interdisciplinary Centre for Gender Studies, version for research, 2017). These were applied to 200 university students (142 females and 55 males), aged 18-44 (M = 20.54; SD = 4.435) who were attending the Maia University Institute. Data analysis was performed using the statistical program IBM- Statistics Package for the Social Sciences (version 24).

**Results**

The results showed that 12.8% of students reported having been victims of some act of violence by someone with whom they maintain or maintained a relationship of intimacy. Men were identified as the main perpetuators, with women having the highest rates of victimization. With regard to the type of violence perpetuated, psychological and social violence appear as the most experienced by students. With regard to gender social beliefs, this study reveals that these students maintain conservative and traditional gender beliefs that continue to perpetuate violence. Regarding the impact of dating violence, there was awareness among respondents of the implications of this violence on the health of the victims.

**Conclusions**

This study shows that despite the efforts that have been made in the implementation of policies and projects to prevent gender violence, this has not been enough to finish its practice. The commitment to the implementation of gender equality programs in school education seems fundamental in order to prevent this public health problem.

**Keywords**

Dating Violence, University Students, Beliefs, Practices, Implications to health.

### O141 Defining clinical conditions in long-term healthcare as a first step to implement Time-Driven Activity Based Costing (TDABC)

#### Ana Sargento^1,2^, Ana Querido^3,4^, Henrique Carvalho^2^, Isa Santos^2^, Catarina Reis^2,5^, Marisa Maximiano^2,5^, Manuela Frederico^6^, Sandra Oliveira^7,8^, Susana Leal^7,9^

##### ^1^Center for Applied Research in Management and Economics, School of Technology and Management, 2411-901 Leiria, Portugal; ^2^School of Technology and Management, Polytechnic Institute of Leiria, 2411-901 Leiria, Portugal; ^3^Center for Innovative Care and Health Technology, Polytechnic Institute of Leiria, 2411-901 Leiria, Portugal; ^4^School of Health Sciences, Polytechnic Institute of Leiria, 2411-901 Leiria, Portugal; ^5^Center for Research in Informatics and Communications, Polytechnic Institute of Leiria, 2411-901 Leiria, Portugal; ^6^Nursing School of Coimbra, 3046-851 Coimbra, Portugal; ^7^School of Management and Technology, Polytechnic Institute of Santarém, 2001-904 Santarém, Portugal; ^8^Center for Health Studies and Research, University of Coimbra, 3004-504 Coimbra, Portugal; ^9^Life Quality Research Centre, 2001-904 Santarém, Portugal

###### **Correspondence:** Ana Sargento (ana.sargento@ipleiria.pt)

**Background**

Increasing healthcare costs is a concern of all developed countries. In Long-Term Healthcare (LTH) this is reinforced by population ageing and corresponding prevalence of chronic diseases. Thus, it is fundamental to accurately measure costs and outcomes in healthcare, improving value created for patients, *i.e.*, patient-centred health outcomes per monetary unit of cost [1, 2]. TDABC methodology applied to healthcare allows identifying the cost for each clinical condition in the full cycle of care, mapping processes, activities, resources and allocated time [3–5]. It has been mostly applied in acute-care settings, partly due to complexity of defining chronic condition [6].

**Objective**

This paper focuses on the cost component of a larger on-going research project (CARE4VALUE), aiming to enhance value creation in LTH providers and applied to a partner LTH unit. Specifically, the main objective is to define clinical conditions in the context of LTH, as a first step in the implementation of TDBAC.

**Methods**

Mixed qualitative and quantitative methods were applied, including: 1) three focus groups conducted with the health team of the LTH unit (physician, nurses, physiotherapist, psychologist, social assistant) to select, discuss and validate the criteria to define clinical conditions; 2) construction of a composite indicator and testing it over a sample of anonymized clinical data from 21 patients; 3) structured observation of processes taken throughout the full cycle of care of patients in different conditions. Qualitative data was submitted to content analysis and validated among participants. Quantitative data used in the composite indicator, based on validated scales, was subject to normalization, aggregation and sensitivity analysis.

**Results**

One consensual outcome of the focus groups was that, in LTH, the disease or cause of entrance is less relevant to costs than the overall complexity of the patient, entailing psychical, social, spiritual and psychic-mental dimensions. Accordingly, a multidimensional classification model of patients in four complexity levels was delivered, after being validated and receiving consensus from the LTH team. Additionally, it will include a logging tool and dashboard to integrate separate patient-centred information and aid patient classification in complexity conditions.

**Conclusions**

The completion of this step allowed progressing in the design and implementation of the cost model, which, in turn, will support value measurement, and enhancing of the focus LTH unit. Besides, all involved professionals stated that their engagement in this phase of the project generated exceptional opportunities for interdisciplinary meetings and debate, contributing to closer ties between different areas of LTH.

**References**

1. Porter ME, Kaplan RS. How to pay for health care. Harv Bus Rev. 2016 Jul-Aug;94(7-8):88-98, 100, 134.

2. Schupbach J, Chandra A, Huckman RS. A Simple Way to Measure Health Care Outcomes. Havard Bus Rev [Internet]. 2016; Disponível em: https://hbr.org/2016/12/a-simple-way-to-measure-health-careoutcomes

3. Crott R, Lawson G, Nollevaux MC, Castiaux A, Krug B. Comprehensive cost analysis of sentinel node biopsy in solid head and neck tumors using a time-driven activity-based costing approach. Eur Arch Oto-Rhino-Laryngology. 2016;273(9):2621–2628.

4. Alaoui S El, Lindefors N. Combining time-driven activity-based costing with clinical outcome in cost-effectiveness analysis to measure value in treatment of depression. PLoS One. 2016;11(10): e0165389.

5. Keel G, Savage C, Rafiq M, Mazzocato P. Time-driven activity-based costing in health care: A systematic review of the literature. Health Policy (New York). 2017;121(7):755–763.

6. Nolte EE, McKee M. Caring for people with chronic conditions : a health system perspective. Eur Obs Heal Syst Policies Ser. 2008;XXI:259.

**Keywords**

Long-term healthcare, Time-Driven Activity Based Costing (TDABC), Clinical conditions, Patient-centered data, patient complexity.

### O142 Effects of aerobic land-based and water-based exercise training programs on clinical and functional parameters in older women

#### Rafael Oliveira^1,2^, Carlos T Santamarinha^3^, João Brito^1,2^

##### ^1^Research Unit in Quality of Life, Sport Sciences School of Rio Maior, Polytechnic of Santarém, 2040-413 Rio Maior, Portugal; ^2^Research Center in Sports Sciences, Health Sciences and Human Development, 6201-001 Covilhã, Portugal; ^3^City Hall of Esposende, 4740-223 Esposende, Portugal

###### **Correspondence:** Rafael Oliveira (rafaeloliveira@esdrm.ipsantarem.pt)

**Background**

In Portugal, most exercise training programs are offered by municipalities, seasonally, by 8 to 10 months.

**Objective**

The aim of the study was to access the clinical and functional effects of the application of different fitness exercise training programs, which included aerobic fitness group classes, with calisthenics exercises and water-based exercise, for nine months, to older women.

**Methods**

In the study, 96 active older women participated. They were divided in four exercise groups: 2xland-based group (GA, n=21; age 71.46±9.75 years; body weight 72.44±11.85 kg; height 153.82±5.83cm); 2xwater-based group (GB, n=9; age 70.10±9.98 years; body weight 70.48±10.92 kg; height 153.68±5.64cm); group of 1xland plus 2xwater-based (GC, n=7, age 71.35±8.32 years; body weight 73.42±11.20 kg; height 154.39±5.01cm), group of 2xland plus 2xwater-based exercise (GD, n=39, age 71.46±7.38 years; body weight 71.70±11.66 kg; height 154.23±6.82 cm). Clinical parameters were also accessed, such as, fast glucose, triglycerides, rest blood pressure, rest heart rate frequency (FCR) and functional parameters [1]: resistance of upper and lower limbs, agility and aerobic capacity. The training intensity of the programs was moderate, 10-14 on the “Rate of Perceived Exertion” scale [2], run accordingly [3]. It was use inferential statistic through T-test to compare baseline *vs* post-training.

**Results**

After nine months of intervention, the main results were at fast: glycemia (GA=116.0±12.11 vs 101.50±13.36 mg/dL; GC= 120.43±15.34 vs 100.47±11.65 mg/dL; GD = 127.29±36.60 vs 111.23±29.18 mg/dL); triglycerides (GA= 288.13±136.78 vs 158.13±47.24 mg/dL; GC= 295.94±112.92 vs 153.63±101.96 mg/dL; GD= 244.79±122.41 vs 144.98±69.27 mg/dL), FCR (GD=71.34±11.26 vs 66.31±8.68 bpm); aerobic capacity (GB= 541.88±51.03 vs 605.0±31.12 m ; GD=127.29±174.06 vs, 111.23±131.65 m), in resistance of lower limbs (GA=16.57±5.19 vs 18.90±5.07 repetitions; GB=14.89±5.21 vs 19.11±4.83 repetitions ; GD=20.54±5.38 vs 22.87±6.39 repetitions) and agility (GA= 7.94±3.52 vs 8.82±2.91 seconds; GB=8.86±4.09 vs 6.53±2.40 seconds; GC=7.90±4.56 vs 6.81±3.78 seconds; GD 6.13±1.66 vs 5.48±1.87 seconds), p < 0.05 for all. Also, it was observed that there were correlations between aerobic capacity and triglycerides; fast glucose and triglycerides.

**Conclusions**

The results showed a positive effect in all exercise training programs offered by municipality of Esposende, for clinical and functional parameters in older women. Groups of land-based exercise, at least twice a week, seam to lead to better results. The study supports the role of physical exercise to improve hemodynamic, lipid profile and functional parameters as reported previously by another similar study [4]. In addition, this study also revealed efficiency to improve clinical parameters that were not studied yet.

**References**

1. Rikli R, Jones C. The development and validation of a functional fitness test for community-residimg older adults. J Aging Phys Activ. 1999;7:129-161.

2. Borg G. Phychophysical bases of perceived exertion. Med Sci Sports Exerc. 1982;14:377-381.

3. American College of Sports Medicine, ACSM. ACSM’s Guidelines for exercise testing and prescription (9th ed). Philadelphia: Lippincott Williams and Wilkins; 2013.

4. Oliveira R, Santa-Marinha C, Leão R, Monteiro D, Bento T, Rocha RS, Brito JP. Exercise training programs and detraining in older women. J Hum Sport Exerc. 2017;12(1):142-155.

**Keywords**

Older women, Water-based exercise, Land-based exercise, Functional capacities, Clinical parameters.s

### O143 The influence of emotional intelligence in stigmatizing attitudes toward mental illness of undergraduate nursing students

#### Ana Querido^1,2^, Catarina Tomás^1,2^, Daniel Carvalho^3^, Marina Cordeiro^1,2^, João Gomes^3^

##### ^1^School of Health Sciences, Polytechnic Institute of Leiria, 2411-901 Leiria, Portugal; ^2^Center for Innovative Care and Health Technology, Polytechnic Institute of Leiria, 2411-901 Leiria, Portugal; ^3^Santo André Hospital, Hospital Center of Leiria, 2410-197 Leiria, Portugal

###### **Correspondence:** Ana Querido (ana.querido@ipleiria.pt)

**Background**

Health care professionals share the general public’s attitude towards people with mental illness, being generalized the harmful beliefs and the subsequent negative attitudes to these patients [1]. A significant correlation between emotional intelligence and mental illness stigma has been found.

**Objective**

To analyse the correlation between emotional intelligence and stigmatizing attitudes towards mental illness and to access the influence of this intelligence in those stigmatizing attitudes, among undergraduate nursing students.

**Methods**

It was performed a cross-sectional correlational study. Data was collected from a non-probabilistic sample of nursing students from a health school of the centre region of Portugal, using a questionnaire with sociodemographic questions, the Wong and Low Emotional Intelligence Scale (1-5) [2] and the Community Attitudes Toward the Mentally Ill Scale (40-200) [3]. Ethical procedures were taken into account during research according the Helsinki Declaration.

**Results**

Most of the nursing students inquired (N=335) were female (N=263). The sample had an average age of 21.69 years (SD=4.64), distributed by all levels of nursing graduation. Some (n=25) had already suffered from mental illness, mainly (N=17) humour disorders, and 41.2% referred to have regular contact with mental health patients in their lives. These nursing students showed a good emotional intelligence (Mean=3.62; SD=0.41), being higher in self-emotional appraisal (Mean=3.73; SD=0.58) and others’ emotional appraisal (Mean=3.85; SD=0.49). Stigmatizing attitudes towards mental illness are medium-low (Mean=122.15; SD=5.02), being more intense in authoritarianism (Mean=23.92; SD=4.44) and social restrictiveness (Mean=20.87; SD=4.94). Emotional intelligence is positively correlated with attitudes in community mental health ideology (R=0.133; p=0.015). Other’s emotional appraisal ability of emotional intelligence is correlated with attitudes regarding benevolence (R=0.276; p=0.000), community mental health ideology (R=0.221; p=0.000), authoritarianism (R=-0.156; p=0.005) and social restrictiveness (R=-0.254; p=0.000). Other’s emotional appraisal ability can explain some of the attitudes regarding authoritarianism (R^2^=0.024; F=8.138; p=0.005), benevolence (R^2^=0.076; F=27.136; p=0.000), social restrictiveness (R^2^=0.065; F=22.560; p=0.000) and community mental health ideology (R^2^=0.049; F=16.862; p=0.000). Regulation of emotions also influences benevolence attitudes (R^2^=0.016; F=16.578; p=0.000).

**Conclusions**

Postgraduate nursing students have good emotional intelligence and low stigmatizing attitudes towards mental illness. A significant correlation was found between emotional intelligence and stigmatizing attitudes, mostly positive, and an influence of this type of intelligence in the four areas of this kind of attitudes. These results enhance the importance of developing emotional intelligence, specially the ability of other’s emotional appraisal, in a way to improve attitudes toward mental illness.

**References**

1. Poreddi V, Thimmaiah R, Pashupu D, Ramachandra, Badamath S. Undergraduate Nursing students’ attitudes towards mental illness: Implications for specific academic education. Indian J Psychol Med. 2014;36(4):368-372.

2. Querido A, Tomás C, Carvalho D, Gomes J, Cordeiro M. Measuring emotional intelligence in health care students – revalidation of WLEIS-P. In: Proceedings of the 3rd IPLeiria’s International health Congress; 2016 maio 6-7; Leiria, Portugal. London: BMC Health Services Research; 2016. p. 87.

3. Taylor S, Dear M. Scaling community Attitudes Toward the Mentally Ill. Schizophrenia Bulletin. 1981, 7(2): 225-240.

**Keywords**

Mental health, Stigma, Nurse students, Emotional intelligence.

### O144 Functional decline in older acute medical in-patients

#### Cecília Rodrigues^1^, Denisa Mendonça^2^, Maria M Martins^3^

##### ^1^Centro Hospitalar do Porto, 4099-001 Porto, Portugal; ^2^Instituto de Ciências Biomédicas Abel Salazar, 4050-313 Porto, Portugal; ^3^Escola Superior de Enfermagem do Porto, 4200-072 Porto, Portugal

###### **Correspondence:** Cecília Rodrigues (ceciliarodrigue@gmail.com)

**Background**

Older patients hospitalised for acute illness are vulnerable to decline in basic self-care. This functional decline determines future health needs and can lead to negative health outcomes.

**Objective**

The aim of this study was to compare basic self-care needs in older acute medical in-patients between 2 weeks before hospitalization and discharge.

**Methods**

Single-centred, observational, and prospective cohort study. Data were collected between May and September 2017 and included 91 patients, aged 65 or older admitted to a medical ward of a 580-bed teaching hospital in Portugal. Performance in basic activities of daily living (BADL) at home (self-reported), at hospital admission (observed) and at discharge (observed) was collected. Functional status of the elderly patients at 2 weeks before hospitalization (baseline), at hospital admission, and at discharge was measured by the Katz Index. Differences in scores for BADL between baseline and admission, between admission and discharge, and between baseline and discharge were used to define pre-admission, in-hospital and overall functional decline.

**Results**

Pre-admission, in-hospital and overall functional decline occurred in 78.0%, 4.4% and 63.7% of the patients, respectively. Patients were independent on average in 3.63, 1.41 and 1.90 BADL 2 weeks before admission, at hospital admission and at discharge, respectively. In-hospital functional improvement occurred in 36.3% of the patients.

**Conclusions**

Due to their attitude to observe, support and guide patients and their 24 hours patient supervision, nurses play a key role in strategies to prevent functional decline in older patients. An adequate planning of nursing care that includes interventions to promote and maintain mobility, in the logic of self-care, can be a valuable contribution in the prevention of functional decline and in the reconstruction of independence in self-care, after a generative event of dependence.

**Keywords**

Functional decline, Elderly, Hospital outcomes.

### O145 Validation of the Weight Focused Feelings Scale in a sample of overweight and obese women participating in a community-based weight management programme

#### Cristiana Duarte^1^, Marcela Matos,^2^ James Stubbs^1^, Corinne Gale^3^, Liam Morris^4^, Paul Gilbert^3,5^

##### ^1^School of Psychology, Faculty of Medicine and Health, University of Leeds, LS2 9JT, Leeds, United Kingdom; ^2^Cognitive and Behavioural Centre for Research and Intervention, University of Coimbra, 3001-802 Coimbra, Portugal; ^3^College of Life and Natural Sciences, University of Derby, Kedleston Road, DE22 1GB, Derby, United Kingdom; ^4^Nutrition and Research Department, Slimming World, Clover Nook Road, DE55 4RF, Derbyshire, United Kingdom; ^5^Mental Health Research Unit, Kingsway Hospital, DE22 3LZ, Derby, United Kingdom

###### **Correspondence:** Cristiana Duarte (c.duarte@leeds.ac.uk)

**Background**

A significant body of literature suggests that negative emotions may undermine self-regulation of eating behaviour during/subsequent to weight loss attempts by influencing loss of control of eating behaviour [1]. Negative feelings related to body weight and shape seem to play a role in eating problems, with some studies suggesting that binge eating may occur as a means of momentarily avoiding/reducing negative affects related to body weight and shape [2, 3]. Recent evidence suggests that positive emotions may also relate to overeating in healthy and obese adults [4-6]. Nonetheless, research on emotions and eating focuses primarily on negative emotions and there is a lack of measuring relating positive emotions to eating.

**Objective**

The current study aimed to test the factorial structure and psychometric properties of the Weight Focused Feelings Scale (WFFS), an 11-item measure that assesses negative and positive feelings linked to body weight and shape.

**Methods**

2,236 women attending a community-based weight management programme participated in this study. Mean (SD) participants age was 41.71 (12.34) years and BMI was 31.62 (6.10) kg/m^2^. Data was randomly split in two independent data sets to conduct an Exploratory Factor Analysis (EFA) in 1,088 participants, and a Confirmatory Factor Analysis (CFA), in 1,148 participants.

**Results**

Results of the EFA indicated a two-factor structure: negative feelings (7 items) and positive feelings (3 items). Items presented factorial loading above .49 on the first factor, and above .64 on the second factor. The CFA confirmed the plausibility of this 2-factor model (X^2^_(41)_ = 283.771: p < .001; CFI = .96; TLI = .95; PCFI =.72; RMSEA = .07 [.06 to .08]). Standardized Regression Weights ranged from .55 to .68 in the Negative affect subscale; and .75 to .90 in the Positive affect subscale. Items' Squared Multiple Correlations ranged from .30 to .81. The subscales presented Composite Reliability values of .93 and .88, respectively. The two subscales were associated, in the expected direction, with measures of depressive, anxiety and stress symptoms, psychological wellbeing, shame, dietary disinhibition, restraint and susceptibility to hunger, and body mass index (BMI).

**Conclusions**

The WFFS is a valid measure to assess body weight and shape-related negative and positive emotions. This measure may be useful for future model testing examining the differential role of negative and positive emotions in eating behaviour and weight management.

**References**

1. Singh M. Mood, food and obesity. Frontiers Psychol. 2014;5:925.

2. Duarte C, Pinto-Gouveia J, Ferreira C. Ashamed and fused with body image and eating: Binge eating as an avoidance strategy. Clin Psychol Psychother. 2017;24(1):195-202.

3. Heatherton T, Baumeister R. Binge eating as escape from self-awareness. Psychol Bull 1991;110(1):86-108.

4. Bongers P, Jansen A, Havermans R, Roefs A, Nederkoorn C. Happy eating. The underestimated role of overeating in a positive mood. Appetite 2013;67:74-80.

5. Cardi V, Leppanen J, Treasure J. The effects of negative and positive mood induction on eating behaviour: A meta-analysis of laboratory studies in the healthy population and eating and weight disorders. Neuroscience & Biobehavioral Reviews in Cardiovascular Medicine 2015;57:299-309.

6. Evers C, Adriaanse M, Ridder DTD, Witt Huberts JC. Good mood food. Positive emotion as a neglected trigger for food intake. Appetite 2013;68:1-7.

**Keywords**

Negative and Positive emotions, Body weight and shape, Eating behavior, Factorial Analysis, Psychometric analysis.

### O146 Nurses’ perceptions of barriers for implementing EBP in a central hospital in the north of Portugal

#### Ana IC Teixeira^1,2,3,4^, António L Carvalho^3,4^, Cristina Barroso^4^

##### ^1^Centro Hospitalar São João, 4200-319 Porto, Portugal; ^2^Instituto de Ciências Biomédicas Abel Salazar, 4050-313 Porto, Portugal; ^3^Centro de Investigação em Tecnologias e Serviços de Saúde, 4200-450 Porto, Portugal; ^4^Escola Superior de Enfermagem do Porto, 4200-072 Porto, Portugal

###### **Correspondence:** Ana IC Teixeira (enf.anat@gmail.com)

**Background**

Evidence based practice (EBP) is defined as an integration of best research evidence with clinical expertise and patient values in clinical decision making [1, 2]. Research confirms positive outcomes when implementing EBP: patient safety; improved clinical outcomes; reduced healthcare costs and decreased variation in patient outcomes [3]. Considered as a standard of care, EBP has benefits for nurses, patients, general population, health care systems, as well as for research and education. Authors describe several types of obstacles to the use of research in practice: characteristics of the adopter, organization, innovation and communication [1]. Individual barriers include lack of knowledge and how to critique research studies; lack of awareness; colleagues not supportive of practice change and nurses feeling a lack of authority to change practice. Organizational barriers include insufficient time to implement new ideas; lack of access to research; and lack of awareness of available educational tools related to research [3]. The most important factor related to nurses’ EBP is support from their employing organizations to use and conduct research. Programme implementation is most likely to be successful when it matches the values, needs and concerns of practitioners. Concerning the development and implementation of competence for EBP, firstly, is important to identify the barriers.

**Objective**

Our objective is to explore and describe nurse’s perceptions of these barriers in our context.

**Methods**

This study is part of a larger one, namely: “Clinical Supervision for Safety and Care Quality” (C-S2AFECARE-Q). To answer our research question a convenience sampling strategy was employed to distribute 500 questionnaires to nurses working in a central hospital in the north of Portugal. Data was collected between April and July 2017. They returned 260 questionnaires, 98 of these with the answer to the following question: “*In your opinion, which are the barriers for the implementation of EBP in your context?*”. Data analyses were based on content analysis proposed by Bardin (1977).

**Results**

Barriers were allocated into categories: Organization; Leaders and Management; Professionals and evidence [4]. Nurses reported perceived barriers in all these levels. In the organization’ barrier, they identified lack of organizational culture; lack of support from management and outdated and unquestioned routines. They also reported insufficient support from the leaders. In individual terms, they identified negative attitudes, such as lack of motivation, resistance to change, lack of time and inadequate knowledge and skills.

**Conclusions**

Our results are consistent with other authors’ findings [5]. Nurses are clearly aware of the barriers in their context.

**References**

1. Munten G, van den Bogaard J, Cox K, Garretsen H, Bongers I. Implementation of Evidence-Based Practice in Nursing Using Action Research: A Review. Worldviews Evid Based Nurs. 2010;7(3):135-157.

2. Ubbink D, Guyatt GH, Vermeulen H. Framework of policy recommendations for implementation of evidence-based practice: a systematic scoping review. BMJ Open Journal. 2013;3:e001881.

3. Black AT, Balneaves LG, Garossino C, Puyat JH, Qian H. Promoting Evidence-Based Practice Through a Research Training Program for Point-of-Care Clinicians. J Nurs Adm. 2015;45(1):14-20.

4. Jylhä V, Oikarainen A, Perälä M, Holopainen A. Facilitating evidence-based practice in nursing and midwifery in the WHO European Region. World Health Organization; 2017 [cited 2018 Jan 18]. Available from: http://www.euro.who.int/en/health-topics/Health-systems/nursing-and-midwifery/publications/2017/facilitating-evidence-based-practice-in-nursing-and-midwifery-in-the-who-european-region-2017.

5. Pereira RPG, Cardoso MJ, Martins MA. Atitudes e barreiras à prática de enfermagem baseada na evidência em contexto comunitário. Revista de Enfermagem Referência. 2012;3(7):55-62.

6. Solomons N, Spross JA. Evidence-based practice barriers and facilitators from a continuous quality improvement perspective: an integrative review. Journal of Nursing Management. 2011;19(1):109-120.

**Keywords**

Nursing, Evidence-Based Practice, Barriers, Hospital health care.

### O147 Patient identification, patient safety and clinical audit

#### Cecília Rodrigues, Manuel Valente

##### Centro Hospitalar do Porto, 4099-001 Porto, Portugal

###### **Correspondence:** Cecília Rodrigues (ceciliarodrigue@gmail.com)

**Background**

The correct identification of the person under health care in an institution is a basic principle of a patient safety culture and quality of care provided. Failures associated with patient identification processes are the cause of medication errors, transfusions, complementary diagnostic and therapeutic screenings, invasive procedures performed on wrong persons, and other incidents of high severity.

**Objective**

Identify if all hospitalized patients have identification wristbands and check if the name is correct and readable.

**Methods**

In a 580-bed adult teaching hospital, between January and December 2017, on random days of each month, patients were audited for identification by wristband by a team of nurses with experience in clinical audit.

**Results**

In the 12 months studied, 161 audits were performed, resulting in 3,539 patients audited. From the total patients audited, 3,406 (96.2%) were correctly identified with wristbands. There were 133 failures: 113 patients had no identification wristband, 19 patients had an identification wristband, but the name was unreadable and 1 patient had a wristband with the wrong name. The rate of correctly identified patients increased progressively over the months: in the first month (January 2017) 89.3% of patients were correctly identified with a wristband; in December 2017, the rate of correctly identified patients was 95.0%. The partial results of this audit were disclosed in general risk management meetings with clinical services in March, June and September 2017.

**Conclusions**

Given the potential negative implications of the absence of identification of the person undergoing health care, these results indicate that there is a clear opportunity for improvement in patient identification. Clinical audit has proved to be an instrument for improving quality and safety, particularly in improving the identification of patients.

**Keywords**

Patient safety, Patient identification, Clinical audit.

### O148 Demographic differences in quality of life in elderly population of Tâmega e Sousa

#### Sara S Lima^1,2^, Raquel Esteves^1,2^, Clarisse Magalhães^1,2^, Fátima Ribeiro^1,2^, Lurdes Teixeira^1,2^, Ana Teixeira^1,2^, Fernanda Pereira^1,2^

##### ^1^Cooperativa De Ensino Superior Politécnico Universitário, 4585-116 Gandra, Paredes, Portugal; ^2^Instituto de Investigação e Formação Avançada em Ciências e Tecnologias da Saúde, 4585-116 Gandra, Paredes, Portugal

###### **Correspondence:** Sara S Lima (ssofialima@gmail.com)

**Background**

Aging population is associated with an increase in pathologies of prolonged evolution, making necessary to redirect and reorganize social and health structures, in order to meet the specific needs of the elderly population.

**Objective**

To characterize the demographic profile of the elderly population of the region of Tâmega e Sousa regarding quality of life as well as functionality level and social support; and to find differences in quality of life according to gender and age.

**Methods**

This cross-sectional study included 200 participants, 67% women with a mean age of 72 years old (SD=5.3). A sociodemographic questionnaire, SF-36 to assess quality of life (physical and mental dimension), Barthel Index to assess functionality level and Satisfaction with Social Support Scale to assess satisfaction with social support were used.

**Results**

For mental quality of life the mean score was 59.69 (SD=12.78) and 49.80 (SD=16.38) for physical quality of life. Satisfaction with social support was positively associated to both mental (r=.476, p<.001) and physical quality of life (r=.457, p<.001) as well as with functionality level (r= .386, p<.001; r=.458, p<.001, respectively). There were differences in mental (t(195)= -2.998, p=.003) and physical quality of life (t(195)= -3.358, p=.001) according to gender and according to age, *i.e.*, the youngest participants (< 71 years old) showed high mental (t(197)=-33.552, p<.001) and physical (t(197)=2.466, p=.015) quality of life.

**Conclusions**

The results highlight the role of social support and level of functionality in the quality of life of this population, emphasizing the need to foster the integration of this population into social programs and leisure time as well as to promote physical activity in order to improve the level of functionality. Physical activity improves independent living, reduces disability, and improves the quality of life of the elderly. Contrary to the literature, programs should be constructed taking into account that men presented worse quality of life and should be directed to the older ones, as expected. Therefore, results show the importance to implement multidisciplinary programs, tailored, with older people profiles, allowing the development of an integrated social and health response to the elderly population of this region.

**Keywords**

Aging, Eldely people, Quality of Life, Social Support, Functionality, Social and health structures.

### O149 Healthy lifestyles & literacy for health in work context- what trend?

#### Otilia Freitas^1,2^, Gregório Freitas^1,2^, Clementina Morna^1,2^, Isabel Silva^1,2^, Gilberta Sousa^1,2^, Rita Vasconcelos^3^, Luís Saboga-Nunes^4,5^, Estudantes Enfermagem^1^

##### ^1^Center for Health Technology and Services Research, University of Madeira, 9020-105 Funchal, Madeira, Portugal; ^2^Higher School of Health, 9020-105 Funchal, Madeira, Portugal; ^3^Faculty of Exact Sciences and Technology, University of Madeira, 9020-105 Funchal, Madeira, Portugal; ^4^institute of Environmental Health, Faculty of Medicine, University of Lisbon, 1649-028 Lisboa, Portugal; ^5^Institute of Sociology, University of Freiburg, 79098 Freiburg, Germany

###### **Correspondence:** Otilia Freitas (omsfreitas@uma.pt)

**Background**

In the National Plan for Occupational Health (PNSO) - 2nd Cycle 2013/2017, goals are established to increase health gains and guarantee the value of the workers’ health in groups of employers by those responsible for governance and society in general. One of the specific objectives is to promote health and work practices and healthy lifestyles at the workplace, concerning private sector companies and Public Administration [1].

**Objective**

To describe health lifestyles and health literacy of workers of a company of the tertiary sector of Região Autónoma da Madeira, Portugal.

**Methods**

Descriptive exploratory study, with a non-probabilistic convenience sample of 118 workers, with mean age of 45 years, predominantly male (88.1%). We used a sociodemographic data collection instrument, the FANTASTIC Lifestyle Questionnaire [2] (αC 0.725) and the European Questionnaire on Literacy for Health, Portuguese version [3], (αC of 0.97). A favourable opinion was obtained from an ethics committee and the ethical procedures inherent to this type of study were respected.

**Results**

It was found that 11.9% of the respondents presented a general level of Good (73 to 86), 55.9% of Very Good (85 to 102) and 32.2% of Excellent (103 to 120) lifestyle. Regarding general health literacy, 56.70% of the respondents had limited literacy levels, being 7.20% inadequate and 49.50% problematic. The trend towards limited literacy, observed in general literacy, remained in all three domains. Thus, in the area of Health Care, 48.3% of the respondents had limited levels of literacy, being 8.47% inadequate and 39.83% problematic. Concerning disease prevention, 44.06% of the respondents showed limited levels of literacy, being 11.86% inadequate and 32.20% problematic. In terms of health promotion, 47.46% of the respondents showed limited levels of literacy, being 11.02% inadequate and 36.44% problematic. There is a positive (ρ = 0.277) and statistically significant correlation between the general lifestyle score and the overall health literacy score (p value = 0.002).

**Conclusions**

The results suggest the importance of an intervention with integrated activities and promoters of healthy lifestyles and of health literacy, focusing strategically on health education in the work context, aiming to raise awareness among the target population.

**References**

1. DGS. Plano Nacional de Saúde Ocupacional 2013/2017. https://www.dgs.pt/saudeocupacional/programa-nacional4.aspx.

2. Saboga-Nunes L, Sorensen K, Pelikan J, Cunha M, Rodrigues E, Paixão E. Cross-cultural adaptation and validation to Portuguese of the European Health Literacy Survey (HLS-EU-PT). Atencion Primaria. 2014;46(1):13.

3. Silva AMM, Brito IS, Amado J. Adaptação e validação do questionário “Estilo de Vida Fantástico”: resultados psicométricos preliminares. Referência. 2011;3:650.

**Keywords**

Healthy lifestyles, Health literacy, Work context, Nursing.

### O150 3rd pressure ulcer prevalence study in community setting - CSAH-USIT

#### Manuela Dias, Ana Rocha

##### Unidade de Saúde da Ilha Terceira, Centro de Saúde de Angra do Heroísmo, 9700-121, Açores, Portugal

###### **Correspondence:** Manuela Dias (maria.mm.dias@azores.gov.pt)

**Background**

Pressure ulcers are a problem to health care institutions and professionals. In Azores Islands, there are few prevalence studies in community settings and the results point to prevalence rates of 18.5%, according to the Nursing Scientific Investigation Group (ICE) [1] in 2006, and of 26.49% according to Rodrigues and Soriano [2], in 2010. The National Security Patient Plan 2015-2020 [3] recommends that all health institutions should supervise this problem every six month, so “Gabinete de Saúde Comunitária” is already conducting the 3rd Pressure Ulcer Prevention Study.

**Objective**

This study aims to examine the prevalence rate of pressure ulcers in patients living at home, to characterize those patients as well as the most severe ulcers.

**Methods**

This was a descriptive cross-sectional study using a quantitative approach with a questionnaire, which took place during November’s first week, in 2017. The population was the one attending Angra Health Centre, assisted by nurses, in a community setting. Data was analysed using Excel 2007. The study was performed after the Health Centre’s approval. A sample of 26 patients, with pressure ulcers, 54% female, with a medium age of 79.09 years participated in the study. Most of the patients (46.15%) belonged to the age group between 80-89 years.

**Results**

The study reported a prevalence rate of 7.58%. There was a ratio of pressure ulcers *per* participant with pressure ulcer of 1.5, where the sacrum/coccyx was the main critical area. Most of the pressure ulcers recorded was category II (42.31%), with an evolution situated on 4.42 months. 77% had high risk of pressure ulcer development according to the Braden Scale; 92.31% of the patients had prevention devices on bed and only 31.25% had them on sitting chairs. Upon analysis of the three studies, we noticed that the prevalence rate had increased, the medium age decreased, with the largest group belonging to the group between 80-89 years old. There was also an increased number of the sacrum/coccyx localization in this group.

**Conclusions**

This study allowed us to analyse, briefly, the pressure ulcer context, in which nurses spend a large amount of time caring and showing some strategies for recovery. Targeting those findings should be implemented in some of these community settings, in order to improve the health quality of the patient.

**References**

1. Gomes LM. Prefácio In Grupo ICE. Investigação Científica em Enfermagem, Enfermagem e úlceras de pressão: Da reflexão sobre a disciplina às evidências nos cuidados. Angra do Heroísmo: Grupo ICE. 2008; p. 8-13.

2. Rodrigues A, Soriano J. Fatores influenciadores dos cuidados de enfermagem domiciliários na prevenção de úlceras por pressão. Revista de Enfermagem Referência. 2011;III(5):55-63.

3. Diário da República. Plano Nacional para a Segurança dos Doentes 2015-2020., 2.ª série — N.º 28 — 10 de fevereiro de 2015.

**Keywords**

Pressure Ulcer, Prevalence, Community settings, Nursing.

### O151 Relationship between health literacy, electronic health literacy and knowledge of sexually transmitted diseases in workers of a Portuguese hospital

#### Alice Gonçalves^1^, Anabela Martins^2^, Clara Rocha^3^, Diana Martins^1,4^, Isabel Andrade^3^, Margarida Martins^5^, Paula Vidas^6^, Paulo Polónio^7^, Fernando Mendes^1,8,9,10^

##### ^1^Biomedical Laboratory Sciences, Coimbra Health School, Polytechnic Institute of Coimbra, 3046-854 Coimbra, Portugal; ^2^Department of Physiotherapy, Coimbra Health School, Polytechnic Institute of Coimbra, 3046-854 Coimbra, Portugal; ^3^Department of Complementary Sciences, Coimbra Health School, Polytechnic Institute of Coimbra, 3046-854 Coimbra, Portugal; ^4^Institute for Research and innovation in Health Sciences, University of Porto, 4200-135 Porto, Portugal; ^5^Emergency Service, Hospital Distrital da Figueira da Foz, 3094-001 Figueira da Foz, Portugal; ^6^Cardiology Service, Hospital Distrital da Figueira da Foz, 3094-001 Figueira da Foz, Portugal; ^7^Laboratory Medicine Hospital, Hospital Distrital da Figueira da Foz, 3094-001 Figueira da Foz, Portugal; ^8^Biophysics Institute, Center for Neuroscience and Cell Biology-Institute for Biomedical Imaging and Life Sciences, Faculty of Medicine, University of Coimbra, 3004-504 Coimbra, Portugal; ^9^Center of Investigation in Environment, Genetics and Oncobiology, Faculty of Medicine, University of Coimbra, 3004-504 Coimbra, Portugal; ^10^Coimbra Institute for Clinical and Biomedical Research, University of Coimbra, 3094-001 Coimbra, Portugal

###### **Correspondence:** Alice Gonçalves (alicebvb@gmail.com)

**Background**

Health literacy (HL) can be defined as the individual capacity of obtaining and understanding information, making appropriate decisions about health. An adequate level of HL is fundamental for risk awareness and behaviour change, as well as for disease prevention. Electronic health literacy (eHL) can be useful in promoting health, but it demands the capacity of judgment towards the obtained information and the ability to work with new technologies. Due to the high prevalence of sexually transmitted diseases (STDs) in the general population, it’s important to evaluate and relate these health concepts.

**Objective**

To evaluate and relate levels of HL and eHL with STD knowledge of professionals of a Portuguese Hospital.

**Methods**

149 individuals from different professional categories, working at a medium size Portuguese Hospital, answered an anonymous questionnaire, composed of four parts: socio-demographic data, STDs knowledge, and the Portuguese version of both Newest Vital Sign (to assess the functional HL) and eHEALS (to assess the eHL).

**Results**

Altogether 66.0% of the sample had the possibility of an adequate HL; 59.2% of those individuals were health professionals and 76.3% had a high knowledge on STDs. 72.4% were female and 27.5% male, showing similar possibility of an adequate HL (66.7% and 61.0%, respectively). Concerning the individuals with the possibility of an adequate level of HL, 27.5% were 31-40 years old and 90.6% held a higher education degree, while 7.3% had only completed high school. In general, “*I know what health resources are available on the Internet*” and “*I have the skills I need to evaluate the health resources I find on the Internet*” had the highest mean (3.99) and “*I feel confident in using information from the Internet to make health decisions*” had the lowest with 3.08.

**Conclusions**

Individuals with higher education, especially in health sciences, have higher probability of an adequate HL. Age may also be an interfering factor, since the youth nowadays, has more access to information early in life. Our results also show that 50% of the studied population had the possibility of an adequate level of HL and a high knowledge in STDs, which is an average percentage for people who work at this hospital. This can be changed, using specific education procedures, such as lectures or seminars, to increase HL and knowledge on STDs.

**References**

1. Sørensen K, Van den Broucke S, Fullam J, Doyle G, Pelikan J, Slonska Z, et al. Health literacy and public health: A systematic review and integration of definitions and models. BMC Public Health;12(1):80.

2. Norman CD, Skinner HA. eHealth Literacy: Essential Skills for Consumer Health in a Networked World. J Med Internet Res. 2006;8(2):e9.

3. Norman CD, Skinner HA. eHEALS: The eHealth Literacy Scale. J Med Internet Res. 2006;8(4):e27.

**Keywords**

Health literacy, eHealth literacy, Risk behaviour, Health professionals.

### O152 Stigmatizing attitudes toward mental illness in future health professionals

#### Daniel Carvalho^1^, Catarina Tomás^2,3^, Ana Querido^2,3^, Marina Cordeiro^2,3^, João Gomes^1^

##### ^1^Santo André Hospital, Hospital Center of Leiria, 2410-197 Leiria, Portugal; ^2^School of Health Sciences, Polytechnic Institute of Leiria, 2411-901 Leiria, Portugal; ^3^Center for Innovative Care and Health Technology, Polytechnic Institute of Leiria, 2411-901 Leiria, Portugal

###### **Correspondence:** Daniel Carvalho (drscarvalho@gmail.com)

**Background**

Health professionals, like nurses, still have concerns and negative attitudes towards people with mental illness, despite development of mental health services [1]. Final year students were found to be less stigmatizing [2], being important to pay special attention to the education of these professionals and on-the-job training [1].

**Objective**

To access the levels of stigmatizing attitudes towards mental illness, and to characterize the determinants of these attitudes, developed by health students.

**Methods**

A quantitative, cross-sectional correlational study was performed. A non-probabilistic sample of health students from a school in the centre region of Portugal was considered, and data was collected with a questionnaire with sociodemographic questions, a question about perception on their knowledge about mental health (scoring 0 to 5) and the Community Attitudes Toward the Mentally Ill Scale (scoring 40 to 200) [3]. Ethical procedures were taken into account during research, according to the Helsinki Declaration.

**Results**

The students inquired (N=636) were representative of five health degrees, namely, Dietetics, Nursing, Physiotherapy, Speech Therapy and Occupational Therapy, mostly female (82.1%), with an average age of 21.35 (SD=4,29; Median=20). A low percentage (6.6%) had already suffered from a psychiatric disease, mainly humour disorders (4.1%) and 37.6% had regular contact with a patient with mental illness in their lives. The sample had medium-low levels of stigmatizing attitudes towards mental illness (Mean=122.72; SD=5.19), being these negative attitudes more relevant in authoritarianism (Mean=24.17; SD=4.34) and social restriction (Mean=20.53; SD=4.93) areas. Male students showed more positive attitudes towards authoritarianism (p=0.004) and social restriction (p=0.007) and females towards Benevolence (p=0.016) and Community Mental Health Ideology (0.004). Nursing students had more frequent stigmatizing attitudes and physiotherapy students showed better attitudes (p=0.021). Students who have had mental illness had more stigmatizing attitudes in general (p=0.039), regarding all areas (p<0.005) except for Community Mental Health Ideology. Students who had regular contact with a mental health patient, had more stigmatizing attitudes in general (0.031) and in authoritarianism (p=0.039).

**Conclusions**

It was found medium-low levels of stigmatizing attitudes towards mental illness among the health students inquired. There was also evidence that having a mental disorder or having regular contact with mental health patients can increase stigmatizing attitudes of health students. This highlights the importance of intervention in these students, not also to improve attitudes towards mental health patients, but also to decrease the self-stigma that was also identified by these results.

**References**

1. Ihalainen-Tamlander N, Vähäniemi A, Löyttyniemi E, Suominen T, Välimäki M. Stigmatizing attitudes in nurses towards people with mental illness: a cross-sectional study in primary settings in Finland. Journal of Psychiatric and mental Health Nursing. 2016;23(6-7):427-437.

2. Mas A, Hatim A. stigma in mental illness: attitudes of medical students towards mental illness. Med J Malaysia. 2002;57(4):433-444.

3. Taylor S, Dear M. Scaling community Attitudes Toward the Mentally Ill. Schizophrenia Bulletin. 1981;7(2):225-240.

**Keywords**

Mental health, Stigma, Health students, Attitudes.

### O153 Psychological symptoms and mental health stigma in teaching professionals

#### Catarina Tomás^1,2^, Ana Querido^1,2^, Marina Cordeiro^1,2^, Daniel Carvalho^3^, João M Gomes^3^

##### ^1^School of Health Sciences, Polytechnic Institute of Leiria, 2411-901 Leiria, Portugal; ^2^Center for Innovative Care and Health Technology, Polytechnic Institute of Leiria, 2411-901 Leiria, Portugal; ^3^Santo André Hospital, Hospital Center of Leiria, 2410-197 Leiria, Portugal

###### **Correspondence:** Catarina Tomás (catarina.tomas@ipleiria.pt)

**Background**

Teaching professionals are recognised to be submitted to a daily stress, partly due to dynamic interactions associated with the teaching role, which leads to experiences of psychological and psychosomatic symptoms [1]. Facing mental health problems influences stigma towards mental illness. In this situation they are predisposed to accept preconceptions leading to internalized stigma [2]. Research has referred teachers as having negative attitudes towards mental illness [3]. Considering that there is little known about teacher professionals in secondary and higher education [4], this study addresses mental health stigma and psychological symptoms in these settings.

**Objective**

To characterise psychological symptoms and mental health stigma in teaching professionals; identify the differences between high-school and higher education teachers stigma; correlate psychological symptoms and mental health stigma.

**Methods**

Cross-sectional correlational study, with a non-probabilistic sample of 96 Portuguese teaching professionals. Data were collected using an on-line questionnaire composed by socio-demographic information, the Portuguese version of Brief Symptom Inventory (BSI) and he Attribution Questionnaire to Measure Mental Illness Stigma (AQ27) [5]. Ethical procedures were taken into account during research, according to the Helsinki Declaration.

**Results**

Teaching professionals were mostly women (70.8%), aged between 30 and 62 years old (Mean=44.8; SD=7.86), 27.1% diagnosed with a mental disease and 41.1% referred contact with mental health patients. Teaching professionals revealed low levels of global psychological symptoms. Higher scores were found in Obsession-Compulsion (M=0.88; SD=0.73). A moderate level of stigma was found in the global sample (M=3.63; SD=0.74). Stigmatizing helping attitudes and behaviour were higher (M=6.50, SD=1.86). Although no difference was found in total stigma between groups, higher education professors rate responsibility higher than high-school teachers (M=3.06, SD=1.08 vs M=2.55, SD=0.97; p=0.02). Significant positive correlations were found between total stigma and psychological symptoms in Somatization, Obsession-Compulsion, Interpersonal Sensitivity, Depression, Anxiety and Psychoticism (p < 0.01).

**Conclusions**

Teaching professionals experience low intensity of several psychological symptoms, and reveal medium stigma towards mental illness. High-school teachers revealed higher psychological distress compared to higher education professors but no differences were found in stigma. The higher the psychological distress, the higher the stigma is expected to be towards people with mental illness. Therefore intervention towards mental health is needed, addressing psychological distress in order to minimize mental health stigma.

**References**

1. Au DWH, Tsang HWH, Lee JLC, Leung CHT, Lo JYT, Ngai SPC, et al. Psychosomatic and physical responses to a multicomponent stress management program among teaching professionals: A randomized study of cognitive behavioral intervention (CB) with complementary and alternative medicine (CAM) approach. Behav Res Ther. 2016;80:10–16.

2.Hamann J, Bühner M, Rüsch N. Self-Stigma and Consumer Participation in Shared Decision Making in Mental Health Services. Psychiatr Serv. 2017;68(8):783–788.

3. Basar MCZ. The beliefs of teachers toward mental illness. Procedia - Soc Behav Sci. 2012;47:1146–1152.

4. Ketchen SL, Gaddis SM, Heinze J, Beck K, Eisenberg D. Variations in Student Mental Health and Treatment Utilization Across US Colleges and Universities. J Am Coll Heal. 2015;63(6):388–396.

5.De Sousa S, Marques A, Curral R, Queirós C. Stigmatizing attitudes in relatives of people with schizophrenia: a study using the Attribution Questionnaire AQ-27. Trends Psychiatry Psychother. 2012;34(4):186-197.

**Keywords**

Mental health, Psychological symptoms, Stigma, Teaching professionals.s

### O154 Exploring quality of life in individuals with cognitive impairment and chronic mental health difficulties in the context of supported employment services

#### Ana R Jesus, Cristina Silva, João Canossa Dias, Karina Sobral, Mário Matos, Patrícia Sá, Rui Moreira, Sara Coutada, Sara A Oliveira, Telma Antunes

##### Associação para a Recuperação de Cidadãos Inadaptados da Lousã, 3200-901 Lousã, Portugal

###### **Correspondence:** Sara A Oliveira (saraoliveira.atalaia@gmail.com)

**Background**

The complex combination of many broad factors may have adverse effects in health with an impact on several areas. Quality of life (QoL) is a useful concept measuring the health state experienced by individuals. Perceived physical health, psychological well-being, social relationships, and environmental factors are QoL’s main dimensions (WHOQOL Group, 1995). In the field of employment, evidence-based supported employment is one of the most effective approaches. Thus, A.R.C.I.L’s Centre of Resources for Employment and Open Labour Market Inclusion started to include workers according to their preferences, mainly individuals with cognitive impairment and chronic mental health difficulties. In Portugal, few studies explored QoL in individuals with cognitive impairment and chronic mental health difficulties, particularly from the individual’s own perspective.

**Objective**

This study aims to explore differences in QoL between individuals with cognitive impairment and chronic mental illness, attending supported employment services given by the Centre of Resources for Employment and Open Labour Market Inclusion. Moreover, from baseline to 6 months, the outcomes from individuals self-reported QOL will be analysed.

**Methods**

The sample was composed by adults (N = 169; 52.1% women and 47.9% men), aged 18-64 years-old, with a mean age of 41.34 (SD = 11.33), who attended supported employment services. All participants had a previous diagnosis of cognitive impairment and/or chronic mental illness. QoL was assessed by WHOQOL-Bref (N=169 at baseline; N=51 at 6 months follow-up).

**Results**

Results from independent t-test revealed significant gender and age differences between individuals QoL, with men reporting better physical and psychological QoL when compared to women. Younger participants (age ≤ 40 years-old) also presented better QoL (in all domains, except in social relations) when compared to older participants. All differences reflected small to moderate effect sizes. Overall QoL, as well as its physical and environmental dimensions were significantly and negatively associated with participants’ age. Finally, after attending six-months of supported employment services 29.4% of participants increased global QoL, 35.3% increased their physical and psychological QoL, 37.3% showed an improvement in their social relationships and 55% reported feeling better in their environment.

**Conclusions**

Overall, results suggest that individuals with cognitive impairment and chronic mental illness, attending supported employment services, have perceived a positive QoL at the 6-month follow-up. As the supported employment services are still on-going, future studies would have to be conducted to explore results in a larger sample and to measure impact in people’s health and living conditions.

**Keywords**

Quality of Life, WHOQOL-Bref, Supported employment services, Cognitive impairment, Chronic mental illness.

### O155 Characterization of pediatric medicines use in pre-scholar and primary school children

#### Isabel C Pinto^1,2^, Luís M Nascimento^2,3^, Ana Pereira^4^, Ana Izidoro^5^, Cátia Patrocínio^6^, Daniela Martins^7^, Margarida Alves^8^

##### ^1^Departamento de Tecnologias de Diagnóstico e Terapêutica, Escola Superior de Saúde, Instituto Politécnico de Bragança, 5300-253 Bragança, Portugal; ^2^Centro de Investigação de Montanha, Instituto Politécnico de Bragança, 5300-253 Bragança, Portugal; ^3^Unidade Local de Saúde do Nordeste, 5300-253 Bragança, Portugal; ^4^Farmácia D'Izeda, 5300-592 Izeda, Bragança, Portugal; ^5^Farmácia Rainha, 5140-067 Carrazeda de Ansiães, Portugal; ^6^Farmácia Confiança, 5300-178 Bragança, Portugal; ^7^Farmácia Holon, 2870-225 Montijo, Portugal; ^8^Farmácia da Ponte, 5370-390 Mirandela, Portugal

###### **Correspondence:** Isabel C Pinto (isabel.pinto@ipb.pt)

**Background**

Parents or other caregivers usually resort to the use of medication without prescription in their children, which can be considered as a facilitative process of drug intoxication. The child is not an adult in small size, which necessarily has implications for the use of drugs to ensure safety and effectiveness.

**Objective**

To examine the use of paediatric medicines and of associated factors, in children of pre-school and of the 1st cycle of basic years of education in the city of Bragança, in the Northeast of Portugal.

**Methods**

This cross-sectional, descriptive and correlational study was based on a questionnaire applied to 371 parents or guardians of children of pre-school and 1st cycle of basic education in the city of Bragança, in the academic year 2014/2015. Statistical analysis was performed on the SPSS program, v. 20.0. It was used descriptive statistics; correlations were accessed using Spearman and qui-square tests, considering the significance level of 5%.

**Results**

The results revealed that 86% of parents use drugs without prescription, of these 49% resort to this practice under the influence of ancient medical guidelines and 28% under the influence of information transmitted in the pharmacy. Mostly parents (53%) resort to self-medication to relieve fever or treatment of influenza symptoms (14%) of their children. No statistically significant factors related to the use of non-prescription medication in children were found.

**Conclusions**

Paediatric self-medication is a common practice, especially made based on old medical guidelines. No explanatory factors have been found for this paediatric self-medication.

**Acknowledgements:**

The authors thank Fundação para a Ciência e a Tecnologia (FCT, Portugal) and the infrastructure FEDER under the PT2020 program for financial support to CIMO (UID/AGR/00690/2013).

**Keywords**

Children, Pediatric medicines use, Pediatric self-medication, Pre- scholar children, Primary school children.

### O156 Sleep and perimenopause: contributions to its management

#### Arminda Pinheiro (aanes@ese.uminho.pt)

##### Higher School of Education, University of Minho, 4710-228 Braga, Portugal

**Background**

There are large geographical differences in the prevalence of menopausal symptoms. Given the differences in study methodologies, it has been difficult to establish comparisons. In middle-aged women, sleep disorders are quite prevalent problems and are sometimes attributed directly to the menopausal transition. Based on the conceptual framework proposed by Meleis and Schumacher, we consider that recent changes in the sleep pattern are an indicator of the transition process, assuming that they can interfere with quality of life, and that we can try to identify the conditions/factors associated with this change.

**Objective**

To evaluate sleep disorders and related factors in perimenopausal women.

**Methods**

This was a cross-sectional study, correlational; with a non-probabilistic convenience sample, in which 600 Portuguese perimenopause women (45–55 years) were requested to complete: the Menopause Rating Scale (MRS), Scale of attitudes and beliefs about menopause and the Satisfaction Scale Social Support; Self-Esteem Scale. Semi-structured interviews collected: socio-demographic and socio-economic data; lifestyle data; psychological data - global health perception; stressful life events; perception of the recent change of body image; have life projects and data on health history. Physical examination included: blood collection for determination of follicle stimulating hormone (FSH) and estradiol (E2), weight, height, and abdominal measurements. Women signed informed consents after exhibition of the study objectives and after guaranteeing anonymity and confidentiality.

**Results**

In this study 43.5% of the women reported having no problems with sleep; 18.2% light intensity problems; 10.2% moderate intensity and 28.2% very intense problems. In relation to the influence of the different factors included in the final model on the probability of a woman having reported uncomfortable sleep problems, the logistic regression Forward: LR revealed that the conditions of socio-demographic and socioeconomic factors: level of education (b_basiceducation_=0.933, X^2^_wald(1)_=4.386, p=0.035, OR=2.222), the conditions of the psychosocial factor: attributed meaning menopause (b_positivemeaning_=-0.504;X^2^_wald(1)_=6.262; p=0.012; OR=0.604), satisfaction with social support (b_familyupport_=-0.154, X^2^_wald(1)_=10.849, p=0.001, OR=0.857), attitudes and beliefs regarding menopause(b_changeshealthaging_=-0.207;X^2^_wald(1)_=10.634, p=0.001, OR=0.813), attitudes(b_physicalchanges_=0.130, X^2^_wald(1)_=5.282; p=0.022; OR=0.878); have projects (b_donothaveprojects_=-0.662;X^2^_wald(1)_=9.907; p=0.002; OR=0.516) and the condition of the lifestyle factor: number of feed (b_numberoffeed_=- 0.285, X^2^_wald(1)_=10.658, p<0.001, OR=0.752), presented a statistically significant effect, significant difference on the Logit of the probability of a woman having referred problems, according to the adjusted Logitmodel(G^2^(10)=173.916; p<0.001;X^2^_wald(8)_=6.484; p= 0.593;R^2^_CS_=0.252;R^2^_N_=0.342;R^2^_MF_=0.218).

**Conclusions**

Problems with sleep can be considered a negative indicator of processes in perimenopausal women. The model suggests some modifiable factors, specifically: eating habits, attitudes, beliefs and meaning attributed to menopause, and importance of satisfaction with family social support. These aspects should be included in the initial nursing assessment and risk evaluation of women who cross this period, in the sense of adequately managing nursing interventions.

**Keywords**

Problems, Sleep, Menopause.

### O157 Preschooler’s executive and socio-emotional functioning: effects of two intervention programs- Psychomotor therapy and Creative Dance

#### Andreia Sarnadinha^1^, Catarina Pereira^1,2,3^, Ana C Ferreira^1,2,3^, Jorge Fernandes^1,2,3^, Guida Veiga^1,2,3^

##### ^1^Department of Sports and Health, School of Science and Technology, University of Évora, 7000-671 Évora, Portugal; ^2^Comprehensive Health Research Center, University of Évora, 7000-671 Évora, Portugal; ^3^Research Center in Sports Sciences, Health Sciences and Human Development, University of Beira Interior, 6201-001 Covilhã, Portugal

###### **Correspondence:** Andreia Sarnadinha (asarnadinha@gmail.com)

**Background**

The preschool years represent a critical time period for the development of children’s executive functioning and socio-emotional competences [1] and therefore it is the ideal period for the stimulation of these competencies [2, 3]. Interventions with children in preschool age should privilege spontaneity, creativity and play as a method of learning and stimulation [4, 5]. Psychomotor therapy and Creative Dance are two therapeutic approaches based on these principles [5, 6]. However, to date, no research has been done comparing the effects of Psychomotor therapy and Creative Dance.

**Objective**

The aim of this study was to examine the feasibility and the impact of two intervention programs, Psychomotor therapy *versus* Creative Dance, on the executive and socio-emotional functioning of pre-schoolers.

**Methods**

Fifty preschool children (M = 4.04 years; SD = 0.67) were divided into two intervention groups and a control group. An experimental group participated in 24 Psychomotor therapy sessions, mainly involving sensorimotor activities and games with rules. The other experimental group participated in 24 Creative Dance sessions. The control group maintained daily life activities. Cold executive functions, hot executive functions, externalized and internalized behaviours and aggressiveness were evaluated.

**Results**

The intervention programs were well tolerated by pre-school aged children. No significant differences were found in terms of intra- and inter-observer comparisons, except in the control group (p < 0.05). Cold executive functions were negatively correlated to reactive aggressiveness (r = -0.408, p = 0.003).

**Conclusions**

The results suggest that both programs were feasible and well tolerated in this age group, but their benefits were not evident. Increased working memory was associated with decreased levels of reactive aggression. This study alerts to the need for further research focused on pre-schoolers’ executive and socio-emotional functioning, particularly on the effects of interventions programs.

**References**

1. Papalia DE, Olds SW, Feldman, RD. O mundo da criança. Lisboa: McGraw-Hill; 2001.

2. Diamond A, Lee K. Interventions shown to aid executive function development in children 4–12 years old. Science, 2011;333(6045):959–964.

3. León CBR, Rodrigues CC, Seabra AG, Dias NM. Funções executivas e desempenho escolar em crianças de 6 a 9 anos de idade. Revista Psicopedagogia. 2013;30(92):113-120.

4. Vygotsky LS. A formação social da mente. São Paulo: Martins Fontes; 2010.

5. Traverso L, Viterbori P, Usai MC. Improving executive function in childhood: evaluation of a training intervention for 5-year-old children. Front Psychol. 2015;6:525-536.

6. Gilbert AG. Creative dance for all ages: a conceptual approach. Australia: Shape America; 2015.

**Keywords**

Executive functions, Mental health, Mind-body therapies, Psychomotor intervention.

### O158 Impact of a 10 km race on inflammatory and cardiovascular markers: comparison between trained and untrained recreational adults

#### Margarida Carvalho^1^, Andreia Noites^2^, Daniel Moreira-Gonçalves^3,4^, Rita Ferreira^5^, Fernando Ribeiro^6^

##### ^1^Hospital de Santa Maria, 4049-025 Porto, Portugal; ^2^Department of Physiotherapy, School of Allied Health Technologies, Polytechnic Institute of Porto, 4200-072 Porto, Portugal; ^3^Research Center in Physical Activity, Health and Leisure, Faculty of Sport, University of Porto, 4200-450 Porto, Portugal; ^4^Department of Surgery and Physiology, Faculty of Medicine, University of Porto, 4200-450 Porto, Portugal; ^5^Mass Spectrometry Group, Department of Chemistry, University of Aveiro, 3810-193 Aveiro, Portugal; ^6^School of Health Sciences and Institute of Biomedicine, University of Aveiro, 3810-193 Aveiro, Portugal

###### **Correspondence:** Margarida Carvalho (margarida.carvalho.ft@gmail.com)

**Background**

Previous studies have found that trained athletes had lower changes in circulating levels of inflammatory biomarkers and cardiovascular stress than untrained athletes, upon prolonged or exhausting exercise. Particularly, recreational runners with less training showed higher risk of cardiac injury and dysfunction after a marathon. Presently, we are observing a steadily growing number of young and older adults engaging in running events without having a professional orientation or training, emphasizing the need to assess biochemical markers that allow the evaluation of the acute changes imposed in these recreational athletes.

**Objective**

To compare the immediate and 24-hour effects of a 10-km run on inflammatory and cardiovascular biomarkers between recreational athletes, with and without specific running training.

**Methods**

18 recreational athletes (38.5 ± 14.5 years), 10 men and 8 women, were recruited and divided in a trained and untrained group. Venous blood samples were taken prior to the 10km race (48 hours before), immediately after (within 30 minutes), and 24 hours after the race. The following biomarkers were analysed by slot blotting assay: vascular endothelial growth factor (VEGF), interleukin 6 (IL-6), high sensitive C-reactive protein (hsCRP), ghrelin, matrix metalloproteinase-2 (MMP-2) and MMP-9.

**Results**

The trained group completed the race in 50.3 ± 13.0 minutes *per* comparison to the 66.8 ± 5.6 minutes of the untrained group (p = 0.003). A significant increase in circulating levels of hsCRP, ghrelin, VEGF and MMP-9 was observed immediately after the race in both groups; the levels of these biomarkers returned to baseline 24h post-race. A significant increase in IL-6 was also detected after the race in both groups, which returned to baseline levels at 24 hours post-race in the untrained group. Regarding MMP-2 levels, a significant increase was detected after the race only in the untrained that returned to baseline levels at 24 hours post-race.

**Conclusions**

The impact of a 10-km race in the inflammatory and cardiovascular markers assessed in this study was different between recreational athletes, with and without specific training.

**Keywords**

Biomarkers, Cardiovascular system, Exercise, Inflammation, Running.

### O159 The health of the informal caregiver of dependent person in self-care

#### Maria A Dixe^1,2^, Ana CS Cabecinhas^2^, Maura R Domingues^2^, Ana JCF Santos^2^, Marina G Silva^2^, Ana Querido^1,2^

##### ^1^Center for Innovative Care and Health Technology, Polytechnic Institute of Leiria, 2411-901 Leiria, Portugal; ^2^School of Health Sciences, Polytechnic Institute of Leiria, 2411-901 Leiria, Portugal

###### **Correspondence:** Maria A Dixe (ana.querido@ipleiria.pt)

**Background**

Caring for a caregiver should be a constant concern and a responsibility of all health professionals, so that those who give care do not end up being uncared-for.

**Objective**

This correlational study had the following main aims: to assess the level and prevalence of burden of the informal caregiver of a person dependent in self-care; to determine the relationship between the levels of burden and the informal caregiver's perception of their level of competence to be a caregiver.

**Methods**

Participants in this study were 33 informal caregivers of self-care dependent-persons in at least one activity of daily living, to whom a structured interview was performed at the time of hospital discharge. The interview included socio-demographic and professional data, perception of the informal caregiver on their level of competence to be a caregiver, and the Portuguese version of the Zarit Burden Interview [1]. This study was approved by the National Data Protection Commission and the ethics committee of the hospital where the study was conducted (nº 24/2017).

**Results**

The majority of dependent-persons were female (60.6%) with a mean age of 81.6 ± 11.3 years old, with the majority being dependent on all self-care activities. The mean age of caregivers was 61.4 ± 12.1 years old, mainly females. The family relationship was mostly a son/daughter (39.4%) or a spouse (33.3%), taking care of the patient on average at 63.9 ± .93 months. It was possible to verify that all caregivers had previous experience of caregiving to a family dependent. We also verified that the 33 caregivers presented a mean of 53.9 ± 15.8 on the emotional burden scale (maximum possible value of 110) which corresponds to little burden. We can also mention that 30.3% of the caregivers present no burden, 30.3%, present mild burden and 39.4% present intense burden. Regarding the relationship between caregiver burden, we verified that higher levels of informal caregivers burden were related to lower levels of perception of their competence to satisfy the needs related to the hygiene of the dependent-person (rs=.-0.514; p> 0.05).

**Conclusions**

Caring of a dependent-person may lead to health risks to the caregiver. Even though the sample size is small, it was possible to verify that a considerable number of caregivers presented intense emotional burden. Therefore, it is necessary that health professionals develop interventions to prevent caregiver burden.

**Acknowledgments**

The current abstract is being presented on the behalf of the Help2Care research project. This study was funded by COMPETE 2020 under the Scientific and Technological Research Support System, in co-promotion. We acknowledge CiTechCare, Polytechnic Institute of Leiria, Polytechnic Institute of Santarém, Centro Hospitalar de Leiria, Polytechnic Institute of Castelo Branco and also all other members, institutions and students involved in the project.

**References**

1. Sequeira CA. Adaptação e validação da Escala de Sobrecarga do Cuidador de Zarit. Revista Referência. 2010;2(12):9-16.

**Keywords**

Emotional burden, Informal caregiver, Self-care, Dependent-person, help2care.

### O160 Growth and puberty in adolescent artistic gymnasts: is energy intake a question of concern?

#### Rita Giro^1^, Mónica Sousa^2^, Inês T Marques^3^, Carla Rêgo^3^

##### ^1^Universidade do Porto, 4099-002 Porto, Portugal; ^2^Escola Superior de Saúde, Instituto Politécnico de Leiria, 2411-901 Leiria, Portugal; ^3^Centro da Criança e do Adolescente, Hospital CUF Porto, 4100-180 Porto, Portugal

###### **Correspondence:** Rita Giro (aritagiro@gmail.com)

**Background**

Whether high-intensity training during childhood and adolescence compromises the growth and pubertal development of artistic gymnasts, remains a question for debate. However, when coupled with low energy availability, this hypothesis strengthens [1].

**Objective**

To characterize growth, sexual maturation, total energy intake and training aspects of competing artistic gymnasts and check for associations.

**Methods**

Convenience sample of 22 competing artistic gymnasts (13.8 ± 1.9 years) of both sexes. Anthropometric evaluation and body composition assessment were performed (InBody230™). Tanner stage was determined in all athletes and age of menarche and regularity of menses were assessed in females. Total energy intake was quantified (3-day food record) and its inadequacy verified according to the recommendations [2]. Athletes' training habits were also characterized.

**Results**

Females showed significantly higher body fat (15.9 ± 3.2 vs 7.2 ± 3.6) and lower skeletal muscle mass (45.7 ± 2.1 vs 51.1 ± 3.0) percentages than males (p < 0.05). No differences were found between genders for any of the additional variables in the study (p ≥ 0.05). Mean age of menarche was 12.6 (± 1.3) years. Short stature was detected in 12.6% of the female gymnasts (z score < -2), but no cases of low weight-for-height (z score <-2) were observed. All athletes presented total energy intakes below the recommendations. A high training frequency and intensity (median: 6 days/week, in a total of 20.8 hours) were reported, as well as an association of both training frequency (ρ=+0.765; p=0,016) and training intensity (ρ=+0.727; p=0,026) with a later onset of menarche.

**Conclusions**

Our data suggests metabolic adaptation to chronically insufficient energy intakes in these athletes which, in accordance with growing evidence, might play a role in growth and pubertal delay.

**Acknowledgements**

Authors would like to thank and recognise the contribution of Cristina Côrte-Real and Manuel Campos to the development of the investigation.

**References**

1. Mountjoy M, Sundgot-Borgen J, Burke L, Carter S, Constantini N, Lebrun C, et al. The IOC consensus statement: beyond the Female Athlete Triad--Relative Energy Deficiency in Sport (RED-S). Br J Sports Med. 2014;48(7):491-497.

2. EFSA Panel on Dietetic Products Nutrition and Allergies (NDA). Scientific Opinion on Dietary Reference Values for energy. EFSA Journal. 2013;11(1):3005.

**Keywords**

Athletes, Energy availability, Health, Nutrition assessment, Paediatrics.

### O161 Communication effectiveness in nursing teams

#### António Calha^1^, Liliana Grade^3^, Olívia Engenheiro^4^, Sandra Sapatinha^5^, Eva Neto^2^

##### ^1^Instituto Politécnico de Portalegre, 7300-110 Portalegre, Portugal; ^2^Centro Hospitalar do Algarve, 8000-386 Faro, Portugal; ^3^Unidade Local de Saúde Baixo Alentejo, 7801-849 Beja, Portugal; ^4^Hospital Espírito Santo, 7000-811 Évora, Portugal; ^5^Unidade Local de Saúde Norte Alentejano, 7300-074 Portalegre, Portugal

###### **Correspondence:** António Calha (antoniocalha@hotmail.com)

**Background**

The communication processes established among nurses are influential factors of the quality and effectiveness of care.

**Objective**

This research had as main objective to identify how nurses evaluate the different dimensions of the communicative process of the service in which they carry out their professional activity and what are the main obstacles to the proper functioning of the communication process.

**Methods**

This is eminently a quantitative study of correlational nature. For the collection of data, a questionnaire was used with closed-ended questions. 75 nurses from four health services (Neonatology, Medicine II, Emergency and Basic Emergency) were surveyed.

**Results**

Five indices were elaborated to measure the different facets of the communicative process: Communication efficiency (α = 0.82); Information sufficiency (α = 0.84); Information timing (α = 0.85); Explicitness of the message (α = 0.90) and practical applicability (α = 0.087). Similarly, five indices were computed in order to measure the nature of the information: Information of a clinical nature (α = 0.88); Organizational information (α = 0.89); Service information (α = 0.91); Team information (α = 0.92) and Personal information (α = 0.93). All indices had a variation range between 1, corresponding to the worst possible appraisal and 5, corresponding to the best possible appraisal. The results show that it is organizational information that nurses (M = 2.96) do worse in the communicative process, especially in the timely manner in which information arrives to them (M = 2.76). The results obtained through a Kruskal-Wallis test allowed to identify differences regarding the consideration of conflict as an obstacle to the communication process (χ^2^_KW(3)_= 30.01, p= 0.000) and overvaluation of personal and professional relations (χ^2^_KW(3)_= 12.60, p= 0.006).

**Conclusions**

The research identifies some communication weaknesses in the clinical context related mainly to the way information of organizational nature is disseminated in the services.

**Keywords**

Communication, Efficiency, Nursing, Teams.

### O162 Factors affecting interpersonal conflict in nursing teams

#### António Calha^1^, Marília Ferreira^2^, Sílvia Alminhas^2^, Telmo Pequito^2^

##### ^1^Instituto Politécnico de Portalegre, 7300-110 Portalegre, Portugal; ^2^Hospital Espírito Santo, 7000-811 Évora, Portugal

###### **Correspondence:** António Calha (antoniocalha@hotmail.com)

**Background**

Teamwork is one of the foundations of nursing, exposing the profession to the vulnerabilities of the dynamics of group functioning. In this context, skills of conflict management in working teams are particularly relevant.

**Objective**

This research aimed to: I) identify how often nurses deal with conflict situations; II) identify the main causes of conflict mentioned by nurses and, III) assess the strategies adopted to deal with conflicting situations.

**Methods**

This is an exploratory, quantitative and correlational research. For the collection of data, a questionnaire was used with closed-ended questions. The sample consisted of a total of 35 nurses from the emergency department of a Hospital of the Portuguese NHS.

**Results**

Five indexes were computed in order to evaluate the different strategies to deal with conflict situations: I) commitment strategy (α = 0.745); II) avoidance strategy (α = 0.699); III) accommodation strategy (α = 0.745); IV) confrontation strategy (α = 0.618) and collaboration strategy (α= 0.698). All indices had a variation that ranged between 1, corresponding to the less frequent possible appraisal and 5, corresponding to most frequent possible appraisal. Most of the nurses reported that they were rarely involved in situations of conflict, however, 57.1% stated that they sometimes observed those situations. Results show that nurses mostly indicated the use of two strategies to deal with conflict: accommodation (M= 3.11) and confrontation (M= 3.07). Data analysis revealed that the strategy of accommodation had a statistically significant positive correlation coefficient in relation with the incompatibility of personalities (rs= 0.400, p < 0.05) and a negative correlation coefficient in relation with the scarcity of material (rs= -0.358, p < 0.05) as cause of conflict.

**Conclusions**

The results obtained allow us to conclude that the nature of the conflict determines the way it is managed by nurses. The data reveal, in particular, that the scarcity of material resources strengthens the confrontation in the nursing team, contributing to the degradation of the organizational environment. Thus, conflict management is an essential skill and tool that nurses can, and should, use as a basis of sustainability and development of nursing practice.

**Keywords**

Interpersonal conflict, Team work, Nursing, Emergency service.

### O163 Numerical modelling of electrical stimulation on scaffolds for tissue engineering

#### Paula Pascoal-Faria^1,2^, Pedro C Ferreira^1^, Abhishek Datta^3,4^, Nuno Alves^1^

##### ^1^Centre for Rapid and Sustainable Product Development, Polytechnic Institute of Leiria, 2411-091 Leiria, Portugal; ^2^School of Technology and Management, Polytechnic Institute of Leiria, 2411-901 Leiria, Portugal; ^3^Soterix Medical Inc., 10001 New York, New York, United States of America; ^4^City City College of New York, 10031 New York, New York, United States of America

###### **Correspondence:** Paula Pascoal-Faria (paula.faria@ipleiria.pt)

Preliminary experimental *in vitro* studies on tissue engineering applications have shown the advantage of using different type of stimuli, namely, mechanical, electrical, magnetic or its combination to enhance cell behaviour. In these studies cells proliferation and differentiation change significantly when electrical stimulation is applied on cells placed inside scaffolds systems within a bioreactor. We established an ambitious research program on numerical modelling of stimuli on scaffolds for tissue engineering. In this study we develop a new finite element-based (FE) multiphysics framework that allows the numerical optimization of the parameters involved when electrical stimulation is applied on bioscaffolds with different geometries and characteristics. The FE framework that has been developed allows the prediction of the electrical stimulation as a function of the scaffold geometry and its electrical characteristics, that may contribute to the acceleration of the proliferation and differentiation of the cells.

**Keywords**

Finite element model, Bioscaffolds, Electrical stimulation, Tissue engineering.

### O164 Motor competence in preschoolers with and without hearing loss

#### Guida Veiga^1,2,3^, Mariana Santos^1^, Brenda S Silva^4^, Catarina Pereira^1,2,3^

##### ^1^Department of Sports and Health, School of Science and Technology, University of Évora, 7000-671 Évora, Portugal; ^2^Comprehensive Health Research Center, University of Évora, 7000-671 Évora, Portugal; ^3^Research Center in Sports Sciences, Health Sciences and Human Development, University of Beira Interior, 6201-001 Covilhã, Portugal; ^4^Developmental Psychology, Leiden University, 2311 EZ Leiden, Netherlands

###### **Correspondence:** Guida Veiga (gveiga@uevora.pt)

**Background**

Several studies have been demonstrating the developmental consequences of early childhood hearing loss in terms of language, communication, academic, and social-emotional functioning [1]. However, there is still no consensus on whether young children with hearing losses (HL) are comparable to hearing peers regarding their motor competence. Whereas some studies associate hearing loss with poorer motor development [2], others show that HI children are as proficient as their hearing peers [3]. Nevertheless, most of these studies has been focusing older children and to date, no study has examined Portuguese HI children’s motor competence.

**Objective**

This study aimed to examine motor competence of children with HL in comparison with hearing children.

**Methods**

A total of 35 children participated in the study; 13 (mean age 4,73 years) with HL and 22 (mean age 5,09) hearing children. Children were tested by the Movement Assessment Battery for Children–Second Edition (MABC-2).

**Results**

Children with HL showed worse performances on manual dexterity, ball skills and balance than hearing peers, however these differences were only significant regarding balance (p=.006).

**Conclusion**

Children with HL are at greater risk for balance deficits.

**References**

1. Joint Committee on Infant Hearing. 2007 position statement: Principles and guidelines for early hearing detection and intervention programs. Pediatrics. 2007;120:898–921.

2. Hartman E, Houwen S, Visscher C. Motor skill performance and sports participation in deaf elementary school children. Adapted Physical Activity Quarterly. 2011;28(2):132-145.

3. Engel-Yeger B, Weissman D. A comparison of motor abilities and perceived selfefficacy between children with hearing impairments and normal hearing children. Disability And Rehabilitation. 2009;31(5):352-358.

**Keywords**

Motor skills, Emotion understanding, Empathy, Deafness, Cochlear implant.

### O165 Determinants of the beginning of breastfeeding in the first half hour of life

#### Dolores Sardo (dolores.sardo@gmail.com)

##### Nursing School of Porto, 4200-072 Porto, Portugal

**Background**

Breastfeeding in the first hour of life is associated with longer duration of breastfeeding, reduction of infant deaths in low-resource countries and improvement of the health status of the child and of mother. This practice favours early contact between mother and baby, and in Step 4 of the Ten Measures to be considered a Baby Friendly Hospital, it is recommended to help mothers to initiate breastfeeding within the first half hour after birth (WHO & UNICEF).

**Objective**

To evaluate the rate of breastfeeding initiation in the first half hour and two hours of the child's life; identify the determinants of breastfeeding in the first half-hour and two hours of life and their relationship to the prevalence of exclusive breastfeeding at four months.

**Methods**

We performed a quantitative, descriptive and cross-sectional study in a Portuguese population. The sample was no probabilistic, intentional (n = 150) 89.3% married mothers or with partner and average age was 31.1 years old, with normal delivery (43.3%). Data collection was conducted through a self-report questionnaire to mothers, 4 months after delivery.

**Results**

The rate of breastfeeding initiation in the first half hour and two hours of the child's life was 48% and 88% respectively. In this sample the determinants related to obstetric history, new-born and breastfeeding were studied: only caesarean delivery revealed statistical significance in the first half hour (X^2^(1) = 6.141; p = 0.010); and there was a statistically significant difference associated with eutocic delivery and the initiation of breastfeeding up to two hours of life. The prevalence of exclusive breastfeeding at 4 months was predicted only by the weight of the new-born (b_pequeno para a idade gestacional_=4.821; X^2^_wald(1)_= 21.616; p < 0.001; OR=124.038).

**Conclusions**

In this study, only the type of delivery had significant statistical influence for the initiation of breastfeeding in the first half hour of life. We also found that the initiation of breastfeeding in the first two hours of life did not predict its prevalence at four months.

**Keywords**

Breastfeeding, Prevalence, First half-hour.

### O166 Body image acceptance as a protector against the effect of body image-related shame memories on emotional eating in women with Binge Eating Disorder

#### Cristiana Duarte^1^, José Pinto-Gouveia^2^

##### ^1^School of Psychology, Faculty of Medicine and Health, University of Leeds, LS2 9JT, Leeds, United Kingdom; ^2^Cognitive and Behavioural Centre for Research and Intervention, University of Coimbra, 3001-802 Coimbra, Portugal

###### **Correspondence:** Cristiana Duarte (c.duarte@leeds.ac.uk)

**Background**

Understanding the factors underlying the public health problem of Binge Eating Disorder (BED) is a pressing need. Research show that body image-related shame memories are significantly associated with the severity of binge eating symptomatology in women with Binge Eating Disorder (BED) [1]. These individuals may eat as a means to temporarily avoid or reduce negative emotions related to body weight and shape [2, 3]. Evidence suggests that body image flexibility (*i.e.*, the ability to accept and fully experience body image-related internal experiences - thoughts, emotions, memories - when doing so is consistent with valued-living [4], may deter the engagement in emotional eating [5].

**Objective**

The current study examined the moderator effect of body image flexibility on the association between shame memories and emotional eating in a sample of women with BED.

**Methods**

109 women with the diagnosis of BED participated in this study. Mean (SD) participants age was 37.39 (10.51) years and BMI was 33.69 (7.75) kg/m^2^. Participants were assessed through the Eating Disorder Examination 17.0D, the Shame Experiences Interview (SEI) and filled self-report measures assessing the centrality of the recalled shame memories, emotional eating and body image flexibility.

**Results**

Descriptive statistics showed that body image-related shame memories were the most frequently recalled memories. Correlational analysis revealed that the extent to which the recalled shame memory is central to identity is significantly associated with emotional eating. Body image flexibility was negatively associated with the centrality of the recalled shame memory and emotional eating. Results of the moderation analysis showed that body image flexibility significantly moderate the association between the centrality of the recalled shame memory and emotional eating. This suggests that patients with BED with a greater trait-like ability to accept body image-related negative internal experiences, present a lower tendency to use food as a form of experiential avoidance.

**Conclusions**

Findings support a conceptual integrative model of binge eating symptomatology that clarifies the role that contextual and interpersonal variables play in the development and persistence of difficulties in regulating eating behaviour in BED, and how body image flexibility may have a protective role in these associations. These results also have important clinical implications supporting the relevance of contextual-behavioural interventions that address the role of shame memories, body image difficulties and that help patients to develop body image flexibility.

**References**

1. Duarte C, Pinto-Gouveia J. The impact of early shame memories in Binge Eating Disorder: The mediator effect of current body image and cognitive fusion. Psychiatry Reseach, 258:511- 517.

2. Duarte C, Pinto-Gouveia J, Ferreira C. Ashamed and fused with body image and eating: Binge eating as an avoidance strategy. Clin Psychol Psychother. 2015. doi: 10.1002/cpp.1996. 3. Heatherton T, Baumeister R. Binge eating as escape from self-awareness. Psychol Bull 1991;110(1):86-108.

4. Sandoz E, Wilson K, Merwin R, Kellum K. Assessment of body image flexibility: The Body Image-Acceptance and Action Questionnaire. Journal of Contextual Behavioral Science. 2013;2(1-2):39-48.

5. Duarte C, Pinto-Gouveia J. Returning to emotional eating: The psychometric properties of the EES and association with body image flexibility. Eating and Weight Disorders. 2015;20 (4):497-504.

**Keywords**

Body image-related shame memories, Shame memories centrality, emotional eating, Body image flexibility, Binge Eating Disorder.

### O167 Psychometric properties of the Psychological Welfare Scale

#### Rosa M Freire, Filipe Pereira, Teresa Martins, Maria R Grilo

##### Nursing School of Porto, 4200-072 Porto, Portugal

###### **Correspondence:** Rosa M Freire (rosafreire@esenf.pt)

**Background**

Pleasure and happiness have been associated with well-being, and obtaining maximum enjoyment is seen as a life goal. Nowadays, well-being is perceived to be either subjective wellbeing (SWB) or psychological well-being (PWB). SWB involves global evaluations that affect life quality, whereas PWB examines perceived existential challenges of life [1].

**Objective**

To measure Psychological Welfare and validate the constructs of 42 items of the Psychological Welfare Scale.

**Methods**

Quantitative, exploratory and cross study was used as methodology. A convenience sample was performed with 252 participants. The measuring instrument was developed using the Qualtrics program. The study had favourable appreciation and consent from the College Nursing of Porto (ESEP) that provided a computer platform that allowed the participants to access to the measurement instrument. The construct in analysis in this study was developed by Ryff [2] and intended to measure Psychological Welfare on six scales: autonomy, environmental mastery, personal growth, positive relations with others, purpose in life, and self-acceptance. Originally, each subscale was composed of 20 items, but the author suggested smaller versions, preferably the version of seven items per subscale. Respondents rated statements on a scale of 1 to 6, with 1 indicating strong disagreement and 6 indicating strong agreement. Participants are, on average, 31.38 years old (SD=12.88) and 81% are female. The analysis of the items of the scale was performed through item descriptive analysis, confirmatory factorial analysis, internal consistency analysis of each subscale and factorial validity using AMOS (version 22, IBM SPSS).

**Results**

The descriptive analysis of the items showed that the response tendency is above the midpoint. In the analysis of the adaptation of the theoretical model to the empirical model, we verified that the model constituted by a factor with six manifested variables resulting from the sum of the seven items of each subscale showed good indexes of adjustment. The internal consistency values of each subscale ranged from 0.69 to 0.82 suggesting a reasonable to good internal consistency. All correlations showed significant values at the 0.01 level.

**Conclusions**

The values found suggest that the concepts of the subscales of the measuring instrument are related but without any redundancy of measurements. The Psychological Welfare Scale is a theoretically grounded instrument which specifically focuses on measuring multiple facets of psychological welfare. We consider this Scale of Psychological Welfare to be a valid and reliable tool to measure psychological welfare in a Portuguese context.

**References**

1. Sweta SM. Cross-cultural Validity of Ryff’s Well-being Scale in India. Asia-Pacific Journal of Management Research and Innovation. 2013;9(4):379–387.

2. Ryff C. Happiness is everything, or is it? Explorations on the meaning of psychological wellbeing. American Psychological Association, Inc. Journal of Personality and Social Psychology. 1989;57(6):1069–1081.

**Keywords**

Assessment, Psychological Welfare, Scale, Psychometrics properties, Validation Studies.

### O168 The influence of statins in the skeletal muscle of Individuals with hypercholesterolemia - ultrasound study

#### Alexandra André^1^, João P Figueiredo^1^, Carlos AF Ribeiro^2^, Gustavo F Ribeiro^3^, Paula Tavares^3^

##### ^1^Escola Superior de Tecnologia da Saúde de Coimbra, Instituto Politécnico de Coimbra, 3046-854 Coimbra, Portugal; ^2^Instituto Biomédico de Investigação da Luz e da Imagem, 3000-548 Coimbra, Portugal; ^3^Faculdade de Ciências do Desporto e Educação Física, Universidade de Coimbra, 3040-248 Coimbra, Portugal

###### **Correspondence:** Alexandra André (alexandra.andre@estescoimbra.pt)

**Background**

The skeletal muscle (SM) is a kind of tissue that has the capacity to adjust with different stimulus. Intake drugs can cause alterations and incapacity to do exercise. To evaluate the muscle macroscopically measurements is important to do. Pennate angle and fascicle length are determinants of muscle strength. Thickness is important to evaluate state of atrophy or hypertrophy. The ecogenicithy evaluates the fat infiltration in atrophy.

Statins interfere with the SM functions and are the prescribed medications for prevention and treatment of high cholesterol more used in the world. A side effect of these drugs is myotoxicity. Creatine Kinase (CK) is a biomarker to evaluate the severity of muscle damage that can change with gender, age, and physical activity. Factors that influence the increase of CK are exercise intensity, if it exceeds the metabolic capacity alterations that origin CK release to the blood circulation and sarcomere degradation.

**Objective**

The aim of this study was to evaluate and analyse macroscopically muscle structures in patients with hypercholesterolemia, in order to interpret the pathophysiological mechanisms produced by statins inducing myotoxicity. The sensitivity and acuity to the ultrasound were used to evaluate the muscle.

**Methods**

The study was performed in 47 individuals, age between [50-65] years old. Ultrasound tests were performed to the 47 individuals in three different groups – control group, individuals that intake statins and individuals that intake statins and do exercise. Gastrocnemius muscle was performed bilaterally using a 13Mhz probe to determinate the dimensions of the pennate angle, fascicle length and thickness. A questionnaire and the informed consent term were signed by all individuals. The study was approved by the Ethics Committee for Health of the Regional Health Administration (Study nº45-2015)

**Results**

Differences in dimensions were observed. Significant differences were obtained for the pennate angle and the fascicle. The main differences were observed between the control group and experimental groups that intake statins.

**Conclusions**

Ultrasound has sensitivity and specificity to analyse the muscle tissue macroscopically. Individuals who intake statins have lower values of the evaluated structures. Differences were observed in the pennate angle in both sides, in the group that intake statins. The literature is unanimous in stating that individuals who take statins develop muscle changes called myalgia. The side effects of statins may increase with exercise. Ultrasonography is an imaging modality not yet widely used in this type of approach.

**Keywords**

Muscle, Ultrasound, Statins, Hypercholesterolemia.

### O169 Maximum expiratory pressure (MEP) Increase through a program with a sportive blowgun in institutionalized women with intellectual disability

#### Marisa Barroso^1,2^, Rui Forte^2^, Rui Matos^1,2^, Luís Coelho^1,2^, David Catela^1^

##### ^1^Life Quality Research Center, 2001-904 Santarém, Portugal; ^2^School of Education and Social Sciences, Polytechnic Institute of Leiria, 2411-901 Leiria, Portugal

###### **Correspondence:** Marisa Barroso (marisa.barroso@ipleiria.pt)

**Background**

Blowgun is a long tube through which projectiles are shot [1]. The propulsive power is limited by the user's strength of respiratory muscles and the vital capacity of the lungs [2]. People with intellectual disability have limited levels of maximum oxygen consumption and ventilatory capacity [3].

**Objective**

The aim of this study is to investigate the effect of a Sportive Blowgun program on the respiratory capacity of institutionalized women with intellectual disability.

**Methods**

The sample was divided into two groups, the control group (n = 9; age 49.80 ± 8.50), and an experimental group (n = 9; age 44.80 ± 13.30). The experimental group underwent a 12-week Sportive Blowgun program, with 2 weekly sessions where each subject threw 40 darts at a distance that progressed from 4m to 10m away from the target. Maximum expiratory pressure (MEP) values (L/min) were collected through a Micro Medical - Micro RPM portable spirometer. Five measurements were taken for both groups. One before the beginning of the program, 3 during the program 1 at the end of the program. Informed permission from the institution and the participants assent was obtained.

**Results**

In the experimental group, a significant difference (p = 0.008) was observed in the mean values of initial maximum expiratory pressure (MEP1 = 48.11 L/min) and final (MEP5 = 73.56 L/min). There is a significant difference between the values of the initial measurement with all other measurements, suggesting a positive evolution between them. There is only no significant difference between the intermediate measurements, which suggests a stagnation of the values between the second, third and fourth measurements. In the control group, except for the second measurement, there was no significant difference between the initial maximal expiratory pressure and the other measurements, which suggests that there was an initial adaptation to the test but that did not last. Comparing groups, except for the first measurement, all the others present a significant difference. A very significant difference was observed in the third (p = 0.005) and final measurement (p = 0.005), where the experimental group presented values always higher than the control group.

**Conclusions**

The results suggest that the Sportive Blowgun program increases maximum expiratory pressure (MEP) by providing a respiratory capacity gain in institutionalized women with mental disability. Is suggested a more extensive study to verify the possibility of considering the sportive blowgun program as a complementary non-clinical therapy for respiratory insufficiencies.

**References**

1. Mariñas AP, Higuchi H. Blowgun Techniques: The Definitive Guide to Modern and Traditional Blowgun Techniques. Vermont: Tutle Publishing; 2010.

2. Nagasaki T, Okada H, Kai S, Takahashi S. Influence of Blowgun Training on Respiratory Function. Rigakuryoho Kagaku 2010; 25(6): 867–871.

3. McGrother CW, Marshall B. Recent trends in incidence, morbidity and survival in Down’s syndrome. Journal of Mental Deficiency Research. 1990;34:49–57.

**Keywords**

Blowgun, Intellectual disability, Respiratory therapy.

### O170 Respiratory control technique and attention deficit hyperactivity disorder in children

#### David Catela^1^, Isabel Piscalho^2^, Rita Ferreira^2^, Ana Victorino^2^, Bárbara Cerejeira^2^, Nicole Marques^2^, Sara Dias^2^

##### ^1^Life Quality Research Center, 2001-904 Santarém, Portugal; ^2^Education School, Polytechnic Institute of Santarém, 2001-904 Santarém, Portugal

###### **Correspondence:** David Catela (catela@esdrm.ipsantarem.pt)

**Background**

Attention deficit hyperactivity disorder (ADHD) comprises a persistent pattern of symptoms hyperactivity, impulsiveness and/or lack of attention [1] and can cause a significant impairment in academic activities [2]. ADHD has a prevalence rate ranging from 3% to 7% among school age children [3]. Respiratory sinus arrhythmia (RSA) is higher among children with typical development than in children with ADHD [4], and ADHD in childhood is associated with abnormal parasympathetic mechanisms [5], with significantly higher mean heart rates, mean R-R interval significantly shorter (lower heart rate variability) and ratio LF/HF significantly higher than children with typical development [6-10].

**Objective**

The purpose of this study is to verify if through breath control ADHD children can increase heart rate variability (HRV).

**Methods**

Vital signs of ten potential ADHD children (11.22 ± .42 years old, 3 girls), were collected for 6 minutes, in supine position, under two conditions: I) normal breathing (NB); and, II) slow abdominal breathing (AB), e.g., [11]. HRV data acquisition was carried out through Polar V800 [12]. HRV analysis was made through gHRV software [13-15].

**Results**

During AB children significantly reduced breathing frequency (BF) (8.9±4.2, Md=8) compared to NB frequency (18.3±5.9, Md=19) (Z= 6.439, p<.001, r=.81); and diastolic pressure (DP) (AB – 61.3±8.7, Md=61; NB – 63.6±6.4, Md=63; Z= 2.146, p<.05, r=.29); significantly augmented standard deviation of HR (AB – 7.4±1.4; NB – 5.9±1.5; Z= 2.310, p<.05, r=.89); standard deviation of mean interval RR (AB – 80.5±22.9ms; NB – 64.3±27.7ms; Z= 2.192, p<.05, r=.73), and SD2 (AB – 106.9±29.7; NB – 82.5±35.6; Z= 2.192, p<.05, r=.73). Also, during AB children reduced systolic pressure (101.3±16.4, Md=97; NB – 104.9±17.1, Md=103; ns), heart rate frequency (79.8±11.7bpm, Md=81.8; NB – 81.7±11.8bpm, Md=83.9; ns); and, mean interval RR was greater (766.9±117.8ms Md=734.8; NB – 749.1±115.2ms, Md=715.8; ns); like rMSSD (112.9±53.3ms, Md=108.1; NB – 97.5±64.1ms, Md=79.9; ns), and HRV index (18.3±4.7ms, Md=18.9; NB – 15.6±6.1ms, Md=14.1; ns); and, ApEn became positive (greater) (.0001±.001ms, Md=.0001; NB - (-).001±.002ms, Md=(-) .0001; ns).

**Results**

Consequently, with one short training session, these children had the capacity to implement a significantly slower BF to values near 8 bpm, cf. [11], which resulted in a reduction in HR, SP, and DP; and, in an augmentation of various parameters of HRV. All these changes point to an increase of vagal activity during AB. If vagal activity was reinforced during AB [16-17], it means that, maybe, a process of bottom-up adjustment of attention and emotional responses can be promoted, cf. [18-20].

**References**

1. American Psychiatric Association. Diagnostic and statistical manual of mental disorders (DSM-5®). American Psychiatric Pub; 2013.

2. Cantwell DP, Baker L. Association between attention deficit-hyperactivity disorder and learning disorders. J Learn Disabil. 1991;24(2):88-95.

3. Rash JA, Aguirre-Camacho A. Attention-deficit hyperactivity disorder and cardiac vagal control: a systematic

review. ADHD Attention Deficit and Hyperactivity Disorders. 2012;4(4):167-77.

4. Buchhorn R, Conzelmann A, Willaschek C, Störk D, Taurines R, Renner TJ. Heart rate variability and methylphenidate in children with ADHD. ADHD Attention Deficit and Hyperactivity Disorders. 2012;4(2):85-91.

5. Musser ED, Backs RW, Schmitt CF, Ablow JC, Measelle JR, Nigg JT. Emotion regulation via the autonomic nervous system in children with attention-deficit/hyperactivity disorder (ADHD). J Abnorm Child Psychol. 2011; 39(6):841-852.

6. Tonhajzerova I, Ondrejka I, Adamik P, Hruby R, Javorka M, Trunkvalterova Z, et al. Changes in the cardiac autonomic regulation in children with attention deficit hyperactivity disorder (ADHD). Indian Journal of Medical Research. 2009;130:44-150.

7. Griffiths KR, Quintana DS, Hermens DF, Spooner C, Tsang TW, Clarke S, et al. Sustained attention and heart rate variability in children and adolescents with ADHD. Biological psychology. 2017;124:11-20.

8. Imeraj L, Antrop I, Roeyers H, Deschepper E, Bal S, Deboutte D. Diurnal variations in arousal: a naturalistic heart rate study in children with ADHD. European child & adolescent psychiatry. 2011;20(8):381-392.

9. Rukmani MR, Seshadri SP, Thennarasu K, Raju TR, Sathyaprabha TN. Heart rate variability in children with attention-deficit/hyperactivity disorder: a pilot study. Annals of neurosciences. 2016;23(2):81-88.

10. de Carvalho TD, Wajnsztejn R, de Abreu LC, Vanderlei LCM, Godoy MF, Adami F, et al. Analysis of cardiac autonomic modulation of children with attention deficit hyperactivity disorder. Neuropsychiatric disease and treatment. 2014;10:613.

11. Lehrer PM, Vaschillo E, Vaschillo B. Resonant frequency biofeedback training to increase cardiac variability: Rationale and manual for training. Applied psychophysiology and biofeedback. 2000;25(3):177-191.

12. Giles D, Draper N, Neil W. Validity of the Polar V800 heart rate monitor to measure RR intervals at rest. European Journal of Applied Physiology. 2016, 116(3):563–571.

13. Rodríguez-Liñares L, Lado MJ, Vila XA, Méndez AJ, Cuesta, P. gHRV: Heart rate variability analysis made easy. Computer Methods and Programs in Biomedicine. 2014;116(1):26–38.

14. Rodríguez-Liñares L, Méndez AJ, Vila XA, Lado MJ. gHRV: A user friendly application for HRV analysis. In: Information Systems and Technologies (CISTI); 2012. p. 1–5.

15. Vila J, Palacios F, Presedo J, Fernández-Delgado M, Felix P, Barro S. Time-frequency analysis of heartrate variability. IEEE Engineering in Medicine and Biology Magazine. 1997;16(5):119–126.

16. Levy MN. Autonomic interactions in cardiac control. Annals of the New York Academy of Sciences. 1990;601(1):209-221.

17. Uijtdehaage SH, Thayer JF. Accentuated antagonism in the control of human heart rate. Clinical Autonomic Research. 2000;10(3):107-110.

18. Ruiz‐Padial E, Sollers JJ, Vila J., Thayer, JF. The rhythm of the heart in the blink of an eye: Emotion modulated startle magnitude covaries with heart rate variability. Psychophysiology. 2003;40(2):306-313.

19. Thayer JF, Brosschot JF. Psychosomatics and psychopathology: looking up and down from the brain. Psychoneuroendocrinology. 2005;30(10):1050-1058.

20. Thayer JF, Lane RD. Claude Bernard and the heart–brain connection: Further elaboration of a model of neurovisceral integration. Neuroscience & Biobehavioral Reviews. 2009;33(2):81-88.

**Keywords**

ADHD, Children, HRV, Breathing Technique.

### O171 Can older adults accurately perceive affordances for a stepping forward task? Differences between faller and non-faller community-dwelling older adults

#### Gabriela Almeida, Jorge Bravo, Hugo Rosado, Catarina Pereira

##### Department of Sports and Health, School of Science and Technology, University of Évora, 7000-671 Évora, Portugal

###### **Correspondence:** Gabriela Almeida (gsna@uevora.pt)

**Background**

Different studies framing an ecological approach to perception [1] have tried to understand how people, mostly children [2] and adults [3], perceive their action limits, in other words, what an environment affords related on individual characteristics. However, studies about older adults seem to be scarcer [4–7], particularly studies focused on whether or not faller and non-faller older adults can accurately perceive affordances for a stepping forward task.

**Objective**

The purpose of this study was to determine if older people could accurately perceive affordances for the task of stepping forward. The relationship between real and estimated maximum distance was explored in community-dwelling older adults, comparing fallers with non-fallers.

**Methods**

A sample of 347 community-dwelling older adults (age 73.02 ± 6.40 yr; non-fallers: 57.9%, fallers: 42.1%) with the absence of cognitive impairment participated in the study. Participants were asked to predict their maximum distance for a stepping forward prior to performing the task. Absolute Percent Error (APE), Absolute Error (AE) and Error Tendency (ET) were calculated accordingly (2.8). APE measured deviation percentage from accurate perceptions, AE indicated the discrepancy (in cm) between estimation and real performance. ET indicated the direction of the error (under- or overestimation bias).

**Results**

On average, non-faller estimated (63.7 ± 15.5 cm) and performed (70.7 ± 14.9 cm) greater distances than faller (estimation: 57.1 ± 14.5 cm; real: 61.7 ± 14.6 cm) older adults. No statistically significant differences were observed in APE (fallers: 7.2 ± 12.4 %; non-fallers: 9.6 ± 12.5 %). However, differences in AE were significant between faller (6.7 ± 5.9 cm) and non-faller (9.6 ± 12.5 cm) older adults (p = .001). Old people had a huge tendency to underestimate (77.2%) the maximum distance achieved in a stepping forward. The results show a significant association between ET and being faller (χ^2^(1) =6.407, p=.01). Despite general participants exhibit an underestimation tendency, this tendency is greater in non-fallers (61.6% vs 38.4%). Further, there were fewer non-fallers than fallers overestimating their ability to step forward (45.6% vs 54.4%).

**Conclusions**

Older adults displayed a tendency to underestimate the maximum distance they can stepping-forward. The bias of overestimation is more frequent in fallers, whereas persons who underestimated tend to do not fall, suggesting that they have a protective behaviour which avoids falls. Data evidence that older adults can perceive what the environment affords, which is in agreement with an ecological perspective to perception and action.

**Acknowledgements**

This study was funded by ESACA Project (Grant ALT20-03-0145-FEDER-000007).

**References**

1. Gibson J. The ecological approach to visual perception. New Jersey: Lawrence Erlbaum; 1979.

2. Almeida G, Luz C, Martins R, Cordovil R. Differences between Estimation and Real Performance in School-Age Children: Fundamental Movement Skills. 2016;2016:3795956.

3. Cole WG, Chan GLY, Vereijken B, Adolph KE. Perceiving affordances for different motor skills. Exp brain Res. 2013;225(3):309–319.

4. Konczak J, Meeuwsen HJ, Cress ME. Changing affordances in stair climbing: the perception of maximum climability in young and older adults. J Exp Psychol Hum Percept Perform. 1992;18(3):691–697.

5. Cesari P, Formenti F, Olivato P. A common perceptual parameter for stair climbing for children, young and old adults. Hum Mov Sci. 2003;22(1):111–24.

6. Luyat M, Domino D, Noel M. Surestimer ses capacités peut-il conduire à la chute? Une étude sur la perception des affordances posturales chez la personne âgée. Psychol NeuroPsychiatr Vieil. 2008;6(4):286–297.

7. Noel M, Bernard A, Luyat M. La surestimation de ses performances : Un biais spécifique du vieillissement? Geriatr Psychol Neuropsychiatr Vieil. 2011;9(3):287–294.

8. Almeida G, Luz C, Martins R, Cordovil R. Do Children Accurately Estimate Their Performance of Fundamental Movement Skills. J Mot Learn Dev. 2017;5(2):193-206.

**Keywords**

Aging, Falling, Perception of affordances, Gait.

### O172 A new affordance perception test to explain falls occurrence: preliminary results of stepping-forward task

#### Catarina LN Pereira, Jorge Bravo, Hugo Rosado, Gabriela Almeida

##### Department of Sports and Health, School of Science and Technology, University of Évora, 7000-671 Évora, Portugal

###### **Correspondence:** Catarina LN Pereira (clnp@uevora.pt)

**Background**

Falls cause injury, dependence, and death. Identify the subjects that are potential fallers is essential for a successful prevention. Researchers developed several models and tests in order to diagnose individual’s risk of falling [1, 2]. Risk factors such as environmental hazards, strength, balance or dual tasks are commonly tested. However, their discriminative power is limited, indicating a gap which these tests do not address. The assessment on the perception of affordances for individual’s ability to perceive the critical boundary action [3, 4], may fill this gap.

**Objective**

To analyse the appropriateness of a new stepping-forward test to explain fall occurrence in community-dwelling adults, that assess perception and action boundary.

**Methods**

Participants were 266 women and 81 men aged 73.0 ± 6.4 years. They were assessed for fall occurrence (yes *vs*. no), and for stepping-forward and perception boundaries. Participants judged their maximum stepping-forward distance prior to the performance of the estimated task. Absolute Error (AE) [|estimated – real|] (cm) and Absolute Percent Error (APE) (%) were computed, and the Error Tendency (ET) was classified (underestimation *vs*. overestimation) [5, 6].

**Results**

Univariate binary regression analysis showed that all the described variables explain significantly fall occurrence (p < 0.05). Data showed that, for each additional cm estimated in the stepping-forward test, the likelihood of falling decreased on 2.9%, OR: 0.971 (95%CI: 0.957-0.986), and for each additional cm performed in the test, this likelihood decreased on 4.0%, OR: 0.960 (95%CI: 0.945-0.975). Furthermore, data showed that for each additional cm computed as AE, the likelihood of falling decreased on 3.6%, OR: 0.964 (95%CI: 0.933-0.996), and for each additional 1% computed as APE this likelihood decreased on 0.9%, OR: 0.991 (95%CI: 0.969-1.013). Finally, data showed that subjects reporting an ET of underestimation were less 47.7% likely for falling, OR: 0.523 (95%CI: 0.315-0.867), than subjects showing an ET of overestimation.

**Conclusions**

The new stepping-forward affordance perception test evidenced to be useful to determine the risk of fall occurrence. A higher estimation of maximum distance achieved or a higher real performance on the test were associated with a lower risk of falling. Further, a higher AE and an underestimation tendency showed to be associated with a decreased risk of falling. This suggests that is the marge of security provided by the higher performance ability, in contrast with a lower perception of affordance, which is protective and avoids falls.

**Acknowledgements**

This study was funded by ESACA Project (Grant ALT20-03-0145-FEDER-000007).

**References**

1. Pereira CLN, Baptista F, Infante P. Role of physical activity in the occurrence of falls and fallrelated injuries in community-dwelling adults over 50 years old. Disabil Rehabil. 2014;36(2):117–124.

2. Lohman M, Crow R, DiMilia P, Nicklett E, Bruce M, Batsis J. Operationalisation and validation of the Stopping Elderly Accidents, Deaths, and Injuries (STEADI) fall risk algorithm in a nationally representative sample. J Epidemiol Community Heal. 2017;71(12):1191–1197.

3. Luyat M, Domino D, Noel M. Surestimer ses capacités peut-il conduire à la chute? Une étude sur la perception des affordances posturales chez la personne âgée. Psychol NeuroPsychiatr Vieil. 2008;6(4):286–297.

4. Noel M, Bernard A, Luyat M. La surestimation de ses performances : Un biais spécifique du vieillissement? Geriatr Psychol Neuropsychiatr Vieil. 2011;9(3):287–294.

5. Almeida G, Luz C, Martins R, Cordovil R. Differences between Estimation and Real Performance in School-Age Children : Fundamental Movement Skills. 2016;2016:3795956.

6. Almeida G, Luz C, Martins R, Cordovil R. Do Children Accurately Estimate Their Performance of Fundamental Movement Skills. J Mot Learn Dev. 2017;5(2):193-206.

**Keywords**

Aging, Falling risk, Boundary action, Perception.

## Poster Communications

### P1 Prevalence of low back pain in surfers: associated factors

#### Beatriz Minghelli^1,2^, Inês Sousa^1^, Sara Graça^1^, Sofia Queiroz^1^, Inês Guerreiro^1^

##### ^1^School of Health Jean Piaget – Algarve, Instituto Piaget de Silves, 8300-025 Silves, Portugal; ^2^Research in Education and Community Intervention, Piaget Institute, 1950-157 Lisbon, Portugal

###### **Correspondence:** Beatriz Minghelli (beatriz.minghelli@silves.ipiaget.pt)

**Background**

Paddling in surf consists on the movement that the surfer most performs during practice, and this repeated movement, associated with a spinal hyperextension posture, may predispose to the occurrence of injuries.

**Objective**

The aim of this study was to verify the prevalence of low back pain in surfers, and its associated factors.

**Methods**

The sample consisted of 50 Algarve surfers, 40 (80%) males, aged between 9 and 57 years (24.26 ± 12.41 years). The measurement instruments consisted on a questionnaire and on the use of the KINOVEA software for movement analysis. The questionnaire contained questions about the socio-demographic characteristics of the population and about the occurrence of low back pain (at the moment, over a 12-month period and during all surfing practice). Surfers were demarcated with a tape on D8 and at the base of the sacrum. Surfers were filmed while performing the paddling movement, in the sea, using their own boards. The movement movies recorded were analysed. A line was drawn between two points, while another line was projected on the board, establishing an angle. Data analysis was performed through the application of binary logistic regression, the method entered used as a binary outcome variable for the prevalence of low back pain during all surfing practice.

**Results**

8 (16%) surfers reported low back pain at the moment of data collection, 16 (32%) reported low back pain in the last 12 months, and 23 (46%) surfers reported that they had felt low back pain throughout all their surfing practice. Spinal hyperextension angles varied between 14^o^ and 38^o^ (23.04^o^ ± 4.73^o^). Female surfers presented a higher risk of sustaining surfing-related injuries than males (odds ratio= 1.36; 95%CI: 0.33-5.55; p= 0.671), individuals who had surfed for less than five years were at 2.6 (95%CI: 0.82-8.20; p = 0.103) more risk than those who had surfed for more than 5 years, surfers with ages equal or upper to 18 years revealed 1.15 (0.38-3.49; p= 0.811) odds than younger surfers, those who didn’t participate in the championships had 1.57 (0.50-4.83; p= 0.442) more chances compared to those who participated, and surfers with an spinal hyperextension angulation above 23^o^ revealed 1.04 (0.34-3.19; p = 0.945) to be more likely to develop low back pain.

**Conclusions**

There was a high prevalence of low back pain in the surfers analysed. Thus, it is necessary to have a better biomechanical analysis of the paddle movement of surfing.

**Keywords**

Low back pain, Prevalence, Surf.

### P2 Assessment risk of work-related musculoskeletal disorders according to the RULA method in Nurses

#### Paula C Santos^1,2^, Sofia Lopes^1,3,4^, Vanessa Silva^1^, Pedro Norton^5^, João Amaro^5^, Cristina Mesquita^1,4^

##### ^1^Department of Phisioterapy, School of Allied Health, Polytechnic Institute of Porto, 4050-313 Porto, Portugal; ^2^Research Centre in Physical Activity, Health and Leisure, Faculty of Sport, University of Porto, 4050-313 Porto, Portugal; ^3^Escola Superior de Saúde de Vale de Sousa, 4585-116 Gandra, Portugal; ^4^Centro de Estudos do Movimento e Atividade Humana, Escola Superior de Saúde, Instituto Politécnico do Porto, 4200-072 Porto, Portugal; ^5^Centro Hospitalar de São João, 4200-319 Porto, Portugal

###### **Correspondence:** Cristina Mesquita (ctmesquita@ess.ipp.pt)

**Background**

In what concerns Occupational Health, the most recurrent injuries are musculoskeletal disorders, which are frequently associated with risk factors, such as, repetition of the task and handling of loads. Among health professionals, nurses are the most affected.

**Objective**

This study evaluates work-related musculoskeletal disorders (WMSDs) in nurses from a central hospital, using the Rapid Upper Limb Assessment (RULA) method.

**Methods**

This is an observational, cross-sectional study with a sample of 34 nurses from the surgery department. Collections were made through the observation of tasks performed by nurses when applying the RULA. The final score was associated with the need for intervention, for prevention of WMSDs varying between 1 and 7; no intervention is required and immediate intervention is required, respectively. Descriptive analysis of the partial and final scores, as well as the Mann-Whitney test, the Fisher exact test and the chi- square test were performed.

**Results**

The tasks with the highest risk were bed hygiene and transfers. Among the evaluated tasks, the majority of the final scores obtained were 6 and 7, which refers to a need for intervention soon or immediately, respectively. There were no significant associations between the risk of injury and gender, age or length of service.

**Conclusions**

It was concluded that most of the tasks performed by nurses presented a high final score, according to the RULA method, and the body segments with the highest risk are shoulders, neck and trunk, suggesting the need for immediate intervention.

**Keywords**

Musculoskeletal disorders, RULA, Occupational Health.

### P3 After disaster: conceptualising the extent and length of the psychological impact

#### Alice Morgado (alice.morgado@northampton.ac.uk)

##### University of Northampton, Northampton, NN2 6JU, United Kingdom

**Background**

Psychosocial responses to disasters have been widely explored in psychological and psychiatric literature. However, some issues have not yet been clarified with regards to conceptualizing disasters and addressing the long-term effects of disasters through a perspective focused on developmental and positive psychology principles.

**Objective**

The aim of this study is to explore existing research regarding psychological dimensions of exposure to disaster.

**Methods**

A literature review was conducted focusing on disaster conceptualisations and long-term adaptive functioning of those who have and have not been identified as individuals at risk for adverse outcomes. Focusing on conceptions of disaster and trauma, the extent of the impact in different populations was also considered, along with existing knowledge regarding reactions to disaster and possible factors involved.

**Results**

There has been significant effort in designing immediate and short-term relief and assistance in disasters, addressing the most common effects of exposure to trauma [1-3]. Developmental considerations have outlined differential psychological outcomes through the lifespan [4-7]. An important body of research has focused on resilience in relation to trauma [8-11], nevertheless, studies regarding long-term consequences and adaptive functioning are still scarce [12]. Efforts seem to focus more on preventing relatively immediate severe symptoms of psychopathology [13, 14] rather than on promoting long-term psychological adjustment.

**Conclusions**

Research aimed at understanding the long-term psychological effects of exposure to disasters, looking at individuals who showed and did not show psychopathology following that incident seems a sensible topic to be developed. Aiming to understand how individuals in different life stages deal with adversity and to design interventions able to support individuals in dealing with the less visible long-term effects of trauma is equally important. In addition, to focusing on the absence of psychopathology, researchers should have in mind the promotion of positive development throughout the life-span.

Researchers should develop measures that assess exposure to disaster/trauma, taking into consideration not only the type of event, dates and duration, but also the type of exposure and involved stressors, attempting to capture disaster exposure in its complexity. At the same time, research should acknowledge the importance of the meaning that individuals attribute to an event and its consequences, more than the event itself [2, 3], and consider perceived individual, family and community resources in relation to it.

**References**

1. Briere, J & Elliott, D. Prevalence, characteristics, and long-term sequelae of natural disaster exposure in the general population. Journal of Traumatic Stress. 2000, 13: 661-679.

2. Norris, F H & Wind, L H. The experience of disaster: Trauma, loss, adversities, and community effects. In Y Neria, S Galea & F H Norris (eds.) Mental health and disasters. Cambridge: Cambridge University Press. 2010. pp.29-44.

3. Park, C L. Meaning making in the context of disasters. Journal of Clinical Psychology. 2016, 72: 1234-1246.

4. Gurwitch, R H et al. When disaster strikes: Responding to the needs of children. Prehospital and disaster medicine. 2004, 19:21-28.

5. Reijneveld, S A, Crone, M R, Verhulst, F C, & Verloove-Vanhorick, S P. The effect of a severe disaster on the mental health of adolescents: A controlled study. The Lancet. 2003, 362: 691-696.

6. Vernberg, E M, Hambrick, E P, Cho, B, & Hendrickson, M L. Positive psychology and disaster mental health: Strategies for working with children and adolescents. Journal of Clinical Psychology. 2016, 72: 1333-1347.

7. Wooding, S & Raphael, B. Psychological impact of disasters and terrorism on children and adolescents: Experiences from Australia. Prehospital and disaster medicine. 2004, 19: 10-20.

8. Bonanno, G A, & Gupta, S. Resilience after disaster. In Y Neria, S Galea, & F H Norris (eds.) Mental health and disasters. Cambridge: Cambridge University Press. 2010. pp.145-160.

9. Cox, R S, Perry, K E. Like a fish out of water: Reconsidering disaster recovery and the role of place and social capital in community disaster resilience. American Journal of Community Psychology. 2011, 48: 395-411.

10. Norris, F H, Stevens, S P, Pfefferbaum, B, Wyche, K F, & Pfefferbaum, R. Community resilience as a metaphor, theory, set of capacities, and strategy for disasterreadiness. American Journal of Community Psychology. 2008, 41: 127-150.

11. Schulenberg, S E. Disaster mental health and positive psychology – Considering the context of natural and technological disasters: An introduction to the special issue. Journal of Clinical Psychology. 2016, 72: 1223-1233

12. Juen, B. State of the art on psychosocial interventions after disasters. Communication at OPSIC (Operationalising Psychossocial Support In Crisis). Tel Aviv, 13th January, 2014.

13. Briere, J & Elliott, D. Prevalence, characteristics, and long-term sequelae of natural disaster exposure in the general population. Journal of Traumatic Stress. 2000, 13: 661-679.

14. North, C S. Current research and recent breakthroughs on the mental health effects of disasters. Current Psychiatry Reports. 2014, 16: 481-489.

**Keywords**

Disaster, Psychological impact, Trauma, Resilience, Development.

### P4 Evaluation of Portuguese athletes knowledge regarding doping in sports

#### Marco Jardim^1^, André Ruivo^2^, Catarina Jesus^3^, David Cristovão

##### ^1^School of Helth, Polytechnic Institute of Setúbal, 2915-503 Setúbal, Portugal; ^2^Portuguese Sports Physiotherapy Interest Group, Portuguese Association of Physiotherapists, 2785-679 São Domingos de Rana, Portugal; ^3^Hospital de Loulé, 8100-503 Loulé, Portugal

###### **Correspondence:** Marco Jardim (marco.jardim@ess.ips.pt)

**Background**

Doping is no longer an exclusive issue in sports and has been recognized as a worldwide public health problem. A relevant part of doping violations has been discovered in athletes from all ages and every competitive level often motivated by their limited knowledge about doping in sports. Portuguese athlete’s knowledge about doping rules violations is far to be known and an accurate picture about their state of knowledge seems to be an important factor to develop effective educational anti-doping programs.

**Objective**

The purpose of this study was to evaluate the knowledge of Portuguese athletes towards doping in sports, regarding substances and methods on the prohibited list, health consequences, athletes’ rights and responsibilities and doping control procedures.

**Methods**

A cross sectional study was performed in several Portuguese sport institutions. A total of 374 non-professional athletes (83% response rate) were evaluated about knowledge on doping in sports. Self-administrated and pretested questionnaire was used to collect sociodemographic data and doping knowledge. Descriptive statistics were carried out to express athlete’s sociodemographic information and mean doping knowledge score. Chi square tests were used to assess the association between study variables and doping knowledge questions. Inferential statistics (Mann–Whitney U test and Kruskal Wallis tests, p < 0.05) were used to examine differences between study variables.

**Results**

Only 21% of the athletes demonstrated good global knowledge on doping. The overall mean knowledge on doping was 56.8 ± 13.8 were the highest mean knowledge area was observed on rights and responsibilities’ (62.7 ± 21.5), while the lowest was perceived on doping control procedures (54.0 ± 18.4). Higher global knowledge on doping was associated with female athletes, aged between 19-21 years with university educational level. No differences were found between team sports and individual sports athletes.

**Conclusions**

No national-level data had been reported so far and this study can provide useful information regarding gaps and trends about doping practices in the country. Doping knowledge among the participants was poor, particularly in terms of prohibited substances and doping control procedures. The results of this study suggest that educational anti-doping programs should be intensified and more effective amongst Portuguese sports populations.

**Keywords**

Public Health, Doping, Doping Knowledge, Sports Population, Education, Portugal.

### P5 Do children with Specific Language Impairment (SLI) present implicit learning (IL) deficits? Evidence from an Artificial Grammar Learning (AGL) paradigm

#### Ana P Soares^1^, Andreia Nunes^1^, Paulo J Martins^2^, Marisa Lousada^3^

##### ^1^Psychology Research Center, School of Psychology, University of Minho, 4710-057 Braga, Portugal; ^2^Center for Humanistic Studies of the University of Minho, University of Minho, 4710-057 Braga, Portugal; ^3^Center for Health Technology and Services Research, School of Health Sciences, University of Aveiro, 3810-193 Aveiro, Portugal

###### **Correspondence:** Ana P Soares (asoares@psi.uminho.pt)

**Background**

Specific Language Impairment (SLI) is a neurodevelopmental disorder involving language deficits in the absence of other associated condition [1]. The aetiology of SLI is hotly debated, ranging from representational deficits in grammar to impairments in the cognitive processes that underlie language acquisition. Recent research suggests that SLI difficulties may arise from implicit learning (IL) deficits, *i.e*. impairments in the cognitive mechanisms that allow children to extract the structural regularities present in the input and to generalize it to new contexts [2]. IL studies have been conducted mainly with adults and unimpaired children using the Serial Reaction Time Task (SRTT). The few studies conducted with language impaired children produced inconsistent results [3]. Since performance of this task involves a motor component that seems to be also impaired in SLI, it is critical to conduct studies using other tasks and paradigms.

**Objective**

To analyse if IL deficits are core in SLI using an Artificial Grammar Learning (AGL) task. The AGL task is particularly suited to study IL deficits in SLI because it mimics language acquisition more closely than SRTT and, in addition, avoids its motor component. In an AGL task, participants are firstly exposed to strings that conform the rules of an artificial-grammar (learning-phase). Then, they are asked to decide whether new strings conform or not these rules (test-phase). Performance is typically better to grammatical (G) than to non-grammatical (NG) strings, indicating that participants learned the grammar even without consciousness of it.

**Methods**

Fourteen Portuguese children participated in this study (M_age_=4.86, SD=.66), 7 with a SLI diagnosis matched in age, sex, and non-verbal IQ with other 7 children with typical development (TD). All children were asked to perform a visual AGL task presented as a computer game. Written consent was obtained from all parents.

**Results**

Results showed that TD children outperformed SLI children in the test-phase. More hits were also observed for the G strings that revealed higher- than lower-similarity with the strings presented in the learning-phase. Furthermore, the analysis of children performance showed that while TD children revealed an increased number of correct responses and a decreased number of attempts to achieve a correct response in the learning-phase, SLI children did not.

**Conclusions**

Children with SLI reveal deficits in their IL abilities as indexed by a worse performance both in the learning and test phases of a visual AGL task. IL malfunctioning should be considered in the aetiology of the disorder.

**References**

1. Bishop DVM. What Causes Specific Language Impairment in Children? Curr Dir Psychol Sci. 2006;15(5):217–21.

2. Lum JAG, Conti-Ramsden G, Morgan AT, Ullman MT. Procedural learning deficits in specific language impairment (SLI): a meta-analysis of serial reaction time task performance. Cortex. 2014;51(100):1–10.

3. Ullman MT, Pierpont EI. Specific language impairment is not specific to language: the procedural deficit hypothesis. Cortex. 2005;41(3):399–433.

**Keywords**

Specific Language Impairment, Implicit learning, Artificial grammar learning, Language impaired children.

### P6 Fatal road accidents: behavior and the use of safety equipment

#### Christine B Godoy^1^, Maria HPM Jorge^2^, Jackeline G Brito^1^

##### ^1^Faculty of Nursing, Universidade Federal de Mato Grosso. 78060-900 Cuiabá, Mato Grasso, Brazil; ^2^School of Public Health, Universidade de São Paulo, 01246-904 São Paulo, Brazil

###### **Correspondence:** Christine B Godoy (christineufmt@gmail.com)

**Background**

At present, road accidents represent the second major cause of death in Brazil, striking mainly the younger population [1, 2]. Several factors contribute to this reality, among them, the accelerated urbanization process with significant population growth and an increase in the number of vehicles in circulation; impunity for violators, lack of proper supervision; old vehicle fleet; poor maintenance of public roads; poor signage; alcohol and driving combination; non-use of safety equipment and improper behaviour of drivers of pedestrians and vehicles [3-6]. Considering that death is the maximum expression of a given problem in a society [7-8], learning about the factors associated to casualties from road accidents may direct prevention actions and contribute to higher effectiveness [9-10].

**Objective**

This study examines the factors related to fatal road accidents involving children, adolescents and youngsters in Cuiabá, the capital of Mato Grosso, in 2009.

**Methods**

This is a domestic survey descriptive in nature. In the first stage of the research, data were collected through Death Certificates (DO), in order to identify, mainly, victims and address. In the second stage of the study, a household survey was conducted with the families of the victims, in which information was collected on the use of safety equipment and behaviour of victims in traffic, according to the families' reports. The analysis has been done with the EpiInfo software.

**Results**

In the period and population studied, deaths occurred only due to land transport accidents (codes V01 to V89 of ICD10), generally referred to as traffic accidents, and there were no fatalities of other types of transport (such as air transport or by boat). We studied 22 deaths due to traffic accidents. Most of whom are male (86.4%). Among the motorcycle driver victims, not all were wearing a helmet (44.4%), many did not respect traffic signs (55.5%), and some used to combine alcohol consumption and driving (33.3%). Among the car driver victims, 85.7% were not using seat belts, and many used to combine alcohol consumption and driving (57.1%). The pedestrian victims were not using the Zebra crossing (50.0%) or respected the red light of the Pelican crossing (50.0%).

**Conclusions**

The results point to the need to intervene directly in risk factors in order to reduce road casualties.

**References**

1. Nukhba Z, Uzma RK, Junaid AR, Prasanthi P, Adnan AH. Understanding unintentional childhood home injuries: pilot surveillance data from Karachi, Pakistan. Inj Prev 2012; 18(Suppl 1): A97-A97.

2. Aguilera SLV, Moysés ST, Moysés SJ. Intervenções de segurança viária e seus efeitos nas lesões causadas pelo trânsito: uma revisão sistemática. Rev Panam Salud Publica 2014; 36(4): 1-13.

3. Wei Y, Chen L, Li T, Ma W, Peng N, Huang L. Self-efficacy of first aid for home acidentes among parents with 0 to 4 year old children at a metropolitan community health center in Taiwan. Accid Anal Prev. 2013; 52:182-7.

4. Fraga AMA, Fraga GP, Stanley C, Costantini TW, Coimbra R. Children at danger: injury fatalities among children in San Diego County. Eur J Epidemiol 2010;25(3):211–217.

5. Koizumi MS, Leyton V, Carvalho DG, Coelho CA, Mello Jorge MHP, Gianvecchio V et al. Alcoolemia e mortalidade por acidentes de trânsito no município de São Paulo, 2007/2008. ABRAMET – Associação Brasileira de Medicina de Tráfego 2010;28(1):25-34.

6. Mello Jorge MHP, Koizumi MS. Acidentes de trânsito como objeto de estudo da medicina de tráfego. O papel da epidemiologia. In: Moreira FDL (org). Medicina do Transporte. Rio de Janeiro: Arquimedes, 2010. P. 355-375.

7. Afzali S, Saleh A, Seif Rabiei MA, Taheri K. Frequency of alcohol and substance abuse observed in drivers killed in traffic acci¬dents in Hamadan, Iran. Arch Iran Med. 2013;16(1):240-242.

8. Andrade SSCA, Mello Jorge MHP. Estimativa de sequelas físicas em vítimas de acidentes de transporte terrestre internadas em hospitais do Sistema Único de Saúde. Ver Bras Epidemiol 2016; 19(1): 100-111.

9. Bravo MS. Aprender a dirigir aos 18 anos de idade: uma visão da psicologia nessa fase da adolescência. Boletim de Psicologia 2015; LXV(43): 147-155.

10. Ivers RQ, Sakashitaa C, Senserrickb T, Elkingtona J, Loa S, Boufousb S, Romea L. Does an on-road motorcycle coaching program reduce crashes innovice riders? A randomised control trial. Accident Analysis and Prevention 2016; 86(1): 40-46.

**Keywords**

Road accidents, External cause, Mortality, Risk factor.

### P7 Education with resource to simulated practice: gains in the implementation of gastric intubation

#### Marta Assunção^1^, Susana Pinto^1^, Lurdes Lopes^2^, Claudia Oliveira^1^, Helena José^3,4^

##### ^1^Institute of Health Sciences, Universidade Católica Portuguesa, 4200-374 Porto, Portugal; ^2^Iberoamerican University Foundation, 1990-083 Lisbon, Portugal; ^3^Health Sciences Research Unit: Nursing, Nursing School of Coimbra, 3046- 851 Coimbra, Portugal; ^4^University of Lisbon, 1649-004 Lisbon, Portugal

###### **Correspondence:** Marta Assunção (martaassuncao@icloud.com)

**Background**

Patient safety is an important issue and a challenge in today’s health care practice to reduce adverse events [1]. A strategy to minimize this problem is the clinical simulation, as it is in this context, where doubt and error are allowed [2], without jeopardizing the integrity of the person [3].

**Objective**

To analyse the technical evolution of the students regarding the accomplishment of a nursing intervention *i.e.*: gastric intubation.

**Methods**

Quasi-experimental study without control group, using medium-fidelity simulators. An observation grid focused on nursing intervention was built for data collection: gastric intubation, with twenty-one items. Sampling was through accessibility. The inclusion criteria were: to be a registered nurse; studying in a course of the Centro de Formação de Saúde Multiperfil (CFS, Angola: Luanda), with a minimum of two years of professional experience and to participate in the three study moments. The study population consisted of 37 nurses, but 7 were excluded because they were not present in all phases of the study (n=30). The first moment of study occurred in laboratory context (in the CFS laboratories), in a realistic scenario, where a clinical situation was presented in which it was necessary to perform gastric intubation, based on the knowledge held by the student. In the second moment, students participated in a theoretical approach to the procedure and trained the procedure under simulated practice. On a third moment (few days after the 2nd moment) a second observation was made. We proceeded to analysis and comparison of data using descriptive statistics.

**Results**

56.7% of the participants were female. Age ranged from 28 and 52 years, the average was thirty-nine [39.27 (± 16.97)] years. 50% of students were from the province of Luanda, the rest from other provinces of Angola. 10% of the students did not obtain gains with the simulated practice, while 90% presented a positive evolution from the first to the second observation. The most significant changes were in the following actions: head positioning, flexion nullification and swallowing request.

**Conclusions**

Similar to what is mentioned in the literature, about the gains obtained from the simulated practice in realistic scenarios, in this study, gains were also observed in the performance of a nursing intervention. The use of simulated practice in nursing education, specifically with respect to the development of instrumental skills, contributes to successful teaching, which can translate into better performance and, subsequently, less risk to the patient.

**References**

1. World Health Organization. Patient safety curriculum guide: Multiprofessional edition. Geneva, Switzerland: WHO; 2011. Available from: http://apps.who.int/iris/bitstream/10665/44641/1/9789241501958_eng.pdf.

2. Teixeira CRS, Kusumota L, Braga FTMM, Gaioso VP, Santos CB, Silva VLS, et al. O Uso de Simulador no Ensino e Avaliação Clínica em Enfermagem. Texto Contexto Enferm (Florianópolis) [serial on the Internet]. 2011 [cited 2017 October 11]; 20: 187-93. Available from: http://dx.doi.org/10.1590/S0104-07072011000500024.

3. Ferreira C, Carvalho JM, Carvalho FLQ. Impacto da Metodologia de Simulação Realística, Enquanto Tecnologia Aplicada a Educação nos Cursos de Saúde. STAES [serial on the Internet]. 2015 [cited 2017 October 11]; 32-40. Available from: www.revistas.uneb.br/index.php/staes/article/view/1617/1099.

**Keywords**

Simulation training, Education, Nursing, Clinical Competence, Intubation, Gastrointestinal, Patient safety.

### P8 Emotional labour in paediatric nursing: a propose model for practice guidance

#### Paula Diogo (pmdiogo@esel.pt)

##### Unidade de Investigação & Desenvolvimento em Enfermagem, Escola Superior de Enfermagem de Lisboa, 1600-190 Lisboa, Portugal

Health-disease processes experienced by children and youth, and their families, are often associated with intense emotionality and, simultaneously, entails a great emotional challenge for nurses in their care, requiring emotional labour of triple centrality: on the client, nurse and nurse-client relationship [1]. Nurses perform this emotional labour according to their personal resources and learning from the day-to-day experience of care [2]. Moreover, this emotional dimension of nursing care continues to be undervalued by health institutions, and by nurses themselves, so that emotional labour is not always the object of reflection and/or support in scientific evidence [3]. For this reason, conceptual models are needed to guide and strengthen nurses in their practice, especially when the context is peculiar as in paediatric care.

Diogo [1], presented an explanatory hypothesis of the process of therapeutic use of emotions in paediatric nursing, arguing that Emotional Labour in Paediatric Nursing translates into actions of positive transformation of emotional experience into interactions of care with the paediatric client, through five categories of intervention: 1) Promoting a safe and affectionate environment; 2) Nurturing care with affection; 3) To facilitate client emotional management; 4) to build stability in the relationship; 5) to regulate their own emotional disposition to care. This Emotional Labour Model in Paediatric Nursing was developed based on the nursing paradigm of transformation [4] whose central concept is Caring, supported by the Watson's Human Care theory [5], and theorizes about “personal knowing” [6]. This Model also integrates the principles of family-centred care and non-traumatic care in Paediatric Nursing, such as the holistic and humanized perspective on health. At the heart of the proposed Model is the Emotional Labour of Nursing conception [2].

**References**

1. Diogo P. Trabalho com as emoções em Enfermagem Pediátrica: Um processo de metamorfose da experiência emocional no ato de cuidar. 2ª ed. Loures: Lusodidacta; 2015.

2. Smith P. Emotional Labour of Nursing Revisited. Can nurses Still Care? 2ª ed. Hampshire: Palgrave Macmillan; 2012.

3. Diogo P, compilador. Investigar os Fenómenos Emocionais da Prática e da Formação em Enfermagem. Loures: Lusodidacta; 2017.

4. Kérouac S, Pepin J, Ducharme F, Duquette A, et al. El pensamiento enfermero. Barcelona: Masson; 1996.

5. Watson J. Nursing: The Philosophy and Science of Caring. Boulder: University Press of Colorado; 2008.

6. Fawcett J, Watson J, Neuman BH, Fitzpatrick JJ. On nursing theories and evidence. J Nurs Scholarsh. 2001; 33(2): 115-119.

**Keywords**

Emotions, Emotional Labour, Conceptual Model, Peadiatric Nursing.

### P9 Cardiovascular risk factors in patients with ischemic and hemorrhagic stroke

#### Ilda Barreira^1^, Matilde Martins^2^, Leonel Preto^2^, Norberto Silva^1^, Pedro Preto^3^, Maria E Mendes^2^

##### ^1^Serviço de Urgência, Unidade Local de Saúde do Nordeste, 5301-852 Bragança, Portugal; ^2^Departamento de Enfermagem, Escola Superior de Saúde, Instituto Politécnico de Bragança, 5300-146 Bragança, Portugal; ^3^Serviço de Ortotraumatologia, Unidade Local de Saúde do Nordeste, 5301-852 Bragança, Portugal

###### **Correspondence:** Leonel Preto (leonelpreto@ipb.pt)

**Background**

Stroke is the second worldwide most common cause of death and the main reason of functional disability [1]. Early identification and treatment of modifiable risk factors can reduce the risk of stroke. In stroke patients, the identification of cardiovascular risk factors is also important for preventing another stroke [2].

**Objective**

To assess the prevalence of cardiovascular risk factors in stroke patients.

**Methods**

Analytical and retrospective cohort study. Data were collected through electronic health records of all patients with stroke admitted to an emergency department for seven years (2010 to 2016). The research protocol has been approved by an ethics committee.

**Results**

Were analysed the electronic health records of 756 patients with ischemic stroke (78.6 ± 10.7 years) and 207 with intracerebral haemorrhage (76.1 ± 11.9 years). In ischemic stroke, the most common risk factors were hypertension (66.7%), hypercholesterolemia (30.7%), diabetes mellitus (26.5%), atrial fibrillation (25.4%), obesity (11.4%) and smoking (5.2%). In haemorrhagic stroke the most prevalent risk factors were hypertension (57.0%), diabetes (25.6%), dyslipidaemia (23.7%), atrial fibrillation (17.4%), obesity (15.5%) and smoking (9.2%).

**Conclusions**

Hypertension was more prevalent in ischemic stroke and is associated with type of stroke (χ^2^ = 6.633, df = 1, p = 0.010). Atrial fibrillation also prevailed in thromboembolic events with statistical significance (p = 0.016). Diagnosis and control of cardiovascular risk factors is a fundamental objective for primary and secondary prevention of stroke.

**References**

1. Donnan GA, Fisher M, Macleod M, Davis SM. Stroke. Lancet. 2008;371(9624):1612-23.

2. Arboix A. Cardiovascular risk factors for acute stroke: Risk profiles in the different subtypes of ischemic stroke. World J Clin Cases. 2015;3(5):418-29.

**Keywords**

Prevalence, Cardiovascular risk factors, Ischemic stroke, Hemorrhagic stroke.

### P10 Topical oxygen therapy in wound healing: a systematic review

#### João L Simões, Dilsa A Bastos, Raquel V Grilo, Marta L Soares, Sílvia S Abreu, Juliana R Almeida, Elsa P Melo

##### School of Health Sciences, University of Aveiro, 3810-193 Aveiro, Portugal

###### **Correspondence:** João L Simões (jflindo@ua.pt)

**Background**

Oxygen is recognised as an essential element in the wound healing process and, it is suggested that the topical application of oxygen may be a promising therapy in wound care. Thus, the importance of oxygen in the tissue healing process is evident, namely in ATP synthesis; production of reactive oxygen species, which stimulate vascular endothelial growth factor synthesis; and microbial growth inhibition through the promotion of macrophage chemotaxis and increase of leukocyte activity. Moreover, oxygen increases the rate of collagen deposition, an important step in healing, which supplies the matrix for angiogenesis and tissue maturation. Thus, according to the P.I.C.O. review model for clinical questions, this systematic review intends to answer the research question “*In chronic wounds, how does topical oxygen therapy affects wound healing*?”. It was considered chronic wounds for “patient population or disease of interest”, topical oxygen therapy for “intervention or issue of interest” and wound healing for “outcome”. However, a “comparison intervention or group” and a “time frame” were not applicable.

**Objective**

The aim of this study was to conduct a systematic review of the current evidence for this therapy through the analysis of primary research studies published between January 2006 and December 2016.

**Methods**

Published literature was identified using Scopus, B-On, Scielo, Pubmed, Ebsco Host and Medline databases. Exclusion criteria and quality indicators were applied and a total of 11 articles with different designs were included in the review.

**Results**

The studies analysed emphasise the evidence of additional O_2_ usage in wound care, since it reduces hypoxia and it allows triggering mechanisms which are essential for the healing process. The analysed literature presents the results of its effects in its various forms: pressurized, continuous and dissolved. Although there are still questions about the exact mechanisms of this treatment and it is necessary to carry out randomised studies, the current results suggest that this therapy plays an important role in restoring the O_2_ balance in the wound bed, necessary for healing.

**Conclusions**

These findings show the potential of this therapy in promoting healing of chronic wounds and improving people’s quality of life. In addition, there are many other potential advantages related to its usage, such as low cost, apparent safety, no associated adverse effects and the possibility to submit a diversified population to this care at any health organisation or even at the patient’s home.

**Keywords**

Oxygen, Topical administration, Wound Healing, Wounds and Injuries.

### P11 Microbiological characterization of bathing areas of a county in the Northern region

#### Joana Mendes, Marlene Mota, António Araújo, Cecília Rodrigues, Teresa Moreira, Manuela Amorim

##### Escola Superior de Saúde, Instituto Politécnico do Porto, 4200-072 Porto, Portugal

###### **Correspondence:** Manuela Amorim (mas@ess.ipp.pt)

**Background**

The management of bathing water aims at the protection of human health and the preservation, protection and improvement of the quality of the environment [1, 2]. In order to control the quality of these same waters for recreational use, microbiological indicators of faecal contamination are monitored, according to Decree-Law 135/2009 of June 3rd [1]. The microbiological indicators of faecal contamination used are *Escherichia coli* and *Enterococcus spp*. since they are commensals of the gastrointestinal flora of humans and most animals [3].

**Objective**

This study aimed to characterize the results of intestinal *E. coli* and *Enterococcus* parameters of inland bathing waters of a county in the northern region of Portugal during 2016.

**Methods**

A retrospective descriptive study was performed using database records from a northern laboratory. The microbiological parameters studied to characterize the inland bathing waters included CFU/100mL of *E.coli* and CFU/100mL of intestinal *Enterococcus*. The results were classified as “Bad”, “Acceptable”, “Good” or “Excellent”, according to the Decree-Law 135/2009 of June 3rd [1].

**Results**

We verified that in the total of 26 inland bathing waters under study, 6 (23.1%) obtained a quality equal to or greater than “Acceptable”. The remaining 20 bathing waters (76.9%) were classified as “Bad”. This result, in 17 samples was due to both parameters, intestinal *Enterococcus* and *E. coli*. In the other three, the “Bad” classification was only due to the *Enterococcus* results. The months with the highest counts of *E. coli* were September (45.69%), June (43.30%) and May (39.62%), and for *Enterococcus* were May (52.83%), June (52.58%) and July (32.35%).

**Conclusions**

In an initial study and applying criteria that will then have to be more extended in terms of time, there is a first tendency for most of the inland bathing waters under study to present “Bad” quality (76.90%). Since all bathing waters should have at least “Acceptable” quality and provisional data, these results indicate an urgent need to take measures in order to counteract this and increase the number of bathing waters classified as “Excellent” or “Good.” The different *E.coli* and intestinal Enterococcus counts observed in different months showed that climatic, environmental, social and urban factors could be involved in this differences and deserves attention in future studies [2, 4]. The quality of bathing water is fundamental in terms of public health. In this sense, the results of this study are worrisome, however these studies should be conducted in a longer time perspective.

**References**

1. Portugal. Decreto-Lei n.º 135/2009, de 3 de junho de 2009. Estabelece o regime de identificação, gestão, monitorização e classificação da qualidade das águas balneares e de prestação de informação ao público sobre as mesmas. Diário da República n.º 107/2009. 3460-3468.

2. Portugal. Decreto-Lei n.º 113/2012, de 23 de maio de 2012. Gestão da qualidade das águas balneares, e ao seu ajustamento ao quadro institucional resultante da publicação do Decreto-Lei n.º 7/2012, de 17 de janeiro, que define a orgânica do Ministério da Agricultura, do Mar, do Ambiente e do Ordenamento do Território, e do Decreto-Lei n.º 56/2012, de 12 de março, que define a orgânica da Agência Portuguesa do Ambiente, I.P.. Diário da República, 1ªa série, n.º 100. 2715-26.

3. Boehm AB, Sassoubre LM. Enterococci as Indicators of Environmental Fecal Contamination. In: Gilmore MS, Clewell DB, Ike Y, Shankar N, editors. Enterococci From Commensals to Leading Causes of Drug Resistant Infection. Boston: Massachusetts Eye and Ear Infirmary; 2014.

4. McMichael AJ. Environmental change, climate and population health: a challenge for inter-disciplinary research. Environmental Health and Preventive Medicine. 2008, 13(4):183-186.

**Keywords**

Inland bathing water, Fecal contamination indicators, Escherichia coli, Enterococci intestinal.

### P12 Microbiological characterization of food handlers in school canteens

#### Diana Gomes, Teresa Moreira, Marlene Mota, Cecília Rodrigues, António Araújo, Manuela Amorim

##### Escola Superior de Saúde, Instituto Politécnico do Porto, 4200-072 Porto, Portugal

###### **Correspondence:** Manuela Amorim (mas@ess.ipp.pt)

**Background**

Food-borne substances are a major concern of Public Health, given that food can be the source of various hazards (biological, physical and chemical). Approximately 20% of outbreaks of foodborne illness are associated with the personal hygiene of food handlers. The personal hygiene of manipulators is one of the best ways to block bacterial contamination and its extension to new areas [1, 2].

**Objective**

To evaluate the microbiological profile of the hands of food handlers in school canteens of the northern region of Portugal during 2016 and to verify the efficiency of the hygiene processes.

**Methods**

Handlers and utensils were tested using a swab soaked in Maximum recovery diluent-Histidine Lecithin and Polysorbate (MRD-HLPS) rubbing against parts were food might get retained, following ISO 18593: 2004 [3]. The parameters evaluated were coliforms at 37ºC/24h, *Escherichia coli* at 44ºC/24h and coagulase positive *Staphylococcus* at 37ºC/48h, according to ISO 4832:2006 [4], ISO 16649-2:2001 [5] and ISO 6888-1:1999 [6], respectively. A statistical analysis of the results of the microbiological profile evaluation was carried out at the hands of the food handlers of public primary school canteens.

**Results**

Ours results of the microbiological profile of the hands of food handlers showed that 9.95% of samples analysed had bacterial contamination. Most of the samples with bacterial contamination were caused by the presence of coliforms, followed by coagulase positive *Staphylococcus*. Only one sample was registered with positive *E. coli*. It was not found a significant difference in the proportions of samples with bacterial contamination and positive for coliform bacteria and coagulase positive *Staphylococcus*, in the distribution line and in the kitchen, over the several months.

**Conclusions**

The food handler is an important and recognized source of bacterial contamination of foodstuffs [1, 2]. The results of the present study indicate the necessity to implement measures to control bacterial contamination in the hands of manipulators of school canteens, aiming at correcting possible flaws encountered. Food legislation, the Hazard Analysis and Critical Control Point (HACCP) system, and reference documents such as the Codex Alimentarius and the Food Code present guidelines to promote improved food hygiene and personal hygiene for handlers [2, 7].

**References**

1. Arduse L, Brown D. HACCP and Sanitation in Restaurants and Food Service Operations. Florida: Atlantic Publishing Company; 2005.

2. World Health Organization, Food and Agriculture Organization. Codex Alimentarius: Higiene dos alimentos. 3rd ed. Brasilia: Agência Nacional de Vigilância Sanitária; 2006.

3. International Organization for Standardization. ISO 18593:2004, Microbiology of food and animal feeding stuffs — Horizontal methods for sampling techniques from surfaces using contact plates and swabs; 2004.

4. International Organization for Standardization. ISO 4832:2006, Microbiology of food and animal feeding enumeration of coliforms — Colony-count technique; 2006.

5. International Organization for Standardization. ISO 16649-2:2001, Horizontal method for the enumeration of B-glucuronidase-positive Escherichia coli; 2001.

6. International Organization for Standardization. ISO 6888-1:1999, Horizontal method for the enumeration of coagulase-positive staphylococci (Staphylococcus aureus and other species); 2003.

7. Food and Drug Administration. Food Code. Virginia: United States Department of Health and Human Services; 2013.

**Keywords**

Food safety, Food handlers, Hand hygiene, Microbiological evaluation, Bacterial contamination.

### P13 Exploring the effectiveness of digital psychoeducational interventions on depression literacy: a scoping review

#### Karin Panitz, Jennifer Apolinário-Hagen

##### Department of Health Psychology, Institute for Psychology, University of Hagen, 58097 Hagen, Germany

###### Karin Panitz (mail@hp-panitz.de)

**Background**

Depression is a huge burden requiring efficient strategies for prevention and treatment [1]. Psychoeducation can improve health literacy and help to reduce the stigma of help-seeking. In recent years, the Internet has been suggested as a way to deliver mental health interventions to a broader range of persons and to reduce barriers to seek help from face-to-face services. However, little is known about the effectiveness of digital psychoeducational interventions on health literacy and psychological outcomes, such as help-seeking intentions [2].

**Objective**

To derive practical implications for health professionals, this scoping review aimed to explore the effectiveness of different digital psychoeducational interventions strengthening depression literacy or knowledge (primary outcome), stigmatizing attitudes and on help-seeking attitudes, intentions and behaviour (secondary outcomes). This review is conceptualized as an update and expansion of previous research [2] with a focus on a broad range of interventions.

**Methods**

In May 2017, a systematic search through electronic databases (e.g. PsycINFO and PSYNDEX) was performed to identify longitudinal studies on the effectiveness of digital interventions targeting depression-related mental health literacy among adults published between 2007 and 2017 in peer-reviewed English journals.

**Results**

Overall, 19 Studies met the inclusion criteria, mostly stemming from Australia. The findings of 13 of the included 17 studies evaluating mental health literacy revealed significant increases in depression literacy. Pure dissemination of information via websites, e-mails or psychoeducational interventions yielded primarily positive findings. Both Internet-based Cognitive Behavioural Therapy and online game programs were found to be knowledge-enhancing, except for one study using a simulated dialogue. Findings on digital intervention targeting stigmatization in terms of individual, as well as perceived attitudes towards mental illness were inconsistent. Concerning perceived stigma, 4 of 8 studies showed positive results in reducing stigma, whereas other results were inconsistent. Likewise, the effects of interventions on help-seeking (n= 8 studies) with respect to attitudes (n= 5 studies), intentions (n= 6 studies) and behaviour (n= 4 studies) were inconclusive.

**Conclusions**

The evidence base on mental health literacy interventions is promising, but still limited. Various digital interventions are overall comparably effective in strengthening depression literacy and reducing stigmatizing attitudes. Given several limitations, future research should compare subpopulations to understand what works are best for whom in clinical practice. Furthermore, the comparability of knowledge levels of healthy and depressed persons should be considered. Finally, eHealth literacy of clients and health professionals should be explored and, where required, promoted with evidence-based information.

**References**

1. Kessler RC. The Costs of Depression. The Psychiatric Clinics of North America. 2012;35(1):1-14. doi:10.1016/j.psc.2011.11.005.

2. Brijnath B, Protheroe J, Mahtani KR, Antoniades J. Do Web-based Mental Health Literacy Interventions Improve the Mental Health Literacy of Adult Consumers? Results From a Systematic Review. Journal of Medical Internet Research. 2016;18(6):e165. doi:10.2196/jmir.5463.

**Keywords**

Depression, Mental health, Health literacy, eHealth, Review.

### P14 Family nurse as a privileged caregiver of families of patient with wounds in domiciliary context: nurse’s perspective

#### Maria FMS Nunes^1^, João L Simões^2^, Marília S Rua^2^

##### ^1^Unidade de Saúde Familiar Flor de Sal, Agrupamento de Centros de Saúde do Baixo Vouga, 3800-039 Aveiro, Portugal; ^2^Escola Superior de Saúde, Universidade of Aveiro, 3810-193 Aveiro, Portugal

###### **Correspondence:** João L Simões (jflindo@ua.pt)

**Background**

Population ageing is a reality that has contributed to the increase of chronic diseases and the number of dependent people with wounds, with the need of home care. This issue has implications in family dynamics. It is important to take care not only of the person with the wound but also of its family. These new health needs led to the reorganization of primary health care, where family nurses emerged as essential professionals.

**Objective**

The aim of this study is to know the perception of family nurses of ACeS Baixo Vouga about their care with families of patient with wounds, in domiciliary context, and the importance given to this nursing practice with families. On the other hand, to identify the factors that nurses consider as barriers or facilitators in their work with families.

**Methods**

It was made a quantitative, descriptive and correlational study. The instrument used for data collection was a questionnaire with two parts. The first part aimed to characterize the sample with the sociodemographic and professional data of the participants, while the second one was built with two questions and the scale of the Perception about Family Nursing. The sample consisted of 150 nurses working in primary health care, in USF or UCSP, of ACeS Baixo Vouga, ARSC. Data processing was made by a descriptive and inferential analysis using the Statistical Package for Social Sciences (SPSS) and a qualitative analysis through content analysis.

**Results**

The results for the subscale Perception of Family Nursing Practice (PFNP) showed that the nurses selected “often” in most of the questions. The PFNP isn’t affected by sociodemographic and professional variables. This subscale is only affected by the nurses’ formation variable. The nurses with curricular formation on family have higher level of applicability of the family nursing in practice. For the subscale Importance Assigned to Family Nursing (IAFN), the most relevant category was “important”. The IAFN is affected by sociodemographic, professional and formation variables. The group of nurses with a higher degree of education give more importance to family nursing.

**Conclusions**

Nurses attribute a higher level of importance on the nurse’s care with families of patient with wounds in the domiciliary context than on the applicability of the family nursing in practice. The characteristics of nursing care are the most relevant facilitator factor for family nursing practice. The characteristics of the institution are the most mentioned as a barrier factor.

**Keywords**

Family nurse, Family, Home care, People with wounds.

### P15 Physical resilience as a key concept in the prevention of frailty in the elderly

#### Rafael Bernardes, Cristina L Baixinho

##### Lisbon Nursing School, 1700-063, Lisbon, Portugal

###### **Correspondence:** Rafael Bernardes (rafael.alvesbernardes@gmail.com)

**Background**

The concept of frailty has been presented in the literature in a variety of ways [1-5] and in close relation with negative health outcomes, such as gait difficulty, falls and weight loss [1, 5]. The correct assessment of frailty in the elderly and the design of an adequate care plan is essential for the provision of personalized care and assistance to caregivers [1-3]. Recent studies associate this concept with physical resilience, as a personal characteristic that determines the capacity to resist functional decline or restore physical health, being a central aspect in active aging [6].

**Objective**

Identify the characteristics of physical resilience that modify (positively or negatively) the fragility of the elder.

**Methods**

Integrative literature review (RIL) to answer the question “*How can physical resilience influence frailty in the elder?*”.

**Results**

Fragility, although a significant syndrome linked to the natural aging process, can be modified [2]. The contextual factors of each person, if well evaluated and controlled, can improve functionality not only physically but also cognitively and socially. Interventions that reduce vulnerability and adverse outcomes reduce the risk of hospitalization. The ability of an elderly person to withstand external stress is strongly related to the physiological reserve [5-6]. Taking into account that one of the main components of the fragility phenotype is sarcopenia, many of the interventions must be operationalized in order to prevent it [6]. The phenomenon of loss of functionality and muscle mass is not an isolated phenomenon [5] and produces negative functional outcomes such as difficulty in climbing stairs, getting up from a chair or bed and lifting heavy objects. The optimization of physical resilience can happen through the design of programs of physical exercise, nutrition, therapeutic reconciliation, psychoeducational support and support by health professionals.

**Conclusions**

Physical resilience is also influenced by factors common to frailty. The main constraint of physical resilience that affects fragility is the physiological reserve. Resilience can be quantified in three ways by determining functional trajectories: “resilient” (without functional changes after adverse events) or “resilient” (functional decline with subsequent recovery) by Physical Resilience levels: “fragile phenotype” vs “robust phenotype” and by determining “chronological age” versus “biological age”. This possibility of quantification “opens” the door for the development of interventions to treat fragility.

**References**

1. Anzaldi LJ, Davison A, Boyd CM, Leff B, Kharrazi H. Comparing clinician descriptions of frailty and geriatric syndromes using electronic health records: a retrospective cohort study. BMC Geriatr. 2017;17:248. DOI 10.1186/s12877-017-0645-7.

2. Fairhall N, Langron C, Sherrington C, et al. Treating frailty-a practical guide. BMC Med. 2011;6,9:83.doi: 10.1186/1741-7015-9-83.

3. Bieniek J, Wilczynski K, Szewieczek J. Fried frailty phenotype assessment components as applied to geriatric inpatients. Clin Interv Aging. 2016;11:453-59. doi: 10.2147/CIA.S101369. eCollection 2016.

4. Bongue, B; Buisson, A; Dupre, C; Beland, F; Gonthier, R; Crawford-Achour, E (2017). Predictive performance of four frailty screening tools in community-dwelling elderly. BMC Geriatr 17(1):262. doi: 10.1186/s12877-017-0633-y.

5. Zaslasvky O, Cochrane BB, Thompson HJ, Woods NF; LaCroix A. Frailty: A Review of the First Decade of Research. Biol Res Nurs. 2013;15(4) 422-32. doi: 10.1177/1099800412462866.

6. Whitson HE, Duan-Porter W, Schmader KE, Morey MC, Cohen HJ, Colón-E CS. Physical Resilience in Older Adults: Systematic Review and Development of an Emerging Construct. J Gerontol A Biol Sci Med. 2016;71(4):489-95. doi: 10.1093/gerona/glv202.

**Keywords**

Motor activity, Nursing, Sarcopenia, Frail elderly, Dependence.

### P16 Safety Protocol for Nasolaringoscopic Evaluation of Swallowing: cultural and linguistic validation and adaption for European Portuguese language

#### Liliana Abreu^1^, Pedro S Couto^2^, Susana Mestre^3^

##### ^1^Faculty of Medicine, Lisbon University, 1649-028 Lisbon, Portugal; ^2^Center for Research and Development in Mathematics and Applications, Department of Mathematics, University of Aveiro, 3810-193 Aveiro, Portugal^; 3^University Hospital Center of Algarve, 8000-386 Faro, Portugal

###### **Correspondence:** Pedro S Couto (p.sa.couto@ua.pt)

**Background**

In practice, a Speech Therapist works with several neurological diseases that present changes in swallowing, especially after acute stroke. These changes, called dysphagia, can lead the patient to death by leading to malnutrition, dehydration, tracheal aspiration and recurrent pneumonia [1]. Since most of these cases are diagnosed in a hospital setting, it becomes increasingly important to create working tools that help health professionals to perform more rigorous therapeutic evaluations and interventions.

**Objective**

The present study aims to contribute to the cultural and linguistic validation and adaptation of the Protocol of Security of a Nasolaryngoscopy Evaluation of Swallowing (PSAND).

**Methods**

The study comprises two parts: a qualitative part, that corresponds to the translation and adaptation of the protocol to European Portuguese Language, and a quantitative part, where the psychometric characteristics of the protocol were studied. Further details about translation and adaptation of the protocol can be found in [2], specially the content validity procedures and its application in a pilot study. A severity assessment scale [3] was used for the functional evaluation of the swallowing safety by classifying the swallow of the subjects as normal, penetration or aspiration. For data collection, it was used the Portuguese adaptation of the PSAND and the nasolaryngoscope as evaluation tools. The content validity index (CVI) was calculated for the qualitative part, and t-student or qui-squared tests were used for comparison between severity groups.

**Results**

The sample consisted of twenty subjects, where all of them have an acute stroke as clinical diagnosis whether or not having dysphagia. The age of the inquired ranged from 31 to 85 years old, being 16 males. The results obtained by the panel of experts allowed us to conclude that all the parameters are relevant to the evaluation of swallowing and important to determinate a safe feeding for each case (CVI>0.80). Thus, by applying the PSAND, it was possible to study two groups: “Penetration” (13 patients) and “Aspiration” (5 patients). There were statistically significant differences (p < 0.05) between the two groups for the variables: dependent or independent feeding; poor oral control; lot of residues; reduction of laryngeal sensitivity; leaking of the bolus and difficulty in cleaning pharyngeal residues.

**Conclusions**

In summary, we can say that the application of this protocol is an asset to diagnose the presence of dysphagia in any clinical diagnosis, evaluate the swallowing function, verifying the risk of penetration and aspiration and classifying the Dysphagia Severity.

**Acknowledgements**

This work was supported in part by the Portuguese Foundation for Science and Technology (FCT-Fundação para a Ciência e a Tecnologia), through Center for Research and Development in Mathematics and Applications (CIDMA), within project UID/MAT/04106/2013.

**References**

1. Michou E, Hamdy E. Cortical input in control of swallowing. Current opinion in Otolaryngology & Head and Neck Surgery. 2009 June; 17:166-71.

2. Abreu, Liliana. Protocolo de Segurança na Avaliação Nasolaringoscópica da Deglutição (PSAND): contributo para a validação cultural e linguística do português Europeu [Master Thesis] [Portuguese]. Escola Superior de Saúde do Alcoitão. 2016.

3. Rosenbeck JC, Robbins JA, Roecker EB, Coyle JL, Wood JL. A penetration Aspiration Scale. New York: Spring; 1996.

**Keywords**

Swallowing, Dysphagia, AVC, Evaluation, PSAND.

### P17 Trend in obesity in an aging society: estimate of obese elderly in Brazil in 2030

#### Adriane Carvalho, Roger S Rosa, Scheila Mai, Rita Nugem, Ronaldo Bordin

##### Federal University of Rio Grande do Sul, Porto Alegre, Rio Grande do Sul, 90040-060, Brazil

###### **Correspondence:** Adriane Carvalho (adrianedasc@hotmail.com)

**Background**

Population aging and the increasing longevity of older people are increasingly relevant worldwide phenomena [1]. In addition, along with ageing, a significant increase in the prevalence of obesity among the elderly is also occurring [2,3].

**Objective**

To estimate the increase in the number of obese individuals, due exclusively to population aging in Brazil from 2014 to 2030.

**Methods**

The number of obese adult Brazilians was obtained by extrapolation of the prevalence estimated by VIGITEL (Surveillance System for Risk and Protection Factors for Chronic Diseases by Telephone Inquiry) [4] in Brazilian capitals, in 2014, for the entire Brazilian population. The population projection for 2030 by age groups was obtained from IBGE (Brazilian Institute of Geography and Statistics) [5]. The prevalence obtained by VIGITEL in 2014 was applied to population projections by 2030, maintaining all other variables constant, with 95% confidence intervals (95% CI).

**Results**

The Brazilian adult population (18 +years) corresponded to 144.5 million people in 2014 of whom 15.5 million (10.7%) were 65 years of age or older. Obese adults accounted for 25.9 million (95% CI 24.9-27.0 million) of the entire adult population (17.9%), of which 3.1 million (95% CI 2.8-3.3 million) were elderly obese. The obese elderly corresponded to 11.9% of adults with obesity. In 2030, it is estimated that the Brazilian adult population will reach 175.2 million people, of whom 30.0 million (17.1%) are elderly. Obese will correspond to 31.4 million (95% CI 30.1-32.8 million) of adult Brazilians of whom 5.9 million (95% CI 5.4-6.4 million) will be obese elderly. That is, exclusively due to aging, it is expected an increase of 5.5 million obese for the entire population. An estimated 2.8 million more are obese in the age group of 65 and over. Therefore, it is expected that the percentage of 11.9% of elderly among obese adults in 2014 will rise to 18.9% in 2030.

**Conclusions**

Considering only the effect of aging with current levels of obesity prevalence, it is estimated that there will be an increase of almost 3 million obese people in Brazil by 2030. The impact of the increase in prevalence itself was not considered, which would make the prospect even more worrying due to the impact on chronic non-communicable diseases and in the use of health services.

**References**

1. Ministério da Saúde (BR). Secretaria de Atenção à Saúde. Estatuto do Idoso. Brasília: Ministério da Saúde, 2013.

2. Ferreira VA, Magalhães R. Obesidade no Brasil: tendências atuais. Rev Port Saude Publica. 2006;24(2):71-81.

3. Mártires MAR, Costa MAM, Santos CSV. Texto Contexto Enferm, Florianópolis. 2013 Jul-Set;22(3):797-803.

4. Malta DC, Bernal RI, Nunes ML, Oliveira MM, Iser BM, Andrade SC, et al. Prevalência de fatores de risco e proteção para doenças crônicas não transmissíveis em adultos: estudo transversal, Brasil 2012. Epidemiol Serv Saúde, Brasília. 2014 Dez;23(4):609-22.

5. Instituto Brasileiro de Geografia e Estatística [homepage na internet]. Projeção da População do Brasil por sexo e idade: 2000- 2060 [acesso em 10 dez 2017]. Disponível em: https://ww2.ibge.gov.br/home/estatistica/populacao/projecao_da_populacao/2013/default_tab.shtm.

**Keywords**

Obesity, Aging, Tendencies, Population projection, Demography.

### P18 Nursing interventions towards the hospitalized elderly patient with delirium – a systematic review of literature

#### Marta Bento, Rita Marques

##### Universidade Católica Portuguesa, 1649-023 Lisboa, Portugal

###### **Correspondence:** Marta Bento (marsofia81@hotmail.com)

**Background**

Delirium is one of the most prevalent neuropsychiatric syndromes in the hospital setting, preferably in the elderly debilitated patients. It is a cognitive alteration of sudden onset, developing in a matter of hours or days; which is interspersed with periods of lucidity and also characterized by disturbances in attention, memory and behaviour. It is also identified by the worsening of the symptoms at night and by changes in the sleep-wake cycle. The presence of this syndrome, makes impossible a holistic care, upsetting an effectively communication, between patient and nurse or family. It may even be considered common for an elderly, given the age, to appear confused, but it should not be considered normal, so investing in concrete studies to specify these mental changes and determinate what interventions are more appropriate for this vulnerable group, is emergent. It is up to nurses, who are in a privileged position, the early recognition/intervention at this neurological condition. It is assumed as an emerging need, to implement non-pharmacological strategies, so that the occurrence of delirium decreases and thus avoids great suffering.

**Objective**

This study aimed to identify the nursing interventions directed to the hospitalized elderly, for the control and prevention of delirium.

**Methods**

Using the methodology recommended by the Cochrane Centre, this systematic review of literature was guided by the following research question: “*What is the scientific evidence regarding nursing interventions directed to the hospitalized adult/elderly for the control of delirium*?” Using a PICO framework as reference, a review of articles published between 2012 and 2017 was carried out. The research was conducted at B-ON and EBSCO host - Research Databases.

**Results**

In this bibliographic review 5 studies were selected, in common, they present tendentially, non-pharmacological strategies adopted by nurses with preventive character towards the predisposing and precipitating factors of delirium. The role of nursing in carrying out preventive actions was important in the maintenance of the sensorial balance (frequently reorientation, encouraging the use of visual and hearing aids improves patients ‘sensorium), optimizing circadian rhythm (minimizing night procedures, allowing periods of rest), assessing the local environment (limiting background noise and light) as well as in the mental status, pain, monitoring hydration, nutrition and stimulation of early mobility.

**Conclusions**

The implementation of nursing delirium preventive measures truth sensibilized professionals reveals to be effective in reducing the incidence of delirium. Research is imperative, to recognize and validate witch interventions may better control delirium and thus reduce its consequences.

**Keywords**

Delirium, Nursing interventions, Hospitalized adult patients, Evidence-based practice.

### P19 Distribution of gama-chamber nuclear equipment is associated to the distribution of physicians in the state of Rio Grande do Sul, Brazil

#### Patrícia Silva, Roger S Rosa, Rita Nugem, Adriane Carvalho, Ronaldo Bordin

##### Federal University of Rio Grande do Sul, 90040-060 Porto Alegre, Rio Grande do Sul, Brazil

###### **Correspondence:** Patrícia Silva (patriciairan@ig.com.br)

**Background**

The use of effective technologies extends the resolution of health services. However, over-supply can create incentives for service over-use, which is not without risk to patients. Nuclear medicine equipment has been increasingly used. Knowing the associations with their spatial distribution can contribute to interventions aimed at reducing inequalities.

**Objective**

To dimension the association among mean number of equipment’s of gamma-chamber, population, Gross Domestic Product and number of physicians, by health region of Rio Grande do Sul, state of southern Brazil.

**Methods**

Observational and cross-sectional descriptive study based on public data from each one of the 30 health regions for 2013, the most recent year at the time of the survey (2016-2017). Data was managed in Microsoft Excel®. Pearson's linear correlation coefficient and multiple linear regression analysis were used with Statistica 12.5® software, at a significance level of 5%. The variable considered for outcome was monthly mean of gamma camera equipment (GamaC) and the predictor variables (I) population (POP), expressed in number of inhabitants; (II) Gross Domestic Product (GDP), expressed in the national coin (Real); and (III) the number of physicians registered in the CNES - National Register of Health Establishments (MED) by health region of the State Health Secretariat, in 2013.

**Results**

The predictive variables POP, GDP and MED were each one highly correlated with GamaC (R = 0.94, 0.92 and 0.98 respectively). Simple linear regressions with each independent variable were elaborated. It was found that POP, PIB and MED significantly affected the GamaC variable (adjusted R^2^ of 0.89, 0.84 and 0.96 respectively). In the final model, where variables were standardized and GamaC was considered to be simultaneously dependent on the predictive variables POP, GDP and MED, the POP variable lost significance (p > 0.05). The variable PIB presented a negative coefficient (-0.54, p < 0.01), while the variable MED, a positive (1.27, p < 0.01).

**Conclusions**

Health regions of the state that had the highest number of physicians, had the highest mean number of scintigraphic chambers. The growth in the supply of medical equipment such as nuclear medicine improves the population's access to services, but the greater supply in Rio Grande do Sul state was associated more with better developed health regions, when considering the number of medical professionals available, than the gross domestic product or the number of residents in the territory.

**Keywords**

Nuclear medicine, Supply, Health needs, Demand of health services.

### P20 Family experiences of the internalized person in situation of critical illness: integrative revision

#### Raquel MV Ramos, Ana CR Monteiro, Sílvia P Coelho

##### Instituto de Ciências da Saúde, Universidade Católica Portuguesa, 4169-005 Porto, Portugal

###### Raquel MV Ramos (raquel_mvr@hotmail.com)

**Background**

The admission of a patient to a critical health unit is usually traumatic for the family, having a major impact on their life, which can result in a moment of crisis, an anxiety enhancer. Fear of death, uncertainty of the future, emotional disturbances, financial worries, changing roles and routines, and the hospital environment are some sources that provide anxiety of a person's family in critical illness [1].

**Objective**

To know the existent evidence about Family Experiences of the Person hospitalized in Situation of Critical Illness.

**Methods**

Integrative literature review using databases CINAHL, MEDLINE, Nursing & Allied Health Collection: Comprehensive, Cochrane, Library, Information Science & Technology Abstracts, Medication with MeSH descriptors: “family”, “needs assessment” and “critical illness”. Were included all English-language articles, available in full text, with abstract and references available, between 2002 and 2017, excluding articles in the paediatrics area.

**Results**

In total, 7 were selected and 4 articles were analysed in full. From the literature, it emerges that the family of the person hospitalized in a critical illness has experiences and needs consequent to this situation, in which it is necessary an intervention from the professionals to support/to encourage during this traumatic transition of the familiar life [2]. The family has its own needs, and these must be met to effectively manage the situation of instability of the family member. Since the family directly influences the evolution of a person's condition in a critical illness situation, it is important to see the family also as a target of care, in a holistic view of caring [3]. The main areas of need experienced by the family are: information on the clinical situation, assurance of patient safety, support by health professionals and willingness to be close to the patient [2].

**Conclusions**

Health professionals should be aware that the family is also a target in care, and that, in a multidisciplinary team, nurses are the most qualified professionals to plan and develop interventions to meet and respond to the family needs of the person hospitalized in critical illness [4]. The team must be able to respond to the identified family needs, through interventions to attenuate and help them to live the moment of hospitalization, making it the least traumatic possible, involving the relatives in the care, through the clarification of doubts and by helping to manage emotions and expectations [3].

**References**

1. Leske J. Interventions to Decrease Family Anxiety. Critical Care Nurse.2002, 22 (6): R61-65.

2. Kinrade T, Jackson A, Tomnay J. The psychosocial needs of families during critical illness: comparison of nurses’ and family members’ perspectives. Australian Journal of Advanced Nursing. 2009, 27 (1): R82-88.

3. Henneman E, Cardin S. Family-Centered Critical Care: A Practical Approach to Making it Happen. Critical Care Nurse. 2002, 22 (6), R12-19.

4. Fortunatti C. Most important needs of family members of critical patients in light of the Critical Care Family Needs Inventory. Invest Educ Enferm. 2014, 32 (2): R306-316.

**Keywords**

Needs assessment, Family, Critical illness.

### P21 Cannabidiol oil vs ozonized extra virgin olive oil in the upp treatment of category ii

#### Carla Jimenez-Rodriguez^1^, Francisco J Hernández-Martínez^2^, María C Jiménez-Díaz^1^, Juan F Jiménez-Díaz^3^, Bienvenida C Rodríguez-De-Vera^3^

##### ^1^Universidad de Jaén, Universidad de Jaén, 23071 Jaén, España; ^2^Cabildo de Lanzarote, 35500 Lanzarote, Las Palmas, Islas Canarias, España; ^3^Universidad de Las Palmas de Gran Canaria, 35015 Las Palmas de Gran Canaria, España

###### **Correspondence:** Carla Jimenez-Rodriguez (carlajimenezrodriguez@gmail.com)

**Background**

Pressure ulcers (UPP) Category II are shallow open wounds. The phytotherapeutic treatments for them are based on healing and antiseptic action. This effect is produced by cannabidiol oil. Also, extra virgin olive oil (EVOO) ozonized has repairing properties with germicidal power.

**Objective**

To determine the effectiveness of cannabidiol oil versus EVOO in the treatment of UPP.

**Methods**

Clinical trial with 60 users with UPP Category II. After the informed consent of the patients, data collection was done in September 2017. Criterion of inclusion: it was essential that each of the users had at least two chronic wounds with the same injury (Category II), in order to apply in each one a product. We excluded users with vascular disease or in situations of extreme severity. Each user included in the study was followed for 20 days. Skin assessment and initial risk assessment was performed with the Braden scale by the principal investigator and another investigator of the team. Subsequently, the skin condition of the patients was evaluated daily, before the application of the product, by the nurse who attended them. Additionally, the patients were evaluated every 7 days by two investigators. The SPSS 25.0 program was used for statistical calculations, considering a level of significance of p < 0.05.

**Results**

Average age 71.45 ± 1.27 years. Of a total of 137 chronic wounds, 56.93% were located in the lower limbs. Regarding the resolution of the wounds, no significant differences were found between the two products, since 68.61% of the lesions improve significantly using both products before 72 hours, and all of them heal at the most in 8 days. It did not appear topically on the skin, no allergic reaction due to the use of both products, ansd the application of cannabis oil on the wound was very well tolerated by patients (p < 0.37).

**Conclusions**

Cannabidiol oil is shown to be as effective as EVOO in the treatment of UPP Category II, both being a good alternative to traditional therapies. In addition, the moisturizing, emollient and anti-inflammatory properties of the two products preserve the perilesional skin in perfect condition. Cannabidiol oil achieves a more favourable analgesic response in patients during wound healing.

**Keywords**

Pressure ulcers, Cannabidiol oil, Extra virgin olive oil ozonized, Traditional therapies.

### P22 Microbial colonization of experimental ulceras in the laboratory animal treated with cannabidiol oil

#### Carla Jiménez-Rodríguez^1^, Carmelo Monzón-Moreno^2^, Juan F Jiménez-Díaz^2^, María-del-Carmen Jiménez-Díaz^1^, Bienvenida-del-Carmen Rodríguez-de-Vera^2^

##### ^1^Universidad de Jaén, Universidad de Jaén, 23071 Jaén, España; ^2^Universidad de Las Palmas de Gran Canaria, 35015 Las Palmas de Gran Canaria, España

###### **Correspondence:** Carla Jiménez-Rodríguez (carlajimenezrodriguez@gmail.com)

**Background**

One of the most undesirable complications in the healing process is infection in the bed of wounds or ulcers.

**Objective**

To verify the microbial colonization of experimental ulcers in the laboratory animal treated with cannabidiol oil (CBD) applied topically.

**Methods**

Experimental study with a control group (with physiological saline to maintain hydration conditions and group with extra virgin olive oil (EVOO) to avoid bias with the oleic excipient), to check the mesophilic microbial colonization after the use of the applied CBD topically on experimental total skin ulcerative lesions in the adult male white rat, Sprague Dawley strain. Ten animals were used for each group under standard laboratory conditions. After anaesthesia with 100% isoflurane, a total skin wound was performed in the region of the back with a disposable surgical punch of 8 mm in diameter. Subsequently, they are distributed in individual cages to prevent them from licking each other and with sufficient height to prevent the friction of the cutaneous ulcer with the passenger compartment. 0.15 ml of the respective product was applied daily to the ulcers. The microbiological analysis was carried out by studying the variation of the bacterial microbiota. The colony-forming units of each wound were determined by counting on a plate, after obtaining a total skin sample and a superficial sweep. The organic samples obtained were placed in sterile tubes containing 1 ml of physiological serum to which vortex was applied for 30 seconds, serial dilutions being made to the tenth of the samples subject to titration. Six plates of Tryptic Soy Agar (TSA) were then labelled, one for each dilution obtained, and 0.1 ml of each of these dilutions was added, spreading it on the surface of the plate by means of the sowing handle. Plates were incubated in an oven at 37° for twenty-four hours and then the colony forming units were counted.

**Results**

Two hundred fourteen different colonies were obtained. The majority genus was *Staphylococcus*. There was no difference in microbial colonization due to the products used in each group, *i.e.*, physiological serum, EVOO and CBD.

**Conclusions**

The analysis of the mesophilic cutaneous microbiota shows a microbial colonization rich in gram-positive organisms, the majority being the presence of coagulase-negative staphylococci (CNS) that behave as opportunistic pathogens in skin continuity solutions.

**Keywords**

Colonization, Microbial cannabidiol, Skin, Ulcer, Rat.

### P23 The impact of dermatologic and cosmetic counseling - case study

#### Stefany Moreira^1^, Ana Oliveira^2^, Rita Oliveira^2,3^, Cláudia Pinho^2^, Agostinho Cruz^2^

##### ^1^Escola Superior de Saúde, Instituto Politécnico do Porto, 4200-072 Porto, Portugal; ^2^Centro de Investigação em Saúde e Ambiente, Escola Superior de Saúde, Instituto Politécnico do Porto, 4200-072 Porto, Portugal; ^3^Secção Autónoma de Ciências da Saúde, Universidade de Aveiro, 3810-193 Aveiro, Portugal

###### **Correspondence:** Stefany Moreira (fany_hill@hotmail.com)

**Background**

Community pharmacy professionals (CPPs) have been recognized as the most accessible and best-positioned health professionals for the provision of pharmaceutical counselling [1]. This happens due to the easy access to pharmacies, and because their interventions translate into: beneficial clinical results; satisfaction of users; reduction of costs and prevention of problems or negative reactions to medicines [1, 2]. The sale of dermatological products and symptoms associated with skin problems has a considerable impact on sales and advice requirements in pharmacies, respectively [3].

**Objective**

To demonstrate the importance of CPPs through the quantitative evaluation of the impact of dermatologic and cosmetic counselling; and to determine which dermatological/cosmetic areas affect most people and what motivates them to turn to this type of counselling.

**Methods**

Prospective, longitudinal and an observational case study. It took place in a pharmacy in the city of Porto, between January and April 2017. It had 3 phases: I) invitation (where were explained the objectives and the methodology); II) first Interview: Completion of PART I of the Questionnaire (description of the situation and the advice provided by the CPP); III) second Interview: Completion of PART II of the Questionnaire (evaluation of the result of the counselling).

**Results**

Of the 16 analysed situations: 62.50% were resolved and/or people were satisfied, 31.25% were in the process of improvement, and 6.25% were not resolved and/or people were not satisfied. The three most mentioned dermatological/cosmetic areas in the requests for counselling were: daily skin care (37.50%); marks, spots, comedones, pimples or signs on the skin (18.75%) and sun protection (12.50%).

**Conclusions**

CPPs have proven to be very valuable in providing counselling on dermatologic products and cosmetics, where, this had a positive impact. The dermatological/cosmetic area that most had expression among the requested situations was daily skin care.

**References**

1. Curley LE, Moody J, Gobarani R, Aspden T, Jensen M, McDonald M, et al. Is there potential for the future provision of triage services in community pharmacy? J Pharm Policy Pract. 2016;9(29):1-22.

2. Coelho RB, Costa FA. Impact of pharmaceutical counseling in minor health problems in rural Portugal. Pharmacy Practice. 2014 Oct;2(4).

3. Tucker R, Stewart D. Why people seek advice from community pharmacies about skin problems. Int J Pharm Pract. 2015;23:150-3.

**Keywords**

Community pharmacy professionals, Counselling, Dermatologic products, Cosmetics.

### P24 Ability of clients for self-management of medication regime: specification of nursing diagnosis

#### Inês Cruz, Fernanda Bastos, Filipe Pereira

##### Nursing School of Porto, 4200-072 Porto, Portugal

###### **Correspondence:** Inês Cruz (inescruz@esenf.pt)

**Background**

There is a growing concern to understand the experience of living with multiple morbidities and the need to manage a medication regime [1, 2] by people experiencing one or more health/disease transitions [3], in order to assist them in this process. Being human responses to different transitions the object of the Nursing discipline, these professionals must identify and represent the nursing care needs of clients in the Nursing Information Systems in use, which are a repository of the Discipline knowledge.

**Objective**

Identify and specify the nursing diagnoses centred on the ability for self-management of the medication regime, as a type of self-care in situations of health deviation.

**Methods**

Qualitative study. All nursing documentation customised in the Portuguese nursing information System - SAPE® (2012) and in Sclinico (2016) - was subject to content analysis. After conducting content analysis, the authors presented it to a group of 14 nursing experts in the field, to reach consensus.

**Results**

From the analysis of the national customisations, we infer a set of nursing diagnoses related to the person's abilities to manage the medication regime. These diagnoses focus on the potential to improve the ability for: self-management of the medication regime; self-management of the medication regime using devices; administering medication; administering subcutaneous medication; administering insulin; administering inhalant medication; administering oxygen therapy; self-monitoring in face of the medication regime; self-monitoring of capillary glycemia; self-monitoring heart rate in face of administering medication; self-monitoring blood pressure in face of administering medication; and self-monitoring urine.

**Conclusions**

The specified diagnoses reflect nursing care needs of people who are challenged to live with chronic illnesses, particularly at the level of skills they need to develop in order to manage the medication regime. It is necessary that nurses identify these needs to prescribe interventions that improve the ability of the person to administer medication, with or without the use of devices; by different routes; and to monitor some physiologic parameters related to the medication taken. We believe this will be a first contribution to the representation of the nursing knowledge in this area.

**References**

1. Meranus M, Engstrom G. Experience of self-management of medications among older people with multimorbidity. J Clin Nurs. 2015; 24: 2757-2764.

2. Duguay C, Gallagher F, Fortin M. The experience of adults with multimorbidity: a qualitative study. J Comorbidity. 2014; 411-21.

3. Meleis A, Sawyer L, Im E, Messias H, DeAnne K, Schumaker K. Experiencing transitions: an emerging middle-range theory. Advances in Nursing Science. 2000; 23 (1): 12-28.

**Keywords**

Self-management, Medication regime, Nursing diagnosis, Nursing information systems.

### P25 Antioxidant activity of Artemisia annua L

#### Rita Vieira^1^, Cláudia Pinho^2^, Ana I Oliveira^2^, Rita F Oliveira^2,3^, Agostinho Cruz^2^

##### ^1^Escola Superior de Saúde, Instituto Politécnico do Porto, 4200-072 Porto, Portugal; ^2^Centro de Investigação em Saúde e Ambiente, Escola Superior de Saúde, Instituto Politécnico do Porto, 4200-072 Porto, Portugal; ^3^Secção Autónoma de Ciências da Saúde, Universidade de Aveiro, 3810-193 Aveiro, Portugal

###### **Correspondence:** Cláudia Pinho (clp@eu.ipp.pt)

**Background**

Tea infusions of *Artemisia annua* are known for their prophylactic and therapeutic efficacy against malaria [1]. However, recent studies have revealed that *A. annua* possess a variety of pharmacological activities such as antibacterial, cytotoxic and antioxidant [2, 3].

Objective

This study aims to evaluate antioxidant activity of *A. annua* plant obtained from two different manufactures, prepared using different solvents.

**Methods**

*A. annua* leaves (obtained from two manufactures) were extracted with two solvents (water and 70% ethanol), and antioxidant activity of the extracts were screened using superoxide and 1,1-diphenyl-2-picryl hydrazyl (DPPH•) radical scavenging, and metal chelating activity.

**Results**

The extracts tested not only showed ability to bind to iron ions but also demonstrated ability to inhibit free radicals. Results showed that antioxidant activity increased with increasing concentrations of the extracts studied. The IC50 values of *A. annua* aqueous extract (infusion), obtained from manufacture A, for DPPH and superoxide radical scavenging activities, and Fe^2+^ chelating activity, ranged from 29.3 to 176.6 μg/mL. For the hydroalcoholic extract, IC50 values ranged from 28.0 to 262.1 μg/mL (all above standards). The IC50 values of *A. annua* aqueous extract (infusion), obtained from manufacture B, for superoxide and DPPH radical scavenging activities, and Fe^2+^ chelating activity, ranged from 6.9 to 282.0 μg/mL. For the hydroalcoholic extract, IC50 values were 40.4, 46.8 and 50.5 μg/mL for Fe^2+^ chelating activity, superoxide and DPPH radical scavenging activities, respectively. Only the aqueous extract, obtained from manufacture B, showed an IC50 value (6.9 μg/mL), for the superoxide radical scavenging activity, lower than positive control (20.6 μg/mL - ascorbic acid).

**Conclusions**

This study confirms the differences in antioxidant activities using different solvents, suggesting that the solvent effect should be taken into account in the evaluation of the antioxidant potential of any sample. However, the origin of the plants including the pre- and post-harvesting practices can be also important for their chemical composition, resulting in different values for the same antioxidant assays and solvents.

**References**

1. van der Kooy F, Verpoorte R. The content of artemisinin in the Artemisia annua tea infusion. Planta Med. 2011, 77(15):1754-6.

2. Kim WS, Choi WJ, Lee S, Kim WJ, Lee DC, Sohn UD, Shin HS, Kim W. Antiinflammatory, Antioxidant and Antimicrobial Effects of Artemisinin Extracts from Artemisia annua L. Korean J Physiol Pharmacol. 2015, 19(1):21-7.

3. Singh NP, Ferreira JF, Park JS, Lai HC. Cytotoxicity of ethanolic extracts of Artemisia annua to Molt-4 human leukemia cells. Planta Med. 2011, 77(16):1788-93.

**Keywords**

*Artemisia annua*, Antioxidant activity, Solvent extraction, DPPH, Superoxide anion radical, Metal chelating activity.

### P26 Swimming pool users and behaviors: practices and motivations

#### Daniel A Marinho^1,2^, Luís Faíl^1^, Mário C Marques^1,2^, António Sousa^1,2^, Henrique P Neiva^1,2^

##### ^1^Department of Sport Sciences, University of Beira Interior, 6201-001 Covilhã, Portugal; ^2^Research Center in Sports Sciences, Health Sciences and Human Development, University of Tras-os-Montes and Alto Douro, 5001-801 Vila Real, Portugal

###### **Correspondence:** Daniel A Marinho (marinho.d@gmail.com)

**Background**

Health and sports professionals have recommended water-based exercises as an alternative to traditional dry-land exercise, leading to an increase in physical exercise performed in an aquatic context. The properties of the aquatic environment, combined with the resistance of the water during all movements, make it beneficial for health-related parameters and physical fitness [1]. However, research is needed to understand the practices of different populations, according to the specificity of some activities. Few is known about people’s practices in these particular activities.

**Objective**

The purpose of this study is to characterize Portuguese practices and motivations to use the swimming pools and to exercise in-water physical activities.

**Methods**

Subjects from the interior region of Portugal, swimming pool users, completed a questionnaire consisting of 33 questions. Those questions were focused on the characterization of their usual in-water activities, and main motivations.

**Results**

Until now, 418 swimming pool users answered the questions, ranging from 18 to 79 years old (44.7% females, 55.3% males). Most of them were active and only 67 subjects were retired from work. They used to practice aquatic actives for more than 2 years (60%), and the majority twice-a-week, preferring the evening time to attend the swimming pool. Among the various types of swimming pool use, it was verified that 31% perform water aerobics, 48% swimming classes and 31% free time schedules. More than half of the sample only performed aquatic activities (54%) and aimed to improve health (47%), physical fitness (31%) and 11% to relief stress. Curiously, only 1% wanted to learn how to swim. They classified the physical activities performed in-water in the last few weeks mostly of moderated/vigorous intensity. People who attend to swimming pools are persistent and committed with aquatic exercitation, practicing for more than two years. Although most of them participate in swimming or water aerobics lessons, there is still a considerable number of free-time users and swimming pools must be prepared for this fact. Interestingly, the majority attend to swimming pool to improve health and physical fitness.

**Conclusions**

This pilot study will be implemented in several other regions of the country and this would allow to understand the motivations and needs of users and to improve offers and support to other areas of research (*i.e.*, development of technological devices).

**Acknowledgements**

This project was supported by the Project NanoSTIMA: Macro-to-Nano Human Sensing, Towards Integrated Multimodal Health Monitoring and Analytics, NORTE-01-0145-FEDER000016, co-financed by European Fund for Regional Development (FEDER) - NORTE 2020.

**References**

1. Barbosa TM, Marinho DA, Reis VM, Silva AJ, Bragada JA. Physiological assessment of head-out aquatic exercises in healthy subjects: a qualitative review. J Sports Sci Med. 2009, 8(2): 179-189.

**Keywords**

In-water activities, Questionnaire, Physical activity.

### P27 Critical patient’s comfort:sStrategies to reduce environmental noise levels

#### Telma Ramos, Filipa Veludo

##### School of Nursing, Institute of Health Sciences, Universidade Católica Portuguesa, 1649-023 Lisbon, Portugal

###### **Correspondence:** Telma Ramos (telmaramos_24@hotmail.com)

**Background**

Noise may have harmful effects. For critically ill patients, highlights have main consequences cardiovascular disorders, reduction of arterial oxygen saturation, increase in gastric secretion, stimulation of the pituitary gland, sleep disturbance, immunosuppression and reduction of the cicatrisation process [1]. Noise has an overall negative impact on patients’ recovery. Identification and dissemination of strategies to reduce environmental noise empowers nurses towards changes in their professional practice.

**Objective**

Identify evidence in Literature of nursing care strategies to reduce environmental noise in critical patient care. **Methods**

This research was conducted in two phases. 1st Phase: Mediated by an integrative literature review (16/04/2017) we carried out data-base research through the Academic Search Complete; Complementary Index; CINAHL Plus with Full Text; Directory of Open Access Journals; Supplemental Index; Psychology and Behavioural; Sciences Collection; SPORTDiscus with Full Text; RCAAP; SciELO; Europeana; Business Source Complete; Education Source; IEEE Xplore Digital Library; MedicLatina; JSTOR Journals; PsycARTICLES; ScienceDirect. Descriptors: (TI (Noise*or sleep*) AND (Nurs*) AND (intervention or care or patient care or care plan* or critical care), non-temporal. Inclusion criteria: Primary, secondary, opinion/reflexion studies. Exclusion Criteria: Paediatrics context, REM, pharmacological intervention. From the initially 441 articles obtained, we excluded 391 by reading abstracts, 22 by summary and 15 by the complete text, concluding with 13 articles as final sample. 2nd Phase: Content analysis according to [2] in order to categorize results.

**Results**

We have identified 6 feasible categories for environmental noise reduction, which we present as main strategies: Behavioural changes (creation of awareness to the importance of the tone of voice and silent handling of equipment and materials); Material and Equipment management (mobile phones, televisions and radios volume configuration; determination of correct parameters for alarm configuration); Management of silence promotion care (implementation of periods of silence, avoid noisy tasks); Training in environmental noise (behavioural change programs and health education about negative effects of noise); Care quality control (usage of ear plugs); Others (infrastructural adaptations, encourage suppliers to produce more silent products).

**Conclusions**

This study systematizes strategies to be implemented by nursing professionals in order to reduce environmental noise within health structures and improve patient comfort. The implementation of a silence culture enables an adequate and essential physical environment to patient recovery [3]. Empower nurses with the identified strategies allows the improvement of people’s quality of life. The shortage of published research reflects the need of forward research.

**References**

1. Christensen, M. (2007). Noise levels in a general intensive care unit: a descriptive study, Nurse Critical Care, 12(4), 188-97.

2. Bardin, L. Análise de Conteúdo. Lisboa: Edições 70 Lda, 2016.

3. Nightingale F. Notas Sobre Enfermagem: o que é e o que não é. Loures: Lusociência; 2005.

**Keywords**

Noise, Comfort, Integrative literature review, Content analysis.

### P28 Nurse-patients’ family interaction in ICU and the establishment of effective therapeutic partnerships: vulnerability experienced and clinical competence

#### Anabela Mendes (anabelapmendes@esel.pt)

##### Escola Superior de Enfermagem de Lisboa, 1600-190 Lisboa, Portugal

**Background**

When faced with a negative event such as the situation of a critical illness, nurses and patients’ family build their interaction on a daily basis [1-3]. The closeness and the joint interest in finding together the answer to their common issues motivates them for a common path, based on trust [4]. We find that nurses' clinical exercise time and their clinical competence can influence this process [5, 6].

**Objective**

To analyse how family perceives the interaction between nurses and the family. To diagnose how to build a daily basis interaction when facing a critical illness, and which steps highlight the existing confidence. Having Benner's theoretical framework as a support, we need to understand the relationship between the time spent in critical care and nurses’ clinical competence.

**Methods**

Qualitative study. Data collection through open interview to 12 family members, of an adult person hospitalized in ICU. The interviews content analysis was carried out according to the phenomenological approach suggested by Van Manen. The Software used for qualitative data analysis was Nvivo. This software showed the advantages of time saving and allowed to carefully explore the relationship between the data [7].

**Results**

Family members recognized how determinate the interaction with nurses to their daily life in the ICU was. They reported the careful construction of discourse and the effective presence with the sick person as nurses' strategy for interaction. The need to know better the situation and to discover what will happen, motivated families to start the interaction. Trust was revealed in founded solicitude and compassion. Families know that nurses are vulnerable to their suffering. During interaction, family members noticed that clinical competence is inherent to the nurse person and not related to the time of practice.

**Conclusions**

The co-existence compromises nurses and family in the construction of an effective therapeutic partnership. They recognized that the information they have from the sick person, arising from different circumstances, must be shared, considering professional ethics, beliefs and values and also the relevance for the therapeutic process. It is in interaction and for the interaction that they discover vulnerability, comfort and trust each other.

**References**

1. Curtis, J. Caring for patients with critical illness and their families: the value of integrated clinical team. Respiratory care. 2008, 53 (4): 480-487.

2. Mendes, A. A informação à família na unidade de cuidados intensivos: Desalojar o desassossego que vive em si. Lisboa : Lusodidacta, 2015.

3. Mendes, Aa. Sensibilidade dos profissionais face à necessidade de informação: a experiencia vivida pela família na unidade de cuidados intensivos. Texto Contexto Enferm. 2016, pp. 5(1):2-9 http://dx.doi.org/10.1590/0104-07072016004470014.

4. Benner, P., Kyriakidis, P. e Stannard, D. Clinical wisdom and interventions in acute and critical care. A Thinking-in-action approach. 2ª. New York : Springer publishing company, 2011. 1ª edição- 1999. 978-0-8262-0573-8.

5. Benner, P., et al., et al. Educating nurses. A call for radical transformation. San Francisco : The carnegie foundation for the advancement of teaching, 2010. 978-0-470-45796-2.

6. Benner, P., Tanner, C. e Chesla, C. Expertise in nursing practice. Caring, clinical judgement & ethics. New york : Springer publishing company, 2009. 978-0826 12544-6.

7. Forte, E., et al., et al. A Hermenêutica e o Software Atlas.ti: União promissora. Texto Contexto Enferm. 2017, 26(4).

**Keywords**

Family, Nursing, Intensive care, Interpersonal relations, Communication.

### P29 Effectiveness of vein visualization technologies on peripheral intravenous catheterization: a systematic review protocol

#### Anabela S Oliveira^1^, João Graveto^1^, Nádia Osório^2^, Paulo Costa^1^, Vânia Oliveira^1^, Luciene Braga^4^, Isabel Moreira^5^, Fernando Gama, Daniela Vidal, João Apóstolo^6^, Pedro Parreira^1^

##### ^1^Health Sciences Research Unit, Nursing School of Coimbra, Coimbra, 3046-851, Portugal; ^2^Coimbra Health School, Polytechnic Institute of Coimbra, Coimbra, 3046-854, Portugal; ^3^Coimbra Hospital and Universitary Centre, 3000-075 Coimbra, Portugal; ^4^Federal University of Viçosa, Minas Gerais, 36570-900, Brazil; ^5^Nursing School of Coimbra, 3046-851 Coimbra, Portugal; ^6^Portugal Centre for Evidence Based Practice: a Joanna Briggs Institute Centre of Excellence, 3046-851 Coimbra, Portugal

###### **Correspondence:** Pedro Parreira (parreira@esenfc.pt)

**Background**

The insertion of a peripheral vascular catheter (PVC) is the most often invasive procedure performed in hospital settings [1-3]. During hospitalization, 33.0-96.7% of patients need to have a PVC inserted [4-6]. These devices are not risk-free, affecting patients’ safety and well-being. In fact, up to 72.5% of the PVCs are removed due to complications [6]. Healthcare professionals should consider using specific technologies that help to select the vein to puncture and reduce the number of attempts and catheter-related mechanical complications.

**Objective**

This review aims to identify and synthesize the effectiveness of the use of vein visualization technologies (near-infrared light or ultrasonography) in patients who need peripheral intravenous catheterization when compared with the traditional technique.

**Methods**

Methodology proposed by Joanna Briggs Institute [7]. A three-step search strategy was used in this review: (I) an initial limited search was undertaken followed by an analysis of the words contained in the title and abstract, and of the index terms used to describe the article; (II) a second search using all identified keywords and index terms was undertaken across all included databases; (III) the reference list of all identified reports and articles was searched for additional studies. Studies of quantitative evidence published between 1999 and 2017 were considered for inclusion in this review. This review included patients of all ages, in any clinical setting. However, studies where patients displayed a previous vascular access device *in situ* were excluded. The assessment of methodological quality, data extraction and synthesis will be conducted by two independent reviewers using standardized tools recommended by the Joanna Briggs Institute [7]. Any arising disagreements will be resolved through discussion or with a third reviewer.

**Results**

An initial limited search of MEDLINE via PubMed and CINAHL was undertaken, using specific terms such as: catheters; cannula; “vascular access devices”; “peripheral intravenous catheterization”; “peripheral venous catheterization”; “peripheral access”; “peripheral intravenous access”; “venous access”; NIR*; near-infrared*; infra-red*; light*; device*; machine*; ultrasonograph*; technolog*; sonography*; ultrasound*. Resultantly, 2,699 studies were retrieved, written in English, Portuguese, Spanish and French. Keywords and index terms are being identified in order to generate a more comprehensive search strategy (step two).

**Conclusions**

The critical analysis of existing data will contribute to the dissemination of the best evidence available on the subject. It is expected that this dissemination will be reflected in the definition of guidelines regarding PVC management and, consequently, in the optimization of current practices.

**Acknowledgements**

This protocol is part of the project “Transfer of technological innovations to nursing practice: a contribution to the prevention of infections”, funded from the European Regional Development Fund, by the Operational Program Competitiveness and Internationalization of PORTUGAL 2020.

**References**

1. Marsh N, Webster J, Mihala G, Rickard CM. Devices and dressings to secure peripheral venous catheters to prevent complications. Cochrane Database Syst Rev. 2015; 6:1-14.

2. Wallis MC, McGrail M, Webster J, Marsh N, Gowardman J, Playford EG et al. Risk factors for peripheral intravenous catheter failure: a multivariate analysis of data from a randomized controlled trial. Infection control and hospital epidemiology. 2014;35(1):63-8.

3. Webster J, Osborne S, Rickard C, New K. Clinically-indicated replacement versus routine replacement of peripheral venous catheters. Cochrane Database Syst Rev. 2015;8. Art. No.: CD007798.

4. Grüne F, Schrappe M, Basten J, Wenchel H, Tual E, Stützer H. Phlebitis Rate and Time Kinetics of Short Peripheral Intravenous Catheters. Infection. 2004;32(1):30-32.

5. Pujol M, Hornero A, Saballs M, Argerich M, Verdaguer R, Cisnal M et al. Clinical epidemiology and outcomes of peripheral venous catheter-related bloodstream infections at a university-affiliated hospital. Journal of Hospital Infection. 2007;67(1):22-9.

6. Braga LM. Práticas de enfermagem e a segurança do doente no processo de punção de vasos e na administração da terapêutica endovenosa [PhD Thesis]. Universidade de Lisboa; 2017.

7. Peters M, Godfrey C, McInerney P, Baldini Soares C, Khalil H, Parker D. Chapter 11: Scoping Reviews. In: Aromataris E, Munn Z, ed. by. Joanna Briggs Institute Reviewer's Manual [Internet]. The Joanna Briggs Institute; 2017 [cited 14 December 2017]. Available from: https://reviewersmanual.joannabriggs.org/.

**Keywords**

Peripheral intravenous catheterization, Near-infrared light, Ultrasonography, Traditional technique.

### P30 Falls Efficacy Scale-International: how does it “behave” with users of adult day care centres?

#### Daniela Figueiredo^1,2^, Martina Neves^1^

##### ^1^School of Health Sciences, University of Aveiro, 3810-193 Aveiro, Portugal; ^2^Center for Health Technology and Services Research, School of Health Sciences, University of Aveiro, 3810-193 Aveiro, Portugal

###### **Correspondence:** Daniela Figueiredo (daniela.figueiredo@ua.pt)

**Background**

The Falls Efficacy Scale-International (FES-I) is a highly reliable instrument to assess fear of falling among older adults. However, the majority of validation studies with the FES-I are conducted with independent and relatively healthy community-dwelling older people, which limits extrapolation to those receiving adult day care services. Reference to higher disability and frailty is common among adult day care users compared to non-users.

**Objective**

This study presents preliminary findings of the psychometric properties of the European Portuguese version of the FES-I in a sample of older users of day care centres.

**Methods**

A cross-sectional study with a convenience sample was conducted. Data collection included a socio-demographic questionnaire, and the Portuguese versions of the FES-I and the Activities-specific Balance Confidence Scale (ABC). Descriptive and inferential statistical analyses were performed.

**Results**

A total of 100 older people users of day-care centres (81.94 ± 6.43 years old; 77% female) have participated in the study. FES-I had excellent internal consistency (α = 0.970) and test-retest reliability (ICC2,1=0.979). A significant negative correlation was found between the FES-I and the ABC (rs = -0.828; p < 0.001), indicating good concurrent validity. FES-I scores were significantly higher among those who were older, female and less educated.

**Conclusions**

The FES-I seems to be a reliable and valid measure of fear of falling for older people who are clients of adult day care services. The findings are highly comparable with those previously found for non-users of day-care centres. FES-I can be also used to prevent risk of falls in this type of care settings.

**Acknowledgements**

This paper was supported by ERDF (European Regional Development Fund) through the operation POCI-01-0145-FEDER-007746 funded by the Programa Operacional Competitividade e Internacionalização – COMPETE2020 and by National Funds through FCT - Fundação para a Ciência e a Tecnologia within CINTESIS, R&D Unit (reference UID/IC/4255/2013).

**Keywords**

Falls Efficacy Scale-International, Older people, Adult day care, Fear of falling, Psychometric properties.

### P31 Function-Focused Care: validation of self-effecacy, outcomes expectations and knowledge scales

#### Lénia Costa^1^, Pedro Sá-Couto^2^, João Tavares^3,4^

##### ^1^Department of Medical Sciences, University of Aveiro, 3810-193 Aveiro, Portugal; ^2^Center for Research and Development in Mathematics and Applications, Department of Mathematics, University of Aveiro, 3810-193 Aveiro, Portugal; ^3^Nursing School of Coimbra, 3046-851 Coimbra, Portugal; ^4^Coimbra Education School, Polytechnic Institute of Coimbra, 3030-329 Coimbra, Portugal

###### **Correspondence:** Lénia Costa (leniacosta@ua.pt)

**Background**

The nursing assistant (NA) plays an important role in maintaining the health and independency of institutionalized older adults (OA) [1]. These professionals are required to help OA to achieve and maintain their highest level of function. The Function-Focused Care (FFC) is a philosophy of care that promotes the restoration and/or maintenance of physical function. In the institutional context it is relevant empowering NA to adopted this philosophy [2].

**Objective**

This study intends to analyse the perception of NA in relation to the FFC, through scales of self-efficacy, outcomes expectations and knowledge, as well as, the validity and reliability related properties.

**Methods**

Quantitative approach of a descriptive/correlational cross-sectional type. A self-report questionnaire consisting of sociodemographic and professional variables and the scales of self-efficacy, expectations and knowledge were applied. Further details about the scales used can be found in Costa [3]. The validation/reliability procedures for each scale consisted in the calculation of exploratory factor analysis, Cronbach's alpha, and in the intra-class correlation coefficient (ICC) for test/retest purposes. Correlation between the scales themselves, with feelings related to the care of elderly, and sociodemographic and professional variables were tested using the Spearman Rank test.

**Results**

The sample consisted of 73 NA (100% women) with a mean age of 46.4 (± 9.9) years from 5 different institutions. The scale of the self-efficacy showed a three-factor model with the total variance of 73.4%, Cronbach's alpha = 85.2% and ICC = 0.80. The scale of outcomes expectations presented one factor, Cronbach's alpha= 95.2% and ICC = 0.97. The scale of Knowledge obtained a percentage of correct answers only of 44.7%. It was not possible to develop predictive models to relate these scales in a pre-intervention situation. Also, the low correlation between the scales and feelings related to the care of OA (difficulty, gratification, physical overload and emotional overload) or sociodemographic and professional variables (age, years of experience, and self-knowledge), indicated a weak dependence between them. Finally, the institution variable showed not to be a confounding variable (that is, does not influence these results).

**Conclusions**

The Portuguese version of the scales analysed showed satisfactory data validity and reliability. These results suggest that the Portuguese version of these scales can be used to evaluate the FFC performed by the NA. These results point to the importance of implementing a FFC program in the institutions and analyse its impact on AO care and on NA.

**Acknowledgements**

This work was supported in part by the Portuguese Foundation for Science and Technology (FCT-Fundação para a Ciência e a Tecnologia), through CIDMA - Center for Research and Development in Mathematics and Applications, within project UID/MAT/04106/2013.

**References**

1. Gray-Stanley JA, Muramatsu, N. Work stress, burnout, and social and personal resources among direct care workers. Research in Developmental Disabilities. 2011; 32(3), 1065–74. http://doi.org/10.1016/j.ridd.2011.01.025

2. Resnick B, Boltz M, Galik E, Pretzer-Aboff I. Restorative Care Nursing for Older Adults. New York; Eds: Graubard A, Claire L (2nd ed); Springer Publishing Company; 2012

3. Costa, Lénia. Cuidado centrado na funcionalidade: validação das escalas de autoeficácia, expectativas e conhecimento [Master Thesis] [Portuguese]. Universidade de Aveiro. 2016

**Keywords**

Aging, Functionality, Function-focused care, Nursing assistant.

### P32 Determinant factors for the development of student competencies in the context of clinical training: one ecological perspective

#### Marília Rua^1^, Isabel Alarcão^2^, Wilson Abreu^3^

##### ^1^Health School, University ofAveiro, 3810-193 Aveiro, Portugal; ^2^University of Aveiro, 3810-193 Aveiro, Portugal; ^3^Nursing School of Porto, 4200-072 Porto, Portugal

###### **Correspondence:** Marília Rua (mrua@ua.pt)

**Background**

The growing complexity of health care settings, as well as the own care, require that training in this area is also a process thought, a dynamic perspective of integration/implementation of knowledge in each context, which is only possible if carried out in close collaboration between school and a real context of clinical practice [1]. In the light of bio-ecological perspective [2] the development of skills of the students in this context may be influenced by several factors related to the person, the process, context and time.

**Objective**

To understand the factors that influence the development of student’s skills in the clinical training.

**Methods**

We selected a qualitative methodology, using a case study [3] referring to the Nursing Degree, in University of Aveiro. Data emerged from narratives of students and supervisors about their experiences on clinical context.

**Results**

The final results allow us to conclude that the development of abilities occurs in an integrating way, combining synergistically different dimensions and important factors related to the PPCT model. For the Person - emerge the activities, the contact with suffering/death and affective-relational climate. For the Process, the proximal process is pointed out, as well as strategies of supervision. In these contexts, emerge, in the microsystem, the specificities of each context; in the mesosystem the importance goes to the multicontextual participation; in the exosystem, to the interinstitutional relationship and, at a macrosystemic, signs the influence of the policies of hospital management. With respect to time, the importance of the continuity of the proximal processes and the periodicity of the clinical teaching were observed.

**Conclusions**

The student’s skills development is a dynamic, dialectical and progressive process which implies: continuity over time; progressive interaction with people of context–process; contexts that establish themselves as important elements in the development of students' skills at different levels.

**References**

1. Rua M dos S. De aluno a enfermeiro - Desenvolvimento de Competências em Contexto de Ensino Clínico. Loures: Lusociência; 2011.

2. Bronfenbrenner U, Morris P. The Ecology of Developmental Process. In: Pedro JG, editor. Stress and Violence in Childhood and Youth. Lisboa: Faculdade de Medicina, Universidade de Lisboa; 1999. p. 21–96.

3. Yin R. Estudo de Caso. Planejamento e Métodos. 3a. Porto Alegre: Artemed Editora; 2005.

**Keywords**

Bioecological model, Student, Competencies, Clinical training.

### P33 Phytochemical screening from Rosmarinus officinalis and Ginkgo biloba leaf extracts

#### Ana França^1^, Diana Silva^2^, Ana I Oliveira^3^, Rita F Oliveira^3,4^, Cláudia Pinho^3^, Agostinho Cruz^3^

##### ^1^Farmácia Holon, Baguim do Monte, 4435-668 Gondomar, Portugal; ^2^Farmácia Higiénica, Fão, 4740-323 Esposende, Portugal; ^3^Centro de Investigação em Saúde e Ambiente, Escola Superior de Saúde, Instituto Politécnico do Porto, 4200-072 Porto, Portugal; ^4^Secção Autónoma de Ciências da Saúde, Universidade de Aveiro, 3810-193 Aveiro, Portugal

###### **Correspondence:** Ana França (anap981@gmail.com)

**Background**

Currently, drug therapy with oral antidiabetic agents, is capable of inducing normoglycemia levels able to decrease the risk of complications associated with *diabetes mellitus*. However, it is also known that the various existing oral antidiabetic agents may trigger a large number of adverse events, either alone or in combination. Some of these tolerability and security issues related to the oral antidiabetic are reported by patients and can influence negatively or satisfaction with treatment or glycaemic control, or the therapeutic adherence and maintenance. It is therefore very important the role of patients in monitoring of adverse events related to the use of the oral antidiabetic drugs in order to optimize treatment and improve the quality of life of patients with type 2 diabetes (DM2).

**Objective**

The aim of this study is to determine the prevalence of adverse events associated with use of oral antidiabetics and assessing their impact on Health-related Quality of Life (HRQoL) of diabetic patients tracked in primary health care.

**Methods**

A total of 357 DM2 patients were enrolled in observational and cross-sectional study, recruited in six Health Care Centres/Family Health Units (FHU) of the central region of Portugal. Data collection comprised three questionnaires to measure the prevalence of adverse events, the diabetes health profile (DHP-18) and EQ-5D-3L.

**Results**

The results show that the highest prevalence of adverse events is in the DipeptidylPeptidase-4 Inhibitors followed by Metformin+Sitagliptin (fixe dose) and Metformin+Vildagliptin (fixe dose) therapeutic classes. We also found that all the correlations between different variables are statistically significant (p < 0.001).

**Conclusions**

Thus, we conclude that patients who show greater number of adverse events tend to have poorer health profile, worse general health and also lower health related quality of life.

**References**

1. Begum A, Sandhya S, Ali SS, Vinod KR, Reddy S, Banji D. An in-depth review on the medicinal flora Rosmarinus officinalis (lamiaceae). Acta Sci Pol Technol Aliment. 2013;12(1):61–73.

2. European Medicines Agency. European Union herbal monograph on Ginkgo biloba L., folium. 2015.

3. Goh LM, Barlow PJ, Yong CS. Examination of antioxidant activity of Ginkgo biloba leaf infusions. Food Chem. 2003;82:275–82.

4. Kontogianni VG, Tomic G, Nikolic I, Nerantzaki AA, Sayyad N, Stosic-Grujicic S, et al. Phytochemical profile of Rosmarinus officinalis and Salvia officinalis extracts and correlation to their antioxidant and anti-proliferative activity. Food Chem. 2013;136(1):120–9.

**Keywords**

*Rosmarinus officinalis*, *ginkgo biloba*, phytochemical screening, leaf extract.

### P34 Systematic review - how comfort and comfort in nursing are characterized

#### Ana R Sousa^1^, Eliette Castela^2^, Patrícia Pontífice-Sousa^2^, Teresa Silveira

##### ^1^Centro Hospitalar de Setúbal, 2910-446 Setúbal, Portugal; ^2^Universidade Católica Portuguesa, 1640-023, Lisboa, Portugal

###### **Correspondence:** Ana R Sousa (anaritasousa00@gmail.com)

**Background**

Comfort is an important concept and a fundamental value of nursing. This is assumed to be a multidimensional, dynamic and intersubjective concept and the nursing intervention measures used to satisfy the specific comfort needs, thus comforting constitutes a competence of the nurse. Recognizing the importance of scientific evidence in practice, the importance of characterizing and understanding the ways and means of comfort centred on the needs of the client, an exploratory research was carried out with the purpose of knowing the meaning of comfort, as well as ways and forms of comfort, in order to define effective interventions that promote comfort.

**Objective**

To know how is evidenced the characterization of comfort and comfort in the nursing scientific literature.

**Methods**

Systematic review of the literature based on the recommendations of the Joanna Briggs Institute on the PICO strategy and PRISMA recommendations. The research was performed in databases CINAHL Plus, MEDLINE, Nursing & Allied Health Collection and MedicLatina, from January 2010 to November 2017, combining the following descriptors: Comfort * AND Nurs * AND research NOT Psyquiatric.

**Results**

Eleven studies were integrated in the review, which involved people with chronic and acute illness. Studies have shown that being socially accepted, being physically comfortable, feeling safe, being close to significant people are some of the characteristics that qualify comfort. Regarding comfort in nursing, the findings analysed demonstrate numerous comforting strategies, namely, effective and empathetic presence, touch, smile, family integration in the care process, among others.

**Conclusions**

The main results, while providing data that allow us to characterize comfort and comfort in nursing, also highlight the need to investigate the focus.

**Keywords**

Caracterization of the comfort term, Comfort care, Nursing care.

### P35 Rotines of life and health of institutionalized young people

#### Tiago Machado^1^, João Serrano^1^, Sergio Ibanez^2^, Helena Mesquita^1^, Pedro Pires^1^

##### ^1^School of Education, Polytechnic Institute of Castelo Branco, 6000-266 Castelo Branco, Portugal; ^2^Faculty of Sports Science, University of Extramadura, 10003 Cáceres, Spain

###### **Correspondence:** Tiago Machado (tiagomachadix@gmail.com)

**Background**

Today's society creates many limitations in the daily lives of children and young people and will have an impact on their well-being, health and quality of life. This is aggravated when we speak of institutionalized children. **Objective**

To study the leisure activities of young people institutionalized in Homes for Children and Youth and to know if these activities contribute to the development of healthy lifestyles.

**Methods**

A questionnaire was used as instrument, which was submitted to validation by specialists. The questionnaires were completed by the young people with the presence of the researcher. The sample consisted of 100 young people aged between 10 and 18 years old, belonging to 6 Homes for Children and Youth.

**Results**

In the period of free time we found that in the recreations the most accomplished activities were to talk with friends (86.4%), play in the sports fields (47.5%) and dating (23.3%). Most of the young people do not participate in school sports (76.7%). The most accomplished activities after school were: watching TV (100%), cleaning the Institution (96.2%), listening to Music (89.3%), studying (87.4%), playing on the computer and on Facebook (82.6%) and doing physical activity (table tennis, football, among others) (73.8%). Regarding the accomplishment of these activities, the young people reported doing them daily or at least 2 to 3 times a week. When asked if they would like to occupy their free time within the institutions otherwise, the young people were divided, even though the majority responded that they did not (53.4%). As young people are in an open regime, we asked them which activities were most frequently carried out, outside the institution, and they answered that it was a walk with their friends (87.4%), and that the frequency of this activity was daily or at least 2 to 3 times a week. When asked if they would like to take their free time away from institutions in a different way, most of the young people said no, given that their activities are their preference.

**Conclusions**

The results showed that the young people in the study carried out activities considered healthy, which contribute to their quality of life and well-being; however, we were able to verify that they present limitations, namely, in relation to physical activity, since most of the activities performed both inside and outside the institutions had low levels of intensity and frequency, being carried out sporadically.

**Keywords**

Life and health routines, Free Time and Leisure, Children and young people at risk.

### P36 Evaluation of pain in patients intubated orotracheally: BPS and CPOT

#### Ana RPQ Pinheiro^1^, Rita Marques^2^

##### ^1^Instituto de Ciências da Saúde, Universidade Católica Portuguesa, 1649-023 Lisbon, Portugal; ^2^Escola Superior de Saúde da Cruz Vermelha Portuguesa, 1300-906 Lisbon, Portugal

###### **Correspondence:** Ana RPQ Pinheiro (queiroz.anarita@gmail.com)

**Background**

Pain is considered as a symptom of difficult assessment and characterization by health care providers who take care of orotracheal intubated patients (EOT) unable to communicate verbally, so their assessment is critical to an effective management of care and therapy. An EOT patient is exposed to a variety of painful procedures and, if the pain is not controlled, it can lead to multiple complications: physical (cardiovascular, neurological and pulmonary) and psychological (stress, anxiety and delirium), so nurses must have credible instruments for evaluation and monitoring.

**Objective**

This systematic literature review (RSL) aimed to identify the most reliable tool for pain assessment, by analysing the validity and reliability of the BPS and CPOT scales, as well as their ease of application.

**Methods**

Using the methodology recommended by the Cochrane Centre, this RSL was guided by the following research question: “*Which is the most appropriate scale, BPS or CPOT, for pain assessment in patients unable to communicate verbally?*” The seven included studies resulted from a research in EBSCO, using the terms “behavioural pain scale” and “critical care pain observation tool”, with Boolean operator “and”, in full text, published between 2007-2017.

**Results**

With a number of participants between 23 and 117, the 7 selected studies, all with quantitative nature, concluded that both scales are reliable and valid for the assessment of pain in this population [1-5]. [4] report that although BPS is more sensitive in identifying the patient's response, CPOT is a good alternative. It was also verified that both instruments are sensitive to painful procedures, with an increase in several indicators [1, 2, 6]. There was also a significant statistically correlation between the values of arterial tension and the performance of a painful procedure, (the higher the pain value, the higher the arterial tension) [5,6].

**Conclusions**

Both scales (BPS and CPOT) are suitable for the evaluation of pain in EOT patients and according to nurses, both are easy to apply and useful for care delivery [5, 7]. However, the literature does not show the most adequate scale, suggesting that other studies must be done.

**References**

1. Rijkenberg S, Stilma W, Endeman H, Bosman RJ, Straaten HMO. Pain measurement in mechanically ventilated critically ill patients: Behavioral Pain Scale versus Critical-Care Pain Observation Tool. Journal of Critical Care.

2015; 30: p. 167-172.

2. Liu Y, Li L, Herr K. Evaluation of Two Observational Pain Assessment Tools in Chinese Critically Ill Patients. Pain Medicine. 2015; 16: p. 1622-1628.

3. Rahu MA, Grap MJ, Ferguson P, Joseph P, Sherman S, Elswick, Jr RK. Validity and Sensitivity of 6 Pain Scales in Critically Ill, Intubated Adults. American Journal of Critical Care. 2015 Nov; 24(6): p. 514-523.

4. Darwish ZQ, Hamdi R, Fallatah S. Evaluation of Pain Assessment Tools in Patients Receiving Mechanical Ventilation. AACN Advanced Critical Care. 2016 4-6; 27(2): p. 162-172.

5. Vadelka A, Busnelli A, Bonetti L. Comparison between two behavioural scales for the evaluation of pain in critical patients, as related to the state of sedation: an observational study. SCENARIO. 2017; 34(2): p. 4-14.

6. Damström DN, Saboonchi F, Sackey PV, Björling G. A preliminary validation of the Swedish version of the critical-care pain observation tool in adults. Acta Anaesthesiol Scand. 2011; 55: p. 379-386.

7. Fothergill Bourbonnais F, Malone-Tucker S, Dalton-Kischel D. Intensive care nurses’ assessment of pain in patients who are mechanically ventilated: How a pilot study helped to influence practice. Canadian Journal of Critical Care Nursing. 2016; 27(3): p. 24-29.

**Keywords**

Behavioral pain scale, Critical care pain observation tool, Pain rating scales, Nursing.

### P37 Analgesic effect of the topical use of cannabidiol oil in experimental ulcers in the laboratory animal

#### María-del-Carmen Jiménez-Díaz^1^, Carla Jiménez-Rodríguez^1^, María-del-Pino Quintana-Montesdeoca^2^, Juan F Jiménez-Díaz^3^, Francisco J Hernández Martínez^3^

##### ^1^Universidad de Jaén, Universidad de Jaén, 23071 Jaén, España; ^2^Universidad de Las Palmas de Gran Canaria, 35015 Las Palmas de Gran Canaria, España; ^3^Cabildo de Lanzarote, 35500 Lanzarote, Las Palmas, Islas Canarias, España

###### **Correspondence:** María-del-Carmen Jiménez-Díaz (cjimenez@ujaen.es)

**Background**

Since ancient times the use of the *Cannabis sativa L.* plant has been known in topical form for the treatment of haemorrhages, inflammations, oedema, various pains, among others. In fact, tincture and cannabis extract were sold without restriction in European and American pharmacies until the beginning of the 20th century.

**Objective**

To study the analgesic effect of cannabidiol oil (CBD) applied topically to experimental ulcerative skin lesions in the laboratory animal.

**Methods**

Experimental study with control group (with physiological saline to maintain hydration conditions and group with extra virgin olive oil (EVOO) to avoid bias with the oleic excipient), to check the analgesic effect of CBD applied topically on ulcerative lesions. Experimental total skin in the adult male white rat, Sprague Dawley strain. Ten animals were used for each group under standard laboratory conditions. After anaesthesia with 100% isoflurane, a total skin wound was performed in the region of the back with a disposable surgical punch of 8 mm in diameter. They were then distributed in individual cages to prevent them from licking each other and with sufficient height to prevent rubbing of the skin ulcer with the passenger compartment. 0.15 ml of the respective product was applied daily to the ulcers. The analgesic response was assessed by obtaining latency of withdrawal of the tail of the animal under thermal stimulus through the meter LE7106 Tail-flick®, Letica®, previous impregnation of the tail of the animals of the different products used by topical friction and, after waiting for 15 minutes, the tail of each of the animals is subjected to the emission of the infrared beam. Similar action was taken with the rest of the products studied, the EVOO and the CBD. The analgesic response was practiced twice, on different days. The statistical program SPSS 25.0 was used, considering a level of significance of p < 0.05.

**Results**

There was no significant difference between the products applied to the different animals. The highest average corresponded to the application of CBD in observation and measurement 1. In observation and measurement 2, no significant difference was detected and the average values were similar between EVOO and CBD.

**Conclusions**

The most favourable analgesic response was obtained under the influence of cannabidiol oil, which also presents a greater tolerance to pain as experimentation progresses.

**Keywords**

Analgesia, Cannabidiol, Skin, Ulcer, Rat.

### P38 Polypharmacy in elderly patients in a community pharmacy

#### Sónia Lopes^1^, Clara Rocha^2^, Rui Cruz^1^

##### ^1^Pharmacy Department, Coimbra Health School, Polytechnic Institute of Coimbra, 3046-854 Coimbra, Portugal; ^2^Complementary Sciences Department, Coimbra Health School, Polytechnic Institute of Coimbra, 3046-854 Coimbra, Portugal

###### **Correspondence:** Sónia Lopes (soniabelina@gmail.com)

**Background**

The definition of polypharmacy isn’t consensual, but all authors refer it as the simultaneous use, and the chronic way, of several drugs by the same person. Polypharmacy affects mainly elderly and it’s due to the high number of chronic diseases in this population and consequent need to take medications to control them.

**Objective**

Characterization and quantification of polypharmacy in a rural elderly population; identification of the most prescribed pharmacotherapeutic classes; evaluation of connection between polypharmacy and elderly characteristics.

**Methods**

It carried out an observational, retrospective, transversal and analytical study in a Popular Pharmacy (Pombal). 230 individuals aged 65 years old or more were surveyed and the data collection was made through a questionnaire prepared for this purpose. Major polypharmacy was defined as the chronic consumption of at least 5 different drugs.

**Results**

The elderly took, on average, 6.20 ± 2.91 drugs daily. The prevalence of Major polypharmacy was 70.4%. The most prescribed pharmacotherapeutics groups were cardiovascular and Central Nervous System. There were statistically significant differences between age and number of medicaments taken, as well between number of drugs and the way to identify the medication, the knowledge of the therapeutics indications, the occurrence of mistakes or take outside advised time, and the self-perception of health state (p ≤ 0.05).

**Conclusions**

In view of the obtained results, it’s concluded that polypharmacy is very high in the Portuguese population under study. It’s the persons most aged who consume a greater number of drugs. The elderly with less academic qualifications are those who have more difficulty in identifying medication and respective therapeutic indications. It’s necessary to adopt strategies in order to reduce polypharmacy, having both the prescriber and the professionals of pharmacy, a preponderant role in this task.

**Keywords**

Polypharmacy, Elderly, Chronic medication.

### P39 Salivary detection of the topical use of cannabidiol oil in experimental ulceras in the laboratory animal?

#### Bienvenida-Del-Carmen Rodríguez-De-Vera^1^, Carla Jiménez-Rodríguez^1^, María C Jiménez-Díaz^2^, Juan F Jiménez-Díaz^1^, Francisco J Hernández-Martínez^3^

##### ^1^Universidad de Las Palmas de Gran Canaria, 35015 Las Palmas de Gran Canaria, España; ^2^Universidad de Jaén, Universidad de Jaén, 23071 Jaén, España; ^3^Cabildo de Lanzarote, 35500 Lanzarote, Las Palmas, Islas Canarias, España

###### **Correspondence:** Bienvenida-Del-Carmen Rodríguez-De-Vera (bienvenida.rodriguez@ulpgc.es)

**Background**

Cannabidiol (CBD) is a phytocannabinoid that does not refer to experiences contrasted with its topical use in ulcerative lesions and skin wounds.

**Objective**

To determine if the topical application of CBD in ulcers exerts a cumulative effect in the organism of the experimental animal, with undesirable effects at the level of the Central Nervous System (CNS), in a similar way to its general use.

**Methods**

Experimental study applying total skin ulcerative lesions in the adult male white rat, Sprague Dawley strain, with cannabidiol oil to check if it accumulates in the body and can be detected in salivary secretion. Ten animals were used under standard laboratory conditions. After anaesthesia with 100% isoflurane, a total skin wound was performed in the region of the back with a disposable surgical punch of 8 mm in diameter. They were then distributed in individual cages to prevent them from licking each other, with sufficient height to prevent rubbing of the skin ulcer with the passenger compartment. 0.15 ml of cannabidiol oil was applied daily to the ulcers. The study of the body accumulation of the drug object of our study, the cannabidiol oil, was done by qualitative detection of the drug and its metabolites in the saliva of the experimental animal. This type of assay provided a preliminary analytical result using monoclonal Ab to selectively detect high levels of a specific drug which, if positive, would require complementing it with other chemical methods to quantify the accumulation of the product. The test, drogotest®, is made up of spongy collectors (chupa-chups) that, after being soaked for 3 minutes in the mouth of the animal treated with cannabidiol oil, allows to pour its contents by manual compression of the same through a strainer in collecting chamber. Finally, 3 drops of the fluid of the animal's saliva, contained in said collecting chamber, are poured over a detector sample in cassette wells that will show, or not, the presence of the drug in a time period of less than 10 minutes, by observation. direct visual with colour changes of the cassette sample.

**Results**

The detection by the drogotest® test of the general accumulation of cannabis in the organism of the animals studied was negative.

**Conclusions**

The benefit of the topical use of cannabidiol in ulcerative lesions is confirmed without the undesirable effects that cannabis causes in the CNS.

**References**

1. Grotenhermen F, Russo E, Navarrete R (eds). Cannabis y cannabinoides, farmacología, toxicología y potencial terapéutico. Sevilla: Castellarte; 2003.

2. Abanades S, Cabrero-Castel A, Fiz J, Farré M. Farmacología clínica del cannabis. Dolor 2005; 20: 187-98.

3. OMS. Serie Informes Técnicos, nº 478. El uso del Cannabis.

4. Fusar-Poli P et al. Distinct effects of (delta) 9-tetrahydrocannabinol and cannabidiol on neural activation during emotional processing. Arch Gen Psychiatry 2009; 66 (1): 95-105.

5. Pertwee RG. Cannabinoid receptors and pain. Prog Neurobiol 2001; 63: 569-611.

6. Walker JM, Huang SM. Cannabinoid analgesia. Pharmacol Ther 2002; 95: 127-35.

**Keywords**

Detection, Saliva, Cannabidiol, Skin, Ulcer, Rat.

### P40 Humour and nurses’ stress: humour contributions on stress management. A literature systematic review

#### Maria I Santos^1^, Rita Marques^2^

##### ^1^Health Sciences Institute, Catholic University of Portugal, 1649-023 Lisbon, Portugal; ^2^Escola Superior de Saúde da Cruz Vermelha Portuguesa, 1300-125 Lisbon, Portugal

###### Maria I Santos (inescamiller@gmail.com)

**Background**

Nurses experience high levels of work-related stress due to their daily contact with critical situations, suffering, negative emotions and death. This stress overload imposes negative consequences at an individual and at an organizational level, with direct and indirect costs. On the contrary, humour seems to produce benefits to health, job satisfaction and group cohesion, when used in an adaptive way. There is scientific evidence that humour may establish an incisive coping strategy in the management of work-related stress that can be used by nurses for their own benefit.

**Objective**

The aim of the present study is to build a framework of a systematic review on the relationship between humour and nurses’ work-related stress.

**Methods**

Using the Cochrane Centre recommended methodology, this systematic review was guided by the following research question: *What is the contribution of humour to nurses’ stress management?* The research was performed through the EBSCO and Google Scholar search engines, in the bibliographic databases: CINAHL®Complete, MEDLINE Complete, Cochrane Controlled Trials Register, MedicLatina, Pubmed, Scielo and RCAAP, since 2007 to 2017, using the following conjugations of descriptors and Boolean operators: humour (OR humor) AND stress AND nurses (OR nursing) NOT children NOT patients NOT students.

**Results**

A sum of four articles, that respond to our research question, was selected. The empirical studies were developed in four different countries (USA, Canada, UK and Portugal) with diverse designs using either qualitative and quantitative approaches. The sample varied from 15 to 61 nurses. All studies demonstrated that humour expressions are used by nurses to deal with stressful situations [1-4].

**Conclusions**

The use of adaptive forms of humour promote detachment and reassessment of the situation contributing to stress management [3, 4]. It also strengthens interpersonal relationships, improves communication and group cohesion, and contributes to job satisfaction [1, 2]. Therefore, evidence shows that humour can be an effective tool in stress management. Moreover, one study points out that humour can also arise in response to stress, warning to an increased stress level [4]. Nevertheless, the reduced empiric evidence found suggests that this subject within Nursing Science is not yet highly established.

**References**

1. Scott T. Expression of humour by emergency personnel involved in sudden deathwork. Mortality. 2007, 12 (4): 350-364.

2. Dean R, Major J. From critical care to comfort care: the sustaining value of humour. Journal of Clinical Nursing. 2008, 17: 1088-1095.

3. Harris T. Caring and Coping. Exploring How Nurses Manage Workplace Stress. Journal of Hospice & Palliative Nursing. 2013, 15 (8): 446-454.

4. Santos M, José H, Capelas M. O Humor e o Stresse dos Enfermeiros que Cuidam com Pessoas em Fim de Vida. Revista Servir. 2016, 59 (4): 69-74.

**Keywords**

Humor/humour, Stress, Nurses.

### P41 Socio-clinical relationships among nursing students in practice context

#### Laura Reis (laurareis@esenf.pt)

##### Porto Nursing School, 4200-072 Porto, Portugal

**Background**

It is in clinical context that the students attribute greater meaning to their peers. This fact is related to a set of new experiences that are significant for these actors. Supported by the literature consulted, we are led to say that they last even beyond the end of the course, unlike the relations established in the classroom.

**Objective**

Analyse the relationships established among students over two consecutive clinical studies.

**Methods**

The study was carried out with students from a group of the 2nd year of a Nursing Degree who were experiencing their first clinical experience in a hospital context, namely 10 weeks in an internal medicine service and 10 weeks in a surgery service. We chose an ethnographic study within the framework of the qualitative paradigm, in a longitudinal approach according to the logic of the case study. As a data collection technique, we used participant observation and semi-structured interviews.

**Results**

We verified that the relation established between students differed according to whether it was a first or a second clinical teaching. The knowledge that the group acquired about themselves and about the methodologies adopted by the tutors, was different in the different contexts. In the first medical school (medicine), despite the little knowledge that the students had among themselves, the established relationship was very cooperative and cordial, based on the spirit of help. This was due to a number of factors, namely, a first clinical experience, leading to a high need to share information and knowledge related to the context and clinical practices; the lack of knowledge about tutors; and the lack of safety in the care of patients. The relationships established in this space were, therefore, strong and cohesive. In the clinical teaching of surgery, the relation established was different, highlighting more divergences/heterogeneities. According to the students' opinion, this fact was related to the work methodology/distribution of students and the request of academic work by the teachers.

**Conclusions**

We verified that on the 2nd clinical teaching, the relationships established between students were influenced by interpersonal competition logics. As it is known, the need to form more restricted groups can lead to unleashing alliance dynamics and oppositions among students, giving rise to behaviours of competitiveness, or of helping others, or even to the development of individualized work. Another aspect that seems to have been strongly conditioning, were the methodological strategies adopted by the tutors of the different clinical contexts.

**Keywords**

Clinical supervision, Clinical teaching, Nursing students, Socio-clinical relations.

### P42 Stress management in under graduation nursing students

#### Marco Oliveira, Andreia Santos, Rafaela Barbosa, Diana Portovedo, Isabel Oliveira

##### Escola Superior de Enfermagem da Cruz Vermelha Portuguesa, 3720-126 Oliveira de Azeméis, Portugal

###### **Correspondence:** Isabel Oliveira (ijoliveira@sapo.pt)

**Background**

Admission to higher education is seen by some students as an opportunity of growth, to explore new environments and build new relationships yet, other students, perceive it as potentially anxiogenic. Some of the stressful factors are: examinations, clinical teaching, academic results, a competitive environment and the experience of transition and adaptation to a new academic environment. Therefore, it is necessary to work on the causes of stress and promote coping strategies. Being such an important subject for under graduation students, that experiencing it so intensely, it is crucial that students are also an active part in promoting the efficient management of stress.

**Methods**

Thus, in order to contribute to the adoption of coping strategies and promote the efficient management of stress, a participatory action research in health was developed in a nursing school. The strategies were: the implementation of a student support line (peer support); monthly meetings, addressed to the student population, on topics related to stress and its management; tutorials for 1st year undergraduate students by 3rd and 4th year students, through a structured program of integration to the nursing school. The follow-up of the effectiveness of the activities was carried out through a questionnaire measuring stress levels. This research foresees an initial evaluation (before implementation), intermediate evaluations and a final one, to measure the achievement of the objectives initially stated.

**Results**

The preliminary findings show a mean of 8.52 and a standard deviation of 2.73 in the score of the domain Sleep/Stress of the Portuguese version of the Fantastic Lifestyle Assessment.

**Conclusions**

With the implementation of this participatory action research it is expected a reduction of stress levels, as well as enabling students to adopt coping strategies in order to manage their stress. On the other hand, it will allow a better integration of students, as well as a better academic development, both in theoretical evaluations and in clinical teaching, consolidating the relational skills, which are so important during the course of clinical teaching and later on in their professional life.

**Keywords**

Nursing students, Stress, Coping skills, Action research, Participatory.

### P43 A contribution to the validation of the volume - viscosity swallow test (V-VST) – Portuguese version

#### Catarina Camões^1^, Marília Dourado^1^, Maria A Matos^2^

##### ^1^Faculty of Medicine, University of Coimbra, 3004-504 Coimbra, Portugal; ^2^School of Health, University of Aveiro, 3810-193 Aveiro, Portugal

###### **Correspondence:** Marília Dourado (mdourado@fmed.uc.pt)

**Background**

The prevalence of swallowing disorders after stroke is well described in the literature [1,2]. The early identification of these alterations resorting to non-invasive and easily administered instruments can minimize its consequences and reduce comorbidity and mortality among these patients [1,4]. The V-VST exhibits good psychometric properties, allowing the early identification of patients at risk of developing respiratory and nutritional complications. Its use also allows dietary preventive recommendations to patients until diagnosis confirmation, by instrumental examinations [2].

**Objective**

The goal of this study is to contribute to the validation of the V-VST –Portuguese version, in patients with subacute stroke.

**Methods**

In phase I, the V-VST- Portuguese version [3], as well as its instructions, was presented to a panel of experts constituted by six speech and language therapists, in order to assess its content validity. In phase II, after ethical approval, it was applied to thirty-three patients with subacute stroke, to analyse its psychometric properties, namely its internal consistency and reliability (inter and intra raters). Criterion validity was assessed through the simultaneous application of the 3Oz wst. Collected data were analysed with IBM SPSS version 24.0. Intraclass Correlation, Cronbach’s Alfa and Cohen's Kappa values were calculated.

**Results**

Results of phase I demonstrate a very good agreement between all members of the panel of experts as for constituent items of the V-VST (I-CVI/Ave = 0.95) as well as to its instructions (I-CVI = 0.83). Preliminary results of phase II, showed that the V-VST presents very good intraclass (ICC=0.816) and interclass correlation coefficients (ICC = 0.837). Values obtained from the comparison between the V-VST and 3Oz wst have given similar results (I- CVI = 0.83).

**Conclusions**

The V-VST - Portuguese version seems to be a valid, reliable and practical tool for assessing dysphagia in patients with subacute stroke. Further studies need to be done in the future.

**Acknowledgements**

We would like to thank to the Nutricia Portugal, to the Faculty of Pharmacy of the University of Coimbra as well as to all patients and members of the neurology C service of the Hospitalar and Universitary Center of Coimbra by all the support and availability showed during this study.

**References**

1. Belafsky PC, Mouadeb DA, Rees CJ, Pryor JC, Postma GN, Allen J, Leonard RJ. Validity and Reliability of the Eating Assessment Toll (EAT-10). Ann Otol Rhinol Laryngol. 2008;117(12):919-924.

2. Clavé P, Arreola V, Romea M, Medina L, Palomera E, Prat MS. Accuracy of the volume - viscosity swallow test for clinical screening of oropharyngeal dysphagia and aspiration. Clin Nutr. 2008 Dec;27(6):806-815.

3. Nogueira DS, Ferreira PS, Reis EA, Lopes IS. Measuring Outcomes for Dysphagia: Validity and Reliability of the European Portuguese Eating Assessment Tool (P-EAT-10). Dysphagia. 2015;30(5):511-520.

**Keywords**

Deglution disorders, Bedside examination, Dysphagia, Aspiration.

### P44 Strategies to improve hand hygiene practices: an integrative literature review

#### Ana C Mestre, Filipa Veludo, Susana Freitas

##### School of Nursing, Institute of Health Sciences, Universidade Católica Portuguesa, 1649-023 Lisbon, Portugal

###### **Correspondence:** Ana C Mestre (catarina-mestre@hotmail.com)

**Background**

Healthcare-associated infections (HAIs) are a global concern and pose a real threat to patient safety. Many of them preventable [1]. Knowing that hands of healthcare professionals are one of the main vehicles in the transmission of microorganisms, hand hygiene (HH) is recognized as the easier and most effective measure to prevent and reduce HAIs [2]. However, despite all evidence available and although 98% of healthcare professionals consider HH as the most important basic precaution in preventing HAIs, compliance is poor, remaining less than 40% [3,4].

**Objective**

To identify, in Literature, the most effective strategies to promote HH compliance.

**Methods**

An integrative review between September and October 2017 was fulfilled with the Boolean strategy: [(TI Title) hand hygiene AND (AB Abstract) nurse AND (AB Abstract) infection AND (AB Abstract) strategy OR compliance OR adherence] in CINAHL®, Science Direct and Academic Search Complete. A total of 396 articles were identified, initially. After applying the inclusion criteria: primary and secondary studies with a qualitative and quantitative approach available in full text in Portuguese, English, French and Spanish; and exclusion criteria: studies published before 2016, a sample of 12 articles was included for analysis.

**Results**

From a total of 12 articles analysed, 10 showed the importance of a multimodal approach to the improvement of HH practices with consequent increase in compliance to this behaviour. It stands out the combination of interventions addressing knowledge (education), awareness, context of action (reminders in the workplace) as well as the involvement and support of leaders and managers in building an institutional safety culture (social influence) as the most effective to ensure greater compliance to HH.

**Conclusions**

In order to improve HH practices and, consequently, adherence to this behaviour, the adoption of a multimodal strategy proved to be more successful when compared to single interventions. At an early stage, it is essential to understand the reasons that lead to non-adherence to HH and after that design interventions based on identified barriers. The approach should be global, including not only healthcare professionals but also leaders and managers.

**References**

1. WORLD HEALTH ORGANIZATION. WHO Guidelines on Hand Hygiene in Health Care. 2009. Accessed 20-11-2017. Available in http://apps.who.int/iris/bitstream/10665/44102/1/9789241597906_eng.pdf

2. DIREÇÃO-GERAL DA SAÚDE- Circular Normativa nº 13/DQS/DSD de 14/06/2010 (2010). Orientação de Boa Prática para a Higiene das Mãos nas Unidades de Saúde. Lisboa: Direção Geral de Saúde. Acccessed 20-11-2017. Available in: https://www.dgs.pt/directrizes-da-dgs/normas-e-circulares-normativas/circular-normativa-n-13dqsdsd-de-14062010.aspx

3. Piras SE, Lauderdale J, Minnick A. An elicitation study of critical care nurses’ salient hand hygiene beliefs. Intensive and Critical Care Nursing. 2017;42:10–16.

4. Farhoudi F, Sanaei Dashti A, Hoshangi Davani M, Ghalebi N, Sajadi G, Taghizadeh R. Impact of WHO Hand Hygiene Improvement Program Implementation: A Quasi-Experimental Trial. Biomed Res Int. 2016;2016:7026169.

**Keywords**

Hand hygiene, Healthcare-associated infections, Multimodal strategy, Integrative literature review.

### P45 Conception and implementation of a nursing intervention program for family caregivers

#### Ricardo Melo^1,2^, Marília Rua^2^, Célia Santos^3^

##### ^1^Centro Hospitalar de Gaia/Espinho, 4400-129 Gaia, Portugal; ^2^Escola Superior de Saúde, Universidade de Aveiro, 3810-193 Aveiro, Portugal; ^3^Escola Superior de Enfermagem do Porto, 4200-072 Porto, Portugal

###### **Correspondence:** Ricardo Melo (rmcmelo@hotmail.com)

**Background**

The increase of longevity of people and prevalence of diseases resulting in situations of dependency [1], emerge a greater need for supportive care to meet the needs expressed [2]. Family caregivers are very important elements in caring for the family member with self-care dependency, at the home context [3, 4]. This is an exhausting process with serious consequences for the general state of health perceived by the caregiver, as well as for the manifested burden [3, 5, 6]. A structured and contextualized intervention program [7] aimed at the qualification and support of family caregivers is essential for the transition and adequate performance of the functions inherent in this role.

**Objective**

To develop and implement a Nursing Intervention Program with family caregivers of dependent persons, in a home context.

**Methods**

This process began with an integrative review of the literature, in order to discover the main needs evidenced by family caregivers. Electronic databases were used, namely EBSCO and B-on, with the following descriptors: Caregiver; Family Caregivers; Needs; Dependent. The second stage corresponded to the adaptation of the Intervention Program, with the use of the Delphi technique on a group of experts. The last phase corresponded to a quasi-experimental study, with pre- and post-intervention evaluation, with the implementation of the program on 70 family caregivers, using home visits.

**Results**

With the review of the literature were obtained 21 articles (ten quantitative studies, five qualitative studies, four systematic reviews of the literature, a review of the literature and a mixed study). The evidenced needs were organized by the Transition Theory: community and social resources; knowledge and preparation; personal meaning, beliefs and attitudes; and socioeconomic condition. The consensus technique allowed the structuring of a Nursing Intervention Program, with 93 interventions, divided in emotional and instrumental support. The implementation of the Intervention Program implied, on average, 6 home visits to the caregivers, emotional support provision and caregiver training.

**Conclusions**

A Nursing Intervention Program, with structured and contextualized interventions in the home context, with family caregivers of dependent persons, is a facilitator for the transition experienced by caregivers, but also an important instrument of the work developed by nurses. Thus, it provides the necessary emotional support and skills that enable caregivers to optimize care delivery.

**References**

1. INE - Instituto Nacional de Estatística IP. Censos 2011 Resultados Definitivos - Portugal. XV recenseamento geral da população; V recenseamento geral da habitação. Lisboa: Instituto Nacional de Estatística; 2012.

2. Figueiredo D. Cuidados Familiares ao Idoso Dependente. Lisboa: Climepsi Editores; 2007.

3. Imaginário C. O Idoso Dependente em Contexto Familiar: Uma Análise da Visão da Família e do Cuidador Principal. 2ª ed. Coimbra: Formasau; 2008.

4. Marques SCL. Os Cuidadores Indormais de Doentes com AVC Coimbra: Formasau - Formação e Saúde Lda.; 2007 Janeiro de 2007.

5. Martins T. Acidente Vascular Cerebral: Qualidade de Vida e bem-estar dos doentes e familiares cuidadores. Coimbra: Formasau – Formação e Saúde Lda.; 2006.

6. Sequeira C. Cuidar de Idosos com Dependência Física e Mental. Lisboa: Lidel; 2010.

7. ICN. CIPE Versão 2 - Classificação Internacional para Prática de Enfermagem Lisboa: International Council of Nurses; 2011.

**Keywords**

Family caregivers, Intervention program, Transition, Needs, Dependency.

### P46 Antimicrobial activity of natural extracts and commercial elixirs in oral pathogens

#### Maria J Alves, Marta Pereira, Sara Fraga, Isabel Ferreira, Maria I Dias

##### Centro de Investigação de Montanha, Instituto Politécnico de Bragança, 5300-253 Bragança, Portugal

###### **Correspondence:** Maria J Alves (maria.alves@ipb.pt)

**Background**

Although *Streptococcus mutans* has been responsible for decades as the etiological agent of dental caries, recent evidence indicates a high prevalence for *S. mutans* in dental biofilms where *Candida albicans* resides; which suggests that the interaction between these two species may mediate cariogenic development [1].

**Objective**

To evaluate the antimicrobial activity of three chemical elixirs of different commercial brands and two aqueous extracts obtained from plants (*Chamomilla recutita L.* and *Foeniculum vulgare Mill*.) in *C. albicans* and *S. mutans.*

**Methods**

Percent growth inhibition was quantified by measurement of optical density (OD) at 600 nm in a microplate reader.

**Results**

Both the extracts and the elixirs presented antimicrobial activity for the two microorganisms previously mentioned. Among the elixirs tested, the one with the highest antimicrobial activity for *S. mutans* was Colgate (100%), followed by Eludril and a white brand (≥ 99%). For *C. albicans*, the Eludril (100%) gave the highest activity, followed by Colgate (99%). A *Chamomilla recutita* extract (10 mg/ml) showed an inhibition percentage of growth of 96% for *S. mutans* very similar to that of the antibiotic (97%). The inhibition percentage of growth decreased for *C. albicans* (87%), although was higher than the antifungal fluconazole (84%).

**Conclusions**

The two extracts showed less antimicrobial activity compared to the elixirs, however, they had higher percentages of inhibition growth than the drugs tested for both microorganisms. The extract of *Chamomilla recutita* was the one that presented the highest antimicrobial activity for the two microorganisms tested comparatively with *Foeniculum vulgare*.

**References**

1. Metwalli KH, Khan SA, Krom BP, Jabra-Rizk MA. Streptococcus mutans, Candida albicans, and the Human Mouth: A Sticky Situation. PLOS Pathogens. 2013;9(10):1-5.

**Keywords**

Oral biofilm, Antimicrobial activity, Elixir, *Foeniculum vulgare mill*, *Chamomilla recutita L*.

### P47 The effects of water walking on body composition – a study with children between 6 and 12 years old

#### Samuel Honório^1,2^, João Oliveira^3^, Marco Batista^1,2^, João Serrano^1,4^, Jorge Santos^3^, Rui Paulo^1,2^, Pedro Duarte-Mendes^1,2^

##### ^1^Department of Sports and Well-being, Polytechnic Institute of Castelo Branco, 6000-084 Castelo Branco, Portugal; ^2^Research on Education and Community Intervention, 4411-801 Arcozelo, Vila Nova de Gaia, Portugal; ^2^Polytechnic Institute of Castelo Branco, 6000-084 Castelo Branco, Portugal; ^4^Centre for the Study of Education, Technologies and Health, Polytechnic Institute of Viseu, 3504-510 Viseu, Portugal

###### **Correspondence:** Samuel Honório (samuelhonorio@hotmail.com)

**Background**

Aquatic activities have been recommended as frequent practice, due to the physical properties of water, especially because fluctuation and reduction of joint impact, with improvements in the body composition of children.

**Objective**

The present research aims to verify if there are differences in body composition in children aged between 6 and 12 years who practice swimming, complemented with water walking at the end of each session and those who only practice swimming.

**Methods**

The sample consisted of 28 individuals aged 6 to 12 years and was divided into two groups: swimming group (SG) with 9 children and a swimming complemented with water walking group (SWWG) of 19 children. In this study, of twelve weeks with three moments of evaluation, with two sessions per week of 45 minutes each, we wanted to identify the benefits in body composition (weight, muscle mass, fat mass, body water, BMI, waist circumference and body percentiles). For that purpose, we used a bio-impedance scale Targa Z29777A, and an anthropometric tape to measure the waist circumference. The water walking activity occurred at the end of each session for 6 minutes, performed in straight line with the water level at the children’s chest. In terms of statistical procedures, we used the program Statistical Package for the Social Sciences version number 20 (SPSS 20.0). We used descriptive statistics (minimum, maximum, means and standard deviations), the Shapiro Wilk test for testing the normality of the sample, inferential statistics (non-parametric Mann-Whitney tests, Friedman's ANOVA, and for the calculation of the magnitude of the effect, the d-Cohen test).

**Results**

After data treatment, regarding the inter-group analysis (comparison between the swimming group and the swimming group with water walking) we observed that there were significant differences in the weight variable, that is, at the end of the 3 moments. Concerning intra-group differences (improvements in the swimming group and in the swimming with water walking group, in the three moments evaluated), the SWWG showed significant improvements in the variables of weight as well, muscle mass, fat mass, body water, body mass index (BMI) and body percentiles.

**Conclusions**

We have concluded that the practice of activities such as swimming and water walking has benefits in the analysed variables and that there are differences in the groups analysed; however, the two activities complemented (swimming and water walking) present much more significant improvements.

**Trial Registration**

NCT03519620

**Acknowledgements**

This work was supported by the Portuguese Foundation for Science and Technology (FCT; Grant Pest – OE/CED/UI4016/2016).

**Keywords**

Children, Water Walking, Swimming, Body composition, Bio-impedance.

### P48 Health literacy level of students at the time of enrolment to health courses in higher education

#### Luis S Luis^1,2^, Victor Assunção^3^, Henrique Luis^4^, Helena Melo^5^

##### ^1^School of Health Science, Polytechnic Institute of Leiria, 2411-901 Leiria, Portugal; ^2^Center for Innovative Care and Health Technology, Polytechnic Institute of Leiria, 2411-901 Leiria, Portugal; ^3^Escola Superior de Saúde, Instituto Politécnico de Portalegre, 7300-074 Portalegre, Portugal; ^4^Faculdade de Medicina Dentária, Universidade de Lisboa, 1600-003 Lisboa, Portugal; ^5^Escola Superior de Saúde Ribeiro Sanches, 1950-396 Lisboa, Portugal

###### **Correspondence:** Luis S Luis (luis.luis@ipleiria.pt)

**Background**

A good level of Health literacy is essential for effective communication between health professionals and patients. Health professionals training should provide them with a set of skills in this area that would allow them to communicate successfully with other health professionals and patients.

**Objective**

The goal of this study was to identify what level of health literacy students have when enrolled at the Universities and Polytechnic Institutes. Students from Nursing Degree courses (Portalegre School of Health Sciences and Ribeiro Sanches Health School) and from the Dental Hygiene Degree (Faculty of Dental Medicine of Lisbon and Portalegre School of Health Sciences), had their health literacy level assessed at enrolment, in the year of 2013/2014.

**Methods**

To evaluate the level of health literacy, the Portuguese version of the NVS (New Vital Sign) was administered to 42 dental hygiene students (6 males and 36 females) and 53 nursing students (14 males and 39 females).

**Results**

The total sample, composed of 95 students, was analysed for provenance to higher education level degree, it was verified that 83.5% came from high school, 6.6% entered by the process of “Maiores de 23”, and 9.9% through other enrolment processes. When analysing these data, by degree, most of the students at the Dental Hygiene degree (92.9%), accessed higher education through high school completion. This value was of 75.5% for Nursing students. Regarding the level of health literacy, it was observed that 72.63% of the students had high level of health literacy, 24.21% moderate health literacy level and 3.16% low health literacy level. There was a statistically significant difference between Nursing and Dental Hygiene students, with the former having a better health literacy (p = 0.007). At the time of entry to the University and Polytechnic, and when considering courses, collected data shows that, for the Degree in Dental Hygiene, 4.8% of the students had a low level of health literacy, 21.4% present a moderate level and 73.8% a high level of health literacy. When considering the Nursing Degree, these values were, respectively, 1.9%, 26.4% and 71.7%.

**Conclusions**

Students access higher education with a level of health literacy appropriate to the courses they intend to attend. The presented results are preliminary and part of a longitudinal study lasting three academic years, evaluating the evolution of health literacy levels of students throughout their training.

**Keywords:** Health literacy, Nursing School, Dental Hygiene School, Students.

### P49 Nursing caring of vulnerable patients in emergency situations: what does the evidence say?

#### Marta Pacheco, Maria T Leal

##### Escola Superior de Enfermagem de Lisboa, 1600-190 Lisboa, Portugal

###### **Correspondence:** Marta Pacheco (mpacheco@campus.esel.pt)

**Background**

Critically ill patient’s (CIP) nursing care quality has improved significantly in the last years because of important technological improvements. In pre-hospital, emergency and intensive care settings, they contribute to the valuation of technical competencies over relational aspects. The emergent need to stabilize the CIP diverts the attention away from the psychological and emotional support, increasing anxiety and diminishing the patient’s cooperation, making the care experience increasingly hostile. Vulnerability arises in these settings [1] as a condition to be appreciated in a “harmonious conciliation between the technological expertise and the art of caring” [2], because it improves expectations and experiences, and increases the positive outcomes of the resuscitation process, giving greater visibility to the nurses’ work. To understand the experiences of patients who lived emergency situations, as well as nursing interventions that reduce vulnerability, will allow for better care of the CIP [3].

**Objective**

Our goal is to review the available evidence regarding nursing interventions that reduce vulnerability at emergency and resuscitation settings.

**Methods**

We performed an integrative review of the literature available from MEDLINE and CINAHL databases and grey literature, to answer the question: *“Which nursing interventions promote the reduction of the CIP’s vulnerability in emergency setting?*” [4].

**Results**

Nine qualitative and quantitative studies satisfied the search criteria and were included. These studies are from different countries and cultures and their analysis identified both the emergency setting and nursing interventions as elements that influence the CIP’s subjective experience of vulnerability. Vulnerability is a permanently present condition in the human being and the recognition of the mutual vulnerability by nurses is a way of preserving the value of caring.

**Conclusions**

The experience of CIP in emergency and resuscitation settings is influenced by organizational, environmental and caring factors. Being cared by competent professionals [5,6], using a therapeutic relationship and responding to the patient main needs are the most valued aspects that convey safety and a significant decrease in vulnerability. Communication and empathy [6], with explanation of clinical procedures, allows the development of trust and reduces vulnerability, facilitating patient collaboration in the care provided. Recognizing the vulnerability of CIP and its influence on collaboration, recovery and satisfaction in care, allows developing strategies and abolishing mechanized and merely technical behaviours. Vulnerability is a continually present condition in humans and the recognition of vulnerability of the professionals themselves [7] is a way to socially preserve the value of caring in an emergency context.

**References**

1. Mitchell M. General anaesthesia and day-case patient anxiety. J Adv Nurs. 2010;66(5):1059–1071.

2. Sá F, Botelho M, Henriques M. Cuidar da Família da Pessoa em Situação Crítica: A Experiência do Enfermeiro. Pensar Enferm. 2015;19(1):31–46.

3. Paavilainen E, Salminen-Tuomaala M, Kurikka S, Paussu P. Experiences of counselling in the emergency department during the waiting period: importance of family participation. J Clin Nurs. 2009;18(15):2217–2224.

4. The Joanna Briggs Institute. Joanna Briggs Institute Reviewers’ Manual: 2014 Edition [Internet]. Adelaide: The Joanna Briggs Institute; 2014. 197 p.

5. Baldursdottir G, Jonsdottir H. The importance of nurse caring behaviors as perceived by patients receiving care at an emergency department. Hear Lung. 2002;31(1):67–75.

6. Wiman E, Wikblad K, Idvall E. Trauma patients’ encounters with the team in the emergency department: a qualitative study. Int J Nurs Stud. 2007;44(5):714–22.

7. Malone RE. Dimensions of vulnerability in emergency nurses’ narratives. Adv Nurs Sci. 2000;23(1):1–11.

**Keywords**

Vulnerability, Critically ill patient, Nursing care, Emergency department.

### P50 Sedentary behavior of older people above 75: where, when and with whom

#### Marta Gameiro, Madalena G Silva

##### Physiotherapy Department, School of Health, Polytechnic Institute of Setúbal, 2910-761 Setúbal, Portugal

###### **Correspondence:** Marta Gameiro (marta.gameiro93@gmail.com)

**Background**

Sedentary behaviours are understood as activities with a low energy expenditure (<1.5 metabolic equivalent - METs) [1]. Even for those who comply with physical activity recommendations, prolonged time in sedentary behaviours is associated with an increased risk of chronic and cardiovascular diseases; decreased muscle mass, strength and power; decreased functional capacity and premature death in elderly people [2]. Older people spend long periods of time in sedentary behaviour [3]. A deeper understanding of what activities represent these behaviours, when and with whom they take place, becomes extremely relevant for health professionals who need to promote a reduction in sedentary time, among very older people in order to enhance health benefits.

**Objective**

To characterize the time spent in sedentary behaviour of older people in three urban day-care centres, as to the duration, type of activities, with whom and where they take place.

**Methods**

A cross sectional study was conducted with 54 participants, average age of 84.53 ± 5.35. An Activity Diary was used to characterize sedentary activity. Each activity noted in the diary was classified in METs, per the Compendium of Physical Activities: Classification of Energy Costs of Human Physical Activities. They were then clustered in four groups: sedentary activities; light intensity; moderate intensity and vigorous intensity activities. Time spent in activities within each group was summed, resulting in the total sum of sedentary, light, moderate, and vigorous intensity activities (min.week^-1^). Relative and absolute frequencies, as well as mean/standard deviation, were used, to characterize sedentary behaviour.

**Results**

Our sample was mainly female (88.9%), widowers (70.4%), living alone (64.8%) and with a low educational background (61.1%). On a regular week, they spent an average of 6h20 min (2608.15 ± 930.67 min.week^-1^) in sedentary behaviour. Half of this time is spent watching TV (50.2%), alone, in the afternoon and evening. Other activities are talking (11.73%), reading (6.7%), resting (6.4%) and playing board games (4.7%).

**Conclusions**

We conclude that our sample spent an average of 6h20min per day in sedentary activity, mainly in the afternoon and evening, watching television alone. In order to break sedentary patterns, clinical interventions need to tackle this particular period of the day, finding alternative strategies for a greater energy expenditure for very old people.

**References**

1. Ainsworth BE, Haskell WL, Whitt MC, Irwin ML, Swartz ANNM, Strath SJ, et al. Compendium of Physical Activities: an MET intensities. Med Sci Sport Exerc. 2000;32(9):498–504.

2. Dunlop DD, Song J, Arntson EK, Semanik PA, Lee J, Chang RW, et al. Sedentary time in U.S. older adults associated with disability in activities of daily living independent of physical activity Dorothy. J Phys Act Heal. 2015;12(1):93–101.

3. Leask CF, Harvey JA, Skelton DA, Chastin SF. Exploring the context of sedentarybehaviour in older adults (what, where, why, when and with whom). Eur Rev Aging Phys Act. 2015;12(1):4.

**Keywords**

Sedentary behavior, Context, Very old people.

### P51 Women’s perception on the role of family nurse in the transition to motherhood

#### Andreia Ferreira, João Simões, Helena Loureiro

##### Escola Superior Saúde, Universidade de Aveiro, 3810-193 Aveiro, Portugal

###### **Correspondence:** Andreia Ferreira (aloferreira84@gmail.com)

**Background**

The study carried out has as its title “*Women's perception on the role of family nurse in the transition to motherhood*” and aims to understand the experiences of puerperal women in relation to the role of the family nurse, in promoting the transition to maternity, in the year 2016/2017, at USF Rainha Tereza.

**Methods**

The accomplishment of the empirical study was authorized by the ARS Centro Ethics Committee. It is part of a paradigm of qualitative nature, based on a descriptive phenomenological methodology, that intends to answer the research question: *What is the perception of puerperal women regarding the interventions of the family nurse in the transition to motherhood?* Eleven mothers for the first time, who were selected in a non-probabilistic manner and for convenience, according to previously established inclusion and exclusion criteria, participated in this study. Once the informed consent of all the participants was obtained, the information collection was obtained through the filling of a questionnaire of sociodemographic characterization and the conduction of a semi-structured interview, recorded in an audiographic record, was later transcribed and analysed by the webQDA program, through the Bardin content analysis technique.

**Results**

Evidence has led to the conclusion that motherhood, as a life cycle transition, is approached by the family nurse in a fragmented way. Women perceive that this health professional has an intervening role in the processes of “Being Woman” and “Being Mother”, but as far as “Being Wife”, the intervention still remains little exiguous. Nevertheless, it became clear that family nurses assume a primordial function in this transition and that this is essentially related to the role of health promoter attributed to it, by mothers for the first time.

**Conclusions**

In conclusion, the traineeship report sensitized the importance of relational skills in the delivery of care and the need to provide care in a holistic way, integrating the family as a partner in the perinatal follow-up.

**Keywords**

Women's Health, Maternal Health, Motherhood, Transition, Nursing.

### P52 Self-perception of health status and physical condition of elderly people practitioners of hydrogymnastics

#### Carlos Farinha^1^, João Serrano^2^, José P Ferreira^1^, João Petrica^2^, Rui Paulo^3^, Pedro Duarte-Mendes^2^, Marco Batista^2^

##### ^1^Faculty of Sport Sciences and Physical Education, University of Coimbra, 3040-256 Coimbra, Portugal; ^2^Higher School of Education, Polytechnic Institute of Castelo Branco, 6000-266 Castelo Branco, Portugal; ^3^Sport, Health and Exercise Research Unit, Polytechnic Institute of Castelo Branco, 6000-266 Castelo Branco, Portugal

###### **Correspondence:** Carlos Farinha (cajo596@gmail.com)

**Background**

The researcher interest in self-perception of health status and physical condition of the elderly is determinant in an increasingly aging society and where one should seek to improve their quality of life.

**Objective**

To study the self-perception of health status and the impact of a physical activity program oriented during 4 months among an elderly population of practitioners of hydrogymnastics.

**Methods**

As instruments were used the questionnaire “mos short health survey – 36 items version 2 (SF-36)” translated and validated for the Portuguese version by Ferreira (2000), and the battery of Functional Tests Fitness Test. Questionnaires were filled by the elderly with the presence of the investigator and the physical fitness was evaluated following the protocol of proof. The sample was constituted by 83 elderly individuals over 55 years of age. In statistical terms we used a percentage analysis in the questionnaire and in the evaluation of the physical condition after application of the Kolmogorov-Smirnov test, we used the non-parametric test for two paired variables, Wilcoxon.

**Results**

According to the different dimensions studied (physical function, physical performance, pain, general health, vitality, social function and emotional performance), we found that the majority of the elderly presents limitations mainly in the most demanding tasks (lift weights, running, walk fast, more intense housework, among others); and that their state of health interfered in the performance of some daily activities, but not in the time spend in performing these same activities. The pains have not been strong in the last times and so, they consider their general health moderate and stable, interfering little in the development of their relationship and social activities. The elderly also said that they feel most of the time calm and tranquil and therefore they feel happy. As for physical fitness, there were improvements in the results in practically all the tests, between the 1st and 2nd evaluation, which showed a positive impact of the work developed during the 4 months.

**Conclusions**

Based on the results, we can conclude that the elderly of the study evidenced a perception of their health condition considered positive and that fits in the normal parameters for their age. The results of the physical activity program revealed improvements in physical fitness levels, between the beginning and the end of the program, aspect that is determinant for the health and autonomy of the elderly, as the investigation reveals.

**Keywords**

Gerontomotricity, Physical activity and health, Physical condition, Hydrogymnastics.

### P53 Impact of dual-task on older adults’ gait spatiotemporal parameters

#### Nádia Augusto^1^, Rodrigo Martins^2^, Madalena G Silva^1^, Ricardo Matias^1,3^

##### ^1^Physiotherapy Department, School of Health, Polytechnic Institute of Setúbal, 2910-761 Setubal, Portugal; ^2^Red Cross School of Health, 1300-125 Lisbon, Portugal; ^3^Champalimaud Centre for the Unknown, 1400-038 Lisbon, Portugal

###### **Correspondence:** Nádia Augusto (naa.fisio.augusto@gmail.com)

**Background**

The ageing process induces changes in gait, particularly in its automatic features [1]. Early detection of gait changes can be a key to ensure timely clinical intervention to prevent falls [2], since most of these occur while walking in dual-task situations [3]. Several studies using 3D kinematic analysis systems have suggested that changes in spatiotemporal parameters can be measured before these changes are noticeable [4,5]. However, their use has been confined to laboratories [6], which is difficult to transfer into clinical practice [7].

**Objective**

To study the effects of the introduction of a dual task, in spatial/temporal parameters, on the gait of older adults, with an ambulatory 3D kinematic analysis system.

**Methods**

An exploratory observational study was performed. Fifteen healthy older adults (age= 75.73 ± 6.03 years old) were recruited from a day centre. All participants were instructed to walk 10 meters in a single self-selected pace, and in a dual task (a verbal fluency task and an arithmetic task) condition. Seven spatiotemporal gait parameters were assessed with the Xsens MVN ambulatory system, based on 17 inertial sensors: stride velocity, stride length, stride width, cadence, stance time, swing time and double support time.

**Results**

The results of the Friedman test revealed statistically significant differences between the temporal parameters of single gait (stride velocity: χ^2^=11.200, p = 0.004; cadence: χ^2^=24.102, p = 0.000; stance time: χ^2^=20.133, p = 0.000; swing time: χ^2^=17.733, p = 0.000 and double support time: χ^2^=19.733, p = 0.000) and each of the two dual task conditions. No statistically significant differences were observed between the cognitive verbal fluency and the arithmetic conditions.

**Conclusions**

Our results suggest that spatiotemporal parameters of gait significantly change under both cognitive dual task conditions, and that these changes are detectable with the ambulatory 3D kinematic analysis system used. These findings strongly support the use of body-worn sensors, to early detect changes in gait patterns, promoting timely interventions to prevent falls.

**References**

1. Bridenbaugh SA. Kressing RW. Motor cognitive dual tasking: early detection of gait impairment, fall risk and cognitive decline. Z Gerontol Geriatr. 2015 ;48(1):15-21.

2. Bridenbaugh SA, Kressing RW. Quantitative gait disturbances in older adults with cognitive impairments. Curr Pharm Des. 2014;20(19):3165-3172.

3. Beauchet O, Annweiler C, Dubost V, Allali G, Kressig RW, Bridenbaugh S, Berut G, Assal F, Herrmann FR. Stops walking while talking: a predictor of falls in older adults?. European Journal of Neurology. 2009;16(7):786-795.

4. Hollman JH, Kovash FM, Kubik JJ, Linbo RA. Age-related differences in spatiotemporal markers of gait stability during dual task walking. Gait Posture. 2007;26(1):113-119.

5. Bridenbaugh SA, Kressing RW. Laboratory Review: The Role of Gait Analysis in Seniors’ Mobility and Falls Prevention. Gerontology. 2011;57(3):256-264.

6. Aminian K, Najafi B, Büla C, Leyvraz PF, Robert P. Spatio-temporal parameters of gait measured by an ambulatory system using miniature gyroscopes. J Biomech. 2002;35(5):689-699.

7. Najafi B, Helbostad JL, Moe-Nilssen R, Zijlstra W, Aminian K. Does walking strategy in older people change as function of walking distance?. Gait Posture. 2009;29(2):261-266.

**Keywords**

Dual task, Spatiotemporal parameters, Gait, Older adults.

### P54 Portuguese workers: perception of wellbeing at work in an industrial company

#### Marina Cordeiro^1,2^, José C Gomes^1,2^, Paulo Granjo^3^

##### ^1^School of Health Science, Polytechnic Institute of Leiria, 2411-901 Leiria, Portugal; ^2^Center for Innovative Care and Health Technology, Polytechnic Institute of Leiria, 2411-901 Leiria, Portugal; ^3^Instituto de Ciências Sociais, Universidade de Lisboa, Lisboa, Portugal

###### **Correspondence:** Marina Cordeiro (marina.cordeiro@ipleiria.pt)

**Background**

People spend a long time working, but work can have a negative impact on wellbeing, especially in situations of poor working conditions and dissatisfaction [1, 2]. This may lead to negative consequences for the worker, company and society [2, 3], therefore, it’s important to recognize the wellbeing perception at work of Portuguese workers and which variables may influence it.

**Objective**

To determine worker’s perception of wellbeing at work and its relationship with sociodemographic and occupational characteristics.

**Methods**

A cross-sectional study, descriptive and correlational, was performed in a Portuguese industrial company. The non-probabilistic convenience sample was composed by 134 workers. Data was collected by a self-administered questionnaire including sociodemographic and occupational questions and the Perception of Wellbeing at Work Questionnaire (PWW). The study was approved by an ethical commission.

**Results**

The sample mean age was 38.58 years (SD=9.52; min=21; max=62), manly composed by female workers (77.6%), 44% concluded high school, 61.2% are married/living under common law, and 31.3% has 2 children. Workers worked in the company for about 107.15 months (SD=51.10), 88.1% had a Permanent Employment Contract (PEC), 87.3% works ≤ 40hours a week, 40.3% had as working schedule from 07h00 to 16h00 and 41% were production operators. PWW had a mean of 67.2 (SD=7.8), Adaptation and Adequacy to Work (AAW) dimension of 27.2 (SD=3.2), Organizational Characteristics (OC) of 19.2 (SD=3.9), Interpersonal Relationship (IR) of 11.2 (SD=1.7), Structure of work (SW) of 5.4 (SD=1.3) and Bound with the Institution (BI) of 4.2 (SD=1.6). There were no statistically significant differences (p > 0.05) between PWW and sociodemographic or occupational variables. However, statistically significant differences (p < 0.05) were found in IR according to gender (men with higher average) and the type of contractual affiliation (workers with Term Employment Contract [TEC] had better PWW in IR than those with PEC) and SW according to the workload (workers working ≤ 40 hours have higher PWW in SW). It was also found that IR was negatively correlated with age and with working time for the same function.

**Conclusions**

The sample presents moderate PWW, which assumes higher values in AAW and lower in BI. Older workers and those who have been working longer at the same function have a lower PWW in IR. Male workers and those with a TEC had a better PWW in the IR, and those who worked ≤ 40 hours per week had higher PWW in SW. These results may be useful for designing interventions to promote wellness at work.

**References**

1. International Labour Office. Psychosocial risks, stress and violence in the world of work. International Journal of Labour Research. 2016;8:1-131.

2. Harvey SB, Modini M, Joyce S, Milligan-Saville JS, Tan L, Mykletun A, Bryant RA, Christensen H, Mitchell PB. Can work make you mentally ill? A systematic meta-review of work-related risk factors for common mental health problems. Occupational & Environmental Medicine. 2017; 74(4): 301-310.

3. Cottini E, Lucifora C. Mental Health and Working Conditions in Europe. ILRReview. 2013; 66(4): 958-988.

**Keywords**

Workplace, Mental health, Occupational health, Workers wellbeing.

### P55 Informal caregivers of oldest old people

#### Sara Alves^1, 2^, Constança Paúl^1,2^, Oscar Ribeiro^2,3^

##### ^1^Unidade de Investigação e Formação sobre Adultos e Idosos, Centro de Investigação em Tecnologias e Serviços de Saúde, Instituto de Ciências Biomédicas Abel Salazar, 4050-313 Porto, Portugal; ^2^Centro de Investigação em Tecnologias e Serviços de Saúde, Universidade do Porto, 4200-450 Porto, Portugal; ^3^Departamento de Educação e Psicologia, Universidade de Aveiro, 3810-193 Aveiro, Portugal

###### **Correspondence:** Sara Alves (ssafalves@gmail.com)

**Background**

Living longer may lead to a long period of disability and frailty with increasing care demands. Informal caregivers, namely family members, play a very important role in the provision of care to very old individuals. Informal care represents crucial support but the available knowledge on this topic is still scarce in the Portuguese context.

**Objective**

To provide an overview of caregiving characteristics in a sample of dyads of informal caregivers and oldest old people (80+) from Porto.

**Methods**

A sample of 72 caregiving dyads was considered. Sociodemographic data, information on the caregiving experience (e.g. length of care, relationship with the person cared) and the disability level of care receiver (Activities of Daily Living – ADL’s and IADL’s, and comorbidities) were obtained.

**Results**

Informal caregivers had a mean age of 63.9 years (SD=9.95), were mostly women (76.4%), with children (70.8%), married (63.9%) and retired (50.0%). Time spent on caregiving was on average 9.73 hrs/day (SD=7.61) and the length of care was around 7 years (SD=6.15). Most of the sample had formal social support to help in the care provision: 54.2% receives support from home care services and 34.7% from day centres. Care receivers mean age was 92.0 years (SD=5.28), were mostly women (73.6%) and widowed (65.3%). Half of the care receivers (n=36; 50.0%) were completely dependent in ADL’s, 30.6% were moderately dependent, 16.7% severely dependent, and 2.8 % were independent. Most of the sample was severely dependent in IADL’s (95.8%). The mean number of comorbidities was 6.94 (SD=1.95), and the mean of medicines intake was 6.17 (SD=3.41).

**Conclusions**

This is an ongoing project but current data already shows that the amount of time spent on caring activities by women who are themselves in advanced age is very high, and probably exhausting due to the high level of functional dependency of the care recipients. A clear picture of tasks and probable exhaustion of the group of people taking care of very old people is crucial in order to plan which and how to deliver formal care to these dyads. Such planning is important for preventing/alleviating burden situations and raising the quality of life of care recipients and their families.

**Acknowledgements**

Project was approved by Ethical Commission of ICBAS.UP, approval number 188/2017 and by Portuguese Data Protection Authority (CNPD), approval number 1338/2017.

**Keywords**

Informal caregiving, Oldest old people, Disability.

### P56 Comparison of antioxidant activity for Ginkgo biloba L. and Rosmarinus officinalis L

#### Diana Silva^1^, Ana França^2^, Cláudia Pinho^3^, Ana I Oliveira^3^, Rita F Oliveira^3,4^, Agostinho Cruz^3^

##### ^1^Farmácia Holon, Baguim do Monte, Gondomar, Portugal; ^2^Farmácia Higiénica, Fão, Esposende, Portugal; ^3^Centro de Investigação em Saúde e Ambiente, Escola Superior de Saúde, Instituto Politécnico do Porto, 4200-072 Porto, Portugal; ^4^Secção Autónoma de Ciências da Saúde, Universidade de Aveiro, 3810-193 Aveiro, Portugal

###### **Correspondence:** Cláudia Pinho (clp@eu.ipp.pt)

**Background**

Natural antioxidant products have gained popularity worldwide and are increasingly being used to treat various diseases [1]. Leaves of *Rosmarinus officinalis L.* and *Ginkgo biloba L.* possess a variety of bioactivities, including antioxidant [2].

**Objective**

Therefore, the present study aims to evaluate *in vitro* antioxidant properties of aqueous and hydroalcoholic extracts of three different commercial brands of *R. officinalis* and *G. biloba*.

**Methods**

*R. officinalis* and *G. biloba* leaves from three different commercial brands were extracted with two solvents (water and 80% ethanol), and antioxidant activity of the extracts were screened using the superoxide anion and 1,1-diphenyl-2-picryl hydrazyl (DPPH•) radical scavenging, and metal ion chelating capacity.

**Results**

A comparison of both plant extracts in the DPPH assay and Fe^2+^ chelating activity indicated that *R. officinalis* showed lower IC_50_ values comparing to G. biloba, ranging from 44.1-61.8 μg/mL (aqueous extracts) and 20.8-23.3 μg/ml (hydroalcoholic extracts) in the DPPH assay, and 93.1-329.0 μg/mL (aqueous extracts) and 33.4-71.0 μg/mL (hydroalcoholic extracts) in the Fe^2+^ chelating activity. For the superoxide radical scavenging activity, the hydroalcoholic extracts of *R. officinalis* showed the best IC_50_ values, ranging from 5.1-15.5 μg/mL, with one brand showing and IC_50_ value lower (5.1 μg/mL) than positive control (5.8 μg/mL - ascorbic acid). Results also showed that in both plants and brands, the highest antioxidant activity was found mainly in the hydroalcoholic extracts, for all the assays tested.

**Conclusions**

The findings of this study support the view that some medicinal plants are promising sources of potential antioxidants. The different brands and solvent types used in the present study may influence the chemical composition of the rosemary and ginkgo extracts obtained and therefore their antioxidant capacity.

**References**

1. Zhang A, Sun H, Wang X. Recent advances in natural products from plants for treatment of liver diseases. Eur J Med Chem. 2013;63:570-577.

2. El-Beltagi HS, Badawi MH. Comparison of Antioxidant and Antimicrobial Properties for Ginkgo biloba and Rosemary (Rosmarinus officinalis L.) from Egypt. Not Bot Horti Agrobo. 2013;41(1):126-135.

**Keywords**

Antioxidant activity, *Ginkgo biloba*, *Rosmarinus officinalis*, Solvent extraction.

### P57 The skills of the wound navigator in the health care team

#### Raquel Silva, Filipa Veludo

##### School of Nursing, Institute of Health Sciences, Universidade Católica Portuguesa, 1649-023 Lisbon, Portugal

###### **Correspondence:** Raquel Silva (raklitasol@hotmail.com)

**Background**

Aging co-morbidities are the main reason for skin changes, requiring qualified professionals to assist the person with this problem [1,2]. In this sense, it emerges in the literature even the tenuous concept – wound navigator, which may enhance the approach to the person with wounds, often described as the tissue viability nurse.

**Objective**

Define the wound navigator and identify his skills.

**Methods**

Integrative literature review using electronic research (CINAHL®, Nursing & Allied Health Collection, Cochrane Plus Collection, MedicLatina, MEDLINE®) and manual research in 12 specialty associations in tissue viability, with the following descriptors (wound OR tissue viability OR ulcer) AND (nurs*) AND (care OR role OR skills OR patient care team OR navigator OR manager OR multidisciplinary OR interdisciplinary OR tissue viability service OR interven* OR pratic*). Inclusion criteria were articles in Portuguese, English, Spanish or French, without temporal limitation, full texts and free access. Exclusion criteria were articles that do not address the study phenomenon. The research was conducted on 08/25/2017, where we obtained 601 articles from the databases and 145 from associations. The titles and abstracts of the publications were read, followed by reading the full text of the selected publications. The sample was defined by 19 articles (15 from databases and 4 from associations).

**Results**

Only one article defines wound navigator, as the health professional with knowledge in the specialty, who acts as a defender of the interests of the clients, which combines the needs felt by them; the objectives of the treatment and the health care treatment plan by referral [3]. It collects the results achieved from the practice and dissemination of research, in order to highlight their actions before the policy of health care [3]. The competences found in the remaining 18 articles were divided into 4 categories: quality (training, auditing, research and elaboration of norms and protocols) management (involvement in product choice, articulation with suppliers, promotion of change and ability to work in multi and interdisciplinary team), care (postgraduate knowledge, experience in the area of tissue viability, prescription of specialized care and treatments with advanced therapies) and leadership (communication, supervision and consulting).

**Conclusions**

There is little literature that precisely defines the wound navigator and his skills, therefore more research is needed to describe in detail. When the term is defined and its competences are known, it may through them formally develop teams with nurses specialized in the area, holders of the general and specific attributes identified.

**References**

1. Bianchi, J. Preventing, assessing and managing skin tears. Nurs Times. 2012;108(13):12,14, 16

2. Dutton, M.; Chiarella, M. e Curtis, K. The role of the wound care nurse: an integrative review. Community Wound Care. 2014; Vol. Supplement:S39-40, S42-7.

3. Moore, Z.; Butcher, G.; Corbet, L.P. et al. AAWC, AWMA, EWMA, Position Paper: Managing Wounds as a Team. J Wound Care. 2014;23(5): S1-S38.

**Keywords**

Tissue viability nurse, Skills, Role.

### P58 Nursing interventions in the prevention and management of aggressive behaviors in psychiatric context

#### Aida Bessa^1^, Isabel Marques^2^, Amorim Rosa^2^

##### ^1^Centro Hospital e Universitário de Coimbra, 3000-075 Coimbra, Portugal; ^2^Escola Superior de Enfermagem de Coimbra, 3046-051 Coimbra, Portugal

###### **Correspondence:** Aida Bessa (bessaaida@gmail.com)

**Background**

Aggressive behaviour emerges in social interaction and is therefore considered as a process arising from the relationship between the person and the person’s physical, social and cultural environment over time. In these situations, where the person is experiencing transition processes that generate misaligned human responses, inducing processes of mental suffering as the manifestation of aggressive behaviours, the need to provide specialized nursing care emerges.

**Objective**

The aim of this study was to analyse the most relevant data from the studies about Nursing Interventions in the prevention and management of aggressive behaviours in the psychiatric context, according to the risk profile of the patients.

**Methods**

An integrative review of the literature was carried out, in libraries of national and international organizations, using the B-On search engine, PubMed database - from the question “*What Nursing interventions in the prevention and management of aggressive behaviours in psychiatric context, according to the risk profile of the patients?*” The following keywords were used: Nursing Interventions, Violence, Aggression, and Risk Psychiat*. 343 articles were considered, published in the period 2004-2016, in Portuguese, Spanish, French or English and with full text. After applying the inclusion and exclusion criteria, we obtained 10 articles that were analysed according to the previously defined protocol.

**Results**

Five articles addressed the prevention and management measures implemented to deal with the incidents of aggression, while all the others emphasize the implementation of interventions to prevent aggressive behaviour. Award-winning nursing interventions are non-restrictive (communicational and de-escalation techniques). Observation emerges as the first intervention, including risk assessment. Environmental and chemical containment arise among the containment measures.

**Conclusions**

It was concluded that the risk assessment of aggressive behaviours, upon stratification, followed by the implementation of preventive/management measures, adapted to the risk level, can be easily implemented in the routines of the services. It therefore contributes both to reducing the incidence and severity of these behaviours and to improving the management/reduction of coercive measures.

**Keywords**

Aggressive behaviors, Psychiatry Context, Prevention, Nursing Interventions.

### P59 An advanced nursing practice model proposal to improve heart failure under mechanical circulatory support patients’ outcomes

#### Teresa Pessoa^1,2^, Maria T Leal^2^

##### ^1^Departmento de Cardiologia, Hospital de Santa Maria, Centro Hospitalar de Lisboa Norte, 1649-035 Lisboa, Portugal; ^2^Escola Superior de Enfermagem de Lisboa, 1600-190 Lisboa, Portugal

###### **Correspondence:** Teresa Pessoa (teresapessoa@gmail.com)

**Background**

The use of left ventricular assist devices has grown rapidly in recent years for patients with end-stage heart failure (HF) [1]. HF under Mechanical Circulatory Support (MCS) patient-centred care assumes that nurses have professional competence, knowledge and ability to make decisions and prioritize care [2]. The ability of decision-making, and prioritization of multiple nursing interventions depends on clinical judgement and professional and personal experience [3].

**Objective**

To identify evidence-based nursing interventions that improve the outcomes of patients with HF under MCS and organize them in the form of an Advanced Nursing Practice Model.

**Methods**

Integrative literature review based on a systematic search of original articles and literature reviews published between January 1st, 2010 and August 31st, 2016, in MEDLINE, CINAHL and Cochrane databases and manual search on Google and ResearchGate. Studies related to adult HF patients with formal indication or under MCS in which the first author was a nurse, have been included. Studies related to patients under MCS devices such as intra-aortic balloon counterpulsation or venovenous extracorporeal membrane oxygenation have been excluded.

**Results**

From the 41 articles included, 43 categories emerged through content analysis. Those were grouped in four areas of care where nurses should intervene. The 15 nursing interventions included in the Model were not mutually exclusive, which makes that the same intervention can be applied to more than one area of care. There are no validated protocols to each intervention. The model was constructed based on the review results and on The Strong Model of Advanced Nursing Practice [4], Clinical Judgement [5] and Patient Centred Care [2] frameworks.

**Conclusions**

HF under MCS patients centred-care is complex and requires teamwork and relational skills. It depends on the best available evidence-based scientific knowledge, on stakeholders’ life experience and on context and environment specificity. Patient-related outcomes can be improved through the application of the proposed model. To make it operational, there is a need to standardize practices and to develop protocols, guidelines and training programs to improve advanced nursing practice.

**References**

1. Creaser JW, Rourke D, Vandenbogaart E, Chaker T, Nsair A, Cheng R, et al. Outcomes of biventricular mechanical support patients discharged to home to await heart transplantation. J Cardiovasc Nurs. 2015;30(4):E13–20.

2. McCormack B, McCance TV. Development of a framework for person-centred nursing. J Adv Nurs. 2006;56(5):472–9.

3. Benner P, Tanner CA, Chesla CA. Expertise in nursing practice: caring, clinical judgment and ethics. 2nd ed. Springer Publishing Company. New York: Springer Publishing Company; 2009.

4. Ackerman MH, Norsen L, Martin B, Wiedrich J, Kitzman HJ. Development of a model of advanced practice. Am J Crit Care. 1996;5(1):68–73.

5. Tanner CA. Thinking like a nurse: a research-based model of clinical judgment in nursing. J Nurs Educ. 2006;45(6):204–11.

**Keywords**

Heart failure, Mechanical circulatory support, Nursing interventions, Patient-related outcomes, Advanced nursing practice.

### P60 Acute effects of aerobic exercise on motor memory consolidation in older people

#### André Ramalho^1^, Pedro Duarte-Mendes^1^, Rui Paulo^1^, João Serrano^1,2^, António Rosado^3^, João Petrica^1,2^

##### ^1^Department of Sports and Well-being, Polytechnic Institute of Castelo Branco, 6000-266 Castelo Branco, Portugal; ^2^Centre for the Study of Education, Technologies and Health, Polytechnic Institute of Viseu, 3504-510 Viseu, Portugal; ^3^Faculty of Human Kinetics, University of Lisbon, 1499-002 Lisbon, Portugal

###### **Correspondence:** André Ramalho (andre.ramalho@ipcb.pt)

**Background**

Scientific evidence suggests that an aerobic exercise session promotes improvements in the consolidation of motor memory in adults.

**Objective**

In this sense, the main purpose of this study was to investigate if an aerobic training session could improve motor memory consolidation in older people.

**Methods**

The participants of this study were 33 subjects of both genders (M = 68 years old; SD = 4.2 years old) divided in two groups: a control group and an experimental group. The participants performed a Soda Pop test before the aerobic training session (Baseline). The training session lasted 45 minutes and was composed of running exercises. After the training session, the motor memory consolidation was held in three different stages: Training; 1 hour after training; 24 hours after training. The Shapiro-Wilk test was applied to the normality distribution of data and One-way ANOVA test for parametric statistics.

**Results**

The results indicated that although the experimental group presented a better performance in motor memory consolidation, 1 hour after training and 24 hours after training, the differences were not significant (p ≥ 0.05).

**Conclusions**

Thus, it seems that an aerobic training session does not significantly improve motor memory consolidation in older people.

**Acknowledgements**

This work was supported by the Portuguese Foundation for Science and Technology (FCT; Grant Pest – OE/CED/UI4016/2016).

**Trial Registration**

NCT03506490

**Keywords**

Aerobic exercise, Motor memory consolidation, Learning, Aging.

### P61 Fundamentals of care in the critically ill person in ICU: an integrative literature review

#### Maria J Pires^1^, Helga R Henriques^2^, Maria C Durão^3^

##### ^1^Escola Superior de Enfermagem de Lisboa, 1600-190 Lisboa, Portugal; ^2^Departamento de Fundamentos de Enfermagem, Escola Superior de Enfermagem de Lisboa, 1600-190 Lisboa, Portugal; ^3^Departamento de Enfermagem Médico-Cirúrgica, Escola Superior de Enfermagem de Lisboa, 1600-190 Lisboa, Portugal

###### **Correspondence:** Maria J Pires (m.joao.pires08@gmail.com)

**Background**

In the critically ill person, the priority of nursing interventions is, in most cases, exclusively directed to the management of the clinical situation, not valuing the fundamentals of care [1,2]. These universal activities are essential to the maintenance of life. They are present in each person, regardless of their health condition, whenever there is the necessary strength, will or knowledge [3,4]. In acute or chronic illness, injury or in the healthy person, the fundamentals of care may be disturbed, which motivate the nurses' intervention [3,5].

**Objective**

To know the evidence related to the fundamentals of care in the recovery of the critically ill person.

**Methods**

Integrative Literature Review, using a PICO model clinical question and carried out through the search of articles in MEDLINE, CINAHL and grey literature databases.

**Results**

We identified 1,268 results, of which 11 documents were selected to compose the final sample. The analysis of the documents showed that there is an invisibility of the fundamentals of care in ICU, caused by the predominance of biomedical model and technological care. Some studies, however, highlight the importance of nurses' intervention in patient recovery in areas of fundamentals of care, such as sleep [6], breathing, skin, nutrition, communication, patient and family education, mobilization, positioning and hygiene [7,8].

**Conclusions**

Fundamentals of care contribute to reducing complications during hospitalization of patients in the ICU, such as malnutrition, development of pressure ulcers or infection. The fundamentals of care are neglected in this context of care to the detriment of the technological care, reason why it is necessary to investigate this issue.

**References**

1. Feo R, Kitson A. Promoting patient-centred fundamental care in acute healthcare systems. Int J Nurs Stud. 2016;57:1–11.

2. Henneman EA. Patient Safety and Technology. AACN Advanced Critical Care, 2009;20(2):128–132.

3. Henderson V. Principios Básicos dos Cuidados de Enfermagem do CIE. Lusodidata, Ed. Loures; 2007.

4. Kitson A, Conroy T, Wengstrom Y, Profetto-McGrath J, Robertson-Malt S. Defining the fundamentals of care. Int J Nurs Pract. 2010;16(4):423–434.

5. Clares JWB, Freitas MC, Galiza FT, Almeida PC. Necessidades relacionadas ao sono/repouso de idosos: estudo fundamentado em Henderson. Acta Paul Enferm. 2012;25(Número Especial 1):54–59.

6. Eliassen K, Hopstock L. Sleep promotion in the intensive care unit-A survey of nurses’ interventions. Intensive and Critical Care Nursing. 2011;27(3):138–142.

7. Shahin E, Dassen T, Halfens R. Pressure ulcer prevention in intensive care patients: Guidelines and practice. J Eval Clin Pract. 2009;15(2):370–374.

8. Curtis K, Wiseman T. Back to basics-Essential nursing care in the ED, Part 2. Australasian Emergency Nursing Journal. 2008;11(2):95–99.

**Keywords**

Critical patient, Fundamentals of care, Recovery, Safety.

### P62 Adaptation and validation for the Portuguese population of the Quality of the Carer-Patient Relationship (QCPR) scale: preliminary results

#### Rosa Silva^1^, Paulo Costa^2^, Isabel Gil^3^, Hugo Neves^4,5^, Nele Spruytte, João Apóstolo^2,5^

##### ^1^Universidade Católica Portuguesa, Institute of Health Sciences, 4200-374 Porto, Portugal; ^2^The Health Sciences Research Unit: Nursing, Nursing School of Coimbra, 3046-851 Coimbra, Portugal; ^3^Nursing School of Coimbra, Coimbra, 3046-851, Portugal; ^4^School of Health Science, Polytechnic Institute of Leiria, 2411-901 Leiria, Portugal; ^5^Center for Innovative Care and Health Technology, Polytechnic Institute of Leiria, 2411-901 Leiria, Portugal; ^6^Centre for Evidence Based Practice: A Joanna Briggs Institute Centre of Excellence, 3000-232 Coimbra, Portugal

###### **Correspondence:** Rosa Silva (rcgsilva@porto.ucp.pt)

**Background**

The number of elderly with dementia is hastily increasing. The informal (family) carer are urged to perform more activities in order to maintain the elderly health needs. Assessing the quality of the relationship between the dyad (a family carer and a patient with dementia) is necessary to assure that care plans are well adjusted. The Quality of The Carer-Patient Relationship (QCPR), [1] emerges as a specific scale for assessing the quality of the bidirectional relation of the dyad.

**Objective**

To adapt the QCPR scale to Portuguese and determine its psychometric properties.

**Methods**

Phase (I), transcultural adaptation according to the international recommendations of the American Association of Orthopedic Surgeons [2], composed by four steps; Phase (II), a pilot study was conducted to assess the preliminary psychometric properties in the new cultural context, with a convenience sample composed by patients with dementia (n=30) and their caregivers (n=30). The factorial structure of the QCPR in this sample was evaluated through a confirmatory factorial analysis (CFA) with the AMOS software (v.24, SPSS Inc, Chicago, IL).

**Results**

Phase (I), the initial translations proved to be close to the original content (first step). Less consensual terminology was critically analysed and synthesized by the translators and elements of the research team (second step), resulting in the first version. The versions obtained in the back-translation process (third step) presented good semantic and idiomatic equivalence. After expert consensus (fourth step), the scale was revised in its entirety and punctually reformulated. This resulted in a second translated version, which maintained the original structure (an individualized questionnaire for each element of the dyad, with 14 items of the 5-point Likert-type scale response).

Phase (II), the CFA shows no violation of normality in all variables, with the presence of factorial weights below 0.5 in items 10 and 11 in the caregiver’s version, and in items 13 and 14 in the patient’s version. Regarding the quality of the adjustment of the model, both the caregiver’s version (χ^2^/df=1.304; CFI=0.922; GFI=0.731; RMSEA=0.097 P[RMSEA≤0.05]> 0.05; MECVI=6.739), as well as the patient’s version (χ^2^/df=1.152; CFI=0.955; GFI=0.760; RMSEA=0.069 P[RMSEA≤0.05]> 0.05; MECVI=6.475) present a model with questionable to good adjustment.

**Conclusions**

A strong and positive relation between both latent variables were contrary to what was expected to observe. The fact that only two observations evidenced conflict may indicate that there is a need to strengthen the number and diversity of participants sampled, in order to clarify these preliminary results.

**Acknowledgements**

This study is part of the project “Cognitive stimulation in the elderly: intervention in cognitive fragility and promotion of self-care”, funded by the Nursing School of Coimbra.

**References**

1. Spruytte N, Audenhove C, Lammertyn F, Storms G. The quality of the caregiving relationship in informal care for older adults with dementia and chronic psychiatric patients. Psychology and Psychotherapy: Theory, Research and Practice. 2002;75(3):295-311.

2. Beaton D, Bombardier C, Guillemin F, Ferraz M. Guidelines for the Process of Cross-Cultural Adaptation of Self-Report Measures. Spine. 2000;25(24):3186-3191.

**Keywords**

Patient-carer relationship, Quality of the relationship, Dementia.

### P63 Association between Mediterranean diet and mood in young volunteers

#### Rafael Bravo^1^, Nuria Perera^1^, Lierni Ugartemendia^1^, Javier Cubero^2^, Ana B Rodríguez^1^, Maria A Gómez-Zubeldia^1^

##### ^1^Department of Physiology, Faculty of Science, University of Extremadura, 06071 Badajoz, Spain; ^2^Health Education Laboratory, Experimental Science Education Area, University of Extremadura, 06071 Badajoz, Spain; ^3^Department of Physiology, Faculty of Medicine, University of Extremadura, 06071 Badajoz, Spain

###### **Correspondence:** Rafael Bravo (rbravo@unex.es)

**Background**

Mediterranean diet (MD) is characterized by a high intake of vegetables, a moderate intake of fish and poultry and a low intake of meat and alcohol. MD is considered as a healthy dietary pattern in terms of morbidity and mortality. These benefits have been associated with the ingested levels of olive oil, fibber, non-simple carbohydrates and proteins obtained from vegetables.

**Objective**

In the last years, it has been reported that dietary patterns may influence on several psychological aspects like mood, anxiety or depression. Therefore, our aim was to elucidate whether MD may have a positive effect on the mood of young volunteers.

**Methods**

In this assay participated 52 male volunteers and 80 female volunteers. Participants filled the following scales: Mediterranean Diet Adherence Questionnaire (MDAQ), Beck’s Anxiety Inventory, Beck’s Depression Inventory, Ruminative Response Scale and the Well-Being Index (WHO-5). After data collection, every psychological variable was correlated with the score obtained in the MD Adherence Questionnaire.

**Results**

Related to male participants our results showed a negative correlation between MDAQ and Personal Dysfunction (p < 0.05). On the other hand, related to women, anxiety showed a negative correlation (p < 0.05) and well-being showed a positive correlation (p < 0.05), when correlated with MDAQ.

**Conclusions**

In our sample, we observed that MD may have positive effects on the mood of both young men and women.

**Acknowledgements**

Authors are gratefull to Junta de Extremadura (Fondos FEDER – GR 15051).

**Keywords**

Nutrition, Mediterranean diet, Mood.

### P64 Health education, interprofessional collaboration and infection control in a house of support in souththern Brazil

#### Andressa T Hoffmann, Adriele Timmen, Alzira MB Lewgoy, Nadia M Kuplich, Ester D Schwarz

##### Hospital de Clínicas de Porto Alegre, Porto Alegre, 90035903, Rio Grande do Sul, Brasil

###### **Correspondence:** Andressa T Hoffmann (athoffmann@hcpa.edu.br)

**Background**

Interprofessional collaboration can be understood as the interaction of professionals from different fields of knowledge aiming at a whole and broad health care. In this sense, health education constitutes a means-activity for the promotion of health, with a transforming role of individual practices [1,2]. The emergence of multidrug resistant (MDR) bacteria is a public health problem, and measures to prevent their spread are essential, together with awareness actions on infectious diseases and basic measures of personal and environmental hygiene.

**Objective**

To describe the interprofessional collaboration carried out by a group of professionals, in the areas of social work, nursing and pharmacy of the hospital infection control commission (HICC), with the staff and the children and relatives housed in a house of support from a university hospital, in Brazil.

**Methods**

Experiences reported from July 2016 to December 2017. After identifying the need for a theoretical deepening in prevention and control of infections, especially in the management of children and adolescents with MDR bacteria and their relationships, in the daily routine of the home, a joint planning of actions was started between the infection control team and the support house staff, focusing on health education. Initially, monthly meetings were made among professionals for reflection and problematization of learning and participatory observation, making feasible the situational diagnosis of the house. In these meetings, subjects such as hand hygiene, bacterial transmission, management of individuals with MDR bacteria, matrix-based strategies, hospital and house of support concepts were discussed, enabling a better understanding of the difference of care inside and outside the hospital. After the development and consolidation of concepts, house staff was encouraged to produce health education actions for their public.

**Results**

The performance of a theatrical play was carried out by both teams in June 2017, where it was identified the consolidation of contents by the professionals in the period of theoretical deepening. After this action, monthly workshops were started with the hosted public, carried out with the participation of both teams, in which issues related to the prevention and control of infections were discussed.

**Conclusions**

Health education and interprofessional collaboration enabled the development of the identity of the house of support, the strengthening of the team in topics of infection control, as well as the planning and carrying out of activities with the house's public. Such health promotion actions enabled employees and family members to become multipliers of good practices in infection prevention and control.

**References**

1. Matuda CG, Pinto NRS, Martins CL, Fazão P. Colaboração interprofissional na Estratégia Saúde da Família: implicações para a produção do cuidado e a gestão do trabalho. Ciência & Saúde Coletiva. 2015; 20(8):2511-2521.

2. Candeias NMF. Conceitos de educação e de promoção em saúde: mudanças individuais e mudanças organizacionais. Rev. Saúde Pública. 1997; 31(2):209-213.

**Keywords**

Heath education, Infection control, Interprofessional collaboration.

### P65 Promotion of patient safety in nursing practice: what strategies?

#### Tânia Ferreira^1^, Rita Marques^2^

##### ^1^Universidade Católica Portuguesa, 1649-023 Lisboa, Portugal; ^2^Escola Superior de Saúde da Cruz Vermelha Portuguesa, 1300-906 Lisboa, Portugal

###### **Correspondence:** Tânia Ferreira (tania.vaf@gmail.com)

**Background**

Patient safety is a priority for World Health Organization (WHO). The organization considers it fundamental to develop a safety culture among health professionals in general and among nurses in particular, ensuring the safety and quality of the health care.

**Objective**

The purpose of this literature review (LR) was to identify nursing strategies for promoting patient safety.

**Methods**

Using the methodology recommended by the Cochrane Centre, this LR was guided by the research question: “*What are the strategies that promote patient safety associated with nursing practice?”* A research was carried out in scientific databases, in EBSCOhost (CINAHL® MEDLINE®) and SciELO, with publication dates between January of 2012 and December of 2017, with descriptors “patient safety”, “safety culture” and “nursing care”, having emerged 47 articles. After reading the title and abstract, 8 articles were selected to answer the research question.

**Results**

The identification of risks for people during nursing care and the incorporation of good practices and trust in teamwork, contribute to the improvement and development of a safety culture in health care [1,2]. The guarantee of satisfaction of the professional, the communication between professionals and institution, and the support to the team given by the administrations, are strategic factors for the assurance of patient safety [3, 4]. Nurses' perceptions about the safety culture of the patient and the intention to report adverse events are significant in promoting patient safety [5-7]. Coaching behaviour showed a significant and positive correlation with safety culture and coaching behaviours of team leaders were associated with higher degrees of perceived safety culture and stronger intentions to report adverse events [2,8]. Openness of communication and non-punitive responses to mistakes, as well as teamwork influence the patient's safety culture [5, 7].

**Conclusions**

Through this LR it was possible to perceive that the safety culture is an issue still underdeveloped in health organizations, where nurses have an essential role. Nursing practice is related to the professionals' perception of patient safety, which is related to a set of strategies that minimize the risks of adverse events.

**References**

1. Oliveira R, Leitão I, Silva L, Figueiredo S, Sampaio R, Gondim M. Estratégias para promover segurança do paciente: da identificação dos riscos às práticas baseadas em evidências. Esc Anna Nery. 2014; 18(1): 122-129.

2. Hwang J. What are hospital nurses’ strengths and weaknesses in patient safety competence? Findings from three Korean hospitals. International Journal for Quality in Health Care. 2015; 27(3): 232-238.

3. Rigobello MCG, Carvalho REFL, Cassiani SHDB, Galon T, Capucho HC, Deus NN. Clima de segurança do paciente: percepção dos profissionais de enfermagem. Acta Paul Enferm. 2012; 25(5): 728-35.

4. Mayeng LM, Wolvaardt JE. Patient safety culture in a district hospital in South Africa: Na issue of quality. Curationis. 2015; 38 (1).

5. Ammouri AA, Tailakh AK, Muliira JK, Geethakrishnan R, Al Kindi SN. Patient safety culture among nurses. International Nursing Review. 2015; 62: 102-110.

6. Costa TD, Salvador PTCO, Rodrigues CCFM, Alves KYA, Tourinho FSV, Santos VEP. Percepção de profissionais de enfermagem acerca de segurança do paciente em unidades de terapia intensiva. Rev Gaúcha Enferm. 2016; 37 (3).

7. Khater WA, Akhu-Zaheya LM, AL-Mahasneh SI, Khater R. Nurses’ perceptions of patient safety culture in Jordanian hospitals. International Nursing Review. 2015; 62: 82-91.

8. Ko Y, Yu S. The Relationships Among Perceived Patients’ Safety Culture, Intention to Report Errors, and Leader Coaching Behavior of Nurses in Korea: A Pilot Study. J Patient Saf. 2015; 00.

**Keywords**

Patient safety, Safety culture, Strategies, Nursing.

### P66 End of life person’s evaluation criteria in the decision making regarding artificial nutrition

#### Tânia Afonso^1^, Filipa Veludo^1^, Patrícia P Sousa^1^, Sónia Santos^2^

##### ^1^Instituto de Ciências da Saúde, Escola de Enfermagem, Universidade Católica Portuguesa, 1649-023 Lisboa, Portugal; ^2^Unidade de Cuidados Intensivos Polivalente, Hospital Prof Doutor Fernando Fonseca, 2720-276 Amadora, Portugal

###### **Correspondence:** Tânia Afonso (tafonso3@gmail.com)

**Background**

Artificial nutrition at the end of life is assumed as a medical intervention, however for a large percentage of person’s and families is considered as basic care [1]. Thinking about artificial nutrition and the end of life person, such as the person with advanced, incurable and progressive disease, with a survival expectancy between 3 to 6 months [2] is often reflected on a set of issues. This is a controversial discussion, about the quality of life resulting of one of these means and ethical questioning [3]. It’s relevant to look to the user/family as one, which motivates the urgent intervention of the nurses in decision-making support.

**Objective**

Identify scientific evidence regarding the end-of-life evaluation criteria, to be considered in the nurses’ decision-making about artificial nutrition.

**Methods**

Literature Review (15-06-2017) with PRISMA guidelines for reviews [4] in Academic Search Complete, Complementary Index, CINAHL Plus with Full Text®, Psychology and Behavioural Sciences Collection, ScieELO, MEDLINE®, Directory of Open Access Journals, Supplemental Index, ScienceDirect, Education Source, Business Source Complete and MedicLatina. Inclusion/exclusion criteria: nurses who care for adult/elderly persons at the end of life, excluding nurses who care for children; articles about nurses’ intervention in nutrition care to the person at the end of life and the person’s evaluation criteria; full text; in French/Spanish/English/Portuguese; peer-reviewed; published between 2000-2017. A sample of 11 articles was selected.

**Results**

The evaluation criteria to be considered when making decisions on artificial nutrition are: the evaluation of symptoms/problems; emotional value of food; the meaning of the diet for the person at the end of life and definition of prognosis [3,5-6]. In every decision-making, it should be considered the existence of a clinical indication/treatment, a therapeutic objective and the informed consent of a user or legal guardian.

**Conclusions**

It is concluded that the decision on artificial nutrition should integrate the person at the end of life and family, be taken by an interdisciplinary team, considering the definition of the prognosis and the effectiveness of the treatment applied [3]. The intervention of the nurse is understood as a primordial one, based on the best evidence, in relation of proximity [5] considered, simultaneously, the principle of autonomy, beneficence, non-maleficence and justice. There is little evidence of end-of-life nutrition and new studies on the role of nurses within the interdisciplinary team are suggested.

**References**

1. Stiles E. Providing artificial nutrition and hydration in palliative care. Nursing Standard. 2013, 27: 35-42.

2. Barbosa A [et al.]. Manual de Cuidados Paliativos. Lisboa: Núcleo de Cuidados Paliativos, Centro de Bioética da Faculdade de Medicina da Universidade de Lisboa. 2010, 2ª edição;

3. Alves P. Intervenção do Enfermeiro que Cuida da Pessoa em Fim de Vida com Alterações do Comer e Beber. Pensar Enfermagem. 2013, 17(1): 17-30;

4. Moher D [et al.]. Preferred Reporting Items for Systematic Reviews and Meta-Analyses: The PRISMA Statement. Ann Intern Med. 2009, 151(4): 264-269;

5. Bryon E [et al.]. Decision-making about artificial feeding in end-of-life care: literature review. Journal of Advanced Nursing. 2008, 63(1): 2-14;

6. Holmes S. Withholding or withdrawing nutrition at the end of life. Nursing Standard. 2010, 25(14): 43-46.

**Keywords**

Nursing, Artificial nutrition, Therapeutic obstinacy, Integrative review.

### P67 Psychometric properties of the Portuguese version of personal outcomes scale for children and adolescents: an initial research

#### Cristina Simões^1,2^, Célia Ribeiro^1^

##### ^1^Economics and Social Sciences Department, Portuguese Catholic University, 3504-505 Viseu, Portugal; ^2^Research Centre on Special Education, Faculdade de Motricidade Humana, 1495-687 Cruz Quebrada, Portugal

###### **Correspondence:** Cristina Simões (cristina-ferreira@iol.pt)

**Background**

The quality of life (QOL) assessment of children and adolescents has been particularly important in the field of intellectual disability (ID) during the past years. Special Education needs to use a systematic approach to the assessment of the QOL domains, in order to implement a social-ecological model and to promote full inclusion in all contexts of life. It is important to develop a scale that provides simultaneously self-report and report-of-others measures to gather information based on a multiperception strategy and to encourage person-centred planning.

**Objective**

This research aims to analyse the validity and reliability of the Portuguese version of the Personal Outcomes Scale for Children and Adolescents (POS-C).

**Methods**

Data were collected from 54 children and adolescents with ID (M_age_ = 12.48, SD = 2.93) and respective proxies (M_age_ = 46.59, SD = 5.68). After the cross-cultural adaptation stage, the validity (content, construct) and the reliability (test-retest, Cronbach’s alpha, inter-rater) properties of the POS-C were examined.

**Results**

All items of the POS-C were considered relevant by 10 experts, who agreed on a Portuguese version of the scale. The scores of the content validity index (CVI) of each item (≥ .80), the scale CVI-universal agreement (≥ .84), the scale CVI-average (≥ .99) and the Cohen’s kappa (≥ .44) showed suitable content validity of the scale. The total score from self-report and domains ranged from moderate (r = .42 in emotional well-being) to high (r = .82 in social inclusion). Regarding the report-of-others, the Pearson’s coefficients ranged from moderate (r = .49 in emotional well-being) to high (r = .85 in interpersonal relations). The test-retest scores were high in practitioners (r = .95) and in family members (r = .90). The internal consistency reliability of the self-report domains ranged from .41 (interpersonal relations) to .70 (self-determination), and in report-of-others ranged from .54 (physical well-being) to .79 (emotional well-being). The overall scale demonstrated good Cronbach’s alpha scores (α = .81 in self-report and α = .87 in report-of-others). The inter-rater of domains ranged from .47 (interpersonal relations) to .81 (personal development).

**Conclusions**

This initial research on the psychometric properties of the scale, introduces the POS-C as a useful measure of personal outcome scales for Portuguese children and adolescents with ID. POS-C is an important tool to improve personalized support plans, based on self-report and report-of-others measures.

**Keywords**

Quality of life, Intellectual disability, Cross-cultural adaptation, Validity, Reliability.

### P68 Development and validation of a multi-domain digital cognitive stimulation program for older adults with mild to moderate cognitive decline

#### Filipa C Couto^1^, Maria A Dixe^1,2,3^, Jaime Ribeiro^2,3,4^, Mónica Braúna^2,3^, Luís Marcelino^5^, João Apóstolo^6^

##### ^1^The Health Sciences Research Unit: Nursing, Nursing School of Coimbra, Coimbra, 3000-232, Portugal; ^2^School of Health Science, Polytechnic Institute of Leiria, 2411-901 Leiria, Portugal; ^3^Center for Innovative Care and Health Technology, Polytechnic Institute of Leiria, 2411-901 Leiria, Portugal; ^4^Research Centre on Didactics and Technology in the Education of Trainers, Department of Education and Psychology, University of Aveiro, Campus Universitário, Aveiro 3810-193, Portugal; ^5^Informatics Engineering Department, School of Technology and Management, Polytechnic of Leiria, Leiria, 2411-901, Portugal; ^6^The Health Sciences Research Unit: Nursing, Portugal Centre for Evidence Based Practice: A Joanna Briggs Institute Centre of Excellence, Nursing School of Coimbra, Coimbra, 3000-232, Portugal

###### **Correspondence:** Filipa C Couto (filipadccouto@gmail.com)

**Background**

Frail older adults present a decline on their cognitive function. Cognitive interventions are related to the maintenance of cognitive function and are associated with independence and well-being [1, 2]. A cognitive intervention could be considered as a complex intervention once it contains several interacting components [3]. The MIND&GAIT project expects the development of a structured digital cognitive stimulation program to be used with older adults with mild to moderate cognitive decline.

**Objective**

To develop and validate an elderly-friendly multi-domain digital cognitive stimulation program.

**Methods**

To develop the program, the research team followed Guidelines for complex interventions from The Medical Research Council. The development process comprises four phases [3]. The first phase corresponds to a Preliminary Phase (I), the second to a Modelling Phase (II), the third to a Field Test Phase (III) and the last to a Consensus Conference Phase (IV).

**Results**

The digital stimulation cognitive program has 8 individual sessions and 1 group session. The program has already passed for Phase I, which corresponds to an initial conceptualization of the program design and its support materials. At the moment, it is on Phase II, being presented to an experts’ panel to gather different opinions and evaluations from specialists in the area of cognitive interventions. Each session of the program will later be evaluated at Phase III. All the contributions and analysis resulted from the previous phases will be synthesized in phase IV. It is expected that the critical evaluation, provided by specialists in the area of cognitive interventions, will result in a well-founded and structured intervention to be applied in older adults with mild to moderate cognitive decline. The program’s construction, supported by guidelines and based in elderly-friendly digital components, intends to give a response to the challenge of an increasing aged population allying e-health. The program is to be used by health professionals and informal caregivers, in order to work as a possible way to prevent or minimize cognitive decline.

**Conclusions**

Cognitive interventions have impact on cognitive decline, a condition that assumes more importance once it is related with frailty in older adults. Although being a multidomain program, it also potentiates people for activities of daily living. As a complex intervention, this program allows health professionals and other people to apply nonpharmacological interventions which can represent the implementation of best practice towards the needs of an ageing population.

**Acknowledgements**

The current abstract is being presented on behalf of a research group. It is also part of the MIND&GAIT project Promoting independent living in frail older adults by improving cognition and gait ability and using assistive products, which is a Portuguese project with the support of COMPETE 2020 under the Scientific and Technological Research Support System, in the copromotion phase. We acknowledge The Health Sciences Research Unit: Nursing (UICISA:E) of the Nursing School of Coimbra, the Polytechnic of Leiria and also to other members, institutions and students involved in the project.

**References**

1. Apóstolo J, Holland C, O'Connell MD, Feeney J, Tabares-Seisdedos R, Tadros G et al. Mild cognitive decline. A position statement of the Cognitive Decline Group of the European Innovation Partnership for Active and Healthy Ageing (EIPAHA). Maturitas. 2016;83:83-93.

2. Mewborn CM, Lindbergh CA, Miller LS. Cognitive interventions for cognitively healthy, midly impaired and mixed samples of older adults: a systematic review and meta-analysis of randomized-controlled trials. Neuropsychol Rev. 2017;27(4):403-439.

3. Craig P, Dieppe P, Macintyre S, Michie S, Nazareth I, Petticrew M. Developing and evaluating complex interventions: the new Medical Research Council guidance. BMJ. 2008;337:a1655.

**Keywords**

Aged, Cognitive decline, Cognitive stimulation, Frailty, Complex intervention.

### P69 Tele-enfermeiro evolution

#### Telmo Sousa^1^, Pedro Brandão^1,4^, Paulino Sousa^2,3^, João Rodrigues^4,5^

##### ^1^Faculdade de Ciências, Universidade do Porto, 4169-007 Porto, Portugal; ^2^Centro de Investigação em Tecnologias e Serviços de Saúde, 4200-450 Porto, Portugal; ^3^Escola Superior de Enfermagem do Porto, 4200-072 Porto, Portugal; ^4^Instituto de Telecomunicações, 1049-001 Lisboa, Portugal; ^5^Administração Regional de Saúde do Norte, 4000-099 Porto, Portugal

###### **Correspondence:** Telmo Sousa (telmocatarina17@gmail.com)

**Background**

Despite the high technological growth envisaged in the health area, the use of technologies is still scarce when we refer to the Primary Healthcare (PHC). PHC care is very important because it allows for the implementation of proximity interventions, such as nursing home care, resulting in an improvement of the health of individuals, families, and communities. The system mentioned above is the one implemented in health centres called S-Clinic and developed by the Serviços Partilhados do Ministério da Saúde (SPMS). However, this process presents some problems, such as the time spent collecting patient data; the introduction of the intervention data into the system, and the lack of a support structure for the data records extracted from the patient by the nurse.

**Objective**

Combining technological evolution, the importance of PHC and the difficulty of the nursing process at home, we propose the development of an application for mobile devices, with the objective of allowing nurses to import patient data through information, data records of the interventions carried out in an electronic format, which are then exported to the system.

**Methods**

In this way, the application will facilitate the work of the nurse because it replaces the records on paper, thus allowing a better collection and structuring of the data, as well as the increase of the efficiency of the work activity, and reduction of the time spent for the collection and introduction of data in S-Clinic. We had to study the essential contents of the nursing process at home and implemented in the system, in order to create a data structure with the closest resemblance to S-Clinic. Then, to obtain this information a meeting was held with nursing experts to provide their knowledge in this area.

**Results**

In this meeting, the contents considered essential for a domicile were addressed, and the key points were: nursing focus or diagnosis and nursing intervention. The data model was implemented in order to cover all the contents. Some security measures that could be implemented have also been discussed, in order to protect data. After the application development was complete, a meeting was held with some of the nursing experts present at the first meeting to gather requirements, in order to evaluate the system.

**Conclusions**

The feedback was very positive, encouraging the research team to continue this development because they see a good solution for the future of the PHC at the home environment.

**Acknowledgements**

This article is a result of the project NanoSTIMA Macro-to-Nano Human Sensing: Towards Integrated Multimodal Health Monitoring and Analytics, Norte-01-0145-FEDER-000016, supported by Norte Portugal Regional Operational Programme (NORTE 2020), through Portugal 2020 and the European Development Fund.

**Keywords**

Nursing, Health information system, Digital health, Innovation, Development.

### P70 Construction of parenthood - role of the family nurse

#### Andreia MJS Azevedo, Elsa MOP Melo, Assunção DL Almeida

##### School of Health Sciences, University of Aveiro, 3810-193 Aveiro, Portugal

###### **Correspondence:** Andreia MJS Azevedo (jarmelo.andreia@gmail.com)

**Background**

The birth of the first child is presented as the most challenging responsibility facing the family, requiring the adaptation of their interactions with singular and holistic impact. The first six months emerge as a transient, predictable and irreversible milestone. The transition to parenting is marked by the changes and repercussions that drive the child's growth and development, predating and precipitating others in the family life cycle. Supporting families in transition is the competence of nurses, namely, their intervention in the field of family health nursing, in the training for the construction of parenting.

**Objective**

Understand how parents build their parenting model during the first semester of life of the first child. Analyse parents' expectations and constraints/difficulties in the transition to paternity. Explore identity figures and resources mobilized by parents in the transition to parenthood.

**Methods**

We developed a phenomenological study of qualitative nature, with a non-probabilistic sampling of convenience that includes 11 subjects, parents with the first child to complete six months of life between October and December 2016, enrolled in USF Rainha D. Tereza. We conducted semi-structured interviews, obtaining the narratives of the experiences and their deepest understanding of them. We complied with ethical procedures and submitted the information collected to analysis using WEBQDA Software.

**Results**

We highlight the experience of parenting in the desire to be a parent, and in the expectations created in pregnancy, contributing to the parental model. This is determined by factors such as the characteristics of the child, the characteristics and previous experiences of the parents, and the family dynamics. Parents face difficulties in providing care for the child and reconciling parental, marital and familial and social roles. Faced with these difficulties, parents use human, community and monetary resources. We highlight the community resource in support of health care, which is valued by parents. The family nurse, when identified and recognized, is described as an effective and accessible resource in adapting to parenting.

**Conclusions**

The results obtained by the research carried out allowed us to acknowledge the experience of the transition to parenthood of the parents interviewed and to affirm the role of the family nurse, in their capacity to build their own model of parenting, as well as contributing with knowledge to be valued in nursing interventions.

**Keywords**

Nursing, Family, Transition, Parenthood.

### P71 Antioxidant activity of the garlic (Allium sativum L.) submitted to different technological processes

#### Carla Sousa^1^, Catarina Novo^2^, Ana F Vinha^1,3^

##### ^1^Unidade de Investigação em Energia, Ambiente e Saúde, Centro de Estudos em Biomedicina, Fundação Fernando Pessoa, 4249-004 Porto, Portugal; ^2^Universidade Fernando Pessoa, 4249-004 Porto, Portugal; ^3^REQUIMTE/LAQV, Departamento de Ciências Químicas, Faculdade de Farmácia da Universidade do Porto 4051-401 Porto, Portugal

###### **Correspondence:** Ana F Vinha (acvinha@ufp.edu.pt)

**Background**

Garlic has become extensively investigated by its benefits for health. Some therapeutic activities are attributed to the garlic, namely, antioxidant, hepatoprotective, anticancer and antitumor properties, among others [1-3].

**Methods**

Therefore, the total phenolic content (TPC) and the total flavonoid content (TFC) have been determined, as well as the antioxidant properties of extracts of the different forms of presentation/parts of the garlic existing in the market (bulb, in powder and in tablets/capsules), by the radical 2,2-diphenyl-1-picrylhydrazyl (DPPH•) method and by evaluation of the ferric reducing antioxidant power (FRAP). The scavenging capacity of the same extracts against reactive species (O_2_^-^•, H_2_O_2_, NO•) was also evaluated. Finally, the biological activity of the presentation forms of garlic existing in the market was compared with the one of the garlic peel, considered food waste, taking also into account some variables that can influence the properties of the bulb, that is, boiling and freezing.

**Results**

TPC was superior in the frozen chopped garlic sample, having the garlic tablets the lowest content. The cooked garlic presented an inferior value of TPC when comparing with the raw chopped bulb. These results indicate that cooking and freezing methods intervene directly with the total phenolic content, but in an opposite way. The extract of cooked garlic had the higher value of TFC, belonging the lowest tenor to garlic tablets. Radical DPPH• and FRAP methods allowed to verify that the cooked garlic extract evidenced a superior antioxidant activity. This result can be explained by cell wall rupture derived from heating, provoking antioxidant substance release, new and/or stronger antioxidant substance formation or oxidant enzymes inhibition [4]. The frozen chopped garlic extract presented the highest scavenging capacity of the three studied reactive species. In general, the higher the total phenolic content, the greater the capacity of inhibition of reactive species NO•, O_2_^-^• and H_2_O_2_.

**Conclusions**

This study has showed that the diverse forms of presentation/parts of the garlic possess high bioactive compounds content, and consequently antioxidant activity, presenting health benefits.

**References**

1. Banerjee SK, Mukherjee PK, Maulik SK. Garlic as an antioxidant : the good, the bad and the ugly. Phytother Res. 2003, 17(2): 97–106.

2. Naji KM, Al-Shaibani ES, Alhadi FA, Al-Soudi SA, D’souza MR. Hepatoprotective and antioxidant effects of single clove garlic against CCl4-induced hepatic damage in rabbits. BMC Complement Altern Med. 2017, 17: 411.

3. Oommen S, Anto RJ, Srinivas G, Karunagaran D. Allicin (from garlic) induces caspase-mediated apoptosis in cancer cells. Eur J Pharmacol. 2004, 485(1-3): 97-103.

4. Ali M, Mahsa M, Barmak MJ. Effect of boiling cooking on antioxidant activities and phenolic content of selected iranian vegetables. Res J Pharm Biol Chem Sci 2015, 6(3): 663-641.

**Keywords**

*Allium sativum L.*, Bioactive compounds, Antioxidant activity, Reactive species.

### P72 Influence of gamma irradiation in the antioxidant potential of pumpkin seeds and mung beans

#### Anabela Macedo^1^, Carla Sousa^2^, Ana F Vinha^2,3^

##### ^1^Universidade Fernando Pessoa, 4249-004 Porto, Portugal; ^2^Unidade de Investigação em Energia, Ambiente e Saúde, Centro de Estudos em Biomedicina, Fundação Fernando Pessoa, 4249-004 Porto, Portugal; ^3^REQUIMTE/LAQV, Departamento de Ciências Químicas, Faculdade de Farmácia da Universidade do Porto 4051-401 Porto, Portugal

###### **Correspondence:** Ana F Vinha (acvinha@ufp.edu.pt)

Food conservation is a challenge for the food industry. The high respiration rate, the lack of physical protection to avoid water loss and the changes due to microbial attack are often associated with loss of food quality, contributing to deterioration through browning, weight loss and texture changes [1]. Furthermore, bacteria, moulds, enzymatic activity (mainly polyphenol oxidase) and biochemical changes can cause spoilage during storage [2]. The use of ionizing energy for preservation has been widely studied by the food industry. However, studies evaluating the effects of ionizing radiation are mostly available in cultivated species, being scarce the reports on wild species and food waste, considered add-value foods. In this regard, food technology is making progress towards increasing food preservation and contributing to a reduction of the incidence of food-related diseases. Previous studies assessing the potential of gamma irradiation as a suitable technique to increase natural products shelf-life were focused in nutritional and chemical parameters, including bioactive compounds and their antioxidant activity [3]. Many natural compounds found in edible food wastes (seeds) or grains (beans) present antioxidant activity. Among the most important natural antioxidants are phenolic compounds (flavonoids, phenolic acids and tannins), nitrogenous compounds (alkaloids, amino acids, peptides, amines and chlorophyll byproducts), carotenoids, tocopherols and ascorbic acid.

In the present work, the effects of gamma radiation dose (0, 0.5, 1.0, 1.5 and 5.0 kGy) on the chemical composition (total phenolics and total flavonoids) of pumpkin seeds and mung beans were evaluated. The antioxidant activity was studied using DPPH• and FRAP assays. It was observed a slight increase in the content of bioactive compounds, as well as in antioxidant activity, with irradiation doses below 1.5 kGy. Final results showed that irradiation may be a viable technique to guarantee the content of bioactive compounds, as well as their biological properties, including antioxidant activity.

**References**

1. Singh P, Langowski HC, Wanib AA, Saengerlaub S. Recent advances in extending the shelf life of fresh Agaricus mushrooms: a review. J Sci Food Agric. 2010, 90: 1393-1402.

2. Fernandes Â, Barreira JCM, Antonio AL, Bento A, Botelho ML, Ferreira ICFR. Assessing the effects of gamma irradiation and storage time in energetic value and in major individual nutrients of chestnuts. Food Chem Toxicol. 2011, 49: 2429-2432.

3. Antonio AL, Fernandes Â, Barreira JCM, Bento A, Botelho ML, Ferreira ICFR. (Influence of gamma irradiation in the antioxidant potential of chestnuts (Castanea sativa Mill.) fruits and skins. Food Chem Toxicol. 2011, 49: 1918-1923.

**Keywords**

Food conservation, Gamma irradiation, Pumpkin seeds, Mung beans, Antioxidants.

### P73 The effects of swimming and swimming complemented with water walking on spirometry values

#### Pedro Duarte-Mendes^1,2^, Samuel Honório^1,2^, João Oliveira^1^, João Petrica^1,3^, André Ramalho^1,2^, António Faustino^1^, Rui Paulo^1,2^

##### ^1^Department of Sports and Well-being, Polytechnic Institute of Castelo Branco, 6000-084 Castelo Branco, Portugal; ^2^Research on Education and Community Intervention, 4411-801 Arcozelo, Vila Nova de Gaia, Portugal; ^3^Centre for the Study of Education, Technologies and Health, Polytechnic Institute of Viseu, 3504-510 Viseu, Portugal

###### **Correspondence:** Pedro Duarte-Mendes (Pedromendes@ipcb.pt)

**Background**

Spirometry is a standard pulmonary function test that measures how an individual inhales or exhales volumes of air as a function of time. It is the most important and most frequently performed pulmonary function testing procedure, having become indispensable for the prevention, diagnosis and evaluation of various respiratory impairments. However, there have been only a few studies addressing the effect of physical activity on pulmonary function test results and investigating the association between body composition and respiratory parameters in sports activities [1-3].

**Objective**

The objective of this study was to verify if there are differences in spirometry values in children aged between 6 and 12 years who practice swimming complemented with water walking at the end of each session and those who only practice swimming.

**Methods**

In this study 28 subjects (mean age, 7.68 ± 1.16 years) participated and were divided into two groups: swimming group (SG) (N=9) and swimming complemented with water walking group (SWWG) (N=19). The study was performed in 12 weeks with 3 moments of evaluation (M1, M2 and M3), with two sessions per week of 45 minutes each, we wanted to identify the benefits in pulmonary function - Forced Vital Capacity (FVC), Forced Expiratory Volume in 1 second (FEV1) and Peak Expiratory Flow (PEF). The water walking activity occurred at the end of each session for 6 minutes, performed in straight line with the water level at the children’s chest. The spirometry tests were realized with the microQuark Spirometer®. For the analysis of the results, we used descriptive statistics, the Shapiro Wilk test for testing the normality of the sample and for inferential statistics the Mann-Whitney tests, Friedman's Anova, and d-Cohen for the magnitude of effect.

**Results**

The results show, that from the inter-group analysis (comparison between the SG and the SWWG) we observe that there were significant differences in the FVC (M2 - p=0.025), VEF1 (M2 - p=0.01; M3 - p=0.008) and PEF (M1 - p=0.033; M2 - p=0.012; M3 - p=0.037) values. Concerning intra-group differences (improvement in the SG and the SWWG in the three moments evaluated), the SWWG showed significant differences in FVC (p= 0.003) and FEV_1_ (p=0.008), and the SG showed significant differences in VEF_1_ (p=0.034) and PEF (p=0.013).

**Conclusions**

These results show that “swimming” and “swimming complemented with water walking” show improvements in spirometry values in children. The swimming complemented with water walking group showed better results.

**Acknowledgements**

This work was supported by the Portuguese Foundation for Science and Technology (FCT; Grant Pest – OE/CED/UI4016/2016).

**Trial Registration**

NCT03506100

**References**

1. Durmic T, Lazovic B, Djelic M, Lazic J, Zikic D, Zugic V, Dekleva M, Mazic S. Sport-specific influences on respiratory patterns in elite athletes. J Bras Pneumol. 2015, 41: 516-522.

2. Vaithiyanadane V, Sugapriya G, Saravanan A, Ramachandran C. Pulmonary function test in swimmers and non-swimmers- a comparative study. Int J Biol Med Res. 2012, 3, 1735-1738.

3. Lopes, M. d., Bento, P. C., Lazzaroto, L., Rodacki, A. F., & Leite, N. (2015). The effects of water walking on the anthropometrics and metabolic aspects in young obese. Rev Bras Cineantropom Desempenho Hum. 2015, 17, 235-237.

**Keywords**

Spirometry, Swimming, Water walking.

### P74 Preliminary translation and validation of Movement Imagery Questionnaire – Children (MIQ-C) to Portuguese

#### Pedro Duarte-Mendes^1,2^, Daniel Silva^1^, João Petrica^1,3^, Daniel Marinho^4,5^, Bruno Travassos^4,5^, João Serrano^1,3^

##### ^1^Department of Sports and Well-being, Polytechnic Institute of Castelo Branco, 6000-084 Castelo Branco, Portugal; ^2^Research on Education and Community Intervention 4411-801 Arcozelo – Vila Nova de Gaia, Portugal; ^3^Centro de Estudos em Educação, Tecnologias e Saúde, Instituto Politécnico de Viseu, 3504-510 Viseu, Portugal; ^4^Department of Sport Sciences, University of Beira Interior, 6201-001 Covilhã, Portugal; ^5^Research Center in Sports Sciences, Health Sciences and Human Development, University of Tras-os-Montes and Alto Douro, 5001-801 Vila Real, Portugal

###### **Correspondence:** Pedro Duarte-Mendes (Pedromendes@ipcb.pt)

**Background**

The ability to perform movement imagery has been shown to influence motor performance and learning in sports and rehabilitation. Imagery is a cognitive process that can play an important role in planning and execution of movements or actions. Several instruments have been developed in order to evaluate the ability of Imagery in adults such has the MIQ-3, with Portuguese Athletes [1]. However, none focused on imagery ability questionnaire for children with the three modalities (kinesthetic, visual internal and visual external imagery) for Portuguese children’s.

**Objective**

The objective of this study was to translate and validate preliminary, for the Portuguese children’s population, the Movement Imagery Questionnaire for Children [2], determining its initial psychometric qualities through an exploratory factor analysis model that supports it.

**Methods**

In this study 162 subjects of both genders (124 male, 38 female) with a mean age of 10.1 years (SD = 16) participated. For the development of the Portuguese adaptation of the evaluation instrument, a methodology was developed in two phases: (1) the translation phase and cultural adaptation of the questionnaire and (2) the application of the Exploratory Factor Analysis method of the instrument. In the statistical analysis of data, we used the Kaiser-Meyer-Olkin (KMO) and Bartlett tests, to evaluate the quality of the correlations, and an exploratory factorial analysis (EFA) to determine the number of factors to be retained, the number of items associated with them and their internal consistency. The type of rotation adopted was the oblique rotation Promax.

**Results**

Initially it was found that the procedures of translation and adaptation originated a Portuguese version of MIQ - C similar to the original version. Secondly, we found that the psychometric qualities proved their suitability of adaptation performed (KMO=0.822, Bartlett test p=.000), demonstrating that its factor structure is the same as the original version (12 items grouped into 3 factors, with 4 items each factor), with quite acceptable levels of validity and reliability (Cronbach's alpha: 0.85 to MIQ - C, 0.79 for the kinesthetic imagery, 0.74 for the visual internal imagery and 0.76 for the visual external imagery).

**Conclusions**

The results showed that the Portuguese version of the Movement Imagery Questionnaire for Children, with the aim to assess the imagery ability in three modalities (kinesthetic, visual internal and visual external imagery) has quite acceptable indexes for its validation.

**Acknowledgements**

This work was supported by the Portuguese Foundation for Science and Technology (FCT; Grant Pest – OE/CED/UI4016/2016).

**References**

1. Mendes P, Marinho D, Petrica J, Silveira P, Monteriro D, Cid L. Translation and Validation of the Movement Imagery Questionnaire – 3 (MIQ - 3) with Portuguese Athletes. Motricidade. 2016, 12, 149-158.

2. Martini R, Carter M, Yoxon E, Cumming J, Ste-Marie M. Development and validation of the Movement Imagery Questionnaire for Children (MIQ-C). Psychology of Sport and Exercise. 2016, 22: 190-201.

**Keywords**

Imagery, Translation and validation, Children, Exploratory factor analysis.

### P75 New emerging point-of-care platforms for Clostridium difficile testing

#### Isabel Andrade^1^, Chantal Fernandes^2,3^, Teresa Gonçalves^2,3^

##### ^1^Coimbra Health School, Polytechnic Institute of Coimbra, 3046-854 Coimbra, Portugal; ^2^Institute of Microbiology, Faculty of Medicine, University of Coimbra, 3004-504 Coimbra, Portugal; ^3^Center for Neuroscience and Cell Biology, University of Coimbra, 3004-504 Coimbra, Portugal

###### **Correspondence:** Isabel Andrade (imandrade@estescoimbra.pt)

**Background**

Since 2000, the incidence and severity of *Clostridium difficile* (*C. difficile*) infections have increased, justifying the need for expedite, sensitive and specific methods of diagnosis. The existing rapid diagnostic tests vary widely in terms of clinical usefulness, which is evaluated on its sensitivity, specificity, turnaround time (TAT), cost, and availability. Besides that, there is no generally accepted gold standard or single optimal approach for *C. difficile* testing. This has challenged various stakeholders to develop point-of-care (POC) platforms with the best test performance characteristics, easy to use, and with rapid TAT, a critical element of POC testing to improve the clinical management of this infectious disease. POC testing can be done at home, at the primary care level, by hospital staff in emergency or operating rooms, intensive care units, as well as in extreme environments, such as remote or low-resource settings, or in conditions following emergency crises or natural disasters.

**Objective**

To review current evidence regarding emerging POC platforms for *C. difficile* testing.

**Methods**

PubMed database was searched using keywords relevant to POC testing of *C. difficile*, since 2000, yielding a total of 10 articles included out of 17 initially identified/selected for full review.

**Results**

The findings show that during the last decade extensive research efforts were underway to develop stand-alone platforms suitable for POC testing of *C. difficile* infections. Multistep algorithms using the polymerase chain reaction test for *C. difficile* toxin gene(s) have the best test performance characteristics, and a trend seems to exist in favour of molecular tests in detriment of immunoassays, in POC platforms. A common feature to all POC devices is the rapid TAT, within seconds-minutes to a few hours, which is crucial for *C. difficile* infection management. Some of these POC devices enable to run either multiplex tests on a single sample, or multiple samples. Although the majority of these stand-alone POC platforms for *C. difficile* testing are still prototypes, they may be looked at as a step towards more rapid, miniaturized, portable and easier to use test devices with the potential to affect healthcare decisions at its earliest stage.

**Conclusions**

In conclusion, research efforts show an increasing number of technologies evolving for the development of POC platforms for *C. difficile* testing.

**Keywords**

Clostridium difficile, Point-of-care testing, Turnaround time, Stand-alone platform.

### P76 Contributions for the validation of the Portuguese version of the Cohen-Mansfield Agitation Inventory (CMAI)

#### Rafael Alves^1^, Daniela Figueiredo^2,3^, Alda Marques^2,4^

##### ^1^Psychiatric Hospital Centre of Lisbon, 1749-002 Lisbon, Portugal; ^2^School of Health Sciences, University of Aveiro, 3810-193 Aveiro, Portugal; ^3^Institute of Biomedicine, University of Aveiro, 3810-193 Aveiro, Portugal; ^4^Center for Health Technology and Services Research, University of Aveiro, 3810-193 Aveiro, Portugal

###### **Correspondence:** Rafael Alves (susanapupocorreia@gmail.com)

**Background**

Dementia represents one of the greatest health challenges due to its incidence and frequency, costs and impacts on the individual, family and society. The Diagnostic and Statistical Manual of Mental Disorders – V (DSMV) considers that the behavioural changes, *i.e.*, the non-cognitive aspects of dementia, should be diagnosed, however, this is often not common practice. The identification and quantification of Behavioural and Psychological Symptoms of Dementia (BPSD) can be objectively assessed using the Cohen-Mansfield Agitation Inventory (CMAI). CMAI is a reliable and valid instrument used in clinical and research practice [3], however, it has never been validated for European Portuguese.

**Objective**

To contribute for the adaptation and validation of the CMAI for the European Portuguese population.

**Methods**

The study was conducted in two phases. The first phase consisted of the translation and cultural and linguistic validation of the CMAI according to the International Society for Pharmacoeconomics and Outcomes Research (ISPOR). The following methodology was used a) Translation, b) Reconciliation, c) Retroversion, d) Harmonization, e) Cognitive testimony/analysis and e) Spelling review. In phase 2, the internal consistency was calculated using the Cronbach's alpha. The intra- and inter-rater reliability was determined using the Intraclass Correlation Coefficient (ICC), using the intra-rater (1,1) and the inter-rater (2,1) equations. A factorial exploratory analysis of the construct validity was conducted with 101 people with dementia (83.3 ± 8.0 years; n=83; 82.2% female). Statistical analysis was performed with the Kaiser-Meyer-Olkin (KMO) test, on the behaviours manifested by more than 10% of the sample and only items weighing more than 0.4 were included in the extracted factors.

**Results**

The Portuguese version of CMAI revealed good reliability inter-rater (ICC> 0.4 for 22/29 items) and excellent intra-rater (ICC> 0.75 for 21/29 items) reliability and good internal consistency (α = 0.694). The factorial exploratory analysis was applied to 21 items that meet the criterion. An association in three factors “non-aggressive physical behaviour” (67.3%), “aggressive behaviour” (66.3%) and “verbal agitation behaviour” (63.4%) was found with a reasonable quality (KMO = 0.664) and reasonable internal consistency values (0.754; 0.633; 0.714).

**Conclusions**

This study contributed to the availability of a measurement instrument for the European Portuguese, that can be used in clinical or research contexts, with people with dementia. As a future study it is suggested to analyse the remaining validation processes (criterion and confirmatory factor analysis).

**Keywords**

Dementia, Instrument, BPSD, Agitation.

### P77 Analysis of the activations of the Intra-Hospital Emergency Team

#### Marisa J Cardo^1^, Pedro Sousa^2,3^

##### ^1^Centro Hospitalar de Leiria, 2410-197 Leiria, Portugal; ^2^School of Health Sciences, Polytechnic Institute of Leiria, 2411-901 Leiria, Portugal; ^3^Center for Innovative Care and Health Tecnhology, Polytechnic Institute of Leiria, 2411-901 Leiria, Portugal

###### **Correspondence:** Marisa J Cardo (marisacardo@gmail.com)

**Background**

The safety of patients is extremely important, therefore the Intra-Hospital Emergency Team (IHET) has emerged to respond to situations of clinical deterioration of hospitalized patients. When the patient presents deterioration of his clinical condition, it requires being examined in a timely manner by a team that provides the highest level of care. That seems to be the better way to avoid the occurrence of critical events, such as mortality, cardiovascular arrest and unplanned admission in intensive care units.

**Objectives**

This study aims to determine the characteristics of the IHET activations of Centro Hospitalar de Leiria (CHL).

**Methods**

This exploratory study analysed the registrations of the activations occurred in the last half of 2011, totalling 325 records. Sociodemographic and clinical characteristics of the patients, and the characteristics of the activations were analysed using the chi-square test and ANOVA.

**Results**

This study showed IHET activations mainly for male patients (56%), with a mean age of 74.48 ± 13.34 years, with an admission diagnosis related to respiratory diseases (34%) and with the main activation criterion of worried professional (33%). Despite the lack of records on the presence of previous signs of clinical deterioration, it was found that only 27% of patients presented them. The relationship between the presence of previous signs of deterioration and the result of the activation stands out (χ^2^ = 18.695; p ≤ 0.001).

**Conclusions**

It was found that the IHET was activated in a timely manner by nurses who were knowledgeable of their patients and with the capacity to predict critical events, which enabled the best possible outcomes.

**Keywords**

Hospital rapid response team, Nursing, Emergency situation, Clinical deterioration.

### P78 Virtual assistant to facilitate self-care of older people with type 2 diabetes: preliminary study protocol

#### Mara P Guerreiro^1^, Adriana Henriques^1^, Isabel CE Silva^1^, Anabela Mendes^1^, Ana P Cláudio^2^, Maria B Carmo^2^, João Balsa^2^, Susana Buinhas^3^, Nuno Pimenta^4^, Afonso M Cavaco^5^

##### ^1^Escola Superior de Enfermagem de Lisboa, 1600-190 Lisboa, Portugal; ^2^Instituto de Biossistemas e Ciências Integrativas, Universidade de Lisboa, 1749-016 Lisboa, Portugal; ^3^Faculdade de Ciências, Universidade de Lisboa, 1749-016 Lisboa, Portugal; ^4^Escola Superior de Desporto de Rio Maior, Instituto Politécnico de Santarém, 2040-413 Rio Maior, Portugal; ^5^Faculdade de Farmácia, Universidade de Lisboa, 1649-003 Lisboa, Portugal

###### **Correspondence:** Mara P Guerreiro (mara.guerreiro@esel.pt)

**Background**

More than a quarter of people aged 60-79 years is diabetic [1]. The management of type 2 diabetes (T2D) includes diet, physical activity and, often, medicines; it requires daily self-care and constant lifestyle-related choices [2]. It has been estimated that glycaemic control is achieved in less than 50% of T2D patients due to low self-care behaviour [2]. Sustained hyperglycaemia causes complications and premature death, as well as significant costs [1]. Improving self-care and T2D management is therefore crucial.

Technology-based interventions, such as text messages, have successfully been used in T2D management; nevertheless, challenges remain, such as acceptability to users and attrition [3,4]. Relational agents, which are computational artefacts designed to establish rapport and trust by simulating face-to-face counselling in long-term interactions, may overcome such challenges. In particular, they have shown acceptability to older and low literacy patients in other contexts [5,6]. There is a paucity of research on the use of relational agents in older people with T2D.

**Objective**

To develop a viable prototype of a relational agent software to assist older T2D patients in self-care and to test its use in this patient group.

**Methods**

This is a mixed-method study, grounded in the Medical Research Council framework to develop and evaluate complex interventions [7]. The first stage - development of the optimal intervention – will comprise the definition of pre-requisites, content production (e.g. dialog creation guided by behaviour-change theories) and empowering existing virtual humans [8] with artificial intelligence. Users and health care professionals will be involved iteratively. The software is expected to run in a tablet, independently from internet connections, targeting adherence to physical activity, diet and medication-use, without assistance from health care professionals. The second stage will be a non-randomised, non-controlled feasibility trial. Eligible subjects enrolled in diabetes nursing consultations in primary care will be invited to participate. Main outcome measures include software use (timing and frequency, usability, patient satisfaction), reported self-care and reported medication adherence. Acceptability will be researched through focus groups. In both stages data will be analysed with the aid of SPSS; qualitative data will be transcribed verbatim and thematically analysed with NVIVO software.

**Results**

Data collection will start once ethical approval is obtained.

**Conclusions**

The study is expected to yield a software prototype to facilitate self-care of older T2D patients, with potential to become an effective, scalable and sustainable intervention.

**Acknowledgements**

The study is funded by Compete 2020 and FCT (024250, 02/SAICT/2016).

**References**

1. Sociedade Portuguesa de Diabetologia. Diabetes: Factos e Números - o ano de 2015.2016.

2. García-Pérez L-E, Alvarez M, Dilla T, Gil-Guillén V, Orozco-Beltrán D. Adherence to therapies in patients with type 2 diabetes. Diabetes Ther. 2013;4:175–94.

3. Arambepola C, Ricci-Cabello I, Manikavasagam P, Roberts N, French DP, Farmer A. The Impact of Automated Brief Messages Promoting Lifestyle Changes Delivered Via Mobile Devices to People with Type 2 Diabetes: A Systematic Literature Review and Meta-Analysis of Controlled Trials. J. Med. Internet Res. 2016;18.

4. Stellefson M, Chaney B, Barry AE, Chavarria E, Tennant B, Walsh-Childers K, et al. Web 2.0 chronic disease self-management for older adults: A systematic review. J. Med. Internet Res. 2013;15:e35.

5. Bickmore T, Caruso L, Clough-Gorr K, Heeren T. “It”s just like you talk to a friend’ relational agents for older adults. Interact. Comput. 2005;17:711–35.

6. Bickmore TW, Pfeifer LM, Paasche-Orlow MK. Health Document Explanation by Virtual Agents. In: Pelachaud C, Martin J-C, André E, Chollet G, Karpouzis K, Pelé D, editors. Intell. virtual agents. Springer; 2007:183–96.

7. Möhler R, Köpke S, Meyer G. Criteria for Reporting the Development and Evaluation of Complex Interventions in healthcare: revised guideline (CReDECI 2). Trials; 2015;16:1–9.

8. Cláudio AP, Carmo MB, Pinto V, Guerreiro MP. Virtual Humans for Training and Assessment of Self- medication Consultation Skills in Pharmacy Students. Proc. IEEE ICCSE 2015- 10th Int. Conf. Comput. Sci. Educ. 2015;175–80.

**Keywords**

Diabetes, Self-care, Relational agents, Technology, Elderly

### P79 Use of maggot therapy in a hard-to-heal wound care unit: application and home follow-up protocol

#### Rodrigo C Ferrera^1^, María AF Fernández^1^, Pablo G Molina^2^, Evelin B Lopez^2^, Alberto P Paredes^3^, Adán A Ordiales^4,5^

##### ^1^Nursing Department, University of Las Palmas de Gran Canaria, 35015 Canary Islands, Spain; ^2^Nursing Department, University of Valencia, 46010 Valencia, Spain; ^3^Hospital Clínico Universitario, 46010 Valencia, Spain; ^4^Hard-to-heal Nursing Care Unit, Hospital Clínico Universitario, 46010 Valencia, Spain; ^5^Nursing Department, University of Valencia, 46010 Valencia, Spain

###### **Correspondence:** Alberto P Paredes (alpe1591@gmail.com)

**Background**

Larval debridement therapy or “maggot” therapy or biosurgery is described as the use of worms for the removal of non-viable tissue. Debridement is achieved thanks to the action of proteolytic enzymes, which are secreted by these larvae, liquefying the protein material on the surface of the wound, which is subsequently used by the larvae as a nutritive material. Healthy tissues are not affected, making this method the most selective among those available. In addition, this therapy helps to fight infection and helps normalization and closure of injuries. Larval debridement has been successfully used in a large range of chronic hard-to-heal wounds with presence of non-viable tissue of many aetiologies such as pressure ulcers, venous or ischemic wounds or diabetic foot ulcers [1-5]

**Objective**

To ensure continuity in care in the aim of obtaining the best results from the use of larval debridement therapy, while allowing the administration of treatment in any healthcare context, especially at the patient's own home.

**Methods**

The procedure for the use of Maggot therapy has been elaborated and coordinated by the Nursing Unit of Ulcers and Complex Wounds of the Hospital Clínico de Valencia. This protocol includes the administrative process, selection of the size of the dressings and the procedure of application and care of the therapy itself. At the same time, an information brochure was prepared for family members, patients and care professionals with information about daily surveillance and application of the therapy.

**Results**

This protocol is in the process of being implemented, having been applied in several patients with different aetiology of hard-to-heal wounds within home follow-up care with successfully results.

**Conclusions**

The implementation of a protocol for the use of Maggots debridement therapy seems to be effective in ensuring continuity in the treatment and follow-up of patients with difficult healing wounds in a home care context.

**References**

1. Ballester Martínez L, Martínez Monleón E, Serra Perucho N, Palomar Llatas F. Utilización de la Terapia Larval en Heridas Desvitalizadas: Revisión Bibliográfica [Use of Maggots Therapy in Necrotic Wounds: Literature Review]. Enf Derm. 2016, 10: 27-33.

2. McCaughan D, Cullum N, Durnville J, VenUS II Team. Patient2s perceptions and experiences of venous leg ulceration and their attitudes to larval therapy: an in-depth qualitative study. Health Expect. 2015, 18(4): 527-541.

3. Mudge E, Price P, Walkley N, Harding KG. A randomized controlled trial of larval therapy for the debridement of leg ulcers: results of a multicenter, randomized, controlled, open, observer blind, parallel group study. Wound Repair Regen. 2014, 22(1): 43-51.

4. EWMA Document. Larvae debridement therapy. J Wound Care. 2013, 22(1): 522-525.

5. Whitaker IS, Twine C, Whitaker MJ, Welck M, Brown CS, Shandall A. Larval trerapy from antiquity to the present day: mechanisms of actions, clinical applications and future potential. Postgrad Med J. 2007, 83(980): 409-413.

**Keywords**

Maggot therapy, Wound care, Debridement, Home-care settings, Protocole.

### P80 Adverse reactions and dietary supplements

#### Andreia Barros^1^, Cláudia Pinho^2^, Ana I Oliveira^2^, Rita F Oliveira^2,3^, Agostinho Cruz^2^

##### ^1^Escola Superior de Saúde, Instituto Politécnico do Porto, 4200-072 Porto, Portugal; ^2^Centro de Investigação em Saúde e Ambiente, Escola Superior de Saúde, Instituto Politécnico do Porto, 4200-072 Porto, Portugal; ^3^Secção Autónoma de Ciências da Saúde, Universidade de Aveiro, 3810-193 Aveiro, Portugal

###### **Correspondence:** Rita F Oliveira (rfo@ess.ipp.pt)

**Background**

Over the last years, the use of dietary supplements has increased substantially [1]. Although these products are considered as safe and can be beneficial, there are risks associated with some. Manufacturers are not required to demonstrate their safety and efficacy, so it is essential that consumers have good knowledge about dietary supplements [2]. The attribution of injury to a specific supplement can be challenging, especially because of the multiple ingredients, the variability in quality and content, as well as the vast underreporting of adverse reactions [3].

**Objective**

This study aims to identify the main adverse reactions and knowledge on reporting adverse events associated to the use of dietary supplements, by the population of Porto (Portugal).

**Methods**

A descriptive, cross-sectional study was performed through an anonymous, confidential and voluntary questionnaire to 404 adult participants from the municipality of Porto (Portugal). Data were analysed quantitatively using SPSS version 24.0.

**Results**

Of the 404 participants, 54.7% (221) were females and 45.3% (183) were males. Results revealed that 55.9% (226) of the participants were users of dietary supplements and the common reasons for consuming supplements were to improve memory, concentration and reduce fatigue. Of the 226 consumers of supplements, only 1.3% (3) identified adverse reactions after taking supplements with multivitamins and used for insomnia and anxiety. Of the 404 participants, 21.5% (87) referred to know that is possible to report an adverse reaction associated to dietary supplements, in Portugal, since 2014. Also, only 8.9% (36) referred to know which entity is responsible for the adverse reactions associated to supplements, and of these 36 participants only 5.6% (2) had correctly answered the name of the entity - Direção Geral da Alimentação e Veterinária (DGAV).

**Conclusions**

The findings of this survey indicate the need to provide knowledge on reporting adverse events associated with dietary supplements use. It is essential to provide adequate information to facilitate better understanding of the risks associated with the use of these products.

**References**

1. Kantor ED, Rehm CD, Du M, White E, Giovannucci EL. Trends in dietary supplement use among US adults from 1999–2012. JAMA. 2016, 316:1464–1474.

2. Axon DR, Vanova J, Edel C, Slack M. Dietary Supplement Use, Knowledge, and Perceptions Among Student Pharmacists. Am J Pharm Educ. 2017, 81(5): 92.

3. Felix TM, Karpa KD, Lewis PR. Adverse Effects of Common Drugs: Dietary Supplements. FP Essent. 2015, 436:31-40.

**Keywords**

Dietary supplements, Risks, Adverse reactions reporting, DGAV.

### P81 Urinary tract infections and dietary supplements: counselling in pharmacy

#### Marta Novais^1^, Cláudia Pinho^2^, Ana I Oliveira^2^, Rita F Oliveira^2,3^, Agostinho Cruz^2^

##### ^1^Escola Superior de Saúde, Instituto Politécnico do Porto, 4200-072 Porto, Portugal; ^2^Centro de Investigação em Saúde e Ambiente, Escola Superior de Saúde, Instituto Politécnico do Porto, 4200-072 Porto, Portugal; ^3^Secção Autónoma de Ciências da Saúde, Universidade de Aveiro, 3810-193 Aveiro, Portugal

###### **Correspondence:** Rita F Oliveira (rfo@ess.ipp.pt)

**Background**

Urinary tract infections (UTIs) are some of the most common bacterial infections [1]. Treatment usually involves antibiotics, and recurrence is a major concern [2]. Therefore, identifying new and effective strategies, like the use of botanical dietary supplements, to control UTIs is a high priority. It is also important to provide health professionals with adequate knowledge related to the use of dietary supplements and other complementary and/or alternative medicines.

**Objective**

This study aims to evaluate the counselling practices by pharmacy professionals, working in Barcelos (Portugal), related to the use of botanical dietary supplements, in the prevention and/or treatment of urinary infections.

**Methods**

A descriptive, cross-sectional study was performed through an anonymous, confidential and voluntary questionnaire to a convenience sample of 108 pharmacy professionals from Barcelos (Portugal). Data were analysed using SPSS version 24.0.

**Results**

Of the 108 participants, 67.6% were females and 32.4% were males. The results showed that 96.3% of the professionals usually advise the use of dietary supplements for the prevention and/or treatment of lower urinary tract infections. The common reasons to recommend supplements include the efficacy and safety of these products, and the lower price. It was also observed that 64.8% of pharmacy professionals consider their knowledge sufficient to recommend dietary supplements for the prevention and/or treatment of urinary infections. Regarding the recommendations by professionals for the prevention of urinary tract infections, the products containing *Vaccinium macrocarpon* L. were the most recommended. On the other hand, products containing *Arctostaphylos uva ursi* L were the most recommended for the treatment of urinary tract infections. In general, the main plants sold by pharmacy professionals for the control of urinary tract infections included *Vaccinium macrocarpon L.*, *Arctostaphylos uva ursi L.*, *Vaccinium myrtillus L.*, *Equisetum arvense L.* and *Hibiscus sabdariffa L..*

**Conclusions**

The findings of this study revealed that pharmacy professionals recommend dietary supplements for control of urinary tract infections and consider their knowledge sufficient to properly advise these products. Because evidence on the efficacy of dietary supplements is often scarce or controversial, providing consistent recommendations about these products to their patients can be challenging for healthcare professionals.

**References**

1. Stamm WE, Norrby SR. Urinary tract infections: disease panorama and challenges. J Infect Dis. 2001, 183 (Suppl 1):S1-S4.

2. Guay DR. Contemporary management of uncomplicated urinary tract infections. Drugs. 2008, 68(9):1169-205.

**Keywords**

Urinary Tract Infections, Botanical Dietary supplements, Counseling, Pharmacy Professionals.

### P82 Nurse’s intervention – end of life nutrition approach protocol

#### Tânia S Afonso^1^, Filipa Veludo^1^, Patrícia P Sousa^1^, Dulce Oliveira^2^

##### ^1^Instituto de Ciências da Saúde, Escola de Enfermagem, Universidade Católica Portuguesal, 1649-023 Lisboa, Portugal; ^2^Unidade de Medicina Paliativa, Hospital de Santa Maria, Centro Hospitalar Lisboa Norte, 1649-035 Lisboa, Portugal

###### **Correspondence:** Tânia S Afonso (tafonso3@gmail.com)

**Background**

To know that nutrition in the present society is increasingly associated with life maintenance and comfort, helps us to understand the complexity of this subject when approached the end of life. Artificial nutrition remains controversial in a palliative context, given the questioning about the quality of life that offers [1]. Protocols help nurses in the decision-making process and increasing their competences.

**Objective**

To present an end-of-life nutrition approach protocol for palliative care.

**Methods**

This study is the result of three integrative literature reviews that intended to measure: *which nursing interventions promote end of life nutrition in people without artificial nutrition criteria?*; *what are the evaluation criteria for the end-of-life person for the nurse’s decision-making of start, don’t start or suspending artificial nutrition?*; *does the nurse’s interventions towards the end-of-life reduce the risk of therapeutic obstinacy associated with artificial nutrition*? Based in Buckman & Spikes Communication Protocol [2], the results were integrated in a protocol form and submitted to the opinion of 13 experts, from 18th October to 6th November 2017, and the respective changes were made. Inclusion criteria of experts were: being health professionals; palliative care experience and/or work development in nutrition subjects.

**Results**

Our experts have on average 37 years old; 10 carry out their activity in Palliative settings, 8 of these have advanced training in Palliative Care. Our protocol considers: I) setting - preparing the environment; II) perception - prior knowledge of the person/family information about nutrition, preferences and considerations regarding the future commitment of feeding and active listening, understanding what the person/family wants to know, especially as to the meaning of nutrition, what that moment represents and invite them to address the subject; III) knowledge - provide adequate information in phases, contextualizing the present symptoms in the disease process (prognosis) and discuss the evaluation criteria before starting artificial nutrition; IV) emotions – attend to the emotions and provide realistic hope; V) strategy – interventions from the patient’s needs are presented in an algorithm form, promoting oral feeding as long as possible. In all process, the person and family autonomy in decision making is preserved. At each step, we identified an element to avoid in the communication process [1,2].

**Conclusions**

The set of nurse’s interventions in end-of-life nutrition approach systematizes the elements to be considered in decision-making and guarantees the importance of nurses' contribution in risk reduction of therapeutic obstinacy.

**References**

1. Alves P. Intervenção do Enfermeiro que Cuida da Pessoa em Fim de Vida com Alterações do Comer e Beber. Pensar Enfermagem. 2013, 17(1): 17-30;

2. Baile W [et al.]. SPIKES — A Six-Step Protocol for Delivering Bad News: Application to the Patient with Cancer. Oncologist. 2000, 5(4):302-311.

**Keywords**

Nursing, Nutrition, Spikes protocol, Communication, Palliative care.

### P83 Associated factors with polymedication in elderly accompanied in the health strategy of the family of the city of Palhoça, Santa Catarina, Brazil

#### Fabrícia M Almeida^1^, Mônica R Moraes^1^, Giovanna G Vietta^1^, Roberta TS Shirasaki^2^, Ísis M Sousa^2^, Pedro F Simão^1^, Bárbara O Gama^1^, Fabiana O Gama^1^, Paulo F Freitas^1^, Márcia Kretzer^1^

##### ^1^Universidade do Sul de Santa Catarina, 88704-900, Tubarão, Santa Catarina, Brasil; ^2^Unidade Básica de Saúde Ponte do Imaruim, Palhoça, Santa Catarina, 88130-300, Brasil

###### **Correspondence:** Fabrícia M Almeida (fabricia_dani@hotmail.com)

**Background**

Aging implies in an increase in the number of morbidities, which requires medical treatment and may result in the use of multiple medications. Polymedication is associated with a risk of loss of quality of life, negative health outcomes and a risk of dependent mortality.

**Objective**

To evaluate the factors associated to polymedication in the elderly followed up in the Family Health Strategy of the city of Palhoça, Santa Catarina, Brazil.

**Methods**

A cross-sectional study carried out in the elderly accompanied by two Basic Health Units in Palhoça. Data collected between august and November 2017, using a questionnaire with sociodemographic and clinical data and the Geriatric Depression Scale (GDS). Polymedication was defined as the use of 5 or more drugs on an ongoing basis. Analysis by SPSS 20.0, with chi-square and Fisher's exact test, Prevalence Ratio (PR), Confidence Interval (CI) 95%, p < 0.005. The project was approved by the Research Ethics Committee of the Southern University of Santa Catarina.

**Results**

135 individuals were interviewed, with a mean age of 69.9 years and a standard deviation of 8 years, ranging from 60 to 97 years. The highest frequency was female (70.0%), white (81.3%), married (50.4%), and widowed (28.1%). About the occupation, 80.7% were retired and 54.6% received up to 250 Euros monthly. The schooling was predominantly until elementary school (74.6%), with 9.7% being illiterate, 4.5% with higher education and 0.7% with post-graduation. The majority (63.7%) did not practice physical exercise, 6.8% were smokers. The frequency of depression was 39.6%, with 5.2% categorized as severe depression. The use of drugs among the interviewees ranged from none (4.4%) to 25 different medications per day (0.7%), with 42.7% using 5 or more medications. Among those who reported the use of medication, 63% used antihypertensives, 32.6%, anti-depressants, 37.8%, anti-diabetics and 14.8% analgesics. Self-medication was identified in 23.9%. Polymedication had a significant association (p < 0.001) with the presence of arterial hypertension (RP = 2.85, CI 1.59-5.09), Diabetes Mellitus (RP = 2.31, CI 1.58-3.36), arthritis (RP = 1.60, CI 1.10-2.33), depression (RP = 1.87, CI 1.30-2.69), and cardiovascular diseases (RP = 2.29, CI 1.61 -3.26).

**Conclusions**

Polymedication in the elderly presented high prevalence and was associated with the presence of cardiovascular, endocrine, joint and depression diseases. A presence of symptoms of depression was present in 39.6% of the elderly.

**Keywords**

Polymedication, Associated factors, Comorbidity, Elderly.

### P84 Lack of Vitamin D in elderly individuals: case study – Figueira da Foz

#### Ana Azul^1^, Cristina Santos^1^, António Gabriel^2^, João P Figueiredo^3^, Ana Ferreira^1^

##### ^1^Department of Environmental Health, Coimbra Health School, 3046-854 Coimbra, Portugal; ^2^Department of Laboratory Biomedical Sciences, Coimbra Health School, 3046-854 Coimbra, Portugal; ^3^Department of Complementary Sciences, Coimbra Health School, 3046-854 Coimbra, Portugal

###### **Correspondence:** Ana Azul (ana.sofia.azul@hotmail.com)

**Background**

Nowadays, the increase of aging among world’s population is becoming a reality, due to the decrease of the fertility and mortality rates and to the consequent increase of the average life expectancy [1]. This population aging brings different health, economic and social needs, turning elderly dependent and making them living in an institutionalized way [2]. In turn, the institutionalization favours the decline of their physical and cognitive functions, which consequently, weakens the old-aged. For all this, it is very likely that this age group will have D vitamin lack, being considered a serious public health problem at a worldwide level. Therefore, its supplementation must be considered, since this age group has the tendency of staying shortly exposed to solar radiation, has little mobility and their bodies manifest a reduction of the ability of synthesis of this hormone [3-5].

**Objective**

This investigation intends to evaluate de existence of the lack of D vitamin in old-aged people who may be institutionalized and old-aged people in ambulatory from the region of Figueira da Foz.

**Methods**

Application of a questionnaire and collection of blood samples.

**Results**

With the purpose of evaluating the concentrations of 25(OH)D for each one of the studied groups, it was verified that the non-institutionalized group showed higher 25(OH)D values, when to compared to the institutionalized group. This result has shown to be statistically significant, with a p-value of 0.003. On the other hand, other variables were compared, as for example, feeding, sun exposure, chronic diseases and intake of vitamin supplements, to try to understand if they had any influence on the 25(OH)D levels. Nevertheless, concerning these parameters, no great differences were verified, because p-values were always higher than 0.005.

**Conclusions**

With this study, we conclude that the geriatric population presents a high lack of vitamin D, both the institutionalized group (although with higher values of 25(OH)D) and the ambulatory group.

**References**

1. Nogueira P, Afonso D, Alves MI, Vicêncio PO, Silva Jd, Rosa MV, et al. Portugal Idade Maior em números, 2014: A Saúde da População Portuguesa com 65 ou mais anos de idade. 2014. p. 223

2. Bárrios MJ, Fernandes AA. A promoção do envelhecimento ativo ao nível local: análise de programas de intervenção autárquica. Revista Portuguesa de Saúde Pública. 2014;32(2):188-96.

3. Jalal S, Khan NU. Frequency of Vitamin D Deficiency in Elderly Patients Visiting Tertiary Care Hospital in a Low Income Country. 2014;40:44-53.

4. Lanske B, Razzaque MS. Vitamin D and aging: old concepts and new insights. The Journal of nutritional biochemistry. 2007;18(12):771-7.

5. Zumaraga MP, Medina PJ, Rectoa JM, Abrahanc L, Azurinc E, Tanchoco CC, et al. Targeted next generation sequencing of the entire vitamin D receptor gene reveals polymorphisms correlated with vitamin D deficiency among older Filipino women with and without fragility fracture. 2017:98- 108.

**Keywords**

Aging, Vitamin D, Vitamin supplementation, Public health.

### P85 Error prevention in nursing: strategies for a safety culture

#### Teresa Vinagre^1^, Rita Marques^2^

##### ^1^Instituto de Ciências da Saúde, Universidade Católica Portuguesa, 1649-023 Lisbon, Portugal; ^2^Escola Superior de Saúde da Cruz Vermelha Portuguesa, 1300-906 Lisbon, Portugal

###### **Correspondence:** Rita Marques (ritamdmarques@gmail.com)

**Background**

The Safety culture is becoming more and more linked to quality and excellence of care, being a crucial factor for health error prevention. Nursing assumes a crucial role in patient safety, being at forefront of patient care, and should protect patient interests and assure consolidation of strategies that guarantee safety.

**Objective**

Identify what are the strategies for an effective safety culture and to prevent errors in nursing.

**Methods**

Literature Review, following the recommended methodology of the Cochrane Centre, guided by the investigation question: *What are the strategies for an effective safety culture and to prevent errors in nursing?*

The study includes the analysis of articles found in EBSCO (CINAHL, MEDLINE, Nursing & Allied Health Collection, Cochrane Database of Systematic Reviews), in B-ON and in SCIELO, with the following descriptors: Nursing; Patient Safety; Errors, with the timeframe between 2012 and 2017. The sample resulted in 12 articles.

**Results**

The Team Work [1-5,8-12] and communication [1-6,8-11] where referenced in 75.0% of the studies as vital measures in an effective safety culture and error preventing in nursing, 66.7% reinforce the importance of error notification [1,2,4,7-11], 58.3% defend that continuous improvement/training is essential [1,2,4,5,9,11,12], 33.3% consider global safety perception [4,10-12] and the importance of trust in superiors and their compromise with their subordinates [4,10-12] as effective methods, 25.0% stand out the importance of error feedback to health professionals [5,10,11]. As an ending, less than 10% of the analysed studies refer work conditions [4], critical reflexion [6], supervision and existence of standards [3], conflict management [3], and assume the person as the centre of health care [3] as important strategies.

**Conclusions**

Many are the strategies used for an effective safety culture and error prevention in nursing, being the most significant, team work and communication, followed by error notification and continuous improvement/training. Besides the aspects mentioned above, in every article analysed two crucial factors where identified: the direct relation between the existence of a safety culture and the decrease of advert events in health and the need to make the system safer, instead of trying to change human conditions, as a mean to ensure safety and quality care provision.

**References**

1. MarinhoM, RadünzV, TourinhoF, RosaL, MisiakM. IntervençõesEducativas e seu Impacto na Cultura de Segurança: Uma Revisão Integrativa. Enferm Foco. 2016. 7 (2): 72-77.

2. Mendes C, Barroso, F. Promover uma cultura de segurança em cuidados de saúde primários. Rev Port Saúde Pública. 2014. 32 (2): 197-205.

3. SilvaE, RodriguesF.Segurançadodoenteeosprocessossociaisnarelação com enfermeiros em contexto de bloco operatório. Cultura de los Cuidados. 2016. 45: 134-145.

4. Paese F, Sasso G. Cultura de segurança do paciente na atenção primária à saúde. Texto e Contexto Enferm. 2013. 22 (2): 302-310.

5. MinuzzA, SalumN, Locks, M.Avaliaçãodaculturadesegurançadopaciente em terapia intensiva na perspectiva da equipe de saúde. Texto e Contexto Enferm. 2016. 25 (2): 1-9.

6. Araújo M, Filho W, Silveira R, Souza J, Barlem E, Teixeira N. Segurança do paciente na visão de enfermeiros: uma questão multiprofissional. Enferm Foco. 2017. 8 (1): 52-56.

7. Correia T, Martins M, Forte E. Processes developed by managers regarding the errors. Rev Enferm Referência. 2017. IV (12): 75-84.

8. Cavalcante A, Cavalcante F, Pires D, Batista E, Nogueira L. Cultura de segurança na percepção da enfermagem: Revisão integrativa. Rev Enferm UFPE On Line. 2016. 10 (10): 3890-3897.

9. Wang X, Liu K, You L, Xiang J, Hu H, Zhang L, Zheng J, Zhu X.The relationship between patient safety culture and adverse events: A questionnaire survey. Int J Nurs Stud. 2014. 51: 1114-1122.

10. Noord I, Wagner C, Dyck C, Twisk J, Bruijne M. Is culture associated with patient safety in the emergency department? A study of staff perspectives. Int J Qual Health Care. 2013. 26 (I): 64-70.

11. Ballangrud R, Hedelin B, Hall-Lord M. Nurses’ perceptions of patient safety climate in intensive care units: A cross-sectional study. Intensive Crit Care Nurs. 2012. 28: 344-354.

12. Feng X, Bobay K, Krejci J, McCormick B. Factors associated with nurses’ perceptions of patient safety culture in China: a cross-sectional survey study. J Evid Based Med. 2012. 50-56.

**Keywords**

Nursing, Patient Safety, Errors.

### P86 Error notification: a strategy for a safety culture

#### Teresa Vinagre^1^, Rita Marques^2^

##### ^1^Instituto de Ciências da Saúde, Universidade Católica Portuguesa, 1649-023 Lisbon, Portugal; ^2^Escola Superior de Saúde da Cruz Vermelha Portuguesa, 1300-906 Lisbon, Portugal

###### **Correspondence:** Teresa Vinagre (teresasousasoares@gmail.com)

**Background**

Errors are an inevitable condition of the human being, and one of the biggest contributors to morbidity and mortality around the world. Error notification is a scientifically proved strategy as being one of the most effective of a safety culture [1-8], being essential for the prevention and detection of factors that contribute for the error. The Operation Room (OR) is one of the most prone health services for the occurrence of adverse events/errors [3], hence being vital the fulfilment of studies regarding this issue, to contribute to an improvement in health care and assure patient safety.

**Objective**

To determine the error notification frequency in the OR; and to characterise the safety culture of the OR.

**Methods**

Exploratory study, with a quantitively approach. A survey was conducted with 9 closed questions to 33 nurse professionals that work in a OR in a Lisbon Hospital.

**Results**

54.6% of the adverse events that caused damage to the patient were always notified by the nurses, nevertheless, none of the participants pointed this regular notification for adverse events, that could have resulted in damage for the patient, but that did not. Of the several adverse events, 55.6% of the occurred cases were not notified, being the more frequent justification for not notifying them, the lack of time to notify. A negative correlation was obtained between professional experience and the error notification frequency, being this difference statistically significant (p < 0.05). Regarding the factors that contribute the most for error occurrence in OR, all of the participants mentioned the pressure for working fast, 87.0% referred the lack of human resources, 85.2% absence of motivation, 82.6% professional inexperience and workload overcharge and 65.2% of the participants considered fails in communication, as a factor preponderant to error. Patient safety perception by the nurse professionals of the OR was evaluated as “acceptable” by the majority of the participants.

**Conclusions**

It was evident the low notification frequency of adverse events/errors, and it was found that professional experience is inversely proportional to error notification. Error notification is a central aspect of health care, particularly in the OR, hence it is fundamental to educate teams for the setting of strategies that promote a safety culture. It is important to continuously train professionals as well as work on the errors, making them a learning opportunity to prevent new errors associated to the same causes, to achieve a quality safety culture.

**References**

1. Marinho M, Radünz V, Tourinho F, Rosa L, Misiak M. Intervenções Educativas e seu Impacto na Cultura de Segurança: Uma Revisão Integrativa. Enferm Foco. 2016. 7 (2): 72-77.

2. Mendes C, Barroso, F. Promover uma cultura de segurança em cuidados de saúde primários. Rev Port Saúde Pública. 2014. 32 (2): 197-205.

3. Paese F, Sasso G. Cultura de segurança do paciente na atenção primária à saúde. Texto e Contexto Enferm. 2013. 22 (2): 302-310.

4. Correia T, Martins M, Forte E. Processes developed by managers regarding the errors. Rev Enferm Referência. 2017. IV (12): 75-84.

5. Cavalcante A, Cavalcante F, Pires D, Batista E, Nogueira L. Cultura de segurança na percepção da enfermagem: Revisão integrativa. Rev Enferm UFPE On Line. 2016. 10 (10): 3890-3897.

6. Wang X, Liu K, You L, Xiang J, Hu H, Zhang L, Zheng J, Zhu X. The relationship between patient safety culture and adverse events: A questionnaire survey. Int J Nurs Stud. 2014. 51: 1114-1122.

7. Noord I, Wagner C, Dyck C, Twisk J, Bruijne M. Is culture associated with patient safety in the emergency department? A study of staff perspectives. Int J Qual Health Care. 2013. 26 (I): 64-70.

8. Ballangrud R, Hedelin B, Hall-Lord M. Nurses’ perceptions of patient safety climate in intensive care units: A cross-sectional study. Intensive Crit Care Nurs. 2012. 28: 344-354.

**Keywords**

Nursing, Safety Culture, Error Notification.

### P87 The use of ultrasound in peripheral venous catheterization

#### Bruno Santos^1^, José Pinho^2^, Rogério Figueiredo^2^, Pedro Parreira^2^, Luciene Braga^3^, Anabela Salgueiro-Oliveira^2^

##### ^1^Hospital Privado do Algarve, 8500-322 Alvor, Portugal; ^2^Escola Superior de Enfermagem de Coimbra, 3046-851 Coimbra, Portugal; ^3^Universidade Federal de Viçosa, 36570-900 Viçosa, Minas Gerais, Brasil

###### **Correspondence:** Anabela Salgueiro-Oliveira (anabela@esenfc.pt)

**Background**

The insertion of peripheral vascular catheters (PVCs) is the most common procedure performed in clinical settings [1]. The traditional method for detection and selection of a venous access includes the use of a tourniquet, palpation, and observation. However, when veins are not visible or palpable, this may lead to successive puncture attempts, causing pain to the patient and discomfort to the nurse, which results in increased costs [2]. In this regard, nurses should consider using vascular visualization technologies that aid in vein identification and selection for difficult intravenous access [3]. However, these technological resources available today, are still underutilized in Portugal.

**Objective**

This study aims to explore an alternative method (ultrasound) for assistance of the traditional venous cannulation, in order to ensure the satisfaction of the patients and of the health professionals.

**Methods**

The search method used was the integrative literature review, which analysed relevant research that supports decision-making and the improvement of clinical practice [4]. The investigation question was formulated based on the PICO strategy: *How important is the use of ultrasound technology by the nurses in patients with PVCs insertion needs?* The search was conducted between 4 and 10 January, 2017, using the timeframe between 01-01-2011 and 31-12-2016, with the purpose of finding only primary scientific studies developed over the past 5 years, in English, Portuguese or Spanish. The search was conducted in the following databases: Medline, Cinahl, Psychology and Behavioural Sciences Collection, MedicLatina, ERIC, Business Source Complete, Library, Information Science & Technology Abstracts e Academic Search. We used two search strategies P1 and P2 with the following descriptors, respectively: Ultrasound AND Peripheral catheterization AND Cannulation; Ultrasound AND Peripheral catheterization AND ultrasound guided NOT Paediatric NOT PICC NOT Artery. We found 146 scientific articles and after reading the title, abstract and full text we retained 8 studies for analysis.

**Results**

The results found confirm the venous cannulation with assistance of ultrasonography has a higher success rate, when compared to the traditional venous cannulation. It was also possible to observe a decrease in: the numbers of attempts to puncture the vein; the time used in the procedure; the incidence of central venous catheter placement; and as a consequence, the reduction of possible complications. The patient also presented lower levels of pain and higher degrees of satisfaction.

**Conclusions**

The implementation of ultrasound in clinical settings, such as in nurses training programs, are important to perform ultrasound-guided PVCs placement and quality care.

**References**

1. Webster J, Osborne S, Rickard C, New K. Clinically-indicated replacement versus routine replacement of peripheral venous catheters. Cochrane Database Syst Rev. 2015;8. Art. No.: CD007798.

2. Aponte H, Acosta S, Rigamonti D, Sylvia B, Austin P, Samolitis T. The use of ultrasound for placement of intravenous catheters. AANA Journal. 2007;75(3):212-216.

3. Gorski L, Hadaway L, Hagle M, McGoldrick M, Orr M, Doellman, D. Infusion Nursing Standards of Practice. Journal of Infusion Nursing. 2016; 39(1S): 1-159.

4. Mendes K S, Silveira R C, Galvão C M. Revisão integrativa: Método de pesquisa para a incorporação de evidências na saúde e na enfermagem. Texto e Contexto Enfermagem.2008;17(4):758-764.

**Keywords**

Peripheral venous cannulation, Ultrasound, Nurses.

### P88 Nursing care in the person with intestinal elimination ostomy

#### Igor Pinto^1^, Silvia Queirós^1^, Célia Santos^2^, Alice Brito^2^

##### ^1^Universidade Católica Portuguesa, 1649-023 Lisbon, Portugal; ^2^Escola Superior de Enfermagem do Porto, 4200-072 Porto, Portugal

###### **Correspondence:** Igor Pinto (isp.igor@gmail.com)

**Background**

Performing an intestinal elimination stoma triggers changes in the physical, psychological, social, self-care and lifestyle of the person. The way this event is experienced is influenced by several factors. The literature suggests that a systematized nursing care, started in the preoperative period, which includes the postoperative period and continues after hospital discharge, is associated with a better level of adaptation and a higher quality of life of the person with intestinal elimination ostomy.

**Objective**

To identify the existing literature on nursing care programs and to map the respective interventions performed in the person proposed for the construction of a stoma or with an intestinal elimination stoma.

**Methods**

A literature review was performed in the Web of Science, CINAHL Plus with Full Text, CINAHL Complete and Scopus databases, based on the Joanna Briggs Institute for Scoping Reviews model, from inception to April 2017. Two independent reviewers performed the analysis of article relevance, extraction and synthesis of the data.

**Results**

A total of 1,728 articles was identified and only 17 were included for content analysis. It was not possible to find a program that contemplates all phases of perioperative and post-discharge intervention, with the studies focused essentially on one or two specific moments. Considering the interventions mentioned in the literature, the most stated were: stoma site marking; preoperative education; post-operative education; and nursing follow-up after hospital discharge. However, there is still no evidence to suggest timings, methodology and contents to guide the implementation of each of the interventions.

**Conclusions**

A systematized nursing care in the person with intestinal elimination ostomy, covering the perioperative period and follow-up after discharge, has a significant impact on the adaptation to the stoma, reduces complications, increases the perception of self-efficacy and also the quality of life. It is imperative to create and test an intervention program that contemplates all these phases and all the interventions mentioned in the literature. On the other hand, further studies should be carried out to determine the defining characteristics of these interventions, which help in the decision-making process and the nurses' performance.

**Keywords**

Ostomy, Nursing Care, Cecostomy, Colostomy, Ileostomy.

### P89 Nurses competencies in catastrophes and disaster nursing

#### Patrícia MG Godinho, Maria T Leal

##### Escola Superior de Enfermagem de Lisboa, 1600-190 Lisboa, Portugal

###### **Correspondence:** Patrícia MG Godinho (p.guerra@campus.esel.pt)

**Background**

Disasters and catastrophes are unpredictable multi-victim events, leading to a sudden demand for emergency health care. An adequate response requires multidisciplinary professionals that must be experienced and specialized in the field. There is evidence that nurses are important in a catastrophic situation as key-elements that can contribute positively in these situations, because of their broad care-giving skills, that can be applied in a variety of disaster settings, with high levels of creativity, adaptability, and leadership [1]. Nevertheless, nurses need to be competent in disaster nursing, in order to make the difference [2].

**Objective**

To review the available evidence regarding nursing competencies/interventions that improve victims’ outcomes in the context of catastrophe or multi-victim emergencies.

**Methods**

We completed an integrative review of the literature available from MEDLINE and CINAHL databases and grey literature, to answer the question: “*Which nursing competencies/interventions contribute to improve victims’ outcomes in the context of catastrophes or multi-victim emergencies*?” [3].

**Results**

Eight articles, published between 2012 and 2016, satisfied the search criteria and were analysed. The number of participants varied between 16 and 620 nurses. Most of the articles demonstrate that nurses’ competencies have gaps in this field, due to lack of knowledge, training, and simulation, both in nursing education as in working contexts. One of the studies emphasized that nurses from military hospitals have a superior knowledge and preparation in catastrophes, when compared with nurses from civil hospitals, given their military training [4]. The academic training, and the implementation of catastrophe training in the nursing curriculum is also addressed in three of the analysed articles [4–6]. The fact that hospital administrations and nursing leadership fail as promoters of training and promotion of regular exercises and simulations of disasters, is also evidenced in three articles [5,7,8].

**Conclusions**

Globally, the results of this integrative review show that most nurses don’t have enough training in disaster nursing. They are not prepared to respond adequately in a mass-causality event. The recommendations are that both, in academic fields or at work contexts, regular training and simulations should be part of disaster preparedness.

**References**

1. World Health Organization, International Council of Nurses, editors. ICN Framework of Disaster Nursing Competencies. Geneva: ICN & WHO; 2009.

2. Loke AY, Fung OWM. Nurses’ Competencies in Disaster Nursing: Implications for Curriculum Development and Public Health. In: Kapur GB, Baéz AA, editors. International disaster health care: preparedness, response, resource management, and education. Oakville: Apple Academic Press; 2017. p. 185–203.

3. The Joanna Briggs Institute. Joanna Briggs Institute Reviewers’ Manual: 2014 Edition [Internet]. Adelaide: The Joanna Briggs Institute; 2014. 197 p. Available from: http://joannabriggs.org/assets/docs/sumari/ReviewersManual-2014.pdf

4. Thobaity A, Plummer V, Innes K, Copnell B. Perceptions of knowledge of disaster management among military and civilian nurses in Saudi Arabia. Australas Emerg Nurs J. 2015 Aug;18(3):156–64.

5. Labrague LJ, Yboa BC, McEnroe-Petitte DM, Lobrino LR, Brennan MGB. Disaster Preparedness in Philippine Nurses. J Nurs Scholarsh. 2016 Jan;48(1):98–105.

6. Khalaileh MA, Bond E, Alasad JA. Jordanian nurses’ perceptions of their preparedness for disaster management. Int Emerg Nurs]. 2012 Jan;20(1):14–23.

7. Baack S, Alfred D. Nurses’ preparedness and perceived competence in managing disasters. J Nurs Scholarsh. 2013 Sep;45(3):281–7.

8. Li YH, Li SJ, Chen SH, Xie XP, Song YQ, Jin ZH, et al. Disaster nursing experiences of Chinese nurses responding to the Sichuan Ya’an earthquake. Int Nurs Rev. 2017 Jun;64(2):309–17.

**Keywords**

Disaster nursing, Nursing competencies, Catastrophe, Multi-victims emergencies, Mass causality events.

### P90 Patient safety culture: the same functional typology, distinct cultures

#### Vanda Pedrosa (terapeutavandapedrosa@gmail.com)

##### School of Health Sciences, Polytechnic Institute of Leiria, 2411-901 Leiria, Portugal

**Background**

Fostering a culture of safety in health organizations should begin by evaluating the current culture. In Primary Health Care, patient safety becomes more important because a considerable proportion of safety incidents, detected in hospitals, originate from earlier levels of the system, from most of the interactions and from the largest volume of appointments of the functional units. At this level of health care, in family health units that provide accessible care, global and longitudinal follow-ups on the health process in a lifetime, enables greater health gains, and greater proximity to the patient. These are elementary health care units, based on multi-professional teams, composed by doctors, nurses and administrative staff. Still, many are on very different levels in terms of safety culture of the patient, although in all, the patient wants to have security.

**Objective**

Describe the patient's safety culture of health professionals from two family health units (USF), belonging to the same Health Centre, to understand the similarities and/or differences between them.

**Methods**

Qualitative study, with a semi-structured interview (to GPs and nurses), at two USFs, models A and B, of one Health Centre in the Lisbon area. Content analysis was supported by maturity levels of a patient safety culture, five maturity levels ranging from 1 (worst culture) to 5 (best culture).

**Results**

By adjusting the responses within a maturity level for the patient safety culture of the 2 functional units, it was observed that the culture oscillated between 1.8 values in the USF model A, close to a reactive culture, in which the organization only cares about safety when problems occur, and 4 values for USF B, close to a proactive culture, with patient safety measures, even without adverse events, close to the ideal, with an informed and worried team.

**Conclusions**

The functional units have the same typology, belong to the same Health Centre, but align the patient's safety culture with its greater and lesser complexity, respectively, model B and A. In other words, patient safety is not observed from the same perspective, although it operates in the same geographical area. There is a need for more and a better evaluation, information and training so that the safety culture develops.

**Keywords**

Functional units, Primary Health Care, Patient Safety Culture, Education and Training in Patient Safety.

### P91 Online opinion leaders and weight loss: a literature review based model

#### Inga Saboia^1^, Ana M Almeida^2^

##### ^1^Universidade Federal do Ceará, 60020-181 Fortaleza, Ceará, Brasil; ^2^Universidade de Aveiro, 3810-193 Aveiro, Portugal

###### **Correspondence:** Inga Saboia (guiguisaboia@gmail.com)

**Background**

Online opinion leaders shape the trends of the current Web 2.0 [1] and eHealth context [2]. In 2013, in the USA, 72% of the netizens sought out others with the same health problem [3]. This illustrates a scenario of a network among users that mutually influence health behaviours and health decisions, creating a new setting of an increasing digital literacy in health [4], in which the patient becomes an agent of their treatment. One of the most researched topics is weight control (3). The subject of this study is related to public health, specifically obesity, the epidemic of the 21st century [5].

**Objective**

This study pretends to build a model of analysis, based on a literature review. This model is expected to support a deeper understanding of the role of the opinion leaders on social networks, particularly the ones which sphere of influence acts on weight loss.

**Methods**

A literature review was conducted through a systematic mapping [6]. The handled databases were: Web of Science, Scopus, PubMed, and Google Scholar. The keywords were: Opinion leader OR Digital Influencer OR Powerful Patient OR Community AND Nutrition OR Obesity OR Weight AND Behaviour change, from 2012 to 2017.

**Results**

Opinion leadership relates to the degree in which an individual can influence others, according to their characteristics and practices, building interpersonal ties [7]. The opinion leaders types in the current Web 2.0 context are: professionals and non-professionals, being these last ones mainly patient opinion leaders (POLs) [8] or digital influencers [9]. POLs are patients who share content as support for others [8]. Besides these agents, we see the rise of health professionals who connect directly with their audience [9]. These have conquered the media by promoting the “right way to feed” and legitimating themselves through a scientific discourse [10]. Considering the digital influencers context, a dichotomy arises: they can be taken as a threat to public health, or as partners fostering the communication between doctors and patients [9]. Furthermore, social networks have also an important role in this scenario as they connect people with a common purpose (weight loss) [10-15].

**Conclusions**

This study identified two types of opinion leaders: health professionals, and nonprofessional ones. Both have different behaviours in social networks, but both have an important role in influencing the experience of their followers in weight loss.

**Keywords**

Online opinion leaders, Online social network, Digital influencers, Patient opinion leader, Digital literacy in health, Nutrition, Public health, Obesity, eHealth, Web 2.0.

### P92 Pharmacotherapeutic follow-up in institutionalized elderly

#### Ana Grou^1^, Carmen Monteiro^2^, Jorge Balteiro^1^

##### ^1^Escola Superior de Tecnologia da Saúde de Coimbra, Instituto Politécnico de Coimbra, 3046-854 Coimbra, Portugal; ^2^Farmácia Luciano e Matos, 3000-142 Coimbra, Portugal

###### **Correspondence:** Jorge Balteiro (balteiro@estescoimbra.pt)

**Background**

The pharmacotherapy follow-up is one of the best methods to diminish health problems and medicine therapy-associated morbidity. This procedure aims at improve the results obtained at the clinical level and optimize therapeutic plans.

**Objective**

This work aimed to perform the pharmacotherapy follow-up of elderly residents in long-stay institutions, identify their prevalent pathologies and trends in medicines consumption, as well as to identify and solve negative results of medication (NOM).

**Methods**

Pharmacotherapy follow-up procedures were applied to 38 elderly Residents in a Long-Stay Institution. The population analysed referred to suffer from a total of 212 pathologies. The majority of these concerned the circulatory system (n=45) and mental and behavioural disturbs (n=38). Daily, 273 medicines are consumed by this population and most of them target the nervous and cardiovascular systems (n= 93 and n=74, respectively).

**Results**

During the pharmacotherapy follow-up interviews 88 NOM were identified. Upon pharmacist intervention, 52 of the identified NOM were solved or placed under control. During this pharmacotherapy follow-up procedure 131 interventions were performed.

**Conclusions**

The establishment of pharmacotherapy follow-up is greatly advantageous for patients, particularly for elderly ones. This procedure optimized the results of the therapeutic plan, decreased the impact and solved NOM.

**Keywords**

Pharmacotherapy Follow-upk, Elderly Residents in Long-Stay Institutions, Negative Outcomes Associated with Medication Pharmacist Intervention.

### P93 The person with ostomy of intestinal elimination: social representation of nurses

#### Joana Pinho, Tânia Jesus, Liliana Mota

##### Escola Superior de Enfermagem da Cruz Vermelha Portuguesa, 3720-126 Oliveira de Azeméis, Portugal

###### **Correspondence:** Liliana Mota (saxoenfermeira@gmail.com)

**Background**

During the surgical formation of an intestinal elimination ostomy, the person is challenged to develop a set of self-care skills to guarantee quality of life throughout the health/illness transition process, and the nurse should act as a facilitator in this process [1]. Nurses have an important role to prepare these patients to return back home. This study intends to extract the essence from the nurses' point of view, about people with ostomy of intestinal elimination.

**Objective**

To describe the social representation of nurses about the person with intestinal elimination ostomy.

**Methods**

We conducted a qualitative, descriptive-exploratory study. Data was collected with an online form. The online form had five questions focused in the aim of the study, and each participant answered each question with five words according to their perception. The sample was of convenience, non-probabilistic constituted by 64 nurses which answered to an online form. Data were collected during the month of November (2017) sending emails to all contacts of nurses in the data bases of the nursing school. Anonymity was preserved. Data analysis was computed in IRAMUTEQ. We performed a classic lexicographical analysis.

**Results**

Participants had on average 33.08 (± 8.83) years old (between 22 and 58 years). The majority (76.6%) belonged to the feminine gender and 84.4% of participants were graduated. When the nurses think in ostomy of intestinal elimination, they think in the characteristics of the stoma. The smell has an important role in this category. Focused in person with intestinal ostomy, nurses are centred in self-care. The nurses consider support (emotional and familiar) and the teachings, the most important necessities of the person with an intestinal ostomy. When they think in the care of these persons they are focused in capacity and knowledge. The preparation to return back home is centred in the acceptance of the disease and on the relationship between nurses and patient.

**Conclusions**

The social representation of nurses about the person with intestinal elimination ostomy is focused in emancipatory patterns of nursing. The person is the centre of care and the care plan is focused in helping the person to live with quality with this new condition. These results are an important contribute to enhance the practices and to demonstrate the relevance of nursing health/illness transitions of the person with intestinal elimination ostomy.

**References**

1. Mota M, Gomes G, Petuco V, Heck R, Barros E. Facilitadores do processo de transição para o autocuidado da pessoa com estoma: subsídios para enfermagem. Revista da Escola de Enfermagem USP. 2015. 49(1):82-88.

**Keywords**

Ostomy, Intestinal elimination, Social representation, Nursing.

### P94 A synthesis of Portuguese studies regarding infertile patients

#### Joana Romeiro, Sílvia Caldeira

##### Institute of Health Sciences, Universidade Católica Portuguesa, 1649-023 Lisbon, Portugal

###### **Correspondence:** Joana Romeiro (joana.m.romeiro@gmail.com)

**Background**

Infertility is clinically defined as the inability to conceive and to achieve successful clinical pregnancy after 12 months of regular and unprotected sexual intercourse [1]. In 2010, 48.5 million couples worldwide were reported to have fertility problems [2], affecting both genders in 40% of the cases [3]. Due to this high and broad prevalence, infertility is acknowledged as a public health issue with prioritized intervention [4]. The prevalence in Portuguese population was first known in 2009, when a study estimated that about 260-290 thousand individuals were infertile and approximately 9% to 10% of couples displayed some type of reproductive confinement [5]. These results triggered scientific interest in the study of Portuguese infertile patients and a synthesis of the published Portuguese studies regarding infertility seems important in understanding and caring for these patients.

**Objective**

To review scientific health empirical research in the study of Portuguese infertile patients.

**Methods**

Literature review based on search conducted in December 2017. A total of 12 scientific data bases were searched: CINAHL with full text, MEDLINE with full text, MedicLatina, Academic Search Complete, Pubmed, Web of Science, LILACS, SciELO, RCAAP, and across ESENFC; Nursing School of Lisbon and Nursing School of Porto databases. No date limit has been applied. Studies considered eligible for inclusion were primary studies in Portuguese samples of male or female individuals and/or in couples having reproductive impairment, available in a full-text format, published on peer-reviewed journals in English, Spanish or Portuguese language.

**Results**

A total of 2,052 results have been identified and 101 papers were included. Empirical research regarding infertile couples started to be published in 1995. Until current date, 2013 was the year with the highest publication score (13.8%) with psychological aspects of the infertile experience being the most explored (57.4%) in comparison with other health aspects, like for instance related to nursing (2.9%), and psychiatry (0.9%). Primary studies were also published in international journals (53.4%) as original papers (62.3%), and in a thesis format (37.6%).

**Conclusions**

Although the developments in health research regarding infertile couples, a significant gap in the knowledge remains, particularly concerning other health disciplines (despite psychology). This seems to be a global tendency in healthcare, and further investigation is needed to fully acknowledge this phenomenon and consequently allow the provision of an effective patient-centred care to these patients.

**References**

1. Zegers-Hochschild F, Adamson G D, Mouzon J de, Ishihara O, Mansour R, Nygren K van der, Poel S. The International Committee for Monitoring Assisted Reproductive Technology (ICMART) and the World Health Organization (WHO) Revised Glossary on ART Terminology, 2009. Human Reproduction, 24(11), 2683–2687.

2. Mascarenhas M N, Flaxman S R, Boerma T, Vanderpoel S, Stevens G A. National, Regional, and Global Trends in Infertility Prevalence Since 1990: A Systematic Analysis of 277 Health Surveys. PLOS Medicine. 2012, 9(12), 1–12.

3. NICE. Fertility, Assessment and treatment for people with fertility problems. 2013. National Institute for Health and Care Excellence.

4. Centers for Disease Control and Prevention. National Public Health Action Plan for the Detection, Prevention, and Management of Infertility. U.S. Department of Health and Human Services. 2014. Retrieved from http://www.cdc.gov/reproductivehealth/infertility/pdf/drh_nap_final_508.pdf

5. Carvalho, J. L. S., & Santos, A. Estudo Afrodite, Caracterização da Infertilidade em Portugal (p. 74). Porto: Faculdade de Medicina da Universidade do Porto, Sociedade Portuguesa de Medicina da Reprodução, KeyPoint. 2009.

**Keywords**

Infertility, Health, Evidence-based, Review.

### P95 Knowledge and consumption of vitamins and food supplements in sportspeople and physical exercise in Coimbra

#### Adriana Ferreira, Clara Rocha, Jorge Balteiro

##### Coimbra Health School, Polytechnic Institute of Coimbra, 3046-854 Coimbra, Portugal

###### **Correspondence:** Jorge Balteiro (balteiro@estescoimbra.pt)

**Background**

The demand for healthy lifestyles, the concern with health and well-being, and the relentless pursuit of the trend of the “ideal body” has been increasing in recent years, as well as the prevalence of supplement use, as a compensation for an unbalanced diet and search for physical/psychic intensity.

**Methods**

In order to evaluate the consumption and knowledge about vitamin and dietary supplements in Coimbra, a sample of 333 individuals practicing sports was studied.

**Results**

The study lasted for nine months. The collection of information was carried out through a questionnaire. The study found that 201 (60.4%) subjects have consumed supplementation, with a prevalence of higher consumption in males (73.3%). Supplement use was higher between 33 and 40 years old individuals. The most consumed type of vitamin supplement was multivitamins with minerals (44.3%) and the food supplement was protein (69%). The most cited reason for the consumption of supplements was “physical and/or intellectual fatigue” (50.5%). The daily frequency of supplementation was high (33.7%), with the highest expenditure on consumption of supplements varying from 10 to 20€, monthly. The place of purchase and the source from which subjects obtained knowledge about supplements was the Internet. As for knowledge on the subject, it was noted that it has been classified as “insufficient” (45.8%) by respondents.

**Conclusions**

In Portugal, the prevalence of supplementation consumption is still unknown, so it becomes necessary to raise awareness among the population, about potential risks associated with improper supplementation, special diets and unbalanced exercise.

**Keywords**

Vitamins supplements, Food supplements, Consumption, Knowledge.

### P96 Stability of paediatric oral diazepam suspensions

#### Patrícia Marinho^1^, Patrícia Correia^2^

##### ^1^Escola Superior de Saúde do Porto, Instituto Politécnico do Porto, 4200-072 Porto, Portugal; ^2^Centro de Investigação em Saúde e Ambiente, Escola Superior de Saúde, Instituto Politécnico do Porto, 4200-072 Porto, Portugal

###### **Correspondence:** Patrícia Marinho (patriciampmarinho@gmail.com)

**Background**

Currently, hospital pharmacies prepare formulations that aim to adjust the medication to the needs of each patient when the pharmaceutical industry is not able to respond to those needs [1]. One of the formulations produced in the hospital pharmacy is the diazepam suspension 0.4 mg/ml for paediatric use, obtained from diazepam tablets. However, the use of tablets or powders in oral liquid formulations may alter the stability of the active ingredients. Therefore, these formulations should be submitted to stability studies [2]. Nevertheless, the information on the stability of manipulated oral suspensions is scarce [3], so this study is relevant.

**Objective**

The main goal of this study is to validate a method of diazepam quantification in suspensions. Additionally, we aim to evaluate the stability of diazepam in suspensions during 30 days, after the suspension preparation, establishing an expiration date.

**Methods**

The quantification method of diazepam in oral suspensions arose from the adaptation of the method described in Portuguese Pharmacopoeia [4], for the same active ingredient in tablets. After the method’s validation, the stability of diazepam was evaluated weekly, during 30 days, and the first analysis was done immediately after the preparation of the suspension. During the study period, suspensions were stored under suitable cold conditions (4°C).

**Results**

With an accuracy, evaluated by the mean recovery of 80%, and a precision, evaluated by the variation coefficient, varying between 6.1 and 11.5%, the method proved to be practicable. Two suspension’s samples were prepared with a similar diazepam concentration (0.43 mg/ml). The stability study of those suspensions showed that diazepam concentration decayed linearly, and that diazepam suspensions lose about 70% of their active principle within 30 days. Moreover, given the limits indicated by the Portuguese Pharmacopoeia [4] for diazepam tablets, it was verified that these suspensions only comply with these limits after 7 days, and that within the established period of validity these limits are no longer met.

**Conclusions**

Despite all limitations, the adapted method proved to be practicable and the results that followed have pointed to the possible instability of diazepam, when included in this oral suspension formulation. Given the dosage limits set for diazepam tablets [4] and knowing in advance that the validity period usually attributed to the suspension is 15 days, the results point to a new shelf-life of approximately 7 days. However, for a more consistent period of validity to be established, a more detailed stability study is required.

**References**

1. Patel VP, Desai TR, Chavda BG, Katira RM. Extemporaneous dosage form for oral liquids. Pharmacophore, 2011, 2(2), 86-103.

2. Schlatter J, Bourguignon E, Majoul E, Kabiche S, Balde I B, Cisternino S, Fontan J E. Stability study of oral paediatric idebenone suspensions. Pharmaceutical Development and Technology, 2016, 22(2), 296-299

3. Ensom M H H, Kendrick J, Rudolph S, Decarie D. Stability of Propranolol in Extemporaneously Compounded Suspensions. The Canadian Journal of Hospital Pharmacy, 2013, 66(2), 118–124.

4. INFARMED. Farmacopeia Portuguesa. 8ª edição. 2008, 1925-1926; 2224-2225.

**Keywords**

Diazepam suspensions, Chemical stability, Validation tests, Dosing method, Expiration date.

### P97 First-time grandparents and transition to grandparenthood: integrative review of the literature

#### Sónia Coelho^1^, Rogério Rodrigues^2^, Isabel Mendes^2^

##### ^1^Health Sciences Research Unit: Nursing, Nursing School of Coimbra, 3046- 851 Coimbra, Portugal; ^2^Nursing School of Coimbra, 3046-051 Coimbra, Portugal

###### **Correspondence:** Rogério Rodrigues (rogerio@esenfc.pt)

**Background**

Nowadays families become smaller but at the present time a family involves several generations (even if they do not live together). The family members’ roles change and the role of the grandparents in the transition of first-time parents to grandparenthood needs to be understood.

**Objective**

To systematize an integrative review of the literature related to the transition to grandparenthood in contemporary Western societies.

**Methods**

We conducted an integrative review of the literature, in electronic databases (EBSCO®, b-On® and Web of Knowledge®) in order to answer the question: “*How is experienced the transition to grand-parenting?*” The search was limited to articles published between 2006-2016 years, with the descriptors “grandparents” and “transition" in English, French, Portuguese or Spanish.

**Results**

After analysing the abstracts of 179 articles, excluding repetitions, and those who did not respond to the original question, we obtained 13 articles to include in the integrative review. The level of the methodological approach was level 4. Only descriptive and qualitative studies (non-experimental) were included. The results of the literature review on the topic were grouped into five themes: grand-parenting and gender; become a grandfather/grandmother; parenting and the transition process; role and health; parenting and intergenerational relations.

**Conclusions**

It was found that the transition to Grandparenthood is studied in risk situations, and more studied in women than in men. Grandparenthood can be seen as a transition or as an adaptive process; as the search for the meaning of life; opportunity for personal growth; a normative event that has emotions and positive and negative cognitions. The process of becoming a grandparent can be considered an event of great social impact. Grandparents see their grandchildren as their extension in time and this gives them a more positive view of aging. The perception that grandparents have of themselves may be important in promoting a positive and healthy aging.

**Keywords**

Grandparenthood, Grandparents, Transition.

### P98 Intestinal microbiota - impact on host health

#### Nastasia Fernandes, Alice Nunes, Maria José Alves

##### Polytechnic Institute of Bragança, 5300-253 Bragança, Portugal

###### **Correspondence:** Nastasia Fernandes (nastasia-ar1000@hotmail.com)

**Background**

At present it is known that in addition to establish and maintain a normal intestinal health, the intestinal microbiota can exacerbate a multitude of diseases, ranging from colorectal cancer to autoimmune and allergic diseases [1]. The interest in studying the human microbiome, its diversity and human-microorganism interactions has been developing in the last years, as such it has been made available immense information in this area.

**Objective**

Bibliographic review of the intestinal microbiota: constitution, what affects it, and its influence in the triggering of some pathologies.

**Methods**

A comprehensive search was performed on the PubMed search, being obtained from this review 112 articles from which 67 were used.

**Results**

The intestinal microbiota is considered a “superorganism” and is extremely complex. It is composed of a great diversity of microorganisms, which varies among individuals; however, it is essentially dominated by two phyla, the Bacteroidetes and the Firmicutes. It is dynamic and can be affected by several factors such as diet [2], breastfeeding [3], use of antibiotics [4,5] and type of delivery [6,7]. When an imbalance of the microbiota occurs, known as dysbiosis [1,8], the host is affected, being related to pathologies such as allergies, obesity and Crohn's disease. Some studies [9,10-14] have demonstrated that the microbiota participates in the maturation of the immune system and as such is predominant in the response to infectious processes. On the other hand, the intestinal microbiota seems to play a fundamental role in the prevention of allergies [15-26]. Inappropriate colonization after birth and excessive hygiene during childhood, may promote greater allergic reactions. Rats without bacteria have been shown to present more severe allergic reactions [22-26]. Regarding obesity, several authors [27-30] have demonstrated that changes in microbiota are strongly related to the establishment of obesity. It has been demonstrated that the type of microbiota influences obesity; rats with higher amounts of Firmicutes compared to the amount of Bacteroidetes, present greater capacity to promote fat deposition. On the other hand, a switch to a less caloric diet produced a change in the microbiota that led to a decrease in Firmicutes and an increase in Bacteroidetes. These results are surprising and suggest that future obesity control may originate from the type of intestinal microbiota.

**Conclusions**

Intestinal microbiota is of great relevance because it protects against external factors and the development of certain pathologies. It is therefore important to keep the population informed so that a microbiota considered “normal” can be maintained from childhood to adulthood.

**References**

1. Parnell JA, Reimer RA. Prebiotic fiber modulation of the gut microbiota improves risk factors for obesity and the metabolic syndrome. Gut Microbes. 2012;3(1):29-34.

2. David LA, Maurice CF, Carmody RN, Gootenberg DB, Button JE, Wolfe BE. Diet rapidly and reproducibly alters the human gut microbiome. Nature. 2014;505(7484):559-563.

3. Cox LM, Blaser M. J. Antibiotics in early life and obesity. Nat Rev Endocrinol. 2015;11(3):182-190.

4. Clemente JC, Ursell LK, Parfrey LW, Knight R. The impact of the gut microbiota on human health: an integrative view. Cell. 2012;148(6):1258-1270.

5. Jernberg C, Löfmark S, Edlund C. Long-term impacts of antibiotics exposure on the human intestinal microbiota. Microbiology. 2010;156(Pt11):3216-3223.

6. Adlerberth I, Strachan DP, Matricardi PM, Ahrne S, Orfei L, Aberg N, et al. Gut microbiota and development of atopic eczema in 3 European birth cohorts. J Allergy and Clin Immunol. 2007;120(2):343–50.

7. Gronlund MM, Lehtonen OP, Eerola E, Kero P. Fecal microflora in healthy infants born by different methods of delivery: permanent changes in intestinal flora after cesarean delivery. J. Pediatr Gastroenterol Nutr. 1999;28(1):19–25.

8. Blumberg R, Powrie F. Microbiota, Disease, and Back to Health: A Metastable Journey. Sci Transl Med. 2012;4(137):137rv7.

9. Swidsinski A, Loening-Baucke V, Lochs H, Hale LP. Spatial organization of bacterial flora in normal and inflamed intestine: a fluorescence in situhybridization study in mice. Word J Gastroenterol. 2005;11(8):1131- 1140.

10. Hartstra AV, Bouter KE, Backhed F, Nieuwdorp M. Insights into the role of the microbiome in obesity and type 2 diabetes. Diabetes Care. 2015;38(1):159-65.

11. Chow J, Lee SM, Shen Y, Khosravi A, Mazmanian SK. Host-bacterial symbiosis in health and disease. Adv Immunol. 2010;107:243–274.

12. O'Hara AM, Shanahan F. The gut flora as a forgotten organ. EMBO Rep. 2006;7:688–693.

13. Purchiaroni F, Tortora A, Gabrielli M, Bertucci F, et al. The role of intestinal microbiota and the immune system. Eur Rev Med Pharmacol Sci. 2013;17(3):323-33.

14. Round JL, Mazmanian SK. The gut microbiota shapes intestinal immune responses during health and disease. Nat Rev Immunol. 2009;9:313–323.

15. Bach JF. The effect of infections on susceptibility to autoimmune and allergic diseases. N Engl J Med. 2002;347:911-20.

16. Pelucchi C, Galeone C, Bach JF, La Vecchia C, Chatenoud L. Pet exposure and risk of atopic dermatitis at the pediatric age: a metaanalysis of birth cohort studies. J. Allergy Clin Immunol. 2013;132(3):616622.e7.

17. Stiemsma LT, Turvey SE. Asthma and the microbiome: defining the critical window in early life. Allergy Asthma Cli Immun. 2017;13:3.

18. Chieppa M, Rescigno M, Huang AY, Germain RN. Dynamic imaging of dendritic cell extension into the small bowel lumen in response to epithelial cell TLR engagement. J Exp Med. 2006;203(13):2841–52.

19. Ignacio A, Morales CI, Camara NO, Almeida RR. Innate sensing of the gut microbiota: modulation of inflammatory and autoimmune diseases. Front Immunol. 2016;7:54.

20. Round JL, Lee SM, Li J, Tran G, Jabri B, Chatila TA, et al. The Toll-like receptor pathway establishes commensal gut colonization. Science. 2011;332(6032):974-977.

21. Hessle C, Hanson LA, Wold AE. Lactobacilli from human gastrointestinal mucosa are strong stimulators of IL-12 production. Clinical and Experimental Immunology. 1999;116(2):276-282.

22. Herbst T, Sichelstiel A, Schar C, Yadava K, Burki K, Cahenzli J, et al. Dysregulation of allergic airway inflammation in the absence of microbial colonization. Am J Respir Crit Care Med. 2011;184(2):198–205.

23. rompette A, Gollwitzer ES, Yadava K, Sichelstiel AK, Sprenger N, NgomBru C, et al. Gut microbiota metabolism of dietary fiber influences allergic airway disease and hematopoiesis. Nat Med. 2014;20(2):159– 66.

24. Schuijs MJ, Willart MA, Vergote K, Gras D, Deswarte K, Ege MJ, et al. Farm dust and endotoxin protect against allergy through A20 induction in lung epithelial cells. Science. 2015;349(6252):1106–10.

25. Kumar H, Lund R, Laiho A, Lundelin K, Ley RE, Isolauri E, et al. Gut microbiota as an epigenetic regulator: pilot study based on wholegenome methylation analysis. MBio. 2014;5(6):e02113–4.

26. Thorburn AN, McKenzie CI, Shen S, Stanley D, Macia L, Mason LJ, et al. Evidence that asthma is a developmental origin disease influenced by maternal diet and bacterial metabolites. Nat Commun. 2015; 6:7320.

27. Turnbaugh PJ, Ley RE, Mahowald MA, Magrini V, Mardis ER, Gordon JI. An obesity-associated gut microbiome with increased capacity for energy harvest. Nature. 2006;444(7122):1027–1031.

28. Bäckhed F, Ding H, Wang T, Hooper LV, Koh GY, Nagy A, et al. The gut microbiota as an environmental factor that regulates fat storage. Proc Natl Acad Sci U S A. 2004; 101:15718–23.

29. Schwiertz A, Taras D, Schäfer K, Beijer S, Boss NA, Donus C, Hardt PD. Microbiota and SCFA in lean and overweight healthy subjects. Obesity (SilverSpring) 2010;18:190–195.

30. Bäckhed F, Manchester JK, Semenkovich CF, Gordon JI. Mechanisms underlying the resistance to diet- induced obesity in germ-free mice. Proc Natl Acad Sci U S A. 2007; 104:979–84.

**Keywords**

Intestinal microbiota, microbioma, immune system, dysbiosis, obesity, allergies, Crohn's disease.

### P99 Effects of aging on neuromuscular activity during the performance of a ballistic motor skill

#### António M Vencesbrito^1,2,3,4^, Mário A Rodrigues-Ferreira^1,2,3^

##### ^1^Escola Superior de Desporto de Rio Maior, Instituto Politécnico de Santarém, 2040-413 Rio Maior, Portugal; ^2^Unidade de Investigação do Instituto Politécnico de Santarém, 2040-413 Rio Maior, Portugal; ^3^Centro de Investigação em Qualidade de Vida, 2040-413 Rio Maior, Portugal; ^4^International Martial Arts and Combat Sports Scientific Society, Rzeszów, Poland

###### **Correspondence:** António M Vencesbrito (abrito@esdrm.ipsantarem.pt)

**Background**

Human aging leads to a progressive decline of biological functions that affects the neuro-muscular systems. Sport practice is associated with health maintenance and a better quality of life in older people.

**Objective**

The aim of this study was to investigate the effects of aging on the neuromuscular reaction time and electromechanical delay during the performance of the karate frontal kick (Mae-Geri).

**Methods**

Participated in this study 9 elite karate athletes (age, 21.0 ± 2.47 years; height, 175 ± 6.53 cm; weight, 72.0 ± 9.25 kg) and 9 veteran karate practitioners (age, 54.0 ± 3.87 years; height, 176 ± 4.72 cm; weight, 76.0 ± 9.17 kg). Surface electromyography was recorded from rectus femoris (RF) and vastus lateralis (VL) portions of the quadriceps femoris, long head of the biceps femoris (BF), tibialis anterior (TA) and lateralis gastrocnemius (GA). Kinematic analysis was performed with the Ariel Performance Analysis System (APAS, Ariel Dynamics-2003). The neuromuscular reaction time was defined as the time interval between the auditory stimulus and the onset of electrical activation of a muscle, while the electromechanical delay was the time interval between the onset of the electric activity of a muscle and the beginning of joint movement. Student t-test (two-tailed) was used to analyse the differences between groups, with a significance level of p < 0.05 (SPSS 17.0).

**Results**

It was observed a tendency to a longer neuromuscular reaction time of the TA in veteran karate practitioners than among elite karate athletes (136.00 ± 58.80 vs 122.00 ± 45.94 ms, p = 0.566), although a significantly shorter neuromuscular reaction time was found in the RF in veteran karate practitioners (137.00 ± 27.93 vs 184 ± 51.55 ms, p = 0.030). Veteran karate practitioners presented a significantly longer RF electromechanical delay than elite karate athletes (127.00 ± 59.11 vs 39.00 ± 47.68 ms, p = 0.003).

**Conclusions**

The results of the study showed that with the aging process there is an increase in the electromechanical delay, although no negative impact on the neuromuscular reaction time has been observed. Therefore, continuous sport practice in veteran karate practitioners seems to attenuate the effects of aging on neuromuscular systems.

**Keywords**

Neuromuscular reaction time, Electromechanical delay, Electromyography, Karate.

### P100 Nutritional status in institutionalized elderly: is it influenced by polymedication and length of stay?

#### Maria A Marques^1,2^, Ana Faria^3,4^, Marisa Cebola^2,5^

##### ^1^Santa Casa da Misericórdia de Alvaiázere, 3250-115 Alvaiázere, Portugal; ^2^Faculdade de Medicina, Universidade de Lisboa, 1649-028 Lisboa, Portugal; ^3^Centro Hospitalar e Universitário de Coimbra, 3000-075 Coimbra, Portugal; ^4^Escola Superior de Tecnologia da Saúde de Coimbra, Instituto Politécnico de Coimbra, 3046-854 Coimbra, Portugal; ^5^Escola Superior Tecnologia da Saúde de Lisboa, Instituto Politécnico de Lisboa, 1990-096 Lisboa, Portugal

###### **Correspondence:** Maria A Marques (mariaanfm@gmail.com)

**Background**

Aging is frequently associated with conditions, like malnutrition, that may affect the elderly health status and quality of living. Malnutrition has a high prevalence in institutionalized elderly [1]. Polymedication is also frequent in older individuals, impairing appetite and possibly contributing to the development of malnutrition [2].

**Objective**

The aim of this study was to identify nutritional risk in institutionalized elderly and establish a relationship between length of stay in the institution, pharmacotherapy and presence of malnutrition.

**Methods**

Day of admission, number of medications and sociodemographic data were collected from the patient’s medical file. Malnutrition and nutritional risk were assessed using the Mini Nutritional Assessment (Short Form) (MNA-SF).

**Results**

Seventy-eight individuals, mainly female (59.0%), with a mean age of 81.7 years (SD= 10.2) were evaluated. Mean length of stay was 6.4 years (SD= 7.8). MNA-SF classified 32 individuals (41.0%) at risk of malnutrition and 20 (25.6%) as malnourished. According to the elderly Body Mass Index (BMI) classification, 28 were at risk of malnutrition and 23 were malnourished (35.9% and 29.7%, respectively), showing a positive correlation with MNA-SF results (p < 0.05). The female gender presented an overall higher risk of malnutrition (p < 0.05). Those who were more dependent to feed themselves were at risk of malnutrition or malnourished (p < 0.05). Fifty-seven of the subjects (73.0%) were under polymedication, with a mean number of daily medications of 7.5 (SD= 3.4). A higher number of regular medications is correlated with higher BMI (p < 0.05). No association was found between length of stay or number of drugs taken and malnutrition.

**Conclusions**

In this population, a high prevalence of risk of malnutrition was identified, particularly in the female gender. Although previously described, a correlation between polypharmacy and malnutrition was not found. A closer look to type of medication might be necessary. In this sample, a very long length of stay was also found. Nutritional intervention in this population should be prompt since admission and regularly provided, preventing the development of malnutrition and comorbidities.

**References**

1. Cereda E. Mini nutritional assessment. Curr Opin Clin Nutr Metab Care. 2012;15(1):29–41.

2. Jyrkkä J, Mursu J, Enlund H, Lönnroos E. Polypharmacy and nutritional status in elderly people. Curr Opin Clin Nutr Metab Care. Janeiro de 2012;15(1):1–6.

**Keywords**

Malnutrition, Elderly, Polypharmacy .

### P101 Food safety and public health in canteens of public and private educational establishments and in private institutions of social solidarity

#### Cristina Santos^1^, Esmeralda Santos^2^

##### ^1^Department of Environmental Health, Coimbra Health School, 3046-854 Coimbra, Portugal; ^2^Agrupamentos de Centros de Saúde do Baixo Mondego, 3150-195 Condeixa, Portugal

###### **Correspondence:** Cristina Santos (cristinasofiasantos@gmail.com)

**Background**

To promote and guarantee hygiene and food safety is nowadays a requirement in any service involving the provision of food, as a means of ensuring the promotion of a high level of protection and consumer confidence. These changes boosted the growth of the catering industry. However, they also require the evolution of techniques, so as to enable catering and catering companies to offer food of quality [1-3]. Most cases of food poisoning are due to poor hygiene habits. Structural failures and ignorance or neglection of good hygiene and food safety practices may also lead to food contamination [4,5].

**Objective**

The sample consisted of canteens of public and private educational establishments and of public and private social solidarity institutions, totalling 26 canteens and 127 professionals. Data collection was performed using a diagnostic sheet of the structural conditions and operation of the facilities.

**Results**

Measurements of polar compounds in canteens indicated good quality, except for one of the measurements that indicated a less satisfactory quality. In the evaluation of food temperature, it was found that there are some foods that are served in the “danger zone” (< 65°C). School cafeterias (without food confectionery) had, in majority, deficient conditions of installation because they were rooms of activities where the meals were served. For this reason, there were no water baths or meal service facilities.

**Conclusions**

With this work it was concluded that there are deficiencies regarding the structural and operating conditions of canteens/refectories, which could be filled by the construction/enlargement of spaces. Regarding the evaluation of the quality of the oils and temperature of the meals, there were flaws, with possible repercussions on the quality of the meals served. It is also important to develop skills for the elaboration of menus suited to the different age groups and the confection of healthier diets. Emphasis can be placed on the training of manipulators in order to raise awareness of the repercussions of their role and responsibilities in preventing contamination. Ensuring and promoting food safety is nowadays a requirement of any institution, where food is produced or distributed, as a means of ensuring the promotion of high levels of confidence and safeguarding of the consumer’s health.

**References**

1. Baptista P, Antunes C. Higiene e Segurança Alimentar na Restauração. Vol. I. Guimarães: Forvisão – Consultoria em Formação Integrada; 2005.

2. Baptista P, Antunes C. Higiene e Segurança Alimentar na Restauração. Vol. II. Guimarães: Forvisão – Consultoria em Formação Integrada; 2005.

3. Afifi HS, Abushelaibi A. Assessment of personal hygiene knowledge, and practices in Al Ain, United Arab Emirates. Food Control. 2012;25:249-253.

4. Associação da Restauração e Similares de Portugal. Higiene e Segurança Alimentar – Código de boas práticas para a restauração pública; 2006.

5. Lima VT. Educação nutricional na escola. In: Seminário de Alimentação ESCOLAR, 3, 1999, ITAL. Resumos. Campinas, São Paulo. p.61.

**Keywords**

Public Health; Food Safety; Canteens; Promoting Food Safety.

### P102 Evaluation of the correlation between height and health of the spine in the student population in the age group of 16 - 19 years old - evaluation with spinal mouse^®^

#### Alexandra Monteiro, Nelson Azevedo, João Silva, Liliana Rodrigues, Gilvan Pacheco

##### Instituto Superior de Saúde do Alto Ave, 4720-155 Amares, Portugal

###### **Correspondence:** Nelson Azevedo (nelsonjaze@gmail.com)

**Background**

The increasing number of postural deviations observed in the student population leads to changes in the spine' normal curvature, which translates into a greater vulnerability to mechanical stress and traumatic injuries. Although the causes of these postural deviations are diverse and difficult to analyse, the present study decided to investigate whether the height of the students may be one of the factors influencing the appearance of postural alterations detected by the non-invasive evaluation method of the Spinal Mouse®.

**Objective**

Analyse differences in the incidence of postural changes in a sample of students aged 16-19 years with different heights (cm).

**Methods**

Eighty-five (85) students aged 16-19 years from Amares High school (Braga) were selected and submitted to a non-invasive postural evaluation by the Spinal Mouse® device, which showed the presence of hypomobility, normal mobility and hypermobility at the sagittal plane in three zones of the vertebral spine (sacral, thoracic and lumbar), as well as an overall tilt in three distinct positions: orthostatic, flexion and extension. Data analysis was performed using the statistical program IBM® SPSS® (Statistical Package for the Social Sciences), version 25. In the statistical tests performed, it was considered as levels of significance, the values of 0.05 (significant) and of 0.01 (extremely significant).

**Results**

The results indicate statistically significant differences (Kruskal-Wallis test, H) in the incidence of postural deviations (sagittal plane) in the sacral zone in flexion position (H = 6.629, p-value = 0.036), in general slope in flexion position (H = 6.738, p-value = 0.046), in the thoracic zone in the extension position (H = 11.390, p-value = 0.003) and in the lumbar zone in the extension position (H = 6.738, p-value= 0.034) for the different height groups considered (“<159 cm”, “159 - 177 cm” and “> 177 cm”).

**Conclusions**

Through the results we can conclude that there is a significant relationship between postural changes and students' height. In this way, it is fundamental to equate the ergonomic model of the school support material in order to adjust to different postures.

**Keywords**

Spine, Postural changes, Adolescent height, Ergonomics, Spinal Mouse.

### P103 The contribution of a Portuguese innovation to prevent complication in venous catheterization

#### Inês Cardoso^1^, Anabela Salgueiro-Oliveira^1^, Arménio Cruz^1^, José M Martins^1^, Liliana Sousa^1^, Sara Cortez^2^, Filipa Carneiro^3^, Pedro Parreira^1^

##### ^1^Coimbra Nursing School, 3046-851 Coimbra, Portugal; ^2^Muroplás - Indústria de Plásticos, 4745-334 Muro, Trofa, Portugal; ^3^Innovation in Polymer Engineering, University of Minho, 4800-058 Guimarães, Portugal

###### **Correspondence:** Inês Cardoso (inescardoso@esenfc.pt)

**Background**

Venous catheterization is one of the most frequent procedures in nursing clinical practice. Despite the procedure’s importance for healthcare quality, it has some risks and complications such infiltration, bloodstream infection and phlebitis. Healthcare associated infections are considered a worldwide problem, having a considerable impact on the patients’ and community’s health and economy [1,2]. According to the European Centre for Disease Prevention and Control, prevalence of this type of infections is 6.0% and 10.6%, in Europe and in Portugal, respectively. Bloodstream infections, related with venous catheters, have one of the lowest prevalence, but they may lead to serious consequences [2].

**Objective**

Explore the procedure of venous catheterization as a risk factor for infection. Explore the role of an innovation as a contribution to the implementation of a prevention measure, in the practice of flushing.

**Methods**

A literature review involved search in EBSCOhost databases, including articles up to 2017. Some terms used were “infection”, “venous catheter”, “catheterization”, catheter-related infection” “complication” “prevention” “management” “practices”, “flushing”. Articles regarding haemodialysis and urinary catheters were excluded. Guidelines of the societies regarding infusion nursing practices were also included.

**Results**

Catheterization is considered a risk factor because it creates a possibility for microorganisms to access to the bloodstream. The implementation of an insertion protocol is not enough to eliminate microorganisms and avoid the formation of a biofilm. Integrated in maintenance procedures, flushing contributes to reduce the risk of complications. Studies concluded that the use of an adequate technique of flushing may reduce the biofilm and the probability of infection [3]. Infusion Nurses Society [4] and the Royal College of Nursing [5] include it as a procedure to prevent complications of the infusion therapy. Despite the recommendations, the implementation of this practice is not consistent. To explain lack of adherence, one of the reasons pointed out is the complexity of the task [6]. These results support the development of a Medical Device (MD) (double chamber syringe) that may contribute to the adherence of the flushing practice.

**Conclusions**

It is important to create strategies to improve adherence to guidelines in clinical context, particularly regarding healthcare associated infections. The development of a MD that may simplify the accomplishment of a good practice, such as, flushing in venous catheter to prevent complication, is proposed in this project.

**Acknowledgements**

Work funded by the FEDER fund, through the Operational Programme for Competitiveness and Internationalisation (COMPETE 2020), project POCI-01-0247-FEDER-017604.

**References**

1. World Alliance for Patient Safety. The Global Patient Safety Challenge 2005-2006 “Clean Care is Safer Care”. Geneva, World Health Organization; 2005.

2. European Centre for Disease Prevention and Control. Point prevalence survey of healthcare- associated infections and antimicrobial use in European acute care hospitals. ECDC; 2013.

3. Ferroni A, Gaudin F, Guiffant G, Flaud P, Durussel JJ, Descamps P, et al. Pulsative flushing as a strategy to prevent bacterial colonization of vascular access devices. Medical Devices. 2014;7:379-383.

4. Royal College of Nursing. Standards for infusion therapy (4th ed.). London: Royal College of Nursing; 2016.

5. Infusion Nurses Society. Infusion therapy standards of practice. Journal of Infusion Nursing. 2016;39(1S):S1-160.

6. Keogh S, Shelverton C, Flynn J, Davies K, Marsh N, Rickard C. An observational study of nurses’ intravenous flush and medication practice in the clinical setting. 2017;3(1):3-10.

**Keywords**

Venous catheter, Infection, Prevention, Flushing.

### P104 Patient safety culture: evaluation of multiprofessional teams

#### Luciane PA Cabral^1^, Daniele Brasil^1^, Andressa P Ferreira^2^, Clóris RB Grden^1^, Caroline Gonçalves^1^, Guilherme Arcaro^2^

##### ^1^Departamento de Enfermagem e Saúde Pública, Universidade Estadual de Ponta Grossa, 4748 Ponta Grossa, Paraná, Brasil; ^2^Hospital Universitário Regional dos Campos Gerais, 84031-510 Ponta Grosa, Paraná, Brasil

###### **Correspondence:** Luciane PA Cabral (luciane.pacabral@gmail.com)

**Background**

Patient safety is an essential constituent of the quality of care, and assumes absolute relevance for managers, health professionals, family members and patients, in an attempt to provide a safe care.

**Objective**

The aim of the study was to evaluate the characteristics of the Patient Safety Culture among professionals of an Intensive Care Unit, through the application of the Hospital Survey on Patient Safety Culture (HSOPSC) instrument.

**Methods**

The study population consisted of 2% (n= 1) resident physicians; 2% (n= 1) nutritionists; 2% (n= 1) administrative assistants; 5% (n= 3) general service aiders; 7% (n= 4) social workers; 8% (n= 5) dentists; 17% (n= 10) nurses; 18% (n= 11) therapists and 40% (n= 24) nursing technicians. Of these 77% (n= 46) were females and 23% (n= 14) males. When questioned about whether their errors were recorded on their functional sheets or used against them in future opportunities, the data became alarming. When questioned about the conduct of their supervisor or boss, it was noted that a considerable number of respondents opted to abstain from comments, marking the option “*I do not agree or disagree*”. In the issue that addresses the communication in the service, the results were partially satisfactory. Regarding the frequency of reported events, there is a lack of notifications in the sector. When questioned about how patient safety is in the industry 3% (n= 2) considered it excellent, 63% (n= 38) very good and 33% (n= 20) regular. Regarding the general information of the interviewees, which were cited in the introduction of the results, in the open question, only 5% (n= 3) of respondents answered.

**Conclusions**

The study allowed the evaluation of patient safety characteristics from the perspective of the multi-professional team of an Intensive Care Unit, indicating that there are many aspects to improve in several dimensions on the patient's culture. However, there are areas with greater fragility, that need a closer look, such as the lack of notifications on the part of the team, issues on the supervisor/chief of the sector and mainly fear of punitive culture.

**Keywords**

Patient, Safety, Multi-professional.

### P105 Innovation in nursing in the creation of medical devices: a Portuguese case study

#### Pedro Parreira^1^, Inês Cardoso^1^, Liliana Sousa^1^, Arménio Cruz^1^, José Martins^1^, Sara Cortez^2^, Filipa Carneiro^3^, Luciene Braga^4^ Anabela Sousa-Salgueiro^1^

##### ^1^Coimbra Nursing School, 3046-851 Coimbra, Portugal; ^2^Muroplás - Indústria de Plásticos, 4745-334 Muro, Trofa, Portugal; ^3^Innovation in Polymer Engineering, University of Minho, 4800-058 Guimarães, Portugal; ^4^Federal University of Viçosa, 36570-900 Viçosa, Minas Gerais, Brazil

###### **Correspondence:** Pedro Parreira (parreira@esenfc.pt)

**Background**

The administration of intravenous medication is a frequent practice in health units (approximately 90% of hospitalized patients experience intravenous medication). Venous catheterization (peripheral or central) allows the administration of medication directly into the bloodstream through an inserted catheter. Although the flushing procedure is desirable between and after the administration of intravenous medication, this procedure is often not observed.

**Objective**

In order to reduce this problem, a consortium with complementary experience and skills was created between: Muroplás company, Innovation Polymers Engineering Centre (PIEP) and the Coimbra Nursing School (ESEnfC). The consortium developed the “DUO SYRINGE”, a new Medical Device consisting in a sequential-release double-chamber syringe. The benefits of this new device are several: a greater adherence to flushing by health professionals, a higher level of patient safety and a significant reduction of complications.

**Methods**

After a literature review regarding disease control and prevention, mainly sustained by the Infusion Nurses Society and Royal College of Nursing guidelines, we established important characteristics for this new MD. A focus groups with nurse experts identified and validated the technical characteristics of the device. After developing the preliminary geometry of the syringe through three-dimensional modelling (3D), a new expert panel of end users evaluated the usability of the MD alpha version sustained in Technology Acceptance Model. In the future, we also intend to carry out laboratory tests for safety validation and to perform clinical studies in a hospital environment.

**Results**

We identified a set of characteristics that the MD should incorporate, namely syringe size, volume of the two chambers, cannon syringe configuration, and plunger configuration. There was an alignment between literature review and the experts panel opinion.

**Conclusions**

Clinical practice creates daily new challenges to Nursing and it is crucial to create responses that promote better quality of health care. Identifying problems, creating technological partnerships with companies and technological centres allows to innovate through the development and creation of new MDs. The clinical research with MD allows the evaluation of safety, making clinical practice more effective and safe. This is a new challenge placed to the health professionals. It is desirable to display the intellectual capital available to generate innovations for citizens, reverting in gains to the quality of care.

**Acknowledgements**

This work is funded by the FEDER fund through the Operational Programme for Competitiveness and Internationalisation (COMPETE 2020) within project POCI-01-0247- FEDER-017604.

**Keywords**

Medical Device, Nursing, Innovation, Syringe.

### P106 Nursing home care: nurses' perspective

#### Tatiana Antunes^1^, Teresa Capelo^1^, Anabela Salgueiro-Oliveira^1^, Luciene Braga^2^, Pedro Parreira^1^

##### ^1^Coimbra Nursing School, 3046-851 Coimbra, Portugal; ^2^Federal University of Viçosa, 36570-900 Viçosa, Minas Gerais, Brazil

###### **Correspondence:** Anabela Salgueiro-Oliveira (anabela@esenfc.pt)

**Background**

Nursing home care is a trend of the current society, due to the aging of the population and shorter hospitalization times, for economic reasons and also to prevent infections. In addition, it represents a high potential for improving the quality of care by enabling self-care of patients in their life contexts, involving their families and taking advantage of existing social support networks.

**Objective**

Analyse the scientific production, in the last seven years, concerning domiciliary nursing care, attending to the nurse’s perspective.

**Methods**

The search method used was the integrative literature review. The investigation question was formulated based on the PICO strategy: *What is the nurse’s perspective about nursing home care?* The search was conducted between 22 and 26 May, 2017, in the following databases: SciELO, Medline with full text, CINHAL with full text, Academic Search Complete, Complementary Index. We only looked for primary scientific studies, published in the last six years, in English, Portuguese or Spanish. The selected descriptors were: Home care nursing OR Domiciliary Care Or Home visits) AND Self-care AND Nurse. We accessed 1,293 scientific articles. After reading title, abstract and full text we retained 9 studies for analysis.

**Results**

The mobilization of different nursing competencies is important because of patient’s profile and difficulties associated with the contexts where they perform nursing care [1]. Nurses should demonstrate availability, sensitivity, education, creativity and attend to the care needs of the person and family [2]. The care process success depends on the relationship between nurse and patient and/or family [3]. The unpredictability, the lack of in house resources, distance and work overload are some of the difficulties manifested by the nurses [2, 4].

**Conclusions**

Nurses who provide nursing home care, due to the limited resources they face, will have to carry out a rigorous planning of care, mobilize and inform patients and families about social support networks, as well as to promote self-care.

**References**

1. Sherman H, Forsberg C, Törnkvist A. The 75-year-old persons’ self-reported health conditions: a knowledge base in the field of preventive home visits. Journal of Clinical Nursing. 2012;21:3170-3182.

2. Consoni E, Salvaro M, Ceretta L, Soratto, M. Os desafios do enfermeiro no cuidado domiciliar. Enfermagem Brasil. 2015;14(4):229-234.

3. Gago E, Lopes M. Cuidados domiciliares – interação do enfermeiro com a pessoa idosa/família. Acta Paulista de Enfermagem. 2012;25(1):74-80.

4. Rodrigues A, Soriano J. Fatores influenciadores dos cuidados de enfermagem domiciliários na prevenção de úlceras por pressão. Revista de Enfermagem Referência. 2011;3(5):55-63.

**Keywords**

Patients, Home nursing care.

### P107 Knowledge on pharmacogenomics: gaps and needs of educational resources

#### Andreia Pinho, Marlene Santos

##### Escola Superior de Saúde, Instituto Politécnico do Porto, 4200-072 Porto, Portugal

###### **Correspondence:** Marlene Santos (mes@estsp.ipp.pt)

**Background**

Pharmacogenomics is a science that aims to predict the contribution of genes in an individual's response to the administration of a drug, in order to increase the therapeutic effect and minimize any Adverse Drug Reaction (ADR). The professionals must have knowledge on the subject, however the studies point to a lack of information of the future health professionals, about concepts and applications of Pharmacogenomics.

**Objective**

To compare the study plans of Pharmacy, Pharmaceutical Sciences and Medicine courses at a national level and to find out the existence of topics related to Pharmacogenomics, and to verify the knowledge of the students of the Degree in Pharmacy of Escola Superior de Saúde (ESS) of Porto on the topic “Pharmacogenomics”, identifying gaps and needs of educational resources among students of this course.

**Methods**

A questionnaire-type study was carried out, the first one being applied to the Coordinators of the three courses, at a national level, and another applied to the ESS Pharmacy students about their knowledge about Pharmacogenomics.

**Results**

The courses have an hourly schedule for Pharmacogenomics between 2.5 hours in Pharmacy and 60 hours in Pharmaceutical Sciences. The students' knowledge of this subject went from 15.91% in the first year, to 95.92% and 97.3% in the 3rd and 4th years, respectively. Between 76% and 86% of the students were not able to identify drugs or drug metabolizing enzymes whose activity is influenced by genetic variations. Comparing the 3 courses it can be stated that the workload in the curricular plans is reduced, being especially evident in the course of Pharmacy. There is a significant increase in knowledge about Pharmacogenomics as the years of undergraduate studies progress, and the difference between the 3rd and 4th year is not significant, since this subject is taught only on the 2nd year.

**Conclusions**

The knowledge passed on to undergraduate students and future health professionals is reduced, with an insufficient workload, and does not take place uniformly at a national level. In the case of ESS Pharmacy, there was an increase of knowledge as the degree progresses, despite the few contents taught regarding Pharmacogenomics. In the future, it may be useful to create supplementary courses and trainings for students on this subject.

**Keywords**

Pharmacogenomics, Knowledge, Students, Curricular plan

### P108 Influence of the rs776746 CYP 3A5 gene polymorphism on response to immunosuppressant tacrolimus in patients undergoing liver transplantation: a systematic review

#### Cristiana Rocha, Marlene Santos

##### Escola Superior de Saúde, Instituto Politécnico do Porto, 4200-072 Porto, Portugal

###### **Correspondence:** Marlene Santos (mes@estsp.ipp.pt)

**Background**

Hepatic transplantation is a lifesaving therapy that has been increasing over the years in Portugal. Its success is due largely in part to the use of immunosuppressants, like tacrolimus, the first-line immunosuppressant drug for people undergoing liver transplantation. It is a drug with narrow therapeutic window and great inter-individual variability. This variability is explained in part by polymorphisms of the CYP3A5 gene, which encodes the CYP3A5 metabolizing enzyme. The rs776746 polymorphism affects the CYP3A5 gene and gives rise to a non-functional metabolizing enzyme. The CYP3A5 gene is expressed in both the liver and the gut, that is, the metabolism of tacrolimus is affected by the transplanted liver (donor) genotype, as well as by the gut (receptor) genotype. The identification of polymorphisms becomes important especially in the period immediately after transplantation in order to avoid acute rejection of the organ.

**Objective**

The objective of this work was to review the influence of rs776746 polymorphism of the CYP3A5 gene on pharmacokinetics of tacrolimus.

**Methods**

A systematic review was conducted through the Pubmed database search, from 2000 to 2017. Articles that meet the study query and the inclusion and exclusion criteria were included for review.

**Results**

We selected 23 articles that discuss the influence of the rs776746 polymorphism on the pharmacokinetics of tacrolimus. The evidence suggests that individuals with the CYP3A5*3 (non-expressing) allele have a decreased metabolism of tacrolimus and, consequently, lower blood concentrations of the drug compared to individuals carrying the CYP3A5*1 (expressing) allele. The receptor genotype plays a more important role in the first days after transplantation and the donor genotype becomes more important later when the transplanted organ begins to function properly.

**Conclusions**

This review concluded that regarding hepatic transplantation it is important to identify both the polymorphisms affecting the metabolism of tacrolimus in the donor and recipient genotypes for a more effective dose adjustment, especially in the critical period immediately after transplantation.

**Keywords**

Transplant, Liver, Polymorphism, rs776746, Tacrolimus, CYP3A5.

### P109 The FITWORK European Project - good practices to develop physical activity programs at work

#### Maria Campos, Alain Massart, Carlos Gonçalves, Luís Rama, Ana Teixeira

##### Faculty of Sport Sciences and Physical Education, University of Coimbra, 3040-156 Coimbra, Portugal

###### **Correspondence:** Maria Campos (mjcampos@fcdef.uc.pt)

**Background**

Workplace physical demands have widely changed in the last century. Nowadays, most of the jobs in the European Union (EU) have a low overall energy demand. In this context, the FITWORK project aims to develop good practices to support ergonomics and health by implementing physical activity programs, addressed to reduce specific ergonomic risks at the workplace. This 2-year project (2017-2018) is co-funded by the Erasmus+ Programme of the European Union and coordinated by Instituto de Biomecánica of Valencia (IBV) Spain. The partners are the University of Coimbra (UC); Romtens Foundation, Romania; Eindhoven University of Technology (TU/e); the European Network for Workplace Health Promotion (ENWHP) and KOMAG, Poland (http://fitwork.eu/).

**Objective**

Therefore, the general objective of the project is to promote physical activity at work, awareness of workers and health and safety professionals on the significance of health-enhancing physical activity attending to job demands. To meet this objective, FITWORK will identify good practices in occupational risk prevention through physical activities, including motivational aspects, and best practices for implementing workplace health promotion programs (WHPP).

**Methods**

The workout programs are being implemented in two different organizations, with experimental group and control group, during six months at the Institute of Mining Technology KOMAG, Poland and INNEX S.R.L, Italy, with the following aims: I) to identify and evaluate the worksites and the professional risks within each organization; II) to adapt the WHP Programme to every worksite: identify the most appropriate exercises to carry out in each worksite and when the workers have to perform them; III) to monitor and collect data using specific instruments and report periodically about the development of the programme; IV) to give recommendations related to good practice and aspects for improving the implementation of the program.

**Results**

The primary purposes of the analysis of the results are to validate the effect of the designed physical activity programs and to elaborate good practices guidelines in developing and implementing WHP Programs.

**Conclusions**

There is evidence that behaviour changes are ignited by a complex cocktail of perceived benefits other than health alone, but a lack of evidence still exists on the effectiveness of health promotion activities on productivity, absenteeism or wellbeing. Hence, the desired impact of this European Project is to raise awareness and to engage stakeholders and target groups, sharing solutions and know-how with professional audiences.

**Keywords**

FITWORK, Job demands, Workplace, Physical activity programs, Erasmus+ Programme.

### P110 Adventitious respiratory sounds to monitor lung function in pulmonary rehabilitation

#### Cristina Jácome^1,2^, Joana Cruz^2,3,4^, Alda Marques^2,5^

##### ^1^Center for Health Technology and Information Systems Research, Faculty of Medicine, University of Porto, 4200-450 Porto, Portugal; ^2^Respiratory Research and Rehabilitation Laboratory, School of Health Sciences, University of Aveiro, 3810-193 Aveiro, Portugal; ^3^Center for Innovative Care and Health Technology, Polytechnic Institute of Leiria, 2411-901 Leiria, Portugal; ^4^School of Health Sciences, Polytechnic Institute of Leiria, 2411-901 Leiria, Portugal; ^5^Institute of Biomedicine, University of Aveiro, 3810-193 Aveiro, Portugal

###### **Correspondence:** Cristina Jácome (cristinajacome@ua.pt)

**Background**

Peak expiratory flow (PEF) has been traditionally used to monitor lung function in patients with chronic obstructive pulmonary disease (COPD) before pulmonary rehabilitation (PR) sessions. However, PEF mainly reflects changes in large airways and it is known that COPD primarily targets small airways. Adventitious respiratory sounds (ARS - crackles and/or wheezes), are related to changes within lung morphology and are significantly more frequent in patients with acute exacerbations of COPD. Thus, ARS may be also useful for the routine monitoring of lung function during PR programs.

**Objective**

This study explored the convergent validity of ARS and PEF in patients with COPD.

Methods

Twenty-four (24) stable patients (66 ± 9 years; FEV1 71 ± 19% pred) participating in a PR program were included. Assessments were conducted immediately before one PR session. Presence of ARS (crackles and/or wheezes) at posterior right chest was first assessed by a physiotherapist using a digital stethoscope (ds32a, ThinkLabs, CO, USA). Resting dyspnoea was collected using the modified Borg scale (0-10) and PEF with a peak flow meter (Micro I, Carefusion, UK). Independent t-tests, Pearson and point-biserial correlations were used.

**Results**

ARS were present in 5 participants (20.8%). Patients with ARS had a lower PEF than patients without ARS (294 ± 62 l/min vs. 419 ± 128 l/min; p = 0.048). PEF was negatively correlated with presence of ARS (r = -0.41; p = 0.048). Resting dyspnoea was negatively correlated with PEF (r = -0.41; p = 0.039), but not with ARS (r = 0.21; p = 0.32).

**Conclusions**

Findings suggest that both ARS and PEF offer complementary information before a PR session, but that ARS provide additional information on the patents’ respiratory status. Further research correlating ARS and PEF with patients’ performance and progression during PR is needed to strengthen the usefulness of assessing these parameters in PR.

**Keywords**

Peak expiratory flow, Adventitious respiratory sounds, Crackles, Wheezes, Pulmonary rehabilitation.

### P111 Health care services and their influence on the autonomy and quality of life of the elderly

#### Keila CR Pereira, Silvania Silva, Jefferson L Traebert

##### Universidade do Sul de Santa Catarina, 88137-272, Palhoça, Santa Catarina, Brasil

###### **Correspondence:** Keila CR Pereira (keilarausch@gmail.com)

**Background**

Primary Care (PA) performance can be assessed by Ambulatory Care-Sensitive Conditions (ACSC) [1]. They are health problems whose morbidity and mortality can be reduced through resolute and comprehensive care. The performance and access to the health system can delay the hospitalizations of the elderly with all the risks arising from it [2,3].

**Objective**

Analyse the impact of actions of health care of the elderly on primary care in the ICSAB rate.

**Methods**

Ecological study of the information on the hospitalizations of people over 60 years of age was obtained by hospital admission authorizations (HAA) from the Hospital Information System (HIS), from all municipalities in the state of Santa Catarina (SC) from 2008 to 2015. For the definition of the Primary Care Sensitive Conditions (PCSC), the official report published by the Ministry of Health was used [4]. The crude PCSC rate was calculated by the ratio between the number of PCSC in the elderly and the reference population for the period multiplied by 10,000. Next, the PCSC hospitalization rates for the elderly were standardized by age using the direct method, using the world population [5] as the standard. To soften the historical series, as a function of the oscillation of the points, was calculated the moving average centred in three groups. The analysis was performed through the Joinpoint program, version 4.3.1, used to calculate the variation of the rates of hospitalization of elderly people by age-adjusted PCSC, in the period from 2008 to 2015, resulting in the behaviour of the rate in the period studied for each municipality of Santa Catarina.

**Results**

The analysis showed that for each percentage point of increase in the elderly population rate, one percentage point increases in the annual rate of hospitalization rate for PCSC in the elderly (R^2^ = 0.025). The variables of the performance of attention to the elderly did not show association in the hospitalizations.

**Conclusions**

The individual's lifestyle may be more determinant for a healthy aging than access to services when the individual has aged. In order for services to effectively act as a reducer of hospitalizations in the elderly they must be offered before the establishing of the aging process in the individual.

**References**

1. Caminal J, Starfield B, Sanchez E, Casanova C, Morales M. The role of primary care in preventing ambulatory care sensitive conditions. Eur J Public Health. 2004 Sep;14(3):246-51

2. Santos M. Epidemiologia do envelhecimento. In: Nunes I M; Ferreri R E L; Santos M. Enfermagem em geriatria e gerontologia. Rio de Janeiro: Guanabara Koogan, 2012. pp. 4-8.

3. Silveira R E, Santos A S, Sousa M C, Monteiro T S A. Gastos relacionados a hospitalizações de idosos no Brasil: perspectivas de uma década. Gestão e Economia em Saúde, São Paulo, 2013. Dec;11(4):514-520.

4. BRASIL. Ministério da Saúde. Secretaria de Atenção à Saúde. Departamento de Atenção Básica. Política Nacional de Atenção Básica / Ministério da Saúde. Secretaria de Atenção à Saúde. Departamento de Atenção Básica. – 2012. Brasília.

5. Doll R, Payne P, Waterhouse J. Cancer Incidence in Five Continents: A Technical Report. Berlin: Springer-Verlag (for UICC), 1966.

**Keywords**

Aged, Primary Health Care, Health Promotion, Healthy Aging, Life Style.

### P112 Morphological and functional cardiac changes in TAVI follow-up – evaluation through transthoracic echocardiography

#### Virginia Fonseca, Ana Costa, Inês Antunes, João Lobato

##### Escola Superior de Tecnologia da Saúde de Lisboa, 1990-094 Lisboa, Portugal

###### **Correspondence:** Virginia Fonseca (virginia.fonseca@estesl.ipl.pt)

**Background**

Aortic Stenosis is a valvular disease with increasing prevalence. Transcatheter aortic valve implantation (TAVI) is a treatment option for patients who cannot undergo surgical valve replacement [1,2,3,4].

**Objective**

The aim of this study was to describe and compare morphological and functional cardiac changes, through transthoracic echocardiography, in the follow-up after TAVI.

**Methods**

Patients, with ages between 63 and 85 years old, submitted to TAVI were evaluated by transthoracic echocardiography between 24h to 72h and 1 to 4 months after the procedure. The study variables selected were perivalvular regurgitation, maximum velocity and gradient, left ventricular (LV) function and dimensions, and left atrium (LA) diameter. Statistical analysis of the study variables was made using descriptive statistics, Shapiro-Wilk test, Wilcoxon's test and McNemar test. The results were considered statistically significant when p value < 0.05.

**Results**

It was registered a significant increase in maximum velocity and gradient (p=0.004 and p=0.010, respectively) from the first to the second echocardiogram. There weren’t significant differences in LV ejection fraction, LV telediastolic and telesistolic volumes and in LA diameter. LV índex mass decreased comparing to the first echocardiogram (from 157.92 to 142.28 g/m2), however, this difference wasn’t statistically relevant. The prevalence of regurgitation (80%) was unchanged between evaluations.

**Conclusions**

Transcatheter valve aortic implantation is a relatively new procedure for aortic stenosis treatment, with morphological and functional changes in the heart [3] The studied variables didn’t demonstrate any significant changes, with the exception of maximum velocity and gradient. LV mass decreased in average 15.71 g/m2, and from a clinical perspective, can have an impact in the patient’s prognostic.

**References**

1. Pereira E, Silva G, Caeiro D, Fonseca M, Sampaio F, Fonseca C, et al. Cirurgia cardíaca na estenose aórtica severa: o que mudou com o advento do tratamento percutâneo? Revista Portuguesa de Cardiologia. 2013 Oct;32(10):749–56.

2. Gavina C, Gonçalves A, Almeria C, Hernandez R, Leite-Moreira A, Rocha-Gonçalves F, et al. Determinants of clinical improvement after surgical replacement or transcatheter aortic valve implantation for isolated aortic stenosis. Cardiovascular ultrasound. 2014;12:41.

3. Holmes DR, Mack MJ, Kaul S, Agnihotri A, Alexander KP, Bailey SR, et al. 2012 ACCF/AATS/SCAI/STS Expert Consensus Document on Transcatheter Aortic Valve Replacement. Journal of the American College of Cardiology. Elsevier Inc.; 2012;59(13):1200–54.

4. Leon MB, Smith CR, Mack M, Miller DC, Moses JW, Svensson LG, et al. Transcatheter Aortic-Valve Implantation for Aortic Stenosis in Patients Who 10 Cannot Undergo Surgery. New England Journal of Medicine. 2010 Oct 21;363(17):1597–607.

**Keywords**

TAVI, Transcatheter aortic valve implantation, Aortic stenosis, Transthoracic echocardiography.

### P113 Literacy of family caregivers of people with Alzheimer's disease

#### Rui Moreira^1^, Lia Sousa^2^, Carlos Sequeira^3^

##### ^1^Centro Hospitalar de São João, Pólo Valongo, 4440–563 Valongo, Portugal; ^2^Centro Hospitalar de São João, 4200-319 Porto, Portugal; ^3^Escola Superior de Enfermagem do Porto, 4200-072 Porto, Portugal

###### **Correspondence:** Rui Moreira (ruijorgelm@gmail.com)

**Background**

Increasing population ageing is bringing with it a higher incidence and prevalence of Alzheimer's disease. In addition to incapacitating the sick person this disease has destabilising effects on the family, particularly because they are the caregivers. Therefore, it is important to know how informed family caregivers are about Alzheimer’s disease.

**Objective**

To characterise family caregivers of people with Alzheimer's disease and identify their level of knowledge about the disease.

**Methods**

A quantitative, transversal and descriptive study was carried out. For this, 52 caregivers who are relatives of people with Alzheimer's disease were identified through a convenience sample from both private homes and day-care centres in the North region of Portugal. A questionnaire was administered that contained the sociodemographic characterisation of the caregivers as well as questions that dealt with different facets of the disease, ranging from pathophysiology to intervention strategies. The questionnaire was designed for this study, underpinned by consistent theoretical bases, and was sent to five experts for evaluation. These experts were nurses with experience in gerontology/Alzheimer's disease. The questions are essentially closed, with the only open question coming at the end, relating to the difficulties experienced by the family members in caring for the patient.

**Results**

Focusing on aspects related to literacy, the main results indicate that although 87% of these caregivers know how to define Alzheimer's disease, only about 30% understand what underlies it, while about 50% show some difficulties in identifying risk factors. Most of them (75%) are able to list symptoms of the disease but only half know how to keep the sick person active. It should be noted that only 38% can identify ways to preserve memory and that about 30% of family caregivers are unaware of the purpose of the medication.

**Conclusions**

It was found that there is considerable uncertainty among family caregivers about several facets of Alzheimer's disease. There was also some lack of knowledge about existing resources and support. The study also highlights the fact that the family members questioned do not often ask nurses for information relevant to the care process.

**Keywords**

Health literacy, Family caregivers, Alzheimer’s disease.

### P114 Positive effects of a health promotion program in sedentary elderly with type 2 diabetes

#### Luís Coelho^1,2^, Nuno Amaro^1,2^, João Cruz^1,2^, Rogério Salvador^1,2^, Paulino Rosa^1,2^, Ricardo Gonçalves^1,2^, Rui Matos^1,2^

##### ^1^School of Education and Social Sciences, Polytechnic Institute of Leiria, 2411-091 Leiria, Portugal; ^2^Life Quality Research Centre, 2001-904 Santarém, Portugal

###### **Correspondence:** Luís Coelho (coelho@ipleiria.pt)

**Background**

Diabetes is projected to be the 7th leading cause of death by 2030 by WHO (2017) [1]. Physical Activity along with a healthy diet and medication is one of the crucial options to prevent and control diabetes. About 40% of the Portuguese population aged 25 to 79 years-old presents a condition of diabetes or intermediate hyperglycaemia (Observatório Nacional da Diabetes, 2016) [2].

**Objective**

To examine the impact of a health promotion program in sedentary elderly with type 2 diabetes.

**Methods**

The program consisted on a 30-minute daily walking routine and a weekly educational session regarding healthy behaviours for 8 weeks. All participants were medicated with insulin and anti-diabetics. Twenty-six elderly diabetics (16 male and 10 female) aged 70.1 ± 8.0 years old, were assessed for Body Mass (BM), Body Mass Index (BMI), Systolic and Diastolic Blood Pressure (SBP, DBP respectively), Waist Circumference (WC), and Capillary Glycaemia (CG). Wilcoxon test was used on inferential analysis for repeated measures (pre-post). Significance level was kept at 5%. The effect size for this test was calculated by dividing the z value by the square root of N, being N the number of observations over the two times points [3].

**Results**

All parameters measured values decreased significantly from initial to final moments: BM from 80.8 ± 8.9 kg to 78.2 ± 9.0 kg (p=0.000; r=-0.512), BMI 30.2 ± 3.8 kg/m^2^ to 29.1 ± 3.1 kg/m^2^ (p=0.000; r=-0.543), SBP from 143.4 ± 10.9 mmHg to 134.3 ± 10.4 mmHg (p=0.002; r=-0.426), DBP from 79.5 ± 8.6 mmHg to 75.3 ± 8.7 mmHg (p=0.035; r=-0.292), WC from 100.9 ± 7.8 cm to 96.7 ± 6.7cm (p=0.000; r=-0.584), CG from 182.5 ± 56.3 mg/dl to 124.1 ± 17.7 mg/dl (p=0.000; r=-0.588).

**Conclusions**

The inclusion of physical activity and the awareness of engaging in healthy behaviours seem to complement the medication-based therapeutic in sedentary elderly with type 2 diabetes. Although the physical activity assessment was self-reported, sport sciences can play an important role in the prescription and monitoring of exercise in clinical patients. Multidisciplinary interventions in community health programs are recommended in order to achieve stronger and consistent results. These should include medical practitioners, physiologists and nutritionists.

**References**

1. World Health Organization. Diabetes Fact Sheet. Updated November 2017. http://www.who.int/mediacentre/factsheets/fs312/en/.

2. Diabetes: Factos e Números – O Ano de 2015 − Relatório Anual do Observatório Nacional da Diabetes. Sociedade Portuguesa de Diabetologia. Depósito Legal n.º: 340224/12 ISBN: 978-989-96663-2-0.

3. Pallant J. SPSS Survival Manual: A Step by Step Guide to Data Analysis using SPSS for Windows (3rd ed.). Milton Keynes, UK: Open University Press: 2007.

**Keywords**

Health promotion, Type 2 diabetes, Active life styles, Elderly.

### P115 Prevalence of childhood obesity

#### Fátima Frade^1^, Joana M Marques^1^, Luis Sousa^1^, Maria J Santos^1^, Fátima Pereira^1^, Dora Carteiro^1^, João MG Frade^2,3,4^

##### ^1^Escola Superior de Saúde Atlântica, 2730-036 Barcarena, Portugal; ^2^School of Health Sciences, Polytechnic Institute of Leiria, 2411-901 Leiria, Portugal; ^3^Center for Innovative Care and Health Technology, Polytechnic Institute of Leiria, 2411-901 Leiria, Portugal; ^4^Multidisciplinary Unit for Biomedical Research, Institute of Biomedical Sciences Abel Salazar, University of Porto, 4050-313 Porto, Portugal

###### **Correspondence:** Fátima Frade (ffrade@uatlantica.pt)

**Background**

The prevalence of obesity in children and adolescents has been increasing worldwide [1, 2], having impact on children's physical, psychological and social well-being [3, 4].

**Objective**

To identify the prevalence of childhood obesity worldwide.

**Methods**

A systematic review of the literature began with the question: “*What is the prevalence of childhood obesity worldwide?*” The research was carried out on the EBSCO host, Google Scholar and B-On, on the scientific databases Medline/Pubmed, LILACS, CINAHL, Nursing & Allied Health Collection, Cochrane Plus Collection, MedicLatina and SciELO. The inclusion criteria were: full-text articles, in English, Portuguese or Spanish, published from 2013 to 2017. The Boolean equation used was: (Pediatric Obesity) OR (Overweight) AND (Children) AND (Prevalence). One hundred twenty-two (122) articles were found, of these, 24 were selected after comprehensive reading.

**Results**

Globally, in 2016, there were 41 million children under 5 years of age who were overweight or obese and 340 million children and adolescents aged 5 to 19 years were overweight/obese [5]. In 2013, in the European region the prevalence of overweight/obese people was 31.6%, with 17.7% corresponding to pre-obesity and 13.9% to childhood obesity [6, 7]. In China, the prevalence of overweight people doubled from 13% in 1986, to 27.7% in 2009. In the United States, 31.8% of children were overweight or obese [8]; in New Zealand, 31.7% were overweight and obese, and 2.5% were severely obese [9]. In Mexico City, 30.8% of adolescents, 24.2% of school-age children, 14.5% of latent and 11.5% of children in preschool age were overweight and obese [2]. In Brazil, 30.59% of the children/adolescents studied were overweight, obese or severely obese [8].

**Conclusions**

Childhood obesity is one of the Public Health problems worldwide, it becomes urgent to monitor the problem properly and implement preventive measures to reduce this risk.

**References**

1. Viveiro C, Brito S, Moleiro P. Sobrepeso e obesidade pediátrica: a realidade portuguesa. Rev Port Saúde Pública. 2016;34(1):30-37.

2. Wollenstein-Seligson D, Iglesias-Leboreiro J, Bernárdez-Zapata I, BravermanBronstein A. Prevalencia de sobrepeso y obesidad infantil en uno hospital privado de la ciudad de México. Rev Mex Pediatr. 2016;83(4):108-114.

3. Vásquez-Guzmán M, González-Castillo J, González-Rojas J. Prevalencia de período de sobrepeso y obesidade en escolares. Rev Sanid Milit Mex. 2014;68(2):64-67.

4. Salinas_Martínez A, Mathiew-Quirós, Hernández-Herrera R, GonzálezGuajardo E, Garza-Sagástegui M. Estimación de sobrepeso y obesidad en preescolares – Noramtividad nacional e international. Rev Med Inst Mex Seguro Soc. 2014;52(Supl 1):S23-S33.

5. World Health Organization - WHO. Obesity and Overweight. [online] 2013. [cited 2013 May 15]. Available from: http://www.who.int/ mediacentre/factsheets/fs311/en/.

6. Wijnhoven T, van Raaij J, Breda J. WHO European Childhood Obesity Surveillance Initiative: Implementation of Round 1 (2007/2008) and Round 2 (2009/2010). Copenhagen: WHO Regional Office for Europe,2014. Disponível em: www.euro.who.int/__data/assets/pdf_file/0004/258781/C OSI-report-round1-and-2_final-for-web.pdf

7. Kulaga Z, Gurzkowska B, Grajda A, Wojtylo M, Gózdz M, Litwin M. The prevalence of owerweight and obesity among Polish pre-school-aged children. Dev Period Med. 2016;XX,2:143-149.

8. Cabrera T, Correia I, Oliveira dos Santos D, Pacagnelli F, Prado M, Dias da Silva T, Monteiro C, Fernani D. Analisys of the prevalence of owerwight and obesity and the level of physical activity in chilfren and adolescents of a soudwestern city of São Paulo. JHGD. 2014;24(1):67-66.

9. Farrant B, Utter J, Ameratunga S, Clark T, Fleming T, Simon D. Prevalence of severe obesity among New Zealand adolescents and associations with health risk behaviors and emotional well-Being. J Pediatr. 2013;25:1–7.

**Keywords**

Prevalence, Pediatric Obesity, Overweight, Children.

### P116 The urgency for a nursing intervention towards sexual education at Cape Verde: university students’ perception

#### Sónia Ramalho^1,2^, Carolina Henriques^1,2^, Elisa Caceiro^1^, Maria L Santos^1^

##### ^1^School of Health Sciences, Polytechnic Institute of Leiria, 2411-901 Leiria, Portugal; ^2^Center for Innovative Care and Health Technology, Polytechnic Institute of Leiria, 2411-901 Leiria, Portugal

###### **Correspondence:** Sónia Ramalho (sonia.ramalho@ipleiria.pt)

**Background**

We know today that knowledge about sexuality is essential for young people to live in a society that allows to develop healthy attitudes and behaviours. To that end, health professionals, namely nurses, should be able to Educate for Sexuality in order to contribute for the improvement of affective-sexual relationships among the young; contribute to the reduction of possible negative occurrences resulting from sexual behaviours, such as early pregnancy and sexually transmitted infections (STIs), and contribute to conscious decision-making in the area of health education/sex education [1-4].

**Objective**

To evaluate young people's knowledge about sexuality.

**Methods**

A descriptive, cross-sectional study using a questionnaire consisting of questions related to sociodemographic data, and a questionnaire consisting of twenty questions related to the anatomy of the reproductive system, contraceptive methods and sexually transmitted infections was applied. One hundred and eight (108) young people from the Republic of Cape Verde participated in the study. All formal and ethical procedures were taken into account.

**Results**

The results show that in a sample of 108 university students, 81.5% female, with a mean age of 21.26 years; 1.9% reported having already been forced by a stranger, family member or older person to have sex, and 10.2% reported having had sex after a party, under the influence of alcohol or drugs. As far as knowledge is concerned, it can be said that the level of knowledge of young people regarding the sexual health aspects is satisfactory, safeguarding that the most erroneous questions were those related to: male anatomy (40.7%) and hormonal physiology of women (25.9%). It was found that 32.4% of the university students did not know/did not answer the questions related to female hormonal processes and their functioning when associated with an oral contraceptive.

**Conclusions**

It is essential to know what young people know about sexuality, so that specific nursing interventions can be designed to meet their sexual education needs.

**References**

1. Barbosa A, Gomes-Pedro J. Sexualidade. Lisboa: Faculdade de Medicina da Universidade de Lisboa; 2000.

2. López F, Fuertes A. Para compreender a sexualidade. Lisboa: Associação para o Planeamento da Família; 1999.

3. Epstein D, O’Flynn S, Telford D. Innocence and Experience: Paradoxes in sexuality and education. In: Richardson D, Seidman S, editors. Handbook of Lesbian and Gay Studies. Thousand Oaks, CA: Sage; 2002. p 271-311.

4. Louro GL. Um corpo estranho: Ensaios sobre sexualidade e Teoria Queer. Belo Horizonte: Autêntica Editores; 2004.

**Keywords**

Young people, Knowledge, Sexuality.

### P117 Cape Verde young university students: determinants of whether or not to have sex

#### Carolina Henriques^1,2^, Sónia Ramalho^1,2^, Elisa Caceiro^1^, Maria L Santos^1^

##### ^1^School of Health Sciences, Polytechnic Institute of Leiria, 2411-901 Leiria, Portugal; ^2^Center for Innovative Care and Health Technology, Polytechnic Institute of Leiria, 2411-901 Leiria, Portugal

###### **Correspondence:** Carolina Henriques (carolina.henriques@ipleiria.pt)

**Background**

In Cape Verde there is still no national study regarding the sexual behaviour of its youth, however, data provided by the Cape Verde Association for Family Protection (VERDEFAM) [1], tells us that more than half of Cape Verde adolescents and youngsters start their sexual lives before the age of 16 and the determinants of having or not having sex are not known. In this study, we tried to make some efforts to know some determinants of whether or not to have sex in young Cape Verde university students.

**Objective**

To know the determinants of whether or not to have sex in young Cape Verde university students.

**Methods**

A descriptive, cross-sectional study using a questionnaire consisting of questions related to sociodemographic data and the motivation scale for having or not having sex by Gouveia, Leal, Maroco and Cardoso (2010) [2]. Ninety-eight (98) university students from the Republic of Cape Verde participated in the study. All formal and ethical procedures have been taken into account.

**Results**

Youngsters had a mean age of 21.25 years (SD = 2.76), 62.2% started sexual activity with their boyfriend, and 64.3% used the condom as contraceptive method. Considering the determinants of having sex, the young people that participated in the study considered not important to have sex: “*because my partner wanted*” (51.9%), “*to please my partner*” (56.6%), “*to seduce*” (64.8%), “*for curiosity*” (53.7%) and “*for fun or to play*” (72.2%). In turn, they consider very important not to have sex: for fear of venereal diseases (24.1%); fear of AIDS (37%); fear of pregnancy (28.7%); lack of opportunity or inability to find a partner (25%) and because they did not know their partner long enough (46.3%).

**Conclusions**

The Cape Verde youth that participated in the study emphasizes the importance of health-related and safe relationships, not emphasizing both the desire of the other and pleasure for pleasure, factors that are strongly associated with the determinants of whether or not to have sex.

**References**

1. Cape Verdean Association for the Protection of the Family (VERDEFAM). Recovered from http://www.verdefam.cv/

2. Gouveia P, Leal I, Maroco J, Cardoso J. Escala de Motivação para fazer e para não fazer Sexo. In: Leal I, Maroco J, editors. Avaliação em sexualidade e parentalidade. Porto: LivPsic; 2010. p. 84-99.

**Keywords**

Young; Sex; Determinants; Cape Verde.

### P118 Attitude of Cape Verdean young university students towards sexuality

#### Carolina Henriques^1,2^, Sónia Ramalho^1,2^, Maria L Santos^1^, Elisa Caceiro^1^

##### ^1^School of Health Sciences, Polytechnic Institute of Leiria, 2411-901 Leiria, Portugal; ^2^Center for Innovative Care and Health Technology, Polytechnic Institute of Leiria, 2411-901 Leiria, Portugal

###### **Correspondence:** Carolina Henriques (carolina.henriques@ipleiria.pt)

**Background**

Cape Verde, a country with about 500,000 inhabitants, has a markedly young population, a fact that places special responsibility on the role of local health professionals, in the contexts of sexual and reproductive health, where sexuality issues are included.

**Objective**

To evaluate the attitudes of Cape Verdean university students towards sexuality.

**Methods**

A descriptive, cross-sectional study using a questionnaire consisting of questions related to sociodemographic data and the sexual attitudes scale of Gouveia, Leal, Maroco and Cardoso (2010) [1]. One hundred and eight (108) university students from the Republic of Cape Verde participated in the study. All formal and ethical procedures have been taken into account.

**Results**

The results show that participants of this cross-border research had a mean age of 21.26 years (SD = 2.93), were mostly female (81.5%) and started their sexual activity with a mean age of 17.37 years (SD = 1.31). As far as sexual attitudes are concerned, 11.1% agrees that “*one does not have to be committed to the person to have sex with her/him*”; 18.5% agree that “*casual intercourse is acceptable*”; 76.9% totally disagree with “*I would like to have sex with many partners*”; 74.1% disagree completely that “*it is right to have sex with more than one person during the same time period*” and 36.1% agree that “*sex is primarily a physical activity*”. 10.2% of young people agree that “*sex, by sex alone, is perfectly acceptable*”, 33.3% agree that *“sex is primarily a bodily function, just like eating*”. Regarding the permissiveness of university students in relation to occasional/non-engaging sex, they present a significant level of agreement (M = 14.44, Sd = 3.66, Xmax = 24.00) which relates to instrumentality (M = 11.93, SD = 2.62, Xmax = 19.00).

**Conclusions**

Data shows that young Cape Verdean university students seek to have sexual relations with respect for their partner, although they agree with sex without commitment, closely associated with the vision of the sexual act as a corporal function response.

**References**

1. Gouveia P, Leal I, Maroco J, Cardoso J. Escala de Atitudes Sexuais–Versão adolescentes (EAS-A). In: Leal I, Maroco J, editors. Avaliação em sexualidade e parentalidade. Porto: LivPsic; 2010. p. 58-72.

**Keywords**

Youth, Sexuality, Attitudes, Cape Verde.

### P119 Central auditory processing and sleep deprivation

#### Diogo Garcia, Carla Silva

##### Audiology Department, Coimbra Health School, Polytechnic Institute of Coimbra, 3046-854 Coimbra, Portugal

###### **Correspondence:** Diogo Garcia (diogojorge04@gmail.com)

**Background**

Auditory Processing is a natural process of taking in sound through the ear and having it travel to the language area of the brain to be interpreted. Sleep deprivation may influence this process.

**Objective**

To evaluate the impact of sleep deprivation, for a 24-hour period, on central auditory processing in healthy young adults.

**Methods**

Fourteen (14) healthy young adults were selected, 9 of which (64.3%) were female and 5 (35.7%) were male, aged 18-29 years. The subjects were submitted to audiological evaluation using tonal audiometry and the threshold of 1 in 1 dB was obtained in the frequencies of 250Hz, 500Hz, 1000Hz, 2000Hz, 4000Hz and 8000Hz, alternating disyllabic dichotic listening test Staggered Spondaic Words Test (SSW). The evaluation of central auditory processing, as well as the determination of the auditory threshold was performed in two situations: without sleep deprivation and after 24 hours of sleep deprivation.

**Results**

At the level of the Audiogram, the reduction of the auditory thresholds after sleep deprivation in the frequencies of 500Hz and 1000Hz, around 3dB, increased in the frequency of 250Hz and 4000Hz, between 1 and 9dB, and remained in the right ear in frequencies of 2000Hz and 8000Hz. In the right ear, none of the differences found were statistically significant. In the left ear the auditory threshold increased at the frequency of 2000Hz about 5dB, and decreased in the frequencies of 250Hz, 500Hz, 1000Hz, 4000Hz and 8000Hz between 1 and 8dB, without statistically significant differences. In the SSW, there was a slight decrease in the percentage of correct answers in both ears, as well as in the percentage of total hits, after sleep deprivation. In none of the ears there were statistically significant differences in SSW results before and after 24 hours of sleep deprivation.

**Conclusions**

The results demonstrate that there are no statistically significant changes before and after 24h of sleep deprivation, both at the level of the Auditory Threshold and in the SSW.

**Keywords**

Central Auditory Processing (PAC), Sleep Deprivation, Staggered Spondaic Words Test (SSW), Audiological Evaluation.

### P120 Missed nursing care: incidence and predictive factors - integrative literature review

#### Ivo CS Paiva^1,2^, Isabel M Moreira^1^, António FS Amaral^1^

##### ^1^Nursing School of Coimbra, 3046-851 Coimbra, Portugal; ^2^Department of Internal Medicine and Palliative Care, Portuguese Oncology Institute of Coimbra Francisco Gentil, 3000-075 Coimbra, Portugal

###### **Correspondence:** Ivo CS Paiva (ivocsoarespaiva@gmail.com)

**Background**

The increasing complexity and demand in health care, together with patients’ changing needs, put into evidence the need for nurses to focus their interventions on people’s real needs, thus rethinking their role. In this context, unfinished, delayed, or missed nursing care (MNC), which is a strong indicator of health care quality, can compromise quality care and patient safety.

**Objective**

To identify the most common MNC, as well as its predictors and strategies to prevent its occurrence.

**Methods**

A systematic literature review was conducted on studies available in the EBSCOhost and B-on databases. Twenty-four articles were selected based on predefined criteria. All articles were published between 2012 and 2017.

**Results**

The studies showed that nurses and patients have different perceptions about MNC. Autonomous interventions related to early mobility and walking, repositioning every 2 hours, or oral and body hygiene are more often missed than interdependent interventions. [1-5]. Medication administration within 30 minutes of prescription, planning and update of care plans, vital signs monitoring, and treatment effectiveness assessment also emerged as MNC [5-7]. Predictive factors are associated with the patient (health status/workload) [2, 5]; the nurse (interruptions by other professionals or patients’ relatives) [4, 8]; the materials or equipment (late deliveries) [9], or the hospital’s health care policies (management/leadership and staffing) [10]. The nurse-patient/family communication emerged as MNC, whereas the nurse-other health professional communication emerged as a predictive factor because teamwork and its effectiveness are compromised [2, 11]. Nurses’ personal interests and moral sense can influence the occurrence of MNC. Therefore, the strategies used to reduce the incidence of MNC should be adjusted to the needs of each setting [2, 5].

**Conclusions**

Although MNCs are being explored internationally, it is an understudied topic in Portugal, which may be explained by the punitive error reporting culture. The findings cannot be generalized due to the diversity of studies. Thus, the phenomenon of MNC and its impact on patient/family prognosis should be explored in different settings, with the purpose of achieving excellence in health care and ensuring that people recognize the importance of health care.

**References**

1. Bruyneel K, Li B, Ausserhofer D, Lesaffre E, Dumitrescu I, Smith H, etal. Organization of Hospital Nursing, Provision of Nursing Care, and Patient Experiences with Care in Europe. Med Care Res Rev. 2015;72(6):643-664.

2. Chapman R, Rahman A, Courtney M, Chalmers C. Impact of teamwork on missed care in four Australian hospitals. J Clin Nurs. 2017;26(1-2):170-181.

3. Cho S, Kim Y, Yeon K, You S, Lee I. Effects of increasing nurse staffing on missed nursing care. Int Nurs Rev. 2015;62(2):267-274.

4. Kalisch B, Xie B, Dabney B. Patient Reported Missed Nursing Care Correlated With Adverse Events. Am J Med Qual. 2014;29(5):415-422.

5. Papastavrou E, Charalambous A, Vryonides S, Eleftheriou C, Merkouris A. To what extent are patients' needs met on oncology units? The phenomenon of care rationing. Eur J Oncol Nurs. 2016;21:48-56.

6. Ball J, Murrels T, Rafferty A, Morrow E, Griffiths P. ‘Care left undone’ during nursing shifts: associations with workload and perceived quality of care. BMJ Qual Saf. 2014, 23:116-125.

7. Schubert M, Ausserhofer D, Desmedt M, Schwendimann R, Lessafre E, Li B, Geest S. Levels and correlates of implicit rationing of nursing care in Swiss acute care hospitals - A cross sectional study. Int J Nurs Stud. 2013;50(2):230-239.

8. Cho S, Mark B, Knafl G, Chang H. Yoon H. Relationships Between Nurse Staffing and Patients’ Experiences, and the Mediating Effects of Missed Nursing Care. J Nurs Scholarsh. 2017;49(3):347-355.

9. Moreno-Monsiváis M, Moreno-Rodríguez C, Interial-Guzmán M. Missed Nursing Care in Hospitalized Patients. Aquichán. 2015;15:318-328.

10. Dehghan-Nayeri N, Ghaffari F, Shali M. Exploring Iranian nurses’ experiences of missed nursing care: a qualitative study: a threat to patient and nurses' health. Med J Islam Repub Iran. 2015;29:276.

11. Bragadóttir H, Kalisch B, Tryggvadóttir G. Correlates and predictors of missed nursing care. J Clin Nurs. 2017;26(11-12):1524-1534.

**Keywords**

Missed Nursing Care, Delayed Nursing Care, Unfinished Nursing Care.

### P121 The meaning of the family for future family nurses

#### João MG Frade^1,2^, Carolina Henriques^1,2^, Célia Jordão^1^, Clarisse Louro^1^

##### ^1^School of Health Sciences, Polytechnic Institute of Leiria, 2411-901 Leiria, Portugal; ^2^Center for Innovative Care and Health Technology, Polytechnic Institute of Leiria, 2411-901 Leiria, Portugal

###### **Correspondence:** Carolina Henriques (carolina.henriques@ipleiria.pt)

**Background**

The family must be understood as a natural context of growth, involving the notion of complexity, of blood and/or affective bonds, generating love, but also suffering. The systemic view of the family considers the family as a whole, in which its members interact with one another, being that the imbalance of the system can cause imbalance in the individual and vice versa. The Family Nurse can be the reference professional, ensuring for the specialized accompaniment of the family, as a care unit, throughout the life cycle.

**Objective**

To know the perception about “family” and “family health nursing” by future family nurses.

**Methods**

A descriptive, cross-sectional study using a questionnaire consisting of questions related to sociodemographic data, and open questions regarding conceptual understanding of family and family health nursing. A total of 13 nurses participated in the study to develop skills in the field of family health nursing. All formal and ethical procedures have been taken into account.

**Results**

Nurses report that they (100%) never attended before any training course in the field of family health nursing, recognizing the importance of knowing who the family members are (92.3%). 38.5% disagrees that the presence of family members alleviates their workload and 23.1% state that the presence of family members makes them feel that they are being evaluated. Mostly, the family is defined as a group of people with a common link (n= 10). Family health nursing is seen as: caring for the family; personalizing and integrating the nursing care provided to the person and to the family; the existence of a family nurse who knows all its members; the one that equates the care to the individuals according to the characteristics of the family; a health support of the group; a nurse that focuses mainly on the patient and on the family, in partnership with the respective family doctor.

**Conclusions**

Given the data obtained, it is understood that the conceptualization of family and family health nursing should be clarified so that nurses can focus on the internal dynamics of the families and their relationships, family structure and functioning. This can be as such, that the relationship of the different subsystems, of the family as a whole and with the surrounding environment, generates changes in the intrafamilial processes and in the interaction of the family with the environment.

**Keywords**

Family, Nurses, Family nursing.

### P122 The dizziness in patients with cochlear implants

#### Ana Rosado, Carla Silva

##### Audiology Department, Coimbra Health School, Polytechnic Institute of Coimbra, 3046-854 Coimbra, Portugal

###### **Correspondence:** Ana Rosado (anarnamoradorosado@gmail.com)

**Background**

The cochlear implant (CI) is a surgical method widely used today for the (re)habilitation of individuals with bilateral, severe to profound sensorineural hearing loss. Due to the proximity between the cochlea and the surrounding structures without vestibular system, and a delivery endolymphatic fluid, there may be a relationship between this form of (re)hearing activation and vestibular dysfunctions presented after a cochlear implant placement surgery.

**Objective**

Through a systematic review of the literature, we intend to determine the post-surgical vestibular changes existing in individuals submitted to CI placement, as well as to understand the procedures involved in this evaluation.

**Methods**

Scientific articles were searched according to the search engines B-on, PubMed, Scielo and ScienceDirect, obtaining a total of 48 articles. After applying the inclusion criteria, 12 articles were analysed in more detail, 4 of which were selected to integrate this systematic review of the literature.

**Results**

After a systematic review of all articles, we found data reporting a decrease in the amplitude of the response wave vestibular evoked myogenic potential (cVEMP), an increase in average scores in the Dizziness Handicap Inventory (DHI), and an alteration of the classification of the caloric response (normal → hyporeflexia and/or hyporeflexia → arreflexia), in the postoperative period. However, it is not possible, to conclude that these vestibular changes are directly related to the placement of the CI. The use of a protocol evaluating vestibular function at the pre- and post-surgical periods was not verified, nor was the anatomical relationship between the cochlea and the vestibule and semi-circular canals (SSC).

**Conclusions**

We conclude that the studies directed to the vestibular evaluation during the protocol of placement of CI are reduced and without conclusions on the long term, since this follow up was done within a short time after its placement. There is a lack of a basic protocol, which helps the health professionals involved in the process to evaluate the vestibular system, as well as the sensitization of these to the possible influence of the CI insertion surgery in this system.

**Keywords**

Audiology, Vestibular Disorders, Dizziness, Cochlear Implant, cVEMP.

### P123 Practice of episiotomy during labour

#### Manuela Ferreira^1^, Onélia Santos^2^, João Duarte^1^

##### ^1^Instituto Politécnico de Viseu, 3504-510 Viseu, Portugal; ^2^Centro Hospitalar Cova da Beira, 6200-251 Covilhã, Portugal

###### **Correspondence:** Manuela Ferreira (mmcferreira@gmail.com)

**Background**

The World Health Organization (WHO, 1996) recommends the use of limited episiotomy since no credible evidence that its widespread use or routine practice has a beneficial effect.

**Objective**

To demonstrate scientific evidence of the determinants of the practice of selective episiotomy in women with normal/eutocic delivery; to identify the prevalence of episiotomy; and analyse the factors (sociodemographic variables, variables related to the new-born, contextual variables of pregnancy and contextual delivery) that influence the practice of episiotomy.

**Methods**

The empirical study I (part I) followed the systematic methodology of literature review. A search of studies published between January 2008 and December 23, 2014, was made in the databases: EBSCO, PubMed, Scielo, RCAAP. The studies found were evaluated taking into account the inclusion criteria previously established. Two reviewers assessed the quality of the studies to include using a prospective, randomized and controlled grid for critical evaluation. After critical evaluation of the quality of the study, 4 research articles that obtained a score between 87.5% and 95% were included.

The empirical study II (part II) is part of a quantitative, cross-sectional, descriptive, retrospective study, developed in the Obstetrics Service of Hospital Cova da Beira, according to a non-probability sampling process for convenience (n = 382). Data collection was executed by consulting medical records of women aged ≥ 18 years who had a vaginal delivery with a live foetus after 37 weeks of gestation.

**Results**

Evidences that episiotomy should not be performed routinely and that should be limited to specific clinical situations were found. While performing selective episiotomy, when compared to routine episiotomy, a lower risk of trauma of the posterior perineum was associated with less need for suturing and fewer healing complications. Studies carried out on a sample of 382 women, aged between 18-46 years, which did not carry episiotomy in 41.7% of the cases, pointed to the relevance of selective episiotomy. Among the sample, a significant number of women with eutocic delivery (80.5%), with suture (95.0%), grade I lacerations (64.9%), perineal pain (89.1%) were subjected to episiotomy (58.3%). Among the group of women undergoing episiotomy (91.4%), most of the babies born presented a normal weight (92.3%).

**Conclusions**

In view of these results and based on the available scientific evidence recommendations given for several years, that a more a selective use of episiotomy should be made; it is suggested that health professionals should be more awake to this reality, so we can override the resistance and barriers against the selective use of episiotomy.

**Keywords**

Eutocic Childbirth, Selective Episiotomy, Routine episiotomy.

### P124 RNAO’s Best Practice Guidelines in the nursing curriculum – implementation update

#### Ana V Antunes^1^, Olga Valentim^1^, Fátima Pereira^1^, Fátima Frade^1^, Cristiana Firmino^1^, Joana Marques^1^, Maria Nogueira^1^, Luís Sousa^1,2^

##### ^1^Escola Superior de Saúde Atlântica, 2730-036 Barcarena, Portugal; ^2^Hospital Curry Cabral, Centro Hospitalar Lisboa Central, 1069-166 Lisboa, Portugal

###### **Correspondence:** Ana V Antunes (vantunes@uatlantica.pt)

**Background**

In the last 30 years Nursing Education in Portugal went through several changes which directly impacted on the professional development model and on the recognition of nurse’s scope of practice. Since the Declaration of Bolonha, nursing students are provided with a more practical and profession oriented nursing training [1, 2]. As our professionals’ skills become more recognized in the global health market, also our need to improve education and professional development rises. The best way to enhance the quality of practice education provided to undergraduate nursing students and to improve clinical outcomes is by enriching the academic *curriculum* with evidence-based nursing practices (EBNP) [3]. The Best Practice Guidelines Program (BPGP) was developed by the Registered Nurses Association of Ontario (RNAO) to support EBNP [4].

**Objective**

Provide an update on the process of implementation of RNAO’s Best Practice Guidelines (BPGs) in the nursing curriculum.

**Methods**

The implementation process was supported by the RNAO’s Toolkit for Implementing Best Practice Guidelines (BPGs) [5]. It is a comprehensive resource manual, grounded in theory, research and experience, that provides practical processes, strategies and tools to both Providers, Educational Institutions, Governments, and others committed to implement and evaluate BPGs.

**Results**

The BPGs selection and implementation brought together some of the suggested activities from the six steps of the manual. It resulted in the selection of three clinical guidelines (Engaging Clients Who Use Substances [6]; Prevention of Falls and Fall Injuries in the Older Adult [7]; Primary Prevention of Childhood Obesity [8] and a Healthy Work Environments Guideline (Practice Education in Nursing, [9]). We considered two main areas to intervene, in order to address the challenge of generating scientific evidence for nursing practice: the academic and the clinical setting (partner institutions, where students undertake their clinical practice). The implementation process included three fundamental players from both settings: professors, nursing students and clinical nursing instructors. To evaluate our performance and measure the improvements, we created structure, process and outcome indicators for each guideline. Data collection tools were first used in the curricular units that precede clinical teaching, and results will be processed and analysed.

**Conclusions**

Professors, students and partner institutions were successfully engaged in the initiative. We are investing in an action plan to embed the evidence-based practice culture, through an orientation program for clinical nursing instructors. The strategy is to strengthen the relationship with providers in order to standardize evidence-based procedures and improve both nurses’ education and quality of care.

**References**

1. Hvalič-Touzery S, Hopia H, Sihvonen S, Diwan S, Sen S, Skela-Savič B. Perspectives on enhancing international practical training of students in health and social care study programs-A qualitative descriptive case study. Nurse Educ Today. 2017;48:40-47.

2. Arrigoni C, Grugnetti AM, Caruso R, Gallotti ML, Borrelli P, Puci M. Nursing students’ clinical competencies: a survey on clinical education objectives. Ann Ig. 2017;29(3):179-188.

3. Drayton-Brooks SM, Gray PA, Turner NP, Newland JA. Building clinical education training capacity in nurse practitioner programs. J Prof Nurs. 2017;33(6):422-428.

4. Athwal L, Marchuk B, Laforêt, Fliesser Y, Castanza J, Davis L, LaSalle M. Adaptation of a Best Practice Guideline to Strengthen Client‐ Centered Care in Public Health. Public Health Nursing. 2014 Mar 1;31(2):134-43.

5. Registered Nurses’ Association of Ontario (RNAO). Toolkit: Implementation of best practice guidelines (2nd ed.). Toronto, ON: Registered Nurses’ Association of Ontario. 2012.

6. Registered Nurses’ Association of Ontario (RNAO). Engaging Clients Who Use Substances. Toronto, ON: Registered Nurses’ Association of Ontario. 2015.

7. Registered Nurses’ Association of Ontario (RNAO). Prevención de caídas y lesiones derivadas de las caídas en personas mayores. (Revisado). Toronto, ON: Asociación Profesional de Enfermeras de Ontario. 2015.

8. Registered Nurses’ Association of Ontario (RNAO). Primary Prevention of Childhood Obesity (Second Edition). Toronto, ON: Registered Nurses’ Association of Ontario. 2014.

9. Registered Nurses’ Association of Ontario (RNAO). Practice Education in Nursing. Toronto, ON: Registered Nurses’ Association of Ontario. 2016.

**Keywords**

Evidence-Based Nursing, Nursing Education, Substance-Related Disorders, Accidental Falls, Pediatric Obesity.

### P125 Swimming practice and hearing disorders

#### Mara Rebelo, Carla Silva

##### Audiology Department, Coimbra Health School, Polytechnic Institute of Coimbra, 3046-854 Coimbra, Portugal

###### **Correspondence:** Mara Rebelo (mararebelo@hotmail.com)

**Background**

Health assessment, promotion and prevention are a crucial pillar in the face of emerging trends and threats. In fact, disease prevention is surely the way to go. This is not to say that we should neglect the treatment of disease, but rather that we must make a clear bet on its prevention according to our daily behaviour and the circumstances in which we live. The practice of swimming is recommended, especially for children, since it presents benefits, namely in the treatment of respiratory diseases, allergic problems and in the improvement of motor coordination and/or postural problems. However, during swimming lessons, children are exposed to numerous harmful risk factors to the middle ear and the outer ear. Therefore, preventive measures are essentially the eviction of some factors that increase the associated risks.

**Objective**

To analyse possible audiological changes in swimming children, aged between 3 and 10 years. It is also intended to sensitize educators about the risk factors associated with possible hearing loss, even if temporary.

**Methods**

The sample consisted of 56 students from the School Group of Benedita - Municipality of Alcobaça. All children underwent: otoscopy, tympanogram and tonal audiogram of screening, after the prior authorization of their legal representatives for their participation in the study.

**Results**

Among the 112 otoscopies performed were: 85.7% without alterations in the right ear and 83.9% in the left ear. Of the 56 individuals participating in the sample, it was verified that 17.9% had audiological alterations, of which 20.7% were swimming practitioners and 14.8% did not practice swimming. Of the 29 swimming practitioners, 6.9% had a type B tympanogram, of which 7.1% in the 3 to 5 age group, and 6.7% in the 6 to 10 age group.

**Conclusions**

It seems unlikely that swimming practice is directly related to the increase of middle ear secretions, and possible auditory alterations, given the minimal differences observed between swimmers and non-swimmers. Swimming practice beyond the malefic factors can to some extent be associated with beneficial factors, both in promotion and in the prevention of middle ear health.

**Keywords**

Hearing Loss, Swimming, Children, Middle ear infections, Audiological changes.

### P126 The impact of physical activity on spirometric parameters in non-institutionalised elderly people

#### Fernanda Silva^1^, João Petrica^1,3^, João Serrano^1,3^, Rui Paulo^1,2^, André Ramalho^1,2^, José P Ferreira^4^, Pedro Duarte-Mendes^1,2^

##### ^1^Department of Sports and Well-being, Polytechnic Institute of Castelo Branco, 6000-266 Castelo Branco, Portugal; ^2^Centro de Estudos em Educação, Tecnologias e Saúde, Instituto Politécnico de Viseu, 3504-510 Viseu, Portugal; ^3^Research on Education and Community Intervention, 4411-801 Arcozelo – Vila Nova de Gaia, Portugal; ^4^Research Unit for Sport and Physical Activity, University of Coimbra, 3040-248 Coimbra, Portugal

###### **Correspondence:** Fernanda Silva (f.m.a.s_298@hotmail.com)

**Background**

Physical activity decreases as a result of the ageing process. Therefore, the elderly tend to spend more time adopting sedentary behaviours [1]. The respiratory system also undergoes progressive involution with age, resulting in anatomical and functional alterations [2]. The positive relationship between physical activity and spirometric parameters has been thus confirmed [3]. It is recommended that the elderly should accumulate at least 30 minutes of moderate- to vigorous-intensity physical activity per day [4].

**Objective**

The aim of this paper is to verify the existence of differences regarding spirometric values between two groups of people: those who complied and those who did not comply with the Global Recommendations on Physical Activity for Health [4].

**Methods**

The current study included 36 participants of an average age of 72.28, both male and female (SD= 6.58). The group which has fulfilled the recommendations included 16 elderly individuals (53.76 ± 24.39 minutes); the group which has not fulfilled the recommendations included 20 elderly individuals (15.95 ± 7.79 minutes). Physical activity was assessed using accelerometry (ActiGraph®, GT1M model, Fort Walton Beach, Florida, EUA). Data were recorded for three consecutive days and 600 minutes of daily recording, at least. Spirometry tests were performed using the Cosmed® Microquark spirometer. The following parameters were analysed: Forced Vital Capacity (FVC), Forced Expiratory Volume in one second (FEV1), Peak Expiratory Flow (PEF) and Expiratory Volume in one second and Forced Vital Capacity rate (FEV1/FVC). In order to analyse data, descriptive and inferential statistics were used. The Shapiro-Wilk test was applied to assess normality, whereas the Mann-Whitney test and the t-Test were used for independent samples.

**Results**

The group which has fulfilled the recommendations on physical activity has achieved better percentage spirometric values in VEF1, PEF e VEF1/FVC. However, significant differences were only found in VEF1/FVC% (p= 0.023).

**Conclusions**

The results therefore suggest that compliance with the Global Recommendations on Physical Activity for Health is associated with better VEF1/FVC% values in non-institutionalised elderly people.

**Acknowledgements**

This work was supported by the Portuguese Foundation for Science and Technology (FCT; Grant Pest – OE/CED/UI4016/2016).

**References**

1. Matthews CE, Moore SC, Sampson J, Blair A, Xiao Q, Keadle SK, Hollenbeck A, Park Y. Mortality Benefits for Replacing Sitting Time with Different Physical Activities. Med Sci Sports Exerc. 2015 ;47:1833-1840.

2. Lalley PM. The aging respiratory system-Pulmonary structure, function and neural control. Respir Physiol Neurobiol. 2013;187:199-210.

3. Nawrocka A, Mynarski W. Objective Assessment of Adherence to Global Recommendations on Physical Activity for Health in Relation to Spirometric Values in Nonsmoker Women Aged 60-75 Years. J Aging and Phys Act. 2016;25:123-127.

4. WHO. Global Recommendations on Physical Activity for Health. Switzerland: World Health Organization; 2011. Available from: http://apps.who.int/iris/bitstream/10665/44399/1/9789241599979_eng.pdf

**Keywords**

Accelerometry, Recommendations on physical activity, Elder, Spirometry.

### P127 Predictors of abandonment of exclusive breastfeeding before 6 months

#### Cristiana Afonso, Cristiana Lopes, Fernanda Pais, Suzi Marques, João Lima

##### School of Health Sciences, Polytechnic Institute of Leiria, 2411-901 Leiria, Portugal

###### **Correspondence:** Cristiana Afonso (cristiana_oa@hotmail.com)

**Background**

The World Health Organization (WHO) recommends exclusive breastfeeding up to 6 months of age, considering its nutritional, immunological, psychological and economic benefits. However, according to the *Inquérito Alimentar Nacional e de Atividade Física*, only about 46% of the children were exclusively breastfed for less than 4 months and only 21.6% for 6 months or more.

**Objective**

This study aimed to identify the predictors that most influence mothers in the decision to not to exclusively breastfeed until 6 months.

**Methods**

A review of the literature on the main determinants of exclusive breastfeeding was carried out, and an inquiry was then compiled with the identified predictors. In this context, mothers who had not breastfed or had not exclusively breastfed until 6 months of age were asked to choose the determinant that best suited their situation. The inquiry was made available through several online platforms, some of which aimed at childcare. It was considered inclusion criteria to be a mother.

**Results**

A total of 1,685 mothers were questioned, 1,644 (97.57%) of whom breastfed and 866 (51.39%) exclusively breastfed up to 6 months. The predictors most frequently identified by mothers who had not breastfed or had not breastfed exclusively until 6 months (819 mothers) were “*work-constrains related to work schedule to breastfeeding*” (33.1%), “*milk drying*” (27.1%), “*few daily periods of breastfeeding*” (12.9%), “*personal condition*” (11.5%), “*missing or few conditions for breastfeeding at work*” (11.2%).

**Conclusions**

This work allowed the identification of the predictors of non-breastfeeding or of its non-exclusivity until 6 months, observing a strong contribution of the working conditions to this problem. Knowledge of this reality may be important to develop policy measures to act against this trend.

**Keywords**

Exclusive breastfeeding, Predictors, Inquiry.

### P128 Auditory training in children and youngsters with learning disabilities

#### Mariana Araújo, Cristina Nazaré

##### Coimbra Health School, Polytechnic Institute of Coimbra, 3046-854 Coimbra, Portugal

###### **Correspondence:** Mariana Araújo (mariana_msda@hotmail.com)

**Background**

Hearing has a fundamental role in the learning process, and studies had showed that some children and youngsters, with normal hearing, could present auditory processing disorders with possible implications in the learning process. It is also known that not all learning problems are due to auditory processing disorders and all cases of auditory processing disorders do not lead to learning problems. Studies also point out that an adequate and personalized auditory training may be a viable option in the rehabilitation of auditory information processing in the central nervous system (brain neuroplasticity training), being the early assessment and intervention important to minimize the associated consequences, such as the possible difficulties on the learning process.

**Objective**

To analyse the influence that auditory training has on the improvement of auditory processing disorders of children and youngsters, with learning disabilities.

**Methods**

For this purpose was conducted a systematic literature review with search of scientific papers on electronic databases B-on, PubMED, ScienceDirect and SciELO with keywords such as auditory training, auditory processing, auditory processing disorders, learning disabilities, learning difficulties, children and youngsters (in Portuguese, English or Spanish). For this review were established inclusion criteria such as: publication type and date (original articles available since 2007); sample (in accordance with our purpose) and tests used (to evaluate and training the auditory processing).

**Results**

After the search strategies five articles in accordance with the pre-established inclusion criteria were selected from out of the 127 found.

**Conclusions**

The auditory training is effective in the rehabilitation of auditory processing disorders in children and youngsters with learning disabilities, and studies showed that a specific diagnosis of the abilities affected is fundamental, in order to achieve the perfect and most efficient training plan for each individual, as well as, a continuous re-evaluation to adjust the training. Since it is a complex interaction between those disorders it is still necessary to carry out further studies in this area that should try to establish some guidelines and try to clarify the plan of the auditory training program.

**Keywords**

Auditory training, Auditory processing disorders, Learning disabilities, Children, Youngsters.

### P129 Inadequated environmental sanitation diseases (IESDs) in Porto Alegre – RS/ Brasil

#### Rita C Nugem^1^, Roger S Rosa², Ronaldo Bordin^3^, Caroline N Teixeira^4^

##### ^1^Health Services and Performance Research, Claude Bernard Lyon University, 69100 Lyon, France; ^2^Department of Social Medicine, Medical School, Rio Grande do Sul Federal University, 90040-060 Porto Alegre, Brazil; ^3^Administration School, Rio Grande do Sul Federal University, 96201-900 Rio Grande do Sul, Brazil; ^4^Fundação Hospitalar Getúlio Vargas, 93210-020 Sapucaia do Sul, Rio Grande do Sul, Brazil

###### **Correspondence:** Rita C Nugem (rcnugem@gmail.com)

**Background**

Countries in Europe and North America managed to control and eradicate most of the infectious-parasitic diseases that occurred in the first half of the twentieth century [1]. Nevertheless, infectious and parasitic diseases are still present in certain metropolitan areas of Brazil, despite the increased prevalence of chronic diseases. This work aims to present the general aspects of the situation of diseases related to inadequate environmental sanitation (IESDs) and of the sanitation policy of Porto Alegre.

**Objective**

The general objective was to examine the public policy for environmental sanitation in Porto Alegre, and, the specific objectives were: I) to analyse the relationship between indicators of poverty and inadequate environmental sanitation and the occurrence of diseases related to inadequate environmental sanitation; and, II) to present the situation of IESDs and the sanitation policy of Porto Alegre.

**Methods**

The method was qualitative and quantitative, with data collection and analysis of public policies. The period analysed was from 2008 to 2012. Data were obtained from Health Information Systems, DATASUS website of the Ministry of Health, along with a set of basic indicators from the Porto Alegre’s Observatory. The indicators were classified according to the specific objectives: poverty(P); environmental sanitation(S); diseases(D). Pearson's linear correlation coefficient was used for statistical analysis to test the associations between poverty and basic sanitation indicators with IESDs indicators.

**Results**

The results showed that the biggest problems related to IESDs occur in the poorest regions, which are: *Restinga, Parthenon, Nordeste, Lomba do Pinheiro, Gloria, Ilhas and Extremo Sul*. The higher concentration of Dengue was found in the Parthenon region; Leptospirosis in the regions of *Restinga, Extremo Sul, Lomba do Pinheiro, Norte and Eixo Baltazar;* Hepatitis A in the regions of *Ilhas, Nordeste, Humaitá /Navegantes, Centro, Lomba do Pinheiro, Norte, Leste and Parthenon*. Regarding the public policy for Environmental Sanitation in Porto Alegre, we concluded that there are some urban policies, but the subject needs greater systemic view directed to the most specific problems of the city. About the Sanitation Plans, we concluded that the regions that need the sanitation, at most - a sewage collection network - have a lower footage for infrastructure installation, such as, the region of *Ilhas*. The sanitation basic plan (Water) gives various information about areas that need the implementation of infrastructures of universal supply, however there is still no date of when that will be possible.

**Conclusions**

Finally, infectious and parasitic diseases are a reality in Porto Alegre. Still at the XXI century, there are about 1,200 annual hospitalizations in health services (SUS) and are responsible for about 750 deaths per year in the capital city.

**References**

1. Carvalho EMF, Lessa F, Gonçalves FR, Silva JAM, Lima MEF, Melo Jr, SW. O processo de transição epidemiológica e iniquidade social: o caso de Pernambuco. RASPP Rev. Assoc. Saúde Pública de Piauí. 1998;1(2):107-119.

**Keywords**

Environmental sanity, Health, Hydric disease, Sanitation, Public policy.

### P130 Lateralization of the visual word form area in patients with alexia after stroke

#### Inês Rodrigues^1,2^, Nádia Canário, Alexandre Castro-Caldas^3^

##### ^1^Center for Innovative Care and Health Technology, Polytechnic Institute of Leiria, 2411-901 Leiria, Portugal; ^2^School of Health Sciences, Polytechnic Institute of Leiria, 2411-901 Leiria, Portugal; ^3^Instituto de Ciências da Saúde, Universidade Católica Portuguesa, 1649-023 Lisboa, Portugal

###### **Correspondence:** Inês Rodrigues (ines.rodrigues@ipleiria.pt)

**Background**

Knowledge of the process by which visual information is integrated into the brain reading system promotes a better understanding of writing and reading models.

**Objective**

This study aimed to use functional Magnetic Resonance Imaging (fMRI) to explore whether the Blood-oxygen-level dependent (BOLD) contrast imaging patterns, of putative cortical region of the Visual Word Form Area (VWFA), are distinct in aphasia patients with moderate and severe alexia.

**Methods**

Twelve chronic stroke patients (5 patients with severe alexia and 7 patients with moderate alexia) were included. A word categorization task was used to examine responses in the VWFA and its right homolog region. Patients performed a semantic decision task in which words were contrasted with non-verbal fonts to assess the lateralization of reading ability in the ventral occipitotemporal region.

**Results**

A fixed effects (FFX) general linear model (GLM) multi-study from the contrast of patients with moderate alexia and those with severe alexia (FDR, p = 0.05, corrected for multiples comparisons using a Threshold Estimator plugin (1000 Monte Carlo simulations), was performed. Activation of the left VWFA was robust in patients with moderate alexia. Aphasia patients with severe reading deficits also activated the right homolog VWFA.

**Conclusions**

This bilateral activation pattern only in patients with severe alexia could be interpreted as a result of reduced recruitment of the left VWFA for reading tasks due to the severe reading deficit. This study provides some new insights about reading pathways and possible neuroplasticity mechanisms in aphasia patients with alexia. Additional reports could explore the predictive value of right VWFA activation for reading recovery and aid language therapy in patients with aphasia.

**Keywords**

Stroke, Aphasia, Alexia.

### P131 Sexuality in women with oncological pathology

#### Filomena Paulo^1^, Manuela Ferreira^2^

##### ^1^Centro Hospitalar Tondela-Viseu, 3509-504 Viseu, Portugal; ^2^Instituto Politécnico de Viseu, 3504-510 Viseu, Portugal

###### **Correspondence:** Filomena Paulo (filopaulo@live.com.pt)

**Background**

It is a challenge for current nursing to care for women with oncological pathology, in what a nursing philosophy of care is concerned, and whose main focus of care are women in their multidimensionality and not the disease itself.

**Objective**

To identify some determinant factors of physical well-being, quality of life and sexuality in women with oncological disease.

**Methods**

The methodology that guided the research was an integrative review (IR) of literature. A survey was carried out in the databases: LILACS, SCIELO, Google Academic, BDENF and B-ON, between 2012 and 2016, starting from the previously defined inclusion criteria. The selected studies were subsequently evaluated. 71 articles emerged from this research study strategy. After reading all the abstracts of the articles, 27 were selected, including integrative review articles, systematic reviews of cross-sectional studies and descriptive and exploratory studies, whose contents were of interest to this review.

**Results**

After analysing the articles, it was concluded that sexual health and treatments are important aspects for the quality of life of women with oncological disease and that radical mastectomy has repercussions on body image and sexual function, since it affects self-esteem and the feeling of femininity and sexuality. Factors such as hair loss, gain or loss of weight, chronic fatigue, nausea, pain, stress, feeling of not being a complete woman, decreased arousal, lack of interest or dissatisfaction are determinants for a woman's sexual activity.

**Conclusions**

Integrative care for women with oncological pathology is a challenge for health professionals. The strategies to be adopted involve the inclusion of women's sexuality in the nursing care plan, without taboos or prejudices, because the evidence identifies the need for intervention in this field to improve, effectively, the quality of life of these women.

**Keywords**

Sexuality, Woman, Oncology, Integrative care, Quality of life.

### P132 Patient compliance to arterial hypertension treatment: integrative review

#### Diana Tavares^1^, Célia Freitas^2^, Alexandre Rodrigues^3^

##### ^1^USF Salinas – Agrupamento de Centros de Saúde do Baixo Vouga, Portugal; ^2^Center for Research in Health Technologies and Services, Aveiro University, Higher School of Health, 3810-193 Aveiro, Portugal; ^3^Center for Health Studies and Research of the University of Coimbra, Aveiro University, Higher School of Health, 3810-193 Aveiro, Portugal

###### **Correspondence:** Diana Tavares (diana.marsilia@ua.pt)

**Background**

Cardiovascular diseases are the main cause of worldwide mortality, and hypertension is an important public health problem [1]. Nonadherence to treatment influences the health of the patient and can generate several complications. In this way, health care providers are confronted with the best strategies to promote effective management of the disease, together with patients and families. The nursing consultation seems to be a privileged setting for assisting patients for healthy behaviour changes with a personalized intervention adjusted to the needs of each one [2, 3].

**Objective**

To analyse the factors that promote or inhibit adherence to treatment and to identify the nursing interventions that determine treatment adherence.

**Methods**

We conducted an integrative literature review with qualitative and quantitative studies through electronic databases Scielo, Scopus, Medline, LILACS and B-on, using the following selected research discriminators, “adherence”, “patient”, “hypertension” and “nursing care” from the DeCs and MeSH for articles published between 2011 and 2016. The PICOD [4, 5] method and the QualSyst [6] tool were used to evaluate the research question and the quality of the articles, respectively.

**Results**

In this research 372 articles were found, and 10 were selected, corresponding to 2,565 hypertensive patients over 18 years old, of whom 64% were female. Despite the differences found in the methodologies of these studies, the results indicated a poor adherence to the treatment [7]. The analysis resulted in two major categories that explain patients' adherence to the treatment of hypertension, namely the factors that influence adherence and nursing interventions. The most significant factors that inhibit adherence are complex drug regimens, lack of knowledge, and poor physical activity. The nursing interventions that enhance adherence were the adoption of continued educational strategies.

**Conclusions**

Patient adherence is a complex issue with a huge burden for the health system because it not only implies the change of patient behaviours, but also other factors like the involvement of caregivers and families. Considering the factors described in this review, the nursing consultation, through the definition of educational strategies, seems to increase patient adherence to treatment. In future research, guidelines will be suggested that aim to increase adherence to treatment.

**References**

1. Direção-Geral da Saúde. Doenças Cérebro-Cardiovasculares em Números 2015 [Internet]. 2016 [cited 2016 Mar 18]. Available from: https://www.dgs.pt/em-destaque/portugal-doencascerebro-cardiovasculares-em-numeros-201511.aspx

2. Organização Mundial da Saúde [OMS]. Adherencia a los tratamientos a largo plazo [Internet]. Genebra; 2004 [cited 2016 Feb 20]. p. 127–32. Available from: http://www.paho.org/hq/index.php?option=com_docman&task=doc_view&gid=18722&Itemid=

3. Organização Mundial da Saúde [OMS]. A global brief on HYPERTENSION [Internet]. World Health Day 2013. Geneva; 2013 [cited 2016 Feb 22]. Available from: http://www.who.int/iris/handle/10665/79059

4. The Joanna Briggs Institute. Reviewers’ Manual [Internet]. 2011th ed. Vol. 53, Adelaide: The Joanna Briggs Institute; 2011 [cited 2016 Oct 5]. Available from: http://joannabriggs.org/assets/docs/sumari/reviewersmanual-2011.pdf

5. Ramalho A. Manual para redacção de Estudos e Projectos de Revisão Sistemática com e sem metanálise – Estrutura, funções e utilização na investigação em enfermagem. Coimbra: Formasau - Formação e Saúde, Lda; 2005.

6. Kmet LM, Lee RC, Cook LS. Standard quality assessment criteria for evaluating primary research from a variety of fields. HTA Initiat [Internet]. 2004;13(February). Available from: http://gateway.nlm.nih.gov/MeetingAbstracts/103140675.html%5Cnpapers3://publication/uuid/C9499D35-AE13-428E-960B-68727C1B1833

7. Organização Mundial da Saúde. Adherence to long-term therapies: evidence for action. 2003.

**Keywords**

Patient compliance, Nursing care, Hypertension.

### P133 Didactic material as an intervention strategy in homecare for families of patients with mental disorders

#### Luísa FT Ferreira^1^, Ednéia AN Cerchiari^1^, Simara S Elias^1^, João B Almeida^2^

##### ^1^Mato Grosso do Sul State University, 79804-970 Mato Grosso do Sul, Brazil; ^2^Centro Universitário Salesiano de São Paulo, 13467-600 Americana, São Paulo, Brazil

###### **Correspondence:** Luísa FT Ferreira (luisaftf@gmail.com)

**Background**

On a daily basis, we have discussed and reflected about health education on public care as a way of interaction between health care professional and patients, with the purpose of, not only to inform, but also to exchange knowledge and experiences.

**Objective**

To elaborate and validate a Practical Guide of Care for family members of patients with mental disorders: guidelines and highlights.

**Methods**

This is a qualitative, descriptive, ongoing study with relatives of intern patients with mental disorder of a university hospital in Brazil, starting in August 2014 and finishing by October 2018.

**Results**

Five families, a total of 9 people, were interviewed through a semi-structured questionnaire. In order to create the Practical Guide, the methodological steps of the Popular Education process of Paulo Freire were used to analyse the data, as following: Thematic investigation; Thematic and Problematization. The Practical Guide of Care for Families of Patients with Mental Disorders: Guidelines and Highlights, was created in 2015 and published in 2017, containing 32 pages printed on coloured on couché paper with watercolour pictures and information arranged in short and direct texts, related to the home care of patients with mental disorders. The guide validation process began in January 2018 and is going along with professional judges that were chosen by their expertise, with one of each area: physician, psychiatrist, psychologist, nurse, social worker, pharmacist and occupational therapist. For collecting data, an instrument adapted from the Oliveira’s (2006) [1] study was used, allowing apparent and content evaluation. The instrument has affirmations about the evaluated material and was developed based on a Likert scale. The data collected will be analysed through simple statistics and organized through tables, respecting the ethical aspects proposed by Resolution 466/2012 of the National Health Council, regarding research with humans. An apparent and content evaluation with the relatives of the patients will also take place.

**Conclusions**

We expect that the final product of this study, the Validated Practical Guide, will provide support for adoption of increasingly effective strategies for the home treatment of patients with mental disorders.

**References**

1. Oliveira VLB, Landim FLP, Collares PM, Santos ZMSA. Modelo explicativo popular e profissional das mensagens de cartazes utilizados nas campanhas de saúde. Texto Contexto Enferm. 2007;16(2):287-293.

**Keywords**

Teaching materials, Public health education, Mental Disorder.

### P134 Family health in Leiria council: study of some determinants

#### Marta Serrano, Rodrigo Correia, André Branco, Luís Mousinho, Teresa Kraus^1,2^

##### ^1^School of Health Sciences, Polytechnic Institute of Leiria, 2411-901 Leiria, Portugal; ^2^Center for Innovative Care and Health Technology, Polytechnic Institute of Leiria, 2411-901 Leiria, Portugal

###### **Correspondence:** Teresa Kraus (teresa.kraus@ipleiria.pt)

**Background**

Family is the main responsible unit for personal development, and any change on a member causes alterations to the entire family [1]. The binomial family-determinants has an impact in education, socialization, in healthcare, on believes and values of the family, as well as in the well-being and health of its members.

**Objective**

To characterize the families according to its composition, family cohesion and adaptability, protective factors, health state and its determinants. Identify points of intervention on the community and family.

**Methods**

This is a quantitative, correlational and transversal study, whose data was collected in February and March 2017, in the council of Leiria, by the application of a questionnaire which evaluated sociodemographic data, family functioning, family protective factors, and the participants’ mental health, resorting to MHI5 [2], FACES IV and to the inventory of protective factors [3].

**Results**

The sample (N=224) had an average age of 40.7 years, the participants were mainly females (58.9%) residing in the countryside (69.9%). The family composition went from the nuclear type (the most common), as well as a couple, single parent, celibate, extended family and even a reconstituted family. Most of the participants consider their family function as reasonable, are satisfied with the quality of their communication and perceive family protection. However, these consider having few gratifying experiences. The majority of the sample claimed to have a good family health state, furthermore 25.0% showed signs and symptoms of severe depression and 13.4% was going through some chronical disease. As for the determinant factors, most of the respondents seemed to have a good access to health care, unlikely to transportation and social services. Around 80.0% denied daily tobacco or alcohol consumption and the religious activities were the most popular among the participants.

**Conclusions**

The results of this study allow to identify two major focus of community attention in the council of Leiria: the accessibility and regular function of social services; the early diagnosis and treatment of the family/person with depressive symptoms, with prevention of new cases; and the knowledge and integration of care related to the person living with chronical disease. Associated with the Calgary Model and with the Dynamic Model, the philosophy of care expressed in the Competency for Proactive Unconditional Care guides the discovery of meaning and promotes rewarding relationships, even during apparently negative experiences [4].

**References**

1. Hanson S. Enfermagem de Cuidados de Saúde à Família: Teoria, Prática e Investigação. 2nd edition. Loures : Lusociência; 2005.

2. Ribeiro JLP. Mental Health Inventory: Um estudo de adaptação à população portuguesa. Psicologia, Saúde & Doenças. 2001;2(1):77-99.

3. Augusto C, Ferreira O, Araújo B, Rodrigues V, Figueiredo M. Adaptation and validation of the Inventory of Family Protective Factors for the Portuguese culture. Revista latino-americana de enfermagem. 2014;22(6):1001-1008.

4. Kraus T, Dixe M, Rodrigues M. Dor, Sofrimento e Sentido de Vida: Desafio Para a Ciência, a Teologia e a Filosofia. In Lehmann O, Kroeff P, editors. Finitude e Sentido da Vida: Logoterapia no embate com a tríade trágica. São Paulo: Evangraf; 2014. p. 193-237.

**Keywords**

Family, Family health, Health determinants.

### P135 Simulation as a pedagogical strategy in nursing teaching: teachers’ perspective

#### Cláudia Chambel^1^, Catarina Carreira^1^, Catarina Pinheiro^1^, Luís Ramos^1^, Catarina Lobão^1,2^

##### ^1^School of Health Sciences, Polytechnic Institute of Leiria, 2411-901 Leiria, Portugal; ^2^Center for Innovative Care and Health Technology, Polytechnic Institute of Leiria, 2411-901 Leiria, Portugal

###### **Correspondence:** Catarina Carreira (cataricacarreira01@hotmail.com)

**Background**

Currently, classes with the resource of simulated practice have become fundamental to the nursing degree. In this way, teachers who teach classes with the resource of simulated practice feel obliged to prepare their students for clinical situations and there is a need to increase their training on new teaching strategies, such as simulation. Consequently, it becomes important for the development of students' competences, being therefore essential to deepen knowledge in this area.

**Objective**

We intend to know the perception of teachers of the nursing graduation course on the use of simulated practice as a pedagogical strategy.

**Methods**

To achieve our objective, we developed a research study using a qualitative approach and a semi-structured interview applied to seven teachers who teach classes with the resource of simulated practice in Escola Superior de Saúde de Leiria.

**Results**

From the results of our study, we verified that for the teachers, the simulation is a pedagogical strategy important in the creation of scenarios, a facilitator of the learning process that contributes to the increase of the security, confidence, satisfaction, motivation and development of technical and non-technical competences, for the formation of professional identity, management of autonomy, development of critical thinking and standardization of nursing care. Throughout the interviews they identified constraints in the use of simulation, such as: economic and material resources, realism, time available for training resourcing to simulators and the constitution of classes, since the number of students is considered excessive, and the personality of the students is also called into question. To overcome the constraints mentioned above, teachers appeal for the acquisition of material, in sufficient numbers to respond to the constitution of the classes and the desired realism. They affirm that the practical classes should have a superior workload, with unfolding of the classes, thus increasing the time given to debriefing.

**Conclusions**

Our results agree with the opinion of Gomes & Germano (2007) [1], Guhde (2011) [2] and Amendoeira, et al., (2013) [3], when they affirm throughout their studies that simulated practice allows a better articulation of the theoretical content with the practice, facilitating the learning of the contents taught in theory, via a consequent reflection of previous learning in the context of clinical practice, with the development of students' competences. In summary, the teachers interviewed highlight the importance of simulation, to answer to our research question, once they recognize the importance of simulation in the health field.

**References**

1. Amendoeira J, Godinho C, Reis A, Pinto R, Silva M, Santos J. Simulação na Educação em Enfermagem. Conceitos em Transição. Revista da UIIPS. 2013;2:212-228.

2. Gomes C, Germano R. Processo Ensino/Aprendizagem no Laboratório de Enfermagem: visão dos estudantes. Revista Gaúcha de Enfermagem. 2007;28(3):401-408.

3. Guhde JA. Nursing Students' Perceptions of the Effect on Critical Thinking, Assessment, and Learner Satisfaction in Simple. J Nurs Educ. 2011;50(2):73-78.

**Keywords**

Simulation, Nursing, Trainning.

### P136 Simulation as a pedagogical strategy in nursing teaching: students’ perspective

#### Cláudia Chambel^1^, Catarina Carreira^1^, Catarina Pinheiro^1^, Luís Ramos^1^, Catarina Lobão^1,2^

##### ^1^School of Health Sciences, Polytechnic Institute of Leiria, 2411-901 Leiria, Portugal; ^2^Center for Innovative Care and Health Technology, Polytechnic Institute of Leiria, 2411-901 Leiria, Portugal

###### **Correspondence:** Cláudia Chambel (simulacaoenfermagem@gmail.com)

**Background**

Nowadays, students increasingly recognize the importance of using simulated practice as an excellent pedagogical strategy, since it leads to experience situations very similar to reality, in an environment free of risks and penalties, which allows reflection and eventually to be repeated within useful time, thus leaving them readier to practice, in clinical situations.

**Objective**

We intend to know the perception of students of the nursing degree, on the use of simulated practice as a pedagogical strategy.

**Methods**

To achieve this, we developed a research study using a qualitative approach and a semi-structured interview applied to six students of the nursing degree of Escola Superior de Saúde de Leiria.

**Results**

From the results achieved we verify that the students indicate that the simulation is a pedagogical strategy, that facilitates the learning process and contributes to safety, confidence, satisfaction, motivation and development of technical and non-technical skills, with the recreation of scenarios closer to reality. However, they identify some constraints in the use of simulation, such as; economic resources for the acquisition of more recent and sophisticated material; realism, because the available material doesn’t allow feedback; time available for simulated practice and the constitution of classes (since they consider that the number of students is excessive and also the different personality of the students, because some students can’t take advantage of this strategy).

To overcome the constraints mentioned above, nursing students appeal for the acquisition of new and recent material, in sufficient number to overcome the heterogenic constitution of the classes and the desired realism. They affirm that practical classes should contribute more to the workload of the degree and should receive more attention from the teacher (either reducing the number of students or allowing the practical classes to be taught by two teachers simultaneously).

**Conclusions**

Our findings are in line with studies which states that high fidelity simulation facilitates the students' learning and acquisition of competencies, and results in increased motivation, satisfaction, critical thinking, and clinical decision-making. Also, Batista, Pereira and Martins (2014) [1] point out that in order for the simulated practice to reach its maximum exponent of realism, it is necessary equipment, environmental conditions similar to clinical practice and a high-fidelity simulator. In summary, the students interviewed highlight the importance of simulation in the health field.

**References**

1. Batista R, Pereira M, Martins J. Simulação no Ensino de Graduação em Enfermagem: Evidências Científicas. Série Monográfica Educação e Investigação em Saúde: A simulação no ensino de Enfermagem. 2014; pp. 65-81.

**Keywords**

Simulation, Nursing, Trainning.

### P137 Partnership between nurses and security forces to reinforce literacy in the use of child safety seats

#### Rosa Moreira^1^, Anabela Almeida^2^

##### ^1^Hospital Center Cova da Beira, 6200-251 Covilhã, Portugal; ^2^Research Unit in Business, University of Beira Interior, 6201-001 Covilhã, Portugal

###### **Correspondence:** Anabela Almeida (aalmeida@ubi.pt)

**Background**

In Portugal the low literacy in health is recognized. Literacy in health is not only related to education, it arises from a convergence of factors involving education, cultural and social factors and health services. According to data provided by the World Health Organization and World Bank, if awareness is not raised and if global behavior does not change, road traffic injuries will increase dramatically by 2020, becoming the third leading cause of death around the world [1]. Nurses assume that they can be real agents of change and have a role to play in helping to shape the behavior of parents/other educators and to train them in the correct use of child safety seats (CSS).

**Objective**

The aim of this study was to evaluate whether the partnership between nurses from the Cova da Beira Hospital Center and the regional security forces generated better results in the effective use of CSS of parents or other carers during car transport and whether there is a gap between them and parents who have never been targeted by the team of nurses and security forces.

**Methods**

A cross-sectional descriptive-correlational study with a quantitative approach, whose participants are the children and their educators from 1st cycle schools in the counties of Fundão, Covilhã and Belmonte. Sample collected by accidental or convenience method, not random. The interview and the observation occur at the same moment with the driver of the vehicle that carries the child and is the subject of stop operation.

**Results**

The stop operations had a strong pedagogical and informative component, where the drivers were clarified about the data found during the observation, thus offering a good training opportunity in an informal context. In this study, 83% of the sample was for the first time benefiting from a stop operation promoted by the PROVIDAS and it was possible to conclude that drivers who have already been subject to the supervision of PROVIDAS make fewer errors in the use of CSS in relation to drivers who had never been audited by this team.

**Conclusions**

The results suggest to the positive influence of the training and pedagogical activity of nurses and the importance of the partnership with security forces in the effective use of CSS. Drivers were found to have made more mistakes without connection to the PROVIDAS in relation to drivers had contact with PROVIDAS and security forces.

**References**

1. Fundación Gonzalo Rodríguez. Manual de Buenas Prácticas: Cómo Abordar la Seguridad de los Niños como Pasajeros de Vehículos. Uruguay : Fundación Gonzalo Rodríguez; 2010.

**Keywords**

Child safety seats, Health literacy, Partnership.

### P138 Physical activity and body image in physiotherapy students

#### Paula C Santos^1^, Rafael B Pereira^1^, Sofia MR Lopes^1^, Cristina C Mesquita^2^

##### ^1^School of Health, Polytechnic Institute of Porto, 4200-465 Porto, Portugal; ^2^Center for Research and Rehabilitation, School of Health, Polytechnic Institute of Porto, 4200-465 Porto, Portugal

###### **Correspondence:** Cristina C Mesquita (ctmesquita@ess.ipp.pt)

**Background**

There is a decrease in the physical activity of future physiotherapists, due to readjustments after admission to college. The high levels of sedentarism, cause diverse physical consequences, especially in terms of corporal image perception. This fact is due to a multidimensional construction of several psychosocial factors, which include motivational factors and behavioural changes.

**Objective**

To characterize the level of physical activity and satisfaction with body image among students of the first year of graduation in physiotherapy. Analyse the influence of initiating a graduation degree, in terms of physical activity and perception of corporal image and to also identify the main barriers to regular practice of physical activity.

**Methods**

An analytical cross-sectional study was carried out on a sample of 60 students (13 males and 47 females) from the first year of the physiotherapy degree at Escola Superior de Saúde do Porto (ESS-P), excluding those who had already attended a graduation degree. A sample characterization questionnaire and the International Questionnaire on Physical Activity (IPAQ) - Short Version were administered through the Qualtrics platform. For the anthropometric measurements and body composition, the Tanita BC-545NTM scale and a Seca stadiometer were used. Satisfaction with body image was assessed through the Body Shape Questionnaire. The questionnaire score and the Body Mass Index (BMI) were calculated. Data and its treatment was performed in the SPSS software, with a level of significance α = 0.05.

**Results**

About 20% individuals of the total sample were physically inactive and 56.7% were moderately active, there were differences in the level of activity between male and female; males were more in a “very active level” than females (61.5 vs 14.9%; p = 0.008). To start a graduation degree leads to a decrease in the regular practice of physical activity (78.3 vs 40.0%; p = 0.001 before and after, respectively). The main barriers identified for the regular practice of physical exercise were: 71.7% inadequate schedules; 30.0% laziness and 20.0% fatigue. About satisfaction with body image, only females were dissatisfied (30% vs 0%; p < 0.001). Starting a graduation degree made the perception of body image worse in 46.7% of the sample, without differences in gender.

**Conclusions**

There is a high percentage of students physically inactive and dissatisfied with body image, being this process more notorious among the female gender. Being admitted to a graduation degree has shown to influence negatively the level of physical activity and body satisfaction. Inadequate schedules are the main barrier to the practice of a physical activity.

**Acknowledgements**

We thank all the students of the first year of the degree in Physiotherapy of ESS-Porto of the 2016/2017 school year for the readiness and willingness to collaborate in the present study.

**Keywords**

Physical Activity, Students, Higher Education, Physiotherapy, Body Image.

### P139 Physical activity and stress vulnerability in physiotherapy students

#### Cristina C Mesquita^1^, Elsa S Rodrigues^2^, Sofia MR Lopes^2^, Paula CR Santos^2^

##### ^1^Center for Research and Rehabilitation, School of Health, Polytechnic Institute of Porto, 4200-465 Porto, Portugal; ^2^School of Health, Polytechnic Institute of Porto, 4200-465 Porto, Portugal

###### **Correspondence:** Paula CR Santos (pcs@ess.ipp.pt)

**Background**

International and national recommendations in Health, consider adopting an active lifestyle as fundamental. Due to the decrease in physical activity observed in students of higher education, it became fundamental to raise awareness and promote healthy behaviours. Physiotherapy students are future healthcare professionals who are experts in movement, with a primary role in health promotion. Physical activity has benefits in physical well-being, stress reduction, and academic performance.

**Objective**

Characterize the physical activity level of first year physiotherapy students and its influence on stress vulnerability, as well as to analyse the evolution of physical exercise practice in the transition to higher education.

**Methods**

Cross-sectional analytical study of 60 first year physiotherapy students from Escola Superior de Saúde, Instituto Politécnico do Porto (ESS-PP). The level of physical activity was evaluated through the International Physical Activity Questionnaires (IPAQ) and the vulnerability to stress with the Stress Vulnerability Assessment Scale (23QVS). The 23QVS is a self-assessment tool, consisting of 23 questions and allows assessing the vulnerability of an individual to stress. The higher the final score, the more vulnerable the individual is to stress, considering that the value 43 is the value above which an individual is considered vulnerable to stress. The Qualtrics software was used to fill in the questionnaires and the SPSS program for data analysis, with significance of α=0.05.

**Results**

Forty percent (40%) of the students practiced physical exercise, 18.3% were considered insufficiently active, with significant differences between genders, with males being more active (61.5 vs. 14.9, p = 0.003). (78.3% vs. 40.0%, p <0,001). It was verified that 40% of the individuals obtained a value of > 43 in the 23 QVS, showing more vulnerability to stress, being the greater proportion among the feminine gender. Nevertheless, statistically significant differences were not identified between genders (p = 0.074). Physical activity did not present a statistically significant relationship with stress vulnerability (p = 0.134; r_S_ = -0.195).

**Conclusions**

More than half of the students did not practice physical exercise and about a fifth were considered insufficiently active. The male gender had a higher level of physical activity. A large percentage of students showed excessive stress vulnerability. Starting higher education led to a decrease in the practice of physical exercise. There was no relationship between the level of physical activity and vulnerability to stress.

**Acknowledgements**

We thank all the students of the first year of the degree in Physiotherapy of ESS-Porto of the 2016/2017 school year for the readiness and willingness to collaborate in the present study.

**Keywords**

Physical Activity, Health Promotion, Stress, Academic Success.

### P140 Representations of dementia experienced in the first person: a hermeneutic analysis

#### Carlos Laranjeira^1^, Helena Quaresma^2^

##### ^1^Hospital Distrital da Figueira da Foz, 3094-001 Figueira da Foz, Portugal; ^2^Escola Superior de Enfermagem de Coimbra, 3046-051 Coimbra, Portugal

###### **Correspondence:** Carlos Laranjeira (calaranjeira@sapo.pt)

**Background**

The global incidence of dementia has been growing exponentially in recent decades. As a chronic disease, it poses as a threat to physical and social existence, amputating or redefining the roles we assume as socially integrated individuals, leading to a heavy deconstruction on the everyday world.

**Objectives**

a) Describe the representations of the person with dementia on the disease, after the diagnosis; b) understand the process of adjustment of the person with dementia, from the lived experience.

**Methods**

The methodological option was an empirical research study of the qualitative type, of phenomenological-interpretative nature and inspired by the hermeneutic philosophy of Paul Ricoeur. Seven people with mild dementia were interviewed, most diagnosed with Alzheimer's disease, with a mean of 71 years old. For each participant, two interviews were conducted in a natural setting [residence], with data collection occurring between July and October 2017.

**Results**

The main focus of the present analysis focused on the identity of the person with dementia. Two main themes were created, taking into account the factors that may influence their (de)construction. The first theme “*life in suspense*” aims to describe knowledge and representations about dementia. Regarding the analysis of the lived experience, it was represented by the second theme “*map the transition process - living on the edge of the cliff*”.

Findings from this study indicate that disease representations are useful frameworks for developing an understanding of how people with dementia try to manage the threats posed by disease as they negotiate the day-to-day process. The development of disease representations reflects an understanding that the progressive decline imposed by dementia is linked to a set of consequences that are circumscribed in the personal, relational, and transcendental dimensions.

**Conclusions**

In summary, the person with dementia faces several challenges, the first one stems from the need to manage the treatment; the second arises from the need to create and assign meaning to their social roles and finally the need to deal with the emotional consequences that arise from the process of disease by providing the person with adaptive strategies that promote their adjustment. In fact, this study, in addition to revealing the lived experience of the person with dementia, has the potential to contribute to the improvement of nursing care in mental health.

**Keywords**

Mild dementia, Lived-experience, Hermeneutic, Illness representation.

### P141 The institutionalized elderly person: representations of happiness and well-being

#### Magda Guerra, Carlos Laranjeira, Zaida Azeredo

##### School of Health, Jean Piaget Institute, Research in Education and Community Intervention, 3515-776 Viseu, Portugal

###### **Correspondence:** Magda Guerra (magdasantosguerra@gmail.com)

**Background**

Population aging in developed and developing countries is an unequivocal reality and poses multiple challenges to their communities and political entities. The societies aim at prolonging the lives of their citizens but also at an improvement of their quality of life; however, the constraints of the elderly population are diverse and it is sometimes necessary to institutionalize them. The elderly persons will have to become familiar with a set of new situations such as a new space, routines and unknown people with which they will share their life. The often-negative connotations associated with these institutions may not be appropriate to reality, because of the changes that have taken place in social policy in recent times.

**Objective**

We sought to know which representations the institutionalized elders have about their happiness and well-being.

**Methods**

The sample consisted of 13 elderly people institutionalized in a Nursing Home of Viseu, aged between 77 and 94, with 4 to 12 years of institutionalization, an option for some, due to their own volition; while for others, due to decision of another (children/nephews). It is a qualitative study, using the semi-structured interview. The results were analysed according to content analysis, with *a priori* categorization.

**Results**

Happiness for most of the elderly depends on a number of factors, such as being healthy, being well with oneself, being cherished at home, living with others, escaping from loneliness, not starving oneself, being loved and loving, possessing money for oneself and for others and fun. Most elderly people have confirmed that they feel good about themselves, yet two elderly people do not feel well because of sadness and illness. Their memories of the past relate to marriage, family constitution, strength to work and conviviality with friends; whereas, in the present, relate to happiness, a sense of general well-being, not being alone and living with other institutionalized elders.

**Keywords**

Elderly, Institutionalization, Happiness, Well-being.

### P142 Prevention of ventilator associated pneumonia- evidence in oral care

#### Ana Sousa^1,2,3^, Cândida Ferrito^4^

##### ^1^Universidade Católica Portuguesa, 4169-005 Porto, Portugal; ^2^Centro Hospitalar S. João, 4200-319 Porto, Portugal; ^3^Escola Superior de Enfermagem do Porto, 4200-072 Porto, Portugal; ^4^Escola Superior de Saúde, Instituto Politécnico de Setúbal, 2914-503 Setúbal, Portugal

###### **Correspondence:** Ana Sousa (sabrinasousa72@hotmail.com)

**Background**

Ventilator-associated pneumonia (VAP) is the most important nosocomial infection in intensive care units (ICUs), with an estimated incidence rate of 50% and the major cause of mortality and morbidity in ICUs [1,2]. Inadequate oral care develops an important role in this setting allowing various organisms to flourish in oral cavity and cause infections [1]. Many VAP prevention guidelines include oral care, but they don’t specify its demandings.

**Objective**

The aim of this study is to describe evidence-based VAP prevention oral care in ICU, in terms of products, frequency and technique.

**Methods**

Integrative review. Research was conducted on B-on, PUBMED, and RCAAP between 24 and 28 December 2015, including guidelines and original articles from the last 5 years. We found 256 documents and after analysing their abstract and methodological quality, nine documents were selected. Data were compiled in a chart in terms of grade of evidence, acceptance and applicability.

**Results**

We found inconsistent results regarding the use of an antiseptic solution in oral care, though there were meta-analysis which indicated the benefit of chlorhexidine mostly in cardio-thoracic surgical patients [2-4]. We also found evidence that tooth brushing reduces oral bacterial colonization and may reduce VAP when used with chlorhexidine [5,6]. There is no consensus regarding the adequate concentration of chlorhexidine. Some studies, thought, find an association with the use of chlorhexidine 2% and the incidence of Acute Respiratory Distress Syndrome [7]. Because of this potential risk, we do not recommend the use of this type of concentration, as more randomized controlled trials are needed. We found evidence in VAP prevention oral care comprising suctioning, tooth and gums wash and rising with 15 mL chlorhexidine 0.12%. This procedure should be performed at least 2 times a day. Secretions removal and moisturization should occur between 2 to 4 times a day [1-9].

**Conclusions**

This review allowed us to describe the adequate oral care in ICUs in order to potentially reduce VAP. As limitation of this study, we can find the lack of high grade of evidence concerning most recommendations. More randomized controlled trials are needed to support the impact of each intervention separately.

**References**

1. Munro CL, Grap MJ. Oral health and care in the intensive care unit: state of the science. Am J Crit Care. 2004;13(1):25-33.

2. Eom JS, Lee MS, Chun HK, Choi HJ, Jung SY, Kim YS, et al. The impact of a ventilator bundle on preventing ventilator-associated pneumonia: a multicenter study. Am J Infect Control. 2014;42(1):34-37.

3. Labeau SO, Van de Vyver K, Brusselaers N, Vogelaers D, Blot SI. Prevention of ventilator-associated pneumonia with oral antiseptics: a systematic review and metaanalysis. Lancet Infect Dis. 2011;11(11):845-854.

4. Shi Z, Xie H, Wang P, Zhang Q, Wu Y, Chen E, et al. Oral hygiene care for critically ill patients to prevent ventilator-associated pneumonia. Cochrane Database Syst Rev. 2013;8:Cd008367.

5. Munro, C. L., Grap, M. J., Jones, D. J., McClish, D. K., & Sessler, C. N. Chlorhexidine, toothbrushing, and preventing ventilator-associated pneumonia in critically ill adults.American Journal of Critical Care : An Official Publication, American Association of Critical-Care Nurses. 2009; 18(5), 428–438.

6. Roberts N, Moule P. Chlorhexidine and tooth-brushing as prevention strategies in reducing ventilator-associated pneumonia rates. Nursing in Critical Care. 2011;16(6):295-302.

7. Klompas M, Speck K, Howell MD, Greene LR, Berenholtz SM. Reappraisal of routine oral care with chlorhexidine gluconate for patients receiving mechanical ventilation: systematic review and meta-analysis. JAMA Intern Med. 2014;174(5):751-761.

8. Kornusky J, Schub E. Oral Hygiene: Performing for an Intubated Patient. CINAHL. 2015; Nursing Guide.

9. Pileggi C, Bianco A, Flotta D, Nobile CG, Pavia M. Prevention of ventilatorassociated pneumonia, mortality and all intensive care unit acquired infections by topically applied antimicrobial or antiseptic agents: a meta-analysis of randomized controlled trials in intensive care units. Critical Care. 2011;15(3) :R155.

**Keywords**

ICU, Oral care, Chlorhexidine, Tooth brushing, Ventilator-associated pneumonia.

### P143 Assessing preferences and features for a mobile app to promote healthy behaviors in adolescence: an exploratory study

#### Pedro Sousa^1,2^, Roberta Frontini^1^, Maria A Dixe^1,2^, Regina Ferreira^3,4^, Maria C Figueiredo^3,4^

##### ^1^Center for Innovative Care and Health Technology, Polytechnic Institute of Leiria, 2411-901 Leiria, Portugal; ^2^School of Health Sciences, Polytechnic Institute of Leiria, 2411-901 Leiria, Portugal; ^3^School of Health Sciences, Polytechnic Institute of Santarém, 2005-075 Santarém, Portugal; ^4^Indicators Monitoring Unit in Health, Polytechnic Institute of Santarém, 2005-075 Santarém, Portugal

###### **Correspondence:** Pedro Sousa (pedro.sousa@ipleiria.pt)

**Background**

A mobile application (TeenPower) to promote healthy behaviours in adolescents is being created. To better tailor the features and digital content of the mobile app, it was important to understand some of the characteristics of the devices more frequently used by the adolescents. Moreover, it was important to understand which contents are essential for health-professionals who frequently work with these adolescents. This data is extremely important during the conception and planning phase of the creation of the mobile app.

**Objective**

This study has two main aims. Firstly, to characterize and assess the devices frequently used by adolescents, as well as the preferences of adolescents for the mobile app. Secondly, to understand what features are more important for health-professionals who work closely with adolescents to promote healthy behaviours.

**Methods**

Two samples were recruited. A sample of 15 adolescents (M = 15.20; SD = 0.68) with the characteristics of the future users of the mobile app was recruited. A sample of 11 health-professionals who work closely with adolescents was also recruited. Both samples answered 2 questionnaires specifically created for the purpose. Five-point Likert scales and open questions were used. The instruments comprised questions regarding the type of devices frequently used by adolescents, the content that both, adolescents and health-professionals, consider more important regarding the promotion of healthy behaviours, and the reasons adolescents consider to use mobile apps.

**Results**

All adolescents use smartphones, but only 20% of the sample frequently use lifestyle and health apps, with the majority (93.3%) using social networks. The majority of the sample referred that food suggestions (93.3%) and physical activity suggestions (93.3%) should be included in the app. Adolescents also referred what reasons and features would influence them to use a health mobile app. Health-professionals (90.9% nurses and 9.1% psychologists) referred that the app should have food suggestions (90.9%) and physical activity suggestions (90.9%). They all referred that they would advise an adolescent to use a health-related app, with 81.8% referring that they would feel comfortable giving advices through a mobile app.

**Conclusions**

The results of our study help us tailor and choose the most important features present in the TeenPower app. Understanding what content may be more appealing for adolescents may also help the creation of future content for prevention programs.

**Acknowledgements**

The current abstract is being presented on behalf of the research group of the project TeenPower: e-Empowering teenagers to prevent obesity, co-funded by the FEDER (European Regional Development Fund), under the Portugal 2020 Program, through COMPETE 2020 (Competitiveness and Internationalization Operational Program). We acknowledge the Polytechnic Institutes of Leiria, Santarém and Castelo Branco, the Municipality of Leiria (City Hall), and also other members, institutions and students involved in the project.

**Keywords**

Adolescents, e-Health, Preferences, Prevention, TeenPower.

### P144 Epidemiological and clinical characterization of men who age with HIV/AIDS in Teresina-Piauí, Brasil

#### Keila MGS Fortes^1^, Maria LS Fortes^2^, João GO Freitas^2^, Lucas S Terto^3^

##### ^1^Municipal Health Foundation, 64002-595 Piauí, Brazil; ^2^Federal University of Piauí, 64049-550 Piauí, Brazil; ^3^University Center UniNovaFAPI, 64073-505 Piauí, Brazil

###### **Correspondence:** Lucas S Terto (terto_7@hotmail.com)

**Background**

AIDS is not a “young-only disease” as many older people still evaluate [1]. People with a more advanced age range are starting to appear in the settings of those living with HIV/AIDS [2]. Many causes are attributed: sociocultural changes, especially in sexuality; resistance in using condoms; healthcare innovations; access to antiretroviral therapy and other innovations in clinical areas [3]. Among men aged 60 years and over, in the last decade, there has been an increase in the rate of detection [4], which raises the need for public policies aimed at this reality.

**Objective**

To investigate epidemiological and clinical characteristics of men housed in a shelter for people living with HIV/AIDS.

**Methods**

Descriptive study of epidemiological and clinical information on people living with HIV/AIDS, housed in a shelter in Teresina-Piauí, Brazil. Data were collected from 28 selected records, with the following criteria: male, aged 50 years or older. The following variables were considered: age group, educational level, place of residence, time of antiretroviral therapy and clinical manifestations. The data were typed in an Excel 2007 spreadsheet, analysed through percentage differences and discussed, with reference to documents and also articles in which there was content about the proposed theme.

**Results**

Regarding the age of the group, ages were between 50 and 64 years old, with predominance of individuals aged from 50 to 60 years old. The majority of the elderly presented a low educational level (79.75%), coming from cities with less than 50,000 inhabitants of other states of Brazil (53.57%), who seek Teresina for treatment and follow-up of their health, shelter to receive food, nursing, social and spiritual assistance and, in some cases, family reintegration. Most used antiretroviral drugs since 10 to 20 years ago, with 7.15% using it for more than 30 years. The clinical manifestations detected were: Lung Cancer, Hepatitis B and C, depression, dermatitis, tuberculosis, neuropathy and leishmaniasis.

**Conclusions**

This study draws attention to the increase in the detection of HIV in men aged 50 years and over, especially those aged 60 years or older, inhabitants of cities of low demographic density in Brazil, with several clinical manifestations, low educational level, which makes difficult the access to preventive information on sexual health, and under the use of antiretrovirals, from more than ten years ago. The study points towards the need for more research on HIV infection among the elderly, to help to implement more effective public policies for this group.

**References**

1 Gorinchteyn J. Sexo e AIDS Depois dos 50.1st edition. São Paulo: Ícone; 2010.

2 Okuno MFP, Gomes AC, Meazzini L, Júnior GS, Junior DB, Belasco AGS. Qualidade de Vida de Pacientes Idosos Vivendo Com HIV/AIDS. Cad. Saúde Pública, Rio de Janeiro. 2014;30(7):1551-1559.

3 Serra A, Sardinha, AHL, Pereira ANS, Lima SCVS. Percepção de Vida dos Idosos Portadores do HIV/AIDS atendidos em Centro de Referência Estadual. Saúde em Debate, Rio de Janeiro. 2013;37(97):294-304.

4 Ministério da Saúde. Secretaria de Vigilância em Saúde. Boletim Epidemiológico - AIDS e DST. Ano V, Nº 1. 2015-2016.

**Keywords**

Old men, AIDS, Health care.

### P145 Assessing digital contents for health promotion and obesity prevention in adolescence

#### Rita Luz^1^, Pedro Sousa^1,2^, Roberta Frontini^2^, Andreia Silva^1^, Briana Manual^1^, Cláudia Ramos^1^, Mónica Ruivo^1^, Rúben Abreu^1^, Tiago Pozo^1^, Ana E Sardo^1^, Francisco Rodrigues^1^, Luís Fernandes^1^

##### ^1^School of Health Sciences, Polytechnic Institute of Leiria, 2411-901 Leiria, Portugal; ^2^Center for Innovative Care and Health Technology, Polytechnic Institute of Leiria, 2411-901 Leiria, Portugal

###### **Correspondence:** Rita Luz (ritafluz@gmail.com)

**Background**

The TeenPower project is an e-Health multidisciplinary program to promote healthy behaviours and prevent obesity in adolescents. In this study we focus in two of the components present in the platform: stress management and interpersonal relationships. It is important to focus on stress while designing prevention programs for adolescents, given that literature has associated higher levels of stress with obesity in youth. Moreover, interpersonal relationships were also found to influence obesity-related behaviours. Online sessions were specifically created to address those issues on the mobile app. This digital content (videos and posters) should not only be appealing for the adolescent but also scientifically valid and correct.

**Objective**

The main aim of this study was to assess the scientific quality and adequacy of posters and videos created for the TeenPower mobile app, regarding stress management and interpersonal relationships.

**Methods**

Digital resources in video and poster format were created. Contents regarded stress management and interpersonal relationships. Videos were 2 minutes long and posters included infographics and written content with adequate language for adolescents. These resources were developed by students of health, sports and design, in collaboration with health-professionals and researchers. The sample included adolescents with the sociodemographic characteristics of the future users of the mobile app, and health-professionals working in this field. A questionnaire was developed and validated for the purpose, based on the questionnaire create by Junior and colleagues.

**Results**

Results regarding the concept idea, information, construction of scenes and characters, dialogues, visual and audio style, and quality and relevancy of the information were obtained. Moreover, adolescents answered regarding the attractiveness and adequacy of the content, and health-professionals answered regarding the scientific accuracy of the content information. Both samples suggested content improvements.

**Conclusions**

The digital content of the mobile app regarding stress management and interpersonal relationships is adequate and appealing for both adolescents and health-professionals. The assessment of digital content is crucial to understand the acceptability of the contents for future users. Digital contents and online sessions are extremely important, given that adolescents use new technologies on a daily basis. Furthermore, it is important to note that digital contents may have the potential to enhance the adherence to programs, promoting healthy behaviours and preventing obesity.

**Acknowledgements**

The current abstract is being presented on behalf of the research group of the project TeenPower: e-Empowering teenagers to prevent obesity, co-funded by the FEDER (European Regional Development Fund), under the Portugal 2020 Program, through COMPETE 2020 (Competitiveness and Internationalization Operational Program). We acknowledge the Polytechnic Institutes of Leiria, Santarém and Castelo Branco, the Municipality of Leiria (City Hall), and also other members, institutions and students involved in the project.

**Keywords**

Adolescents, e-Health, Obesity, Health promotion, Digital contents.

### P146 Assessing digital content in the TeenPower project: development and validation of a questionnaire

#### Roberta Frontini^1^, Pedro Sousa^1,2^, Rita Luz^2^, Ana Duarte^2^, Beatriz Sismeiro^2^, Maria Moreira^2^, Romeu Machado^2^

##### ^1^Center for Innovative Care and Health Technology, Polytechnic Institute of Leiria, 2411-901 Leiria, Portugal; ^2^School of Health Sciences, Polytechnic Institute of Leiria, 2411-901 Leiria, Portugal

###### **Correspondence:** Roberta Frontini (roberta_frontini@hotmail.com)

**Background**

The TeenPower project aims to develop a program to promote healthy behaviours and prevent obesity in adolescents. It is a multidisciplinary project with an important e-Health component. Therefore, including valid digital content may help to maximize and optimize the impact of the program. Given that over the years digital resources had a great evolution, there is a growing concern regarding the acceptance of the digital content by the target public. Thus, to assess the digital resources of the TeenPower project, there was the need to develop and validate a questionnaire that could accurately assess the quality and adequacy of the digital content of the TeenPower mobile app.

**Objective**

Develop and validate a questionnaire to assess the quality and adequacy of the videos and posters of the TeenPower.

**Methods**

Two scales were developed based on the questionnaire created by Junior and colleagues [1]: one for adolescents (12-16 years old) and one for health-professionals. The questionnaire for adolescents comprised 18 items answered on a 5-point Likert scale and 2 open questions (to assess the video content); as well as 11 items answered on a 5-point Likert scale and 2 open questions (to assess the poster content). The questionnaire for health-professionals comprised 17 items answered on a 5-point Likert and 2 open questions (to assess the video content); as well as 11 items answered on a 5-point Likert scale and 2 open questions (to assess the poster content). The sample included adolescents with the sociodemographic characteristics of the future users of the mobile app, and specialized health-professionals. Exploratory factor analyses and analysis of internal consistency through Cronbach's alpha were performed.

**Results**

Data regarding the concept idea, the construction of scenes and characters, the dialogues, visual and audio style and the quality and relevance of the information was obtained. Data regarding the acceptance and comprehension of the content and digital form of the app was obtained from adolescents. The quality and rigorousness of the scientific information was validated by health-professionals.

**Conclusions**

The questionnaire presented good psychometric qualities with adequate values for internal consistency and factorial analysis. Given that nowadays there is a vast offer of digital content related to health, there is a concern to use not only appealing content for future users, but also valid and scientifically correct information. This questionnaire may be an important tool to understand the acceptability and quality of the scientific content of the videos and posters.

**Acknowledgements**

The current abstract is being presented on behalf of the research group of the project TeenPower: e-Empowering teenagers to prevent obesity, co-funded by the FEDER (European Regional Development Fund), under the Portugal 2020 Program, through COMPETE 2020 (Competitiveness and Internationalization Operational Program). We acknowledge the Polytechnic Institutes of Leiria, Santarém and Castelo Branco, the Municipality of Leiria (City Hall), and also other members, institutions and students involved in the project.

**Keywords**

Adolescents, e-Health, Validation, Questionnaire, Digital content.

### P147 Implementation process of “Engaging Clients Who Use Substances” guideline in a nursing school curriculum

#### Olga Valentim^1^, Maria José Nogueira^1^, Luís Sousa^1,2^, Vanessa Antunes^1^, Sandy Severino^1,3^, António Ferreira^4^, Luís Gens^4^, Luís Godinho^5^

##### ^1^School of Health Sciences, Atlântica University, 2730-036 Barcarena, Portugal; ^2^Hospital Center Lisbon Central, Curry Cabral Hospital, 1050-099 Lisbon, Portugal; ^3^Health Center Groupings Loures-Odivelas, Regional Health Administration Lisboa e Vale do Tejo, 2685-101 Sacavém, Portugal; ^4^Hospitaller Order of São João de Deus, Telhal Health House, 1600-871 Lisboa, Portugal; ^5^Psychiatry Department, Garcia da Orta Hospital, 2805-267 Almada, Portugal

###### **Correspondence:** Olga Valentim (ommvalentim@gmail.com)

**Background**

Nursing research has led to knowledge which has contributed to improving health care and to reduce costs. The implantation of guidelines ensures the transfer of the best evidence for clinical practice [1]. Substance-related problems can occur at any age, but usually begin in adolescence [2]. The Guideline Engaging Clients Who Use Substances developed by the Registered Nurses' Association of Ontario (RNAO), provides evidence-based recommendations related to the assessment and interventions for people over 11 years of age, who use substances, may be at risk of, or have a substance use disorder [3].

**Objective**

To present the experience of the guideline implementation process of the RNAO’s Engaging Clients Who Use Substances, in the *curriculum* of the nursing degree (CLE) of the Atlantic Health School (ESSATLA).

**Methods**

Implementation procedures indorsed by the RNAO were followed, involving teachers, students and nurses from several clinical practice contexts. First, an analysis and reflection were made considering ESSATLA's CLE *curriculum*, Unit Sheets and the Engaging Clients Who Use Substances guideline recommendations. Afterwards, a guideline implementation plan was designed to fit the CLE, based on structure, process and outcome indicators. Teachers and clinical tutor train was performed and some guideline topics were included in several units: establishing therapeutic relationships [4] and person- and family-centred care [5].

**Results**

To date, guideline implementation process results include several outcomes: seminar meetings held with all stakeholders involved in the guideline’s implementation process; a partnership training project - Partnership training seminars; a workshop scheduling plan; Portuguese translation of the “Engaging Clients Who Use Substances” in process (teacher and nursing expert stakeholder collaboration); didactic materials to support content implementation in the nursing *curriculum*; student evaluation tools and instruments; three students included the topic of substance use in their end-of-course monograph project; some students in the clinical practice of the elderly did a in-service training session on this subject.

**Conclusions**

The implementation of this guideline in the CLE curriculum has empowered students to become more confident and competent to care for substance abusers, namely regarding screening, the assessment process and intervention in substance use disorders. It also meets the expectations of the stakeholders involved, empowering their performance based on scientific evidence.

**References**

1. Silva AG. Implementación de guías de buenas prácticas clínicas elaboradas por Registered Nurses Association of Ontario (RNAO) en el curriculum de Enfermería Universidad de Chile. MedUNAB. 2015;17(3):182-9.

2. Serviço de Intervenção nos Comportamentos Aditivos e nas Dependências (SICAD). Relatório Anual 2015: A Situação do País em Matéria de Drogas e Toxicodependências. Lisboa: SICAD. 2016.

3. Registered Nurses’ Association of Ontario (RNAO). Engaging Clients Who Use Substances. Toronto, ON: Registered Nurses’ Association of Ontario. 2015.

4. Registered Nurses’ Association of Ontario (RNAO). Establishing Therapeutic Relationships. Toronto, ON: Registered Nurses’ Association of Ontario. 2002.

5. Registered Nurses’ Association of Ontario (RNAO). Person and family centred care Toronto, ON: Registered Nurses’ Association of Ontario. 2015.

**Keywords**

Substance-Related Disorders, Evidence-Based Nursing, Nursing Education.

### P148 An overview of vitamin B in food supplements

#### Isabel M Costa, Alexandra Figueiredo, Deolinda Auxtero

##### Instituto Universitário Egas Moniz, 2829-511 Caparica, Portugal

###### **Correspondence:** Isabel M Costa (imargaridac@gmail.com)

**Background**

Over last decade, sales of vitamins have had a significant increase worldwide. Besides the growing of self-diagnosis and self-medication by consumers, these products are also often consumed without any control or medical supervision, during extended periods of time, due to the general misperception that natural indicates harmless. Despite its beneficial effects, excess intake of vitamin is not innocuous. Although vitamin B6 is a co-factor for several enzymatic reactions, involved in numerous metabolic and physiological processes, overdoses may produce neurological disturbances, including sensory neuropathy.

**Objective**

The aim of this study was to check food supplements (FS) labels in terms of vitamin B (vitB) dosages, compared to the recommended daily allowances (RDA) defined by European Union Directive for these vitamins. A total of 80 FS sold in Portuguese pharmacies, supermarkets or health shops and on the internet were examined for indicated daily intake and dosage of vitamin B1, B2, B3, B5, B6, B7 and B12. Selection criteria included: oral solid pharmaceutical forms for adults, containing vitB in its composition, as stated in the label, regardless of the purpose of the FS.

**Results**

Results showed FS label doses above RDA: 70.0% (vitB1), 75.0% (vitB2), 67.4% (vitB3), 51.1%, (vitB5), 74.3% (vit B6), 45.7% (vitB7) and 60.3% (vit B12). Thirty-three (33) FS contained all the studied VitB, six of which with all vitamins above RDA. Four (4) FS (5.7%) indicated a daily dose of vitB6 ≥ the tolerable upper intake level defined by EFSA (UL=25 mg/day).

**Conclusions**

The majority of FS presented vitB far above defined RDA. Although reports of toxic events due to vitamins are scarce, it is crucial that the daily doses present in FS are reviewed ensuring for the safety of these products. Authors also consider that FS should be under the same quality control of pharmaceuticals, safeguarding the health of the consumers.

**Keywords**

Vitamin B, Food Supplements, Recommended Daily Allowances.

### P149 Is the International Physical Activity Questionnaire (IPAQ-sf) valid to assess physical activity in patients with COPD? Comparison with accelerometer data

#### Joana Cruz^1,2,3^, Cristina Jácome^3,4^, Alda Marques^3,5^

##### ^1^Center for Innovative Care and Health Technology, Polytechnic Institute of Leiria, 2411-901 Leiria, Portugal; ^2^School of Health Sciences, Polytechnic Institute of Leiria, 2411-901 Leiria, Portugal; ^3^Respiratory Research and Rehabilitation Laboratory, School of Health Sciences, University of Aveiro, 3810-193 Aveiro, Portugal; ^4^Center for Health Technologies and Information Systems Research, Faculty of Medicine, University of Porto, 4200-319 Porto, Portugal; ^5^Institute of Biomedicine, University of Aveiro, 3810-193 Aveiro, Portugal

###### **Correspondence:** Joana Cruz (joana.cruz@ipleiria.pt)

**Background**

The International Physical Activity Questionnaire short form (IPAQ-sf) is primarily designed for physical activity (PA) surveillance, presenting good psychometric properties in people with an age range of 15-69 years. However, studies conducted in older people have shown conflicting results, suggesting that it may not be adequate for this population. Therefore, the use of the IPAQ-sf for the assessment of PA in patients with chronic conditions such as chronic obstructive pulmonary disease (COPD), in which patients are frequently older, remains unclear.

**Objective**

To preliminary evaluate the validity and test-retest reliability of the IPAQ-sf in patients with COPD.

**Methods**

This exploratory cross-sectional study included 10 patients with COPD (71.6 ± 7.3 years old, 7 males, FEV1=77.2 ± 20.7% predicted). Participants completed the IPAQ-sf on two occasions separated by 1 week and wore an accelerometer (Actigraph GT3X+) for 7 consecutive days. The following statistical analyses were conducted: 1) Pearson’s correlation coefficient (r) to assess correlations between the results obtained from the IPAQ-sf (PA in METs-min/week; sitting time in min/day) and the accelerometer (PA: total moderate-to-vigorous physical activity [MVPA] per week and recommended MVPA per week – i.e., MVPA conducted in bouts of at least 10-min as internationally recommended [1]; sedentary time in min/day); 2) percentage of agreement (%agreement) and Cohen’s kappa to assess the agreement between categorical scores obtained from the two measures (*i.e.*, ‘sufficiently’ and ‘insufficiently’ active patients); 3) Intraclass Correlation Coefficient (ICC2,1) and 95% limits of agreement (LoA) to assess test-retest reliability and agreement.

**Results**

Significant correlations were found between IPAQ-sf METs-min/week and total MVPA (r=0.729, p=0.017), but not between METs-min/week and recommended MVPA (r=0.346, p=0.327) or between IPAQ-sf sitting time and accelerometer-based sedentary time (r=-0.383, p=0.308). Agreement between the IPAQ-sf and accelerometer-based data, in identifying ‘sufficiently’ and ‘insufficiently’ active patients, was low (total MVPA: kappa=-0.538, %agreement=20%; recommended MVPA: kappa=-0.087, %agreement=50%). Test-retest reliability of the IPAQ-sf was poor to moderate (PA: ICC2,1=0.439 [-0.267→0.838]; sedentary time: ICC2,1=0.511 [-0.178→0.864]) and the agreement was low (PA: LoA: -10361→4548 METs-min/week; sedentary time: LoA: -194→148 min/day).

**Conclusions**

Findings suggest that the IPAQ-sf has limited validity and reliability in the assessment of PA in patients with COPD. Further research with a larger sample is needed to support these findings.

**References**

1. Garber CE, Blissmer B, Deschenes MR, Franklin BA, Lamonte MJ, Lee IM, etal. Med Sci Sports Exerc. 2011;43(7):1334-1359.

**Keywords**

Accelerometry, Chronic obstructive pulmonary disease, Psychometric properties, Physical activity, Self-report measure.

### P150 Concurrent validity of the Portuguese version of the brief physical activity assessment tool

#### Joana Cruz^1,2,3,^ Cristina Jácome^3,4^, Nuno Morais^1,2,5^, Ana Oliveira^3,6^, Alda Marques^3,6^

##### ^1^Center for Innovative Care and Health Technology, Polytechnic Institute of Leiria, 2411-901 Leiria, Portugal; ^2^School of Health Sciences, Polytechnic Institute of Leiria, 2411-901 Leiria, Portugal; ^3^Respiratory Research and Rehabilitation Laboratory, School of Health Sciences, University of Aveiro, 3810-193 Aveiro, Portugal; ^4^Center for Health Technologies and Information Systems Research, Faculty of Medicine, University of Porto, 4200-319 Porto, Portugal; ^5^Centre for Rapid and Sustainable Product Development, Polytechnic Institute of Leiria, 2411-901 Leiria, Portugal; ^6^Institute of Biomedicine, University of Aveiro, 3810-193 Aveiro, Portugal

###### **Correspondence:** Joana Cruz (joana.cruz@ipleiria.pt)

**Background**

Physical activity (PA) is recognised as an important health enhancing behaviour and should be routinely assessed in clinical practice to identify insufficiently active people. Activity monitors, such as accelerometers, provide objective assessment of free-living PA being the preferred assessment method in research settings. However, they are too expensive to be used in resource-constrained clinical settings. Several PA questionnaires have already been validated to the European Portuguese but some of them take too long to complete, hence unfeasible for use in clinical practice. Shorter PA assessment tools are, therefore, needed.

**Objective**

To explore the relationship between the Portuguese version of a short PA questionnaire, the Brief physical activity assessment tool (Brief-PA tool), and the International Physical Activity Questionnaire short form (IPAQ-sf), which is a valid and reliable PA assessment tool already tested in the Portuguese population. A secondary aim was to explore the test-retest reliability of the Brief-PA tool.

**Methods**

The Brief-PA tool [1] consists of 2 questions which assess the frequency and duration of moderate and vigorous PA undertaken in a ‘usual’ week. The total score is obtained by summing the results of the two questions (range 0-8). People with a score ≥ 4 are considered ‘sufficiently active’. Since the tool is not available in Portuguese, a linguistic adaptation was conducted using the forward- and back-translation method. Then, 86 healthy volunteers (49.5±18.1 years, age range 20-69; 53 female) completed the Brief-PA tool and the IPAQ-sf. A sub-sample (n=56, 43.1±18.1 years, 37 female) completed the Brief-PA tool one week later. Spearman’s rank correlation coefficient (ρ) was used to assess correlations between the Brief-PA total score with IPAQ-sf results (MET-min/week). Percentage of agreement (%agreement) and Cohen’s kappa were used to assess the agreement between categorical scores obtained from the two measures (i.e., ‘sufficiently’ and ‘insufficiently’ active) and test-retest reliability of the Brief-PA tool.

**Results**

Significant correlations were found between the Brief-PA tool and the IPAQ-sf (ρ = 0.721, p < 0.001). The Brief-PA tool identified 34.8% sufficiently active participants while the IPAQ-sf identified 59.3%. Agreement between measures was moderate (%agreement=70.9%, kappa=0.450). Test-retest reliability of the Brief-PA tool was substantial (%agreement=89.3%, kappa=0.755).

**Conclusions**

The Brief-PA tool seems to be valid and reliable for assessing PA in the Portuguese adult population, although the agreement with the IPAQ-sf was only moderate. Further research assessing the validity of the Brief-PA tool with objective measures is needed.

**References**

1. Marshall AL, Smith BJ, Bauman AE, Kaur S. Reliability and validity of a brief physical activity assessment for use by family doctors. Br J Sports Med. 2005;39(5):294-297.

**Keywords**

Concurrent validity, Daily living, Physical activity, Self-report measure.

### P151 Effect of an exercise program on risk of fall in a community dwelling older adults

#### Sara Martins, Anabela Martins, Carla Guapo, Sílvia Vaz

##### Physiotherapy Department, Coimbra Health School, Polytechnic Institute of Coimbra, 3046-854 Coimbra, Portugal

###### **Correspondence:** Sara Martins (ftsaramartins@gmail.com)

**Background**

Falls are a problem among the elderly population. It is known that currently about 30% of people over 65 years fall every year. The European Union estimates a cost of € 281 per inhabitant per year and a cost of € 25 billion per year in health care [1] which translates into a significant economic impact. The World Health Organization (WHO) [2] argues that it is possible to reduce these costs through prevention and health promotion strategies. For this, it is important to raise awareness, evaluate risk factors and identify and implement intervention programs.

**Objective**

To test the effect of an exercise program on the prevention of risk of fall.

**Methods**

This study, which lasted 4 months, was experimental, prospective. The experimental group (EG) performed an exercise program and the control group (CG) maintained their usual routine. For the measurement and evaluation of the variables under study, were used: a sociodemographic data questionnaire, the self-efficacy for exercise scale, the Portuguese version of the falls efficacy scale (FES), the 10m walking speed (WS), the Timed Up & Go test (TUG), step test and the Hercules® Force Platform (static balance). A significance level of 5% (p ≤ 0.05) was considered for all comparisons.

**Results**

After intervention, there were differences in walking speed (p < 0.001), FES (p < 0.001), static balance (p < 0.001), and self-efficacy for exercise (p = 0.004), with EG scoring higher than CG.

**Conclusions**

This exercise program integrated activities of daily living (ADL), muscle strengthening, balance and flexibility exercises, complemented by walking, showed improvements in static balance and walking speed. There was also a change in the behaviour regarding confidence in the performance of the ADL and perception of ability to learn and integrate exercise in daily life, thus contributing to decrease the risk of fall.

**Acknowledgements**

To the Penacova Health Center - ARS CENTRO for opening their facility to the program implementation and for the collaboration provided by all professionals.

**References**

1 Active ageing through preventing falls: “Falls prevention is everyone’s business”. European Stakeholders Alliance for Active Ageing through Falls Prevention. Prevention of Falls Network for Dissemination (ProFouND).; 2015.

2 Global recommendations on physical activity for health. Switzerland.: World Health Organization (WHO); 2010.

**Keywords**

Prevention, Active Ageing, Fall, Fall Risk, Exercise Programs.

### P152 Nutritional impact of food waste in a school cafeteria

#### Ana Braga^1^, Ana Pedrosa^1^, Fabiana Estrada^1^, Matilde Silva^1^, Ana Sousa^1^, Cidália Pereira^1,2^, João Lima^1,3^, Vânia Ribeiro^1,2^

##### ^1^School of Health Sciences, Polytechnic Institute of Leiria, 2411-901 Leiria, Portugal; ^2^Center for Innovative Care and Health Technology, Polytechnic Institute of Leiria, 2411-901 Leiria, Portugal; ^3^Coimbra Health School, Polytechnic Institute of Coimbra, 3046-854 Coimbra, Portugal

###### **Correspondence:** Ana Braga (anabraga423@gmail.com)

**Background**

In Portugal, it is estimated that about 31% of food waste occurs in the last stage of the food production chain and that about 13% of the food served is not consumed [1]. Food waste contributes to an inadequate nutritional intake, since part of the served food is being discarded, therefore its evaluation is important [2].

**Objective**

To evaluate the nutritional impact of food waste in relation to nutritional requirements in a food unit of the Social Services of the Polytechnic of Leiria.

**Methods**

Food waste was determined by the aggregate component weighing method, being evaluated at lunch and dinner periods of a randomly chosen day. The data were collected in the cafeteria and snack bar that served the largest number of meals. Only non-fractioned dishes were considered in this analysis. The nutritional impact of food waste was evaluated considering the nutritional requirements of a typical costumer of the food services - an average energy value of 2000 kcal and the percentages corresponding to the two evaluation moments of the day, corresponding to 55% of the Total Energy Value (TEV), 30% to lunch and 25% to dinner [3, 4].

**Results**

It was produced 288.9 kg of food, being 40.3 kg of plate waste. The average amount of food served per meal was 329 g and an average of 50 g per meal was plate waste. It can be concluded that about 14% of the total food produced was discarded as plate waste, which represents around 123 meals. When evaluating the nutritional impact of food waste, it was observed that the average waste per person was 84 kcal, which represents about 15.3% of the energy value of meals. It was also observed that 2.4 g of lipids, 5.6 g of protein and 9.6 g of carbohydrates were wasted, per meal, which is the equivalent to about 13.1%, 20.4% and 14.0%, respectively, of the needs of the typical costumer of the food services.

**Conclusions**

Based on these results, we can observe a food waste impact of about 10% of the nutritional requirements of an individual, being imperative the reinforcement of periodic evaluation of consumption and plate waste in school meals, attending that food waste is a decisive factor in terms of energy and nutritional adequacy [5]. On the other hand, it is still necessary to develop actions to raise the awareness among the student community about the nutritional impact of food waste on the health of the population.

**References**

1. Baptista P, Campos I, Pires I, Vaz S. Do Campo ao Garfo. 2012.

2. Figueira J. Influência da satisfação com as refeições escolares no desperdício alimentar, em crianças do 4º ano de escolaridade. 2012.

3. Afonso C, Santos MCT, Morais C, Franchini B, Chilro R, Rocha A. Sistema de planeamento e avaliação de refeições escolares - SPARE. Rev Aliment Humana. 2011;17(1–3):37–46.

4. Jorge IN de SDR. Tabela da Composição de Alimentos. 2007.

5. Bergman EA, Buergel NS, Englund TF, Femrite A. The Relationship of Meal and Recess Schedules to Plate Waste in Elementary Schools. J Child Nutr Manag. 2004;28(2).

**Keywords**

College, Dining, Lunch, School Meals, Rests.

### P153 Food insecurity and obesity paradox: nutritional intervention strategies

#### Carla C Correia^1^, Ana L Baltazar^2^, José Camolas^1^, Manuel Bicho^1^

##### ^1^Faculty of Medicine, University of Lisbon, 1649-028 Lisbon, Portugal; ^2^Coimbra Health School, Polytechnic Institute of Coimbra, 3046-854 Coimbra, Portugal

###### **Correspondence:** Carla C Correia (carlacamposcorreia@gmail.com)

**Background**

The economic crisis in the recent years has triggered social disparities in Europe, which are shown in people’s food insecurity levels and public health. Food insecurity occurs when the consumer’s physical, social and economic access to adequate and nutritional food are scarce or non-existent. Food insecurity is associated to chronic diseases, such as obesity, type 2 diabetes, dyslipidaemia, hypertension, and a poor health status, due to unbalanced food habits and sedentary lifestyles. In this low socioeconomic position, people need social and nutritional intervention to improve their habits and their health, in general.

**Objective**

Analyse and discuss the existing strategies to further intervene at the paradox “food insecurity *versus* obesity”.

**Methods**

A scientific narrative of the state of art was performed according to PRISMA standards and in snowball, inserting scientific articles, official documents and books applied to the European population, from 2007-2017.

**Results**

The access of each person to a health and nutritive diet should be a right guaranteed by any country. The strategies to deal with the impact of food insecurity in health status should be multidisciplinary, addressing economic, psychologic, social and physiological issues, together with the health, social, education, agriculture and economic sectors. The prices are an important determinant for people’s choices. Food marketing control and agriculture and local markets supports are strategies to facilitate the access to healthy food. It’s important to implement monitoring programs in primary health care and schools to develop nutrition and physical activity projects at a local level, to alert the professionals to food insecurity issues and the relation with obesity, and to intervene timely in pregnancy and family planning appointments, as means to prevent diseases related to food insecurity.

**Conclusions**

Chronic diseases bring us high costs to health systems and some questions about their sustainability. An adequate and timely intervention should consider food education and health lifestyles promotion, so that the integration of nutritionists into food assistance programs is emergent.

**Keywords**

Food insecurity, Chronic diseases, Social disparities, Nutritional intervention.

### P154 Numerical methodology to support a medical device development

#### Filipa Carneiro^1^, Lourenço Bastos^1^, Ângelo Marques^1^, Rita Marques^1^, Jordana Gonçalves^1^, Andreia Vilela^1^, André Maia^2^, Sara Cortez^2^, Anabela Salgueiro-Oliveira^3^, Pedro Parreira^3^, Bruno Silva^1^

##### ^1^Innovation in Polymer Engineering, Universidty of Minho, 4800-058 Guimarães, Portugal; ^2^Muroplás – Indústria de Plásticos, S.A., 4745-334 Trofa, Portugal; ^3^Health Sciences Research Unit: Nursing, Nursing School of Coimbra, 3046-851 Coimbra, Portugal

###### **Correspondence:** Filipa Carneiro (f.carneiro@piep.pt)

**Background**

The use of numerical simulations as part of product development process allows the existence of virtual prototypes that can be tested quickly and cheaply. These computational tools facilitate and improve the design optimization and the materials selection, to match the pre-defined product requirements, during the development process, for use in health contexts [1].

**Objective**

Development and validation of a numerical methodology to support an innovative syringe, predicting its mechanical and flow behaviour, during the syringe loading and patient administration; as also the injection molding process of its constituent components. This methodology aims to optimize the geometries of the syringe’s components, creating in this way an iterative process for product development based on numerical simulations.

**Methods**

This numerical methodology was implemented using software for fluid dynamics, mechanical behaviour and injection process, respectively. To study the fluid-structure interaction (FSI), during syringe loading and patient administration of medicines and washing solutions, the fluid dynamics output served as structural simulations input. Material properties were experimentally determined. The FSI numerical models were validated by comparison with experimental tests on a single chamber syringe. Afterwards, the same numerical models were implemented in new innovative syringe concepts. The injection molding processes of these concepts were also numerically evaluated.

**Results**

Validation of the numerical simulations using a simple case of a single chamber syringe, where the numerical solution is compared with an experimental case. Development and application of an iterative process for product development, based on numerical simulations. Optimization of an innovative product design that fulfils all the specifications and requirements predefined.

**Conclusions**

This iterative process based on numerical simulations is a powerful tool for product development that allows obtaining fast and accurate results, without the strict need of prototypes. An iterative process can be implemented, consisting on consecutive constructions and evaluations of new concepts, to obtain an optimized solution, which fulfils all the predefined specifications and requirements. The prior validation of the numerical methodology with a reference model, is an extremely important step to guarantee the reliability of the numerical model applicability in the development of medical devices.

**Acknowledgments**

Work funded by the FEDER fund, Operational Programme for Competitiveness and Internationalisation (COMPETE 2020), project POCI-01-0247-FEDER-017604.

**References**

1. Oliveira RF, Teixeira SFCF, Silva LF; Teixeira JCF, Antunes H. Development of new spacer device geometry: a CFD study (Part I), Computer Methods in Biomechanics and Biomedical Engineering. 2012;15(8):825-833.

**Keywords**

Numerical simulation, Fluid-structure interaction, Injection process, Syringe.

### P155 The impact in burden of care provided by informal caregivers of patients with mental illness

#### Catarina Tomás^1,2^, Ana Querido^1,2^, Marina Cordeiro^1,2^, Daniel Carvalho^3^, João Gomes^3^

##### ^1^School of Health Sciences, Polytechnic Institute of Leiria, 2411-901 Leiria, Portugal; ^2^Center for Innovative Care and Health Technology, Polytechnic Institute of Leiria, 2411-901 Leiria, Portugal; ^3^Santo André Hospital, Hospital Center of Leiria, 2410-197 Leiria, Portugal

###### **Correspondence:** Catarina Tomás (catarina.tomas@ipleiria.pt)

**Background**

Psychiatric disease is one of the most incapacitating conditions, creating the need for continuous care, generating burden in family members [1, 2]. Recent research has revealed some tasks developed by family caregivers like preparing meals, help in daily living activities and in-house maintenance which contributes to enhance burden.

**Objective**

To understand the correlation between provided care and burden in informal caregivers of patients with mental illness and to analyse the impact of the care provided in caregivers’ burden.

**Methods**

This is a cross-sectional correlational study. Data was collected in a sample of 113 caregivers, in 2015, using a face-to-face interview which comprised sociodemographic questions, the type of care provided and the Zarit Burden Interview (Portuguese version by Sequeira [3]). Ethical procedures were taken into account during research according to the Helsinki Declaration.

**Results**

Sample was mostly composed by females (70.8%), aged between 20 and 81 years old (Mean=49.85; SD=14.25), married (74.3%), wives (15.9%) and mothers (17.7%) of the patients. Depressive disorders (36.3%) were the most common mental problems of their sick relatives. Most caregivers categorized themselves has primary (39.8%) and secondary caregivers (42.5%) providing total care most frequently (Mean=26.58; SD=31.20). The majority of these caregivers presents no burden (62.8%), scoring the mean 38.31 (SD=22.54). Total burden is correlated with the provision of total care (R=0.340; p=0.000) and support (R=0.216; p=0.022). This kind of care provision is also correlated with impact of giving care (R=0.355; p=0.000), interpersonal relations (R=0.360; p=0.000) and perceptions of self-efficacy (R=0.275; p=0.003). Burden is 11.5% explained by the total care provided (F=14.330; p=0.000) and 6% by support provided (F=11,542; p=0,000). Additionally, 25.3% of burden is explained by care in dressing and shoeing (F=37.288; p=0.000), 5% by preparing the meals (F=23.724; p=0.000) and 4.8% by support to accomplish patient professional demands. All factors of burden were influenced by total care provided (p<0.005).

**Conclusions**

A medium range of burden was found in this sample of caregivers of patients with mental illness inquired. By considering themselves has secondary caregivers they provide regularly or occasionally care depending on their relative’s needs. Nevertheless, they provide total care frequently. There was a positive and significant correlation found between burden and care provided, with an impact of this care in the caregiver’s burden. Intervention in caregivers of patients with mental illness should address this relation, providing support and developing their skills to improve the care provided and preventing caregivers’ burnout.

**References**

1. Albuquerque E, Cintra A, Bandeira M. Sobrecarga de familiares de pacientes psiquiátricos: comparação entre diferentes tipos de cuidadores. J Bras Psiquiatr. 2010;59(4):308-316.

2. Eloia S, Oliveira E, Eloia S, Lomeo R, Parente J. Sobrecarga do cuidador familiar de pessoas com transtorno mental: uma revisão integrativa. Saúde Debate. 2014;38(103):996-1007.

3. Sequeira C. Adaptação e validação da Escala de Sobrecarga do Cuidador de Zarit. Revista Referência. 2010;2(12):9-16.

**Keywords**

Family caregivers, Care provided, Mental disorders, Burden.

### P156 Levels of physical activity and sedentary behavior in school-time of elementary school children

#### Mariana Lima^1^, Ana Soares^1^, Andreia Santos^1^, Fernando Martins^1,2^, Rui Mendes^1,3^

##### ^1^Coimbra Education School, Polytechnic Institute of Coimbra, 3030-329 Coimbra, Portugal; ^2^Telecommunications institute, University Beira Interior, 6200-161 Covilhã, Portugal; ^3^Center for Research on Sport and Physical Activity, University of Coimbra, 3040-248 Coimbra, Portugal

###### **Correspondence:** Mariana Lima (asms.mecl@gmail.com)

**Background**

Zimmo et al. (2017) [1], concluded that in what concerns the physical activity (PA) of children in elementary schools (ES), only 39% of the children reached the recommended values of moderate (M) and vigorous (V) PA (30 or more daily minutes), and showed that children spend most of their school time involved in sedentary activities (SB).

**Objective**

The aims of this study were I) the description of PA (light, moderate and vigorous levels) and sedentary behaviours (SB) of ES boys and girls in relation with the time spent in each level of PA; II) to determine the average time children spend on each level of PA during 4 weekdays (9:00 a.m. to 5:30 p.m., Monday to Thursday) and, III) to compare the MVPA developed by children during formal physical education sessions, with other weekdays.

**Methods**

Forty (40) voluntarily children with 7.9 ± 0.6 years (30 girls and 10 boys) were authorized by their parents to participate in the study. PA was assessed using a three-axial accelerometer (ActiGraph® wGT3X-BT) during to 7.5 daily hours of school period. For classifying moderate-to-vigorous physical activity (MVPA) a cut-off point of 818 counts per 5s was used.

**Results**

The average duration of MVPA was 36.51 ± 13.50 min per day. Only 35% of the participating children reached the recommended school-based MVPA of 30 min or more, per day. Children spent on average 71.74 ± 6.37% of their school time on SB. MVPA of girls were lower (31.9 ± 8.9 min/day) than boys (51.3 ± 16.1 min/day, ES = 1.76, p=0.004). Our results showed that the percentage of MVPA on the day of the physical education lesson (11.4 ± 3.8%) was higher, when compared to other weekdays (7.5 ± 2.6%).

**Conclusions**

We objectively assessed PA during school hours among elementary school-children. This study found that children spend the majority of their school time in SB and many of them do not perform sufficient time of being physically active at school. The low participation of girls in MVPA and the lesser time spend in MVPA on weekdays without physical education lessons are relevant data to reflect and implement strategies to increase the time of physical activity during school-time, which corresponds to one third of the daily life of children.

**Acknowledgements**

Research partially supported by QREN, Mais Centro - Programa Operacional Regional do Centro, FEDER (CENTRO-07- CT62-FEDER- 005012; ID: 64765).

**References**

1. Zimmo L, Farooq A, Almudahka F, Ibrahim I, Al-Kuwari M. School-time physical activity among Arab elementary school children in Qatar. BMC Pediatrics. 2017;17(1):76.

**Keywords**

Physical activity, Motor development, School-time, Accelerometer, Physical education.

### P157 Labor pain relief: sterile water injection vs finger ischemic compression technique on the lumbosacral region

#### Ana Moulaz (anamoulaz@gmail.com)

##### Polytechnic Institute of Bragança, 5300-253 Bragança, Portugal

**Background**

Nowadays there is an overvaluation about the pain of giving birth. Therefore, Brazil presented 56% of caesareans and Portugal 33.1% in 2016. In order to assist women when giving birth, analgesia has been a strong ally to enable delivery without pain. There are non-pharmacological techniques for pain control in obstetrics. There is, the Sterile Water Injection on the Michaelis Triangle which causes immediate pain relief in the lumbosacral region. Physiologically, sterile water doesn’t act as a local anaesthetic and doesn’t inhibit the fibres that report visceral pain; however, causes a release of the C fibres and A-delta fibres associated with somatic pain. Distilled water stimulates A-delta fibres and subjugates the visceral pain reported by the C fibres, which fails to notify visceral pain, modulating afferent patterns of pain. In this way, it silences the C fibres and releases endorphins. Based on this mechanism, an experimental study has been done applying the finger ischemic compression technique to the same region, in Brazil in 2014, to characterize the technique as a tool to pain control.

**Objective**

To compare the technique of sterile water injection with the experiment of finger ischemic compression in the lumbosacral.

**Methods**

A Comparative study between those techniques through a systematic review of literature based on the Clinical Practice Guide on Normal Childbirth Care, by the Basque Government, about sterile water injection and the finger ischemic compression technique (Nursing Residency Program in Obstetrics, in Brazil in 2015). This study compares the effect versus time duration of analgesia between them.

**Results**

The analysis of 292 studies showed that the sterile water injection into the Michaelis triangle decreased lumbar pain during delivery by approximately 60%, and the effect remained for up to 2 hours. In the finger ischemic compression experiment, there was a pain level reduction by 66%, remaining for 4 hours. However, in both cases, patients referred an intense burning during application.

**Conclusions**

This study contributes to pain control in obstetrics, as the two methods lead to a significant reduction of the pain level. However, finger ischemic compression had a longer duration of analgesia, when compared to sterile water injection. The injection in obstetrics is already recommended as an effective method for pain control and there is a need for further research on the finger ischemic compression experiment, to also consolidate the technique as a method of pain management.

**Keywords**

Labor pain relief, Obstetrics, Giving birth, Non-pharmacological techniques, Analgesia.

### P158 Microencapsulation of phytosterols and/or other bioactive ingredients for minimizing cardiovascular risk

#### Pedro Vieira^1^, Isabel Andrade^2^, Rui Cruz^1^

##### ^1^Pharmacy Department, Coimbra Health School, Polytechnic Institute of Coimbra, 3046-854 Coimbra, Portugal; ^2^Complementary Sciences Department, Coimbra Health School, Polytechnic Institute of Coimbra, 3046-854 Coimbra, Portugal

###### **Correspondence:** Pedro Vieira (pedromdvieira96@gmail.com)

**Background**

Several bioactive ingredients such as phytosterols, resveratrol, curcumin and catechin have shown efficacy in the prevention of chronic diseases, namely in cardiovascular diseases, one of the leading causes of death in the world. Besides all the potentialities, many of these bioactive ingredients present the disadvantage of instability and sensitivity to environmental factors (e.g. light and oxygen) and are also characterized by low water solubility due to its lipophilicity. Microencapsulation of bioactive compounds emerges as a strategy to improve their stability, avoiding adverse conditions and promoting their bioavailability. It includes a set of several techniques that allows the coating of these ingredients through a protective film, to guarantee its protection and to modulate its release at the target cells. Many techniques of microencapsulation and different materials can be used, the choice depends on the intended application, particle size, the release mechanism and on the physicochemical properties of both active material to be encapsulated and encapsulating agent.

**Objective**

To review the available research in this field of expertise.

**Methods**

A research was conducted using Google Scholar, PubMed and Elsevier databases, in Portuguese, English or Spanish languages over the last 10 years, and using as keywords Microencapsulation; bioactive compounds; phytosterols; cardiovascular risk.

**Results**

The search retrieved 10 studies. The findings demonstrated not only the potential of the compounds in the reduction of cardiovascular risk, through the action on risk factors, but also the increase of their activity due to the increase of their bioavailability, achieved through microencapsulation. The research also revealed a wide range of materials that can be used, while the choice of method must take into account several factors such as its cost and necessary equipment, as well as the quality of the microcapsules formed. The microencapsulation process has a wide range of advantages, namely the ability to increase gastrointestinal absorption of the bioactive compounds encapsulated, and in addition, while the conversion of those compounds into the powder form is enhanced, their stability increases and their handling is facilitated.

**Conclusions**

The use of microencapsulation avoids some undesirable characteristics, proving to be an alternative to increase the bioavailability of the bioactive compounds. Future studies should address the exploration of newer encapsulating methods and materials.

**Keywords**

Microencapsulation, Bioactive compounds, Phytosterols, cardiovascular risk.

### P159 A weighted decision making approach for a new medical device concept selection

#### Marta Gomes^1^, Ângelo Marques^1^, Ricardo Freitas^1^, Anabela Salgueiro-Oliveira^2^, Pedro Parreira^2^, Alberta Coelho^3^, Sara Cortez^3^, Bruno Silva^1^, Filipa Carneiro^1^

##### ^1^Innovation in Polymer Engineering, Universidty of Minho, 4800-058 Guimarães, Portugal; ^2^Health Sciences Research Unit: Nursing, Nursing School of Coimbra, 3046-851 Coimbra, Portugal; ^3^Muroplás – Indústria de Plásticos, S.A., 4745-334 Trofa, Portugal

###### **Correspondence:** Filipa Carneiro (f.carneiro@piep.pt)

**Background**

The development of an innovative syringe results, at an early stage, in several preliminary concepts that should be compared and analysed to select the most promising one. Making the selection decision is a very important task that should be structured and well-founded. Decision making methods can be applied to help in the selection process, leading to more informed and better decisions.

**Objective**

To apply a weighted decision matrix, in order to select the most promising concept for an innovative syringe.

**Methods**

To select the most suitable concept for the device to be developed, it is mandatory to define the specifications that it must obey, considering the functional requirements of the product and the requirements for the production process. Not all the specifications have the same importance in concept selection, and so this was analysed trough a weighted decision matrix. Based on the identified specifications, it was defined the corresponding criteria, which must be evaluated and weighted regarding their relative importance to each of the other criteria. These weights were summed for each criterion. As experts from different areas were involved, it was decided that each expert group should weigh, considering their experience. As expected, different matrixes were obtained and results were managed to achieve only one weight matrix which included the opinion of all the groups. A final weighted matrix with all the criteria and its weight for concept selection was obtained. Each concept of the syringe was evaluated according to these defined criteria and calculated it weighted sum. It was selected the concept with the highest weighted sum of all the defined criteria.

**Results**

By analysing the proposed weighting table of the specifications, and considering the defined relative weight, it was verified that the most relevant specifications are the cost, the number of operations to be performed and the possibility of error. Based on this, the concept with better weighted performance was selected for further detailed development.

**Conclusions**

A weighted decision matrix has showed to be a very effective tool to assist the development of new products for use in health, particularly in cases where there are many concepts and many criteria of varying importance to be considered.

**Acknowledgments**

Work funded by the FEDER fund, Operational Programme for Competitiveness and Internationalisation (COMPETE 2020), project POCI-01-0247-FEDER-017604.

**Keywords**

Weighted decision matrix, Medical device, Product design.

### P160 Vestibular symptoms in sensorineural hearing loss

#### Maria Araújo^1^, Luís Rama^2^

##### ^1^Audiology Department, Coimbra Health School, Polytechnic Institute of Coimbra, 3046-854 Coimbra, Portugal; ^2^Research Center for Sport and Physical Activity, Faculty of Sport Sciences and Physical Education, University of Coimbra, 3040-256 Coimbra, Portugal

###### **Correspondence:** Maria Araújo (ines@estescoimbra.pt)

**Background**

The type of aetiology of hearing loss is directly related with the embryonic and physiological interactions of the anatomical structures of the auditive and vestibular systems. The proximity between both system structures can evolve simultaneously audition and balance, mainly in individuals with peripheral pathologies.

**Objective**

To characterize and evaluate the vestibular symptoms in individuals with bilateral severe and profound hearing loss.

**Methods**

The information was gathered with resource to two questionnaires: one for anamnesis and the Dizziness Handicap Inventory (DHI). The sample was constituted by 28 adults with severe and profound sensorineural hearing loss, between 19 and 64 years old (15 females and 13 males). 42.9 % of the sample had idiopathic aetiology, 10.7 % had meningitis and the same percentage with the aetiology of measles.

Results

Seventy-five percent 75% had tinnitus, being 33% bilateral. Regarding vertigo symptoms, 53.6% reported at least one episode, describing it as a rotatory vertigo (66.7%), with the length of minutes (33.3%) to days (26.7%). Regarding DHI, the functional sub scale is the one that perceives more difficulties when performing daily tasks, as a consequence of vertigo and/or unbalance (5.7 ± 8.5), followed by the physical sub scale (4.8 ± 6.8).

**Conclusions**

The study has shown that the major part of the sample had already faced symptoms as tinnitus, vertigo and/or unbalance.

**Keywords**

Hearing Loss, Vestibular symptoms, Tinnitus, Vertigo.

### P161 Men’s prenatal experience in the transition to fatherhood

#### Catarina Silva^1^, Cristina Martins^2^, Cândida Pinto^3^

##### ^1^Agrupamento de Centros de Saúde do Alto Ave, 4810-503 Guimarães, Portugal; ^2^Nursing School, University of Minho, 4700-057 Braga, Portugal; ^3^Nursing School of Porto, 4200-072 Porto, Portugal

###### **Correspondence:** Catarina Silva (catsilva@gmail.com)

**Background**

Pregnancy is a demanding period in terms of psychological reorganization in the transition to fatherhood [1,2]. The involvement of men in this period is associated with their own psychological well-being as well as the whole household [3]. This is a transition with implications for the couple, for the father/child relationship, and child development [4]. Contemporary fatherhood emphasizes the involvement of men and greater affective contact with their children, in addition to their traditional role as a financial provider [5].

**Objective**

This study sought to understand the experiences of men as they transition to fatherhood during the prenatal period, aiming to contribute to a complete assistance to the family and improving the health gains of the family.

**Methods**

Qualitative research paradigm. Exploratory, descriptive, cross-sectional and retrospective study, with the participation of 10 men experiencing, for the first time, the pregnancy of their partners, in the last trimester, in a common-law marriage and with gestation without maternal-foetal pathology. Data collection was performed using the semi-structured interview. The data was analysed with the content analysis technique, with semantic categorization and an inductive approach.

**Results**

Through content analysis three themes emerged, “experiencing transition”, “development of father identity” and “(de)construct bridges to transition.” The results revealed a male experience of pregnancy characterized by an enormous psychic and emotional depth. Men accept and try to actively engage in the pregnancy process and experience a panoply of positive and negative feelings and emotions, sometimes ambivalent. The prenatal period triggers the development of paternal identity. During this process, expectant fathers revaluate their personal values, reflect on their own fathering experiences and reshape their view of the world and themselves. Despite their proactive role in the fatherhood journey, they find obstacles and not bridges to their transition. The fact that they feel peripheral in prenatal care services makes it more difficult to embrace fatherhood and may compromise overcoming the transition process.

**Conclusions**

This study provided insight on the complex nature of this developmental transition and showed fragile environments in the approach of the expectant fathers to the prenatal care services. This reality encourages healthcare professionals to think critically about how fatherhood transition can be facilitated by practices which promote smoother transitions and benefit the family as a whole.

**References**

1. Condon J, Boyce P, Corkindale C. The First-Time Fathers Study: a prospective study of the mental health and wellbeing of men during the transition to parenthood. Aust N Z J Psychiatry. 2004;38(1-2):56-64.

2. Genesoni L, Tallandini M. Men's Psychological Transition to Fatherhood: An Analysis of the Literature, 1989-2008. Birth. 2009;36(4):305-318.

3. Plantin L, Olukoya A, Ny P. Positive Health Outcomes of Fathers' Involvment in Pregnancy and Childbirth Paternal Support: A Scope Study Literature Review. Fathering: A Journal of Theory, Research, and Practice about Men as Fathers. 2011;9(1):87-102.

4. Bawadi H, Qandil A, Al-Hamdan Z, Mahallawi H. The role of fathers during pregnancy: A qualitative exploration of Arabic fathers’ beliefs. Midwifery. 2016;32:75-80.

5. McGill B. Navigating New Norms of Involved Fatherhood. J Fam Issues. 2014;35(8):1089-1106.

**Keywords**

Fathers, Parenting, Pregnancy, Qualitative research.

### P162 Diffuse large b-cell lymphoma, which treatment options are available?

#### Mariana Carvalho^1^, Fernando Mendes^2,3,4^, Salomé Pires^2,4^, Ricardo Santo^2,7^, Nicole Eicher^8^, Ricardo Teixo^2,4^, Rui Cruz^1^

##### ^1^Pharmacy Department, Coimbra Health School, Polytechnic Institute of Coimbra, 3046-854 Coimbra, Portugal; ^2^Biophysics Unit, Faculty of Medicine of University of Coimbra, 3004-504 Coimbra, Portugal; ^3^Department Biomedical Laboratory Sciences, Coimbra Health School, Polytechnic Institute of Coimbra, Coimbra Health School, 3046-854 Coimbra, Portugal; ^4^Center of Investigation in Environment- Genetics and Oncobiology, Faculty of Medicine of University of Coimbra, 3004-504 Coimbra, Portugal; ^5^Immunology Institute, Faculty of Medicine, University of Coimbra, 3004-504 Coimbra, Portugal; ^6^Applied Molecular Biology and Clinical University of Hematology, Faculty of Medicine of University of Coimbra, 3004-504 Coimbra, Portugal; ^7^Faculty of Sciences and Technologies, University of Coimbra, 3004-504 Coimbra, Portugal; ^8^Biomedical Laboratory Sciences Department, University of Applied Sciences, 6711 Gratz, Austria

###### **Correspondence:** Fernando Mendes (fjmendes@estescoimbra.pt)

**Background**

Diffuse large B-cell lymphoma (DLBCL) is one of the most common and aggressive subtypes of non-Hodgkin's lymphoma (NHL). The current therapy for DLBCL is the combination of rituximab, cyclophosphamide, doxorubicin, vincristine and prednisone (R-CHOP). R-CHOP-14 and R-CHOP-21 are two subtypes of R-CHOP, which differ in their treatment, like CHOP-14 that has better results within high dosage in advanced stages, while CHOP-21 has better efficiency and better overall survival in elderly patients. These treatments have increased the survival of patients, though about 40% of patients there is still a failure rate of treatment. New approaches for relapsed DLBCL are outlined here, for example the understanding of the new drugs role, individualized treatments and dosage regimes.

**Objective**

To review the treatments available for DLBCL and all the developments of novel treatments, particularly in DLBCL recurrence variants of treatment with R-CHOP and in patients over 80 years.

**Methods**

A research was conducted using Google Scholar and PubMed databases, in English languages over the last 5 years, and using as keywords: Diffuse large B-cell lymphoma (DLCBL) treatment; rituximab, cyclophosphamide, doxorubicin hydrochloride, vincristine sulphate, prednisone (R-CHOP); cyclophosphamide, doxorubicin hydrochloride, vincristine sulphate, prednisone (CHOP).

**Results**

The treatment of this pathology has advanced over time starting with CHOP and later incorporating rituximab with lower relapse and upturn survival rates. Currently, about 80% to 90% of patients in early stage of DLBCL remain free of the disease after treatment with R-CHOP and radiotherapy consolidation. Concerning elderly patients (> 80 years), the addition of rituximab, namely, a reduced dose R-CHOP provides a good compromise between toxicity and the efficiency of overall survival. The survival of patients with DLBCL with more than 66 years has improved substantially since the introduction of rituximab. However, finding the optimal dose for older patients without associated toxicity is an important focus for further research.

**Conclusions**

The DLBCL is a heterogeneous disease, both clinically and biologically. Although, DLBCL therapy results have improved significantly over the past decades with the introduction of new specific therapeutic antitumor strategies.

**Keywords**

Diffuse large B-cell lymphoma, Rituximab, Cyclophosphamide, Doxorubicin, Vincristine, Novel treatments.

### P163 Mistreatment to elderly in family context

#### Maria FP Ribeiro (fatima.ribeiro@ipsn.cespu.pt)

##### School of Health of Vale do Sousa, North Polytechnic Institute of Health, 4760-409 Vila Nova de Famalicão, Portugal

**Background**

The evidence about the increasing number of elderly people who, in their everyday lives, are abused and the implications that these events have on the practice of health professionals, plus the need for new «powers» bear alone the importance of the subject of this study. This issue of great sensitivity imposes a multifactorial attention to the social, educational, clinical and ethical domains. This involves the concerted action by all health professionals whose mission passes through contacting with seniors.

**Objective**

To identify signs of elder abuse and typology of abuse in a family context.

**Methods**

Exploratory study and empirical work, constituted by a sample of four hundred (400) elderly, enrolled in a ACES in the north of the country, in order to identify signs of abuse. In this study, a questionnaire was used to collect information about indicators of abuse in the elderly [1]. For processing and analysis, we’ve recurred to technics of descriptive and inferential statistics using factor analysis.

**Results**

The analysis made allowed us to sort indicators of abuse, by type of abuse, in the following order: neglection (64.8%), emotional/psychological (26.6%), economic (21.6%) and physical abuse (7.1%).

**Conclusions**

The results of this study suggest the emergence and necessity of early screening for signs and/or symptoms indicative of risk factors that may lead to the installation of maltreatment. The role of health professionals proves to be of prime importance and helps fighting this phenomenon.

**References**

1. Carney MT, Kahan FS, Paris Barbara EC. Questions to Elicit Elder Abuse (QEEA), 2003. Translated and adapted into Portuguese by Alves, JF; Sousa M. 2005.

**Keywords**

Abuse, Elderly, Mistreatment, Prevention.

### P164 Guidelines and training a role to play for learning health organizations? The HAIs example

#### Sandra Oliveira^1,2^, Sofia Ferreirinha^3^, Carla Cordiro^3^, António Lopes^4^

##### ^1^Polytechnic Institute of Santarém, 2001-902 Santarém, Portugal; ^2^Center for Health Studies and Research, University of Coimbra, 3004-504 Coimbra, Portugal; ^3^District Hospital of Santarém, 2005-177 Santarém, Portugal; ^4^Hospital Center of the Medio Tejo, 2304-909 Tomar, Portugal

###### **Correspondence:** Sandra Oliveira (sandra.oliveira@esg.ipsantarem.pt)

**Background**

International projections estimate that, in 2050, 390,000 people will die annually in Europe, as a direct consequence of Healthcare Associated Infections (HAIs) [1]. In Portugal, we estimate that 5 in 100 patients could have acquired HAIs during hospitalization [2]. Research based on practice guidelines was published, and despite evidence that good practice strategies are sufficient to reduce the rate of HAIs, hospitals struggle to comply [3]. Investigation of organizational solutions that may contribute to reduce the rate of HAIs is much needed.

**Objective**

This exploratory study has the following objectives: (1) to identify if the legal standards are known by health professionals of acute services of Portuguese hospitals, detect training actions or courses as well as the most common subjects taught in formal training actions; (2) to recognize the strategies implemented in the hospitals; (3) to analyse whether there are differences between knowledge and implementation of practices in hospitals; (4) to study the suggestions made by the health professionals.

**Methods**

Through a quantitative and qualitative approach, using quantitative data analysis and content analysis procedures, this study explores the different perspectives given by the health professional groups. The design of the study involved a literature review, study of the legal standards and conduction of informal interviews to field experts, with the aim of producing a list of topics to assess the level of implementation of the legal norms. A focus group method was used to encourage participants to exchange experiences and perspectives. These interactions allowed the collection of more detailed information and an in-depth exploration of the opinions of the participants [4][5]. The focus group also served as a pre-test to the questionnaire. A convenience sample of four acute services of Portuguese Hospitals was selected. The questionnaire was distributed in the hospitals after receiving the accordance of the Administration Board and Ethical Commission of the institutions involved. Participation in the study was voluntary and ranged all the health professionals of the medical and surgery services.

**Results**

The results suggest that, although aware of the legal norms, when we control for differences between groups, we find differences between health professional groups. Health professionals recognize and value the existence of training, mainly under the responsibility of the Health Institutions, but do not consider it effective.

**Conclusions**

This research highlights the importance of spreading knowledge and training in healthcare organizations, notably through the identification of the need for new approaches of training as well as for new training areas.

**References**

1. Direção Geral de Saúde 2016.

2. OPSS Acesso aos cuidados de saúde. Um direito em risco?. Relatório de Primavera Observatório Português dos Sistemas de Saúde (OPSS). Lisboa; 2015.

3. Zingg W, Holmes A, Dettenkofer M, Goetting T, Sicca F, Clack L, et al. Hospital organization, management, and structure for prevention of health-care-associated infection: a systematic review and expert consensus. Lancet Infect Dis. 2015;15(2):212-224.

4. Kitzinger J. Introduction focus groups in qualitative research. In: Mays N, Pope C., editors. Health care. London: Blackwell; 1996. p. 36–45.

5. Morgan DL. Focus groups as qualitative research. 2nd Edition. Outstand Oaks, CA: Sage; 1994.

**Keywords**

Guide lines, HAIs, Training and Health Organizations.

### P165 Barriers, obstacle, difficulties or challenges in development of health partnerships in community intervention projects: a systematic review

#### Odete Alves^1^, Paula C Santos^2,3^, Lídia Fernandes^4^, Paulo Moreira^5,6^

##### ^1^Abel Salazar Institute of Biomedical Sciences (ICBAS), University of Porto, 4050-313 Porto, Portugal; ^2^Physical Therapy Department, Health School, Polytechnic Institute of Porto, 4200-465 Porto, Portugal; ^3^Research Center in Physical Activity, Health and Leisure, Faculty of Sport, University of Porto, 4200-450 Porto, Portugal; ^4^School of Health, Polytechnic Institute of Viana do Castelo, 4900-314 Viana do Castelo, Portugal; ^5^Center of Administration and Public Policies of the University of Lisbon, 1300-663 Lisbon, Portugal; ^6^University Atlântica, 2730-036 Oeiras, Portugal

###### **Correspondence:** Odete Alves (odetemaalves@hotmail.com)

**Background**

Engaging communities in authentic partnerships is increasingly accepted as best practice in community intervention projects, despite the many barriers or challenges to doing so.

**Objective**

The purpose of this study is to identify barriers, obstacles, difficulties or challenges in development of health partnerships in community intervention projects of some countries.

**Methods**

We conducted a systematic review using the following data sources: PubMed, B-on, Medline and EBSCOhost. We searched articles from September 2006 through January 2016. A standard form was used to extract data using the key words in the search: Health Partnerships AND Community Health AND Primary Health Care. The articles were selected according to inclusion and exclusion criteria. In the end, we grouped these results based on the six categories used by the Wilder Research Centre: Environment; Membership; Process and structure; Purpose; Communication; Resources, which includes leadership and power.

**Results**

From the research conducted, 844 articles emerged, which were submitted to the filter, which implied references to at least one of the keywords: Barriers OR Obstacle OR Difficulties OR Challenges. According to the inclusion criteria, a total of fifty-six articles was found. Of these articles, forty-four dealt with factors relating to Environment, which included factors related to community, geography, culture, religious faith and homophily, and politics. Regarding the characteristics of the members that influence the development of the partnership, the relationship between the partners is key and this was commented in fifty-three articles. Factors relating to the process of collaboration were found in a total of forty-six articles, while factors related to structural elements were mentioned in forty articles. Thirty-one articles identified the factors relating to objectives, vision and mission. Another factor that was highly discussed was communication, appearing in a total of thirty-four articles. Factors relating to resources were given great importance in the literature, appearing in forty-eight articles. In twenty-three articles, the subject of leadership and factors relating to power were mentioned. The literature reviewed highlighted that factors such as relationships, commitment, communication, funding and structure, are key in the long-term sustainability of the partnership. This topic appeared in fifteen articles.

**Conclusions**

The systematic literature review identified a set of barriers, obstacles, difficulties or challenges for the development of health partnerships in community intervention projects. In each of the categories we present the factors that are related to them and that can positively or negatively influence the development of those collaborations.

**Acknowledgements**

We are very grateful to Dr. Alcino Maciel Barbosa for his insightful comments on an earlier draft of this project and to Caroline Esteves for her contributions in this paper.

**Keywords**

Barriers, Obstacle, Difficulties, Challenges, Partnerships.

### P166 Parental perception of child body image: retrospective analysis of two studies

#### Graça Aparício^1,2^, Patrícia Nascimento^3^, Madalena Cunha^1,2^, João Duarte^1,2^

##### ^1^Escola Superior de Saúde de Viseu, Instituto Politécnico de Viseu, 3500-843 Viseu, Portugal; ^2^Centro de Estudos em Educação, Tecnologias e Saúde, 3504-510 Viseu, Portugal; ^3^Centro de Saúde de Seia, 6270-468 Seia, Portugal

###### **Correspondence:** Graça Aparício (gaparicio5@hotmail.com)

**Background**

Childhood obesity is a major problem in Portugal and studies show that parents are concerned about their children's overweight, but their perception of children's nutritional status is not always adequate or is even distorted.

**Objective**

The general objective was to explore the parental perception of their children's body image in two studies, study A [1] and study B [2].

**Methods**

Cross-sectional and retrospective study using two samples, from study A (792) and study B (1424) totalizing 2,216 pre-school children, with mean age = 4.51 years old (SD= 0.97), living in the region of Viseu and Dão (study A) and Viseu, Lamego, Vila Real, Évora and Leiria (study B). The original authors performed the children's anthropometric evaluation and the nutritional classification based on NCHS reference (CDC, 2000). A Sociodemographic Characterization Questionnaire for Children and Parents was used and the Parental Perception of Children's Body Image Assessment [3].

**Results**

In Study A, overweight was 31.3% (including 12.4% obesity) and 2.7% underweight. In study B was 34.3% overweight (17.4% obesity) and 5.5% underweight, with significant differences (Chi-square= 21.355; p= 0.000). In study B, parents were significantly more concerned about their children nutritional status (UMW = 498564.000, p = 0.000) and a higher percentage of parents pointed the representative pre-obesity images (27.5%) and obesity (0.6%), compared to study A, where more children in the normal and low-weight group (56.3% and 20.4% respectively) were selected. A significant difference of means from the parental perception of the child's body image was found between studies (UMW = 528960.500; p = 0.037), showing a closer perception to the higher values of BMI, *i.e.*, parents presented a less distorted perception of their children's body image, when they have higher BMI values.

**Conclusions**

The results indicate more accuracy of the parental perception of the children body image and an oncoming to their real nutritional status in the latest study. This may be the first step towards their recognition of their children's overweight that is critical to prompting family action, and consequently preventing and treating childhood obesity.

**References**

1. Aparício G, Cunha M, Duarte J, Pereira A. Olhar dos pais sobre o estado nutricional das crianças pré-escolares. Millenium 2011;(40):99-115.

2. Aparício G, Cunha M, Duarte J, Pereira A, Bonito J, Albuquerque C. Nutritional status in preschool children: Current trends and concerns. Atención Primaria 2013;(45)(Espec cong 1):94-200.

**Keywords**

Parents, Weight perception, Body image, Pediatric obesity.

### P167 Informal caregivers of mental health patients: burden and care provided

#### Daniel Carvalho^1^, Catarina Tomás^2,3^, Ana Querido^2,3^, Marina Cordeiro^2,3^, João Gomes^1^

##### ^1^Santo André Hospital, Hospital Center of Leiria, 2410-197 Leiria, Portugal; ^2^School of Health Sciences, Polytechnic Institute of Leiria, 2411-901 Leiria, Portugal; ^3^Center for Innovative Care and Health Technology, Polytechnic Institute of Leiria, 2411-901 Leiria, Portugal

###### **Correspondence:** Daniel Carvalho (drscarvalho@gmail.com)

**Background**

Families and informal caregivers play an important role in providing care for mental health patients [1]. Family caregivers of these patients usually are overloaded with caring activities [2], being more vulnerable to psychological disturbance and burden due to the care provided [3].

**Objective**

To characterize the care provided; access the burden experienced by informal caregivers of mental health patients and identify its determinants.

**Methods**

Cross-sectional correlational study, with a non-probabilistic sample of 113 Portuguese relatives and caregivers of mental health patients. Data were collect in the first semester of 2015. Caregivers were interviewed about sociodemographics, type of care (wholly compensatory, partially compensatory, supervision), time spent in self-care daily activities and its intensity (0-7) and with the Portuguese version of the Zarit Burden Interview [4]. Ethical procedures, according to Helsinki Declaration, were taken into account.

**Results**

Caregivers were mainly females (n=80), with a median age of 51 years, married (n=84), with 12 years of education (n=33) and patients-mothers (n=20). These patients had majorly depressive disorders (36.3%). Caregivers cared for their relatives from 0 up to 54 years (Median=3.25; Mean=7.86; SD=10.78). Most of them (42.5%) corresponded to someone who provided care occasionally. Nevertheless, they provided a wholly compensatory care in most areas of patient self-care daily activities (Mean=26.58; SD=31.20). Preparing and providing meals (Mean=3.54; SD=3.29), Organizing medication (Mean=3.57; SD=3.16) and its administration (Mean=3.30; SD=3.29) was the most frequent self-care provided. Intensity of wholly compensatory care provided was higher in female (t=-2.950; p=0.004) older (r=0.215; p=0.022) relatives, who took full responsibility for caring (p<0.001), living with the patient (t=-2.762; p=0.007). Most caregivers revealed no burden (62.8%; Mean=38.31; SD=22.54). Burden was higher in females (t=-2.869; p=0.005), older (r=0.259; p=0.006) and among primary or secondary caregivers (p<0.001). Impact of giving care, interpersonal relations and perceptions of self-efficacy were also higher in older females with lower education relatives, primary caregivers and caregivers that perspective gravity of their relative illness higher.

**Conclusions**

The caregivers inquired were mainly secondary and provided total care in all areas. The burden presented by those was low, and they provided care especially in preparing meals and organizing and administering medicines. In this sample, care provided and burden were higher in older female caregivers, low educated and with a high perspective of gravity of their relative illness. Intervention among these caregivers is needed, promoting knowledge, improving skills and providing support, which can allow to reduce the psychological consequences of the needed assistance by the relatives.

**References**

1. Pakenham K. Caregiving tasks in caring for an adult with mental illness and associations with adjustment outcomes. Int J Behav Med. 2012;19(2):186-198.

2. Martins S, Bandeira M, Nascimento E. Sobrecarga de familiares de pacientes psiquiátricos atendidos na rede pública. Rev Psiq Clín. 2007;34(6):270-277.

3. Cabral L, Duarte J, Ferreira M, Santos C. Anxiety, stress and depression in family caregivers of the mentally ill. Atención Primaria. 2014;46(5):176-179.

4. Sequeira C. Adaptação e validação da Escala de Sobrecarga do Cuidador de Zarit. Revista Referência. 2010;2(12):9-16.

**Keywords**

Informal caregivers, Mental health, Burden, Family care.

### P168 Your PEL - promote and empower for literacy in health in young people: from investigation to action

#### Hélia Dias^1^, José Amendoeira^1^, Ana Spínola^1^, Maria C Figueiredo^1^, Celeste Godinho^1^, Clara André^1^, Filipe Madeira^1^, Manuela Ferreira^2^, José C Quaresma^3^, Mónica Ferreira^4^, Teresa Simões^5^, Rosário Martins^6^, António Duarte^1^, Madalena Ferreira^1^, Marta Pintor^1^

##### ^1^Escola Superior de Saúde de Santarém, Instituto Politécnico de Santarém, 2005-075 Santarém, Portugal; ^2^Escola Superior de Saúde de Viseu, Instituto Politécnico de Viseu, 3500-843 Viseu, Portugal; ^3^Escola Superior de Saúde de Leiria, Instituto Politécnico de Leiria, 2411-901 Leiria, Portugal; ^4^Agrupamento de Escolas da Chamusca, 2140-052 Chamusca, Portugal; ^5^Agrupamento de Escolas da Golegã, Azinhaga e Pombalinho, 2154-909 Golegã, Portugal; ^6^Unidade Cuidados na Comunidade Chamusca Golegã, Agrupamento de Centros de Saúde Lezíria, 2140-078 Chamusca, Portugal

###### **Correspondence:** Hélia Dias (helia.dias@essaude.ipsantarem.pt)

**Background**

The “Your PEL - Promote and empower for literacy in health in young people” project focus on a health approach, supported by the new technologies, including three different areas: feeding, harmful consumption and sexuality. It is based on scientific evidences of health promotion, on which the need to refocus the action on the results implies the development of appropriate interventions [1, 2]. It is a multi-regional project outlined in the national strategy of smart specialization, in a partnership between IPSantarém – ESSS and ESGT, IPLeiria – ESSL e IPViseu – ESSV, the Agupamento de Escolas da Chamusca, the Agrupamento de Escolas da Golegã and ACES Lezíria – UCC Chamusca/Golegã. The students’ participation reveals itself in the project construction, framed in the curricular program and valued by the knowledge mobilization and by the skill gaining on real context.

**Objective**

Develop a tool for impact evaluation of health education programs for school in the feeding, harmful consumption and sexuality areas, at ages between 12 to 15 years old and monitoring the health determinants and the effectiveness of the developed strategies.

**Methods**

A research-action study divided in three phases: I) construction of the data collection tool and web communication platform; II) evaluation of the intervention needs of the students, development and implementation of the intervention program using the web platform; and III) evaluation of the impact of the program developed. The valuation of the knowledge generated by the project is based on a plan of actions different from the diffusion and dissemination of results, involving the institutions plots and adapted to the very essence.

**Results**

The project strategic impact is situated at two levels. One, evaluated after execution, corresponding to the expected results: tool development for impact measurement of the program and intervention program creation, supported by the web platform. The other, longer in time, that will be evaluated by health gains of the populations in the future.

**Conclusions**

The project’s relevance and originality support themselves on scientific evidence, reviling the monetarization as an essential component of the promotional health program [3, 4], valorising the innovation and sustentation of the action on the results, including more suitable interventions for the young population on a school environment. The scientific and technological knowledge impact generated and disseminated by the project will contribute for regional and national valorisation, on a logic translation of knowledge.

**References**

1. Amendoeira J, Carreira T, Cruz O, Dias H, Santiago MC. Programas de educação sexual em meio escolar: Revisão sistemática da literatura”, Revista da UIIPS. 2013;1(4):198-211.

2. André C, Amendoeira J. Intervention programs for the prevention of smoking in children and adolescents: A systematic literature review. Atención Primaria. 2013;45(Especial C):23.

3. Matos M, Simões C, Camacho I, Reis M. A Saúde dos Adolescentes Portugueses em tempos de recessão. HBSC; 2015. Acedido em:www.aventurasocial.com

4. Ministério da Saúde. Plano Nacional de Saúde - Orientações estratégicas para 2012-2016. Lisboa, Portugal: DGS; 2012.

**Keywords**

Health promotion, Empowerment, Health literacy, Young population.

### P169 Effects of education on functional health: mobility and musculoskeletal back pain in the elderly

#### Gustavo Desouzart^1,2,3^, Cristina Farias^3^, Sandra Gagulic^1,2,3^

##### ^1^Research in Education and Community Intervention, Piaget Institute, 1950-157 Lisbon, Portugal; ^2^Piaget Institute, 1950-157 Lisbon, Portugal; ^3^Health School of Viseu, Piaget Institute, 3515-776 Viseu, Portugal

###### **Correspondence:** Gustavo Desouzart (gustavodesouzart@gmail.com)

**Background**

The bio-psycho-social changes that seniors undergo determine the importance of promoting a better quality of life, simultaneously with the extension of life expectancy. In what concerns this theme, a new concept of aging emerges, “the active aging” [1,2].

**Objective**

The aim of the study is to evaluate the effects of a functional health education program on the functional capacity of a group of elderly.

**Methods**

This is an experimental study, with a sample of 20 elderly people aged 67-91 years (mean of 80.70 ± 5.99), who attended day centres and were enrolled in the “Atividade Sénior” Program. This Program was developed by the social responsibility of the Viseu city council and is very important among the community, to promote physical activity in the population. Elders were randomly assigned to Experimental (n=10) and Control (n=10) groups. During this study all individuals maintained the physical activity training of the “Atividade Sénior” Program, where the experimental group had a training with aerobic, flexibility and strength components, associated with stimulation. The exercise program lasted 12 weeks with a frequency of 3 times a week, with each session lasting 30 minutes. The study was pre and post-tested, with the following scales: Timed Up and Go (TUG), to check the functional capacity [3] and the Visual Analogue Scale (VAS) to check pain level [4], with the use of body chart for identifying the site of pain. The study was submitted to the International Ethics Committee in accordance with the guidelines of the World Health Organization (WHO).

**Results**

In the study population, 80% of the elderly indicated some type of back pain. Of these, 50% indicated that the pain is chronic and 81.2% indicated that the main location is low back pain. At the beginning of the study participants had a mean pain according to the VAS of 5.70; the experimental group with a mean pain score of 7.10 and the control group with an average of 4.30. After 12 weeks, the participants presented a reduction in pain level (3.78), the experimental group with a significant reduction to 3.38 (p= 0.034), and the control group also with a reduction to 3.70 (p= 0.529). Regarding the functionality, according to the TUG, the experimental group obtained the mean initial time of 21.10 +10.06 and the final time of 16.32 ± 7.80 (p= 0.043), compared to the control group (p= 0.436).

**Conclusions**

The implemented program demonstrated that physical exercise in general allows global improvements in the elderly population of day centres, and the specific implementation of a functional mobility physiotherapy program has allowed significant results. Then there is the awakening to this theme.

**Acknowledgements**

Authors would like to thank those responsible for the senior activity program of the Municipal Council of Viseu, as well as to thank participants, local promoters and day centres.

**Trial registration**

Australian New Zealand Clinical Trials Registry (ANZCTR) registration number: ACTRN12617001170314.

**References**

1. Desouzart G, Matos R, Melo F, Filgueiras E. Effects of sleeping position on back pain in physically active seniors: A controlled pilot study. Work. 2016;53(2).

2. WHO AA. A policy Framework. Geneva, Switz World Heal Organ. 2002.

3. Steffen TM, Hacker TA, Mollinger L. Age-and gender-related test performance in community-dwelling elderly people: Six-Minute Walk Test, Berg Balance Scale, Timed Up & Go Test, and gait speeds. Phys Ther. Oxford University Press; 2002;82(2):128–137.

4. Ferreira-Valente MA, Pais-Ribeiro JL, Jensen MP. Validity of four pain intensity rating scales. PAIN®. Elsevier; 2011;152(10):2399–2404.

**Keywords**

Functional health, Senior activity, Back pain.

### P170 The Practice Environment Scale of the Nursing Work Index (PES-NWI): validation to primary health care

#### Eva Menino^1^, Maria A Dixe^2,3^, Clarisse Louro^2^, Francisco Stefanie^4^

##### ^1^Escola Superior de Saúde da Cruz Vermelha Portuguesa, 1300-906 Lisbon, Portugal; ^2^School of Health Sciences, Polytechnic Institute of Leiria, 2411-901 Leiria, Portugal; ^3^Center for Innovative Care and Health Technology, Instituto Politécnico de Leiria, 2411-901 Leiria, Portugal; ^4^Posto Saúde da Junta de Freguesia de Penha de França, 1170-070 Lisboa, Portugal

###### **Correspondence:** Eva Menino (eva.guilherme@gmail.com)

**Background**

The environment of nurses' professional practice and the adequacy of resources are structural factors that are related to the results and quality of care. The validated PES scale for the Portuguese population is adequate to evaluate the conditions for nursing practice, and it was validated in Portugal in hospital settings but not in primary health care. This study proposes to make content validation for primary health care and its psychometric validation, having obtained previous authorization of the author for this validation.

**Objective**

Validation of the Practice Environment Scale of the Nursing Work Index (PES-NWI) for Primary Health Care.

**Methods**

The original scale was submitted to a panel of 5 experts. We decided to keep items with levels of agreement greater than 75%, after analysing and after making the changes suggested, we submitted the scale to a second round by the same panel of experts. The new version was used to determine its psychometric characteristics and its revalidation.

**Results**

The PES version adapted for primary health care presents a Cronbach value of 0.905, meaning a very good internal consistency, with reasonable correlation values for each item with the total of the scale (0.315-0.685). In the analysis through the matrix of main components, only factorial loads above 0.30 were considered, so it was necessary to regroup the items by factors, other than the original scale. Considering the KMO value of 0.797, which indicates that there is a good correlation between the variables, and with a Bartlett test (1407.494, p < 0.001), we could infer that the variables are significantly correlated and consequently we can confirm the validity of the adapted scale. Between the original scale and the one obtained, we identified factors that focus on the same areas, but there was a change in the items that make up these factors. These changes are expected and show greater adequacy regarding the differences between primary and hospital health care. There are differences regarding the items that portray the nurse's functions, which is in line with the evidence that shows that the community context is generally more favourable for nurses to perform autonomous functions.

**Conclusions**

It is considered essential to use valid instruments to evaluate the characteristics of the nursing practice environment in Primary Health Care, since it is the first “door” to access to health services, with nurses having a central role in this context.

**Keywords**

Public Health Nursing, Continuous quality management, Validation studies.

### P171 Elaboration of an IAP prevention clinical practice guideline using the ADAPTE methodology

#### Ana Sousa^1,2,3^, Cândita Ferrito^4^, José A Paiva^2^

##### ^1^Universidade Católica Portuguesa, 4169-005 Porto, Portugal; ^2^Centro Hospitalar S. João, 4200-319 Porto, Portugal; ^3^Escola Superior de Enfermagem do Porto, 4200-072 Porto, Portugal; ^4^Escola Superior de Saúde, Instituto Politécnico de Setúbal, 2914-503 Setúbal, Portugal

###### **Correspondence:** Ana Sousa (sabrinasousa72@hotmail.com)

**Background**

Intubation associated pneumonia (IAP) is the most frequent health care associated infection in Intensive Care Units (ICU), causing increased length of stay, multiple health and economic costs and antibiotic resistance [1-4]. The impact of this infection motivated this study.

**Objective**

Elaborate a Clinical Practice Guideline (CPG).

**Methods**

Using the ADAPTE methodology, we performed the following sequence of steps: Configuration (definition of the study area, objectives and research questions), Adaptation (search for guidelines and other relevant documents, quality selection and assessment of currency, acceptability and applicability and elaboration of recommendations); Finalization (production of the final document, implementation and statistical data collection in terms of feedback by users about its contributions and final result). We evaluated the document regarding its quality through the application of the AGREE II instrument and its clarity, content and applicability through the presentation of the CPG draft and the application of a questionnaire to all its users in three ICUs. These results were processed using the Statistical Package for the Social Sciences system.

**Results**

After assessment of evidence grade, applicability and acceptability, we included in the CPG eight recommendations: avoid endotracheal intubation; daily sedation assessment and reduction; daily ventilator weaning; change ventilator circuit only when visibly soiled; head of bed elevation of 30-45º; early exercise and patient mobilization; maintenance of endotracheal tube cuff pressure between 20-30 cm H_2_O; oral hygiene care with chlorhexidine 0.12% or 0.20% [5-9]. Regarding the application of the AGREE II, the CPG obtained a rating of 7 in all domains, which corresponds to a high quality. The questionnaire obtained a total of 82 responses, which corresponds to a rate of 45.6% of the respondents. All of the health care professionals stated that CPG’s objective is clear, relevant and agree with the content and the adequacy of the recommendations. Regarding applicability, 89% of the respondents stated that is applicable.

**Conclusions**

There is a lack of systematization and adequacy concerning CPG’s elaboration. This methodology allowed us to find the most recent recommendations regarding IAP prevention and adapt to a specific scenario successfully according to quality and user’s evaluation. We can find a limitation of the study in the fact that the evidence supporting some of the recommendations included in the CPG is moderate, and there is a shortage of experimental studies that assess the impact of implementing each individual recommendation. In the next phase

**References**

1. Agbaht K, Díaz E, Muñoz E, Lisboa T, Gómez F, Depuydt PO, et al. Bacteremia in patients with ventilator-associated pneumonia is associated with increased mortality: a study comparing bacteremic vs. nonbacteremic ventilator-associated pneumonia. Crit Care Med. 2007;35:2064-2070.

2. American Thoracic Society. Guidelines for the management of adults with hospital-acquired, ventilator-associated, and healthcare-associated pneumonia. Am J Respir Crit Care Med. 2005;171(4):388-416.

3. Tablan OC, Anderson LJ, Besser R, Bridges C, Hajjeh R. Guidelines for preventing health-care-associated pneumonia., 2003: recommendations of CDC and the Healthcare Infection Control Practices Advisory Committee. MMWR Recomm Rep. 2004;53:1-36.

4. Tejerina E, Frutos-Vivar F, Restrepo MI, Anzueto A, Abroug F, Palizas F, et al. Incidence, risk factors, and outcome of ventilator-associated pneumonia. J Crit Care. 2006;21:56-65.

5. Klompas M, Branson R, Eichenwald EC, Greene LR, Howell MD, Lee G, et al. Strategies to prevent ventilator-associated pneumonia in acute care hospitals: 2014 update. Infect Control Hosp Epidemiol. 2014;35 Suppl 2:S133-54.

6. Shi Z, Xie H, Wang P, Zhang Q, Wu Y, Chen E, et al. Oral hygiene care for critically ill patients to prevent ventilator-associated pneumonia. Cochrane Database Syst Rev. 2013;8:Cd008367.

7. Bo, Lulong & Li, Jinbao & Tao, Tianzhu & Bai, Yu & Ye, Xiaofei & S. Hotchkiss, Richard & H. Kollef, Marin & Crooks, Neil & Deng, Xiaoming. Probiotics for preventing ventilator-associated pneumonia. Cochrane Database Syst Rev. 2014; 10.

8. Paiva et al. “Feixe de Intervenções” de Prevenção de Pneumonia Associada à Intubação. Departamento da Qualidade na Saúde da Direção-Geral da Saúde. Direção-Geral da Saúde. 2015; 021/2015.

9. National Guideline C. Prevention of ventilator-associated pneumonia. Health care protocol. 2011.

**Keywords**

ADAPTE, Clinical Practice Guideline, Health Care Associated Infection, Intubation-associated pneumonia, ICU.

### P172 The factorial analysis of a quality of life scale for people addicted to drugs in methadone programs

#### Paulo Seabra^1^, José Amendoeira^2^, Luís Sá^1^, Olga Valentim^3^, Manuel Capelas^1^

##### ^1^Interdisciplinary Research Health Center, Portuguese Catholic University, Health Sciences Institute, 1649-023 Lisbon, Portugal; ^2^Interdisciplinary Research Health Center, Polytechnic Institute of Santarém, Health School, 2005-075 Santarém, Portugal; ^3^Portuguese Catholic University, Health Sciences Institute, 1649-023 Lisbon, Portugal

###### **Correspondence:** Paulo Seabra (pauloseabra@ics.lisboa.ucp.pt)

**Background**

The evaluation scale of drug users Quality of life (QoL) in a substitution program with methadone, was developed with 21 items, two subscales, “family and economic situation” (11 items) and “personal satisfaction” (10 items) [1]. Concerning their reliability, in the validation study for the Portuguese population in 2005 (n=236) was obtained an Alfa of Cronbach of 0.88 and in a recent study in 2011(n=308) of 0.93 [1].

**Objective**

To determine a scale factorial structure and its psychometric properties.

**Methods**

Methodological study. Participants – 180 drug users participated, aging an average of 41 years (SD=7.58 [24-69]), mostly men (73.3%), single (55.6%) and with children (52.8%), from 3 outpatients drug units. Data analyses - The correlation matrix of the items was evaluated through exploratory and confirmatory factorial analysis. The factorial load as well as the internal consistency estimated the dimensionality.

**Results**

In the reliability analysis with 21 items was found an Alfa of Cronbach of 0.89, all communalities >0.40, KMO=0.88 (p<0.001) and 58.42% of explained variance by 5 factors. When extracted item 18 (item-total correlation 0.16), all items assumed an item-total correlation > 0.20; alpha increased (α=0.90) and KMO increased to 0.88 maintaining the stability of the Bartlett test; communalities maintained above 0.40; total variance explained by the 5 factors increased to 60.4%, but 5 factors diverged from theoretical matrix and 10 items weighed in more than one factor. Through confirmatory analysis (excluding item 18) forcing for the 2 original scale factors, we verified that 4 items had commonalities <0.30. The total variance explained after the spin fell to 43.11% and 4 items weighed in more than one factor. We explored with 3 factors, KMO maintained in 0.88, Bartlett test remained within criteria, total variance explained after de rotation stayed 49.6%. Although this structure presents 3 items weigh in more than one factor, justifies its maintenance by underlying the theoretical model. Alpha is higher to initial (α=0.90). The extraction of any item will weaken scale consistency. The most stable structure was with 3 renamed factors: 1- Personal satisfaction and self-care (8 items) (38.5% explained variance); Social Family situation (8 items) (7.4%); 3- Socio professional and economic situation (4 items) (6.5%). The fidelity of the scale is reinforced by the internal consistency of its subscales, factor 1 α=0.85; factor 2 α=0.79; factor 3 α=0.72 and by the correlation between them (0.51-0.67; p<0.01).

**Conclusions**

Good internal consistency. Factorial analysis supported by the theoretical matrix. Good discriminant capacity by differences pointed out in some variables.

**References**

1. Murcho N, Pereira P. A qualidade de vida dos doentes toxicodependentes em programas de substituição com metadona no Algarve: Um estudo comparativo da sua situação em 2003 e 2008. Rev Investig em Enferm. 2011;(23):57–64.

**Keywords**

Quality of Life, Substance related disorders, Assessment, Nursing, Methadone.

### P173 Occupational sedentary lifestyle and overweight among workers of a higher education institution – Coimbra

#### Sónia Fialho^1^, Anabela Martins^2^, João Almeida^3^

##### ^1^Dietetics and Nutrition Department, Coimbra Health School, 3046-854 Coimbra, Portugal; ^2^Physiotherapy Department, Coimbra Health School, 3046-854 Coimbra, Portugal; ^3^Environmental Health Department, Coimbra Health School, 3046-854 Coimbra, Portugal

###### **Correspondence:** Sónia Fialho (sonia.fialho@estescoimbra.pt)

**Background**

In the last decades, with the introduction of changes in the work processes and with the innovation inherent to new technologies, there has been an increase of the sedentary labour lifestyle, where the worker remains sitting for long periods of time in a working day. Sedentary behaviour is associated with an increased risk of developing chronic diseases such as obesity, type II diabetes, cardiovascular diseases, and these diseases are the main cause of mortality and morbidity in Portugal.

**Objective**

This study aims to evaluate the relationship between sedentary work and overweight in teaching and non-teaching workers.

**Methods**

The Occupational Sitting and Physical Activity Questionnaire (OSPAQ) was applied and then calculated the percentage of the activity for each domain (sitting, standing, walking) by the number of hours worked per day. Data on age, gender and body mass index, between December 2017 and January 2018, were collected from a sample of 58 adult men and women in full-time employment at the time of the study. To study the correlation between the percentage of the occupational sitting by the number of working hours per day and overweight, authors analysed the information with SPSS Statistics.

**Results**

In the present study, 39 of the individuals were females and 19 males, aged between 31 and 62 years. In the analysis done to the OSPAQ, 44 (75.8%) individuals spend more than 50% of their working day in the sitting position. In relation to the BMI, considering the purpose of the study and according to the classification of the World Health Organization, 32 (55.2%) of the individuals presented a BMI ≥ 25. Pearson correlation revealed that there is no association between the sitting time and the BMI ≥ 25 (p> 0.05).

**Conclusions**

With this study it was possible to verify that there are individuals with a sedentary lifestyle associated to their work day. Although in this study there is no association between occupational sitting and BMI ≥ 25, despite there are studies that demonstrate a significant association between these two parameters. A further study, including other criteria, is in progress, involving the anthropometric level, such as body fat, waist circumference and physical activity assessments.

**References**

1.Yang L, Hipp JA, Lee JA., Tabak RG, Dodson EA, Marx CM, Brownson RC. Work-related correlates of occupational sitting in a diverse sample of employees in Midwest metropolitan cities. Preventive Medicine Reports. 2017;6:197-202.

2. Chau J, Van der Ploeg H, Dunn S, Kurko J, Bauman A. Validity of the occupational sitting and physical activity questionnaire. Medicine & Science in Sports & Exercise. 2012;44(1):118-125.

3. Lin T, Courtney TK, Lombardi DA, Verma SK. Association between sedentary work and BMI in a U.S. national longitudinal survey. American Journal of Preventive Medicine. 2015;49(6):117-123.

4. Mummery WK, Schofield GM, Steele R, Eakin EG, Brown WJ. Occupational sitting time and overweight and obesity in Australian workers. American Journal of Preventive Medicine. 2005;29:91-97.

**Keywords**

Health promotion, Sedentary behaviour, Body mass index, Workplace.

### P174 Oral health assessment among elderly stroke patients

#### Nélio Veiga^1,2^, Ricardo Figueiredo^1^, António Coelho^1^, André Almeida^1^, Gonçalo Lopes^1^, Salvatore Bellantone^1^

##### ^1^Health Sciences Institute, Portuguese Catholic University, 3504-505 Viseu, Portugal; ^2^Center for Interdisciplinary Health Research, Portuguese Catholic University, 3504-505 Viseu, Portugal

###### **Correspondence:** Nélio Veiga (nelioveiga@gmail.com)

**Background**

Oral hygiene can become a very difficult task for patients who have suffered a stroke, due to motor and cognitive complications and lack of coordination, which usually accompany the post-stroke period. Many of these patients require support from caregivers to properly hygiene the oral cavity as well as, their prostheses.

**Objective**

Characterization of oral health among elderly who have suffered a stroke.

**Methods**

A cross-sectional observational study was carried out in institutionalized elderly individuals aged between 60 and 98 years old. Data collection was carried out in two households in the city of Viseu, Portugal: Fundação Dona Mariana Seixas and Viscondessa House of São Caetano. Due to health limitations of the study participants, in terms of speech and cognitive problems, the final sample consisted of 30 participants. Data collection was achieved through the application of a questionnaire.

**Results**

Of the final sample, 20 elderlies had at least one stroke episode, representing 67% of the sample. Of these 20 elderlies, 5 reported having had cognitive or speech sequelae, while the remaining 15 reported that the main sequel was motor dysfunction. Of a total of 30 institutionalized elderly, 19 had total absence of dental pieces, corresponding to 63% of the sample. However, the remaining 37% reported multiple dental losses due mainly to dental caries and periodontal problems. These problems may be associated with deficient oral hygiene of the participants, where 18 affirm that they perform dental or denture brushing only once a day, while 40% said they did not take care of their own oral hygiene.

**Conclusions**

Within the limitations of this study, it is possible to conclude that stroke is a constant among the Portuguese population and that patients who have suffered from stroke have a lower oral hygiene.

**Keywords**

Stroke, Oral hygiene, Elderly, Oral health, Dysfunction.

### P175 The daily life of people with human immunodeficiency virus in an island space: what trends?

#### Gilberta Sousa^1^, Maria A Lopes^2^, Vitória Mourão^3^

##### ^1^Universidade da Madeira, 9000-034 Funchal, Madeira, Portugal; ^2^Escola Superior de Enfermagem Lisboa, 1600-190 Lisboa, Portugal; ^3^Universidade de Lisboa, 1649-004 Lisboa, Portugal

###### **Correspondence:** Gilberta Sousa (gfranca@uma.pt)

**Background**

Human immunodeficiency virus/acquired immunodeficiency virus (HIV /AIDS) infection continues to haunt the lives of millions of people and, despite the progress made in the treatment of HIV/AIDS, it is estimated that 36.7 million people in the world living with HIV in 2017 [1]. In the year 2016 and until June 30, 2017, 1,030 new cases of HIV infection were reported, corresponding mostly (99.7%) to individuals aged ≥ 15 years. Portugal has had the highest rates of new cases of HIV and AIDS infection in the European Union [2]. We found that it is not possible to stop the HIV epidemic only with medical interventions. It is vital to address in everyday life the underlying social issues that prevent people from accessing interventions for prevention, diagnosis and treatment of infection, including unequal human rights, stigma and discrimination. When a person is stigmatized or unable to access services as a result of discrimination, the health of the entire community is threatened and epidemic HIV transmission continues to expand rather than to contract [3].

**Objective**

To understand how people live with HIV/AIDS, everyday life in an island space.

**Methods**

Qualitative study, grounded theory [4], in-depth interviews were carried out, in a convenience sample, to seropositive adults of any sexual orientation who wished to talk about their experience. Data analysis included initial coding, grouping of codes, identification of categories/subcategories and memos. Fulfilled ethical requirements and assent of an ethics committee were obtained.

**Results**

Participants were between the ages of 25 and 67 and had primary education as well as secondary education. The analysis of the data gave rise to three categories: “living with fear”, “surviving” and “facing fear”. The data are discussed in the light of the theory of transitions [5] of how to live everyday life in an island space.

**Conclusions**

We hope that the findings help in understanding the daily lives of people with HIV/AIDS, because in order to overcome this transition they have to reconfigure their way of living, especially when living on an Island. Realize how they face and fight in the daily life against stigma; what are the most demanding situations they face and what strategies they use. The strategies used and suggested will give subsidies to the health system and nursing professionals towards the design of new programs that will enable each patient to respond to their individual needs with the resources that each one has.

**References**

1. UNAIDS, Right to Heath - My health, My right. 2017. Available in: http://www.unaids.org/sites/default/files/media_asset/RighttoHealthReport_Full_web% 2020%20Nov.pdf

2. PORTUGAL. Ministério da Saúde. Instituto Nacional de Saúde Doutor Ricardo Jorge, IP, Infeção VIH/SIDA: a situação em Portugal a 31 de dezembro de 2016/Departamento de Doenças Infeciosas do INSA. Unidade de Referência e Vigilância Epidemiológica; Programa Nacional para a Infeção VIH/SIDA. Direção-Geral da Saúde. - Lisboa: Instituto Nacional de Saúde Doutor Ricardo Jorge, IP, - (Documento VIH/SIDA; 148). 2017.

3. PEPFAR. President’s Emergency Plan for AIDS Relief. U.S. Department of State Office of the U.S. Global AIDS Coordinator and Health Diplomacy 2017. https://www.pepfar.gov/documents/organization/267809.pdf

4. Charmaz K. Constructing Grounded Theory. 2nd edition. London: Sage Publications Limited ; 2014.

5. Meleis AI, Sawyer LM, Im EO, Hilfinger Messias DK, Schumacher K. Experiencing Transitions: An Emerging Middle-Rang Theory. ANS Adv Nurs Sci. 2000;23(1):12-28.

**Keywords**

People with HIV/AIDS, Theory of transitions, Everyday life, Stigma, Nursing.

### P176 Validation of the nursing diagnosis of impaired walking in elderly

#### Cristina Marques-Vieria^1,2^, Luís Sousa^3,4^, Débora Costa^5^, Cláudia Mendes^5^, Lisete Sousa^5,6^, Sílvia Caldeira^1,2^

##### ^1^Lisbon School of Nursing, Institute of Health Sciences, Portuguese Catholic University, 1649-023 Lisbon, Portugal; ^2^Interdisciplinary Research Center for Health, Portuguese Catholic University, 1649-023 Lisbon, Portugal; ^3^Curry Cabral Hospital, Central Lisbon Center Hospital, 1069-166 Lisbon, Portugal; ^4^Atlântica School of Health, 2730-036 Barcarena, Portugal; ^5^Faculty of Science, Lisbon University, 1749-016 Lisbon, Portugal; ^6^Statistics and Applications Center, Lisbon University, 1749-016 Lisbon, Portugal

###### **Correspondence:** Cristina Marques-Vieria (cristina_marques@ics.lisboa.ucp.pt)

**Background**

The increase in longevity causes restriction of activity in the elderly, causing changes on the execution of daily activities and consequently on the quality of life [1]. Walk is an activity that requires using a variety of skills and can be highly complex particularly for the elderly people [2]. The nursing diagnosis impaired walking is part of NANDA International since 1998 and requires further validation to improve the clinical evidence [3].

**Objective**

To validate the nursing diagnosis impaired walking in a sample composed of elderly.

**Methods**

Observational, cross-sectional and quantitative study. After the first research phase of systematic literature review several defining characteristics and related factors of the diagnosis impaired walking have been listed.2 Then, the translation, linguistic and cultural adaptation of the nursing diagnosis was conducted, and finally, the clinical validation of the diagnosis using the clinical validation model of Richard Fehring [4], in a sample of elderly and counting on the collaboration of registered nurses and rehabilitation nurses to collect the data and fill the questionnaires, which comprised demographic data, the defining characteristics, related factors and falls efficacy scale international [5]. The study was approved by the ethical committee of SESARAM. E.P.E (Madeira Island Healthcare System).

**Results**

In the systematic literature review 17 defining characteristics and 34 etiological factors of impaired walking have been identified. A European Portuguese version was obtained to validate in a sample of 126 elderly, whose average age was 73.86 years, mostly female, with the primary school, in a situation of retirement, widowed and with history of falls. The prevalence of “impaired walk” was 64.3% according to the expert's opinion and 67.5% according to the elderly. All defining characteristics and related factors have been validated. The most sensitive defining characteristic was nine (e.g. impaired ability of gait speed) and also four related factors (fear of falling, physical deconditioning, medication and feminine gender).

**Conclusions**

This study justifies the need to review the defining characteristics and related factors of impaired walking. The identification of the most sensitive defining characteristics facilitates nurses’ clinical reasoning and interventions towards effective nursing outcomes.

**References**

1. Marques-Vieira CM, Sousa LM, Carias JF, Caldeira SM. Nursing diagnosis “impaired walking” in elderly patients: integrative literature review. Rev Gaucha Enferm. 2015;36(1):104-111.

2. Marques-Vieira CM, Sousa LM, Sousa LM, Berenger SM. The nursing diagnosis impaired walking in elderly: systematic literature review. Texto & Contexto Enferm. 2016;25(3):e3350015.

3. Herdman HT, Kamitsuru S, editors. Nursing Diagnoses: Definitions & Classification 2018-2020. Oxford: Wiley-Blackwell; 2017.

4. Fehring RJ. Methods to validate nursing diagnoses. Heart Lung. 1987;16(6):625-629.

5. Marques-Vieira CM, Sousa LM, Severino S, Sousa L, Caldeira S. Cross-cultural validation of the falls efficacy scale international in elderly: systematic literature review. J Clin Gerontol Geriatr. 2016;7(3):72-76.

**Keywords**

Nursing, Nursing Diagnosis, Walking, Gait, Validation studies.

### P177 Validation of the nursing diagnosis risk for falls in elderly

#### Cristina Marques-Vieria^1,2^, Luís Sousa^3,4^, Débora Costa^5^, Cláudia Mendes^5^, Lisete Sousa^5,6^, Sílvia Caldeira^1,2^

##### ^1^Lisbon School of Nursing, Institute of Health Sciences, Portuguese Catholic University, 1649-023 Lisbon, Portugal; ^2^Interdisciplinary Research Center for Health, Portuguese Catholic University, 1649-023 Lisbon, Portugal; ^3^Curry Cabral Hospital, Central Lisbon Center Hospital, 1069-166 Lisbon, Portugal; ^4^Atlântica School of Health, 2730-036 Barcarena, Portugal; ^5^Faculty of Science, Lisbon University, 1749-016 Lisbon, Portugal; ^6^Statistics and Applications Center, Lisbon University, 1749-016 Lisbon, Portugal

###### **Correspondence:** Cristina Marques-Vieria (cristina_marques@ics.lisboa.ucp.pt)

**Background**

Falls and their consequences are critical for for elderly well-being quality of life, for caregivers, and for health care providers [1]. The nursing diagnosis risk for falls is listed in NANDA International since 2000 [2]. This diagnosis seems particularly important in planning effective nursing care for the community-dwelling elderly.

**Objective**

To validate the nursing diagnosis risk for falls in a sample of elderly.

**Methods**

Observational, cross-sectional and quantitative study conducted in three phases. The first phase, corresponded to a systematic literature review to identify the risk factors of risk for falls [3]. The second phase consisted of the translation, linguistic and cultural adaptation of the nursing diagnosis for European Portuguese language. The third, was the clinical validation of the diagnosis using the clinical validation model of Richard Fehring [4], in a sample of elderly and counting on the collaboration of registered nurses and rehabilitation nurses to collect the data and fill the questionnaires, which comprised demographic data, the risk factors and falls efficacy scale international [5]. The study was approved by the ethical committee of SESARAM. E.P.E (Madeira Island Healthcare System).

**Results**

A total of 50 risk factors of risk for falls have been identified in the systematic literature review. A European Portuguese version was obtained and submitted to the clinical validation in a sample of 126 elderly, whose average age was 73.86 years, mostly female, with the primary school, in a situation of retirement, widowed and with history of falls. The prevalence of risk for falls was 68.3% in the expert's opinion and 63.5% in the opinion of the elderly. All risk factors have been validated. The most sensitive risk factor was history of falls, comorbidities, feminine gender, polymedication, difficulty with gait, and drugs.

**Conclusions**

This study found the main risk factors for falls in a sample of community-dwelling elderly. The identification of the most sensitive risk factors may support nurses’ clinical reasoning and interventions for effective fall prevention.

**References**

1. Lusardi MM, Fritz S, Middleton A, Allison L, Wingood M, Phillips E, Criss M, Verma S, Osborne J, Chui KK. Systematic Reviews. J Geriatr Phys Ther. 2017;40:1-36.

2. Herdman HT, Kamitsuru S, editors. Nursing Diagnoses: Definitions & Classification 2018-2020. Oxford: Wiley-Blackwell; 2017.

3. Sousa LM, Marques-Vieira CM, Caldevilla MN, Henriques CM, Severino SS, Caldeira SM. Risk for falls among community-dwelling older people: systematic literature review. Rev Gaucha Enferm. 2017;37(4):e55030.

4. Fehring RJ. Methods to validate nursing diagnoses. Heart Lung. 1987;16(6 Pt 1):625-629.

5. Marques-Vieira CM, Sousa LM, Severino S, Sousa L, Caldeira S. Cross-cultural validation of the falls efficacy scale international in elderly: systematic literature review. J Clin Gerontol Geriatr. 2016;7(3):72-76.

**Keywords**

Nursing, Nursing Diagnosis, Risk for falls, Fear of falling, Validation studies.

### P178 Teachers and professors’ mental health: prevalence of self-reported psychological symptoms

#### Ana Querido^1,2^, Catarina Tomás^1,2^, Daniel Carvalho^3^, Marina Cordeiro^1,2^, João Gomes^3^

##### ^1^School of Health Sciences, Polytechnic Institute of Leiria, 2411-901 Leiria, Portugal; ^2^Center for Innovative Care and Health Technology, Polytechnic Institute of Leiria, 2411-901 Leiria, Portugal; ^3^Santo André Hospital, Hospital Center of Leiria, 2410-197 Leiria, Portugal

###### **Correspondence:** Ana Querido (ana.querido@ipleiria.pt)

**Background**

The work of teaching professionals is recognized to be demanding, involving dynamic interactions with students, parents, colleagues and school authorities [1]. Teaching has been ranked as one of the most stressful profession and its nature is applicable to all professional teaching roles. Several research reports have consistently documented physical and psychological symptoms experienced by teaching professionals. Several physical complains and psychosomatic symptoms such as lower back pain, headache, voice disorders and anxiety are frequently faced by teaching professionals, both in secondary and higher education, especially in women [1-5]. Presence of psychopathology symptoms in teachers are related to their rating of children mental health behaviors [6], as well as determinants to professional burnout. Therefore, identification of psychological symptoms among teachers is relevant in secondary and higher education.

**Objective**

To identify the prevalence of self-reported psychological symptoms; Characterize the symptoms by its dimension and determine the differences in psychological symptoms between high school teachers and higher education professors.

**Methods**

Cross-sectional correlational study, with a non-probabilistic sample of 96 Portuguese teaching professionals. Data were collected using an on-line questionnaire composed by sociodemographic questions and the Portuguese version of Brief Symptom Inventory (BSI) - 53 items covering nine symptom dimensions: Somatization, Obsession-Compulsion, Interpersonal Sensitivity, Depression, Anxiety, Hostility, Phobic anxiety, Paranoid ideation and Psychoticism; and three indices of distress: Global Severity Index (GSI), Positive Symptom Distress Index (PSDI), and Positive Symptom Total (PST). Ethical procedures were taken into account.

**Results**

Teaching professionals were mostly women (70.8%), aged between 30 and 62 years old (Mean=44.8; SD=7.86), 43.8% with a bachelor degree, 27.1% diagnosed with a mental disease, and 41.1% acquainted with mental health patients. Professors (37.5%) were from different fields, including health, engineering, arts, communication, social sciences, marketing and sports. High school teachers (62.5%) were mainly from sociology, philosophy and mathematics. The most scored dimension was Obsession-Compulsion in high school teachers (Mean=1.03; SD=0.75). Globally high school teachers revealed more symptoms of distress than higher education professors. Significant differences between groups were found in Somatization, Obsession-Compulsion, Interpersonal Sensitivity, Depression, Anxiety, Phobic Anxiety, Psychoticism, GSI and PST (p < 0.05). In the 53 BSI items, the PST was low (Mean=19.09; SD=12.70).

**Conclusions**

Prevalence of symptoms were high in the samples of teaching professionals, although they experienced psychological distress in low intensity. Differences between high school teachers and higher education professors were highlighted in this study. Therefore, there is a need for intervention among high school teachers to minimize the impact of detecting mental disorders in students, as well as preventing professional absents and burn-out.

**References**

1. Au DWH, Tsang HWH, Lee JLC, Leung CHT, Lo JYT, Ngai SPC, et al. Psychosomatic and physical responses to a multi-component stress management program among teaching professionals: A randomized study of cognitive behavioral intervention (CB) with complementary and alternative medicine (CAM) approach. Behav Res Ther. 2016;80:10–16.

2. Chan AHS, Chong EYL. Subjective Health Complaints of Teachers From Primary and Secondary Schools in Hong Kong. Int J Occup Saf Ergon. 2010;16(1):23–39.

3. Ferreira RC, Silveira AP da, Sá MAB de, Feres S de BL, Souza JGS, Martins AME de BL. Transtorno mental e estressores no trabalho entre professores universitários da área da saúde. Trab Educ e Saúde. 2015;13(suppl 1):135–155.

4. Seibt R, Spitzer S, Druschke D, Scheuch K, Hinz A. Predictors of mental health in female teachers. Int J Occup Med Environ Health. 2013;26(6):856-869.

5. Zamri EN, Moy FM, Hoe VCW. Association of psychological distress and work psychosocial factors with self-reported musculoskeletal pain among secondary school teachers in Malaysia. PLoS One. 2017;12(2):e0172195.

6. Kokkinos CM, Kargiotidis A. Rating students’ problem behaviour: the role of teachers’ individual characteristics. Educ Psychol. 2016;36(8):1516–32.

**Keywords**

Mental health, Psychological symptoms, Teachers, Teaching professionals.

### P179 Generating high vegetable liking among young children to promote healthy eating: results from an intervention at a kindergarten school

#### Cátia Braga-Pontes^1,2^, Ana Pinto Moura^3^, Luís Cunha^4^

##### ^1^Center for Innovative Care and Health Technology, Polytechnic Institute of Leiria, 2411-901 Leiria, Portugal; ^2^School of Health Sciences, Polytechnic Institute of Leiria, 2411-901 Leiria, Portugal; ^3^Sciences and Technology Departament, Open University of Portugal, 4200-055 Porto, Portugal; ^4^Department of Geosciences, Environment and Spatial PlanningsFaculty of Sciences, University of Porto, 4485-661 Vila do Conde, Portugal

###### **Correspondence:** Luís Cunha (lmcunha@fc.up.p)

**Background**

Fruit and vegetables have always played a prominent role in dietary recommendations because of their high concentration of vitamins, minerals, phytochemicals and because they are a great source of fibre [1,2]. Recent data show that in general, the population should at least double consumption of fruit and vegetables in order to reach the 400g/day recommended by World Health Organization (WHO) [3]. In Portugal, the data presented by the National Food Survey in 2017 showed that 68.9% of children do not consume more than 400g/day of fruit and vegetables, highlighting a lower consumption of vegetables compared to fruit [4].

**Objective**

The purpose of this study was to evaluate the impact of a food intervention, with exposure combined with a tangible reward, on food neophobia, liking and intake of different vegetables, at the kindergarten school environment, in the South of Portugal.

**Methods**

Children (n=82) aged 2 to 5 years old, from different classes, were randomly assigned by class to intervention (n=68) or control group (n=16) and the intervention lasted nine weeks. Children food neophobia [5] and eating behaviour [6,7] were evaluated by parents at the beginning of the intervention. Mother’s food neophobia [8] was also measured. In each week, children attended an educative session about the vegetable they would eat at lunch (carrot, bell pepper, broccoli, tomato, cucumber, purple cabbage, spinach, arugula and beet), being rewarded with a sticker when eating the vegetable. Assessments of intake and liking were recorded at baseline sessions and after each exposure, using ASTM’s pictorial scales for children [9]. Children at control group were exposed at the same experiment after the intervention group during the subsequent nine weeks.

**Results**

Children from both groups presented high levels of liking for the different vegetables, with this being modulated by the children’s traces of personality and eating behaviour.

**Conclusions**

Exposure to the different vegetables with a playful approach yields high liking scores for a range of vegetables, indicating that such an approach has good potential to overcome vegetables avoidance by young children.

**Trial Registration**

NCT03513081

**References**

1. Rodriguez-Casado A. The Health Potential of Fruits and Vegetables Phytochemicals: Notable Examples. Critical reviews in food science and nutrition. 2016;56(7):1097-107.

2. Slavin JL, Lloyd B. Health benefits of fruits and vegetables. Advances in Nutrition. 2012;3(4):506-16.

3. Pem D, Jeewon R. Fruit and Vegetable Intake: Benefits and Progress of Nutrition Education Interventions- Narrative Review Article. Iranian Journal of Public Health. 2015;44(10):1309-21.

4. Lopes C, Oliveira A, Severo M, Alarcão V, Guiomar S, Mota J, et al. Inquérito Alimentar Nacional e de Atividade Física. Porto: Universidade do Porto; 2017.

5. Pliner P. “Development of measures of food neophobia in children”. Appetite 1994;23(2):147-163.

6. Wardle J, Guthrie C A, Sanderson S, Rapoport L. Development of the Children's Eating Behaviour Questionnaire. J Child Psychol Psychiatry 2001;42(7):963-970.

7. Viana V, Sinde S. O Comportamento Alimentar em Crianças: estudo de validação de um questionario numa amostra portuguesa (CEBQ). Análise Psicológica 2008;1(XXVI):111-120.

8. Pliner P, Hobden K. Development of a scale to measure the trait of food neophobia in humans. Appetite 1992;19(2):105-120.

9. ASTM. E 2299-03. Standard Guide for Sensory Evaluation of Products by Children. United States, ASTM International; 2008.

**Keywords**

Children, Food Neophobia, Playful intervention, Vegetables.

### P180 The limitations of medicinal package leaflets

#### Ana P Izidoro^1^, Patrícia Correia^2^

##### ^1^Farmácia Rainha, 5140-067 Carrazeda de Ansiães, Bragança, Portugal; ^2^Escola Superior de Saúde do Porto, 4200-072 Porto, Portugal

###### **Correspondence:** Ana P Izidoro (anaizi_20@outlook.pt)

**Background**

It is known that, in order to solve most of the existing health problems, it is often fundamental that a pharmacological approach exists. For this reason, patients must be adequately informed about their health status and about the medicines they are using. One of the most important resources of medicines information, available for patients, is the information leaflet. It provides a set of understandable information and should contribute to the appropriate and safe use of information, complementing the information given by health professionals. However, the content appears to be quite complex or too technical and the text is very dense and with a reduced font size, making it intimidating and difficult to read.

**Objectives**

The main objectives of this study are: to identify if users read the information leaflet and whether reading frequency is associated with the importance they attribute to it; and to identify the limitations attributed by the respondents to information leaflets and possible relationships with the socio-demographic characteristics of the population.

**Methods**

This was a transversal and inferential descriptive observational study, which took place between March and November, 2017, based on the application of a questionnaire in the form of an interview to 320 Bragança residents. Various data were collected, particularly with regard to the frequency of reading the information leaflets, the importance attributed to the same or each section, and existing limitations.

**Results**

Regarding the possible relationships between the data obtained, it was verified that there is sufficient statistical evidence to affirm that there is an association between the reading frequency of the information leaflet and the importance attributed to it, but the same cannot be trusted as to the association between the limitations attributed to the information leaflets and the socio-demographic characteristics of the respondents.

**Conclusions**

In sum, these associations and the fact that most respondents have indicated, as the main limitation in reading information leaflets, the use of very technical language, may mean that the information leaflet is not being developed in order to promote reading by part of the youngest or the oldest, which are the groups presenting the major reading difficulties.

**Keywords**

Information leaflet, Illiteracy, Information, Limitations, Adherence to therapy.

### P181 Eating habits and perception of body image in higher education students

#### Helena Catarino^1,2^, Clementina Gordo^1^

##### ^1^School of Health Sciences, Polytechnic Institute of Leiria, 2411-901 Leiria, Portugal; ^2^Center for Innovative Care and Health Technology, Polytechnic Institute of Leiria, 2411-901 Leiria, Portugal

###### **Correspondence:** Helena Catarino (helena.catarino@ipleiria.pt)

**Background**

The transition to higher education is one of the risk factors in the adoption and maintenance of healthy lifestyles [1] and, at this stage, many students develop concerns related to eating habits, their body [2] and body image.

**Objective**

This correlational study had two main objectives, namely, to characterize eating habits and the perception of body image among higher education students; and to relate eating habits to the students’ perception of body image.

**Methods**

We used a questionnaire to collect socio-demographic and anthropometric data, an eating habits scale [3] and the body shape questionnaire [4]. The non-probabilistic convenience sample consisted of 386 students, with mean age of 21.94 years (SD = 5.30), of which 82.9% were females.

**Results**

According to WHO Body Mass Index classification, 72.3% of the students presented normal weight, 14% pre-obesity and 6.2% moderate thinness. From the point of view of the concerns related with body image, the majority, 54.7% presented no distortion of body image. However, the remaining 45.3% presented distortion of body image and among these, 16.6% presented severe distortion. From the point of view of eating habits, the results show that the sample had adequate eating habits. The majority of respondents ate 5 (36.3%) or 4 (32.6%) meals per day and for the four dimensions of the eating habits scale (quality, quantity, variety and food adequacy), the scores varied between a minimum of 2 and a maximum of 5, where the highest mean value was found at the level of food variety (M = 3.62, SD = 0.53) at the lowest mean value observed was found at the level of food quality (M = 3.40, SD = 0.56). The correlations between the dimensions of the eating habits scale (quality, quantity, variety and food adequacy) and students' perceptions of body image are statistically non-significant (respectively, r = 0.099; r = 0.011; r = -0.046; r = -0.038 and p > 0.05).

**Conclusions**

Although the results show that these students have adequate eating habits, it is important to continue to study the food culture of the populations, to identify individuals at risk, to intervene from the point of view of promoting the acquisition of healthy eating habits, and to study the factors underlying the distortion of body image and its influence on students' physical and mental well-being.

**References**

1. Soares A, Pereira M, Canavarro J. Saúde e qualidade de vida na transição para o ensino superior. Revista Psicologia, Saúde & Doenças. 2014;15(2):356-379.

2. Tekin C, Bozkir C, Nese Karakas N, Gunes G. The relation between the body perceptions and eating habits of the students in Inonu University. Anals of Medical Research. 2017:24(1):1-9.

3. Marques A, Luzio F, Martins J, Vaquinhas M. Hábitos alimentares: validação de uma escala para a população portuguesa. Escola Anna Nery Revista de Enfermagem. 2011;15(2):402-409.

4. Pimenta F, Leal I, Maroco J, Rosa B. Validação do Body Shape Questionnaire (BSQ) numa amostra de mulheres de meia-idade. Atlas do 9º Congresso Nacional de Psicologia da Saúde. Lisboa: Placebo Editora; 2011.

**Keywords**

Eating habits, Body image, Students.

### P182 Psychosocial impact of the assistive technologies for mobility on the participation of wheelchair’s users

#### Anabela Martins^1^, João Pinheiro^2^, Patrícia Francisco^1^, Inês Domingues^2^

##### ^1^Physiotherapy Department, Coimbra Health School, 3046-854 Coimbra, Portugal; ^2^Faculty of Medicine, University of Coimbra, 3004-504 Coimbra, Portugal

###### **Correspondence:** Anabela Martins (anabelacmartins@estescoimbra.pt)

**Background**

Recognizing that assistive technology (AT) for mobility play an important role in their users’ participation, it will be useful to rehabilitation professionals and services to assess if the perceived psychosocial impact of such devices and associated services, systems and policies contributes to enhance lifelong capacity and performance [1-3]. To build comprehensive rehabilitation services, information on a person’s experience in every aspect of his/her life is essential, as well as the role of AT on functioning and, particularly, in participation.

**Objective**

To study the psychosocial impact of manual and powered wheelchairs on the participation of their users.

**Methods**

From May to December 2017, sixty AT users (30 powered and 30 manual wheelchairs), aged 28-66 (mean 46.63 ± 10.66) years old, 53.3% female, with mix diagnosis, were interviewed using the Psychosocial Impact of Assistive Devices Scale (P-PIADS), the Activities and Participation Profile related to Mobility (PAPM) and demographics, clinical and questions about AT use and training.

**Results**

The participation profiles revealed that 6.7% of the participants present no restrictions, 20.0% mild, 35.0% moderate and 38.3% severe restrictions in social participation. All subscales (competence 1.50, adaptability 1.45, self-esteem 1.15) and P-PIADS total (1.38) were positive and moderately correlated to the activities and participation profile. Age and type of wheelchair and training of AT as an intervention do not influence statistically the participation, however, number of years on a wheelchair does. In general, training experiences were described as occurring in a clinical setting base, in terms of to enhance the capacity for handing the new device, with less attention to users’ performance in the community and at home, reducing the barriers and promoting a facilitator environment.

**Conclusions**

These results encourage the authors to keep studying the impact of AT for mobility on participation, namely the manual and powered wheelchairs, to develop robust evidence for rehabilitation, giving a contribution to the efforts of the Global Cooperation on Assistive Technology Initiative, and create support for Rehabilitation 2030 A Call for Action4.

**References**

1. Salminen AL, Brandt A, Samuelsson K, Toytari O, Malmivaara A. Mobility devices to promote activity and participation: a systematic review. J Rehabil Med. 2009;41(9):697-706.

2. Lofqvist C, Pettersson C, Iwarsson S, Brandt A. Mobility and mobility-related participation outcomes of powered wheelchair and scooter interventions after 4-months and 1-year use. Disabil Rehabil Assist Technol. 2012;7(3):211-218.

3. Martins A, Pinheiro J, Farias B, Jutai J. Psychosocial Impact of Assistive Technologies for Mobility and Their Implications for Active Ageing. Technologies. 2016;4(3):28.

4. Gimigliano F, Negrini S. The World Health Organization “Rehabilitation 2030: a call for action”. Eur J Phys Rehabil Med 2017;53:155-68.

**Keywords**

Assistive Technologies, Wheelchair, Psychosocial impact, Social participation.

### P183 Unconventional therapies in nursing - innovating the practice

#### Maria I Santos (marirene_mps@hotmail.com)

##### Higher School of Health, Polytechnic Institute of Santarém, 2005- 075-Santarém, Portugal

**Background**

The use of unconventional therapies by Nurses was transmitted to us as nursing students, in various clinical teaching contexts. The disciplinary and professional coherence of these therapies, as well as the therapeutic efficacy they present, legitimizes, from the perspective of nurses, the innovation they constitute in the practice of care.

**Objective**

To understand the process of integrating nonconventional therapies into nursing practice, in the dimensions: identification of therapies in use; meanings assigned and used strategies evaluated by nurses and patients.

**Methods**

Grounded Theory was used, in the constructivist perspective of Kathy Charmaz. The main data collection techniques were the intensive interview and participant observation. Participants were 15 nurses from 9 public hospitals, from the district and central levels, from the north, central and south of the country, and a team of 10 nurses and 17 users of a pain service from an oncology hospital.

**Results**

From the results we point out: nurses innovate their practice of care through the use of nonconventional therapies of environmental, manipulative, mental-cognitive, energetic and relationship nature. The physical, social and normative environments condition the practice of these therapies; the modes of action evidence the importance they confer to the ethical aspects and the (re)combination of several techniques, result in innovative and individualized care. Nurses evaluate these therapies as very effective in various health/illness situations, emphasizing their contribution to well-being.

**Conclusions**

Conclusions and implications of the study: nurses identify a sense of high conceptual and professional coherence of nonconventional therapies with nursing, considering that they innovate and considerably increase the repertoire of practice. The innovation of Nursing practices, through the integration of these therapies, contributes to the well-being of the people cared for and enables them to better manage their health, which is relevant in an aging society.

**Keywords**

Unconventional therapies, Nursing, Innovation practices, Well-being.

### P184 Translation and cultural adaptation of English Modified DABS (EMDABS) Scale for Portuguese language

#### Pedro JC Luz^1,2^ (pedrojcluz@gmail.com)

##### ^1^Hospital Professor Doctor Fernando Fonseca, 2720-276 Lisbon, Portugal; ^2^Lisbon Nursing School, 1600-190 Lisbon, Portugal

**Background**

The occurrence of aggressive episodes by clients in the contexts of mental health settings are recurrent, being imperative to adapt staff interventions to stabilize the psychopathological state of the client, consequently reducing the risk of injury. The de-escalation technique prevents the escalation of aggressiveness, allows a relationship of trust to be established by demonstrating understanding of the client’s problems and concerns [1]. The same authors performed the psychometric validation of the English modified De-Escalating Aggressive Behaviour Scale (EMDABS). This scale aims to evaluate the education/training and de-escalation skills of health professionals, based on seven items: “valuing the client; reducing fear; inquiring about client’s queries and anxiety; providing guidance to the client; working out possible agreements; remaining calm; risky”. The scale revealed a high level of consistency between the seven items of the de-escalation α=0.901.

**Objective**

This study aims to translate and effect the cultural adaptation of the EMDABS scale to the Portuguese language in order to improve nursing de-escalation techniques.

**Methods**

The translation and cultural adaptation of this instrument followed a methodological sequence: translation of the scale into Portuguese; focus group discussion; retroversion to English and pre-testing [2]. The study was authorized by the hospital ethics committee. The participants of the focus-group agreed to participate freely and clarified.

**Results**

The first translation was carried out by two teachers, born in Portugal and fluent in English, with a Degree in Modern Languages and Literatures. Conceptual translation was privileged instead of literal translation, promoting the conservation of simple and accessible language characterized by the original instrument. The translation was evaluated by a focus group (6 professionals) with experience in the field, all participants speak Portuguese and are fluent in English, having evaluated the translation obtained and suggested necessary changes. The translation was then back-translated into the English language, by a Clinical Psychologist, from South Africa, with English native language, fluent in Portuguese, with experience in the field, as well as in the translation of evaluation instruments. This professional had no previous contact with antecedent versions of this instrument. The Pre-testing was applied to a group of ten nurses to evaluate the adequacy of the structure and items of this scale, as well as to validate eventual difficulties in filling it.

**Conclusions**

In the pre-test, the instrument showed a good internal consistency and the participants did not present filling difficulties. The next step is to determine the psychometric properties of the scale.

**References**

1. Mavandadi V, Bieling P, Madsen V. Effective ingredients of verbal de-escalation: validating an English modified version of the De-Escalating Agressive Behaviour Scale. J Psychiatr Ment Health Nurs. 2016;23(6-7):357-68.

2. Waltz C, Strickland O, Lenz E. Measurement in Nursing and Health Research (4th. Ed.). New York: Springer Publishing Company; 2010.

**Keywords**

Aggressive behavior, Cultural adaptation, De-escalation, Mental illness, Nursing interventions.

### P185 Supporting informal caregivers of people dependent in self-care

#### Maria A Dixe^1,2^, Ana CS Cabecinhas^2^, Ana JCF Santos^2^, Marina G Silva^2^, Maura R Domingues^2^, Ana Querido^1,2^

##### ^1^Center for Innovative Care and Health Technology, Polytechnic Institute of Leiria, 2411-901 Leiria, Portugal; ^2^School of Health Sciences, Polytechnic Institute of Leiria, 2411-901 Leiria, Portugal

###### **Correspondence:** Maria A Dixe (maria.dixe@ipleiria.pt)

**Background**

Informal caregivers (IC) are crucial in care provision to dependent patients, frequently aged and suffering from chronic diseases. Therefore, besides helping the dependent people carrying out daily activities, they should also provide them support and assistance in dealing with self-care difficulties.

**Objective**

This quantitative exploratory descriptive study aims to: identify self-care needs in which the person is dependent, and the dependency degree; identify socio-demographic characteristics and ICs support to care for a dependent-person; identify the type, quality of information provided to the IC and the health-professional who delivered the information; assess the perception of ICs regarding their abilities to care for dependent-patients.

**Methods**

An intentional sample of thirty-three dyads of dependent-patients (at least in one self-care daily activity) and their caregivers participated in this study. Participants were referred by the hospital health-care team of a hospital service, at the time of discharge. Dependent-people clinical and sociodemographic data were collected from clinical files. ICs answered a structured interview composed by sociodemographic and professional data, type of support in caring for dependent-people, ability of ICs to care for the dependent-person and to promote self-care. This study was approved by the ethic committee of the hospital where the study was conducted (nº 24/2017).

**Results**

The majority of dependent-persons were female (60.6%) with a mean age of 81.6 (±11.3) years. Regarding self-care activities, the majority of persons were dependent and did not take part in preparing medication (66.7%), preparing food for ingestion (51.5%), nor in obtaining objects for the bath (51.5%). Caregivers were mostly women, mean age of 61.4 (±12.1) years old. Regarding family ties, is mostly a son/daughter (39.4%) or a spouse (33.3%) who takes care of their family member on average for 63.9 (±93) months. Nurses were the main providers of information, nonetheless most of the caregivers were not provided with information about auxiliary equipment (79.3%) nor economic support available (71.0%). ICs feel less competent in caring for a dependent-person regarding ability to transfer. Ability to dress was the area in which the dependent-person presented the highest degree of dependence. Regarding “feeding”, ICs refer the identification of signs of malnutrition and dehydration as the main difficulty.

**Conclusions**

Supporting families in acquiring practical problem-solving skills in every day’s life, in the management of resources and of emotional needs of the dependent-person should be promoted by health-professionals at the beginning of hospitalization, in order to take good care of the family member and of oneself.

**Acknowledgments**

The current abstract is being presented on the behalf of the Help2Care research project. This study was funded by COMPETE 2020 under the Scientific and Technological Research Support System, in co-promotion. We acknowledge CiTechCare, Polytechnic Institute of Leiria, Polytechnic Institute of Santarém, Centro Hospitalar de Leiria, Polytechnic Institute of Castelo Branco and also all other members, institutions and students involved in the project.

**Keywords**

Capacity, Informal caregiver, Self-care, Dependent-person, help2care project.

### P186 Absenteeism on nurses in primary health care

#### Rosa M Freire^1^, Rosário Vieira^2^, Elisabete Borges^1^

##### ^1^Escola Superior de Enfermagem do Porto, 4200-072 Porto, Portugal; ^2^Agrupamento de Centros de Saúde Tâmega II Vale do Sousa Sul, 4560-682 Porto, Portugal

###### **Correspondence:** Rosa M Freire (rosafreire@esenf.pt)

**Background**

The Primary Health Care reform and the introduction of Health Centre Groupings brought changes in the management standards in force until then. The new model care reorganized and changed work contexts and human resources. The new organizational paradigm requires adaptation of the professionals to this new reality. In any situation of change people reacts with resistance in greater or lesser intensity. Also changes in organizations can create resistances that can manifest themselves with absenteeism. In health organizations the absenteeism of nurses constitutes a complex problem and needs to be valued and monitored by nursing managers [1].

**Objective**

To identify the presence and causes of absenteeism and analyse the relationship between absenteeism and demographic and professional variables.

**Methods**

Quantitative, exploratory and cross study was used as methodology. A sociodemographic/professional questionnaire and questions related to absenteeism were used for the collection of data. The sample was composed by 109 nurses working at primary health care units of northern Portugal. The sample was composed by 77.1% of females, 54.1% aged more than 36 years, 72.5% married, 82.6% working on fixed schedules, 51.4% with nearly 14 years of job experience (varied between 7 and 33 years), and 81.7% considered their job as stressful.

**Results**

Of all nurses, 66% admitted to having been absent from service in the last year: 28.4% due to their own illness and 16.5% due to their relatives’ illness. Statistically, there were found significant differences in the association between the variables of to have children (p = 0.007), place of work (p = 0.045) and absenteeism. We also found that nurses with children and those who work in Health Family Units are the ones that are absent from work most frequently.

**Conclusions**

We believe that the results can induce the development, implementation and evaluation of preventive measures, aiming at the promotion and protection of nurses' health. In this regard, they can be crucial for the management of human resources and for strategic planning of the ACeS, in particular, in grounded decision-making.

**References**

1. Sancinetti TR, Soares AVN, Lima AFC, Santos NC, Melleiro MM, Fugulin FMT, et al . Nursing staff absenteeism rates as a personnel management indicator. Rev. esc. enferm. USP. 2011;45(4):1007-1012.

**Keywords**

Nurse, Absenteeism, Primary health care, Management in nursing.

### P187 Assessment and intervention in a family with a care dependent person and mental illness: a case study

#### Inês Esteves, Isabel Bica

##### Health School, Polytechnic Institute of Viseu, 3504-510 Viseu, Portugal

###### **Correspondence:** Isabel Bica (isabelbica@gmail.com)

**Background**

Mental illness can have profound repercussions in family dynamics, since it is a serious illness and generates great stress. The assessment and intervention of a family with psychosocial problems requires the nursing team to use models that allow the design of care oriented both for data collection and for the planning of interventions in domiciliary context.

**Objective**

To analyse the care needs in a family with mental illness using the Dynamic Model of Family Assessment and Intervention (MDAIF).

**Methods**

The case study focused on the process of family intervention developed with a family in a domiciliary context in Primary Health Care, according to MDAIF. The following data collection instruments were applied: genogram, ecomap, FACES II, Graffar and Family Apgar Scales.

**Results**

The family consists of a middle-aged couple, childless, lower middle class (grade IV, Graffar, 1956). The woman is the main care provider of her husband, with mental illness. They have an extensive network of community support, namely in Social Solidarity Institutions, Recreational Associations, the extended family and the Community Care Unit (health team). As a couple, they show good cohesion and adaptability facing everyday problems (Faces II Scale) and perceive their family as highly functional (score 10 - Apgar Scale), despite the perception of communicational difficulties and emotional expression. After the interventions of the nursing team, in the different dimensions of the MDAIF, gains were obtained in the knowledge and capacity of income management, heating system of the residential building, management and knowledge about the mental illness, communication and marital satisfaction and the role of caregiver. In this way, the caregiver has been trained in knowledge and skills in the field of care for the dependent person and with mental pathology, in order to maximize their health, as well as in the care and importance of the satisfaction of her own needs.

**Conclusions**

The valuation of caregiver perspectives is essential for the adjustment of the formal responses of the nursing team. Empowerment of family caregivers - as well as being able to express themselves and be an active element in the healthcare process, as well as the articulation of formal and informal responses constitute challenges for health professionals. The support provided by the health team to the caregiver and dependent patient has proved to be effective not only in improving family functioning but also in reducing stress, burnout and isolation of the caregiver.

**Keywords**

Caregiver, Family, Nursing, Primary health care.

### P188 Elderly people, physical therapy services and human resources: current and future challenges

#### Carla Leão^1,2^ (carlaleao@sapo.pt)

##### ^1^Escola Superior de Saúde, Universidade Atlântica, 2730-036 Barcarena, Portugal; ^2^Instituto Português de Relações Internacionais, 1000-155 Lisboa, Portugal

**Background**

The aging process implementation in Portugal, and the consequent increase in the number of elderly people is evident and constitutes an increasing process. Undeniably, the elderly people are the main users of health services and specifically physiotherapy services. The economic and financial crisis that affected Portugal, has imposed restrictions on the health system and specifically on the National Health Service (NHS) [1]. Considering this scenario, we have put as a guiding question: *Are, in Portugal, physiotherapy health services and human resources included in the health system at NUTS III regional level, and essentially in the NHS, proportional to the number of elderly people and respond to their current and future needs?* [NUTS III - Nomenclature of territorial units for statistics (NUTS) with level III corresponding to the territorial division constituted by 30 regions, according to the division of 2013].

**Objective**

We aim to understand the current and future numbers of the elderly people at the regional level (NUTS III); to know the current number of physiotherapy services and human resources at the same regional level; to prospective the future physical therapy services and human resources, considering the health political trends related to services and human resources; and to assess if these services and human resources respond to the needs of the current and future elderly people.

**Methods**

For this purpose, we performed a statistical analysis at the regional level NUTS III, considering the indicators - number of elderly people and physical therapy services and human resources. We used the data from the Statistics National Institute, the Health Regulatory Body, the Portuguese Physiotherapists Union and the Portuguese Association of Physiotherapists.

**Results**

The results showed that at the regional level (NUTS III), physiotherapy services and human resources are scarce and there is no proportionality between the number of elderly people and the physiotherapy services and human resources. With the increasing number of elderly people and the restrictive political trend, we assume that, if the political trends continue, proportionality will remain non-existent.

**Conclusions**

In this way, we conclude that current and future physiotherapy services and human resources do not respond to the needs of the current and future elderly population, and there is a need to adapt physiotherapy services and human resources to the current and future demographic reality, in order to achieve better levels of healthy life expectancy.

**References**

1. Rodrigues T F, Martins M R O (Coordenadores). Envelhecimento e saúde. Prioridades políticas num Portugal em mudança. Lisboa. CEPESE/Instituto Hidrográfico. 2014.

**Keywords**

Portugal, Elderly people, Physical therapy services, Physical therapy human resources.

### P189 Ultrasound evaluation of subcutaneous tissue and relation with the evaluation of fat mass in women with cellulitis

#### Alexandra André, João P Figueiredo, Óscar Tavares, Catarina Bulamarqui, Catarina Frade

##### Escola Superior de Tecnologia da Saúde de Coimbra, Instituto Politécnico de Coimbra, 3046-854 Coimbra, Portugal

###### **Correspondence:** Alexandra André (alexandra.andre@estescoimbra.pt)

**Background**

Ultrasound (US) is an easily accessible imaging modality that allows the evaluation of various structures. Despite the potential of this technique there are a few studies referring to subcutaneous tissue analysis with US. This allows the evaluation of altered tissues such as gynoid lipodystrophy, a very common alteration in female. the better the characterization, location, definition of the affected areas and the degree of severity of LDG, the greater the success of treatment. In this study US and densitometry was performed to evaluate the thickness of the subcutaneous tissue and the body mass composition, such as lean mass and fat mass.

**Objective**

The aim was, by using two different images modality, to evaluate the subcutaneous tissue in normal weight and overweight, as well as the possible relation with the percentage of fat in women with cellulite.

**Methods**

The study was performed in 25 individuals aged between [18-60] years old. Some inclusion and exclusion criteria were done. The images were performed in the midpoint between the great trochanter and the medial aspect of the gluteal region and the thighs in the proximal third, particularly in the proximal and lateral portion of the thigh, between the great trochanter and the knee joint.

**Results**

Spearman's correlation tests for a correlation between bone mineral density and fat mass with subcutaneous cell tissue thickness at a rho = 1 p-value must be less than or equal to 0.005%. Thus, with the results obtained, we verified that there is no possible correlation between measurements of bone mineral density, fat mass and subcutaneous cellular tissue of the gluteal and thigh regions. There were no significant differences in the variances between groups.

**Conclusions**

Ultrasonography has sensitivity to evaluate subcutaneous cellular tissue as well as deformations that may exist in it. We concluded that the percentage of fat measured by bone densitometry does not correlate with the thickness of subcutaneous tissue, with the percentage of fat being higher in overweight women but the thickness of subcutaneous tissue varies from woman to woman. The only possible relationship is between the bone mineral density and the thickness of the subcutaneous tissue of the right gluteus that leads to force that the greater the thickness the subcutaneous cellular tissue, the greater the bone mineral density, because although they are structurally different both respond to the stimuli of atrophy and hypertrophy.

**Keywords**

Subcutaneous tissue, Ultrasound, Dexa.

